# Integrated analysis of lncRNA-associated ceRNA network identified potential regulatory interactions in osteosarcoma

**DOI:** 10.1590/1678-4685-GMB-2019-0090

**Published:** 2020-05-20

**Authors:** Yongwei Wang, Yaxian Gao, Sen Guo, Zhihong Chen

**Affiliations:** 1 Department of Anatomy, Basic Medical Institute, Chengde Medical College, Chengde 067000, Hebei, China.; 2 Department of Immunology, Basic Medical Institute, Chengde Medical College, Chengde 067000, Hebei, China.

**Keywords:** Osteosarcoma, differentially expression mRNA, differentially expression miRNA, competing endogenous RNAs network, lncRNA

## Abstract

This study aimed to identify potential therapeutic targets in osteosarcoma (OS) through the network analysis of competing endogenous RNAs (ceRNAs). The differentially expressed miRNAs (DEMIs) and mRNAs (DEMs) were identified between OS cell lines and human mesenchymal stem cells (hMSCs) from the data deposited under GSE70415 using limma package. Functional analysis of DEMs was performed using DAVID and clusterProfiler to identify significantly enriched Gene Ontology biological processes and KEGG pathways, respectively. The DEMI-DEM interaction network was constructed using Cytoscape. LncRNA–miRNA interactions were predicted using starBase database. The ceRNA regulatory network was constructed by integrating mRNAs, miRNAs, and lncRNAs, and functional enrichment analysis was performed for the genes involved. The analysis revealed a total of 326 DEMs and 54 DEMIs between OS cells and hMSCs. We identified several novel therapeutic targets involved in the progression and metastasis of OS, such as *CBX7*, *RAD9A*, *SNHG7* and miR-34a-5p. The miRNA, miR-543 (target gene: *CBX7*) was found to be associated with the pathway Mucin type O-glycan biosynthesis. Using the ceRNA network, we established the following regulatory interactions: NEAT1/miR-543/CBX7, SNHG7/miR-34a-5p/RAD9A, and XIST/miR-34a-5p/RAD9A. *CBX7*, *RAD9A*, lncRNA *SNHG7*, miR-543, and miR-34a-5p may be explored as novel therapeutic targets for treatment of OS.

## Introduction

Osteosarcoma (OS) is the most commonly diagnosed primary malignant bone tumor in adolescents (He *et al.*, 2014).The age-adjusted worldwide incidences per million of OS in the age group of 0-24 is 4.4, with the incidence rate in males being more than females in every race (male:female = 1.34:1) (Mirabello *et al.*, 2009). Though the 5-year overall survival rate for OS is 68% (Ottaviani and Jaffe, 2009), it is highly metastatic (Marina *et al.*, 2004), with lung being the most common site of metastasis (Fitzgerald *et al.*, 1973). The complex molecular mechanisms associated with progression and metastasis of OS is not clear and is challenging to treat. Hence, it is important to identify the underlying molecular mechanisms associated with the development and metastasis of OS and identify better therapeutic targets for its treatment.

Long non-coding RNAs (lncRNAs), a class of nonprotein-coding RNA transcripts longer than 200 nucleotides (Morris and Mattick, 2014), are known to be involved in many biological processes, such as transcriptional regulation, cell proliferation, metastasis and tumorigenesis (Tang *et al.*, 2016; Wei *et al.*, 2016). Earlier studies have implicated that lncRNAs may be important factors in the malignant transformation of the tumors, and can be considered as novel biomarkers (Yarmishyn and Kurochkin, 2015). Similarly, micro RNAs (miRNAs), a class of short non-coding RNAs, play important regulatory roles in many biological processes, such as cell migration, apoptosis, cell differentiation and oncogenesis, by targeting the mRNA molecules (Wang *et al.*, 2015). It is well known that lncRNAs act as competing endogenous RNA (ceRNA) and affect the level of mRNAs by negatively regulating miRNA expression (Salmena *et al.*, 2011; Liu *et al.*, 2014; Cao *et al.*, 2015; Wang *et al.*, 2016). For example, the lncRNA *TUG1* acts as a ceRNA for miR-335-5p and promotes OS cells migration and invasion (Yong *et al.*, 2017). Uzan *et al*. reported that high expression of the lncRNA HULC promotes metastasis and is associated with poor prognosis of OS (Uzan *et al.*, 2016). The up-regulated lncRNA *HNF1A-AS1* promotes proliferation and metastasis of OS cells by activating Wnt/catenin signaling pathway (Zhao H *et al.*, 2016a). However, the complex mechanisms associated with metastasis of OS has not been fully understood. The integrative analysis can help us in better understanding of related genes, functions, and the complex mechanisms associated with the development and metastasis of OS.

The aim of the present study is to understand the molecular mechanisms by exploring the regulatory interactions through ceRNA regulatory network, and identify potential therapeutic targets in OS using bioinformatics analysis.

## Material and Methods

**Figure 1:**
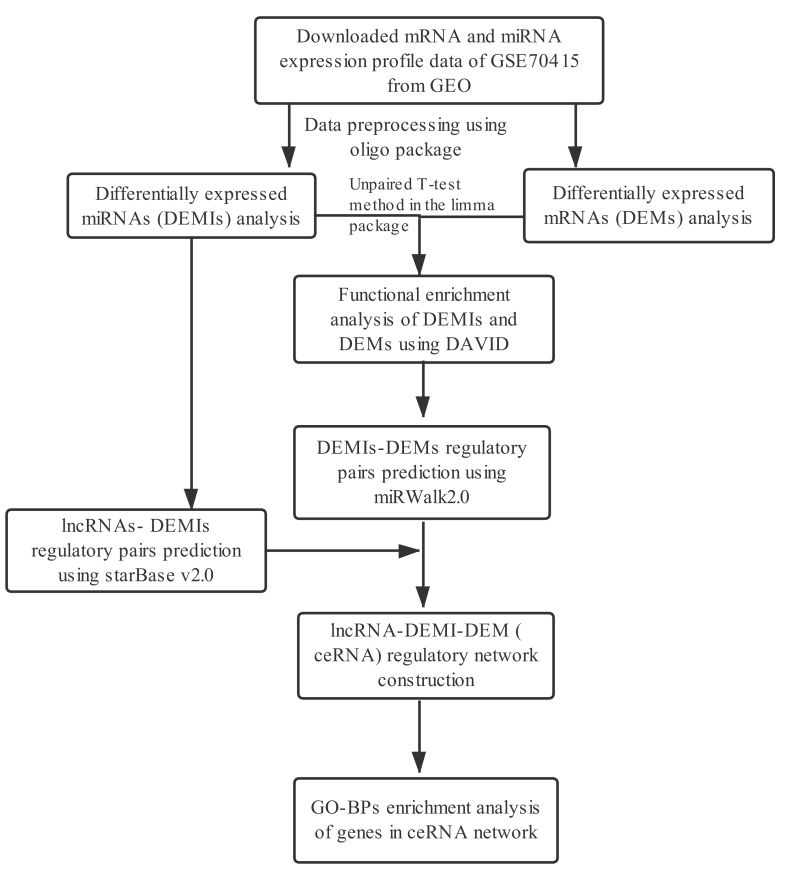
Overall strategy and analyses followed in the current study.

The flow of the analysis steps used in this study is shown in Figure 1.

### Data source and preprocessing

The mRNA and miRNA expression profiles were downloaded from the Gene Expression Omnibus (GEO) series GSE70415 (Wang H *et al.*, 2017a). This series included two subseries: GSE70414 and GSE70367 corresponding to mRNA and miRNA expression data, respectively. The original study used five OS cell lines and t human mesenchymal stem cells, (hMSCs) as control. The mRNA expression data was generated using Affymetrix Human Genome U133 Plus 2.0 (HG-U133_Plus_2) array (GPL570), and the miRNA expression data was generated using Affymetrix Multispecies miRNA-3 (miRNA-3) array (GPL16384). The original study published by Wang *et al.* (2017a) corresponding to the data deposited in GSE70415, mainly focused on miRNA-mRNA regulatory interactions, however, the interactions between lncRNAs and miRNAs were studied.

The R-based oligo package (Irizarry *et al.*, 2003) was used for data preprocessing, including background correction, normalization and summarization (median-polish). The Robust Multichip Average (RMA) method in oligo package was used to implement background correction with treating the perfect-match probe intensities as a convolution of noise and true signal, followed by normalization. The annotation file was used to map the probes in the mRNA matrix to gene symbols. When multiple probes corresponded to the same gene symbol, summarization at the probeset level was performed, and the mean expression values across multiple probe-sets were considered as the final expression value for that gene. Since the miRNA platform was composed of multi-species probes, the expression values of probes corresponding to human miRNA were selected for further analysis.

### Identification of differentially expressed mRNAs and miRNAs between OS cell lines and hMSCs

The differentially expressed miRNAs (DEMIs) and mRNAs (DEMs) were identified using the unpaired T-test method implemented in limma package (Smyth, 2005). The DEMIs and DEMs with a P-value < 0.05 and log2 fold change (log2FC) > 1 were considered as statistically significant. The heatmaps and volcano plots of the significant DEMIs and DEMs were constructed using the R-based pheatmap (Wang *et al.*, 2014) and ggplot2 (Warnes *et al.*, 2009) software packages, respectively.

### Regulatory pair prediction and functional enrichment analysis of DEMIs and DEMs

The DEMI probes were annotated using miRBase version 21 to identify the corresponding miRNAs (updated list of miRNAs using miRBase version 22.1 are shown in Table S1). The mRNA targets for the top (based on log2FC) 10 up- and down-regulated DEMIs were predicted using miRWalk2.0 tool (Dweep and Gretz, 2015). Further, the miRNA-target pairs showing consensus across five prediction methods integrated in miRWalk (miRanda, miRDB, miRMap, RNA22, and TargetScan) were considered as the for further analysis. Among the DEMI targets, only those that overlapped with the DEMs were considered for further analysis.

The Kyoto Encyclopedia of Genes and Genomes (KEGG) (Ogata *et al.*, 1999) pathways enriched by the miRNA targeted DEMs were analyzed using the cluster-profiler package in R software (Yu *et al.*, 2012). The KEGG pathways with adjusted P-value < 0.05 were considered as statistically significant. Database for Annotation, Visualization and Integrated Discovery (DAVID, version 6.8) (Huang *et al.*, 2008) was used to obtain enriched Gene Ontology biological processes (GO-BPs) (Ashburner *et al.*, 2000) for the DEMs targeted by the DEMIs. The GO-BP terms with a gene count ≥ 2 and P-value < 0.05 were considered as significantly enriched.

### Construction of DEMI-DEM network

The regulatory network between DEMIs and DEMs was constructed by using the Cytoscape  software, version 3.2.0 (Shannon *et al.*, 2003). The topological properties of the network were analyzed, and the results were presented with the Degree Centrality (DC). The nodes with higher DC scores were considered as hub proteins.

### Construction and functional enrichment analysis of genes involved in lncRNA-miRNA-mRNA regulatory network

starBase database provides the most comprehensive CLIP-Seq experimentally supported lncRNA-miRNA interactions. It contains 35,459 human miRNA-lncRNA interactions (Li *et al.*, 2014). The regulatory interactions between the lncRNAs and differentially expressed miRNAs were extracted from the starBase database based on the following criteria: medium stringency ≥ 2; number of cancer types ≥ 1; clade: mammal; genome: Human; assembly: hg19.

Using the lncRNA-DEMI and DEMI-DEM regulatory interactions, the lncRNA and DEM that were regulated by the same DEMI were identified, and were used for the construction of lncRNA-DEMI-DEM (lncRNA-miRNA-mRNA) regulatory network, also known as ceRNA regulatory network.

The enrichment analysis of the genes involved in ceRNA network was performed to obtain GO-BP and annotations with a P-value < 0.05 and gene count ≥ 2 were considered for further analysis.

## Results

### Identification of DEMs and DEMIs between OS cell lines and hMSCs

**Figure 2:**
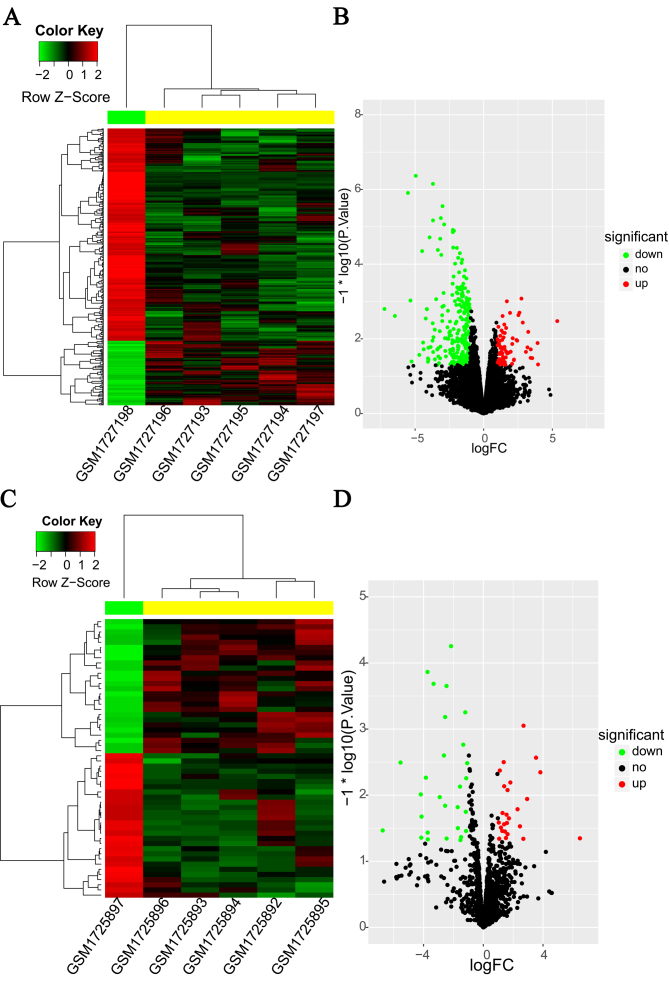
Heatmap and volcano plot of differentially expressed mRNAs (A & B, respectively) and miRNAs (C & D, respectively) between osteosarcoma (OS) cell lines and human mesenchymal stem cells (hMSCs). In the heatmap, the red color indicates up-regulation, and the green color indicates down-regulation.

The DEMs and DEMIs between OS cell lines and hMSCs were derived using the microarray dataset GSE70414. A total of 326 DEMs (Table S2), including 76 up- and 250 down-regulated were identified, and are shown in the double-level clustering heatmap (Figure 2A) and volcano plot (Figure 2B). The heatmap shows independent clustering of OS cell lines and hMSCs. Similarly, a total of 54 DEMIs, including 26 up- and 28 down-regulated (Figure 2C & D) were identified. The complete list of DEMs and DEMIs can be found in Table S1 and Table S3, respectively.

### Functional enrichment analysis of DEMs and DEMIs

**Figure 3:**
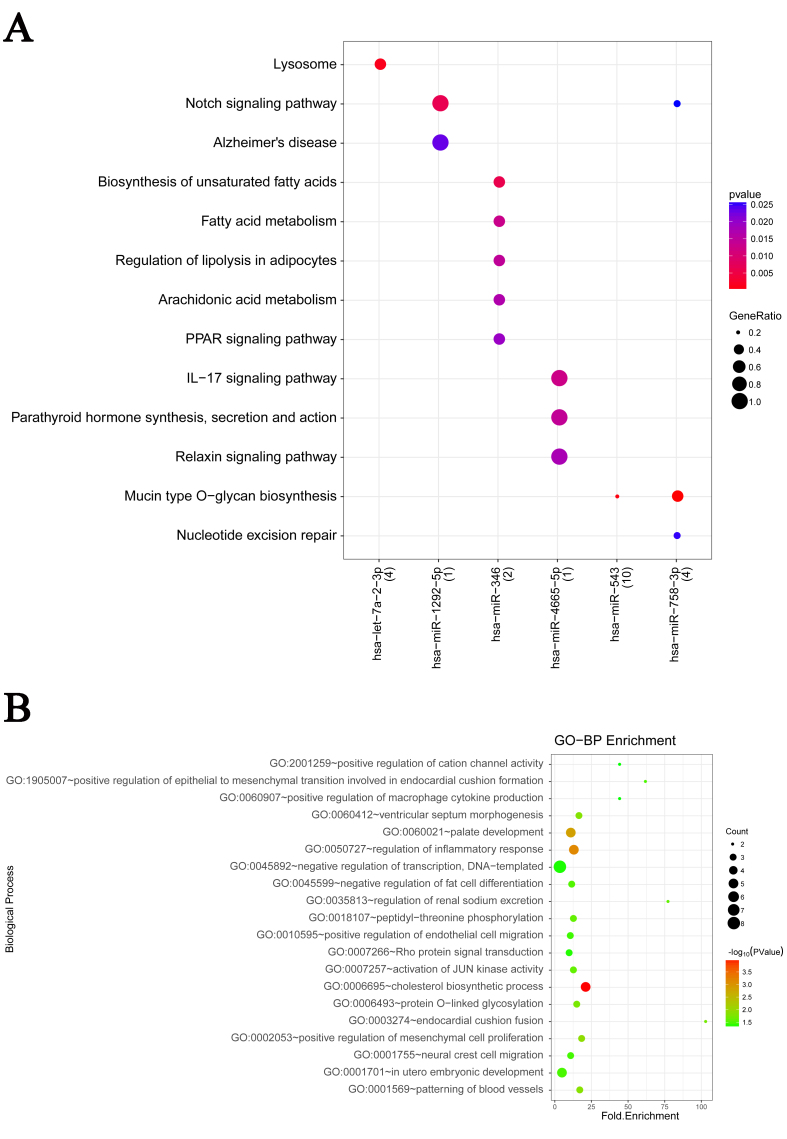
Functional enrichment analysis of differentially expressed mRNAs and miRNAs between osteosarcoma (OS) cell lines and human mesenchymal stem cells (hMSCs). A: representative enriched pathways by DEMIs; B: representative enriched biological processes by DEMs.

Functional enrichment analysis was performed to obtain GO-BPs and pathways influencing the development of OS. As shown in Figure 3A, a total of six miRNAs were found to be significantly enriched in 18 KEGG pathways. For example, has-miR-543 was found to be associated with “Mucin type O-glycan biosynthesis” pathway. The GO-BP enrichment analysis showed *RPS6KA5* to be significantly related to “in utero embryonic development (GO: 0001701)”, *EDNRA* to be significantly related to “negative regulation of transcription, DNA-templated (GO: 0045892)” and *TNFSF4* to be significantly related to “regulation of inflammatory response (GO: 0050727)”, Figure 3B.

### Analysis of DEMI-DEM regulatory network

**Figure 4:**
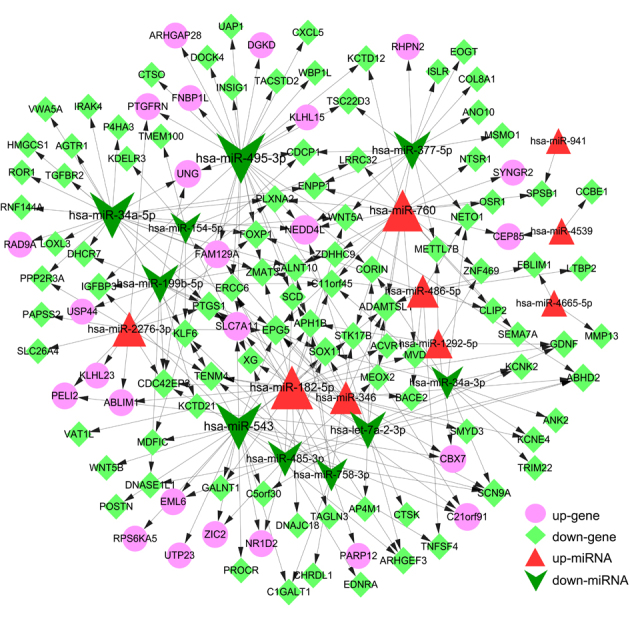
Regulatory network showing mRNA-miRNA interactions. Pink circles: Up-regulated mRNA;Light green diamonds: Down-regulated mRNA; the Red triangles: Up-regulated miRNA; Green arrows: Down-regulated miRNA.

For the 54 DEMIs obtained from the microarray analysis, potential target genes consistent across multiple target prediction tools. The interactions between the target DEMIs and DEMs were used to construct a miRNA-gene regulatory network (e.g., has-miR-543-CBX7, miR-34a-5p-RAD9A and has-miR-495-3p-DGKD. The DEMI-DEM regulatory network (Figure 4), had a total of 139 nodes and 238 interactions (edges). Top five miRNAs (miR-495-3p, miR-543, miR-34a-5p, miR-182-5p and miR-760) and genes (*EPG5*, *ADAMTSL1*, *CDC42EP3*, NEDD4L, and PTGS1) identified based on the degree ranking are shown in Table 1. 

**Table 1:** The top 5 differentially expressed miRNAs and differentially expressed genes between osteosarcoma cells and human mesenchymal stem cells

miRNA	Description	Degree	Gene	Description	Degree
hsa-miR-495-3p	down	29	*EPG5*	down	6
hsa-miR-543	down	25	*NEDD4L*	up	5
hsa-miR-34a-5p	down	24	*ADAMTSL1*	down	5
hsa-miR-182-5p	up	21	*CDC42EP3*	down	5
hsa-miR-760	up	19	*PTGS1*	down	5

miRNAs, microRNAs.

### Analysis of ceRNA regulatory network

**Figure 5:**
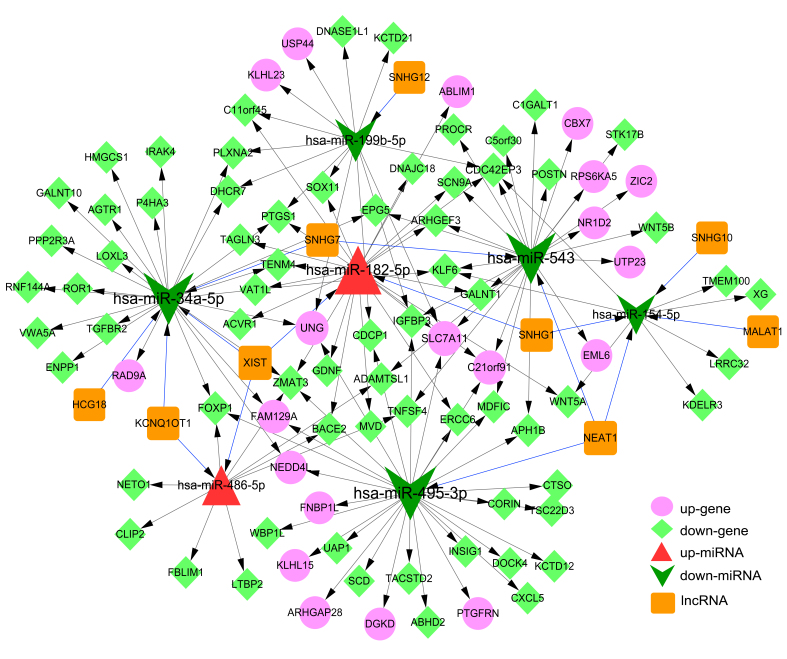
Competing-endogenous RNA regulatory network. Pink circles: Up-regulated mRNA; Light green diamonds: Down-regulated mRNA; Red triangles: Up-regulated miRNA; Green arrows: Down-regulated miRNA; Orange squares: LncRNA. Blue lines (edges): LncRNA-miRNA interactions; Gray lines (edges): miRNA-mRNA interactions.

To obtain miRNA and lncRNA interactions, the lncRNAs regulating 19 miRNAs present in the DEMI-DEM network were predicted (Supplemental File S1). The miRNA-lncRNA interaction network consisted of 16 nodes, including seven miRNAs and nine lncRNAs. Examples of lncRNA-miRNA interactions include NEAT1-has-miR-543, SNHG7-has-miR-34a, and XIST-miR-34a-5p. Finally, the lncRNA-miRNA and miRNA-gene regulatory networks were merged to obtain the integrated ceRNA regulatory network. The ceRNA network had 145 pair-wise interactions among 105 nodes, consisting of two up-regulated and five down-regulated miRNAs, 20 up-regulated and 69 down-regulated DEMs, and nine lncRNAs (Figure 5 and Table S4). Top five miRNAs, mRNAs and lncRNAs in the network are listed in Table 2.

**Table 2:** The top 5 miRNAs, lncRNAs, and mRNAs in ceRNA regulatory network

miRNA	Description	Degree	Gene	Description	Degree	LncRNA	Degree
hsa-miR-495-3p	down	30	*CDC42EP3*	down	4	NEAT1	3
hsa-miR-34a-5p	down	28	*SLC7A11*	up	4	XIST	3
hsa-miR-543	down	27	*ZMAT3*	down	4	SNHG1	2
hsa-miR-182-5p	up	23	*KLF6*	down	3	SNHG7	2
hsa-miR-199b-5p	down	15	*C2lorf91*	up	3	KCNQ1OT1	2

lncRNA, long non-coding RNA; ceRNA, competing endogenous RNAs.

**Figure 6:**
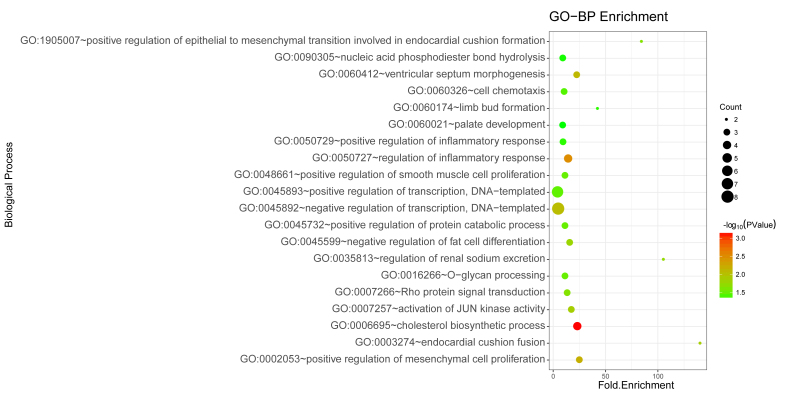
Enrichment of Gene ontology biological processes by differentially expressed mRNAs involved in the ceRNA regulatory network. Count represents the number of genes associated with the annotation

Function enrichment analysis of the network identified 20 significant GO-BP annotations. As shown in Figure 6, the DEMs were found to be significantly related to “positive regulation of transcription, DNA-templated (GO: 0045893)” as well as “negative regulation of transcription, DNA-templated (GO: 0045892)”.

## Discussion

In the current study, a total of 326 DEMs and 54 DEMIs were identified by re-analyzing the dataset, GSE70415 using limma package. Several potential targets that may be involved in the progression and metastasis of OS were identified, e.g., *CBX7*, *RAD9A*, SNHG7 (lncRNA) and has-miR-34a-5p. In addition, few potential regulatory interactions, such as NEAT1/ miR-543, miR-543/CBX7, SNHG7/ miR-34a-5p, miR-34a-5p/RAD9A, and XIST/miR-34a-5p were identified in the OS cells through the ceRNA network. The dataset GSE70415 had been analyzed in a previous study (Wang H *et al.*, 2017a); and a total of 3,856 DEMs and 250 DEMIs were identified by using GCBI. When compared, the results were found to be similar between the earlier and current study. For example, the key gene POSTN discussed in the earlier study was also found to be highly downregulated in our study. Similarly, the miRNAs, miR-34a, miR-182, miR-493 and miR-29 were found to be differentially expressed in the previous study. However, there were significant differences observed between the current and the previous study. For instance, the GO annotations including extracellular matrix organization, small molecule metabolic process, cell adhesion, and KEGG pathways including PI3K-Akt signaling pathway and metabolic pathways were found to be significantly enriched in the previous study, however not in the current study. One of the reasons behind this could be the method used for analysis. Additionally, we performed lncRNA and ceRNA network analysis, which was not performed by the previous study.

Nuclear enriched abundant transcript 1 (*NEAT1*) is a lncRNA transcribed from the multiple endocrine neoplasia locus. Study by Zhao *et al*., have shown that *NEAT1* acts as an oncogene and is associated with the development of OS (Zhao H *et al.*, 2016b). Knockdown of *NEAT1* is known to inhibit tumor cells proliferation and metastases and induce cell apoptosis (Wang H *et al.*, 2017b). In addition, it promotes oncogenic proliferation by affecting the epigenetic structure of target gene promoters and driving their transcription in prostate cancer (Chakravarty *et al.*, 2014). In the current study, miR-543 was predicted to be a target of *NEAT1*. The molecular mechanism of this interaction has not been characterized in the progression of OS. The expression of miR-543 inhibits epithelial-mesenchymal transition during tumor metastasis (Haga and Phinney, 2012). Over-expression of miR-543 inhibits tumor cell proliferation and has been reported to reduce the migration and invasion of tumor cells in endometrial cancer (Bing *et al.*, 2014). Based on the published studies and findings from the current study, it can be hypothesized that the lncRNA *NEAT1* promotes proliferation and metastasis of OS cells by sponging the miRNA, miR-543.

Chromobox 7 (*CBX7*, also known as chromobox homolog 7), a polycomb family protein, and a component of the polycomb repressive complex 1, is known to extend the lifespan of some normal human cells. Reportedly, the over-expressed *CBX7* acts as an oncogene in the gastric cancer (Zhang *et al.*, 2010). Further, studies have shown high expression of *CBX7* in various prostate cancer cell lines (Bernard *et al.*, 2005) and clear cell adenocarcinoma of the ovary (Shinjo *et al.*, 2014). In addition, another member of the polycomb family, chromobox 4 (*CBX4*, also known as chromobox homolog 4) is known to be involved in progression of OS, and over-expression of *CBX4* is associated with advanced clinical stage of OS (Yang *et al.*, 2016). However, the role of *CBX7* in OS progression has been not reported. Based on the existing evidences for *CBX4*, we presume that *CBX7* may act as an oncogene during the development of OS. In addition, based on lncRNA regulatory interactions, we believe that the lncRNA *NEAT1* may affect the expression of *CBX7* by competing for miR-543 during the progression of OS.

Small nucleolar RNA host gene 7 (*SNHG7*) belongs to the long non-coding RNA class. Reportedly, SNHG7 contributes to the growth and metastasis of glioblastoma by suppressing miR-5059 and activating the *wnt*/β-catenin signaling pathway (Ren *et al.*, 2018). Additionally, in prostate cancer, *SNHG7* promotes cell proliferation via cyclin D1 by modulating miR-503 (Qi *et al.*, 2018). Further, *SNHG7* sponges miR-34a and induces over-expression of *GALNT7*, which in turn results in the progression of colo-rectal cancer (Li *et al.*, 2018). In our study, miR-34a-5p was predicted to be a target of *SNHG7*, for the first time in OS cell lines. It was reported as a tumor suppressor in for the first time in neuroblastoma (Welch *et al.*, 2007). The over-expression of miR-34a has been reported to inhibit cell growth and induce differentiation of glioma stem cells in human glioma tumors (Guessous *et al.*, 2010). The, lncRNA *C2dat1* has been shown to promote cell proliferation, metastasis and infiltration of OS cells by targeting miR-34a-5p (Jia *et al.*, 2018). Thus, our results suggest that lncRNA *SNHG7* may promote cell proliferation and metastasis of OS cells by suppressing miR-34a-5p.

RAD9 checkpoint clamp component A (*RAD9A*, also known as *RAD9*) is a cell cycle checkpoint protein associated with cell cycle arrest and DNA damage repair. It is an oncogene and is known to be regulated by DNA methylation, and its chromosome locus 11q13 has been reported to be amplified in breast cancer (Cheng *et al.*, 2005). A study by Lieberman *et al*., has shown abnormal over-expression of *RAD9* in approximately 45% of clinically detected prostate tumors, and its significant correlation with tumor stage (Lieberman *et al.*, 2018). However, the role of *RAD9A* in OS is has not been explored. In the current study, *RAD9A* was predicted to be a target of miR-34a-5p. A study by Wang *et al.* has shown that over-expression of miR-34a-5p inhibits cell proliferation and metastasis of cervical cancer cells and promotes cell apoptosis (Wang X *et al.*, 2017c). In colon cancer cells (HCT116), the expression of miR-34a-5p has been reported to induce apoptosis, cell cycle arrest at G1 stage and transcription of P53 (Gao *et al.*, 2015). Hence, based on previous reports and the results from the current study, it can be implicated that the *RAD9A* regulation by miR-34a-5p may affect the progression of OS through cell cycle regulation. In summary, the lncRNA *SNHG7* and the protein coding gene *RAD9A* may play an important role in the progression of OS by competing with each other for miR-34a-5p.

X inactivate-specific transcript (*XIST*) is a well-known lncRNA. The high expression of XIST is associated with cell proliferation and poor prognosis of OS (Li *et al.*, 2017). In our ceRNA network, miR-34a-5p was predicted to be a target of XIST. This regulatory interaction has been reported in multiple cancers, such as pancreatic cancer (Sun Z *et al.*, 2018), colon cancer (Sun N *et al.*, 2018), and human nasopharyngeal carcinoma (Song *et al.*, 2016). However, the underlying regulatory mechanisms of XIST have been hardly reported in the progression of OS. The current study indicates that interaction of *XIST* and miR-34a-5p may regulate downstream mRNA levels associated with progression of OS.

The interaction of different RNA molecules explored in the current study may provide insights into novel targets and underlying molecular mechanisms associated with development and metastasis of OS. However, the current study has limitations. The results obtained in the current study including, genes and their interactions need to be validated using *in vivo* and *in vitro* experimental approaches to confirm their regulatory roles. Further, the number of samples considered by the dataset used in the current study is less.

In conclusion, *CBX7*, *RAD9A*, *SNHG7* lncRNA and has-miR-34a-5p may be explored as novel therapeutic targets for the treatment of OS. Further, the lncRNA *NEAT1* may be involved in promoting the proliferation and metastasis of OS cells by sponging miR-543. The lncRNA *SNHG7* and the protein coding gene *RAD9A* may play a role in the progression of OS through their competitive interaction with miR-34a-5p. Finally, the interaction between the lncRNA *XIST* and miR-34a-5p may be important in understanding molecular mechanisms associated with OS.

## Supplementary material

Supplemental File S1 Identifiers and FASTA sequences of lncRNAs

Table S1 Updated list of miRNAs using miRBase version 22.1

Table S2 Standardization and annotation mRNA data

Table S3 Standardization and annotation miRNA data

Table S4 Interactions present in the ceRNA network
