# Supplementary material for: Integrated analysis of lncRNA-associated ceRNA network identified potential regulatory interactions in osteosarcoma
Source: Genet Mol Biol. 2020 May 20;43(2):e20190090. doi: 10.1590/1678-4685-GMB-2019-0090 (PMC7252519; doi:10.1590/1678-4685-GMB-2019-0090)
Supplement: Supplemental File S1 [file 1415-4757-GMB-43-2-e20190090-s1.pdf]

Genetics and Molecular Biology 43, 2, (2020)

# Supplementary Material to “Integrated analysis of lncRNA-associated ceRNA network identified potential regulatory interactions in osteosarcoma”

**Supplemental File S1.** Identifiers and FASTA sequences of lncRNAs

>MALAT1
>ENST00000619449.2|ENSG00000251562.8|OTTHUMG00000166322.5|OTTHUMT00000473534.2|AP000769.3-004|MALAT1|5340|
GACGCAGCCCCACCGGTTGCGCAGTCCCTCCCCGCCCCCGCTCTCCCCTCCGCAGCCTGCAGCCCGAGACTTCTGTAAAGGACTGGGGCCCCGCAAC
TGGCCTCTCCTGCCCTCTTAAGCGCAGCGCCATTTTAGCAACGCAGAAGCCCGGCGCCGGGAAGCCTCAGCTCGCCTGAAGGTGGTAAACTATACCTA
CTGTCCCTCAAGAGAACACAAGAAGTGCTTTAAGAGGCGGCGGAAGGTGATCGAATTCCGGTGATGCGAGTTGTTCTCCGTCTATAAATACGCCTCGC
CCGAGCTGTGCGGTAGGCATTGAGGCAGCCAGCGCAGGGGCTTCTGCTGAGGGGGCAGGCGGAGCTTGAGGAAACCGCAGATAAGTTTTTTTCTCTT
TGAAAGATAGAGATTAATACAACTACTTAAAAAATATAGTCAATAGGTTACTAAGATATTGCTTAGCGTTAAGTTTTTAACGTAATTTTAATAGCTTAAGA
TTTTAAGAGAAAATATGAAGACTTAGAAGAGTAGCATGAGGAAGGAAAAGATAAAAGGTTTCTAAAACATGACGGAGGTTGAGATGAAGCTTCTTCAT
GGAGTAAAAAATGTATTTAAAAGAAAATTGAGAGAAAGGACTACAGAGCCCCGAATTAATACCAATAGAAGGGCAATGCTTTTAGATTAAAATGAAGG
TGACTTAAACAGCTTAAAGTTTAGTTTAAAAGTTGTAGGTGATTAAAATAATTTGAAGGCGATCTTTTAAAAAGAGATTAAACCGAAGGTGATTAAAAG
ACCTTGAAATCCATGACGCAGGGAGAATTGCGTCATTTAAAGCCTAGTTAACGCATTTACTAAACGCAGACGAAAATGGAAAGATTAATTGGGAGTGG
TAGGATGAAACAATTTGGAGAAGATAGAAGTTTGAAGTGGAAAACTGGAAGACAGAAGTACGGGAAGGCGAAGAAAAGAATAGAGAAGATAGGGA
AATTAGAAGATAAAAACATACTTTTAGAAGAAAAAAGATAAATTTAAACCTGAAAAGTAGGAAGCAGAAGAAAAAAGACAAGCTAGGAAACAAAAA
GCTAAGGGCAAAATGTACAAACTTAGAAGAAAATTGGAAGATAGAAACAAGATAGAAAATGAAAATATTGTCAAGAGTTTCAGATAGAAAATGAAAA
ACAAGCTAAGACAAGTATTGGAGAAGTATAGAAGATAGAAAAATATAAAGCCAAAAATTGGATAAAATAGCACTGAAAAAATGAGGAAATTATTGGTA

ACCAATTTATTTTAAAAGCCCATCAATTTAATTTCTGGTGGTGCAGAAGTTAGAAGGTAAAGCTTGAGAAGATGAGGGTGTTTACGTAGACCAGAACCA
ATTTAGAAGAATACTTGAAGCTAGAAGGGGAAGTTGGTTAAAAATCACATCAAAAAGCTACTAAAAGGACTGGTGTAATTTAAAAAAAACTAAGGCA
GAAGGCTTTTGGAAGAGTTAGAAGAATTTGGAAGGCCTTAAATATAGTAGCTTAGTTTGAAAAATGTGAAGGACTTTCGTAACGGAAGTAATTCAAGA
TCAAGAGTAATTACCAACTTAATGTTTTTGCATTGGACTTTGAGTTAAGATTATTTTTTAAATCCTGAGGACTAGCATTAATTGACAGCTGACCCAGGTG
CTACACAGAAGTGGATTCAGTGAATCTAGGAAGACAGCAGCAGACAGGATTCCAGGAACCAGTGTTTGATGAAGCTAGGACTGAGGAGCAAGCGAG
CAAGCAGCAGTTCGTGGTGAAGATAGGAAAAGAGTCCAGGAGCCAGTGCGATTTGGTGAAGGAAGCTAGGAAGAAGGAAGGAGCGCTAACGATTTG
GTGGTGAAGCTAGGAAAAAGGATTCCAGGAAGGAGCGAGTGCAATTTGGTGATGAAGGTAGCAGGCGGCTTGGCTTGGCAACCACACGGAGGAGGC
GAGCAGGCGTTGTGCGTAGAGGATCCTAGACCAGCATGCCAGTGTGCCAAGGCCACAGGGAAAGCGAGTGGTTGGTAAAAATCCGTGAGGTCGGCA
ATATGTTGTTTTTCTGGAACTTACTTATGGTAACCTTTTATTTATTTTCTAATATAATGGGGGAGTTTCGTACTGAGGTGTAAAGGGATTTATATGGGGACG
TAGGCCGATTTCCGGGTGTTGTAGGTTTCTCTTTTTCAGGCTTATACTCATGAATCTTGTCTGAAGCTTTTGAGGGCAGACTGCCAAGTCCTGGAGAAAT
AGTAGATGGCAAGTTTGTGGGTTTTTTTTTTTTACACGAATTTGAGGAAAACCAAATGAATTTGATAGCCAAATTGAGACAATTTCAGCAAATCTGTAA
GCAGTTTGTATGTTTAGTTGGGGTAATGAAGTATTTCAGTTTTGTGAATAGATGACCTGTTTTTACTTCCTCACCCTGAATTCGTTTTGTAAATGTAGAGT
TTGGATGTGTAACTGAGGCGGGGGGGAGTTTTCAGTATTTTTTTTTGTGGGGGTGGGGGCAAAATATGTTTTCAGTTCTTTTTCCCTTAGGTCTGTCTAG
AATCCTAAAGGCAAATGACTCAAGGTGTAACAGAAAACAAGAAAATCCAATATCAGGATAATCAGACCACCACAGGTTTACAGTTTATAGAAACTAGA
GCAGTTCTCACGTTGAGGTCTGTGGAAGAGATGTCCATTGGAGAAATGGCTGGTAGTTACTCTTTTTTCCCCCCACCCCCTTAATCAGACTTTAAAAGT
GCTTAACCCCTTAAACTTGTTATTTTTTACTTGAAGCATTTTGGGATGGTCTTAACAGGGAAGAGAGAGGGTGGGGGAGAAAATGTTTTTTTCTAAGAT
TTTCCACAGATGCTATAGTACTATTGACAAACTGGGTTAGAGAAGGAGTGTACCGCTGTGCTGTTGGCACGAACACCTTCAGGGACTGGAGCTGCTTTT
ATCCTTGGAAGAGTATTCCCAGTTGAAGCTGAAAAGTACAGCACAGTGCAGCTTTGGTTCATATTCAGTCATCTCAGGAGAACTTCAGAAGAGCTTGA
GTAGGCCAAATGTTGAAGTTAAGTTTTCCAATAATGTGACTTCTTAAAAGTTTTATTAAAGGGGAGGGGCAAATATTGGCAATTAGTTGGCAGTGGCCT
GTTACGGTTGGGATTGGTGGGGTGGGTTTAGGTAATTGTTTAGTTTATGATTGCAGATAAACTCATGCCAGAGAACTTAAAGTCTTAGAATGGAAAAAG
TAAAGAAATATCAACTTCCAAGTTGGCAAGTAACTCCCAATGATTTAGTTTTTTTCCCCCCAGTTTGAATTGGGAAGCTGGGGGAAGTTAAATATGAGC
CACTGGGTGTACCAGTGCATTAATTTGGGCAAGGAAAGTGTCATAATTTGATACTGTATCTGTTTTCCTTCAAAGTATAGAGCTTTTGGGGAAGGAAAGT
ATTGAACTGGGGGTTGGTCTGGCCTACTGGGCTGACATTAACTACAATTATGGGAAATGCAAAAGTTGTTTGGATATGGTAGTGTGTGGTTCTCTTTTGG
AATTTTTTTCAGGTGATTTAATAATAATTTAAAACTACTATAGAAACTGCAGAGCAAAGGAAGTGGCTTAATGATCCTGAAGGGATTTCTTCTGATGGTA
GCTTTTGTATTATCAAGTAAGATTCTATTTTCAGTTGTGTGTAAGCAAGTTTTTTTTTAGTGTAGGAGAAATACTTTTCCATTGTTTAACTGCAAAACAAG
ATGTTAAGGTATGCTTCAAAAATTTTGTAAATTGTTTATTTTAAACTTATCTGTTTGTAAATTGTAACTGATTAAGAATTGTGATAGTTCAGCTTGAATGTC

TCTTAGAGGGTGGGCTTTTGTTGATGAGGGAGGGGAAACTTTTTTTTTTTCTATAGACTTTTTTCAGATAACATCTTCTGAGTCATAACCAGCCTGGCAG
TATGATGGCCTAGATGCAGAGAAAACAGCTCCTTGGTGAATTGATAAGTAAAGGCAGAAAAGATTATATGTCATACCTCCATTGGGGAATAAGCATAAC
CCTGAGATTCTTACTACTGATGAGAACATTATCTGCATATGCCAAAAAATTTTAAGCAAATGAAAGCTACCAATTTAAAGTTACGGAATCTACCATTTTA
AAGTTAATTGCTTGTCAAGCTATAACCACAAAAATAATGAATTGATGAGAAATACAATGAAGAGGCAATGTCCATCTCAAAATACTGCTTTTACAAAAG
CAGAATAAAAGCGAAAAGAAATGAAAATGTTACACTACATTAATCCTGGAATAAAAGAAGCCGAAATAAATGAGAGATGAGTTGGGATCAAGTGGATT
GAGGAGGCTGTGCTGTGTGCCAATGTTTCGTTTGCCTCAGACAGGTATCTCTTCGTTATCAGAAGAGTTGCTTCATTTCATCTGGGAGCAGAAAACAGC
AGGCAGCTGTTAACAGATAAGTTTAACTTGCATCTGCAGTATTGCATGTTAGGGATAAGTGCTTATTTTTAAGAGCTGTGGAGTTCTTAAATATCAACCA
TGGCACTTTCTCCTGACCCCTTCCCTAGGGGATTTCAGGATTGAGAAATTTTTCCATCGAGCCTTTTTAAAATTGTAGGACTTGTTCCTGTGGGCTTCAG
TGATGGGATAGTACACTTCACTCAGAGGCATTTGCATCTTTAAATAATTTCTTAAAAGCCTCTAAAGTGATCAGTGCCTTGATGCCAACTAAGGAAATTT
GTTTAGCATTGAATCTCTGAAGGCTCTATGAAAGGAATAGCATGATGTGCTGTTAGAATCAGATGTTACTGCTAAAATTTACATGTTGTGATGTAAATTGT
GTAGAAAACCATTAAATCATTCAAAATAATAAACTATTTTTATTAGAGAATGTATACTTTTAGAAAGCTGTCTCCTTATTTAAATAAAATAGTGTTTGTCTG
TAGTTCAGTGTTGGGGCAATCTTGGGGGGGATTCTTCTCTAATCTTTCAGAAACTTTGTCTGCGAACACTCTTTAATGGACCAGATCAGGATTTGAGCG
GAAGAACGAATGTAACTTTAAGGCAGGAAAGACAAATTTTATTCTTCATAAAGTGATGAGCATATAATAATTCCAGGCACATGGCAATAGAGGCCCTCT
AAATAAGGAATAAATAACCTCTTAGACAGGTGGGAGGTAACAGCACAATATCTTTGAACTATATACATCCTTGATGTATAATTTGTCAGGAGCTTGACTT
GATTGTATATTCATATTTACACGAGAACCTAATATAACTGCCTTGTCTTTTTCAGGTAATAGCCTGCAGCTGGTGTTTTGAGAAGCCCTACTGCTGAAAAC
>ENST00000534336.1|ENSG00000251562.8|OTTHUMG00000166322.5|OTTHUMT00000389143.1|AP000769.3-001|MALAT1|8708|
GTAAAGGACTGGGGCCCCGCAACTGGCCTCTCCTGCCCTCTTAAGCGCAGCGCCATTTTAGCAACGCAGAAGCCCGGCGCCGGGAAGCCTCAGCTCG
CCTGAAGGCAGGTCCCCTCTGACGCCTCCGGGAGCCCAGGTTTCCCAGAGTCCTTGGGACGCAGCGACGAGTTGTGCTGCTATCTTAGCTGTCCTTAT
AGGCTGGCCATTCCAGGTGGTGGTATTTAGATAAAACCACTCAAACTCTGCAGTTTGGTCTTGGGGTTTGGAGGAAAGCTTTTATTTTTCTTCCTGCTCC
GGTTCAGAAGGTCTGAAGCTCATACCTAACCAGGCATAACACAGAATCTGCAAAACAAAAACCCCTAAAAAAGCAGACCCAGAGCAGTGTAAACACT
TCTGGGTGTGTCCCTGACTGGCTGCCCAAGGTCTCTGTGTCTTCGGAGACAAAGCCATTCGCTTAGTTGGTCTACTTTAAAAGGCCACTTGAACTCGCT
TTCCATGGCGATTTGCCTTGTGAGCACTTTCAGGAGAGCCTGGAAGCTGAAAAACGGTAGAAAAATTTCCGTGCGGGCCGTGGGGGGCTGGCGGCAA
CTGGGGGGCCGCAGATCAGAGTGGGCCACTGGCAGCCAACGGCCCCCGGGGCTCAGGCGGGGAGCAGCTCTGTGGTGTGGGATTGAGGCGTTTTCC
AAGAGTGGGTTTTCACGTTTCTAAGATTTCCCAAGCAGACAGCCCGTGCTGCTCCGATTTCTCGAACAAAAAAGCAAAACGTGTGGCTGTCTTGGGA
GCAAGTCGCAGGACTGCAAGCAGTTGGGGGAGAAAGTCCGCCATTTTGCCACTTCTCAACCGTCCCTGCAAGGCTGGGGCTCAGTTGCGTAATGGAA
AGTAAAGCCCTGAACTATCACACTTTAATCTTCCTTCAAAAGGTGGTAAACTATACCTACTGTCCCTCAAGAGAACACAAGAAGTGCTTTAAGAGGTAT

TTTAAAAGTTCCGGGGGTTTTGTGAGGTGTTTGATGACCCGTTTAAAATATGATTTCCATGTTTCTTTTGTCTAAAGTTTGCAGCTCAAATCTTTCCACAC
GCTAGTAATTTAAGTATTTCTGCATGTGTAGTTTGCATTCAAGTTCCATAAGCTGTTAAGAAAAATCTAGAAAAGTAAAACTAGAACCTATTTTTAACCG
AAGAACTACTTTTTGCCTCCCTCACAAAGGCGGCGGAAGGTGATCGAATTCCGGTGATGCGAGTTGTTCTCCGTCTATAAATACGCCTCGCCCGAGCTG
TGCGGTAGGCATTGAGGCAGCCAGCGCAGGGGCTTCTGCTGAGGGGGCAGGCGGAGCTTGAGGAAACCGCAGATAAGTTTTTTTCTCTTTGAAAGAT
AGAGATTAATACAACTACTTAAAAAATATAGTCAATAGGTTACTAAGATATTGCTTAGCGTTAAGTTTTTAACGTAATTTTAATAGCTTAAGATTTTAAGA
GAAAATATGAAGACTTAGAAGAGTAGCATGAGGAAGGAAAAGATAAAAGGTTTCTAAAACATGACGGAGGTTGAGATGAAGCTTCTTCATGGAGTAA
AAAATGTATTTAAAAGAAAATTGAGAGAAAGGACTACAGAGCCCCGAATTAATACCAATAGAAGGGCAATGCTTTTAGATTAAAATGAAGGTGACTTA
AACAGCTTAAAGTTTAGTTTAAAAGTTGTAGGTGATTAAAATAATTTGAAGGCGATCTTTTAAAAAGAGATTAAACCGAAGGTGATTAAAAGACCTTGA
AATCCATGACGCAGGGAGAATTGCGTCATTTAAAGCCTAGTTAACGCATTTACTAAACGCAGACGAAAATGGAAAGATTAATTGGGAGTGGTAGGATG
AAACAATTTGGAGAAGATAGAAGTTTGAAGTGGAAAACTGGAAGACAGAAGTACGGGAAGGCGAAGAAAAGAATAGAGAAGATAGGGAAATTAGA
AGATAAAAACATACTTTTAGAAGAAAAAAGATAAATTTAAACCTGAAAAGTAGGAAGCAGAAGAAAAAAGACAAGCTAGGAAACAAAAAGCTAAGG
GCAAAATGTACAAACTTAGAAGAAAATTGGAAGATAGAAACAAGATAGAAAATGAAAATATTGTCAAGAGTTTCAGATAGAAAATGAAAAACAAGCT
AAGACAAGTATTGGAGAAGTATAGAAGATAGAAAAATATAAAGCCAAAAATTGGATAAAATAGCACTGAAAAAATGAGGAAATTATTGGTAACCAATTT
ATTTTAAAAGCCCATCAATTTAATTTCTGGTGGTGCAGAAGTTAGAAGGTAAAGCTTGAGAAGATGAGGGTGTTTACGTAGACCAGAACCAATTTAGA
AGAATACTTGAAGCTAGAAGGGGAAGTTGGTTAAAAATCACATCAAAAAGCTACTAAAAGGACTGGTGTAATTTAAAAAAAACTAAGGCAGAAGGCT
TTTGGAAGAGTTAGAAGAATTTGGAAGGCCTTAAATATAGTAGCTTAGTTTGAAAAATGTGAAGGACTTTCGTAACGGAAGTAATTCAAGATCAAGAGT
AATTACCAACTTAATGTTTTTGCATTGGACTTTGAGTTAAGATTATTTTTTAAATCCTGAGGACTAGCATTAATTGACAGCTGACCCAGGTGCTACACAG
AAGTGGATTCAGTGAATCTAGGAAGACAGCAGCAGACAGGATTCCAGGAACCAGTGTTTGATGAAGCTAGGACTGAGGAGCAAGCGAGCAAGCAGC
AGTTCGTGGTGAAGATAGGAAAAGAGTCCAGGAGCCAGTGCGATTTGGTGAAGGAAGCTAGGAAGAAGGAAGGAGCGCTAACGATTTGGTGGTGAA
GCTAGGAAAAAGGATTCCAGGAAGGAGCGAGTGCAATTTGGTGATGAAGGTAGCAGGCGGCTTGGCTTGGCAACCACACGGAGGAGGCGAGCAGGC
GTTGTGCGTAGAGGATCCTAGACCAGCATGCCAGTGTGCCAAGGCCACAGGGAAAGCGAGTGGTTGGTAAAAATCCGTGAGGTCGGCAATATGTTGTT
TTTCTGGAACTTACTTATGGTAACCTTTTATTTATTTTCTAATATAATGGGGGAGTTTCGTACTGAGGTGTAAAGGGATTTATATGGGGACGTAGGCCGATT
TCCGGGTGTTGTAGGTTTCTCTTTTTCAGGCTTATACTCATGAATCTTGTCTGAAGCTTTTGAGGGCAGACTGCCAAGTCCTGGAGAAATAGTAGATGG
CAAGTTTGTGGGTTTTTTTTTTTTACACGAATTTGAGGAAAACCAAATGAATTTGATAGCCAAATTGAGACAATTTCAGCAAATCTGTAAGCAGTTTGTA
TGTTTAGTTGGGGTAATGAAGTATTTCAGTTTTGTGAATAGATGACCTGTTTTTACTTCCTCACCCTGAATTCGTTTTGTAAATGTAGAGTTTGGATGTGT
AACTGAGGCGGGGGGGAGTTTTCAGTATTTTTTTTTGTGGGGGTGGGGGCAAAATATGTTTTCAGTTCTTTTTCCCTTAGGTCTGTCTAGAATCCTAAAG

GCAAATGACTCAAGGTGTAACAGAAAACAAGAAAATCCAATATCAGGATAATCAGACCACCACAGGTTTACAGTTTATAGAAACTAGAGCAGTTCTCA
CGTTGAGGTCTGTGGAAGAGATGTCCATTGGAGAAATGGCTGGTAGTTACTCTTTTTTCCCCCCACCCCCTTAATCAGACTTTAAAAGTGCTTAACCCC
TTAAACTTGTTATTTTTTACTTGAAGCATTTTGGGATGGTCTTAACAGGGAAGAGAGAGGGTGGGGGAGAAAATGTTTTTTTCTAAGATTTTCCACAGA
TGCTATAGTACTATTGACAAACTGGGTTAGAGAAGGAGTGTACCGCTGTGCTGTTGGCACGAACACCTTCAGGGACTGGAGCTGCTTTTATCCTTGGAA
GAGTATTCCCAGTTGAAGCTGAAAAGTACAGCACAGTGCAGCTTTGGTTCATATTCAGTCATCTCAGGAGAACTTCAGAAGAGCTTGAGTAGGCCAAA
TGTTGAAGTTAAGTTTTCCAATAATGTGACTTCTTAAAAGTTTTATTAAAGGGGAGGGGCAAATATTGGCAATTAGTTGGCAGTGGCCTGTTACGGTTGG
GATTGGTGGGGTGGGTTTAGGTAATTGTTTAGTTTATGATTGCAGATAAACTCATGCCAGAGAACTTAAAGTCTTAGAATGGAAAAAGTAAAGAAATAT
CAACTTCCAAGTTGGCAAGTAACTCCCAATGATTTAGTTTTTTTCCCCCCAGTTTGAATTGGGAAGCTGGGGGAAGTTAAATATGAGCCACTGGGTGTA
CCAGTGCATTAATTTGGGCAAGGAAAGTGTCATAATTTGATACTGTATCTGTTTTCCTTCAAAGTATAGAGCTTTTGGGGAAGGAAAGTATTGAACTGGG
GGTTGGTCTGGCCTACTGGGCTGACATTAACTACAATTATGGGAAATGCAAAAGTTGTTTGGATATGGTAGTGTGTGGTTCTCTTTTGGAATTTTTTTCA
GGTGATTTAATAATAATTTAAAACTACTATAGAAACTGCAGAGCAAAGGAAGTGGCTTAATGATCCTGAAGGGATTTCTTCTGATGGTAGCTTTTGTATTA
TCAAGTAAGATTCTATTTTCAGTTGTGTGTAAGCAAGTTTTTTTTTAGTGTAGGAGAAATACTTTTCCATTGTTTAACTGCAAAACAAGATGTTAAGGTAT
GCTTCAAAAATTTTGTAAATTGTTTATTTTAAACTTATCTGTTTGTAAATTGTAACTGATTAAGAATTGTGATAGTTCAGCTTGAATGTCTCTTAGAGGGT
GGGCTTTTGTTGATGAGGGAGGGGAAACTTTTTTTTTTTCTATAGACTTTTTTCAGATAACATCTTCTGAGTCATAACCAGCCTGGCAGTATGATGGCCT
AGATGCAGAGAAAACAGCTCCTTGGTGAATTGATAAGTAAAGGCAGAAAAGATTATATGTCATACCTCCATTGGGGAATAAGCATAACCCTGAGATTCT
TACTACTGATGAGAACATTATCTGCATATGCCAAAAAATTTTAAGCAAATGAAAGCTACCAATTTAAAGTTACGGAATCTACCATTTTAAAGTTAATTGCT
TGTCAAGCTATAACCACAAAAATAATGAATTGATGAGAAATACAATGAAGAGGCAATGTCCATCTCAAAATACTGCTTTTACAAAAGCAGAATAAAAGC
GAAAAGAAATGAAAATGTTACACTACATTAATCCTGGAATAAAAGAAGCCGAAATAAATGAGAGATGAGTTGGGATCAAGTGGATTGAGGAGGCTGTG
CTGTGTGCCAATGTTTCGTTTGCCTCAGACAGGTATCTCTTCGTTATCAGAAGAGTTGCTTCATTTCATCTGGGAGCAGAAAACAGCAGGCAGCTGTTA
ACAGATAAGTTTAACTTGCATCTGCAGTATTGCATGTTAGGGATAAGTGCTTATTTTTAAGAGCTGTGGAGTTCTTAAATATCAACCATGGCACTTTCTCC
TGACCCCTTCCCTAGGGGATTTCAGGATTGAGAAATTTTTCCATCGAGCCTTTTTAAAATTGTAGGACTTGTTCCTGTGGGCTTCAGTGATGGGATAGTA
CACTTCACTCAGAGGCATTTGCATCTTTAAATAATTTCTTAAAAGCCTCTAAAGTGATCAGTGCCTTGATGCCAACTAAGGAAATTTGTTTAGCATTGAA
TCTCTGAAGGCTCTATGAAAGGAATAGCATGATGTGCTGTTAGAATCAGATGTTACTGCTAAAATTTACATGTTGTGATGTAAATTGTGTAGAAAACCAT
TAAATCATTCAAAATAATAAACTATTTTTATTAGAGAATGTATACTTTTAGAAAGCTGTCTCCTTATTTAAATAAAATAGTGTTTGTCTGTAGTTCAGTGTT
GGGGCAATCTTGGGGGGGATTCTTCTCTAATCTTTCAGAAACTTTGTCTGCGAACACTCTTTAATGGACCAGATCAGGATTTGAGCGGAAGAACGAATG
TAACTTTAAGGCAGGAAAGACAAATTTTATTCTTCATAAAGTGATGAGCATATAATAATTCCAGGCACATGGCAATAGAGGCCCTCTAAATAAGGAATAA

ATAACCTCTTAGACAGGTGGGAGATTATGATCAGAGTAAAAGGTAATTACACATTTTATTTCCAGAAAGTCAGGGGTCTATAAATTGACAGTGATTAGAG
TAATACTTTTTCACATTTCCAAAGTTTGCATGTTAACTTTAAATGCTTACAATCTTAGAGTGGTAGGCAATGTTTTACACTATTGACCTTATATAGGGAAG
GGAGGGGGTGCCTGTGGGGTTTTAAAGAATTTTCCTTTGCAGAGGCATTTCATCCTTCATGAAGCCATTCAGGATTTTGAATTGCATATGAGTGCTTGGC
TCTTCCTTCTGTTCTAGTGAGTGTATGAGACCTTGCAGTGAGTTTATCAGCATACTCAAAATTTTTTTCCTGGAATTTGGAGGGATGGGAGGAGGGGGT
GGGGCTTACTTGTTGTAGCTTTTTTTTTTTTTACAGACTTCACAGAGAATGCAGTTGTCTTGACTTCAGGTCTGTCTGTTCTGTTGGCAAGTAAATGCAG
TACTGTTCTGATCCCGCTGCTATTAGAATGCATTGTGAAACGACTGGAGTATGATTAAAAGTTGTGTTCCCCAATGCTTGGAGTAGTGATTGTTGAAGGA
AAAAATCCAGCTGAGTGATAAAGGCTGAGTGTTGAGGAAATTTCTGCAGTTTTAAGCAGTCGTATTTGTGATTGAAGCTGAGTACATTTTGCTGGTGTA
TTTTTAGGTAAAATGCTTTTTGTTCATTTCTGGTGGTGGGAGGGGACTGAAGCCTTTAGTCTTTTCCAGATGCAACCTTAAAATCAGTGACAAGAAACA
TTCCAAACAAGCAACAGTCTTCAAGAAATTAAACTGGCAAGTGGAAATGTTTAAACAGTTCAGTGATCTTTAGTGCATTGTTTATGTGTGGGTTTCTCT
CTCCCCTCCCTTGGTCTTAATTCTTACATGCAGGAACACTCAGCAGACACACGTATGCGAAGGGCCAGAGAAGCCAGACCCAGTAAGAAAAAATAGCC
TATTTACTTTAAATAAACCAAACATTCCATTTTAAATGTGGGGATTGGGAACCACTAGTTCTTTCAGATGGTATTCTTCAGACTATAGAAGGAGCTTCCA
GTTGAATTCACCAGTGGACAAAATGAGGAAAACAGGTGAACAAGCTTTTTCTGTATTTACATACAAAGTCAGATCAGTTATGGGACAATAGTATTGAAT
AGATTTCAGCTTTATGCTGGAGTAACTGGCATGTGAGCAAACTGTGTTGGCGTGGGGGTGGAGGGGTGAGGTGGGCGCTAAGCCTTTTTTTAAGATTT
TTCAGGTACCCCTCACTAAAGGCACCGAAGGCTTAAAGTAGGACAACCATGGAGCCTTCCTGTGGCAGGAGAGACAACAAAGCGCTATTATCCTAAG
GTCAAGAGAAGTGTCAGCCTCACCTGATTTTTATTAGTAATGAGGACTTGCCTCAACTCCCTCTTTCTGGAGTGAAGCATCCGAAGGAATGCTTGAAGT
ACCCCTGGGCTTCTCTTAACATTTAAGCAAGCTGTTTTTATAGCAGCTCTTAATAATAAAGCCCAAATCTCAAGCGGTGCTTGAAGGGGAGGGAAAGGG
GGAAAGCGGGCAACCACTTTTCCCTAGCTTTTCCAGAAGCCTGTTAAAAGCAAGGTCTCCCCACAAGCAACTTCTCTGCCACATCGCCACCCCGTGCC
TTTTGATCTAGCACAGACCCTTCACCCCTCACCTCGATGCAGCCAGTAGCTTGGATCCTTGTGGGCATGATCCATAATCGGTTTCAAGGTAACGATGGTG
TCGAGGTCTTTGGTGGGTTGAACTATGTTAGAAAAGGCCATTAATTTGCCTGCAAATTGTTAACAGAAGGGTATTAAAACCACAGCTAAGTAGCTCTAT
TATAATACTTATCCAGTGACTAAAACCAACTTAAACCAGTAAGTGGAGAAATAACATGTTCAAGAACTGTAATGCTGGGTGGGAACATGTAACTTGTAG
ACTGGAGAAGATAGGCATTTGAGTGGCTGAGAGGGCTTTTGGGTGGGAATGCAAAAATTCTCTGCTAAGACTTTTTCAGGTGAACATAACAGACTTGG
CCAAGCTAGCATCTTAGCGGAAGCTGATCTCCAATGCTCTTCAGTAGGGTCATGAAGGTTTTTCTTTTCCTGAGAAAACAACACGTATTGTTTTCTCAG
GTTTTGCTTTTTGGCCTTTTTCTAGCTTAAAAAAAAAAAAAAGCAAAAGATGCTGGTGGTTGGCACTCCTGGTTTCCAGGACGGGGTTCAAATCCCTGC
GGCGTCTTTGCTTTGACTACTAATCTGTCTTCAGGACTCTTTCTGTATTTCTCCTTTTCTCTGCAGGTGCTAGTTCTTGGAGTTTTGGGGAGGTGGGAGG
TAACAGCACAATATCTTTGAACTATATACATCCTTGATGTATAATTTGTCAGGAGCTTGACTTGATTGTATATTCATATTTACACGAGAACCTAATATAACT
GCCTTGTCTTTTTCAGGTAATAGCCTGCAGCTGGTGTTTTGAGAAGCCCTACTGCTGAAAACTTAACAATTTTGTGTAATAAAAATGGAGAAGCTCTAA

A

>ENST00000620902.1|ENSG00000251562.8|OTTHUMG00000166322.5|OTTHUMT00000473535.1|AP000769.3-005|MALAT1|352|
GGTAGGCATTGAGGCAGCCAGCGCAGGGGCTTCTGCTGAGGGGGCAGGCGGAGCTTGAGGAAACCGCAGATAAGTTTTTTTCTCTTTGAAAGATAGA
GATTAATACAACTACTTAAAAAATATAGTCAATAGGTTACTAAGATATTGCTTAGCGTTAAGTTTTTAACGTAATTTTAATAGCTTAAGATTTTAAGAGAA
AATATGAAGACTTAGAAGAGTAGCATGAGGAAGGAAAAGATAAAAGGTTTCTAAAACATGACGGAGTGGTAGGATGAAACAATTTGGAGAAGATAGA
AGTTTGAAGTGGAAAACTGGAAGACAGAAGTACGGGAAGGCGAAGAAAAGAATAGAG
>ENST00000617791.1|ENSG00000251562.8|OTTHUMG00000166322.5|OTTHUMT00000473536.1|AP000769.3-006|MALAT1|394|
GGTAGGCATTGAGGCAGCCAGCGCAGGGGCTTCTGCTGAGGGGGCAGGCGGAGCTTGAGGAAACCGCAGATAAGTTTTTTTCTCTTTGAAAGATAGA
GATTAATACAACTACTTAAAAAATATAGTCAATAGGTTACTAAGATATTGCTTAGCGTTAAGTTTTTAACGTAATTTTAATAGCTTAAGATTTTAAGAGAA
AATATGAAGACTTAGAAGAGTAGCATGAGGAAGGAAAAGATAAAAGGTTTCTAAAACATGACGGAGGTTGAGATGAAGCTTCTTCATGGAGTAAAAA
ATGCGAAGAAAAGAATAGAGAAGATAGGGAAATTAGAAGATAAAAACATACTTTTAGAAGAAAAAAGATAAATTTAAACCTGAAAAGTAGGAAGCAG
AA
>ENST00000544868.2|ENSG00000251562.8|OTTHUMG00000166322.5|OTTHUMT00000473537.1|AP000769.3-007|MALAT1|480|
GGTAGGCATTGAGGCAGCCAGCGCAGGGGCTTCTGCTGAGGGGGCAGGCGGAGCTTGAGGAAACCGCAGATAAGTTTTTTTCTCTTTGAAAGATAGA
GATTAATACAACTACTTAAAAAATATAGTCAATAGGTTACTAAGATATTGCTTAGCGTTAAGTTTTTAACGTAATTTTAATAGCTTAAGATTTTAAGAGAA
AATATGAAGACTTAGAAGAGTAGCATGAGGAAGGAAAAGATAAAAGGTTTCTAAAACATGACGGAGGTTGAGATGAAGCTTCTTCATGGAGTAAAAA
ATGTATTTAAAAGAAAATTGAGAGAAAGGACTACAGAGCCCCGAATTAATACCAATAGAAGGGCAATGCTTTTAGATTAAAATGAAGGTGACTTAAAC
AGCTTAAAGTTTAGTTTAAAAGTTGTAGGTTTCTCTTTTTCAGGCTTATACTCATGAATCTTGTCTGAAGCTTTTGAGGGCAGACTG
>ENST00000610481.1|ENSG00000251562.8|OTTHUMG00000166322.5|OTTHUMT00000473538.1|AP000769.3-008|MALAT1|353|
AGAAGAATTTGGAAGGCCTTAAATATAGTAGCTTAGTTTGAAAAATGTGAAGGACTTTCGTAACGGAAGTAATTCAAGATCAAGAGTAATTACCAACTT
AATGTTTTTGCATTGGACTTTGAGTTAAGATTATTTTTTAAATCCTGAGGACTAGCATTAATTGACAGCTGACCCAGGAGCCAGTGCGATTTGGTGAAGG
AAGCTAGGAAGAAGGAAGGAGCGCTAACGATTTGGTGGTGAAGCTAGGAAAAAGGATTCCAGGAAGGAGCGAGTGCAATTTGGTGATGAAGGTAGC
AGGCGGCTTGGCTTGGCAACCACACGGAGGAGGCGAGCAGGCGTTGTGCGTAGAGGAT
>ENST00000508832.2|ENSG00000251562.8|OTTHUMG00000166322.5|OTTHUMT00000389144.2|AP000769.3-002|MALAT1|1519|
GGCAAATATTGGCAATTAGTTGGCAGTGGCCTGTTACGGTTGGGATTGGTGGGGTGGGTTTAGGTAATTGTTTAGTTTATGATTGCAGATAAACTCATGC
CAGAGAACTTAAAGTCTTAGAATGGAAAAAGTAAAGAAATATCAACTTCCAAGTTGGCAAGTAACTCCCAATGATTTAGTTTTTTTCCCCCCAGTTTGA

ATTGGGAAGCTGGGGGAAGTTAAATATGAGCCACTGGGTGTACCAGTGCATTAATTTGGGCAAGGAAAGTGTCATAATTTGATACTGTATCTGTTTTCCT
TCAAAGTATAGAGCTTTTGGGGAAGGAAAGTATTGAACTGGGGGTTGGTCTGGCCTACTGGGCTGACATTAACTACAATTATGGGAAATGCAAAAGTT
GTTTGGATATGGTAGTGTGTGGTTCTCTTTTGGAATTTTTTTCAGGTGATTTAATAATAATTTAAAACTACTATAGAAACTGCAGAGCAAAGGAAGTGGC
TTAATGATCCTGAAGGGATTTCTTCTGATGGTAGCTTTTGTATTATCAAACTTTTTTCAGATAACATCTTCTGAGTCATAACCAGCCTGGCAGTATGATGG
CCTAGATGCAGAGAAAACAGCTCCTTGGTGAATTGATAAGTAAAGGCAGAAAAGATTATATGTCATACCTCCATTGGGGAATAAGCATAACCCTGAGAT
TCTTACTACTGATGAGAACATTATCTGCATATGCCAAAAAATTTTAAGCAAATGAAAGCTACCAATTTAAAGTTACGGAATCTACCATTTTAAAGTTAATT
GCTTGTCAAGCTATAACCACAAAAATAATGAATTGATGAGAAATACAATGAAGAGGCAATGTCCATCTCAAAATACTGCTTTTACAAAAGCAGAATAAA
AGCGAAAAGAAATGAAAATGTTACACTACATTAATCCTGGAATAAAAGAAGCCGAAATAAATGAGAGATGAGTTGGGATCAAGTGGATTGAGGAGGC
TGTGCTGTGTGCCAATGTTTCGTTTGCCTCAGACAGGTATCTCTTCGTTATCAGAAGAGTTGCTTCATTTCATCTGGGAGCAGAAAACAGCAGGCAGCT
GTTAACAGATAAGTTTAACTTGCATCTGCAGTATTGCATGTTAGGGATAAGTGCTTATTTTTAAGAGCTGTGGAGTTCTTAAATATCAACCATGGCACTTT
CTCCTGACCCCTTCCCTAGGGGATTTCAGGATTGAGAAATTTTTCCATCGAGCCTTTTTAAAATTGTAGGACTTGTTCCTGTGGGCTTCAGTGATGGGAT
AGTACACTTCACTCAGAGGCATTTGCATCTTTAAATAATTTCTTAAAAGCCTCTAAAGTGATCAGTGCCTTGATGCCAACTAAGGAAATTTGTTTAGCAT
TGAATCTCTGAAGGCTCTATGAAAGGAATAGCATGATGTGCTGTTAGAATCAGATGTTACTGCTAAAATTTACATGTTGTGATGTAAATTGTGTAGAAAA
CCATTAAATCATTCAAAATAATAAA
>ENST00000616527.4|ENSG00000251562.8|OTTHUMG00000166322.5|OTTHUMT00000473539.1|AP000769.3-012|MALAT1|572|
AGTCTTAGAATGGAAAAAGTAAAGAAATATCAACTTCCAAGTTGGCAAGTAACTCCCAATGATTTAGTTTTTTTCCCCCCAGTTTGAATTGGGAAGCTG
GGGGAAGTTAAATATGAGCCACTGGGTGTACCAGTGCATTAATTTGGGCAAGGAAAGTGTCATAATTTGATACTGTATCTGTTTTCCTTCAAAGTATAGA
GCTTTTGGGGAAGGAAAGTATTGAACTGGGGGTTGGTCTGGCCTACTGGGCTGACATTAACTACAATTATGGGAAATGCAAAAGTTGTTTGGATATGGT
AGTGTGTGGTTCTCTTTTGGAATTTTTTTCAGGTGATTTAATAATAATTTAAAACTACTATAGAAACTGCAGAGCAAAGGAAGTGGCTTAATGATCCTGA
AGGGATTTCTTCTGATGGTAGCTTTTGTATTATCAAACTTCACAGAGAATGCAGTTGTCTTGACTTCAGGTCTGTCTGTTCTGTTGGCAAGTAAATGCAG
TACTGTTCTGATCCCGCTGCTATTAGAATGCATTGTGAAACGACTGGAGTATGATTAAAAGTTGTGTTCCCCAA
>ENST00000618925.1|ENSG00000251562.8|OTTHUMG00000166322.5|OTTHUMT00000473540.1|AP000769.3-009|MALAT1|424|
TAAAGGCAGAAAAGATTATATGTCATACCTCCATTGGGGAATAAGCATAACCCTGAGATTCTTACTACTGATGAGAACATTATCTGCATATGCCAAAAAA
TTTTAAGCAAATGAAAGCTACCAATTTAAAGTTACGGAATCTACCATTTTAAAGTTAATTGCTTGTCAAGCTATAACCACAAAAATAATGAATTGATGAG
AAATACAATGAAGAGGCAATGTCCATCTCAAAATACTGCTTTTACAAAAGCAGAATAAAAGCGAAAAGAAATGAAAATGTTACACTACATTAATCCTG
GAATAAAAGAAGCCGAAATAAATGAGAGATGAGTTGGGATCAAGTGGATTGAGGAGGCTGTGCTGTGTGCCAATGTTTCGTTTGCCTCAGACAGAAA

CTTTGTCTGCGAACACTCTTTAATGGACC
>ENST00000620465.4|ENSG00000251562.8|OTTHUMG00000166322.5|OTTHUMT00000473541.1|AP000769.3-011|MALAT1|333|
TAGTTCAGTGTTGGGGCAATCTTGGGGGGGATTCTTCTCTAATCTTTCAGAAACTTTGTCTGCGAACACTCTTTAATGGACCAGATCAGGATTTGAGCG
GAAGAACGAATGTAACTTTAAGAGGCATTTCATCCTTCATGAAGCCATTCAGGATTTTGAATTGCATATGAGTGCTTGGCTCTTCCTTCTGTTCTAACTT
CACAGAGAATGCAGTTGTCTTGACTTCAGGTCTGTCTGTTCTGTTGGCAAGTAAATGCAGTACTGTTCTGATCCCGCTGCTATTAGAATGCATTGTGAA
ACGACTGGAGTATGATTAAAAGTTGTGTTCCCCAA
>ENST00000612781.1|ENSG00000251562.8|OTTHUMG00000166322.5|OTTHUMT00000473542.1|AP000769.3-010|MALAT1|234|
GGGGATTCTTCTCTAATCTTTCAGAAACTTTGTCTGCGAACACTCTTTAATGGACCAGATCAGGATTTGAGCGGAAGAACGAATGTAACTTTAAGAGGC
ATTTCATCCTTCATGAAGCCATTCAGGATTTTGAATTGCATATGAGTGCTTGGCTCTTCCTTCTGTTCTAACTTCACAGAGAATGCAGTTGTCTTGACTTC
AGATGCAACCTTAAAATCAGTGACAAGAAACATT
>ENST00000618132.1|ENSG00000251562.8|OTTHUMG00000166322.5|OTTHUMT00000473543.1|AP000769.3-013|MALAT1|725|
TTTCCAAAGTTTGCATGTTAACTTTAAATGCTTACAATCTTAGAGTGGTAGGCAATGTTTTACACTATTGACCTTATATAGGGAAGGGAGGGGGTGCCTG
TGGGGTTTTAAAGAATTTTCCTTTGCAGAGGCATTTCATCCTTCATGAAGCCATTCAGGATTTTGAATTGCATATGAGTGCTTGGCTCTTCCTTCTGTTCT
AACTTCACAGAGAATGCAGTTGTCTTGACTTCAGGTCTGTCTGTTCTGTTGGCAAGTAAATGCAGTACTGTTCTGATCCCGCTGCTATTAGAATGCATTG
TGAAACGACTGGAGTATGATTAAAAGTTGTGTTCCCCAATGCTTGGAGTAGTGATTGTTGAAGGAAAAAATCCAGCTGAGTGATAAAGGCTGAGTGTT
GAGGAAATTTCTGCAGTTTTAAGCAGTCGTATTTGTGATTGAAGCTGAGTACATTTTGCTGGTGTATTTTTAGGTAAAATGCTTTTTGTTCATTTCTGGTG
GTGGGAGGGGACTGAAGCCTTTAGTCTTTTCCAGATGCAACCTTAAAATCAGTGACAAGAAACATTCCAAACAAGCAACAGTCTTCAAGAAATTAAA
CTGGCAAGTGGAAATGTTTAAACAGTTCAGTGATCTTTAGTGCATTGTTTATGTGTGGGTTTCTCTCTCCCCTCCCTTGGTCTTAATTCTTACATGCAGGA
ACACTCAGCAGACACACGTATGCGAAG
>ENST00000613376.1|ENSG00000251562.8|OTTHUMG00000166322.5|OTTHUMT00000473544.1|AP000769.3-014|MALAT1|132|
CCAGCTGAGTGATAAAGGCTGAGTGTTGAGGAAATTTCTGCAGTTTTAAGCAGTCGTATTTGTGATTGAAGCTGAGTACATTTTGCTGGTGTATTTTTAG
GAACACTCAGCAGACACACGTATGCGAAGGGC
>ENST00000610851.1|ENSG00000251562.8|OTTHUMG00000166322.5|OTTHUMT00000473545.1|AP000769.3-015|MALAT1|584|
TGTTGAGGAAATTTCTGCAGTTTTAAGCAGTCGTATTTGTGATTGAAGCTGAGTACATTTTGCTGGTGTATTTTTAGGTAAAATGCTTTTTGTTCATTTCT
GGTGGTGGGAGGGGACTGAAGCCTTTAGTCTTTTCCAGATGCAACCTTAAAATCAGTGACAAGAAACATTCCAAACAAGCAACAGTCTTCAAGAAAT
TAAACTGGCAAGTGGAAATGTTTAAACAGTTCAGTGATCTTTAGTGCATTGTTTATGTGTGGGTTTCTCTCTCCCCTCCCTTGGTCTTAATTCTTACATGC

AGGAACACTCAGCAGACACACGTATGCGAAGGGCCAGAGAAGCCAGACCCAGTAAGAAAAAATAGCCTATTTACTTTAAATAAACCAAACATTCCATT
TTAAATGTGGGGATTGGGAACCACTAGTTCTTTCAGATGGTATTCTTCAGACTATAGAAGGAGCTTCCAGTTGAATTCACCAGTGGACAAAATGAGGAA
AACAGGTAATAGCCTGCAGCTGGTGTTTTGAGAAGCCCTACTGCTGAAAACTTAACAATTTTGTGTAATAAAAATGGAGAAGCTCTAA
>ENST00000618227.1|ENSG00000251562.8|OTTHUMG00000166322.5|OTTHUMT00000473546.1|AP000769.3-016|MALAT1|593|
CGTATGCGAAGGGCCAGAGAAGCCAGACCCAGTAAGAAAAAATAGCCTATTTACTTTAAATAAACCAAACATTCCATTTTAAATGTGGGGATTGGGAA
CCACTAGTTCTTTCAGATGGTATTCTTCAGACTATAGAAGGAGCTTCCAGTTGAATTCACCAGTGGACAAAATGAGGAAAACAGGTGAACAAGCTTTT
TCTGTATTTACATACAAAGTCAGATCAGTTATGGGACAATAGTATTGAATAGATTTCAGCTTTATGCTGGAGTAACTGGCATGTGAGCAAACTGTGTTGG
CGTGGGGGTGGAGGGGTGAGGTGGGCGCTAAGCCTTTTTTTAAGATTTTTCAGGTACCCCTCACTAAAGGCACCGAAGGCTTAAAGTAGGACAACCAT
GGAGCCTTCCTGTGGCAGGAGAGACAACAAAGCGCTATTATCCTAAGACCCTTCACCCCTCACCTCGATGCAGCCAGTAGCTTGGATCCTTGTGGGCA
TGATCCATAATCGGTTTCAAGGTAATAGCCTGCAGCTGGTGTTTTGAGAAGCCCTACTGCTGAAAACTTAACAATTTTGTGTAATAAAAATGGAGAAGC
TC
>ENST00000617489.1|ENSG00000251562.8|OTTHUMG00000166322.5|OTTHUMT00000473547.1|AP000769.3-003|MALAT1|318|
CACCGAAGGCTTAAAGTAGGACAACCATGGAGCCTTCCTGTGGCAGGAGAGACAACAAAGCGCTATTATCCTAAGCTTTTCCAGAAGCCTGTTAAAAG
CAAGGTCTCCCCACAAGCAACTTCTCTGCCACATCGCCACCCCGTGCCTTTTGATCTAGCACAGACCCTTCACCCCTCACCTCGATGCAGCCAGTAGCT
TGGATCCTTGTGGGCATGATCCATAATCGGTTTCAAGGTAATAGCCTGCAGCTGGTGTTTTGAGAAGCCCTACTGCTGAAAACTTAACAATTTTGTGTAA
TAAAAATGGAGAAGCTCTAAA
>ENST00000616691.1|ENSG00000251562.8|OTTHUMG00000166322.5|OTTHUMT00000473548.1|AP000769.3-017|MALAT1|587|
CGAAGGCTTAAAGTAGGACAACCATGGAGCCTTCCTGTGGCAGGAGAGACAACAAAGCGCTATTATCCTAAGGTCAAGAGAAGTGTCAGCCTCACCT
GATTTTTATTAGTAATGAGGACTTGCCTCAACTCCCTCTTTCTGGAGTGAAGCATCCGAAGGAATGCTTGAAGTACCCCTGGGCTTCTCTTAACATTTAA
GCAAGCTGTTTTTATAGCAGCTCTTAATAATAAAGCCCAAATCTCAAGCGGTGCTTGAAGGGGAGGGAAAGGGGGAAAGCGGGCAACCACTTTTCCCT
AGCTTTTCCAGAAGCCTGTTAAAAGCAAGGTCTCCCCACAAGCAACTTCTCTGCCACATCGCCACCCCGTGCCTTTTGATCTAGCACAGACCCTTCAC
CCCTCACCTCGATGCAGCCAGTAGCTTGGATCCTTGTGGGCATGATCCATAATCGGTTTCAAGGTAATAGCCTGCAGCTGGTGTTTTGAGAAGCCCTAC
TGCTGAAAACTTAACAATTTTGTGTAATAAAAATGGAGAAGCTCTAAATTGTTGTGGTTCTTTTGTGAATAAAAAAATCTTGATTGGGGAAAAAA
>XIST
>ENST00000434839.2|ENSG00000229807.10|OTTHUMG00000021839.5|OTTHUMT00000083628.2|RP13-216E22.1-005|XIST|2865|
AGCAGAAGATGGAATTAGACTGATGACACACTGTCCAGCTACTCAGCGAAGACCTGGGTGAATTAGCATGGCACTTCGCAGCTGTCTTTAGCCAGTCA

GGAGAAAGAAGTGGAGGGGCCACGTGTATGTCTCCCAGTGGGCGGTACACCAGGTGTTTTCAAGGACATTCTGAGCATGTGAGACCTGAGGACTGCA
AACAGCTATAAGAGGCTCCAAATTAATCATATCTTTCCCTTTGAGAATCTGGCCAAGCTCCAGCTAATCTACTTGGATGGGTTGCCAGCTATCTGGAGAA
AAAGATCTTCCTCAGAAGAATAGGCTTGTTGTTTTACAGTGTTAGTGATCCATTCCCTTTGACGATCCCTAGGTGGAGATGGGGCATGAGGATCCTCCA
GGGGAAAAGCTCACTACCACTGGGCAACAACCCTAGGTCAGGAGGTTCTGTCAAGATACTTTCCTGGTCCCAGATAGGAAGATAAAGTCTCAAAAAC
AACCACCACACGTCAAGCTCTTCATTGTTCCTATCTGCCAAATCATTATACTTCCTACAAGCAGTGCAGAGAGCTGAGTCTTCAGCAGGTCCAAGAAAT
TTGAACACACTGAAGGAAGTCAGCCTTCCCACCTGAAGATCAACATGCCTGGCACTCTAGCACTTGAGGATAGCTGAATGAATGTGTCTTACCCATTTC
CATGTTTCTCTTGCTAATTTCTTTCGTGTGTGCCTTTGCCTCATTTTCTCTTTTTGTTCACAAGAGTGGTCTGTGTCTTGTCTTAGACATATCTCTCATTTT
TCATTTTGTTGCTATTTCTCTTTGCTCTCCTAGATGTGGCTCTTCTTTCACGCTTTATTTCATGTCTCCTTTTTGGGTCACATGCTGTGTGCTTTTTGTCCTT
TTCTTGTTCTGTCTACCTCTCCTTTCTCTGCCTACCTCTCTTTTCTCTTTGTGAACTGTGATTATTTGTTACCCCTTCCCCTTCTCGTTCGTTTTAAATTTCA
CCTTTTTTCTGAGTCTGGCCTCCTTTCTGCTGTTTCTACTTTTTATCTCACATTTCTCATTTCTGCATTTCCTTTCTGCCTCTCTTGGGCTATTCTCTCTCTC
CTCCCCTGCGTGCCTCAGCATCTCTTGCTGTTTGTGATTTTCTATTTCAGTATTAATCTCTGTTGGCTTGTATTTGTTCTCTGCTTCTTCCCTTTCTACTCAC
CTTTGAGTATTTCAGCCTCTTCATGAATCTATCTCCCTCTCTTTGATTTCATGTAATCTCTCCTTAAATATTTCTTTGCATATGTGGGCAAGTGTACGTGTGT
GTGTGTCATGTGTGGCAGAGGGGCTTCCTAACCCCTGCCTGATAGGTGCAGAACGTCGGCTATCAGAGCAAGCATTGTGGAGCGGTTCCTTATGCCAG
GCTGCCATGTGAGATGATCCAAGACCAAAACAAGGCCCTAGACTGCAGTAAAACCCAGAACTCAAGTAGGGCAGAAGGTGGAAGGCTCATATGGATA
GAAGGCCCAAAGTATAAGACAGATGGTTTGAGACTTGAGACCCGAGGACTAAGATGGAAAGCCCATGTTCCAAGATAGATAGAAGCCTCAGGCCTGA
AACCAACAAAAGCCTCAAGAGCCAAGAAAACAGAGGGTGGCCTGAATTGGACCGAAGGCCTGAGTTGGATGGAAGTCTCAAGGCTTGAGTTAGAAG
TCTTAAGACCTGGGACAGGACACATGGAAGGCCTAAGAACTGAGACTTGTGACACAAGGCCAACGACCTAAGATTAGCCCAGGGTTGTAGCTGGAAG
ACCTACAACCCAAGGATGGAAGGCCCCTGTCACAAAGCCTACCTAGATGGATAGAGGACCCAAGCGAAAAAGGTATCTCAAGACTAACGGCCGGAAT
CTGGAGGCCCATGACCCAGAACCCAGGAAGGATAGAAGCTTGAAGACCTGGGGAAATCCCAAGATGAGAACCCTAAACCCTACCTCTTTTCTATTGTT
TACACTTCTTACTCTTAGATATTTCCAGTTCTCCTGTTTATCTTTAAGCCTGATTCTTTTGAGATGTACTTTTTGATGTTGCCGGTTACCTTTAGATTGACA
GTATTATGCCTGGGCCAGTCTTGAGCCAGCTTTAAATCACAGCTTTTACCTATTTGTTAGGCTATAGTGTTTTGTAAACTTCTGTTTCTATTCACATCTTCT
CCACTTGAGAGAGACACCAAAATCCAGTCAGTATCTAATCTGGCTTTTGTTAACTTCCCTCAGGAGCAGACATTCATATAGGTGATACTGTATTTCAGTC
CTTTCTTTTGACCCCAGAAGCCCTAGACTGAGAAGATAAAATGGTCAGGTTGTTGGGGAAAAAAAAGTGCCAGGCTCTCTAGAGAAAAATGTGAAGA
GATGCTCCAGGCCAATGAGAAGAATTAGACAAGAAATACACAGATGTGCCAGACTTCTGAGAAGCACCTGCCAGCAACAGCTTCCTTCTTTGAGCTTA
GTTTCTGGCATCACTACCACTACTGATTAAACAAGAATAAGAGAACATTTTATCATCATCTGCTTTATTCACATAAATGAAGTTGTGATGAATAAATCTGC
TTTTATGCAGACACAAGGAATTAAGTGGCTTCGTCATTGTCCTTCTACCTCAAAGATAATTTATTCCAAAAGCTAAGATAAATGGAAGACTCTTGAACTT

GTGAACTGATGTGAAATGCAGAATCTCTTTTGAGTCTTTGCTGTTTGGAAGATTGAAAAATATTGTTCAGCATGGGTGACCACCAGAAAGTAATCTTAA
GCCATCTAGATGTCACAATTGAAACAAACTGGGGAGTTGGTTGCTATTGTAAAATAAAATATACTGTTTTGAAAACTTTG
>ENST00000421322.1|ENSG00000229807.10|OTTHUMG00000021839.5|OTTHUMT00000057241.1|RP13-216E22.1-003|XIST|446|
TACAACCCAAGGATGGAAGGCCCCTGTCACAAAGCCTACCTAGATGGATAGAGGACCCAAGCGAAAAAGTTTCTGGCATCACTACCACTACTGATTAA
ACAAGAATAAGAGAACATTTTATCATCATCTGCTTTATTCACATAAATGAAGTTGTGATGAATAAATCTGCTTTTATGCAGACACAAGGAATTAAGTGGC
TTCGTCATTGTCCTTCTACCTCAAAGATAATTTATTCCAAAAGCTAAGATAAATGGAAGACTCTTGAACTTGTGAACTGATGTGAAATGCAGAATCTCTT
TTGAGTCTTTGCTGTTTGGAAGATTGAAAAATATTGTTCAGCATGGGTGACCACCAGAAAGTAATCTTAAGCCATCTAGATGTCACAATTGAAACAAAC
TGGGGAGTTGGTTGCTATTGTAAAATAAAATATACTGTTTTGAAAACTT
>ENST00000417942.5|ENSG00000229807.10|OTTHUMG00000021839.5|OTTHUMT00000083629.1|RP13-216E22.1-006|XIST|724|
GACCCCAGAAGCCCTAGACTGAGAAGATAAAATGGTCAGGTTGTTGGGGAAAAAAAAGTGCCAGGCTCTCTAGAGAAAAATGTGAAGAGATGCTCC
AGGCCAATGAGAAGAATTAGACAAGAAATACACAGATGTGCCAGACTTCTGAGAAGCACCTGCCAGCAACAGCTTCCTTCTTTGAGCTTAGACACTTC
ATTTTCCTAGTCCATCCCTCATGAAAAATGACTGACCACTGCTGGGCAGCAGGAGGGATGATGACCAACTAATTCCCAAACCCCAGTCTCATTGGTACC
AGCCTTGGGGAACCACCTACACTTGAGCCACAATTGGTTTTGAAGTGCATTTACAAGTTTCTGGCATCACTACCACTACTGATTAAACAAGAATAAGAG
AACATTTTATCATCATCTGCTTTATTCACATAAATGAAGTTGTGATGAATAAATCTGCTTTTATGCAGACACAAGGAATTAAGTGGCTTCGTCATTGTCCT
TCTACCTCAAAGATAATTTATTCCAAAAGCTAAGATAAATGGAAGACTCTTGAACTTGTGAACTGATGTGAAATGCAGAATCTCTTTTGAGTCTTTGCTG
TTTGGAAGATTGAAAAATATTGTTCAGCATGGGTGACCACCAGAAAGTAATCTTAAGCCATCTAGATGTCACAATTGAAACAAACTGGGGAGTTGGTT
GCTATTGTAAAATAAAATATACTGTTTTGAAAA
>ENST00000416330.1|ENSG00000229807.10|OTTHUMG00000021839.5|OTTHUMT00000057240.3|RP13-216E22.1-002|XIST|750|
CAGACATTCATATAGGTGATACTGTATTTCAGTCCTTTCTTTTGACCCCAGAAGCCCTAGACTGAGAAGATAAAATGGTCAGGTTGTTGGGGAAAAAAA
AGTGCCAGGCTCTCTAGAGAAAAATGTGAAGAGATGCTCCAGGCCAATGAGAAGAATTAGACAAGAAATACACAGATGTGCCAGACTTCTGAGAAGC
ACCTGCCAGCAACAGCTTCCTTCTTTGAGCTTAGTCCATCCCTCATGAAAAATGACTGACCACTGCTGGGCAGCAGGAGGGATGATGACCAACTAATT
CCCAAACCCCAGTCTCATTGGTACCAGCCTTGGGGAACCACCTACACTTGAGCCACAATTGGTTTTGAAGTGCATTTACAAGTTTCTGGCATCACTACC
ACTACTGATTAAACAAGAATAAGAGAACATTTTATCATCATCTGCTTTATTCACATAAATGAAGTTGTGATGAATAAATCTGCTTTTATGCAGACACAAG
GAATTAAGTGGCTTCGTCATTGTCCTTCTACCTCAAAGATAATTTATTCCAAAAGCTAAGATAAATGGAAGACTCTTGAACTTGTGAACTGATGTGAAAT
GCAGAATCTCTTTTGAGTCTTTGCTGTTTGGAAGATTGAAAAATATTGTTCAGCATGGGTGACCACCAGAAAGTAATCTTAAGCCATCTAGATGTCACA
ATTGAAACAAACTGGGGAGTTGGTTGCTATTGTAAAATAAAATATACTGTTTTGAAAA

>ENST00000429829.5|ENSG00000229807.10|OTTHUMG00000021839.5|OTTHUMT00000057239.1|RP13-216E22.1-001|XIST|19275|
CCTTCAGTTCTTAAAGCGCTGCAATTCGCTGCTGCAGCCATATTTCTTACTCTCTCGGGGCTGGAAGCTTCCTGACTGAAGATCTCTCTGCACTTGGGGT
TCTTTCTAGAACATTTTCTAGTCCCCCAACACCCTTTATGGCGTATTTCTTTAAAAAAATCACCTAAATTCCATAAAATATTTTTTTAAATTCTATACTTTCT
CCTAGTGTCTTCTTGACACGTCCTCCATATTTTTTTAAAGAAAGTATTTGGAATATTTTGAGGCAATTTTTAATATTTAAGGAATTTTTCTTTGGAATCATT
TTTGGTTGACATCTCTGTTTTTTGTGGATCAGTTTTTTACTCTTCCACTCTCTTTTCTATATTTTGCCCATCGGGGCTGCGGATACCTGGTTTTATTATTTTT
TCTTTGCCCAACGGGGCCGTGGATACCTGCCTTTTAATTCTTTTTTATTCGCCCATCGGGGCCGCGGATACCTGCTTTTTATTTTTTTTCCTTAGCCCATC
GGGGTATCGGATACCTGCTGATTCCCTTCCCCTCTGAACCCCCAACACTCTGGCCCATCGGGGTGACGGATATCTGCTTTTTAAAAATTTTCTTTTTTTG
GCCCATCGGGGCTTCGGATACCTGCTTTTTTTTTTTTTATTTTTCCTTGCCCATCGGGGCCTCGGATACCTGCTTTAATTTTTGTTTTTCTGGCCCATCGGG
GCCGCGGATACCTGCTTTGATTTTTTTTTTTCATCGCCCATCGGTGCTTTTTATGGATGAAAAAATGTTGGTTTTGTGGGTTGTTGCACTCTCTGGAATAT
CTACACTTTTTTTTGCTGCTGATCATTTGGTGGTGTGTGAGTGTACCTACCGCTTTGGCAGAGAATGACTCTGCAGTTAAGCTAAGGGCGTGTTCAGATT
GTGGAGGAAAAGTGGCCGCCATTTTAGACTTGCCGCATAACTCGGCTTAGGGCTAGTCGTTTGTGCTAAGTTAAACTAGGGAGGCAAGATGGATGATA
GCAGGTCAGGCAGAGGAAGTCATGTGCATTGCATGAGCTAAACCTATCTGAATGAATTGATTTGGGGCTTGTTAGGAGCTTTGCGTGATTGTTGTATCG
GGAGGCAGTAAGAATCATCTTTTATCAGTACAAGGGACTAGTTAAAAATGGAAGGTTAGGAAAGACTAAGGTGCAGGGCTTAAAATGGCGATTTTGAC
ATTGCGGCATTGCTCAGCATGGCGGGCTGTGCTTTGTTAGGTTGTCCAAAATGGCGGATCCAGTTCTGTCGCAGTGTTCAAGTGGCGGGAAGGCCACA
TCATGATGGGCGAGGCTTTGTTAAGTGGTTAGCATGGTGGTGGACATGTGCGGTCACACAGGAAAAGATGGCGGCTGAAGGTCTTGCCGCAGTGTAA
AACATGGCGGGCCTCTTTGTCTTTGCTGTGTGCTTTTCGTGTTGGGTTTTGCCGCAGGGACAATATGGCAGGCGTTGTCATATGTATATCATGGCTTTTGT
CACGTGGACATCATGGCGGGCTTGCCGCATTGTTAAAGATGGCGGGTTTTGCCGCCTAGTGCCACGCAGAGCGGGAGAAAAGGTGGGATGGACAGTG
CTGGATTGCTGCATAACCCAACCAATTAGAAATGGGGGTGGAATTGATCACAGCCAATTAGAGCAGAAGATGGAATTAGACTGATGACACACTGTCCA
GCTACTCAGCGAAGACCTGGGTGAATTAGCATGGCACTTCGCAGCTGTCTTTAGCCAGTCAGGAGAAAGAAGTGGAGGGGCCACGTGTATGTCTCCC
AGTGGGCGGTACACCAGGTGTTTTCAAGGTCTTTTCAAGGACATTTAGCCTTTCCACCTCTGTCCCCTCTTATTTGTCCCCTCCTGTCCAGTGCTGCCTC
TTGCAGTGCTGGATATCTGGCTGTGTGGTCTGAACCTCCCTCCATTCCTCTGTATTGGTGCCTCACCTAAGGCTAAGTATACCTCCCCCCCCACCCCCCA
ACCCCCCCAACTCCCCACCCCCACCCCCCACCCCCCACCTCCCCACCCCCCTACCCCCCTACCCCCCTACCCCCCTCTGGTCTGCCCTGCACTGCACTG
TTGCCATGGGCAGTGCTCCAGGCCTGCTTGGTGTGGACATGGTGGTGAGCCGTGGCAAGGACCAGAATGGATCACAGATGATCGTTGGCCAACAGGT
GGCAGAAGAGGAATTCCTGCCTTCCTCAAGAGGAACACCTACCCCTTGGCTAATGCTGGGGTCGGATTTTGATTTATATTTATCTTTTGGATGTCAGTCA
TACAGTCTGATTTTGTGGTTTGCTAGTGTTTGAATTTAAGTCTTAAGTGACTATTATAGAAATGTATTAAGAGGCTTTATTTGTAGAATTCACTTTAATTAC
ATTTAATGAGTTTTTGTTTTGAGTTCCTTAAAATTCCTTAAAGTTTTTAGCTTCTCATTACAAATTCCTTAACCTTTTTTTGGCAGTAGATAGTCAAAGTCA

AATCATTTCTAATGTTTTAAAAATGTGCTGGTCATTTTCTTTGAAATTGACTTAACTATTTTCCTTTGAAGAGTCTGTAGCACAGAAACAGTAAAAAATTT
AACTTCATGACCTAATGTAAAAAAGAGTGTTTGAAGGTTTACACAGGTCCAGGCCTTGCTTTGTTCCCATCCTTGATGCTGCACTAATTGACTAATCACC
TACTTATCAGACAGGAAACTTGAATTGCTGTGGTCTGGTGTCCTCTATTCAGACTTATTATATTGGAGTATTTCAATTTTTCGTTGTATCCTGCCTGCCTAG
CATCCAGTTCCTCCCCAGCCCTGCTCCCAGCAAACCCCTAGTCTAGCCCCAGCCCTACTCCCACCCCGCCCCAGCCCTGCCCCAGCCCCAGTCCCCTAA
CCCCCCAGCCCTAGCCCCAGTCCCAGTCCTAGTTCCTCAGTCCCGCCCAGCTTCTCTCGAAAGTCACTCTAATTTTCATTGATTCAGTGCTCAAAATAAG
TTGTCCATTGCTTATCCTATTATACTGGGATATTCCGTTTACCCTTGGCATTGCTGATCTTCAGTACTGACTCCTTGACCATTTTCAGTTAATGCATACAATC
CCATTTGTCTGTGATCTCAGGACAAAGAATTTCCTTACTCGGTACGTTGAAGTTAGGGAATGTCAATTGAGAGCTTTCTATCAGAGCATTATTGCCCACA
ATTTGAGTTACTTATCATTTTCTCGATCCCCTGCCCTTAAAGGAGAAACCATTTCTCTGTCATTGCTTCTGTAGTCACAGTCCCAATTTTGAGTAGTGATC
TTTTCTTGTGTACTGTGTTGGCCACCTAAAACTCTTTGCATTGAGTAAAATTCTAATTGCCAATAATCCTACCCATTGGATTAGACAGCACTCTGAACCCC
ATTTGCATTCAGCAGGGGGTCGCAGACAACCCGTCTTTTGTTGGACAGTTAAAATGCTCAGTCCCAATTGTCATAGCTTTGCCTATTAAACAAAGGCAC
CCTACTGCGCTTTTTGCTGTGCTTCTGGAGAATCCTGCTGTTCTTGGACAATTAAAGAACAAAGTAGTAATTGCTAATTGTCTCACCCATTAATCATGAA
GACTACCAGTCGCCCTTGCATTTGCCTTGAGGCAGCGCTGACTACCTGAGATTTAAGAGTTTCTTAAATTATTGAGTAAAATCCCAATTATCCATAGTTC
TGTTAGTTACACTATGGCCTTTGCAAACATCTTTGCATAACAGCAGTGGGACTGACTCATTCTTAGAGCCCCTTCCCTTGGAATATTAATGGATACAATAG
TAATTATTCATGGTTCTGCGTAACAGAGAAGACCCACTTATGTGTATGCCTTTATCATTGCTCCTAGATAGTGTGAACTACCTACCACCTTGCATTAATATG
TAAAACACTAATTGCCCATAGTCCCACTCATTAGTCTAGGATGTCCTCTTTGCCATTGCTGCTGAGTTCTGACTACCCAAGTTTCCTTCTCTTAAACAGTT
GATATGCATAATTGCATATATTCATGGTTCTGTGCAATAAAAATGGATTCTCACCCCATCCCACCTTCTGTGGGATGTTGCTAACGAGTGCAGATTATTCA
ATAACAGCTCTTGAACAGTTAATTTGCACAGTTGCAATTGTCCAGAGTCCTGTCCATTAGAAAGGGACTCTGTATCCTATTTGCACGCTACAATGTGGGC
TGATCACCCAAGGACTCTTCTTGTGCATTGATGTTCATAATTGTATTTGTCCACGATCTTGTGCACTAACCCTTCCACTCCCTTTGTATTCCAGCAGGGGA
CCCTTACTACTCAAGACCTCTGTACTAGGACAGTTTATGTGCACAATCCTAATTGATTAGAACTGAGTCTTTTATATCAAGGTCCCTGCATCATCTTTGCT
TTACATCAAGAGGGTGCTGGTTACCTAATGCCCCTCCTCCAGAAATTATTGATGTGCAAAATGCAATTTCCCTATCTGCTGTTAGTCTGGGGTCTCATCCC
CTCATATTCCTTTTGTCTTACAGCAGGGGGTACTTGGGACTGTTAATGCGCATAATTGCAATTATGGTCTTTTCCATTAAATTAAGATCCCAACTGCTCAC
ACCCTCTTAGCATTACAGTAGAGGGTGCTAATCACAAGGACATTTCTTTTGTACTGTTAATGTGCTACTTGCATTTGTCCCTCTTCCTGTGCACTAAAGA
CCCCACTCACTTCCCTAGTGTTCAGCAGTGGATGACCTCTAGTCAAGACCTTTGCACTAGGATAGTTAATGTGAACCATGGCAACTGATCACAACAATG
TCTTTCAGATCAGATCCATTTTATCCTCCTTGTTTTACAGCAAGGGATATTAATTACCTATGTTACCTTTCCCTGGGACTATGAATGTGCAAAATTCCAATG
TTCATGGTCTCTCCCTTTAAACCTATATTCTACCCCTTTTACATTATAGAAAGGGATGCTGGAAACCCAGAGTCCTTCTCTTGGGACTCTTAATGTGTATT
TCTAATTATCCATGACTCTTAATGTGCATATTTTCAATTGCCTAATTGATTTCAATTGTCTAAGACATTTCAAATGTCTAATTGATTAGAACTGAGTCTTTTA

TATCAAGCTAATATCTAGCTTTTATATCAAGCTAATATCTTGACTTCTCAGCATCATAGAAGGGGGTACTGATTTCCTAAAGTCTTTCTTGAATTTCTATTAT
GCAAAATTGCCCTGAGGCCGGGTGTGGTGGCTCACACCTGTAATCCCAGCACTTTGGGAGGCTGAGGTGGGAAGATCCCTTACTGCCAGGAGTTTGA
GACCAGCCTGGCCAACATTAAAAAAAAAAAAAAAGTAAGACAATTGCCCTGGAATCCCATCCCCCTCACACCTCCTTGGCAAAGCAGCAGGAGTGCTA
ACTAGCTAGTGCTTCTTCTCTTATACTGCTTAAATGCGCATAATTAGCAGTAGTTGATGTGCCCCTATGTTAGAGTAGAATCCCGCTTCCTTGCTCCATTTG
CATTACTGCAGGAGCTTCTAACTAGCCTGAATTCACTCTCTTGGACTGTTAATGTGCATACTTATATTTGCTGCTGTACTTTTTTACCATGTAAGGACCCC
ACCCACTGTATTTACATCCCAGCTGGAAGTACCTACTACTTAAGACCCTTAGACTAGTAAAGTTAGCGTGCATAATCTTAGGTGTTATATACACATTTTCA
GTTGCATACAGTTGTGCCTTTTATCAGGACTCCTGTACTTATCAAAGCAGAGAGTGCTAATCAATATTAAGCCCTTCTCTTCGAACTGTAGATGGCATGT
AATTGCAGTTGTCAATGGTCCTTCAATTAGACTTGGGTTTCTGACCTATCACACCCTCTTTGCTTTATTGCATGGGGTACTATTCACTTAAGGCCCCTTTC
TCAAACTGTTAATGTGCCTAATGACAATTACATCAGTATCCTTCCTTTTGAAGGACAGCATGGTTGGTGACACCTAAGGCCCCATTTCTTGGCCTCCCAA
TATGTGTGATTGTATTTGTCGAGGTTGCTATGCACTAGAGAAGGAAAGTGCTCCCCTCATCCCCACTTTTCCCTTCCAGCAGGAAGTGCCCACCCCATA
AGACCCTTTTATTTGGAGAGTCTAGGTGCACAATTGTAAGTGACCACAAGCATGCATCTTGGACATTTATGTGCGTAATCGCACACTGCTCATTCCATGT
GAATAAGGTCCTACTCTCCGACCCCTTTTGCAATACAGAAGGGTTGCTGATAACGCAGTCCCCTTTTCTTGGCATGTTGTGTGTGATTATAATCGTCTGG
GATCCTATGCACTAGAAAAGGAGGGTCCTCTCCACATACCTCAGTCTCACCTTTCCCTTCCAGCAGGGAGTGCCCACTCCATAAGACTCTCACATTTGG
ACAGTCAAGGTGCGTAATTGTTAAGTGAACACAACCATGCACCTTAGACATGGATTTGCATAACTACACACAGCTCAACCTATCTGAATAAAATCCTAC
TCTCAGACCCCTTTTGCAGTACAGCAGGGGTGCTGATCACCAAGGCCCTTTTTCCTGGCCTGGTATGCGTGTGATTATGTTTGTCCCGGTTCCTGTGTAT
TAGACATGGAAGCCTCCCCTGCCACACTCCACCCCCAATCTTCCTTTCCCTTCCGGCAGGGAGTGCCCTCTCCATAAGACGCTTACGTTTGGACAATCA
AGGTGCACAGTTGTAAGTGACCACAGGCATACACCTTGGACATTAATGTGCATAACCACTTTGCCCATTCCATCTGAATAAGGTCCTACTCTCAGACCC
CTTTTGCAGTACAGCAGGGGTGCTGATCACCAAGGCCCCTTTTCTTGGCCTGTTATGTGCGTGATTATATTTGTCTGGGTTCCTGTGTATTAGACAAGGA
AGCCTTCCCCCCGCCCCCACCCCCACTCCCAGTCTTCCTTTCCCTTCCAGCAGGGAGTGCCCCCTCCATAAGATCATTACATTTGGACAATCAAGGTGC
ACAATTATAAGTGACCACAGCCATGCACCTTGGACATTATTGGACATTAATGTGCGTAACTGCACATGGCCCATCCCATCTGAATAAGGTCCTACTCTCA
GATGCCCTTTGCAGTACAGCAGGGGTACTGAATCACCAAGGCCCTTTTTCTTGGCCTGTTATGTGTGTGATTATATTTATCCCAGTTTCTGTGTAATAGAC
ATGAAAGCCTCCCCTGCCACACCCCACCTCCAATCTTCCTTTCCCTTCCACCAGGGAGTGTCCACTCCATATACCCTTACATTTGGACAATCAAGGTGC
ACAATTGTAAGTGAGCATAGGCACTCACCTTGGACATGAATGTGCATAACTGCACATGGCCCATCCCATCTGAATAAGGTCCTACTCTCAGACCCTTTTT
GCAGTACAGCAGGGGTGCTGATCACCAAGGCCCCTTTTCCTGGCCTGTTATGTGTGTGATTATATTTGTTCCAGTTCCTGTGTAATAGACATGGAAGCCT
CCCCTGCCACACTCCACCCCCAATCTTCCTTTCCCTTCTGGCAGGAAGTACCCGCTCCATAAGACCCTTACATTTGGACAGTCAAGGTGCACAATTGTA
TGTGACCACAACCATGCACCTTGGACATAAATGTGTGTAACTGCACATGGCCCATCCCATCTGAATAAGGTCCTACTCTCAGACCCCTTTTGCAGTACA

GTAGGTGTGCTGATAACCAAGGCCCCTCTTCCTGGCCTGTTAACGTATGTGATTATATTTGTCTGGGTTCCAGTGTATAAGACATGGAAGCCTCCCCTGC
CCCACCCCACCCTCAATCTTCCTTTCCCTTCTGGCAGGGAGTGCCAGCTCCATAAGAACCTTACATTTGGACAGTCAAGGTGCACAATTCTAAGTGACC
GCAGCCATGCACCTTGGTCAATAATGTGTGTAACTGCACACGGCCTATCTCATCTGAATAAGGCCTTACTCTCAGACCCCTTTTGCAGTACAGCAGGGG
TGCTGATAACCAAGGCCCATTTTCCTGGCCTGTTATGTGTGTGATTATATTTGTCCAGGTTTCTGTGTACTAGACAAGGAAGCCTCCTCTGCCCCATCCCA
TCTACGCATAATCTTTCTTTTCCTCCCAGCAGGGAGTGCTCACTCCATAAGACCCTTACATTTGGACAATCAAGGTGCACAATTGTAAGTGACCACAAC
CATGCATCTTGGAAATTTATGTGCATAACTGCACATGGCTTATCCTATTTGAATAAAGTCCTACTCTCAGACCCCCTTTGCAGTATAGCTGGGGTGCTGAT
CACTGAGGCCTCTTTGCTTGGCTTGTCTATATTCTTGTGTACTAGATAAGGGCACCTTCTCATGGACTCCCTTTGCTTTTCAACAAGGAGTACCCACTAC
TTTTTAAGATTCTTATATTTGTCCAAAGTACATGGTTTTAATTGACCACAACAATGTCCCTTGGACATTAATGTATGTAATCACCACATGGTTCATCCTAAT
TAAACAAAGTTCTACCTTCTCACCCTCCATTTGCAGTATACCAGGGTTGCTGACCCCCTAAGTCCCCTTTTCTTGGCTTGTTGACATGCATAATTGCATTT
ATGTTGGTTCTTGTGCCCTAGACAAGGATGCCCCACCTCTTTTCAATAGTGGGTGCCCACTCCTTATGATCTTTACATTTGAACAGTTAATGTGAATAATT
GCAGTTGTCCACAACCCTATCACTTCTAGGACCATTATACCTCTTTTGCATTACTGTGGGGTATACTGTTTCCCTCCAAGGCCCCTTCTGGTGGACTATCA
ACATATAATTGAAATTTTCTTTTGTCTTTGTCAGTAGATTAAGGTCATACCCCATCACCTTTCCTTTGTAGTACAACAGGGTGTCCTGATCAACCAAAGTC
CTGTTGTTTTGGACTGTTAATATGTGCAATTACATTTGCTCCTGATCTGTGCACTAGATAAGGATCCTACCTACTTTCTTAGTGTTTTTAGCAGGTAGTGC
CCACTACTCAAGACTGTCACTTGGAATGTTCATGTGCACAAACTCAATTCTCTAAGCATGTTCCTGTACCACCTTTGCTTTAGAGCAGGGGGATGATATT
CACTAAGTGCCCCTTCTTTTGGACTTAATATGCATTAATGCAATTGTCCACCTCTTCTTTTAGACTAAGAGTTGATCTCCACATATTCCCCTTGCATCAGG
GGCATGTTAATTATGAATGAACCCTTTTCTTTTAATATTAATGTCATAATTGTATTTGTGGACCTGTGTAGGAGAAAAAGACCCTATGTTCCTCCCATTACC
CTTTGGATTGCTGCTGAGAAGTGTTAACTACTCATAATCTCAGCTCTTGGACAATTAATAGCATTAATAACAATTATCAAGGGCACTGATCATTAGATAAG
ACTCCTGCTTCCTCGTTGCTTACATCGGGGGTACTGACCCACTAAGGCCCCTTGTACTGTTAATGTGAATATTTGCAATTATATATGTCTCCTTCTGGTAG
AGTGGGATATTATGCCCTAGTATCCCCTTTGCATTACTGCAGGGGCTGCTGACTACTCAAAACTTCTCCTGGGACTGTTAATAGGCACAATGGCAGTTAT
CAATGGTTTTCTCCCTCCCTGACCTTGTTAAGCAAGCGCCCCACCCCACCCTTAGTTTCCCATGGCATAATAAAGTATAAGCATTGGAGTATTCCATGCA
CTTGTCTATCAAACAGTGGTCCATACTCCCAACCCTTTTGCATTGCGCCAGTGTGTAAAATCACAGGTAGCCATGGTGTCATGCTTTATATACGAAGTCT
TCCCTCTCTCTGCCCCTTGTGTGCCCTTGGCCCCTTTTTACAGACTATTGCTCACAATCTCAGGTGTCCATATTTGCAGCTATTAGGTAAGATTGTGCTGT
CTCCCTCTTCCCTTCCCTCTGCCCTGCCCCTTTTGCCTCTTTGCTGGGTAATGTTGACCAGACAAGGCCCTTTCTCTTGGACTTAAACAATTCTCAGTTG
CACTTTCCTTGGTCCCACCCATTATACATGAACCCCTCTACTTCCTTTCGCATTGCTTCTGAGTATGCTGACTACCCAAAGCCCCTTCTGTGTTATTAATA
AACACAGTACTGATTGTCCCATTTTTCAGCCCATCAGTCCAAGATCTCCCTACCACTTTGGTGTGTTGGTGCAGTGTTGACTATGAAAAGCAGGCCTGA
ACTAGGTGGATAAGCCTTCACTCATTTTCTTTCATTTATTAATGATCCTAGTTTCAATTATTGTCAGATTCTGGGGACAAGAACCATTCTTGCCCACCTGT

GTTACTGCTTTACTGTGCAAAATACTGAAGGCAAGTCAGACCCAGGGAGCTGGATTGCCATCCTTTATTTTGTGTTTCCAGTGTACACTATAAAATTGTC
TCCCCAGGAAGGAAGGTTGGCACTTTCTCTGCATTCTTCTTTCCAGAGCAGATTGCCTGGTTAAGAATCTCTTGTTGTCCCCTTTGTATATTGTTATTGTA
AAGTGCCAAATGCCAGGATACAGCCAGAAAAATTGCTTATTATTATTAAAAAAATTTTTTTAAGAAAGACATCTGGATTGTAGGGTGGACTCGATAACC
TGGTCATTATTTTTTTGAAGCCAAAATATCCATTTATACTATGTACCTGGTGACCAGTGTCTCTCATTTTAACTGAGGGTGGTGGGTCTGTGGATAGAACA
CTGACTCTTGCTATTTTAATATCAAAGATATTCTAGAGTGGAACTCTTAAGACCAGTATCTTTGTGTGGGCTTTACCAGCATTCACTTTTAGAAAAACTAC
CTAAATTTTATAATCCTTTAATTTCTTCATCTGGAGCACCTGCCCCTACTTATTTCAAGAAGATTGCAGTAAAACGATTAAATGAGGGAACATATGCAGAG
GTGCTTTTAAAAAGCATATGCCACCTTTTTTATTAATTATTATATAAAATGAAGCATTTAATTATAGTAATAATTTGAAGTAGTTTGAAGTACCACACTGAG
GTGAGGACTTAAAAATGATAAGACGAGTTCCCTATTTTATAAGAAAAATAAGCCAAAATTAAATATTCTTTTGGATATAAATTTCAACAGTGAGATAGCT
GCCTAGTGGAAATGAATAATATCCCAGCCACTAGTGTACAGGGTGTTTTGTGGCACAGGATTATGTAATATGGAACTGCTCAAGCAAATAACTAGTCATC
ACAACAGCAGTTCTTTGTAATAACTGAAAAAGAATATTGTTTCTCGGAGAAGGATGTCAAAAGATCGGCCCAGCTCAGGGAGCAGTTTGCCCTACTAG
CTCCTCGGACAGCTGTAAAGAAGAGTCTCTGGCTCTTTAGAATACTGATCCCATTGAAGATACCACGCTGCATGTGTCCTTAGTAGTCATGTCTCCTTAG
GCTCCTCTTGGACATTCTGAGCATGTGAGACCTGAGGACTGCAAACAGCTATAAGAGGCTCCAAATTAATCATATCTTTCCCTTTGAGAATCTGGCCAA
GCTCCAGCTAATCTACTTGGATGGGTTGCCAGCTATCTGGAGAAAAAGATCTTCCTCAGAAGAATAGGCTTGTTGTTTTACAGTGTTAGTGATCCATTCC
CTTTGACGATCCCTAGGTGGAGATGGGGCATGAGGATCCTCCAGGGGAAAAGCTCACTACCACTGGGCAACAACCCTAGGTCAGGAGGTTCTGTCAA
GATACTTTCCTGGTCCCAGATAGGAAGATAAAGTCTCAAAAACAACCACCACACGTCAAGCTCTTCATTGTTCCTATCTGCCAAATCATTATACTTCCTA
CAAGCAGTGCAGAGAGCTGAGTCTTCAGCAGGTCCAAGAAATTTGAACACACTGAAGGAAGTCAGCCTTCCCACCTGAAGATCAACATGCCTGGCAC
TCTAGCACTTGAGGATAGCTGAATGAATGTGTATTTCTTTGTCTCTTTCTTTCTTGTCTTTGCTCTTTGTTCTCTATCTAAAGTGTGTCTTACCCATTTCCA
TGTTTCTCTTGCTAATTTCTTTCGTGTGTGCCTTTGCCTCATTTTCTCTTTTTGTTCACAAGAGTGGTCTGTGTCTTGTCTTAGACATATCTCTCATTTTTC
ATTTTGTTGCTATTTCTCTTTGCTCTCCTAGATGTGGCTCTTCTTTCACGCTTTATTTCATGTCTCCTTTTTGGGTCACATGCTGTGTGCTTTTTGTCCTTTT
CTTGTTCTGTCTACCTCTCCTTTCTCTGCCTACCTCTCTTTTCTCTTTGTGAACTGTGATTATTTGTTACCCCTTCCCCTTCTCGTTCGTTTTAAATTTCACC
TTTTTTCTGAGTCTGGCCTCCTTTCTGCTGTTTCTACTTTTTATCTCACATTTCTCATTTCTGCATTTCCTTTCTGCCTCTCTTGGGCTATTCTCTCTCTCCT
CCCCTGCGTGCCTCAGCATCTCTTGCTGTTTGTGATTTTCTATTTCAGTATTAATCTCTGTTGGCTTGTATTTGTTCTCTGCTTCTTCCCTTTCTACTCACCT
TTGAGTATTTCAGCCTCTTCATGAATCTATCTCCCTCTCTTTGATTTCATGTAATCTCTCCTTAAATATTTCTTTGCATATGTGGGCAAGTGTACGTGTGTGT
GTGTCATGTGTGGCAGAGGGGCTTCCTAACCCCTGCCTGATAGGTGCAGAACGTCGGCTATCAGAGCAAGCATTGTGGAGCGGTTCCTTATGCCAGGC
TGCCATGTGAGATGATCCAAGACCAAAACAAGGCCCTAGACTGCAGTAAAACCCAGAACTCAAGTAGGGCAGAAGGTGGAAGGCTCATATGGATAGA
AGGCCCAAAGTATAAGACAGATGGTTTGAGACTTGAGACCCGAGGACTAAGATGGAAAGCCCATGTTCCAAGATAGATAGAAGCCTCAGGCCTGAAA

CCAACAAAAGCCTCAAGAGCCAAGAAAACAGAGGGTGGCCTGAATTGGACCGAAGGCCTGAGTTGGATGGAAGTCTCAAGGCTTGAGTTAGAAGTC
TTAAGACCTGGGACAGGACACATGGAAGGCCTAAGAACTGAGACTTGTGACACAAGGCCAACGACCTAAGATTAGCCCAGGGTTGTAGCTGGAAGA
CCTACAACCCAAGGATGGAAGGCCCCTGTCACAAAGCCTACCTAGATGGATAGAGGACCCAAGCGAAAAAGGTATCTCAAGACTAACGGCCGGAATC
TGGAGGCCCATGACCCAGAACCCAGGAAGGATAGAAGCTTGAAGACCTGGGGAAATCCCAAGATGAGAACCCTAAACCCTACCTCTTTTCTATTGTTT
ACACTTCTTACTCTTAGATATTTCCAGTTCTCCTGTTTATCTTTAAGCCTGATTCTTTTGAGATGTACTTTTTGATGTTGCCGGTTACCTTTAGATTGACAG
TATTATGCCTGGGCCAGTCTTGAGCCAGCTTTAAATCACAGCTTTTACCTATTTGTTAGGCTATAGTGTTTTGTAAACTTCTGTTTCTATTCACATCTTCTC
CACTTGAGAGAGACACCAAAATCCAGTCAGTATCTAATCTGGCTTTTGTTAACTTCCCTCAGGAGCAGACATTCATATAGGTGATACTGTATTTCAGTCC
TTTCTTTTGACCCCAGAAGCCCTAGACTGAGAAGATAAAATGGTCAGGTTGTTGGGGAAAAAAAAGTGCCAGGCTCTCTAGAGAAAAATGTGAAGAG
ATGCTCCAGGCCAATGAGAAGAATTAGACAAGAAATACACAGATGTGCCAGACTTCTGAGAAGCACCTGCCAGCAACAGCTTCCTTCTTTGAGCTTAG
GTGAGCAGGATTCTGGGGTTTGGGATTTCTAGTGATGGTTATGGAAAGGGTGACTGTGCCTGGGACAAAGCGAGGTCCCAAGGGGACAGCCTGAACT
CCCTGCTCATAGTAGTGGCCAAATAATTTGGTGGACTGTGCCAACGCTACTCCTGGGTTTAATACCCATCTCTAGGCTTAAAGATGAGAGAACCTGGGA
CTGTTGAGCATGTTTAATACTTTCCTTGATTTTTTTCTTCCTGTTTATGTGGGAAGTTGATTTAAATGACTGATAATGTGTATGAAAGCACTGTAAAACATA
AGAGAAAAACCAATTAGTGTATTGGCAATCATGCAGTTAACATTTGAAAGTGCAGTGTAAATTGTGAAGCATTATGTAAATCAGGGGTCCACAGTTTTT
CTGTAAGGGGTCAAATCATAAATACTTTAGACTGTGGGCCATATGGTTTCTGTTACATATTTGTTTTTTAAACAACGTTTTTATAAGGTCAAAATCATTCTT
AGTTTTTGAGCCAATTGGATTTGGCCTGCTGTTCATAGCTTACCACCCCCTGATGTATTATTTGTTATTCAGAGAAAATTTCTGAATACTACTAGTTTCCTT
TTCTGTGCCTGTCCCTGTGCTAGGCACTAAAAATGCAATGATTATTGATATCTAGGTGACCTGAAAAAAAAATAGTGAATGTGCTTTGTAAACTGTAAAGC
ACTTGTATTCTACTGTGATAAGCGTTGTGGATACAAAGAAAGGAGCAAGCATAAAAAAGTGCTCTTTCAAAAGGATATAGTACTATGCAGACACAAGG
AATTGTTTGATAAATGAATAAATTATATGTATATTTGAGGCCAATTTGTGTTTGCTGCTCTGGTAATTTTGAGTAAAAATGCAGTATTCCAGGTATCAGAA
ACGAAAACACATGGAAACTGCTTTTAAACTTTAAAATATACTGAAAACATAAGGGACTAAGCTTGTTGTGGTCACCTATAATGTGCCAGATACCATGCT
GGGTGCTAGAGCTACCAAAGGGGGAAAAGTATTCTCATAGAACAAAAAATTTCAGAAAGGTGCATATTAAAGTGCTTTGTAAACTAAAGCATGATACA
AATGTCAATGGGCTACATATTTATGAATGAATGAATGGATGAATGAATATTAAGTGCCTCTTACATACCAGCTATTTTGGGTACTGTAAAATACAAGATTA
ATTCTCCTATGTAATAAGAGGAAAGTTTATCCTCTATACTATTCAGATGTAAGGAATGATATATTGCTTAATTTTAAACAATCAAGACTTTACTGGTGAGGT
TAAGTTAAATTATTACTGATACATTTTTCCAGGTAACCAGGAAAGAGCTAGTATGAGGAAATGAAGTAATAGATGTGAGATCCAGACCGAAAGTCACTT
AATTCAGCTTGCGAATGTGCTTTCTAAATTATAAAGCACTTGTAAATGAAAAATTTGATGCTTTCTGTATGAATAAAACTTTCTGTAAGCTAGGTATTGTC
TCTACAAAATTCTCATTGTATAGTTAAACCACAGTGAGAAGGGTTCTATAAGTAGTTATACAAACCAAGGGTTTAAATACCTGTTAAATAGATCAATTTTG
ATTGCCTACTATGTGAACTCACTGTTAAAGGCACTGAAAATTTATCATATTTCATTTAGCCACAGCCAAAAATAAGGCAATACCTATGTTAGCATTTTGTG

AACTCTAAGGCACCATATAAATGTAACTGTTGATTTTCTCACTTGGTGCTGGGTACTAGGTTTATAAAATTGTATGATAGTTATTATATTGTGCAAATAAAG
TAGGAAAATTTGAATAACAATGATTATCTTTTGAATACGCATACGCAAGGGATTGGTTGTCTGAAGAATGCCACTATAGTAGTTATCTATTGTGTGCCAAT
CTCATTGCTAGGCATTGGGGATGCAAAGATAAACCATCTTTATTGTGTCTTGGGTAGCAGAAGAAAATATGTGTAAAATCAATTTATAATTTGTAAACTG
CCACCCATATATAAGCTATATCTGCTGAATGATCATTGATTACTCTTATCCTTAGAGATAACAACTGGGGGCACAAACATTTATTATCATTATTGAACCTAC
AACAGAGATCTATGTGTAGATTTACAAAGCCTACAGTTCTATACAGATAGGAATGAACTATTGGCTTACTGAATGGTGATTACTTTCTGTGGGGCTCGGA
ACTACATGCCCTAGGATATAAAAATGATGTTATCATTATAGAGTGCTCACAGAAGGAAATGAAGTAATATAGGTGTGAGATCCAGACCAAAAGTCATTTA
ACAAGTTTATTCAGTGATGAAAACATGGGACAAATGGACTAATATAAGGCAGTGTACTAAGCTGAGTAGAGAGATAAAGTCCTGTCCAGAAGATACAT
GCTTCCTGGCCTGATTGAGGAGATGGAAAATTTTTGCAAAAAACAAGGTGTTGTGGTCTTCCATCCAGTTTCTTAAGTGCTGATGATAAAAGTGAATTA
GACCCACCTTGACCTGGCCTACAGAAGTAAAGGAGTAAAAATAAATGCCTCAGGCGTGCTTTTTGATTCATTTGATAAACAAAGCATCTTTTATGTGGA
ATATACCATTCTGGGTCCTGAGGATAAGAGAGATGAGGGCATTAGATCACTGACAGCTGAAGATAGAAGAACATCTTTGGTTTGATTGTTTAAATAATAT
TTCAATGCCTATTCTCTGCAAGGTACTATGTTTCGTAAATTAAATAGGTCTGGCCCAGAAGACCCACTCAATTGCCTTTGAGATTAAAAAAAAAAAAAA
AAAGAAAGAAAAATGCAAGTTTCTTTCAAAATAAAGAGACATTTTTCCTAGTTTCAGGAATCCCCCAAATCACTTCCTCATTGGCTTAGTTTAAAGCCA
GGAGACTGATAAAAGGGCTCAGGGTTTGTTCTTTAATTCATTAACTAAACATTCTGCTTTTATTACAGTTAAATGGTTCAAGATGTAACAACTAGTTTTA
AAGGTATTTGCTCATTGGTCTGGCTTAGAGACAGGAAGACATATGAGCAATAAAAAAAAGATTCTTTTGCATTTACCAATTTAGTAAAAATTTATTAAAA
CTGAATAAAGTGCTGTTCTTAAGTGCTTGAAAGACGTAAACCAAAGTGCACTTTATCTCATTTATCTTATGGTGGAAACACAGGAACAAATTCTCTAAG
AGACTGTGTTTCTTTAGTTGAGAAGAAACTTCATTGAGTAGCTGTGATATGTTCGATACTAAGGAAAAACTAAACAGATCACCTTTGACATGCGTTGTA
GAGTGGGAATAAGAGAGGGCTTTTTATTTTTTCGTTCATACGAGTATTGATGAAGATGATACTAAATGCTAAATGAAATATATCTGCTCCAAAAGGCATTT
ATTCTGACTTGGAGATGCAACAAAAACACAAAAATGGAATGAAGTGATACTCTTCATCAAACAGAAGTGACTGTTATCTCAACCATTTTGTTAAATCCT
AAACAGAAAACAAAAAAAATCATGACGAAAAGACACTTGCTTATTAATTGGCTTGGAAAGTAGAATATAGGAGAAAGGTTACTGTTTATTTTTTTTCAT
GTATTCATTCATTCTACAAATATATTCGGGTGCCAATAGGTACTTGGTATAAGGTTTTTGGCCCCAGAGACATGGGAAAAAAATGCATGCCTTCCCAGAG
AATGCCTAATACTTTCCTTTTGGCTTGTTTTCTTGTTAGGGGCATGGCTTAGTCCCTAAATAACATTGTGTGGTTTAATTCCTACTCCGTATCTCTTCTACC
ACTCTGGCCACTACGATAAGCAGGTAGCTGGGTTTTGTAGTGAGCTTGCTCCTTAAGTTACAGGAACTCTCCTTATAATAGACACTTCATTTTCCTAGTC
CATCCCTCATGAAAAATGACTGACCACTGCTGGGCAGCAGGAGGGATGATGACCAACTAATTCCCAAACCCCAGTCTCATTGGTACCAGCCTTGGGGA
ACCACCTACACTTGAGCCACAATTGGTTTTGAAGTGCATTTACAAGGTTTGTCTATTTTCAGTTCTTTACTTTTTACATGCTGACACATACATACACTGCC
TAAATAGATCTCTTTCAGAAACAATCCTCAGATAACGCATAGCAAAATGGAGATGGAGACATGATTTCTCATGCAACAGCTTCTCTAATTATACCTTAGA
AATGTTCTCCTTTTTATCATCAAATCTGCTCAAGAAGGGCTTTTTATAGTAGAATAATATCAGTGGATGAAAACAGCTTAACATTTTACCATGCTTAAGTT

TTAAGAATAAAATAAAAATTGGAAATAATTGGCCAAAATTGAAAGGAAAAATTTTTTTAAAATTTCTCTAAATGTAGGCCTGGCTGGGCTTTGACCTTTT
CCGTTTTTAAATCACTCACAGAGGGTGGGACAGGAGGAAGAGTGAAGGAAAAGGTCAAACCTGTTTTAAGGGCAACCTGCCTTTGTTCTGAATTGGT
CTTAAGAACATTACCAGCTCCAGGTTTAAATTGTTCAGTTTCATGCAGTTCCAATAGCTGATCATTGTTGAGATGAGGACAAAATCCTTTGTCCTCACTA
GTTTGCTTTACATTTTTGAAAAGTATTATTTTTGTCCAAGTGCTTATCAACTAAACCTTGTGTTAGGTAAGAATGGAATTTATTAAGTGAATCAGTGTGAC
CCTTCTTGTCATAAGATTATCTTAAAGCTGAAGCCAAAATATGCTTCAAAAGAAGAGGACTTTATTGTTCATTGTAGTTCATACATTCAAAGCATCTGAA
CTGTAGTTTCTATAGCAAGCCAATTACATCCATAAGTGGAGAAGGAAATAGATAAATGTCAAAGTATGATTGGTGGAGGGAGCAAGGTTGAAGATAATC
TGGGGTTGAAATTTTCTAGTTTTCATTCTGTACATTTTTAGTTAGACATCAGATTTGAAATATTAATGTTTACCTTTCAATGTGTGGTATCAGCTGGACTCA
GTAACACCCCTTTCTTCAGCTGGGGATGGGGAATGGATTATTGGAAAATGGAAAGAAGAAAGTAACTAAAAGCCTTCCTTTCACAGTTTCTGGCATCA
CTACCACTACTGATTAAACAAGAATAAGAGAACATTTTATCATCATCTGCTTTATTCACATAAATGAAGTTGTGATGAATAAATCTGCTTTTATGCAGACA
CAAGGAATTAAGTGGCTTCGTCATTGTCCTTCTACCTCAAAGATAATTTATTCCAAAAGCTAAGATAAATGGAAGACTCTTGAACTTGTGAACTGATGTG
AAATGCAGAATCTCTTTTGAGTCTTTGCTGTTTGGAAGATTGAAAAATATTGTTCAGCATGGGTGACCACCAGAAAGTAATCTTAAGCCATCTAGATGTC
ACAATTGAAACAAACTGGGGAGTTGGTTGCTATTGTAAAATAAAATATACTGTTTTGAAAA
>ENST00000635841.1|ENSG00000229807.10|OTTHUMG00000021839.5|OTTHUMT00000488978.1|RP13-216E22.1-011|XIST|1734|
GGACATGGTGGTGAGCCGTGGCAAGGACCAGAATGGATCACAGATGATCGTTGGCCAACAGGTGGCAGAAGAGGAATTCCTGCCTTCCTCAAGAGGA
ACACCTACCCCTTGGCTAATGCTGGGATGCCACCTATAGAAAAGTCAGAGGGTCCAGATCCCATTGAAGATACCACGCTGCATGTGTCCTTAGTAGTCA
TGTCTCCTTAGGCTCCTCTTGGACATTCTGAGCATGTGAGACCTGAGGACTGCAAACAGCTATAAGAGGCTCCAAATTAATCATATCTTTCCCTTTGAGA
ATCTGGCCAAGCTCCAGCTAATCTACTTGGATGGGTTGCCAGCTATCTGGAGAAAAAGATCTTCCTCAGAAGAATAGGCTTGTTGTTTTACAGTGTTAG
TGATCCATTCCCTTTGACGATCCCTAGGTGGAGATGGGGCATGAGGATCCTCCAGGGGAAAAGCTCACTACCACTGGGCAACAACCCTAGGTCAGGAG
GTTCTGTCAAGATACTTTCCTGGTCCCAGATAGGAAGATAAAGTCTCAAAAACAACCACCACACGTCAAGCTCTTCATTGTTCCTATCTGCCAAATCATT
ATACTTCCTACAAGCAGTGCAGAGAGCTGAGTCTTCAGCAGGTCCAAGAAATTTGAACACACTGAAGGAAGTCAGCCTTCCCACCTGAAGATCAACA
TGCCTGGCACTCTAGCACTTGAGGATAGCTGAATGAATGTGTATTTCTTTGTCTCTTTCTTTCTTGTCTTTGCTCTTTGTTCTCTATCTAAAGTGTGTCTTA
CCCATTTCCATGTTTCTCTTGCTAATTTCTTTCGTGTGTGCCTTTGCCTCATTTTCTCTTTTTGTTCACAAGAGTGGTCTGTGTCTTGTCTTAGACATATCT
CTCATTTTTCATTTTGTTGCTATTTCTCTTTGCTCTCCTAGATGTGGCTCTTCTTTCACGCTTTATTTCATGTCTCCTTTTTGGGTCACATGCTGTGTGCTTT
TTGTCCTTTTCTTGTTCTGTCTACCTCTCCTTTCTCTGCCTACCTCTCTTTTCTCTTTGTGAACTGTGATTATTTGTTACCCCTTCCCCTTCTCGTTCGTTTT
AAATTTCACCTTTTTTCTGAGTCTGGCCTCCTTTCTGCTGTTTCTACTTTTTATCTCACATTTCTCATTTCTGCATTTCCTTTCTGCCTCTCTTGGGCTATTC
TCTCTCTCCTCCCCTGCGTGCCTCAGCATCTCTTGCTGTTTGTGATTTTCTATTTCAGTATTAATCTCTGTTGGCTTGTATTTGTTCTCTGCTTCTTCCCTTT

CTACTCACCTTTGAGTATTTCAGCCTCTTCATGAATCTATCTCCCTCTCTTTGATTTCATGTAATCTCTCCTTAAATATTTCTTTGCATATGTGGGCAAGTGT
ACGTGTGTGTGTGTCATGTGTGGCAGAGGGGCTTCCTAACCCCTGCCTGATAGGTGCAGAACGTCGGCTATCAGAGCAAGCATTGTGGAGCGGTTCCT
TATGCCAGGCTGCCATGTGAGATGATCCAAGACCAAAACAAGGCCCTAGACTGCAGTAAAACCCAGAACTCAAGTAGGGCAGAAGGTGGAAGGCTCA
TATGGATAGAAGGCCCAAAGTATAAGACAGATGGTTTGAGACTTGAGACCCGAGGACTAAGATGGAAAGCCCATGTTCCAAGATAGATAGAAGCCTCA
GGCCTGAAACCAACAAAAGCCTCAAGAGCCAA
>ENST00000433732.1|ENSG00000229807.10|OTTHUMG00000021839.5|OTTHUMT00000083630.1|RP13-216E22.1-007|XIST|676|
GGTGTCTCTAGTGACTCATCAAGTGATATTTGGCAAGACATTTTCCCATTTATGCCAGTTTCCTATTCTGTTGAATGAGGAAATTTTCTCTCTAAAGACCT
AAAAGTTTTGACTTTATAGGTTTCAAAGTTCTGTGGAAACATTTTCTATTGCTTATTAATTTGAATCTTATGTAACTCTAGCACAGTACTCAATATTTATGG
CATTTACATGGTTTATCTCATGTTTTTTTATAGCTCTTCATTGTTCCTATCTGCCAAATCATTATACTTCCTACAAGCAGTGCAGAGAGCTGAGTCTTCAGC
AGGTCCAAGAAATTTGAACACACTGAAGGAAGTCAGCCTTCCCACCTGAAGATCAACATGCCTGGCACTCTAGCACTTGAGGATAGCTGAATGAATGT
GTATTTCTTTGTCTCTTTCTTTCTTGTCTTTGCTCTTTGTTCTCTATCTAAAGTGTGTCTTACCCATTTCCATGTTTCTCTTGCTAATTTCTTTCGTGTGTGC
CTTTGCCTCATTTTCTCTTTTTGTTCACAAGAGTGGTCTGTGTCTTGTCTTAGACATATCTCTCATTTTTCATTTTGTTGCTATTTCTCTTTGCTCTCCTAGA
TGTGGCTCTTCTTTCACGCTTTATTTCATGTCTCCTTTTTGGGTCACATGCTGTGTGCTTTTTGTCC
>ENST00000602587.5|ENSG00000229807.10|OTTHUMG00000021839.5|OTTHUMT00000467479.1|RP13-216E22.1-010|XIST|583|
CCACTCTTCAATCCACATGAAGAAAAGGATCTTCCTCAGAAGAATAGGCTTGTTGTTTTACAGTGTTAGTGATCCATTCCCTTTGACGATCCCTAGGTGG
AGATGGGGCATGAGGATCCTCCAGGGGAAAAGCTCACTACCACTGGGCAACAACCCTAGGTCAGGAGGTTCTGTCAAGATACTTTCCTGGTCCCAGAT
AGGAAGATAAAGTCTCAAAAACAACCACCACACGTCAAGCTCTTCATTGTTCCTATCTGCCAAATCATTATACTTCCTACAAGCAGTGCAGAGAGCTGA
GTCTTCAGCAGGTCCAAGAAATTTGAACACACTGAAGGAAGTCAGCCTTCCCACCTGAAGATCAACATGCCTGGCACTCTAGCACTTGAGGATAGCTG
AATGAATGTGTCTTACCCATTTCCATGTTTCTCTTGCTAATTTCTTTCGTGTGTGCCTTTGCCTCATTTTCTCTTTTTGTTCACAAGAGTGGTCTGTGTCTT
GTCTTAGACATATCTCTCATTTTTCATTTTGTTGCTATTTCTCTTTGCTCTCCTAGATGTGGCTCTTCTTTCACGCTTTATTTCAT
>ENST00000445814.1|ENSG00000229807.10|OTTHUMG00000021839.5|OTTHUMT00000057242.1|RP13-216E22.1-004|XIST|437|
CTAGGTGGAGATGGGGCATGAGGATCCTCCAGGGGAAAAGCTCACTACCACTGGGCAACAACCCTAGGTCAGGAGGTTCTGTCAAGATACTTTCCTG
GTCCCAGATAGGAAGATAAAGTCTCAAAAACAACCACCACACGTCAAGCTCTTCATTGTTCCTATCTGCCAAATCATTATACTTCCTACAAGCAGTGCA
GAGAGCTGAGTCTTCAGCAGGTCCAAGAAATTTGAACACACTGAAGGAAGTCAGCCTTCCCACCTGAAGATCAACATGCCTGGCACTCTAGCACTTG
AGGATAGCTGAATGAATGTGTCTTACCCATTTCCATGTTTCTCTTGCTAATTTCTTTCGTGTGTGCCTTTGCCTCATTTTCTCTTTTTGTTCACAAGAGTG
GTCTGTGTCTTGTCTTAGACATATCTCTCATTTTTCATTTTGT

>ENST00000602863.1|ENSG00000229807.10|OTTHUMG00000021839.5|OTTHUMT00000467480.1|RP13-216E22.1-008|XIST|775|
AAATTAAATATTCTTTTGGATATAAATTTCAACAGTGAGATAGCTGCCTAGTGGAAATGAATAATATCCCAGCCACTAGTGTACAGGGTGTTTTGTGGCA
CAGGATTATGTAATATGGAACTGCTCAAGCAAATAACTAGTCATCACAACAGCAGTTCTTTGTAATAACTGAAAAAGAATATTGTTTCTCGGAGAAGGA
TGTCAAAAGATCGGCCCAGCTCAGGGAGCAGTTTGCCCTACTAGCTCCTCGGACAGCTGTAAAGAAGAGTCTCTGGCTCTTTAGAATACTGATCCCATT
GAAGATACCACGCTGCATGTGTCCTTAGTAGTCATGTCTCCTTAGGCTCCTCTTGGACATTCTGAGCATGTGAGACCTGAGGACTGCAAACAGCTATAA
GAGGCTCCAAATTAATCATATCTTTCCCTTTGAGAATCTGGCCAAGCTCCAGCTAATCTACTTGGATGGGTTGCCAGCTATCTGGAGAAAAAGGTGGAG
ATGGGGCATGAGGATCCTCCAGGGGAAAAGCTCACTACCACTGGGCAACAACCCTAGGTCAGGAGGTTCTGTCAAGATACTTTCCTGGTCCCAGATAG
GAAGATAAAGTCTCAAAAACAACCACCACACGTCAAGCTCTTCATTGTTCCTATCTGCCAAATCATTATACTTCCTACAAGCAGTGCAGAGAGCTGAGT
CTTCAGCAGGTCCAAGAAATTTGAACACACTGAAGGAAGTCAGCCTTCCCACCTGAAGATCAACATGCCTGGCACTCTAGCA
>ENST00000602495.1|ENSG00000229807.10|OTTHUMG00000021839.5|OTTHUMT00000467481.1|RP13-216E22.1-009|XIST|827|
AGATAGCCAATTATTAAAAACAGTCAAGACAATTGCACCTCTAAGCAGTAGTAGCAGTTGCCACACCACCTTGAATCTTGAAGTATTTCAGCAACAGG
ATGACCATTAGCCACAAATTTAGTGTCAGCCCTTAAGGTCGGTATTGGTTTGACCCATATTTTCATGTAGTTCTTTTTCTTCACTTGTCTAATCTTCCCGTG
TACTGCCAGGGCTTGTCATTAGAGGACTTTAGGGAGACCAAGCAGGCTAGAAAGTAGAGACAGGAGATACCTATGTCTAATGCTTCAGTTTATACTTCC
TAGGTTTTTTTCATTGGGGTTTTTGTAACTCTTTTGGTATCCTACCGGTGCTTTGGTAGCCTACTGAACCCTGTCTTTCTTCTTAAGGACATTCTGAGCAT
GTGAGACCTGAGGACTGCAAACAGCTATAAGAGGCTCCAAATTAATCATATCTTTCCCTTTGAGAATCTGGCCAAGCTCCAGCTAATCTACTTGGATGG
GTTGCCAGCTATCTGGAGAAAAAGATCTTCCTCAGAAGAATAGGCTTGTTGTTTTACAGTGTTAGTGATCCATTCCCTTTGACGATCCCTAGGTGGAGAT
GGGGCATGAGGATCCTCCAGGGGAAAAGCTCACTACCACTGGGCAACAACCCTAGGTCAGGAGGTTCTGTCAAGATACTTTCCTGGTCCCAGATAGG
AAGATAAAGTCTCAAAAACAACCACCACACGTCAAGCTCTTCATTGTTCCTATCTGCCAAATCATTATACTTCCTACAAGCAGTGCAGAGAGCTGAGTC
TTCAGCAGGTCCAAGAAATTTGAACACACTG
>HCG18
>ENST00000602591.1|ENSG00000231074.8|OTTHUMG00000031138.4|OTTHUMT00000468040.1|XXbac-BPG283O16.6-013|HCG18|1976|
CACAGTTCATGGTAAAGCCCAAGACTGTACCTGCCCATCCACTGCCTTTTCCATGTATCCTGGAACTGAGCATAGACCTCTTCCCAGGCAGAGCTGACA
GCAAGTAAAGGAGATCATAATCAGGGGACCAAACAACTTTGTCTAAAGTGTGAATGTCACCTAAGGAGAAGCTGTGAGATCAGAAGGGTGGGGCAGA
GGAGCAGACACCATGAGGGAGAGTCCTTGGGGGTACATCTGCCAGACTGACACTGTCTGGCCTGGGCAGTGGAGGGGCTAGCAGGAACCACAGGTA
CTGGTGGTGTGGCTACTACCGTTACAACTGCCTGTGCTTGGACATGGACCCTCTGCAATATGCGGCAGTTTCATTCATTGCCCCCTACATTCTACACCAA
GTAGAAATGGAAGGCAATTGGATACTTCACAGACAAGATCTAAGTGGAGAAGGAATGCGTCCTGTGGCTGCAGAGATCCTTGGAGCTTGGAGGGGAG

AGCTTGAGCCCCACTGATGATGACCTCCCACAGCTCGCCAACTCAGCCCTCCCTAAGTCCCCATCGGGGGCCAATTCTCACTCTGGGGTTGGGGGGAC
TCCACCATAGCTCATCCATCATAGGGATGTTGGTATCTACTGTGGGTTGGGTAGGGCCGATGTGCTGAGGATGGCTCCCCCACAAGCAAGAGATGTGGA
TTTGGGGAGCTTCCCATCTTGTGTTGAAGGAACATAACTCAGAATAATAAGAGCCAACTATAACAAACCCACAGCCAACATCATACTGAATGGGCAAA
AGCTGCAGGCATTCCCCTTGAAAAGTGGCACAGGATAAGGAAGCCCTTTCTCACCACTCCTATTCAATATAGTGTTGAAAGTCCTGATCACAGCAGTCA
GGCAACAGAAATAATAAAGGGCATCCAAATAGGAAGAGAGGAAGTCAAACTATCCTTGTTTGCAGACAGTATGATTCTATATCTAGAAAACCCCATAGC
CTCAGCCCAAAAGGTCCTTCATCTGATAATTTCAGCAAAGTTTCAGGAGACAAAATCAGTGTACAAAAATCACTAACATTCCTATACACAGTCGCCAAG
CCAAGAGCCAAATCAGGAGCACAATCCCATTCATAATTGCCACAAAAAAGAATAAAATACCCAGGAATGCAGCTAACCAAGGAAGTAAAGGATCTCTA
CAATGAGAATCACAAACGCTGCTCAAAGAAATCAGAGATGACACAAACAAATGGAAAAACTTTCCATGCTCATGGCTAGGAAGAATCAATATCATTAA
AGTGGCCATACTACCCAAAGCAATTTATAGATTCAATGCTATTCCTATCAAACTATCAATTACATTTTTCACAGAACTAGAAAAAACTTAAAATTCATATG
GAACCAAAAAGCCTGAGTAGTCAAGGCAATCCTAAACAAAAAGAACAAAGGTGGAGGCATCACATTACCTAACTTCAAACTACAGGGCTACCATGAC
CAAAACAGCATGGTACTGGTACAAAAGCAGACACACAGACCAGTGGGACAGAATAGAGAGCCCAAAAATAAGGCCACAAACCTACAGCCATCTGAT
CTTCAACAAAGTTGACAAAAACAAGCAATGGGGAAAGGTCTCCCTATTCAATAAATGGTGCTGGGATAACTGGCTGGCCATATGCAGAAGATCGTAAC
TGGACCCCTTTTACTATGTACAAAAATTAAGATGGTTTAAAGAGTTAAAACCCAAAATTATAAAAATCCTGAAGATAACTTAGGCAATACCATTCTGGAC
ATAGGAACTGGCAAATATTTCATGATGAAGACACCAAAAGCAATTGTAACAAAAGCAAAAATGGACAAATTGGATTTAAGAGCTTTTTCATAGCAAAAT
AAACAACAGGGCAGACAATCTACCAAATGACAGAAAATTTTTGCAAACTATGCATCTGACAAGGGGCTAATATCTAGCATCTATAAGGAATTTAAACAC
AGTTACAGG
>ENST00000602550.5|ENSG00000231074.8|OTTHUMG00000031138.4|OTTHUMT00000468041.1|XXbac-BPG283O16.6-012|HCG18|559|
ACGTCACTTCCGACTAGTGGAGTTTTTAGCCTTTTAACATTCAAGGATTAGGTAAGTTCTCCCCGCGGTAGACTTCAAATACTCTTTGTCCTGCCCCTGC
TTGGCGAAATTTACCGAAATTATCTACCTATCCCGCTGGAGTGGGAGCCGGGGGAGAGCTGGAACCGCGTCTCTGCTGCTCACCTTTGTGTCCTTAGCT
CCTTCGCATGGGGTCCGACCGCAACACGTGCGCTGGATAAACGCTTGTTAACTGGAGCTCCCTCTTTGCCCACTAGCAGGGCATTAGCTGGTGCTGAA
GACAGTGGCTGCTTGGCGAGCCTGGATCTCCAAGTGACCCCCTCAGCAACTCCTGATGAACAGGGACTTGCACTTAGGAATCTGTTACAGTGAGCACA
GAAGCAGACCTGTGGGCTCATAGTCTTCACCCCCCACCCATATATACAAGATGAACAGAGTGGAGGTTAAACAACTTGACTAAACAACACAGTTCATG
GTAAAGCCCAAGACTGTACCTGCCCATCCACTGCCTTTTCCATGTATCCTGGAACTGAGCATAGAC
>ENST00000438412.5|ENSG00000231074.8|OTTHUMG00000031138.4|OTTHUMT00000256034.1|XXbac-BPG283O16.6-003|HCG18|2440|
TGGAGTTTTTAGCCTTTTAACATTCAAGGATTAGAGCTCCCTCTTTGCCCACTAGCAGGGCATTAGCTGGTGCTGAAGACAGTGGCTGCTTGGCGAGCC
TGGATCTCCAAGTGACCCCCTCAGCAACTCCTGATGAACAGGACTGAAGCCAATATTAAAGCAAGTCAACCAAAGGTTCTCTGGTGTAGACAAGACA

GCAAAAGGACAGACTACCTTGTGGAACCTAGCATTGTTCTCCTTCTGCAGCACTAAGTAACATTTGTTCTCCGTTAAGATCTTTGCAAACCACACACAA
GAATTGCTGGTCATCCTGCCAATAGATGCTGCTCACAGAACCAAATTTCCTGTGCTGAATTGTCACTCATGGGCTTGAGAGTAGGAGACTGGAGACCA
AGGTGGCTAGAATCCAGTTGGGCCTGATGTCTCCCTGTTGAAAGGGCTCCTTGTGGAATGAATAGCACATGGCTCCTGTGGTGGATCTGATAGTGGCAT
AGCACCAAGTGATGCAGGCCTGCCAGGGGCCACAGACACAGAAGATGCTCCCGGGGTCCCCCATGTACTCCAGACACACTGCAGGCCACCTCTCCCA
GCAGGTTGCCAGTCATGGGCCCCATCATCATGACTTCTGTCCAAGGTACTGTGTGCAGAAATGTGATTGAGATTCAAGTCAGGGCCTCTCTGCCCTTTT
CCCTCCAGAAACAAAACCAAGATAATTTATCCTGAACACGGTGAAAAAAGGAAGGGAGGGAGGAGAAAAAGTCCGGGTCTCACCTGGGATTCTCTGT
CTCCTGCAACATGAAGGATTTAGCCTGGGAGGAGGTGGTGAGAACTCTGGGAGAGAAAAAAGAAGGAAAGAATAGTTTTACCCATGCTGAAGTTAAT
TTAAACCTTCACCTAGAGAAGCAAAAAAAAAAAAACCCACACTTTCCCATTTTGTGCCTCCCTTCCTAGAGTTTTAGCCAAAGGTTTAGCTAAGTAATTG
GTTTTACCAGCGCACTCACTCCTCCTATCCCAAGTCTGTTTGACTCCCTCCCCATCATCCTCCTCACCTCTTTTCAGGCAGGGTGGGGATAGCAGCAGG
AGGAGATTTTGGGAGCCTGGCAACTCCTGCAAGGACCGCAGGACAGCCCCTCTGTGGGGATGCGTGGTGCCCCATCTGCCGCCCTTCTGAAGAATGC
ACTGCCTTCACTTTTTACTGTGTTAGAGTCCATCCAGACTGTTCTATCCAAAAAAGTTTCTTTTTCCCCCACAGGCAATCAGGAAATGATTCCTTTCCCG
ACTGCTTCTGTCTAGTGCCTGGGAATCTTGAGTCAATCCCTCAGTAAGTCAGTGACTAGGGAAATCCCTCTCTGAGCCTCCCAGTTCATGTTGCTTAGG
GAACCTGATATTTTCGTGAAACCTGCCTACACATGGGCAGCCCAACAGCAGAACAAATGGTGGTGACCAAAGTGAACAAAGAAGTATAGTTGTGCCA
GCTTCGTAGTTGCCCATGTGGACAAGTCAGCAGGATCAGGACACGAGGAAGAGTAAATGTGAGACAGTCAATGTGACTTCTGCGATAAACAGATTTTT
AAACCCCGAAATTTTGCAAAATTTTGGTGAAACCTGAACTTTCTTCGTTGCATATACTGGCACTATCTGTACCATCATACAACTGTCTCACATTAAAGCT
ATTTTTCTTGGGCACTGATGAGTAAGGTTGGTATAAGTTCCTCAGATCAACAAAAACCCATTTTCCTGTAAGTCTTACATTTAGTATTTAAGGAACTAAA
ACTTAAATACATTTTGTGAAATGGTTGACACTTCACTGATAATGATTTATTGCTTGGATTAATAAATTTTCCAAAAGTTGTCTTATGTAGAATATGGTTTGC
AACCAGCAGAACCATTAATCTATACTGCAATGATATGCACTATGTATAATTGTTTAAAAGCCTCTACTTAATGATGTAAAATGCTCTATTTAATTACACATT
TGGGTAAACTGTATACTAACATCTGATGGCATTTTTCCACTGTTTGTTGCTTTTTTCAAATACTTTATTGTACAAAGCTGTTCTTAATATTTTTCAAGTTTT
TTTCTTTGAATTTTGCTAATGTTTTCCTTGAATTATGAGCACTGACAGAATGTGCTTAGCACTTTTGGCTATTCACACAGCTTTTGAGCATGATTTGCATC
CAATATTTACATTGCTAGCAATAATAAGCCATCTGTGAGTTTTGTCAAAAGTTATTGGGGATTTTTTAAATTTTAGAAATGCAAATTATTGTTTCTTTGAAC
TAACTCTTATGCAGTTGCAAAGGCATTTCCAGTTGTTATAGTTTGTGTACAATATCAGGTGTTCCAGATTATGATTCATTATTAATATCATTGCTTCCTTGTT
CCCTCTGAGGTCCAGAAGATTCATGTTTACAATATTTAGAAACAATGTTAAAAAGGT
>ENST00000444126.5|ENSG00000231074.8|OTTHUMG00000031138.4|OTTHUMT00000256039.1|XXbac-BPG283O16.6-008|HCG18|4605|
GACTTCAAATACTCTTTGTCCTGCCCCTGCTTGGCGAAATTTACCGAAATTATCTACCTATCCCGCTGGAGTGGGAGCCGGGGGAGAGCTGGAACCGCG
TCTCTGCTGCTCACCTTTGTGTCCTTAGCTCCTTCGCATGGGGTCCGACCGCAACACGTGCGCTGGATAAACGCTTGTTAACTGGGTGAGAGCCAAGG

CCTGTTCTTTCCACTTCTGGGGCTGTGACTTGAGTTTCTCCGCAGGAGCAGATAGTGTTTGTGTAGGGACTGCGGACTTTTGCATTACAGCCTCCCTGC
TTTGCCCGCCTGCTGCTGGAAGCTTAGCAGGTAAAAGTCTTGCAGTGAAAAACCCGAGGACCCTTACCGCAAGTGTCTTTTGCTCCCAGCTACTGATA
CTGGATTCCACTCGTGATTCTCCCTTTCTTAGCGCATTCATGATATAGACATCAGTCTCTGAGCTGGAGGAGGACAAAGGCAGCGGTCCTGTGAATTCTA
TGCTCTAGCTTGGGTTAAGGGATTTGGAATTGCACTTGTTTCAGAGAGCTCCCTCTTTGCCCACTAGCAGGGCATTAGCTGGTGCTGAAGACAGTGGCT
GCTTGGCGAGCCTGGATCTCCAAGTGACCCCCTCAGCAACTCCTGATGAACAGGACTGAAGCCAATATTAAAGCAAGTCAACCAAAGGTTCTCTGGTG
TAGACAAGACAGCAAAAGGACAGACTACCTTGTGGAACCTAGCATTGTTCTCCTTCTGCAGCACTAAGTAACATTTGTTCTCCGTTAAGATCTTTGCAA
ACCACACACAAGAATTGCTGGTCATCCTGCCAATAGATGCTGCTCACAGAACCAAATTTCCTGTGCTGAATTGTCACTCATGGGCTTGAGAGTAGGAG
ACTGGAGACCAAGGTGGCTAGAATCCAGTTGGGCCTGATGTCTCCCTGTTGAAAGGGCTCCTTGTGGAATGAATAGCACATGGCTCCTGTGGTGGATC
TGATAGTGGCATAGCACCAAGTGATGCAGGCCTGCCAGGGGCCACAGACACAGAAGATGCTCCCGGGGTCCCCCATGTACTCCAGACACACTGCAGG
CCACCTCTCCCAGCAGGTTGCCAGTCATGGGCCCCATCATCATGACTTCTGTCCAAGGTGTGCTCGGAAATCTCTTCCTTAACTGTGACTTTCTGACAG
GTGGAGGATGTGGTCAGGAGTGGGAAAAGGATTCGAGACGGGAAGAGGGAGGGGTTCAGGATGAAGAGAGATAATCTGTGCCACAGATGCTGGCCC
TGCAGTTAGCTCCCACCTAGTCCGTGCACACACATTCTAATCCCCTCCCATTCCTTTACATGCTGCGGCCAGAGAGGCCTTTCTCAAAGTGGAAGTCTC
ATCCTCACTTCTCTGGTTACAGTGCTGGGCCATGGTAACTTACAAGGCTTAGCAGGAACTGTCTGCGCACTCCCCCTTCCTGCCCACTACCTTGTTTCCC
TCCAGTTGCGAGAGAAAACATTGATTGAGCATTAACTATGTGCCAGGCTTGTCCTAAGTCCTTTGCATGTATTCACTCAAACAATCCTCACAACATTCCT
ATCACATCTCCCATTTCACAGTGAGGGTTCTAAAGCACGTAGTGGGTGAGGAACTTGTCCAGGGTCACACAACTAAGTGGGGGTGGAGAAACATCAA
ATCTAGGTGGTCCAGGTGGTGGCCAGAACCCATCAGCACCTCACTACAGCTGCCTCCAGTTTCTCGCTCTAGGAAATACTCTTCCCTGTCAACTCCTGT
TGTCCTCTGGGCTCAGTTGACATGTCATCTCCTTGAGGAGGACATCCTGAGATGCTCCCCGACTAGGGTGGGCACCCACTTCCACACTCCAAATGCCTA
TTTGACCTGTGTATCGCTGCATCCCCACTGCCTGGCAGATAGCAGGCCCCTAATAAATATGATTTGAGCAAATAAATATAGTTCTTCAAAAAATAGGGAG
GACTTCTTGCTTCAGGTAATGGTAGGCTAGGACATTTGGACCAACCCTCCTGCAGAAAATAGCCATTTATTTTGATGCAATATATCTGGCTATGACTAGA
GATTTCAAATATTTGGAGGGCTATTATGTGAAGAACTAAACTTACTCTTTGTGACATAGAGGTCAGAAATTGGTCGTTTCCATAGAGTATGAGAGAGACA
GAATTGTGCTCATCATAAGTATCTTTTTTAAAAGTTAGACTTAATAAAATGGGCTGCCTTGGTAGGTGGTGAGTTCTCTGTCACGAAAGAGATTCAGCCA
CTTGCTCTGAGAAAGCATGGAGAATATCAACATTGTAAGGGTCGACCACCTCATCTCTGAGGCCCCCTCCTCACTTAGGTTGCCATTGCAGGTGGAACA
CTGGGCTGAGTGTCAAAGAACCTGGGTTCCACAAGCTGTGCGACCATGGCTGGTTGTTTTGGATCTCATTCCTAATATCTGTTAGTTAAGCTGACCCGT
CAATACTGACCCACTTCACAGAGTTATGAAGGGATTAAATTAGGTATTACATGTAAATCTGTTTTGTTTGCTTTTTGTTGTTCTTTTTTGTTTTGCTTTTTT
CTTTGCATTATGAAATCCCAAGTCAACTTTTTCCATTCTTTTATTTTTTTAAAAATTGACATCATCATTATAACCACAAAATAATTTTTAAAATGGAAAAAA
AAAACTCACCTGCAACCCCACAATCTAATACAATAATCATACTTTTTTCCCTTTATTCTCCTTTATTGTGACCAAGGATGACTTTGGGCATTGCTGTGAGG

AACTATTTCGTTTAACACCCTACTACCTGAACATGATGCGTACTCAGCCTTTCACCACCCCCCAAGTAGCTACACATGATGTGATAATAGTATTGGTGGT
GGTTTTAGAGAGTTGGAGAGAAAACTGAAGTTGGATTTGGGTAAAAGAAAAAAGAGTGGGAATTATTTTTTCTCCATTCTAAAGCCCATAAAGCAATG
TTAATTGTTCCCACCCCTCTGCTATTCTGTCTCCTCAGGTACTGTGTGCAGAAATGTGATTGAGATTCAAGTCAGGGCCTCTCTGCCCTTTTCCCTCCAG
AAACAAAACCAAGATAATTTATCCTGAACACGGTGAAAAAAGGAAGGGAGGGAGGAGAAAAAGTCCGGGTCTCACCTGGGATTCTCTGTCTCCTGCA
ACATGAAGGATTTAGCCTGGGAGGAGGTGGTGAGAACTCTGGGAGAGAAAAAAGAAGGAAAGAATAGTTTTACCCATGCTGAAGTTAATTTAAACCT
TCACCTAGAGAAGCAAAAAAAAAAAAACCCACACTTTCCCATTTTGTGCCTCCCTTCCTAGAGTTTTAGCCAAAGGTTTAGCTAAGTAATTGGTTTTACC
AGCGCACTCACTCCTCCTATCCCAAGTCTGTTTGACTCCCTCCCCATCATCCTCCTCACCTCTTTTCAGGCAGGGTGGGGATAGCAGCAGGAGGAGATT
TTGGGAGCCTGGCAACTCCTGCAAGGACCGCAGGACAGCCCCTCTGTGGGGATGCGTGGTGCCCCATCTGCCGCCCTTCTGAAGAATGCACTGCCTTC
ACTTTTTACTGTGTTAGAGTCCATCCAGACTGTTCTATCCAAAAAAGTTTCTTTTTCCCCCACAGGCAATCAGGAAATGATTCCTTTCCCGACTGCTTCT
GTCTAGTGCCTGGGAATCTTGAGTCAATCCCTCAGTAAGTCAGTGACTAGGGAAATCCCTCTCTGAGCCTCCCAGTTCATGTTGCTTAGGGAACCTGAT
ATTTTCGTGAAACCTGCCTACACATGGGCAGCCCAACAGCAGAACAAATGGTGGTGACCAAAGTGAACAAAGAAGTATAGTTGTGCCAGCTTCGTAG
TTGCCCATGTGGACAAGTCAGCAGGATCAGGACACGAGGAAGAGTAAATGTGAGACAGTCAATGTGACTTCTGCGATAAACAGATTTTTAAACCCCG
AAATTTTGCAAAATTTTGGTGAAACCTGAACTTTCTTCGTTGCATATACTGGCACTATCTGTACCATCATACAACTGTCTCACATTAAAGCTATTTTTCTT
GGGCACTGATGAGTAAGGTTGGTATAAGTTCCTCAGATCAACAAAAACCCATTTTCCTGTAAGTCTTACATTTAGTATTTAAGGAACTAAAACTTAAATA
CATTTTGTGAAATGGTTGACACTTCACTGATAATGATTTATTGCTTGGATTAATAAATTTTCCAAAAGTTGTCTTATGTAGAATATGGTTTGCAACCAGCA
GAACCATTAATCTATACTGCAATGATATGCACTATGTATAATTGTTTAAAAGCCTCTACTTAATGATGTAAAATGCTCTATTTAATTACACATTTGGGTAAA
CTGTATACTAACATCTGATGGCATTTTTCCACTGTTTGTTGCTTTTTTCAAATACTTTATTGTACAAAGCTGTTCTTAATATTTTTCAAGTTTTTTTCTTTGA
ATTTTGCTAATGTTTTCCTTGAATTATGAGCACTGACAGAATGTGCTTAGCACTTTTGGCTATTCACACAGCTTTTGAGCATGATTTGCATCCAATATTTAC
ATTGCTAGCAATAATAAGCCATCTGTGAGTTTTGTCAAAA
>ENST00000449544.5|ENSG00000231074.8|OTTHUMG00000031138.4|OTTHUMT00000256037.1|XXbac-BPG283O16.6-006|HCG18|4262|
TCTCCCCGCGGTAGACTTCAAATACTCTTTGTCCTGCCCCTGCTTGGCGAAATTTACCGAAATTATCTACCTATCCCGCTGGAGTGGGAGCCGGGGGAG
AGCTGGAACCGCGTCTCTGCTGCTCACCTTTGTGTCCTTAGCTCCTTCGCATGGGGTCCGACCGCAACACGTGCGCTGGATAAACGCTTGTTAACTGG
AGCTCCCTCTTTGCCCACTAGCAGGGCATTAGCTGGTGCTGAAGACAGTGGCTGCTTGGCGAGCCTGGATCTCCAAGTGACCCCCTCAGCAACTCCTG
ATGAACAGGACTGAAGCCAATATTAAAGCAAGTCAACCAAAGGTTCTCTGGTGTAGACAAGACAGCAAAAGGACAGACTACCTTGTGGAACCTAGCA
TTGTTCTCCTTCTGCAGCACTAAGTAACATTTGTTCTCCGTTAAGATCTTTGCAAACCACACACAAGAATTGCTGGTCATCCTGCCAATAGATGCTGCTC
ACAGAACCAAATTTCCTGTGCTGAATTGTCACTCATGGGCTTGAGAGTAGGAGACTGGAGACCAAGGTGGCTAGAATCCAGTTGGGCCTGATGTCTCC

CTGTTGAAAGGGCTCCTTGTGGAATGAATAGCACATGGCTCCTGTGGTGGATCTGATAGTGGCATAGCACCAAGTGATGCAGGCCTGCCAGGGGCCAC
AGACACAGAAGATGCTCCCGGGGTCCCCCATGTACTCCAGACACACTGCAGGCCACCTCTCCCAGCAGGTTGCCAGTCATGGGCCCCATCATCATGAC
TTCTGTCCAAGGTGTGCTCGGAAATCTCTTCCTTAACTGTGACTTTCTGACAGGTGGAGGATGTGGTCAGGAGTGGGAAAAGGATTCGAGACGGGAA
GAGGGAGGGGTTCAGGATGAAGAGAGATAATCTGTGCCACAGATGCTGGCCCTGCAGTTAGCTCCCACCTAGTCCGTGCACACACATTCTAATCCCCT
CCCATTCCTTTACATGCTGCGGCCAGAGAGGCCTTTCTCAAAGTGGAAGTCTCATCCTCACTTCTCTGGTTACAGTGCTGGGCCATGGTAACTTACAAG
GCTTAGCAGGAACTGTCTGCGCACTCCCCCTTCCTGCCCACTACCTTGTTTCCCTCCAGTTGCGAGAGAAAACATTGATTGAGCATTAACTATGTGCCA
GGCTTGTCCTAAGTCCTTTGCATGTATTCACTCAAACAATCCTCACAACATTCCTATCACATCTCCCATTTCACAGTGAGGGTTCTAAAGCACGTAGTGG
GTGAGGAACTTGTCCAGGGTCACACAACTAAGTGGGGGTGGAGAAACATCAAATCTAGGTGGTCCAGGTGGTGGCCAGAACCCATCAGCACCTCACT
ACAGCTGCCTCCAGTTTCTCGCTCTAGGAAATACTCTTCCCTGTCAACTCCTGTTGTCCTCTGGGCTCAGTTGACATGTCATCTCCTTGAGGAGGACATC
CTGAGATGCTCCCCGACTAGGGTGGGCACCCACTTCCACACTCCAAATGCCTATTTGACCTGTGTATCGCTGCATCCCCACTGCCTGGCAGATAGCAGG
CCCCTAATAAATATGATTTGAGCAAATAAATATAGTTCTTCAAAAAATAGGGAGGACTTCTTGCTTCAGGTAATGGTAGGCTAGGACATTTGGACCAACC
CTCCTGCAGAAAATAGCCATTTATTTTGATGCAATATATCTGGCTATGACTAGAGATTTCAAATATTTGGAGGGCTATTATGTGAAGAACTAAACTTACTC
TTTGTGACATAGAGGTCAGAAATTGGTCGTTTCCATAGAGTATGAGAGAGACAGAATTGTGCTCATCATAAGTATCTTTTTTAAAAGTTAGACTTAATAA
AATGGGCTGCCTTGGTAGGTGGTGAGTTCTCTGTCACGAAAGAGATTCAGCCACTTGCTCTGAGAAAGCATGGAGAATATCAACATTGTAAGGGTCGA
CCACCTCATCTCTGAGGCCCCCTCCTCACTTAGGTTGCCATTGCAGGTGGAACACTGGGCTGAGTGTCAAAGAACCTGGGTTCCACAAGCTGTGCGAC
CATGGCTGGTTGTTTTGGATCTCATTCCTAATATCTGTTAGTTAAGCTGACCCGTCAATACTGACCCACTTCACAGAGTTATGAAGGGATTAAATTAGGTA
TTACATGTAAATCTGTTTTGTTTGCTTTTTGTTGTTCTTTTTTGTTTTGCTTTTTTCTTTGCATTATGAAATCCCAAGTCAACTTTTTCCATTCTTTTATTTTT
TTAAAAATTGACATCATCATTATAACCACAAAATAATTTTTAAAATGGAAAAAAAAAAACTCACCTGCAACCCCACAATCTAATACAATAATCATACTTTTT
TCCCTTTATTCTCCTTTATTGTGACCAAGGATGACTTTGGGCATTGCTGTGAGGAACTATTTCGTTTAACACCCTACTACCTGAACATGATGCGTACTCAG
CCTTTCACCACCCCCCAAGTAGCTACACATGATGTGATAATAGTATTGGTGGTGGTTTTAGAGAGTTGGAGAGAAAACTGAAGTTGGATTTGGGTAAAA
GAAAAAAGAGTGGGAATTATTTTTTCTCCATTCTAAAGCCCATAAAGCAATGTTAATTGTTCCCACCCCTCTGCTATTCTGTCTCCTCAGGTACTGTGTG
CAGAAATGTGATTGAGATTCAAGTCAGGGCCTCTCTGCCCTTTTCCCTCCAGAAACAAAACCAAGATAATTTATCCTGAACACGGTGAAAAAAGGAAG
GGAGGGAGGAGAAAAAGTCCGGGTCTCACCTGGGATTCTCTGTCTCCTGCAACATGAAGGATTTAGCCTGGGAGGAGGTGGTGAGAACTCTGGGAG
AGAAAAAAGAAGGAAAGAATAGTTTTACCCATGCTGAAGTTAATTTAAACCTTCACCTAGAGAAGCAAAAAAAAAAAACCCACACTTTCCCATTTTGT
GCCTCCCTTCCTAGAGTTTTAGCCAAAGGTTTAGCTAAGTAATTGGTTTTACCAGCGCACTCACTCCTCCTATCCCAAGTCTGTTTGACTCCCTCCCCAT
CATCCTCCTCACCTCTTTTCAGGCAGGGTGGGGATAGCAGCAGGAGGAGATTTTGGGAGCCTGGCAACTCCTGCAAGGACCGCAGGACAGCCCCTCT

GTGGGGATGCGTGGTGCCCCATCTGCCGCCCTTCTGAAGAATGCACTGCCTTCACTTTTTACTGTGTTAGAGTCCATCCAGACTGTTCTATCCAAAAAA
GTTTCTTTTTCCCCCACAGGCAATCAGGAAATGATTCCTTTCCCGACTGCTTCTGTCTAGTGCCTGGGAATCTTGAGTCAATCCCTCAGTAAGTCAGTGA
CTAGGGAAATCCCTCTCTGAGCCTCCCAGTTCATGTTGCTTAGGGAACCTGATATTTTCGTGAAACCTGCCTACACATGGGCAGCCCAACAGCAGAACA
AATGGTGGTGACCAAAGTGAACAAAGAAGTATAGTTGTGCCAGCTTCGTAGTTGCCCATGTGGACAAGTCAGCAGGATCAGGACACGAGGAAGAGTA
AATGTGAGACAGTCAATGTGACTTCTGCGATAAACAGATTTTTAAACCCCGAAATTTTGCAAAATTTTGGTGAAACCTGAACTTTCTTCGTTGCATATAC
TGGCACTATCTGTACCATCATACAACTGTCTCACATTAAAGCTATTTTTCTTGGGCACTGATGAGTAAGGTTGGTATAAGTTCCTCAGATCAACAAAAAC
CCATTTTCCTGTAAGTCTTACATTTAGTATTTAAGGAACTAAAACTTAAATACATTTTGTGAAATGGTTGACACTTCACTGATAATGATTTATTGCTTGGAT
TAATAAATTTTCCAAAAGTTGTCTTATGTAGAATATGGTTTGCAACCAGCAGAACCATTAATCTATACTGCAATGATATGCACTATGTATAATTGTTTAAAA
GCCTCTACTTAATGATGTAAAATGCTCTATTTAATTACACATTTGGGTAAACTGTATACTAACATCTGATGGCATTTTTCCACTGTTTGTTGCTTTTTTCAA
ATACTTTATTGTACAAAGCTGTTCTTAATATTTTTCAAGTTTTTTTCTTTGAATTTTGCTAATGTTTTCCTTGAATTATGAGCACTGACAGAATGTGCTTAG
CACTTTTGGCTATTCACACAGCTTTTGAGCATGATTTGCATCCAATATTTACATTGCTAGCAATAATAAGCCATCTGTGAGTTTTGTCAAAA
>ENST00000454129.5|ENSG00000231074.8|OTTHUMG00000031138.4|OTTHUMT00000256038.1|XXbac-BPG283O16.6-007|HCG18|4433|
GCCTTTTAACATTCAAGGATTAGGTAAGTTCTCCCCGCGGTAGACTTCAAATACTCTTTGTCCTGCCCCTGCTTGGCGAAATTTACCGAAATTATCTACCT
ATCCCGCTGGAGTGGGAGCCGGGGGAGAGCTGGAACCGCGTCTCTGCTGCTCACCTTTGTGTCCTTAGCTCCTTCGCATGGGGTCCGACCGCAACACG
TGCGCTGGATAAACGCTTGTTAACTGGGTGAGAGCCAAGGCCTGTTCTTTCCACTTCTGGGGCTGTGACTTGAGTTTCTCCGCAGGAGCAGATAGTGT
TTGTGTAGGGACTGCGGACTTTTGCATTACAGCCTCCCTGCTTTGCCCGCCTGCTGCTGGAAGCTTAGCAGAGCTCCCTCTTTGCCCACTAGCAGGGCA
TTAGCTGGTGCTGAAGACAGTGGCTGCTTGGCGAGCCTGGATCTCCAAGTGACCCCCTCAGCAACTCCTGATGAACAGGACTGAAGCCAATATTAAAG
CAAGTCAACCAAAGGTTCTCTGGTGTAGACAAGACAGCAAAAGGACAGACTACCTTGTGGAACCTAGCATTGTTCTCCTTCTGCAGCACTAAGTAACA
TTTGTTCTCCGTTAAGATCTTTGCAAACCACACACAAGAATTGCTGGTCATCCTGCCAATAGATGCTGCTCACAGAACCAAATTTCCTGTGCTGAATTGT
CACTCATGGGCTTGAGAGTAGGAGACTGGAGACCAAGGTGGCTAGAATCCAGTTGGGCCTGATGTCTCCCTGTTGAAAGGGCTCCTTGTGGAATGAAT
AGCACATGGCTCCTGTGGTGGATCTGATAGTGGCATAGCACCAAGTGATGCAGGCCTGCCAGGGGCCACAGACACAGAAGATGCTCCCGGGGTCCCC
CATGTACTCCAGACACACTGCAGGCCACCTCTCCCAGCAGGTTGCCAGTCATGGGCCCCATCATCATGACTTCTGTCCAAGGTGTGCTCGGAAATCTCT
TCCTTAACTGTGACTTTCTGACAGGTGGAGGATGTGGTCAGGAGTGGGAAAAGGATTCGAGACGGGAAGAGGGAGGGGTTCAGGATGAAGAGAGAT
AATCTGTGCCACAGATGCTGGCCCTGCAGTTAGCTCCCACCTAGTCCGTGCACACACATTCTAATCCCCTCCCATTCCTTTACATGCTGCGGCCAGAGA
GGCCTTTCTCAAAGTGGAAGTCTCATCCTCACTTCTCTGGTTACAGTGCTGGGCCATGGTAACTTACAAGGCTTAGCAGGAACTGTCTGCGCACTCCCC
CTTCCTGCCCACTACCTTGTTTCCCTCCAGTTGCGAGAGAAAACATTGATTGAGCATTAACTATGTGCCAGGCTTGTCCTAAGTCCTTTGCATGTATTCA

CTCAAACAATCCTCACAACATTCCTATCACATCTCCCATTTCACAGTGAGGGTTCTAAAGCACGTAGTGGGTGAGGAACTTGTCCAGGGTCACACAACT
AAGTGGGGGTGGAGAAACATCAAATCTAGGTGGTCCAGGTGGTGGCCAGAACCCATCAGCACCTCACTACAGCTGCCTCCAGTTTCTCGCTCTAGGA
AATACTCTTCCCTGTCAACTCCTGTTGTCCTCTGGGCTCAGTTGACATGTCATCTCCTTGAGGAGGACATCCTGAGATGCTCCCCGACTAGGGTGGGCA
CCCACTTCCACACTCCAAATGCCTATTTGACCTGTGTATCGCTGCATCCCCACTGCCTGGCAGATAGCAGGCCCCTAATAAATATGATTTGAGCAAATAA
ATATAGTTCTTCAAAAAATAGGGAGGACTTCTTGCTTCAGGTAATGGTAGGCTAGGACATTTGGACCAACCCTCCTGCAGAAAATAGCCATTTATTTTGA
TGCAATATATCTGGCTATGACTAGAGATTTCAAATATTTGGAGGGCTATTATGTGAAGAACTAAACTTACTCTTTGTGACATAGAGGTCAGAAATTGGTC
GTTTCCATAGAGTATGAGAGAGACAGAATTGTGCTCATCATAAGTATCTTTTTTAAAAGTTAGACTTAATAAAATGGGCTGCCTTGGTAGGTGGTGAGTT
CTCTGTCACGAAAGAGATTCAGCCACTTGCTCTGAGAAAGCATGGAGAATATCAACATTGTAAGGGTCGACCACCTCATCTCTGAGGCCCCCTCCTCA
CTTAGGTTGCCATTGCAGGTGGAACACTGGGCTGAGTGTCAAAGAACCTGGGTTCCACAAGCTGTGCGACCATGGCTGGTTGTTTTGGATCTCATTCCT
AATATCTGTTAGTTAAGCTGACCCGTCAATACTGACCCACTTCACAGAGTTATGAAGGGATTAAATTAGGTATTACATGTAAATCTGTTTTGTTTGCTTTT
TGTTGTTCTTTTTTGTTTTGCTTTTTTCTTTGCATTATGAAATCCCAAGTCAACTTTTTCCATTCTTTTATTTTTTTAAAAATTGACATCATCATTATAACCA
CAAAATAATTTTTAAAATGGAAAAAAAAAAACTCACCTGCAACCCCACAATCTAATACAATAATCATACTTTTTTCCCTTTATTCTCCTTTATTGTGACCAA
GGATGACTTTGGGCATTGCTGTGAGGAACTATTTCGTTTAACACCCTACTACCTGAACATGATGCGTACTCAGCCTTTCACCACCCCCCAAGTAGCTAC
ACATGATGTGATAATAGTATTGGTGGTGGTTTTAGAGAGTTGGAGAGAAAACTGAAGTTGGATTTGGGTAAAAGAAAAAAGAGTGGGAATTATTTTTTC
TCCATTCTAAAGCCCATAAAGCAATGTTAATTGTTCCCACCCCTCTGCTATTCTGTCTCCTCAGGTACTGTGTGCAGAAATGTGATTGAGATTCAAGTCA
GGGCCTCTCTGCCCTTTTCCCTCCAGAAACAAAACCAAGATAATTTATCCTGAACACGGTGAAAAAAGGAAGGGAGGGAGGAGAAAAAGTCCGGGTC
TCACCTGGGATTCTCTGTCTCCTGCAACATGAAGGATTTAGCCTGGGAGGAGGTGGTGAGAACTCTGGGAGAGAAAAAAGAAGGAAAGAATAGTTTT
ACCCATGCTGAAGTTAATTTAAACCTTCACCTAGAGAAGCAAAAAAAAAAAAACCCACACTTTCCCATTTTGTGCCTCCCTTCCTAGAGTTTTAGCCAAA
GGTTTAGCTAAGTAATTGGTTTTACCAGCGCACTCACTCCTCCTATCCCAAGTCTGTTTGACTCCCTCCCCATCATCCTCCTCACCTCTTTTCAGGCAGG
GTGGGGATAGCAGCAGGAGGAGATTTTGGGAGCCTGGCAACTCCTGCAAGGACCGCAGGACAGCCCCTCTGTGGGGATGCGTGGTGCCCCATCTGCC
GCCCTTCTGAAGAATGCACTGCCTTCACTTTTTACTGTGTTAGAGTCCATCCAGACTGTTCTATCCAAAAAAGTTTCTTTTTCCCCCACAGGCAATCAGG
AAATGATTCCTTTCCCGACTGCTTCTGTCTAGTGCCTGGGAATCTTGAGTCAATCCCTCAGTAAGTCAGTGACTAGGGAAATCCCTCTCTGAGCCTCCC
AGTTCATGTTGCTTAGGGAACCTGATATTTTCGTGAAACCTGCCTACACATGGGCAGCCCAACAGCAGAACAAATGGTGGTGACCAAAGTGAACAAA
GAAGTATAGTTGTGCCAGCTTCGTAGTTGCCCATGTGGACAAGTCAGCAGGATCAGGACACGAGGAAGAGTAAATGTGAGACAGTCAATGTGACTTCT
GCGATAAACAGATTTTTAAACCCCGAAATTTTGCAAAATTTTGGTGAAACCTGAACTTTCTTCGTTGCATATACTGGCACTATCTGTACCATCATACAAC
TGTCTCACATTAAAGCTATTTTTCTTGGGCACTGATGAGTAAGGTTGGTATAAGTTCCTCAGATCAACAAAAACCCATTTTCCTGTAAGTCTTACATTTAG

TATTTAAGGAACTAAAACTTAAATACATTTTGTGAAATGGTTGACACTTCACTGATAATGATTTATTGCTTGGATTAATAAATTTTCCAAAAGTTGTCTTAT
GTAGAATATGGTTTGCAACCAGCAGAACCATTAATCTATACTGCAATGATATGCACTATGTATAATTGTTTAAAAGCCTCTACTTAATGATGTAAAATGCTC
TATTTAATTACACATTTGGGTAAACTGTATACTAACATCTGATGGCATTTTTCCACTGTTTGTTGCTTTTTTCAAATACTTTATTGTACAAAGCTGTTCTTAA
TATTTTTCAAGTTTTTTTCTTTGAATTTTGCTAATGTTTTCCTTGAATTATGAGCACTGACAGAATGTGCTTAGCACTTTTGGCTATTCACACAGCTTTTGA
GCATGATTTGCATCCAATATTTACATTGCTAGCAATAATAAGCCATCTGTGAGTTTTGTCAAAA
>ENST00000454269.5|ENSG00000231074.8|OTTHUMG00000031138.4|OTTHUMT00000256035.1|XXbac-BPG283O16.6-004|HCG18|2430|
GGAGTTTTTAGCCTTTTAACATTCAAGGATTAGGTAAGTTCTCCCCGCGGTAGACTTCAAATACTCTTTGTCCTGCCCCTGCTTGGCGAAATTTACCGAA
ATTATCTACCTATCCCGCTGGAGTGGGAGCCGGGGGAGAGCTGGAACCGCGTCTCTGCTGCTCACCTTTGTGTCCTTAGCTCCTTCGCATGGGGTCCGA
CCGCAACACGTGCGCTGGATAAACGCTTGTTAACTGGAGCTCCCTCTTTGCCCACTAGCAGGGCATTAGCTGGTGCTGAAGACAGTGGCTGCTTGGCG
AGCCTGGATCTCCAAGTGACCCCCTCAGCAACTCCTGATGAACAGGACTGAAGCCAATATTAAAGCAAGTCAACCAAAGGTTCTCTGGTGTAGACAA
GACAGCAAAAGGACAGACTACCTTGTGGAACCTAGCATTGTTCTCCTTCTGCAGCACTAAGTAACATTTGTTCTCCGTTAAGATCTTTGCAAACCACAC
ACAAGAATTGCTGGTCATCCTGCCAATAGATGCTGCTCACAGAACCAAATTTCCTGTGCTGAATTGTCACTCATGGGCTTGAGAGTAGGAGACTGGAG
ACCAAGGTGGCTAGAATCCAGTTGGGCCTGATGTCTCCCTGTTGAAAGGGCTCCTTGTGGAATGAATAGCACATGGCTCCTGTGGTGGATCTGATAGTG
GCATAGCACCAAGTGATGCAGGCCTGCCAGGGGCCACAGACACAGAAGATGCTCCCGGGGTCCCCCATGTACTCCAGACACACTGCAGGCCACCTCT
CCCAGCAGGTTGCCAGTCATGGGCCCCATCATCATGACTTCTGTCCAAGGTACTGTGTGCAGAAATGTGATTGAGATTCAAGTCAGGGCCTCTCTGCCC
TTTTCCCTCCAGAAACAAAACCAAGATAATTTATCCTGAACACGGTGAAAAAAGGAAGGGAGGGAGGAGAAAAAGTCCGGGTCTCACCTGGGATTCT
CTGTCTCCTGCAACATGAAGGATTTAGCCTGGGAGGAGGTGGTGAGAACTCTGGGAGAGAAAAAAGAAGGAAAGAATAGTTTTACCCATGCTGAAGT
TAATTTAAACCTTCACCTAGAGAAGCAAAAAAAAAAAAACCCACACTTTCCCATTTTGTGCCTCCCTTCCTAGAGTTTTAGCCAAAGGTTTAGCTAAGTA
ATTGGTTTTACCAGCGCACTCACTCCTCCTATCCCAAGTCTGTTTGACTCCCTCCCCATCATCCTCCTCACCTCTTTTCAGGCAGGGTGGGGATAGCAGC
AGGAGGAGATTTTGGGAGCCTGGCAACTCCTGCAAGGACCGCAGGACAGCCCCTCTGTGGGGATGCGTGGTGCCCCATCTGCCGCCCTTCTGAAGAA
TGCACTGCCTTCACTTTTTACTGTGTTAGAGTCCATCCAGACTGTTCTATCCAAAAAAGTTTCTTTTTCCCCCACAGGCAATCAGGAAATGATTCCTTTC
CCGACTGCTTCTGTCTAGTGCCTGGGAATCTTGAGTCAATCCCTCAGTAAGTCAGTGACTAGGGAAATCCCTCTCTGAGCCTCCCAGTTCATGTTGCTT
AGGGAACCTGATATTTTCGTGAAACCTGCCTACACATGGGCAGCCCAACAGCAGAACAAATGGTGGTGACCAAAGTGAACAAAGAAGTATAGTTGTG
CCAGCTTCGTAGTTGCCCATGTGGACAAGTCAGCAGGATCAGGACACGAGGAAGAGTAAATGTGAGACAGTCAATGTGACTTCTGCGATAAACAGAT
TTTTAAACCCCGAAATTTTGCAAAATTTTGGTGAAACCTGAACTTTCTTCGTTGCATATACTGGCACTATCTGTACCATCATACAACTGTCTCACATTAAA
GCTATTTTTCTTGGGCACTGATGAGTAAGGTTGGTATAAGTTCCTCAGATCAACAAAAACCCATTTTCCTGTAAGTCTTACATTTAGTATTTAAGGAACTA

AAACTTAAATACATTTTGTGAAATGGTTGACACTTCACTGATAATGATTTATTGCTTGGATTAATAAATTTTCCAAAAGTTGTCTTATGTAGAATATGGTTT
GCAACCAGCAGAACCATTAATCTATACTGCAATGATATGCACTATGTATAATTGTTTAAAAGCCTCTACTTAATGATGTAAAATGCTCTATTTAATTACACA
TTTGGGTAAACTGTATACTAACATCTGATGGCATTTTTCCACTGTTTGTTGCTTTTTTCAAATACTTTATTGTACAAAGCTGTTCTTAATATTTTTCAAGTT
TTTTTCTTTGAATTTTGCTAATGTTTTCCTTGAATTATGAGCACTGACAGAATGTGCTTAGCACTTTTGGCTATTCACACAGCTTTTGAGCATGATTTGCA
TCCAATATTTACATTGCTAGCAATAATAAGCCATCTGTGAGTTTTGTCAAAA
>ENST00000426882.5|ENSG00000231074.8|OTTHUMG00000031138.4|OTTHUMT00000256033.1|XXbac-BPG283O16.6-002|HCG18|4862|
GGCAAAACGTCCGCCCGGGCCCGAGGCGGGCGGAGGCAGCTGTGGTTACGTGCGGGGGGCCGGTGACGCAGTCGCGGGGCGCTGGGACGCGGCTT
GCGGAGCCGGCCGGCGAGCGCAGGCCCCAGGCTCGGGCGCGGCGGCGGGACAGCACCTACCTTCCCGGCTTCGGGTCCGGGAGTGCGGGGCGCGG
AGCCGCCGGAGCGCTGCCTCCCTCCTTCCTCCTCCCCTCGCCCCCGCCCCGCGGCGCGACACACAATACTCGCCGGAAGCGGAAGCCGCGCCGAGGC
TCGTGTCAGTGGAAGCTGGGGTGTCGGCGGGCGGCCGCGGCCGGGTCTAGCGGTGCCTGCGGAAGAAGGAGGAGGAGGGCTGGCGATGAGAAGCA
GCAGGGGGAATCAAGGGCAAGAAGGGACCCAGAGGGGCAGGTGGCTGAGATGCTGGCGTCCAGGTGACCCGAGGAATCCTCTGGAGATACAGGCCC
GCGCATGCGCGTGCTCGAGCGCTGAAGCCCATTGCCCGCAGGGCGCTGGACTGGCGCCCCGGCCGGCGAGGCTGTCTAGTCCAGGCTCCAGGCCCCG
GCTCTGGCCAGGCGCCGCCGCCTCCCGGTGCAGCTGAAAGTCGACGAAGAGGGCGGGGCGGGGGCGGCTTGTGGACTTCCACACTTTTCCTCTCTTC
GGGCCAGTCTGGGGAGAGGGTGGCTAGCTGTGGCGGCGGCCAATTACGTGGCGGACCTGCGGGGCTAAGCTGGCACGTCACTTCCGACTAGTGGAGT
TTTTAGCCTTTTAACATTCAAGGATTAGAGCTCCCTCTTTGCCCACTAGCAGGGCATTAGCTGGTGCTGAAGACAGTGGCTGCTTGGCGAGCCTGGATC
TCCAAGTGACCCCCTCAGCAACTCCTGATGAACAGGACTGAAGCCAATATTAAAGCAAGTCAACCAAAGGTTCTCTGGTGTAGACAAGACAGCAAAA
GGACAGACTACCTTGTGGAACCTAGCATTGTTCTCCTTCTGCAGCACTAAGTAACATTTGTTCTCCGTTAAGATCTTTGCAAACCACACACAAGAATTG
CTGGTCATCCTGCCAATAGATGCTGCTCACAGAACCAAATTTCCTGTGCTGAATTGTCACTCATGGGCTTGAGAGTAGGAGACTGGAGACCAAGGTGG
CTAGAATCCAGTTGGGCCTGATGTCTCCCTGTTGAAAGGGCTCCTTGTGGAATGAATAGCACATGGCTCCTGTGGTGGATCTGATAGTGGCATAGCACC
AAGTGATGCAGGCCTGCCAGGGGCCACAGACACAGAAGATGCTCCCGGGGTCCCCCATGTACTCCAGACACACTGCAGGCCACCTCTCCCAGCAGGT
TGCCAGTCATGGGCCCCATCATCATGACTTCTGTCCAAGGTGTGCTCGGAAATCTCTTCCTTAACTGTGACTTTCTGACAGGTGGAGGATGTGGTCAGG
AGTGGGAAAAGGATTCGAGACGGGAAGAGGGAGGGGTTCAGGATGAAGAGAGATAATCTGTGCCACAGATGCTGGCCCTGCAGTTAGCTCCCACCTA
GTCCGTGCACACACATTCTAATCCCCTCCCATTCCTTTACATGCTGCGGCCAGAGAGGCCTTTCTCAAAGTGGAAGTCTCATCCTCACTTCTCTGGTTAC
AGTGCTGGGCCATGGTAACTTACAAGGCTTAGCAGGAACTGTCTGCGCACTCCCCCTTCCTGCCCACTACCTTGTTTCCCTCCAGTTGCGAGAGAAAA
CATTGATTGAGCATTAACTATGTGCCAGGCTTGTCCTAAGTCCTTTGCATGTATTCACTCAAACAATCCTCACAACATTCCTATCACATCTCCCATTTCAC
AGTGAGGGTTCTAAAGCACGTAGTGGGTGAGGAACTTGTCCAGGGTCACACAACTAAGTGGGGGTGGAGAAACATCAAATCTAGGTGGTCCAGGTGG

TGGCCAGAACCCATCAGCACCTCACTACAGCTGCCTCCAGTTTCTCGCTCTAGGAAATACTCTTCCCTGTCAACTCCTGTTGTCCTCTGGGCTCAGTTG
ACATGTCATCTCCTTGAGGAGGACATCCTGAGATGCTCCCCGACTAGGGTGGGCACCCACTTCCACACTCCAAATGCCTATTTGACCTGTGTATCGCTG
CATCCCCACTGCCTGGCAGATAGCAGGCCCCTAATAAATATGATTTGAGCAAATAAATATAGTTCTTCAAAAAATAGGGAGGACTTCTTGCTTCAGGTAA
TGGTAGGCTAGGACATTTGGACCAACCCTCCTGCAGAAAATAGCCATTTATTTTGATGCAATATATCTGGCTATGACTAGAGATTTCAAATATTTGGAGG
GCTATTATGTGAAGAACTAAACTTACTCTTTGTGACATAGAGGTCAGAAATTGGTCGTTTCCATAGAGTATGAGAGAGACAGAATTGTGCTCATCATAAG
TATCTTTTTTAAAAGTTAGACTTAATAAAATGGGCTGCCTTGGTAGGTGGTGAGTTCTCTGTCACGAAAGAGATTCAGCCACTTGCTCTGAGAAAGCAT
GGAGAATATCAACATTGTAAGGGTCGACCACCTCATCTCTGAGGCCCCCTCCTCACTTAGGTTGCCATTGCAGGTGGAACACTGGGCTGAGTGTCAAA
GAACCTGGGTTCCACAAGCTGTGCGACCATGGCTGGTTGTTTTGGATCTCATTCCTAATATCTGTTAGTTAAGCTGACCCGTCAATACTGACCCACTTCA
CAGAGTTATGAAGGGATTAAATTAGGTATTACATGTAAATCTGTTTTGTTTGCTTTTTGTTGTTCTTTTTTGTTTTGCTTTTTTCTTTGCATTATGAAATCCC
AAGTCAACTTTTTCCATTCTTTTATTTTTTTAAAAATTGACATCATCATTATAACCACAAAATAATTTTTAAAATGGAAAAAAAAAAACTCACCTGCAACCC
CACAATCTAATACAATAATCATACTTTTTTCCCTTTATTCTCCTTTATTGTGACCAAGGATGACTTTGGGCATTGCTGTGAGGAACTATTTCGTTTAACACC
CTACTACCTGAACATGATGCGTACTCAGCCTTTCACCACCCCCCAAGTAGCTACACATGATGTGATAATAGTATTGGTGGTGGTTTTAGAGAGTTGGAGA
GAAAACTGAAGTTGGATTTGGGTAAAAGAAAAAAGAGTGGGAATTATTTTTTCTCCATTCTAAAGCCCATAAAGCAATGTTAATTGTTCCCACCCCTCT
GCTATTCTGTCTCCTCAGGTACTGTGTGCAGAAATGTGATTGAGATTCAAGTCAGGGCCTCTCTGCCCTTTTCCCTCCAGAAACAAAACCAAGATAATT
TATCCTGAACACGGTGAAAAAAGGAAGGGAGGGAGGAGAAAAAGTCCGGGTCTCACCTGGGATTCTCTGTCTCCTGCAACATGAAGGATTTAGCCTG
GGAGGAGGTGGTGAGAACTCTGGGAGAGAAAAAAGAAGGAAAGAATAGTTTTACCCATGCTGAAGTTAATTTAAACCTTCACCTAGAGAAGCAAAA
AAAAAAAACCCACACTTTCCCATTTTGTGCCTCCCTTCCTAGAGTTTTAGCCAAAGGTTTAGCTAAGTAATTGGTTTTACCAGCGCACTCACTCCTCCTA
TCCCAAGTCTGTTTGACTCCCTCCCCATCATCCTCCTCACCTCTTTTCAGGCAGGGTGGGGATAGCAGCAGGAGGAGATTTTGGGAGCCTGGCAACTC
CTGCAAGGACCGCAGGACAGCCCCTCTGTGGGGATGCGTGGTGCCCCATCTGCCGCCCTTCTGAAGAATGCACTGCCTTCACTTTTTACTGTGTTAGA
GTCCATCCAGACTGTTCTATCCAAAAAAGTTTCTTTTTCCCCCACAGGCAATCAGGAAATGATTCCTTTCCCGACTGCTTCTGTCTAGTGCCTGGGAATC
TTGAGTCAATCCCTCAGTAAGTCAGTGACTAGGGAAATCCCTCTCTGAGCCTCCCAGTTCATGTTGCTTAGGGAACCTGATATTTTCGTGAAACCTGCC
TACACATGGGCAGCCCAACAGCAGAACAAATGGTGGTGACCAAAGTGAACAAAGAAGTATAGTTGTGCCAGCTTCGTAGTTGCCCATGTGGACAAGT
CAGCAGGATCAGGACACGAGGAAGAGTAAATGTGAGACAGTCAATGTGACTTCTGCGATAAACAGATTTTTAAACCCCGAAATTTTGCAAAATTTTGG
TGAAACCTGAACTTTCTTCGTTGCATATACTGGCACTATCTGTACCATCATACAACTGTCTCACATTAAAGCTATTTTTCTTGGGCACTGATGAGTAAGGT
TGGTATAAGTTCCTCAGATCAACAAAAACCCATTTTCCTGTAAGTCTTACATTTAGTATTTAAGGAACTAAAACTTAAATACATTTTGTGAAATGGTTGA
CACTTCACTGATAATGATTTATTGCTTGGATTAATAAATTTTCCAAAAGTTGTCTTATGTAGAATATGGTTTGCAACCAGCAGAACCATTAATCTATACTGC

AATGATATGCACTATGTATAATTGTTTAAAAGCCTCTACTTAATGATGTAAAATGCTCTATTTAATTACACATTTGGGTAAACTGTATACTAACATCTGATG
GCATTTTTCCACTGTTTGTTGCTTTTTTCAAATACTTTATTGTACAAAGCTGTTCTTAATATTTTTCAAGTTTTTTTCTTTGAATTTTGCTAATGTTTTCCTT
GAATTATGAGCACTGACAGAATGTGCTTAGCACTTTTGGCTATTCACACAGCTTTTGAGCATGATTTGCATCCAATATTTACATTGCTAGCAATAATAAGC
CATCTGTGAGTTTTGTCAAAA
>ENST00000412685.6|ENSG00000231074.8|OTTHUMG00000031138.4|OTTHUMT00000076242.4|XXbac-BPG283O16.6-001|HCG18|2552|
GGCTCGTGTCAGTGGAAGCTGGGGTGTCGGCGGGCGGCCGCGGCCGGGTCTAGCGGTGCCTGCGGAAGAAGGAGGAGGAGGGCTGGCGATGAGAA
GCAGCAGGGGGAATCAAGGGCAAGAAGGGACCCAGAGGGGCAGGTGGCTGAGATGCTGGCGTCCAGGTGACCCGAGGAATCCTCTGGAGATACAG
GCCCGCGCATGCGCGTGCTCGAGCGCTGAAGCCCATTGCCCGCAGGGCGCTGGACTGGCGCCCCGGCCGGCGAGGCTGTCTAGTCCAGGCTCCAGGC
CCCGGCTCTGGCCAGGCGCCGCCGCCTCCCGGTGCAGCTGAAAGTCGACGAAGAGGGCGGGGCGGGGGCGGCTTGTGGACTTCCACACTTTTCCTCT
CTTCGGGCCAGTCTGGGGAGAGGGTGGCTAGCTGTGGCGGCGGCCAATTACGTGGCGGACCTGCGGGGCTAAGCTGGCACGTCACTTCCGACTAGTG
GAGTTTTTAGCCTTTTAACATTCAAGGATTAGGTAAAAGTCTTGCAGTGAAAAACCCGAGGACCCTTACCGCAAGTGTCTTTTGCTCCCAGCTACTGAT
ACTGGATTCCACTCGTGATTCTCCCTTTCTTAGCGCATTCATGATATAGACATCAGTCTCTGAGCTGGAGGAGGACAAAGGCAGCGGTCCTGTGAATTCT
ATGCTCTAGCTTGGGTTAAGGGATTTGGAATTGCACTTGTTTCAGAGAGCTCCCTCTTTGCCCACTAGCAGGGCATTAGCTGGTGCTGAAGACAGTGGC
TGCTTGGCGAGCCTGGATCTCCAAGTGACCCCCTCAGCAACTCCTGATGAACAGGACTGAAGCCAATATTAAAGCAAGTCAACCAAAGGTTCTCTGGT
GTAGACAAGACAGCAAAAGGACAGACTACCTTGTGGAACCTAGCATTGTTCTCCTTCTGCAGCACTAAGTAACATTTGTTCTCCGTTAAGATCTTTGCA
AACCACACACAAGAATTGCTGGTCATCCTGCCAATAGATGCTGCTCACAGAACCAAATTTCCTGTGCTGAATTGTCACTCATGGGCTTGAGAGTAGGA
GACTGGAGACCAAGGTGGCTAGAATCCAGTTGGGCCTGATGTCTCCCTGTTGAAAGGGCTCCTTGTGGAATGAATAGCACATGGCTCCTGTGGTGGAT
CTGATAGTGGCATAGCACCAAGTGATGCAGGCCTGCCAGGGGCCACAGACACAGAAGATGCTCCCGGGGTCCCCCATGTACTCCAGACACACTGCAG
GCCACCTCTCCCAGCAGGTTGCCAGTCATGGGCCCCATCATCATGACTTCTGTCCAAGGTACTGTGTGCAGAAATGTGATTGAGATTCAAGTCAGGGCC
TCTCTGCCCTTTTCCCTCCAGAAACAAAACCAAGATAATTTATCCTGAACACGGTGAAAAAAGGAAGGGAGGGAGGAGAAAAAGTCCGGGTCTCACC
TGGGATTCTCTGTCTCCTGCAACATGAAGGATTTAGCCTGGGAGGAGGTGGTGAGAACTCTGGGAGAGAAAAAAGAAGGAAAGAATAGTTTTACCCA
TGCTGAAGTTAATTTAAACCTTCACCTAGAGAAGCAAAAAAAAAAAACCCACACTTTCCCATTTTGTGCCTCCCTTCCTAGAGTTTTAGCCAAAGGTTT
AGCTAAGTAATTGGTTTTACCAGCGCACTCACTCCTCCTATCCCAAGTCTGTTTGACTCCCTCCCCATCATCCTCCTCACCTCTTTTCAGGCAGGGTGGG
GATAGCAGCAGGAGGAGATTTTGGGAGCCTGGCAACTCCTGCAAGGACCGCAGGACAGCCCCTCTGTGGGGATGCGTGGTGCCCCATCTGCCGCCCT
TCTGAAGAATGCACTGCCTTCACTTTTTACTGTGTTAGAGTCCATCCAGACTGTTCTATCCAAAAAAGTTTCTTTTTCCCCCACAGGCAATCAGGAAAT
GATTCCTTTCCCGACTGCTTCTGTCTAGTGCCTGGGAATCTTGAGTCAATCCCTCAGTAAGTCAGTGACTAGGGAAATCCCTCTCTGAGCCTCCCAGTT

CATGTTGCTTAGGGAACCTGATATTTTCGTGAAACCTGCCTACACATGGGCAGCCCAACAGCAGAACAAATGGTGGTGACCAAAGTGAACAAAGAAG
TATAGTTGTGCCAGCTTCGTAGTTGCCCATGTGGACAAGTCAGCAGGATCAGGACACGAGGAAGAGTAAATGTGAGACAGTCAATGTGACTTCTGCGA
TAAACAGATTTTTAAACCCCGAAATTTTGCAAAATTTTGGTGAAACCTGAACTTTCTTCGTTGCATATACTGGCACTATCTGTACCATCATACAACTGTCT
CACATTAAAGCTATTTTTCTTGGGCACTGATGAGTAAGGTTGGTATAAGTTCCTCAGATCAACAAAAACCCATTTTCCTGTAAGTCTTACATTTAGTATTT
AAGGAACTAAAACTTAAATACATTTTGTGAAATGGTTGACACTTCACTGATAATGATTTATTGCTTGGATTAATAAATTTTCCAAAAGTTGTCTTATGT
>ENST00000602698.5|ENSG00000231074.8|OTTHUMG00000031138.4|OTTHUMT00000468042.1|XXbac-BPG283O16.6-017|HCG18|573|
AGCTCCCTCTTTGCCCACTAGCAGGGCATTAGCTGGTGCTGAAGACAGTGGCTGCTTGGCGAGCCTGGATCTCCAAGTGACCCCCTCAGCAACTCCTG
ATGAACAGGACTGAAGCCAATATTAAAGCAAGTCAACCAAAGGTTCTCTGGTGTAGACAAGACAGCAAAAGGACAGACTACCTTGTGGAACCTAGCA
TTGTTCTCCTTCTGCAGCACTAAGTACTGTGTGCAGAAATGTGATTGAGATTCAAGTCAGGGCCTCTCTGCCCTTTTCCCTCCAGAAACAAAACCAAGA
TAATTTATCCTGAACACGGTGAAAAAAGGAAGGGAGGGAGGAGAAAAAGTCCGGGTCTCACCTGGGATTCTCTGTCTCCTGCAACATGAAGGATTTA
GCCTGGGAGGAGGTGGTGAGAACTCTGGGAGAGAAAAAAGAAGGAAAGAATAGTTTTACCCATGCTGAAGTTAATTTAAACCTTCACCTAGAGAAGC
AAAAAAAAAAAACCCACACTTTCCCATTTTGTGCCTCCCTTCCTAGAGTTTTAGCCAAAGGTTTAGCTAAGTAATTGGTTTTACC
>ENST00000602861.5|ENSG00000231074.8|OTTHUMG00000031138.4|OTTHUMT00000468043.1|XXbac-BPG283O16.6-018|HCG18|372|
AGCTCCCTCTTTGCCCACTAGCAGGGCATTAGCTGGTGCTGAAGACAGTGGCTGCTTGGCGAGCCTGGATCTCCAAGTGACCCCCTCAGCAACTCCTG
ATGAACAGGTACTGTGTGCAGAAATGTGATTGAGATTCAAGTCAGGGCCTCTCTGCCCTTTTCCCTCCAGAAACAAAACCAAGATAATTTATCCTGAAC
ACGGTGAAAAAAGGAAGGGAGGGAGGAGAAAAAGTCCGGGTCTCACCTGGGATTCTCTGTCTCCTGCAACATGAAGGATTTAGCCTGGGAGGAGGT
GGTGAGAACTCTGGGAGAGAAAAAAGAAGGAAAGAATAGTTTTACCCATGCTGAAGTTAATTTAAACCTTCACCTAGAG
>ENST00000413358.6|ENSG00000231074.8|OTTHUMG00000031138.4|OTTHUMT00000256036.2|XXbac-BPG283O16.6-005|HCG18|916|
CAATACTCGCCGGAAGCGGAAGCCGCGCCGAGGCTCGTGTAAAAGTCTTGCAGTGAAAAACCCGAGGACCCTTACCGCAAGTGTCTTTTGCTCCCAG
CTACTGATACTGGATTCCACTCGTGATTCTCCCTTTCTTAGCGCATTCATGATATAGACATCAGTCTCTGAGCTGGAGGAGGACAAAGGCAGCGGTCCTG
TGAATTCTATGCTCTAGCTTGGGTTAAGGGATTTGGAATTGCACTTGTTTCAGAGAGCTCCCTCTTTGCCCACTAGCAGGGCATTAGCTGGTGCTGAAG
ACAGTGGCTGCTTGGCGAGCCTGGATCTCCAAGTGACCCCCTCAGCAACTCCTGATGAACAGGACTGAAGCCAATATTAAAGCAAGTCAACCAAAGG
TTCTCTGGTGTAGACAAGACAGCAAAAGGACAGACTACCTTGTGGAACCTAGCATTGTTCTCCTTCTGCAGCACTAAGTAACATTTGTTCTCCGTTAAG
ATCTTTGCAAACCACACACAAGAATTGCTGGTCATCCTGCCAATAGATGCTGCTCACAGAACCAAATTTCCTGTGCTGAATTGTCACTCATGGGCTTGA
GAGTAGGAGACTGGAGACCAAGGTGGCTAGAATCCAGTTGGGCCTGATGTCTCCCTGTTGAAAGGGCTCCTTGTGGAATGAATAGCACATGGCTCCTG
TGGTGGATCTGATAGTGGCATAGCACCAAGTGATGCAGGCCTGCCAGGGGCCACAGACACAGAAGATGCTCCCGGGGTCCCCCATGTACTCCAGACA

CACTGCAGGCCACCTCTCCCAGCAGGTTGCCAGTCATGGGCCCCATCATCATGACTTCTGTCCAAGGTGTGCTCGGAAATCTCTTCCTTAACTGTGACT
TTCTGACAGGTGGAGGATGTGGTCAGGAGTG
>ENST00000602290.1|ENSG00000231074.8|OTTHUMG00000031138.4|OTTHUMT00000468044.1|XXbac-BPG283O16.6-016|HCG18|821|
CAAGGATTAGGTAAGTTCTCCCCGCGGTAGACTTCAAATACTCTTTGTCCTGCCCCTGCTTGGCGAAATTTACCGAAATTATCTACCTATCCCGCTGGAG
TGGGAGCCGGGGGAGAGCTGGAACCGCGTCTCTGCTGCTCACCTTTGTGTCCTTAGCTCCTTCGCATGGGGTCCGACCGCAACACGTGCGCTGGATAA
ACGCTTGTTAACTGGGTAAAAGTCTTGCAGTGAAAAACCCGAGGACCCTTACCGCAAGTGTCTTTTGCTCCCAGCTACTGATACTGGATTCCACTCGTG
ATTCTCCCTTTCTTAGCGCATTCATGATATAGACATCAGTCTCTGAGCTGGAGGAGGACAAAGGCAGCGGTCCTGTGAATTCTATGCTCTAGCTTGGGTT
AAGGGATTTGGAATTGCACTTGTTTCAGAGAGCTCCCTCTTTGCCCACTAGCAGGGCATTAGCTGGTGCTGAAGACAGTGGCTGCTTGGCGAGCCTGG
ATCTCCAAGTGACCCCCTCAGCAACTCCTGATGAACAGGACTGAAGCCAATATTAAAGCAAGTCAACCAAAGGTTCTCTGGTGTAGACAAGACAGCA
AAAGGACAGACTACCTTGTGGAACCTAGCATTGTTCTCCTTCTGCAGCACTAAGTAACATTTGTTCTCCGTTAAGATCTTTGCAAACCACACACAAGAA
TTGCTGGTCATCCTGCCAATAGATGCTGCTCACAGAACCAAATTTCCTGTGCTGAATTGTCACTCATGGGCTTGAGAGTAGGAGACTGGAGACCAAGG
TGGCTAGAATCCAGTTGGGCCTGATGTCTCCC
>ENST00000602319.1|ENSG00000231074.8|OTTHUMG00000031138.4|OTTHUMT00000468045.1|XXbac-BPG283O16.6-011|HCG18|3342|
GCCATGGCTCCTGAAAGAAATAAAAGATGATCATCTTTCTAAAAAGTCTTAAGTCTGAATTATTAGTAACTTAACTGGAGAATCTCACTTTTCCTACTCT
CGTATTTTAACCACAGTTGCTCTAACACAGACCTTTGAGGATCTTTTCATGACTTCATTCACAAATACCTATTTATGCTGTACAGATGCTACTAGGAAGGA
AATAGGGATGTCTGTTTTGACTGTGGAACTTAACTTGGTCTCGTCTCTTCGTGCATGCAACCCTGTCCTTGGGATAGCTTTCTTGAGCATATCTACTTATG
TTCAAGAGGTAAATTGTCCTGAAACCCCCATTGCTATAAGTATTTATTTTATTACTCATAATACTTAATGCTCCTAAAGTTGGGGTATTTTTTTTTTTGGATA
CCTAAACTTCATTGAGATACTTTGAACTATTTATAGAGAAAACGGAACCTTCTAATACCTGGCTTCTATTTCTTAAAATGTTATGATCATACATGGCTTAG
GGCTTTATGGCCAAATAACTTCACTGAACCCAGGAAAAAGAATAGATCCATCTGAAACAGACCTGTAGCTTCCAGAGGCCTAAATTTTCGGCTCCATTT
GTATCCTTCATTTTCTGTGAGGTAAAGAAGTGGAAGGAGACAAGCCTCAGCCCTTCCCCTGGCACCTTTACTCTTCGCCCTTCCTCCTGGCATGGTGGA
AAGTGCACTGGAGGAGGAGTGAAGGGCCCTAGGTTTGCATCCATATTCTGCCACTTGCCAACCTTAATGGCCCTTACAATTGATTTACCCTCATGAAAT
TTGGAATGATTTCTAAAGTCTTTCCTCGCCCTGAATGTTAACATTTTTTGATAGTCAGGACTTTCTGTAGCTTCACCTTCCTTATTTAGTGTTATTTTTTTC
TCAAGACTGAACAGAGAGGGAAGCTGTCAAAGTGTGCTGGGCACACACCCTGCAGTGGGGCAATGGCCAATTCTAATCTCAAGTCATTAGGCTGCAG
TAGCATGACCACTGCTTCCTGTCTACCCTCAGAGGGTAGAGACAGCTGAGCTCCTGTAGTTGGGGTCAGGCCCAGCCACTCTGTGGGGACAGTGATTA
GTGTTGTGTCACCAATTCAGGGAAGGAGCCACCTTGTCTTATTTTCCCTCTTGAATTATCTTGATATGACCCCATTATAAATTTCCTTTTGTAAACCTCTGT
CTCCCAATTTCTCCTTTTAGCTTACTTTCTATTGAAGTAGAGGAACAGAGTACAACTTCCATCCTCTTTCATCAGCCCTGAGAGCAGAACGCAAGCGCC

GTTACTGGGAACTATATCCTTGGCTCCCTGGATGTGGCTATTAACTTCTGGCCTGCCACTCTATCACATACACATATGGAGATGGTGTCATCCATGTACCT
TACCCCGTATTTACAACTTCTATCACCCAACAGTGCCAATGGCCCTGATGGTCCCTCTGGGAGGGAGAGAAGAGTAAGCTGGAGTCACCCCTTCCCTGT
ACTTCCCACCTCGCCAGGCCTGTTGGTGTTAGTGTCCCTTCTGATCTTGGCCTGACCCCTGTGCCCTGGGCACTGGGCTGCAGGTTGGAGAGGCAGCA
TGATGGAGTGGGGATAACACATACTCCAAAACCAAACAGAAGCCAGACCTGGGTTGGGTCCTGGCGAAACAGTCTAGAGGCTTGGTGACCTTAACCT
CCTAATTAATCTTCCTAAGCATAAGTTTCCTTATCATAAGTTATGTATGATAAAATTTTCCTTGGATGCATTCATTTTAGCATGACTTGAAATTATGTGTGAA
GGAACCTGGCCCACGGAAGTTGCCCTGTAAATTCAGATTCACTTTCCCTTGGACATATGGATGACATTAGCTCATTACAGTTATGACCTCCCTAAAACTC
CCAAATATTCTTTAAGTTCTTCTCTTATTTTCCCTTTAGTTTGTAGTCATATTTCTTAGTTCTTATATCAGTTGGGATTCCCACATCTTCTAGTTGGACAATAT
TGGAGAAGACACCACATTTTAACTGAGTTCCAGTGATATGACAGGCTTTCAATTCTCTAATCTCACAGAAGTTAGAAAAAAAGTAGATAATCAAAATCC
ACAGAAAATATAGAAGATTCCATTAACTCTGAGAATGATTCTCAGGTATCCTTAGGACCTCAAGAAAGCTGTTCTCTCCTGGGCCTGTAGAGAGTTCAA
GTGCCAGGAATCTACCACAAAGTAGCCGGGAGGTGCAGGGCAGCAGGGGGCACAGTGAAGTGCTGAAGGGCTTCTCAGTCTTCTTTAATTAGAGTGA
GAAGAAAAGAGCACCTCCTCATTTTAGAGTACATGGTGTGAACTCACTCTCAGCTGCCAAGTGAGCTTCACCTTGGGCTGTTTTGCATGCTTTCTCCTA
GTGCTTTAAGCCACCCTGAGATGTACAGACCAATACTGGCCATCACAAAAATATACTCGAGTACATAGACCATTGACACTATAAAGCAAGTAAACAATG
AAGTCTACATAACAGCCAAATAACAACATGATGATAGGATCAAATCTGCACATATCAATATTAACCTTGAATGTAAATGAGCTAAATGCCTCAATTAATAG
GCAGAGAGTGGCAAGTTGGACAGAGAAGCAAGACCCAACTGTATGTCTTCAAGAGACCCATCTCATATGCAGGGACACCAATAGCCTCAAAGTAAGG
GATGGAGAAAGATCTATCAAGCAAATGGAAAACAAAAAACAGCACTCTCTGTCCAACAAAAACAGAATATACATTCTTTTCAGCTGCACATGGTACAT
ACTCTTAAAATCGACCACAATTGCTTTATTGGCCAGAAAGCAATTCTCAACAAATTCAAGAAACCTGAAATACCGGCCAGGTGTAGTGGCTCACACCT
GTAATCCCAACACTTTGGAAGGCTGAGGTGGGCAAATCACTTGAGGTCAAGAGTTTGAGACCAGCCTGGCCAACATGGCAAAAACCCATCTCTTCTA
AAAAATATAAAAATTAGCCGTGCATGGTGGCATGCGCCTGTAATCCCAGCTACTTCGGAGGTTGAGTCACGAGAATTGCTTGAACCTGGGAGGAGGAG
GTTGCAGTGAGCTGAGATCACGCCATTGCACTCCAGTCTGGTTGACAGAGTGAGACTCATCTCAAAAAAACAAAAAAACCCTGAAATACCAACCACA
CTCTTGGACCACAGTGCCATAAAAATAAATACCAAGAAGATCTCTCAAAACCATATAATTAAGTGGAAATTAATCTACTCCTGAATGACTTGGGTAAAC
AAAGAGAAATTAAGGCAGAAATCAAGAAATTGTTTACAACTAATGAAAACAAAGATAAAAA
>ENST00000602516.1|ENSG00000231074.8|OTTHUMG00000031138.4|OTTHUMT00000468046.1|XXbac-BPG283O16.6-010|HCG18|2890|
TTAGAGGATCCTGTTTGGTAGTTAAAACCGTATTAAAATAGAGAGGGAAGAAATTAAGCCTCCTTGATGGTGAAGAGATTGGGAACCACCACTCCAGT
GTTAGAAAATGTCAAATGACAGAAGGTGGGCTATATTAAGTGGGAATTTCCAAACCCCACCCCCTGGAAGGAAGGAATGCTTGGTGACAAGCCTTAGA
GGAGAGAGATGCTTGTCTAGCCCATTGCCTGTGTGCCCAGGAAGAGATGTGCATACCTTGAGATATAGAGAAGACTCTAGTGGGGAGAAGCCCCAGGC
CAGCTTGTCAGCACAGGGCATCGGAGGCCCCCAACCAGCTCCAAGTTCTGAACAGCACACAGCCTTCAAAGGCTTGTACTGCTGCTCATACCCAGCA

GAGGCCTTGCACCAGGCCTCCCCATGCAAATCAGTGTCCCCGCAGCGTAGGTAAAGGAGCTCTAGCTGTGCTCCCTATGGGGGAACAGATATACCGTG
GGACACTGAGAGACTGGAAGATTGCCCAACATTTATTTACTGATCTGTGTTCACCACAGAACCTAAGGTATTTATTGGTATTGTTTCAACCAGTACGAGT
GATTCTTTCATTTAGTGGTCATCTCTGCACTCCACCACAGACTTTTGGGAATTACAAGATGAAAAGGGCCACATCCTCACCCTGGCATAGCTCAGAAAA
TTTGGTTGGGGAAGTAGGGACATAGACGTGATCACTACACCATAATTTACTAAGCTGTGAGAACTGGAGGTGCATCAGTGACCAGAGTGCATTTGAGC
CAGAGGTAGAAGGTACTGTAGACAAAAGGAACAACATGCGCTCTAGGCTGTATCATTAGGTACAATTTTCTGTCCTTGGGGGAGTCTTTTTAACCCTAA
GACAAAAATTATGAATATAGAAAGAGGCCACGGTTCATCTTTGCAATTCTTTCAGGAGAAAATTTGCTGCAACTCACTTTCTAAGTTTAAAAAAAAAAA
GAGTAAATGATATGAATATCACCTGAAAGAATTTGAGGTCTCAAACTTGGGAGGATCTTTGAACAACAATCTTGGGGAATGCCTAACAGTTCATACTCA
TTTCTTGATATCTACAGATGGAAACTCTACAGCCTTACATATATTTCTTTTTTCTTTTAACTACTTTATACTCACAGAAAATCAGTGTTCAGCACTTCTAAT
AGCAGGTCTAGTGGAAGCTTGAGATCAGAACAGAGTTCCATAAAGGGAAACTGAGGGCACACAAGGCAGAGCAGTACCCCCTTATCCATGGGGACCT
GTCTGACTGGAAGTAAGGGAGAAGATTCTACTCCACAGAGAATCAGGGAAGGTTAAAACTGTGTGTGTTTGTGACACTTTTGCTCTCTTGGGCCCAGA
TACAGGCCGGAAATCCTTTGGGTTCTGGTGACTCAAAGTGTGGCCAAAAGGACCAGCAGCTTTAGCTTCTCTGGGAGAACATATGAGTGCAGAATTTC
AGGCCTGCACCAAGTTTTTAAACGTCTGCATTTTAACAAGCTGCTCTTAATTTGCTTACGTATTAAAATTTCCAGAAGCACTGTTCTAGGACCATCTTTA
AGTGTTATCCAAAAACATGTAAGATCTTTTTTCAAAGCAAATTCTCAAGCTCTACCCTAGAGGGATTGCTTCTGGTTCTCTGGGGTTGGTGCAGGAATCT
ACATTTTCGGCAAGTGCCTTAGATCCTTGTGCACACTAGCATTTGAGGATTGCTGCCTTAGATGGTGGGAAATCTGAGCAGGGGAGGGCTGTGGGCTG
AAAATGAAATGATGATGTTTAGGAACTGGCACAAGCATGCTTCTGCTCCTGGTGGGAAGCAGTGGGACAGATCCAGTTGATTGTAGAGAATGCAGACC
TCGTGTCATGCTCAAGTGTCACCTTAATGTAATGGCTGGAGACAGCCACATATGACCCTCTCATTGTCCAGTCAGTAAGCTCAATCAATGACAGGAACC
ACTGACTCTGGTAATTAAAAACTTATTGTGGCCGGGCACAGTGGCTCACGCCTGAAATCCCAGCACTTTGGGAGGCAAAGGTGGGCAGATCACCTGA
GGTCAGGAGTTCAAGACCAGCCTGGCCAACATGGTGAAACCCCATCTCTACTAAAAATACAAAATTAGCTGGGCGTGGTGGGGTTGCCTATAATTCCA
GCTACTCAGGAGGCTAAGACAGGAGAAACACTTGAACCCAGGAGGTGGAGGTTGCAGTAAGCTGAGATCGCATCATTGCACTCCAGCCTGGGCAACA
AGAGTGAAACTGTCTAAAAAACAAACAACAACAACAACAACAAAAACACTTATTGGTGGGCAGGTTCTCATAAGAGGCCATGGGAAAGCCATGTCCT
ATCTCAGGGACACAGGGTCATCTGGGCCTCTGGCTAATAGAGGCCAAATAATGGGACTATTTTCCCTGTGAAATCCTGAAAACCAAAAATGGTGGCGT
CTTTATCTGCATTAGCAGAGGTAATTTGCTCCTTCTTGAAATCCAAGGTCACGTCTACTGTCTGGGGATTTTGATCCAGGGTCAGTGTGGTTTCTCCTTT
ACAGGAGAGCCGAGTCTCAGAAAGGTGAGGTGGTTTGTGTTGGTCATTGGCTACCTCAGATTTTAGAGCAGCTCTACCTTGATTGTGGGGTTGACCTA
ATTTTTTTTGCTGTCTTCTTTCTTCTCCAGGTGAGGAAAGAGGACTTCCTGTATATCTCTATCCTTTTGTTTCCATTACTCACTTTCTGTGGCTGCTGCTGC
AGAAGCCACTGCTGACTGATGTGGATACCTCAATCTTTGGTTTACAAAAAGCCTAGGTGTCTTTTGGCCTCTCTCCAGGTTGATAGCCATGGCTCCTGA
AAGAAATAAAAGATGATCATCTTTCTAAAAA

>ENST00000602498.1|ENSG00000231074.8|OTTHUMG00000031138.4|OTTHUMT00000468047.1|XXbac-BPG283O16.6-009|HCG18|327|
CCTGTTCTTTCCACTTCTGGGGCTGTGACTTGAGTTTCTCCGCAGGAGCAGATAGTGTTTGTGTAGGGACTGCGGACTTTTGCATTACAGCCTCCCTGC
TTTGCCCGCCTGCTGCTGGAAGCTTAGCAGGTGAGGAAAGAGGACTTCCTGTATATCTCTATCCTTTTGTTTCCATTACTCACTTTCTGTGGCTGCTGCT
GCAGAAGCCACTGCTGACTGATGTGGATACCTCAATCTTTGGTTTACAAAAAGCCTAGGTGTCTTTTGGCCTCTCTCCAGGTTGATAGCCATGGCTCCT
GAAAGAAATAAAAGATGATCATCTTTCTA
>KCNQ1OT1
>ENST00000597346.1|ENSG00000269821.1|OTTHUMG00000171022.2|OTTHUMT00000411275.2|AC021424.1-001|KCNQ1OT1|91667|
AGAACGGTCGCCGCGTCGCCTCAGCACGGACCTCCAGGGAGCTCCTCAGCAAGATCCTGCCAGGGCGCCCCTCAGCGCGATTCTGCCGGGGTGCCTC
TCAGCGTGGTCCTCCCCGGGGCTCCTCAGCACGATTCTCCCGGTGCGCCCCTCAGCGCGGTCCTCCTCGGTGCGTCAGTCATCGTGGTTCTCCCCGGC
GCGCCCCTCGGCGCGGTTCTCCTCGGGGCTCCTCAGCGCGGCGCTCTTCTGGGGGCTCCTCGGCGCAGTTCTCCCCGGGGACTCCTCGGCGCCGTTCT
CCTCGGGGCACCCGGGGCTTTTCGGCGCGGTTCTCCCCGGGGGTTCTTCGGCGCGGTTGTCCCCGGGGGTTCTTCGGCGCGGTTCTCTCCGGGGGCCT
CTCGCCGCGGTTCTGTTCTCCCCGGGGGCTCCTCAGCATGGTTCTCCTCCGCGCGGTCCTCCCTGGGCCTCCTCAGCGCGGCACTCTCCTGGGGGCTCC
TCAGCGCGGCACTCTCCCCGGGGGCTCCTCAGCGCGGCACTCTCCCGGCGGCTCCTCAGTGCGGTTCTCCCAGACTCTCCTCAGCGCGGCCCTCCCCA
TCTCTCTGGGAGGGTTTGAACACGGTCAGCACGGACCTGGGCGGACGGCGCGGGACGGGTGATCACTGGCGTTGCTGAGGTGAGCTGTGTGCCCCGC
GGCCGTCCCAGATCACAGGCGTCAGCAGTGCAGCCTGGCCTGGGCAGTGCGCTCCCATCTGCACCTTATGGACAGCGTGGCCAGGGTCGAGGTCCGA
GTTCCTGGCCGCGTCCCAAGGATCGGATTCCGGGTCTATAGTTCTCATGGTGGTTCAGAGTGGGCTGAATTGGGATTGGAGTCTGGAATCCGCATCGTG
GTTCTGAGTCCGCGCTATTGGGATGGAAGTTGGGAATCCATGTTGTGGTTTTGAGTCAAAGCCCGAATTGGGACCGGAGTCGGAAATCCACTTAGTGG
TTCTGAGTCAGGGCCCGAATTAGGATCGGAGGTGGGAATCCCCGTTGTGGTTCTGAGTTAGGTATCCTAGTTGGGATCAGAATTCGGGTCTAGGGTCCA
CATCCTGGCTGGGAGTTTGCCTGAGTTGGGATCTGAGTAGTTGGGGTCCCAGTCAGTCCGGGTCTGGAGTCCGCATGGTGGTTTTAGGTAGGGCCTCA
TTAGGGACAGAGGATCGGGAGTCTGAGTTGGGGACCTGAATCCCGGTCTGGAGTCTGCATCATGGCTCTGAGTTGAGATCCAAGGGAGGTCCGAGTT
GGCGACTGGAGTCAGGGTCTGGAGTCTGTCTTTGGGTAGAGGTTCTGAATGGGGGGCTCTGAGTTAAGAGATTGGGATTGGGGTTAGAGGTCTCAGT
GGGGTATGGGAGTCAGGAATTGAAGTCGAAGTTCTGCCTCTAGGTGGGGATTCTGAGTCAGGAATTAGGGGGCGAAGTAAAGGTTGCAGTCTGAGTT
GGGGGTGGTGATCACCTGCCAGGTGAGAGGTAGTGGTAGAAGTCTCTTGGTCATATTGGAGATGAGAGTTGATCAGATGCAGGTCTCAGTCTGGGTAG
TAGGCTTGAAGGGCCACCACTATGTTCAGATCCCAGTACCCCACTGTGGTCCCTGAGCAGGTCAAATAGCCTTTTATATAAAATTGAGAGTAAGAGTGC
CTTCCCACAGCACTGTTCTGGTTATGAAAGAGAATAATTCATCCAGGTAGAATAGTTCTGTCTTAGATTATCACAAAGTATAAAAACTTTCCTGATAATAT
CCACATTTGATGTGCATTTACCAGTTCTTTCTTTACTGTGGTGCCCCTTTTGCTAGACCAAAAGCTCCCAACGAGGAACTCTGTTTTGTTTTCTGCTGCC

TGCTCTGTACCCAGCCAAGAGCCTGATACACAGCCTTACTCAGTCAGTACTTGAAGGAAAGCAGGCAGGCAGGATTAACCAGATTTCTATGGTGTCTC
AAATGATCCATTTCTGCACCTGTTTTCCAGATACTCAGTAGGAGAAAAATTTAAGGATTGAGTTGCTTGATTATGGGTGTATTTTAATGTGTTTCCTAACA
TGCTGAGGAGAAAAGTGGGTCTGTTTCAGGATTTATGCTCCAGCTAGAGGGATTTTTTAAAATTGCGAATCAGAGCATGTCCAAATTCAGATAGAAAGC
ACAAGAAAACTATAAAGAATTTCCCTTATGAATGTAGATGCAGAAGTTCCTTGTACAATGCTAGCTAACTGAATCCAATAATATGTATTAAACAACAATT
CTTAAACAAGTGGGATTGCTCTTAGGAATGCAAAGATGATCCAATATCAGAAACTTTATCCATGTAATTCACATTAAGAGGCTACAGGAGAAAAACCAT
ATGGTTCCCTCAATAGAGGCAGAAAAAAATGATGTGATAAAGTTTAACGCCTATGTGTGATTTTGGGGTAGTGGAGTACACCACTTAGCAAGCCAGGA
CTAGAGAGGAACTTCTTCAACTTGATACAAGTTATAAACCAAAACTTAACTTTTCAGTAAACTTGATAGAGAAATTAAAAGTGTATCATTCTCTTTAAGA
TAAGGAGTAGGCCAAGGATGTCATCCTGGCCATACCCTAATACAAGAAAAAGAAATGGGGATGTGAGGATCAGGTAGGAAGTGATAAAACTTAGTGTA
GCTCATAGGGTTGATGGTCTCCATAAAGATTCTCAAACATTTTGGTGTTACGACTTGTTGTATTCTTAAAAGTTATTGAAACCCCCAAAGGGCTTTTCTT
GATAGAGATTCTATCTGTTGATAGTTACCATCTTAGAAGTTAAAACTAAGAAATGTTTAAAGCAGAACTATACAAGCACATATTCCACTGGGGTCATGGT
GTGCTGCCTCTGGAAAACTCTTTATCTTTGTGAGAATGAAAGAAAAGGCAAATAATGTCGAGTATTATTATGAAAATGTTTTTGACCTTTCCTGAAAGG
GTCTGGGGGGCTCTCTGGAATCCCCAGACTACACTTTGAAAATTACTGGGCTACATGGAAAAAAAAAATTAAGGAACTTAACAACTAAAGAGAGATTT
CAGATTTCAGAGAGGTTTTGGATATAAGATCAGTGTACTCAAAACAAAAAACAAAAACAAACCACATTTCTTTATGCCAATAGCAACTGACTAAAAAA
AAGCATAATAGAGATTAGGACAGCAATCAGAATAGCAGCAAAATTTATACCAGTCTAGGAATTAACAAAGACGACGACTTTTATGGAAAAAATTTTAAA
ACTCTATTGGAGGATATAGAGGGAGATCTTTCCATATGCAGAGACATTTTATTTTCTTGAACAGGGCAACTTGGCATTAGAATGACGTCAGGATTTTCCA
AATCTGTTAAGTTCAACAGGACTCCACTCAAACTTCCAGTTGGAGGGCTTGAGTAATTTAACAAACATATTCTAAAATGTATGTGGAAGAAAAATATTT
GTAAATGCCAACTGAACTTTTGAAAAGGAGAGCAGAGGACTTCTGCTGGCAGATACCAGGGCACACGCCAACTTCAGCAATCAGAGCGGTGTGGTGA
CAGAGGCCCAGAAGAGAGCTCAGAGGTAACTCCAAGTCTAGAGGAAGGGGACATGTGATAAAGGTGGCACCACATATCAGTGGGGACTGTTCAGAA
GATGATGTTGGAAAGCCAGCCCTAAAATGGAGGAATAGGAATATGGATTCCTAACTGAGCCCCTAGGAAAAGTAGACTCCAAATGGACTAAATATTTGA
AAGCAAAAGGTAGTTGCCATTTGTTTAATAGGAGAAAAAATGAATAATCTCCAGGGCCAGGGTGGCCGTGGGGTTCTTAAACAGGATCCAGAAAGCA
CAAACCTCAAGACTAAAATGTATGAACTTGAATGCATTAATGATTATGGATTAAGGATTTCTGTTAAATAAATCCATCATGGCAAGGGTCAAGACTGTTT
TCATAATAATACTAAGGAAACAATTTGCGTTTTTCACTTTTACACTCATGAATGTAACTTTGCCATGACTCATTACAAGATATTTGAAACAATTAAAGCTG
AGGAGGGAACAACAGCTAAAATATACCAGAAAGCACCACAAATCATTAAGAAAAAGGTAGGAAGATTGCCAGTTTGCAGGAGGGTTAACAAACAGAT
GAAAATTTGCCCAAGCTAATCAGTTATCAGAGAAATGTAAATGAAAGCAACAGCTATTCTTCATAGGAGAAGCATTAGAAAGTAGGATAAGGCCAAGT
GTTGGCAGAAGGCAAGGAACAAGCCTCCCTGTGTGCTGCAGGTGGGGACGGAGACAGGCACAGACGTTCTGAAGAGCTGGCCAACACTTCTTCCTC
AAATAAAATATTTTATACCTGTGGCCCAGCAGTTTCCCTCCTAAGTAAACATGCTGGAGCAGTTCTCACCCAGAACAACGAGAGGCTGTGTGTGTGCAT

GCTCAGGGCAGCATGGCCCATAGGGGTGGGCAGTTGGAGCTGGTCACTGTGGTTAGGAGCAGGGGACTAGATGTACACTTCACAAGATAGAGACGCG
TGGAAACACAGTGCTGAGTGACACAAATGCCAAACAGAAAGAGGGCCTCAGTACATGACTGTGTATTTAAATTAAAATTACACACCACCCAGACACG
CAGTACTAGGGAGTGCTCGTGAGTGCACACACACACACACAATGATATAAATAAATTACCCTGGAGAAGCTGTTTGTGTAGGGAGGAGAGCAAGGATA
GAGGCTAGAGATGAAGTGGAAAAGCATGGAGAGGAAGAGAGCGGTGACTTTATGCAGACCCTTACAGGGTGACAGTGTTTCATAGGCTGAGGGCTGT
GATCAACTCTGTGCAGAGTCCCAAAAGCCAAGAACCAACGAATGAATGTGCCTCCATGACTCTCCTGCCAGATACCCTCTAGTGGCTTCTATTTCTAGG
AAATAAAATTCAAATTCCATCAGGTTTTTACAAGATCTGCCCTGCTTACCTCTCTGACCACTGTCCTCCCTCACTGAGCTTTGGCTCTGCCAGCCTTCTT
CTTCTTTGTTAAATCTGCCAAGTATCCCTCCTGCCCCAAGGCCTTCACCACCTCTGCCTGGCTTGTCTTTTCTGGTGATCTTCCCTTGACAATTCCTTCTT
CTTATTTAAATCTCACAGGAAATTTTGCTCTGACCTGCCATATCTGTCATCCCCTTCAGTCACTCACTAGCTGACGACTACCTTTCGAGTCTCTGCAAAG
CACAGGCTACGCTTTTCTAGTTCCTTAGACATCTACTTATTTGTTGGCTGCCTATCTCTTCCACCGGAAGAGAGGCCCCAAGAGAGCATATCTGTCTTCC
GTATAGGGTCCGCACATGGTGGGTGTGTCACAGATTTCTGTTGAATGGTTTCATTCATTTATTGAACGAAGGAAGAGGCCAGGCCAGGAAAGTGTGCC
TCCATTTTCACAACTCAGTGAGAAAGCAAATAGCATGTCAAAATCTGACCCCTTGCAACCAGGGTTTCTTAGCCAGCCACACTGGTAATGCTTGGGAC
AGGATGTATCTTTGTTGTGAGGGGCTGTCATTTATATTGTAAGATGTTTAGCACCCTGCCTGGTCTCTACCTAGTTGATATTAGTAATTCTCCCATAGTCAT
GATAGCCAAAAATATCTCCAGACATTGCCATATGTCCCCTGGGGGGCACCGCAGATGACAACCACTTCCTTAGAACAGCTCTTGGGACTGGGATGGTGT
CTCTGCTGTGGCTCTTTAAAGGCAGCAGGCCCTGGCTTGCATCCTGCCACATCTAACACCTATAGCCTCACCACCTCGGTGAGAAAGACCCTGGCCCA
GGCCTGGCCAATCAGAGTACCCTCTTCCCTGGTCAGCAGTATAATTTGTTTGGCAAGCACTTCCAGTCCCACAACCTATGGGCTGCCACCCAGAGGTTT
AGTTGGAACTACGTGTCAGCTGCCGTCCCTCAGCCATCTATGGCATGGGCACCTGTGGCTTGACCAAGTTGGAGGTGGCCTAGAGAGGCCGGGCTGG
GCCTGCTCACCTTCTCCAGTTAGTGCAGACTGATGAATGAGTGGTTCTCAGCGGATACCGGATCCAGGTTTGCAGTACACTCCAGGAGGAGGACGAGT
AGGGTTTCCCTCAGAGCAGGCCTGGATGGGAGGGCAAGGGGTGGCCTCTAATACTGCCTTTCCTTCCGGTGCCTTTATCAGCCTGTGCCATGCTCCTCT
GAACTGGCCGGCCCCAGCATGTCCAGGTTACTGGCAGGAGCCTGTATAACCCAGGGTTCCAGTCACACCAGCCAGGAAGGCCCATGAGGCTGGAGCG
CACCCAGTCTCTGGCTGCTCTGTCTGCGGAACTAAAAGCCTTTCTCCCAGAGCCTTTCCTACGGCTGGGCGAAGCTCTTGTTTGCAATTTAAATGTGGC
CTCATTTATGGAAGAATCCCTGCTCCTCCGAAAAGCCTTGGGCTCAACCTCCCTAGCAGCCCGGGCCTCAGCCAGGAGAAGAAATGGATCTAAAAGGC
CTTTCTCCTGCTAATTGTAGCAATTAGCATTAGAGAATGGGGACTCGATGGGGGGCCAGCGCCTGGCAAAACAAGCTTATTCCCAGCTTAGGCCCAGGC
CCAGCTCCACACACTGCTGCTTGGCCAGACTCGGCCCCCACCCAGGGTCTGGTTGGCTTCAGGGCAGGGAGAGAGGGCCCAGGCCTGTGGGTGCTAA
GAGCAGTTGTGGAGGGTGTGCATCTGAGTTGTTAAGCCTGGTAGCTGTACAGGTAGCCCTGGGCTATGACTTGTGGTCCTCTCAGAAAAGGGTCTGAC
TGGAACGCAGAGCCAGACCAGATATTCATGAGTGGATCACAGAGTGTAGACTGAGGGCTATCTGGTGATTGACAGAAAGTAGTCTTGGGGCAAATGAC
TCTTAAAGAGTAAGGAGCCATTCCCCTCTATGGCTAACTGGATGCCTTTCTCTCCCCCTGCTCCAGAAAGGACTTCCTGTTCCTTCACTGAGCGTGCTT

GCTTATGAGGCTTCTGGAAACCAGGAGAGTTCAGTGGGCCTCATTATTGAGCGTATGGGCAGGACAACCTGCTTGGGACATAGGCCATGCTACAGTTG
ATTTTAGCCAGCTTGCTGCATCTACACAGCACAGTGCCTTCCCAGGCAGTGAATTATGTCTAACAGGCAGGGGAATGATCATTGCCTCATGTTAGGGAT
GCAGTCATTCAAGAAATATTTGTCAAACACTCAGCAGGTACTAGACCCTGGCCCAGAGGGAGGGATTTGATAGGAGCAGGGACCTGCCTACCCTCCCA
GGGTTAGTGGACAAAGGGGAGAAAGACTAACTAATGTTGAAGGACCGTTTGCACCCTGAGGGGAGTGCTGAGGCCTGGGAGCACAGCAGGTGTATA
GCAGGAACCCACTGGAACTAGCCTGGGGTCTGAAAAACTCCCTTGGGGAAAGTGGCAGGGGCAGGCCAAGGGCCCTCTGGTGGTAGGGAGCCTGTG
AGCACCTGGGATGGAAAGAAGCCAAGCATCTGAACTACCAGAAAGGCAGCTGCAGGATTAAGCAAGACCCACAGGTCTCTAAACTTTGAAAGGATGT
CTGTCCACTGAGTAGATAATTGATTGGAATGGGGCAGGGGATGTGAGAATGCCAGTTAGGAGATGCCTGCAGTACAGAGAAATGGTGGCCTATCAGGA
CAGTGGCAATAGGGATGGAGGGAAGCAGGGAACCTCTAGAACTATGGATGGGTGAAGTAAGCCTGGCTTGGTGATGGGCTTGAGGTGATGGGCAGGT
GGTGAGGAAGCGGGTGTATCGGAAATGACCCCAGAACTTCTGACTTGTGCCACTGAAAGGATGGGTGACACTGAAAGGACAGGGGCCTTCAGTAGA
GTGGGCTATAAAATGATGGAGGCTGTGGAGAGGAGGTGGGGGCAGGTGACTGGAATGTGGGTCTGGCTGGGTTGGAGAGACATTTGGGGGGTCAGG
GCCTCACAGGTGGTCTGGAAGCCCTTGGTGCTGAGGGAACAGCTTAGGGAAATGGTTTGCATAGAGAAAGGCACTGGCATTTAGTGGCCATGCCAGA
AGGGAAAACCTGCCAAGGAGACTGAGGATAGAGCCAGAAAGATGGCAAGAAAGCCAGGAGAACTGGAGGGATGGAAAAGAGGACCTAGGGGTCAT
GGTCCTGGCAGAGGTCACATTGGATCCAGTGCTTGTGATTCTCTGATTGCTGGCAGTGGATGGCCACTGGCCTGCAAATGGCCCTAGTGGAATGTCCCC
TGCCTGTGTGGCTCCCTGTGGGCTGCCTCTCCCCTGGACAGATGGGGAGAGACCCCTTCCCCACCTGGTTCTCTGTTTCACCCTTGGAACTGTGGCGG
TAAAGAGGACCTCCCCACAGTACGTGCAGGGAACAAGCTAGGTTGACTCCCTCAGGGAGGCCTCGTCCTTGCTGAGGTATTTCATAACCTCAGGATAG
AGAAATCCCCCCAACTTTAATGCAAAGCCAAGCAAGCTGAAAAATGGCCTTGAATAGGCTGAGATGAAAAAACAAAGAGGCTGTCAACATCAGAGAC
TCAGTGGGGGCAGGGAGCTTCAGTGGACTCCCCACAGACAGCAGTTCCTGGGCTTCTATGCCCAGACCCAGAGGCCTGAGCCCAAGATGTCAGGATG
GGTGGTCACTGTAAGTGCAGTGGATTGTAGCTCAGGGGTGAGACATCGCCTTTTTCTTGTCAGCTCTCCTTCCTGATTAGTGGCTCAAACAGCCTTCCG
AGACTCAAGGGGTTAACTGCAGATCCTCTAACTCCTCCACTTGCTAATTTTTAAACACAGCTGAGCTCTGAGCACCCCTCCCAAGGCTGTGAGAGGCC
AGAGGCCAGCACAGACTCTGCACCAGGTCTGCACAGCCTCCACCCCTACTGTTGTCAGGAATCTGCAGGCATCTAGAACAAAGGTGCCTCCATTATTT
CCCTCACCCAGCATATCCTGATACCTGAGCCAAGAGTTCACCCAGAGCTTTTGCCCACTTCATTGATGGTCTGGTGGGCTTTTGTTGACACATACCCGT
GACTCCCAAATGAACCTTTCAGGTTGGCTTAAAGCCGCTGGGACCTACACATGACAAAACCCAGACGGAAACAGTGGCTTACACTCCTGTGTCCCTAA
AGGTACCTGTTCCTAACAGTGGGAGTGCCAGAGCCTGGAAGCTGGAAGCCAAGGAGACAGGGACCTCCCTCCTGCTGACCCCCTGCCAGGCCACATC
CACCTAAGGGATTTGGGATCATACTCCATCTTCAACCACTGGGATCAAATGGAAAGCCCTCAGGAATTCTGAGAGACAGCCCCTATACCTATGTGCACT
GACACACATACACTGACAAATACGCATTCTGACACATATATGAACATGCACATATGTGTGCCCCACTCCCTGCCAGGAGGTGGGTTCTGTCCCATCTTTG
ACATGTTACTTCTTGTCCTTCCCTAGGCCCAGTGGCACTTTCTTTGTGATAGGTGCTGAGACCCCCCCGACTGGGCCCAGTGGCTGTTCCCAGGACCCA

GTCTCTGCTCTTATCTTAACCCAGCTCTAGCTCCCAGGACCCCAGCCTTTCGCAGGGAGAGAGATAGCTCTTTTGGTCTGGCACATGTTCCATGCCAAG
TAGTGGAAACAGCAGCTGCAGGGCAGCCTCAGACTTGACATCATTTAATCCTTGTGACAATCCTACAGGGAGGAAGACACTACACATATGTGCAGATG
CAGCAAGGAGCAGCTCAGCCACATGGAATGACTTGCTTGAGGTCACCAGGCACTTGACACAGCAGAGTTTAGCCCAGTCTCTGGAGAGACCAGCCTT
CCACATCCTGTCATGCTGGGGAGGGCTGCCCGCTTTTTGTCCCTGCTCCCCCAGTTCTAACCCCGGAAGAGCGATTGTCCCTGCTGTGCCTTCAGCCCA
GACAGAAGCAGGCTAGGAGAGGGGAGGAGAGGCAGGGCTGGCCCTCGGAAGGCTCACCTTCCTCAACAGTTCTCTGCTTGGGCAAAGGTCTTCCAA
GCAGCCAGGCTCTGAGGCTAGTCACTGTCATCGTTGGGTGAGGCAGCCTGGGAGTACCAGAAGTTGGAGCAGGTGGGAACCTTCCCCAGGGATGGGG
CTGGATAGGCAGGGGTGGGTGCTAAGTGGCAGGCCACAACCAGGCACCTGGGAGGGCCAAGCACCACACTGAGAACGCAGTGCTTCCCCTCCCCTC
CTAGGCTGCTCAGGAACCCATCAGTCTCCTGTAAACAAATGAGACTAAGTCTCTGGCACTCTAGGCCCAGGTGCAAAGGACAGCTGCCCCTGCAGCTA
CTCACTCTGCCCTGGCCCTCTCAGGAGGCTTGTGACCTCTGCACCTGCTGGCTTGTTTTGTTCCTAAAATACCCGGATCCTTGAGAATCTAGAAGACAC
CACTGCTAACAGATTTCCACATCATTCATTCTGTTCTTCCTGTGCAAGCTTCATGCTGGGACAGGGACCATAAAACAAGGTGACCCAAAACCTTCAGCA
GAATGCAGGTAGGCAGAGCAGAGGTCTCTGGGAGTGTCATTCCAAGGCAGTGTCTTCCTCCTCCCCTATGCTCCCCATTTCCCAAGAGTGGGCTTCTTC
CACCCCACTCAAGCACCTCTTCCCAAGGCTGACCTAAAGGCCTCAAGCTCTGCGGATAGGGGCAGTCCTGAGGTGGCATTGAGTCTTAGGGTTGGAGT
GGGAGATATCTGTGCAAAGGCTGATAGGAACGTCCTCCAAGGGCTGACTTCAAAGCTTGGCCTAAACCACTGCATCAAAGTCAGCAAGCTGAGCTGT
TTGCACAGAAAAGGAGGAGGAGCAGGCCCTTGAGCCACCTGTAGCTCTCCTTAGGGTCTCAGGCTTTCACACCAGGGCAGGCCTTGGGCCAGACTAC
CCAACTTCCAGGCCGGATGTGCCACCGGGGAGGAGGAGGTAACTGCCCCTCAAAACCCTGCAGCCCAGGGCTCCAGGATTCCAACCCAGGGACCTG
CAGGCTCCCCTGCTCTCCAGATCAGACCCCTACTCTCAGAGCCCTAGGCCCAGTGGGTGACAGCCTGTGAGACAGGCCTGTGTGGGGTGGCCCTAGC
CCCACCTCAGCTTCCAACCTGACCTTGCTGCATGACCTCTGGCCCAGCCCTCTCCACCTGGAGTGCCATCTCTCTTTGTGGGATGGGCCAGGTGGGCTG
TGTGTGGGGGAGGGCAAGTATATAGGTGTGGTATAGGTGGAAACTGCCACAGTAACCAGCAGCAGCTCAGAGCCGGGCCACCCAATTTGTATGCCAGC
CCCAGCTGGGCCTAGGCACGTGGTGTGTGGAAATGAACGAAGCAGGCAGCCCTATGTTTAGGAGGCAGGTCCAGAGCTCACCCAGGCACAGCACAA
GATGAGTGCCTGGCCTCAGTGAGCGAGCACTGGCTGCTATGAGGCTGTGGTGTGGAACCTTGACACTAGGGCCACTGGCCTGGCTGTGTGACCTCTAC
CTCATCACCCAGAGCAGGGCAACCAGAGGCAGAGCCTCAGTGTGGTGTTCTCCTGTGAAATAGTGATGGCACAGGCAGGGGCTGGGGCCCAGTGCCT
ACACAGAGGATCAGCAAAGGATGGCAGTTATTGAAACCTCTACGGGGAAGTTCCCCAGACACGAGAGGAAGACCCCCATGGAAGCTGTGTGTGTGAC
GTGCTTAGATTTTCCAGAGTCTTCATTTTCCTTGTCTAAACAGAGGTCATGGCGCCTCCCTCTCAGATCAAACAGCCCTGTATGAGTGGCTACAACCTTT
GGTGGGCTCAGTGCAGCCCCTCCTGCCCCCTTGCTTGGGAGCTGTCTGCAGAGGCCTAGGGAGCTGCGTGGGGTCTCTCCACCTCAGCCTGTATCCCC
ACCCCCACCAGGTCCCCTGCCTGACTCCAGCATACCCAGGCCCTAGAAGCACCAACTCCATGGCTGCAGCTGAGTGGAGTGTGTGCCTGCCCAAGCCT
AGAGGGGTGCTGACCCCTCCCTCGCACGAGGAGACATCAAAGGGAAGCAGGAAGCAGCGGCCTGGCTGACCCACAGCGGGTGGAGAAGTGCAGC

CTCACAACTGCTGGGGATTTTCCATCCCACCAGGATTTCCGCGGGACACCCCAAGTCCAGCCTTTGAGGCTCCTCAGGAACCAGAGTTTGGCCTGGAG
CCAGTTGGGGCAGAGGAGGGGGGCCAGGCTGGGTGGCTTAGGGGTGTGGTTAGGCCTGCTAAGGGGCTCCTCTTCTCAGTCTCCCCATGATTTGCTGG
TTACTGGCTTGAAATCTGGGCCCAGACCCATTCCAACACTGAACCCTGAGCTCCCTGAGACCTGGCTGGCAAGGACAGGTCCCAATTTTTACAGGGGC
CTGAGAGGAATGGAGAAGGGATCTCTGCCCCTTCCTTTCTCAGTCACACCTCCTTGACAGGGGGTCATCAGAGTGGGCTGTGGTAGGTGCACCAGGG
AAGGGACCATACAGAGGAGGCTGGTCTCAAGTCCTAGGGTGCCTGGGATGGCCCTATTGAAATGCTGCAGCAGCTCTTGTGCAGGCAGGGATCTGTAT
GGACAGCTGGGTGGACACAGGTGCCTACTGACCTCCCAGATCCTTCAGCAGAGCTCAGCCCTGAGTGACAGCCCAAGGCAGACCTGGCTACCTCAGC
CTCTGGCTCACAGCCCTCAGCCAGAGCCCTGAGAGGCCTGGGATCTCAAAGCTGCAAGGTGGGAGGCCAGAGGAGAGAAGCCCGGAGCTGGTAGTG
ATGCTGGCTGAGTGAAAATTGCACATTTCCCAACAGTGTGATGCTCCCTATCCAGCCTGGGCTTTTGGCAGACTTCTGTGCTTTGTGGAGGAAGCTGGC
TCAGATTCCTCCCCTCCCTGTGCAGTTTGTGTGTCAAGGTGTACTTGTCACACCCAACTCAGGATATCTTGCATCACTTTGTGGGTCCTTGTCCCCCAGA
GCTGGGACTGCTGAGTTTTAGCCCTAAGCTCTTGCTCCCATCTTAGCAAGTCAGGGCCCAGTGTTGTCCTTGCCTCTGCATCAGGATGGTAAGCTGGGC
ATGCTCCGCATCACGGAGACCTGCATGGACCTGGATTGGGTTGCCTCTATCTTCAGGCTGGTCACTGGGCAGACACAAACCAGGGCCTCTGGCCAGAG
GGGACAGTGGCTCTGAGTTACTTGTGCTGCTCTTTAGGGGTGAGCAGGGGCTGCTTGTGCATCATAGTGTATCACAGGGGATGCACATTTACAATTGAA
AGCTACTGGTCCTGGGTAGAAGTGGGGCCGAGTAGGACTTCCCAGCCTTGAACATGGGTTTGTCATCCAACTGGGACCCACACTTGATCCAGCGGCCA
TGCTCTGTGCAGCCTGGGCCTGAGGGCAGCCACGCTCATGTGAAAAGTGCTAGTAGCCAAGCTGCCATTCTAGTCACCACAGCTGTCCTTGCAGGAGC
ACATTGCCCAGGGCTGGGAGCTGTCATTTCCGTGACGGGAGTGAGACTCAACACTGGGTGAGGGTGGGGGTATCTGTTGGGAATTATTTCAAAACAAT
TGTGGAAACTTGGCAGTGGAGGGGCTCTGGGCATAGGTATCCACAATATCCTAGTCAGGGGTTGGGGTACCCTGGCTCTGCTGATAAGCCCAAGTGAG
CATACTCCTCCCCCAGCTTGGAACAGGTGGGACGGGCCAGGTGACACTAGTGTGGTCTGTGGTCTGGCCTCCCAAGCCAGTGGCAGTACTGGGGTGG
CAGACATTTGCTGTGTGCTATCCCAAGGCCTAAAGGTCACACAGCTAGGTTGGAGCAGGGTTTTGGGCAGGATGAGTAGGCATTGCTGTGCTCTCAGA
AAGGCGAAGCAAACCCCAGAAGCAAGGGGGCGAGACAGGGCAAAGGCCTACACTTTGCCATCAACTCAGGCCTACTCAATGCTGGCCCTGTCCTTCC
CCTGCCCCTGCCCCATCAGATGGTGCGAGCTGGCGGGGCTTAGAGTGAGCCCCCCTGACTCTTCTTGGTGTGGAGTCTGAGGCCCAGAGTGTACAGGC
CTCATCCGTGTGACCCAGCAGGAGGATGGGAGAGTGGGCCCAGAACTTGGTCAGGCTCATCCGTGGAGAACAAGTTCTTCCCTAGTCTAGTTGTGGTC
TGAGTCCTGGGTGAAGCAGGAGGCAGCCTGGGAAACAGCCTCGTTCTAGTAGGAATCGCATTCTCAGGAACGCTGACTGTGCAGGGCCCTTTCTCAC
TGCCACCTGGGAGGATGCCTGCCTGCTGGCCATTTCGGCGGACTGTCAGAGCACCCTCCCTGGGGTCCCAGACCGAAGGCCTGAAGTCCACATGGGA
CCCCTGAACTTCTGGTACCTGTGTGGGCTTGTCCCCTCAACCCTGAGTGGAATGGCAACAGCCAGCTGCAACCCTCCTAGCTTCCTGTCATGTGCTGTA
TGGAAGGGCCAAGAGCTGTAGTCATCTGCAGATCCTCCCACTGCACCTGTAGGACTAGCGTCTCCTGTCTGGATGTGAGCAACTTGCTGGTGGCCTGT
GAGATGGGAGTTGCACTGGGCTTCTGGATAAGGGCTTCTCGTTCAGGTGACAATTCTATGTGAAGGAGACCCATGGAAACACATCTATCCCCTGCCCAT

GTACCAGGCAGAGATGGTACCTGCCCATGTACCCTCCATGACATGGCTGGGTCCCTGAAACATCAGACAGATCGATACTCTGTGTGCATGTTTGTGGGC
AAGACACAGTGGCACGTGTGCCCTGGAGTTTGGTAATGGGAGGACTGAAGTAGTGCCTCCGTGTGTGAGCTGTGCCAGCCATCCCCACGAAGGGGGC
ATTCCATGCAGATGGGGATCCTCTCCAGGCAGCTTCTTCCACACCTGATTAGCTCTGACCATCAGACCCCCCGTGCCAAAAGGGCCCCTTCCCTGGAAT
CTGTAATTCTCACAGGCTGTCCCATACATTTTAAATCCAGGAAATGTGCTCACCCATGAGACAGGGATGGCAAGAAGATATGAATGAAAAGCAACCAGT
CCGTAAAATTGGAAGCCATTTCACTCCTTACCAGGTACAAAGATAACATGCTGCATGGATCAGGGACACAAACTTACCTACCTTATGATGTAGTGAAAG
AATCTAAAACTTTTGTCTCAGCAGACTCCCTGGAGGAAGCCAATAACAGGAGGCCTTGACTAGGAGACCTCAAGCCTTGGAATGTGCCCAGACCAGT
GGGGAGGGAAGGCTGGGAGGCAGTCCTCCCTTACCCCTTTTCTCCCAGGCAGACCTACCCTGGGTTCTCTCCAGGCCCCTCCGCCTGAGCAAGTGCTC
AGTTGGCCACTAGCTCCCTGCACATCCAACAAGCCTTTCTTTCCTGTTCCTTCTTCAAGGAAGGCTTAGCCTCAAATCCAGGGCCACAGACAAAGGAC
AGTCACTCTCCTCTGCCAGAAAGTCATTTATGGGACATGGAGAAAGCATCAAATATATTGCTTTTCCAACACCTAACAGTGGAATAGGAGGAATAGAAG
GAGAACTCAGCCAGAAAGTTCTGGAGCATAGTCTTTTTGCCAAAAGAACACGTTTTCAAATGAAAGAGGTGGTGCTGGGGCACTGAGGAATCAGGGG
GATGGACCCCACTGGAGCTTGTCTCATGCTGGCAGCAAGTCCCAGGCCTCAAACACATACCTGGGCCACCTCAGGAGAGAGACTGCAGGGAAGAAAT
GAGTTGCAATGCTCCAGCCTAACCAACTCCCCTTTTGTTAATCCAATCCCAGAAGGTACTGGGTAGGGTCAGTTAACAGACAGCTGCCTGTCTCCTGGC
AGAGTCTGACCAAGCCCCAGTGGGCGCTTCTCTTCCTAAGTCCTCTGGTGTACCCCATTCTTCAAGGGAAACGGTCCCCTTAAGAATCTATGTGAAAG
GGTTCTGAAATTGCTGTACCAGTTAATGAATTATTTACTCTAAACAATGTCTTGATAAAGGGGGAGGATGAAGTTAGCTGATGTTGACTAAGGAGAGAC
GGAGTTGCCAAGGAAATTCTTCTAAATGAAAAAAAAAAAAAAAGTAAAAGCAGTCAGCTGGTTTTGTCTGACTATGAAACGTGTCACAGAGCATGCAC
CTTTGGCCATCTTTTGCATTTATGATTTGGAAGAGAGATGTAGCAGTGAGTGTGGGGCCAGGGAGGGGAATCCAACAAAGCCATTCTGCAAGCTAGTG
GCTGGGATTTTGGGGTATAGAGTAAGAGCAGCCAGCCTTAGAGGCCCATGTCATCTGGGCTGCCTCAGAGCTAAAGCTAGGTAACCAGAGGGAGTAAG
GAAAGGCCTGAGTCCCAGGCTAGGCTTTTCACAGTTGTATTGAGATGGGAAAGTAAGGGATGTTCCTTGCAGCAGATGCAGGGCCCAGTTGTCTAGAG
CAGTATTTGAAATGAGTTGGCATTTCAGGGCAGTGTTTGTGATGCCAGTCAGTAGGTAGGGGCTTCTACCTCTTTTTTAGAAATGTTTACTCTGAACTAA
GCTCTATGCTTTAACAAGTTATGTTTTAATAGGAAAACTGTCATTGATGGAATCATATGTAATTCCTACATTTTTCCAGTTACCCATTCCTGGGGAAAGCTT
AGCCATTGGACAAACCACCCAGAGCCCCAGGAACCTGCATTTGCAGACAGCAGTGGCAGGCACAGTGGCCTAGTGTTGAGCGGGGGAAACCTGGCC
TCTATGTGTCTGGTTTTGCAAACTAAAACGCCATTGCCCATACTCAGGCTGACAGGTTCTGGAGGTGCCATGGACTCCTGCTCTGTATCTCACCTCTAGT
TCCATCACAATGCATGAGCTGTCAGTGGGAGCCTTTTAATGCAGATGCTAATACCCATTCCTAATGCTAGCCCATCCCCACACCCTCCTGAGTTGGTGCG
ATATATCCTGGGAACAAGAAAATCTGAAGGCTCCTGGTCCTCAGGCTAGCAAGAGCCTTGCCTGTAAGAAGTGGGAGAAGGTCACCTCTCTCAGGAG
GCAGAACTAAGGCTGCACAGCACAAGGGAGATGTCTGAATGCACAGTCTGGGTGGCAGAAGGACTCCTGTCCTTTCTTCCACACAGACCTGAGCACC
TCTCCAGGTGCCACCATTTTCTGTCTGGGAACATCTGCTATCCCCAAGCCTCAGTTTCCTGGTCTACAAAACTGAGATGGTCTCTTCTATACAAGGCCCC

AGGAGGATCACTGCAAGTCACTGCATTTCATGGTGCCCAGATTTCCCTGCATTTCTAGAATGTCAACAGAGATGAGTGGGCTCAAGGGGAAGAGATTC
TTTATGACTAGAGATATTCTGGTCAACTTTGGACCCCAGCTTCTATGGACAATAGCATGTGGACATGAGCCCTGGGGAAGCAGGGATGACTGAGCCTGA
AGAAGAGCTCTCAAGCCTTACACTCTCCAGACCAGCCTTGGGTTGCCTGAACCCCAGCATAGCCTTATGGAACCCCTGCCAGGGTCCTAGCACCTGGT
TTGACCTGGGCACAAGTACTCACTCACTCACTCACTCACTCACTCACTCGTCAAACATTCACTGAGGGTCCGTGACAGGCTCCATATGGATTAAGCACT
GAAGATAAAACACTGAATAGAAACAGCCAGCCTATACCCTCATGCTCCTTGAATGTGATAGAAGGAGCCAGCCAGTAAACCCTTTACCACAAATAAAC
ACACAGTTACACATTAAGAATGGGAGGGGAGGTATGTGATTTTTTACAGCCCAGTCTTAAGCTTTAGAAATTGGCTCCTCTGAGGAAGGGACATTTCAG
CTAAGAACCACAGGATAAGACAGTCCTGGCCTGGGGAAGGGGAGAGAAGGTCAGGAGAGAGCTTGATATGTTTAATACTGAAAGAATATTTGATGAG
CTGGGACATCTCCTGCAGATCCACGGCTCCCTAGCTTTGTGACCCTGGGCCCTAGACTCAATCCCTCAACCCTTAGGAGATTATCTATAAAACAGGAGT
GATAATATTCTTGTGTTAAAAGCTGCTGTGAGGATTAGTGAAATGATGTCTATATTTGCCTGGCAAACAGGAAGTGTAAGTAGTAGTATCATTTGAAGATT
ATTCTTCAGCCTAAACGTGTGTTCATTATGGCCGGTGTGTTTTCTCCCCACCTAGATGGTAGTGCTGGGCCTCCTTTGGAGTACCTTGATGTTTGGGGAG
CATGGATGATATGACCATAACCTGAGAAGGCAGGGACTGTACCCATTCTGGTCCTCCCAGGTCCCAGGGCCCCCACAGTGCCCTGGTGTGTGATCAGT
GCCCACAATACTGAATAGAGGAACAGACACATGGGAAGCAACGGCAGCCTGGTCTTTAAGGGTGAGGGAGCACCTTCTCCTGAGAGAGGGAGAGAT
AGGTTGGGGGAGGTTGGGTGGGGGTGGGGAGAGCGAGTGAGCAAGCAAGCTAGAAACTTCCTGAAAAGGGAAGTTACTCTAGAAAAGGGTAAGTTC
CTTCCAGGCATCATGGGCTGGCTTAACCTGGACCAGTTTGACTTTACTAAGTCCCATTTCTCATCTAGGGAGCCTGAGGCCATTAAGGTTATCTGGTCCC
AAAATCCCCTAGAGCTGTCACAGGGATCCCCATTCTTTGCCTTTAGTCACCCCAGCCCCAAGAGCCATCCTCCACATGGAGTGAGCAGGGAGCTACAA
GGAAAAGACGGTCACAGTTGGAGAGAGACAGAGAACCAGGTGGGCTGGTAGGACAGGAAGGTATAGGGAGAAAGTGAAGCTCTCTCCTGGGGAGG
CAGGAATACTCTCCTGCTTCCTTCTACCCACTCTTCATAGAACACAAACACTGCAAAAATGACTTTTTCCAAACCCTCTTAGCATTCAGAAGTAGAATT
GCTATGTAAGAATTTTTTCATAGGTGCTTTTTTGGGGGGCATCTATTTAAAAAATAACCACATCATTAGTGAAATCTTCCAAAGTCTCTTTTTGGAGCCCA
CATTTCACTAATGTCCACATCTGAGGAGGCTTTCATAATAAAAGAACACATACCTATTTCACCTATGATGGTCAGTGCAATCTTGAAAACACTAGTAAAT
AGTGTCCATCAGCTTTAAAAATACAAACCCAAATTGGGTCATACCAAAATGCAAGGATACACACAATAACCTGGGTGAATCTTCAGGGAATTGTGCTGA
GTGAAAAGAGCCGATCCCAAAAAGGTGATTTGCTATGTGGTTCCATTTATATAATTTTCTTGGAATGATAAAGAAATGAAGAATAGGTTAGTAGTTGTCA
GGGGCTAAGGACAGGGTAGGGTGGGAGGGAATTGGGGGTAGCTATAAAAGGGGGACATGAGGGATTCTTGAAGAGTTAGAAATGTCCTGTAGTGAGA
CTATCAATGCAATATCCTGGTCAGAATATTGTACTACAGTTTTGCAAGATGTTACTATCGGGGGAGACTAGGTAAAGGAGTATGTAAGATTTCTCTGTGTT
GTTTCTTAGAACTGCATGAGAAATGTACAATTATCTCAAAATAAAAATTTATTTTAAAAAATGCCCAAATTAAAAAAAAAATCAGTAGTCCTCCTAAAAAT
ACCCAGAAATGAATTTTAGAATGTCCAAAATCCATACAAAGATCTAAAAAAAATATGGAAGGATGTTGCAATGGATTTGAACATGTGGGTAAACTGCTTC
TATTTGTGGCTGGAAAGACTTTTTTTTTGAGGTGGGGAAGGCAATTCTTCATCAGTTAATCTAAATTGAATGAAGTTTCAATAACAAATGCCAATGGGAT

ATGAATATACACACAGATAGACAACTTTTTTTTTTTTTTTTTTTTAATTAGGCAAGCTGAGGCCAGGTGCATTGGCTCACGCCCGTAATCCCAGCACTTT
AGGAGGCTGAGGCGGGCAGATCACTTGAGGTCAGGAGTTTGAGACCAGCCTGGCCAACATGGTGAAACCCTGTCTCTAATAAAAAAAAAAAATTAGCC
AGTCATGGTGGCAGATGTCTGTAGTCCCAGCTACTAGGGAGGCTAAGGCAGGAGAATTGCTTGAACCCAGGAGGTAGAGGTTGCAGTAAGCCGAGAC
TGTGCCACTGCATTCCCGCCTAGGTGACAAAGTGAGACTCTGCCTCAAAAAAAAATAAAAATAAAAAAAAAAATTAGGCGTGCTAGTTCTAAGTTTATATG
AAAAAAATGCATGTCCTATAAAATTCAGAAGTAGCAGCAGTCTGGCCCCTTGTGCTCCTCTAGCCCTCAACTGGCCTAACAGGCCCTAGTGTGCAGCA
GGGACTATTTTCCCATCATCACTGTCCACAGCATCTGGACAAGAATACAGAGAACGGCTGATTTAAAAAAAATCTAATCTGGGATCCTATGCATCCTAAA
TAATCTTATGAGGATGCACTTGTATTTTTTCTGATGCTGATACTATTCATTCCAACTCGCCTCCCCATCAGCTTTGTCAGGCACTGTGAATTGCATGGTAC
ACATGATTACAATGGATGGAATGGCCATCATGAGGTATAGCAATACATTTAAGTAACACTGTGTCACTACTATAGGACAGGCACTTTTCTGCATACTTTAT
ATTATTAATTTGTGTAATCCTCCTAACAACCCTCTGAGGAAGGTACTCTTATGATTCCCATTTTACAAATAAAGAAACTGAGACTAAGTGACTCACTCAA
GGTGTAGCAGGTAGAAAGTAGTGGAATTAGGATATGAGTTCAGACTCCAGACCCTGTTATTAGCCCCTGTCTTTGGCTATGGATTAGTAAAGGAGAGCA
CAGCCAAGGGTGGGACAGGCAGCTGTAGTCCTCTGGACCAGCTTCACATGGATGGGGCTGCATTGTTACAGGTGGCCTAACTCAGACATGGAAACTG
AGTCGCTGCTGCCCAACTCATGCCATGAAACCATTTTGGCCTGCACAGCTTATAACAGCACTCTAGCTCTAGCTATGACACTTTATGGTGTTAGTGGGTT
TTTTTGGTGGTTGGTATGGAGGAGGGGCTGGGCTACAAGTTTTAGATTATGTTTCTTATTGCGGGTTTTTAAAAATTCTTGAATTAGGCCTATTTGCTCTT
TTGTTCTCTATTGAGTTAGCAAATTCAGTTTTTCCAAGATGCCCTGAAAGTATGATCATACATGTATACGAAATGGTCCTTGGGTCCAGCTGGCAACTCC
TGTGCACCCCCGACCTGGATTCTGCCTTCCTTGAAGTGAGCCTTGGAAGAAACAAGTGCTCATGCTCCCTGACATTATTAGCTGCCACGATGAAGCCTC
AGTCCCAAACACCCCAGCTTCCTGTCTAACTCAAGAGCTAATCCTGTGTTTAAATCTTAAGAGTGTTTAGATCTTAGGCCATGCCATGAATTCCCCTGGT
TGACTGCATTTGTGAAAATGAGCTTCCCATTATTGGCTCTTAAGTAATGGAGATGAGGGGGAGTCCTGCTACACAGATAAGTGCATCATAAAGTTACATA
ATTAAAATAACTGAGCTCTGGCCTATCAACAGGCCCATCAGCAGTCCAGAATAGAGTCTAGAAATGGACACAAATTAAATGAGAGTTTGAAATATGATA
AAGGTAGCAGTTGAAATTAGTAGAGAGATGATGATGTACTCAAATAAATAGTGTTGGACAACTGGACAAATATATGGGAAACCAAATTGGATTTAATTA
CTCTGTCAAAATCCCGATAAAGCAAGGATTTAAATGTAAAAAATGAAATAAAAAATGATAGAAGAAAACATGGAAGAATTTATTAAAACAATTTCAGGA
TGAGAGGTTATTTCAGAATTTAAGATAAGGACCTCAAACCATAAAAGACTGACACAGCTGACTATGTGAAATTAAAAAAATTCTGCCACCAGAAAAAT
ATTATAGAAAAAGCCAAAACACCTAAGGAAAATGAGGATAAAATATTTTCAATGAATATGACAAAGCAAGTTTCTACAAATCAGTAAGACAAGGTATTT
TACAAAGTCCAAAGGGTATGAAGAAATAGTTCACAAGAAAGAAAAACAAATGATTTTAAGCATTTGGAAAGATAAAAACCTCATTTCCAACAAAAGA
AATGTAAATTAAAGCTCCAGTGTACCATTTCTCATCTATGAAATGAATGAAGATAAAAACCTGAGTATACACTTGGTAAGGGTGGGGGAAGAAAGTGCA
TTTGTGCAGTCAAAGTGATGTAAATGGTACAGCCCTTATTTGGCAGTATCTATCACAACATAAATGAACATTTTAATCCTGCTGGAAATGGGGGGAAAG
GGAGGATCTCTTAAAGTAAATCCACTGAAAACGGAGACCAAAATTTTACAATAGAGATATTTGATTGGGAAGTTAATTTGAAAAATAACAGTAGTTAAA

ATATTATTTTATCTCATATGCAATAACAGTTGTTAGAGTTACAATTATGTACACGTTTTAGAAAAAAACAAATCCAGAGGGGATTTTGGTTTGTCTTTTGT
AAAGCATTATATGGTAACATCTGAGGGCATCTTTTATACTTGGCAGAGGATGTTGGCAGCACTTCCACAGAGAGGATCATTTTTTGTAGAAGTATGGCAT
GGTTTCACTGCTTGACCAAGAGGCCACCTGCATCAATTATCATCTCCCCTGCTGTCCTCTGAAATGCTTATCTAGGCATCGATCTTGGTTTCCTCTTCAAC
TGATTAACTAACTGGTCTACTCTGCCCAGTCTCTATTTGAGAAATCATTTGACATCATTTTCTTTAACTTGATGAAATCTCCTATTTTGACAAGATTTACAG
TTCCTCTTTATAAGTTCCTCTTTACTTTGTGTGTGCTTTATATTGTCTTTGGTTGACAAGAGACAGCCAAAGAGGTCAGTCAGCAGAGATACAAGTAGGG
ATAAGATGGATAGGGAGAAACAACAGTGTTCAATAGGGAGGGATGTTTCAGGGAAACTAATTTCATAAGTGGTCATTTCAGTAGTGAATATATTTGAAT
GGTTAATGGCTGCTGTTTTTTAGCTGCCCTACATATCCATCAACAGAGGATCTGTTCCATAAATCATGGCACAATGAAACTCTGCAGCTGTTAATGAAAA
GGAGATTTGCTAGATGTCTTAATACAGATCCATCTCCAGAACATACCAGTGTCTTCCTTTTAAGTGGTGCACACTGGGGCTGAACACTTACTAAGAAAT
GAAAGGGCTCCCCAGTCAAAGCTGACAGGTGTCATTACAACATGACCCCTGTGGTAGATCCCAGCACAATACACTGGCCATCACTCCATTCTCTTCAAA
TGTCCTAGGGTTGCTTCATACAGCAGACCATCGTACCCAAAGCCACTATGCATATGACTTGGTTATCTCCTGCCAGGCTGTAGTCCAGTTAGTGCTCCCT
TGGAAATACTCAAGACCTTAATGAACTATCTATCTGTGAATCTGTGAAGCTACCCATTTCTTAGAGGCCTCTTTGGAGAGCTGGCTGTTGAATACCCAGT
ATGATGGTCCTTAGCCTTCTTAGACCTTTCCTGTACCACTACCTCTATGCCTTTTGCCAAGTCCAAGTGTTCACCAGAAATGGAACTGATGCCAGAAAG
ACTACAGCTAACAATCTGAGCAGAACAGTGAGCATGTCCTATCTGGGAAAAGCCCAGCCCATTGGGCTAAATCTCTAGCTGAACACAGAGACTATGCT
CCTTCCAATTATACTGGAAGCCCAACCATGAGAAAACAGCACTTCAACTACACTGTTCAGAGCCATCACCTGTTCACACGTGTGTCTGTATACATGTAA
ACACACATTTTGCAATAAAAATGCTTCTCAGATGTACTGCACCATAATAAATCAGCATTGTATGTACAGCTCAAAAAGAAGTATGGCATACCTATGGAAG
AAGTCAAAGGCAAAGTGTAGAAAAATACATACAGTATGCTACCATTTGTATAAAAAGGAGCCGTATCTACTAAGTACATGCACACACAGACACACGTAT
ATTTGTTTATATAAAATACGTCTGGAAAGATGCAATAGGAAAGTGTTGAGTGGTAACCTTTAGGGAAGGAAACTTGGGAATTAGGGTTGGGTGGGGAG
AGTGTTATTTTTCACCCCTTTTGTACTCTGAATTGGTTTTACCATGTGTGTAAAGACTGTTGTATGATTTTTGTAGCACAACCCTTGAATACATAAAGCAC
GATTGGCTCCCCAGAAGGCAGGTGTTGGCCAGGCAGTGGATGCACGGTAGGGCAGTGTGCAGCCCTGCATGCACGTGGCTGGTATGCCCTGCGGGCA
GCAGCTCCCCGCACACAGTGCTGTGAGTCTCTGGTTCATGTCACTCTGTGGAGCAGGGGCTGCTCCAGCACAAAGAGGTTTTTGACAGGGAAAGGCA
GAGCCAGAGTCAGGGCAGGCCCTGGGCGGGCTCCTAGAGAGCCCAACCACCCTCTAATAAGGGGTGCTCTCCACCCTATCTGAGGAGCAATTGGGAT
CTCCTAGGAAGTGAAACTGTTACGTATGTCTTATTACAGAATTTCCAGTGGAGTGATTCTGGAAGAAATGTGTATGGCATGTTGTGACTGCTATGTCTCA
TGAAATATTTACGTTTTTATGTAACTTTTTTCCATTTTCAGATCATCTTTTATTTTAACGCAAAATGACAATATATTTTTCAATTAATTTGCCCAATATCTGTG
TTTAAATGTTTTCTAATGCACACACGTGTTTTAAACTCATTAAAAAAATATGACTTAGATCCCATCCCACTTTCATGTTTTATATTGCCAAAACTCTGCTTT
TGGGAGAGGTGAGTTATCTTCACTTCTGAAACAGTACTGCTTGGGAGCTGGTTTGGCCCTGGCTATTCTGGGGAAATGACACAATATTGACCTAGAGG
GGCCCTAATCTTTCCCCGCTAGAGCACTACCTCTAATAGGGTGAAGGATGGGAGAAAGAGGCTAGTGAACCCACCTTCAGGTCTGTCCACTCAGGACA

TGCCCACACTAGCCTCTCCAGGGGCTCTGCCCTTGGCATCCCCGTCCCCCACCTGGGCTCTGGCAGAGAAGTCAGGCGGGAAGAAGCCGGCTGAGTG
CCCGCCACTGTAAGAGGAAACGAAGAGAAACAGCTCCAGTCAGCCCCAGAAGCGAGGCCTGGAAAAACTCTGCAGCTGGAGCTAACACAAAGGAC
TTTTCAGACCCTGCAGCCAAGGTGCCCAGTGAGCTTTTTTTTTTTTTTTTTTTTTTTTTTTAAAAAACAGCTATTAGGGTGACAAGCCTTTCCAAAGGC
CAGCAGTTGGCGAGAGTTTCCAGGAAGCTGGTCTGGCACTGGGCTGGGATTGGAGCAACTTTAACGAGTTAAGTTCAGAAGGAAGGAGTCATCAGGA
AAGGTTTAAGGTTCAACAAACTTTTCAAATTATTTTGCAAAAAGTTCTGGCTTTCCAATTCAAAGGCATGTTTCGCTGTGACTGTTTTAAGCCAGAGCC
TATTTAAAAGGCAACACAACTAGCATGCTTCAGATTCACTCACTCGAGGTATTTGTCTAAATCCTCCGAATGGGCTTTGCAAGTGCCATCTAGTGCCTAG
TAACACTGGGGCCATCTTACACATCTCGACAGGCAGCCCTCCTCCCCACGTGCCTCCCCCCAGCCTCTGTGGTCGTGTGCGCGCGGGCGCGCACACAC
ACACGCACGCATGCACCTTCCCTAAGCAGGAAGCTGCTCTTTGCCAAGAGCGAATGGAATTCAGATTTCCAGAGGGTGTCCTTGGCCTCCAGCTCTAC
GGCTGCATGATGAAGAAAGGTCCTAGTGTGTGTGCCGGGGGCTGGGAGAGAGGGGCCTCCAGCTCTGCTCACATCTATCTGGGCCCCAAGAAAAACC
CACAACTGGCTGTGCTCTCCCCAGCCACACCCAGCCTTGGTCACATTCCTTCCCTTCCTGAGCCCACACTGGGTGGCCCCGGCAAACCAGCTTCCACA
CCTCCCTCCCCACCGGCAGGGAGACCTAGCCCAAGCCCCCATGGGGCAGCCAGCAAGTACAAATGAGGGGCTGCACCCCAGGCCCAAAGAGCCAGA
AAGCACGGAGGGCCCTCCCCCCACCCCACCCCCAATGCTGGGCATCCCCTTGTGGCTGCTGTCACTCATGGGGAAAGAGACACACTCCCTTGTCTAGC
ACCCGGTGAGCTGAGCTCCCATCCCTTCCTGTGCCTGCTTTCAGGAAGTCCCTCTTCATATCCAACCTTGACTCTCCTGGCTGTGGTGCTTGTGGGCCT
GGGATTGAAGGGCCATCACTGCCCTTTGCACCAGAGCTTCCCAACATACCAGCAAGAAGGGACTGTACCCCTTCCCCTGTCCAGTCTGGGCCTTCCAA
GCCCTGAGATTCAGCAACCTCAGGAATTAAACATTTATAGGAAAACACCTATGCAGACAAGGGACATGAGTCAGGGTGGTCATCAGCAGTAGCAAGGT
GGGTAGGCTGGTCACGTTTAGCTTCTCTTTAGCCCAAGTAATAGGGTCTTCCACCTGCATGGCCAGGGAGGGGATAGGCTGAGAGCAATCTTTCTACAG
GCTGTCAGCATCAACAAGTGGTTGTGATGAGCAGGAGTCTGTATGGCTTTTCAGGTGTTCCGGCTCAAGGAGGCCCAACACCAAGTTGGAACCTCCA
GGCCTGGTGCCTAGGCAGAAGGTGGGCTAAACAAAGGCCAAGGGGAGCTCTGCTCTGTCACTTCCCTTTGTTTGCTAATGAGACTGTTTCCAAAAGCT
GGCCTGGTAGCCTGTGGGATTGTTTCTCTCAAGATTTCTTCTAGGGGAGAAGGTGGCTGAGGCTTTGCAGTCTGCATCACCCAGTCCCCAGAATCTACA
GGGGACTCAGCTTGCCTAGTCACAGTTCGGCTTTTGGCCTTGGCTCCAGGGCCCATTCTGATCCTCGTGCTTGGGTCAGGGATGGTCAGCACCCCTGC
CCTGATGCTGCCTGCTGGGGTCTCCTGCTGAGTACCGTGAAGGCCTACTGTGGCCTGAGAAGCCAACTTCACTGTCCCTTTTGGCCAGCCCTAGACCCT
ATATTTGTTCAAGTAACACATTGATTCACTGGCTTCTCCTTTAGGCAAGGCCTGCAGGGGACTCAGCTGGGGTTCATGATTCCTTGCCACCTGTGGTGG
GCCTGCATGGCCAGCCCAAGGCCTCAAGTGCCACATACTGCTCCCCTGACAGTCAGGTGGCTAGGGTATGACACTCAAGAAAATGCCCAGGCAGAAC
CCAGAGTTGGGGTCACTTGGTCCAGCCCTTCTGAAGGGGACTGGTTGGATCTGTTGCGGGGAGGAGACAGGCCATGCTGGACCAGCAAAAAAGCATA
GAACTTGGAGTCTGGAACCTGACATCTGGCCTTGGCTCTGCCATCAGCTGGCTGTGTCCACTGGGCAAGGTCCTATCATATCTGGTCTCAGTGCTTCCA
TTTACCCAGAGTCCTTCCATCACCTGACACCTACAGGGATGCTTTCCCAATCAAAATTCAGGCCATGTGGTTTGATAAGGCCACCTTCCAATGTGGAAG

ACAGGCTGGTAATGGAGAGGCCTGGGAGCACAGAGCTCCTAGGCACTGCCATGGAATGTGGGGACCTTAGGGAAGGATATCTCAATTGAAGCTCTTCA
GAGCCCCTGGGGTGGACTGTTCTGTACTCTCATCCCCTAACCCATACCAGCCTGAGCTCAATCATATCAGCCTGTGAAGGCTGCCCCACCACACTGCTT
CTGCACCCACCACACCAGGCCCCCTTGAGCTCCCACCTGGAGCCCACACACCCAGCTGGTGGGGTTCGTGGGGCAGTTCAAAAAGAAAAGCAGAAA
AAAGCATTTGAGTATTTTGGACTGGAAGGAATTCAATTTAACTTAACAAATCAGGACATGAACCTAAAGAAAACACTGAAAAACAAGCCCAACTTTGA
AAGGCCCAAAGAGGGAGGATTAGGTGGGAGAAGAAAGGTCCCCTGTGGGCAGAAGACAGCTGCTCAGTCCAGACCAGGGACCAGGGGAGGATACT
GGGCTTGAGTGCAGACCGGGCCAGGGTAGGGAGACAAGCCCAACATACACTCGAGGACTTGAAGTCAGGGGTGCTAGCTGACTTTTTAGCCCACTCT
TTCTACCCCAGATACAGAGACTAGCTGCCTGAGGAAGCATAGCGGGGCCCACAGGCTGGCTGCCTGGTGCTGCCCACCTCCTCTCCAGGGCTTGGCTC
CCTGGGGCAGAAGGCTCAGACCTATAACCACAGTGGGGTTGCAGGGATTTATTTGGGGGAAGAACCCTGACTGGGTCAGAATGATTATGTGCATGCAT
GTATGTGTATGTAGCTCCTTGTTCTCTGAAAAGCCTTGCCTACCACCCTACTTCCCAAGTACCCTCTATTGCCTTGCAAAGTCACCCCTGCTCTCTGCCT
CAGTTTCCCCAGCAGGGCACCACAGGACACTGCTGTGGCAAGGTTTATACTTAGGGTGCAGTGCAGTCTGATCTTGTATAAATGCTCAGTGCCAAAGA
CTTAGGAGATAAGTCAGGCAGAGTCAAAATTAACCTTTGAAAAATCCCTTGAATTGGCCCTAAAAATAATCCCTTTCTCAGATCTTTGTGTATATCCCCT
GCTGGGTTGCTCTTCTGCCTGCTAAAGTGGGAGAATCTCTGGGTGCACATCCAGAGTACCCTGGGAGAGGCATGCCAGGTGTTCCTGTACCTTTTTCA
GGATATTCCCAGTGTCATCTTCAGAGGGAGCCTCTGAGGCTGCAGCCACACATGGGAAAAGATTACTTATGGAGAAGATCAGAGGCTAAGGGGCTGGG
GATGGTGGGGACATCGGATTTTGAGGTCAGACCTGGACTCCTGTCTACCTCTGTCTCTCTACCTTTTATTTTTCTTTCCTTTTCTCCTAACAAGGCCCTAT
TTCTAAGATGGCACCTCAGGCCTCCCATGGGACCAACCAGGCCTGACAGACTAACTGCTCAGACTAACTGCCAGACTTCCTCCTGCTAGCCAGTCACT
ACCAAGGATGTGTGCCTCAGATACAGCCCCTGCCCAGGACACCTACGTTGTCCCTAACAGGGCCCACATATCTAAAGCATGAATGCCTAGCCAGGGGC
TCTGGGGCACCATAAGAAGGCATGAAGCTGGGCCTGCCAATTTGCATCCTGGGAGGGCAGGTCAGCCTTGTCCAGGCAATCCAGGAGGCTTCCCAGT
GGCCCACTGGCCAGTGAGGACCTTTGACCTCCTTGGCTGGTGGCACCAGGTTTATGATGCACATAGAGGCTGAACTTGAAGAATTCTTATTGCAGAGG
GCAGCCACAAGAGCCACTGGCCAGTGCCCAGATAATATCTGCAGACTGTTCCCTCCTGCTCCATGGGCCACAGAATCCCTTTGTCTTGTTACTGCTACA
CTGCCTTCCTACCAATCTTCAGGGTTCATCCTGGCCTCTGCCATGCATATCCAGGCTCCCCCAGAGATTGTGTGTCTGGGAATGAGCCCTGGCTTCTGCT
CCTATCTCCCAGTGATACTGGCAAGTCCCTATCCTTCTTGAGGCTTCAATTTCCCCTTCTGTGAGCTGGGAGGACAGGCCCACTAGATGCTCTCTGAGG
GACCTACCAAGGATGTGGGCTGTGTGTCTCAGCCAACAGCAGTATCTCCTAACCCCAACACAAGAAATACACATAGTAGGTGTCTGGTTAATGCTCTTG
GAATTTCAGCCTATACTAACCATCTGAAAGCCACTGAGTCTGAACTCTGCTACCATATTTGAGATTATCTCTCCATTGGGCACCTATGGGTTGCCTAGAG
ACAAGCTACCATGGTCCTCTTCCCTCGCCCGTCATGTGCAGGGTCAAAGGCAGGTGAGCTACACAGCTCGCCCTGCTCTTCACTCTGCTTTAATGTGGG
TGCTTGCCTTGGCAGATGGGCCCGACAGTGATAAGAACAAATGACAGATGCATACTGGGGCACCTGTGACTGCCATGAGACACAGTCCACTGATTCTG
CCTCCAACCCCCTCCCCAGAGCAGAATAGTGAGTGCTAGGTTTCCGCCTGTACCTCCACGGACCCCAACAGCTCCTGAACCCCATTCCGAGATTTGA

GGTTGTGACTTGGGGACCTCTAGACTTCCCTTCCCTCCACCTGACTCCAGACTGTCCTGGAGCTTGGGACTCTCAGGCCTCTAGGCACATCTTATTAAG
GACAGCAGCTGACAGCCATATGTTGTGACCCCATTTGTATCCCATGGTAACTCATGACCTGGCAGTCTCAAAAGCTCTCAGGCCAAATAAGGAATGTCC
CCACAGGATCTCCCATCACAAAGAGACATTCTATGCTGGCTAATATTTTCAGGCATTTCCCCGAGTCAGGTATGTGGCTGGATGCCTAACACCATCTCCT
CAAGGCCTTACACTAATACTGTGAGATATACTCCCAATGTATACAAGAGGAAACTGAGGCTGATAGAGATTAGGTGACTGCCCCAGTGTCCTGTGGGCA
GGATGAAATGACCCAAGCTGGCCAGGGCTCCCTGACAGACCTTGTCCCTGGATTCAGCGAACATACATCAAACCATTAGCTGACAAGGAATGAATCTG
ACAAGCTGTTCATGACCACCACACTGAAAACTACAGGGCAAGGTAAAAGATATGAAAGTCAACCTAAATAAACAAGGGACACATGATGCCATGTTTCA
GGAGCCCCAAAACTATAAGGATACCCATTCTCCCATGCCCATCTAGAGGCTGCTGCGCCTAGAGGAGGCTTTCCATGGCAACTGGCATATGTATGGTGA
GGAGAAACCCTGCAAAGACAGCAAAGCCAGTGATGGGGAAGGAGAAGCAGCCAGAGCCAGCAGGACCAGCTGTCAAAACCTGTTAGCAAACTACA
GTGAGAAGGAAGCAAGGTCCTGGCCACAAAGAACAACTTGAATCTGACCAGTGGCACCCAATAGGGTCCAGAAACAGAGCCACACGTAGCCAGTGA
GCTGATTGTAAAGACACTGATACGGCAGGGTAGTTGGGAGAGGACGGGCCTTCCCATGCCGGGTGCTGAGCTAGACTGGATATCCAAGTAGGGGAAG
AGTGGACCTTGACCTCGGCCTCACATTGGACACAAAAATCAATCCCAGATGGAGTGCAGATCTACATGGGAAAGGTGAGCTAATAAAGCTTTAGAATA
AACCAGAATATCTTTATGACCTTGGAGTAGTCAAAGTTTCTTAAATAGGACACAAACAGCCCTATCCAGAGAGGAAATGTTTGATTAACTGGACCACAT
GAAAATTAAGACCTTCTGTTCATCAAAAGACACCATCAAGAGAGGAAGAAGGCAGCCTGCAGAGGGGGAGGCGATATTTGCAATGTGTGTGTCTGTG
AGAGAGTGTGAGTGTGTGTATCTCTCTCTCTGACAAATGACTCCAGAATATCTAAAGATATATAGGACCATAAATAAACATAAAACAGGCAGAAGATGT
AGGCAATTTATACAGAATGATGTATCTGACTAAATGATAACATGGAAAGGGACTCAACTTCATTAGTCAACAGGTTACTGCAAATTAAAATCACCATTTG
ATGCTGCTAGACATTCACCATCATGGCTGAAAAGGAAACGACGGAAAATGCCAAGTGTTAGGATGTGGAGCAGCCAGAATGCTGCCTGTGGGAATGA
AAATTGGAATGACTCCTTTGGTAGACTGCTGGGTAGTATTCACTAAGAGTAAACATATGCCCACCCAGGACCCTGCAGCTCCACACATAGGCATATATTC
CCCAGAAACGCAACCTGATGCTCACCAAAAGCATGTACAGAATATTCTTAAACAGCAACCTACACCAGAAACTGCCTGCTGGCCACAGGTGGTAGAAT
GGATGAATGATGTAGGCCATGCACACAATGGAGCACCAGTCAGCAAGGAGCATGAGGGAGCACTCTCCCTTCAGCCCCATAGAGAGGCTGCCAGACA
TCAAATTGAGTGAGGGACATTGGACACAAAGGTGTATGAGTTGCATCATTCCCACCGCATAAAGTTCAAATGTGGGCAAAGCTGGTCCTTGCTGCTAG
CAGTTAGGCCTGTGGTTAATCTCAAGGTCAGTGACAGGAAATGGGAGCCTGAGAGGCTTTCTGGGGGTTCTGTTGCTTCCTCCGGGTGCTGCACACAT
GGGTCCCAGGGACTGAAGATGCCTCAAGCTGCGCACTTGAGACCTGTGTACTAATCTGTGTGCATGCACATTACCCTTTAACTAAAAGTTTAACACCAC
CACCCTATACCAGGGTAGCCCAGGTGAGCCTAATTAGGTGGATAGCCCTGGCCTTCAGGGCCGCTAGGCCCAGCCCCATCCCACTCCCTCACTTAAGGG
GACACGGCTTCCAGACTTCCTGACTAGGTCACCTCCGTGCTTCAGCCTCCAGAGGCTCTGCAGTTCAATCTCCCCTTTCAGGTATCAGCAGAGCACAC
AAGAGATCTATATGGGATGTGCCCCCCGCCCAACCCCGCCCCGACCCCCTGCCCCAACTGCCAGAAGTGTCTCCATGACAATATCAGTGCAGTTGTGA
AGTCCTTTGCCCTCCAGCCACCTCTGTGGCGTGAATGTTCTCTCTGTCACATCCTCCAACACCATCTGCTTGGCTGAGGACCTGAGCAGCATCATGGCT

GGGAGTTCACAAGTGGAGAGGGCTGAGCCTGGCCAGAGTGCACTGGGGCAGGGTGATTTGGATGGCCTTTCCCTTGCAGAGGAAATGAGAAGCCTC
ACTGCTTTCTTCTGGGCCATCCACCTAGACAGTGCGGCCCTCTCCCCATAGCAGCAGCCCTAGGCCAGCCGCTTCTTCCTCACGTGCTCAGACGGAGG
AAGTG
AAGTTTGGAAAGGTTAGGGGGCCACGGGGGGCCGCCACAGCTGGGCTGATGGCAGGCTGGAAGCCAGATTGAGGGCATCTGATTCCCAATCCCATCT
CTTTCCCACTGAGCCATTACGCCTCGGTTTTAATTTAATTAGAAGAACATTTAATCCCCCCGTGCATTTCAGAGACGGGTTTGAGCCTGGCTGGGGCTAT
GTGGGGCTCTGACGGGGATCCTATGACTCATGCAGCCCTGTACAGCCCTCCCGGGCGGGCCCCCAGAGGGCTTCCTTGGCATCTCGTGCAGACAGAGG
TGGTCTGGAGCTTTGCAGAAGACAGGACACTGGTCTAGAAAGACAAAATGGTCTCATCTGTTCTTGACAAGCCCCTCACTCATCCTTCTGGACACTGG
TTCCCAGAATGTCCTTTCTGTCCTTTCCCACCAAAGTTTACAGGGAGAGAAATATGGTGAGGAGCTTAGACACTCACAGAGCTGCAGTGCCTGGTACC
CTGGGGTCTGTGGGAGTAGGTCACAGTGGACCTGGATGTCTCCAAAATGTCCCAGGCCAAGCTGGTCCCATCAAAAGAGCTAGCCCCTTGGTCCATGA
AGCCAAATTTGGGGCCACCCTTGTGACGTTTGCCCTTATTTCCAGACAGCACAGACTGGCCCTGTCCTTGCTGACAAATGCAGAATTCAGGAGACATT
AGTAGTGATGTGGATGAAAGCCAGGCCACAGCGGCCTTGCTGGAAAAGAGCAACTCGAATGTCTTGGCGTGAGATTCTGCAGGGTCCAGGAGAAAGT
TGAAGACATGGAAATTCTAATTAACGCACACGGGGCTGTTTTTGCTCAAGGATTTGCGAAGCTGTCATGTCACAAGAGGCCAGGCAAGCACATGCTTG
CCAGGGAGGTGCTCACAGTCACCTGGTGGGGGTCTCTGCAGCTTTCCCTCTGCCTTCTTTCCACCCCCTCTGCCCAGCCTCGGGGGTCCTGGTCCTCC
CCCATGCCCATCTCAATGGGCCGGGTGCTCCCTGAGTGTCCCTCTGGAGAGTGGGACTGAGGGCGGTATCTGTTAACCTCGAGCTGGGGCTTGCCCAT
CTAAGATGCCAAAACAGAAGAGACAGGGGCCCTTCAGAGAAACCACATGACTGGGCTGGGCAGAGCCATAGGCTGCAGGGCTTATGGGCCTGTCCTC
TTGGGCCGTCTATCTCAGGTATCCATGTGCAACCAATGGGGCAGACGTGTGAATTCCAGGCCAGTCCTGGCTGGCTTGGCAGTTGGGGATGCAGTGTG
AGGGAGGATTTCACAAAAATGATTTCAGCCTCCCAGGGTTGCTTGGAGAAGCTCACCCAGGCCACACCTGCACACAGCCCCCAAGTTCAGAGCCGCA
GATCCTCAGGCCCCTAGCCCTGTGCCTCAGCACTGGCACAATGTGTCTGCAGTCCTTGCTTCATCTTAGGCAGGGTTGAGTGAGCAGCAATCCTAGGG
CTTCTCCATGCCTGGGGCTTTGGGGGTTCTGGGGAGGAAGAGACCAAGAGGGCTATACATGCCCTTCTCCCTCCTGGCACCATTCGAAAGGCTTAGGG
AACTGGTACTGAGGGCTTTGAGAGCCAAAGGAGGACACAGCCGTACAGCCTAAGTGTGTCCTGGGGGGTTTACTGAGCAGACGTTGGAAAGATGAAT
GGAAATCTGGTGGCTGCAGATGGGTGACAAGGGCATTCTAGATGGCAGGTTACCACACGCAAGGCACAGGGATGCACACAGGCATGGCACCGTCCCC
ACCCACTGTCGTGCCAGTGACCCTTGGTGAGCAGAGGATAGTGAGTGGGTCAGAATCCCAGGAACTCATCCTGGCTCTTGGGGTACAGGGCTCCTCTT
GTCATGGGGGTGCTCCCCAACTGCCCATGCAGGCCTACAGTCTCTACTTCCCACCTTTCTCTCAGAAGCCCTGTCATATGGTTCTGGGTGGGAGACACA
GGCTCAGGGAGTTTGAGGCTGGCAGGGACTCTCTTGTGCCCCATTCAACCTAGGAAGACAGCTTCTGCCTGAAAGGTGTGTTCGAGGCTGAGTCCTG
GCCGCCCAGAGCTGGTTGAGGGTCCTGAGGCTGAGCAGTAGGGCCCACCTATAGGCTGGGGTGCAGCGACGCCACTCTTTATCTCCTGGGTACCCCGT
GCCTCAGCTTGTCATGACTCCCTAGCTTGCATCAGGCCTGCTCACCCCCCACCCCACCTCTGGCTAACAGGCCCCGGAAAGAGGAGCTTCGCCCAGCC

CCAGGGACCTTCCCGTAATCAAGGCTATAGTGACCTCACCTCTCTGGGGCCCTGCGGGCGGGGAGAGCTCGAGATGGAAACAAAATGTGTCTTTATTT
ATGTTTTTAATTTGAATTCTGGAACTGGCCCAGCATGGTAATACATTTAACATCAGTAGAATGCTGAAGAAAAATGGAAATGTTTTTTGGTAACTGCCTC
CCACCCACTCTGCCCCCTGTGAGACCCCAGCACACATCCCTGTCCCTCACACACACCAGGGCGTGTGCCTCTCTTCCCAGTTCAGGGTCCATGGGGCC
AGGCTGCCCACAGGAGCTTCGCTGCATCCCTGAGCAGCTGGAGCCGAATTTAATTATGGGCATCACTGGGCGGGCTGTGCAGGAGGTGGAGGAATGC
AGCCCCAATGCGGGCAGAAGACCTGCACGGCCCCCACACCCCTGCCGCCAGGCGGTTTGGGCCCTGAGGAAACAGGGTGCCCACTGCTGCCAGCCT
TCTCCCCCAGATGGAGTGGGGCCTCTACCCAGCCTGTATCCCAGAGGAGCCTGGGTTCCTAGGCCCTGACACTGTACGTGCCCACTCTCCCCTTCAGG
CCTCTCTGGAGGGACCGTACCCTGACTTCTGAGGATGCAGGAACCCAGCTCCCCTGAAGCCTCAGCCCTGAGTCTTCCCTGACCAGAACACAATGCCT
CAAGGTGGCCCCTTTGCAGCAACCTCCTTGTTTTTCCCTCTCCCAGTCCCCTCTTCAGATCCCTCACCCCAAGCCCCATGCCTGGGCTCCTTCCTGCCTT
CCCAGGCTGGCTCATATCTAGCAGCCACTTTTGGAACAGCCTGCTCTGAGAGAGGCCTTGGTAAGTTACAGGGCAGGGACCTGGTTGGGTAATGACTG
GAGTCTCTGACCTGCCTGGGCCCACTTATCACTGGAGGCCATGGGAGTGCCAACCTGAAAAGGCTGCAGGATCTTGCAGAATGGTAATGTGCCAGACC
CAAAGTGGGCAGAGAGCTTGGGGATGGATGTTCAGGTCTCTCAGGCCCTGTCATCCTGGAGTGGAACAAGAAACAGCCCAGTGTCCTAGATGACACA
GCCACACTGGCTTGGGGTCACCCTTGGGCAGGTGGGTAACCTCTGGCTCCAGGGAGCTTGGCATGTGCTGATACCAGCACTGGCAGCCCTCACCCCCA
TAGTGGTGATTTACTGGGCAGCTGCAAGTTTGGCTGTGATCCACATAAGTGCCTGGTCTCTCACCACCTTCACCTGCAGGGGCCTTGCAAGACCCAAG
AGTTGGTAAGGCCTGGCTGCCCAGCTAGATGGACTGTGGGTGATGGCCCTCTCCTCCCAGTAGGACTTTTTGCATGTCTGGATGCTGGTCCGTGACCAA
GAGAACCAAGGTGAGCGAGAGCTCCAACAGGAGAAGCAAGCAGCCAGAAGGATGAGAAGGGACAGTCCCCTCTCTGAGGGGACAGCCAAGAGGC
AATACAGGCACTGCCACCTGTATTGCACACAACATGAGCCACTAAACTAACACTGATCACTTTGGAGGTCCATCATATCCAGGACTTGGTACAGGCCTT
GGCCACAGTGTAAGCCATCATTCTGCCTTCTGGTGCTGACCTGTACCTGGGCAGCCCATCTGGCTACCAGCCTGCTCACACCTCTGCTCCTGCCTTTTTA
CCCAGCCAGGAGGGGCCCAGAAGCTCAGAGCCCTATTCTCAGGGTGTCCATGACTAGTCACCCCTGCTTTCCAGTGATCTGCTGGCTCCCTTGGAGCC
TCTTCACACCCTACCCACCATGAGGTACTGCCAACCTCTCTGTGAACTGGGGGGGCCTGGAAAGACAGTGGGCAGGTCCTGTCTGATCATAATTACCC
CTTAAGATAGCCACTCCTTCCCTGGAGGTGGCCTCATTCTGGCTACCCAACCCTGCCAGTTCTCTGGTTACACCTGCAGCCCCCAGCACCATGGGTCCC
GATTTCCTCAGTGATTTGCAAGGCCAGGCCTTGCACCTGGCTGGCTGCAAAGGCAGGAGGCCCAGGGCCTCACTACGACAAAGACACACATGGCTCA
GGACAACCTCCTGACCCCCAGAGAGTCCAAAGAACAAAACACCCTTTAGAAAAATGTTGGTAGAGAGAAGGAGAGTTTCCACAAGTGACCCTCAGA
AGGGCCGCGAGTGTCACTCACAGATCTTGACACAAAGACATTTATCAAGGGGAATCCCCAACGGGTTAGCAGCCAGAATGGGACTGTGGCCAGAATG
GACTTGGGTGCCCCACTGACCCCGCTGCACGGCTGTCACCGCTGGGCACCCCAGCGGGGAGTCACATGTCGCAGGGGAATCACACCGTGGCCGGGCA
CCGGGGATTGGCCTGGCCATGCCGTGACGGCGGGGGAGGACGGGCATGCATCCCAGGCCAGCTGTGCCTCAGGGGAAGGCCAACTGGAAGGCGTGG
AAAATCAGCCATTTCCTCCACCTGAAGGAGCCCCTGAGGACAGCAGCAGCAACTGGCAGACTGCACCCGGCATCTGCCCCATGGCTTTGGCCAGGGT

TCCATGGGGAAATGCTAAAAAACAAAGTCGTCTCTTTTTTGGGGGGCAGGGGAGGGGGCTGGGGAGAGAAAAACAATCTCAGACTCTCAAAGCAT
GAAAACAAGTGGAAAATGATCAGTCTGGTTTTCCATAAGTCAAAACAAGAGGATCATGTTCCCAGGCTCAGTGGGAAGTGCCCTTGCTCTCCTCTCTT
GCCTGGTGCCTCCGTGTTGGGGCCAGGCAGACGGCAAGTGGTGGGCACTAGGCGAGTAGATAGCACTCTGGCCAGAGAGCAAGGCTTCCCCAGCAC
CAAGTCTGACTCCTTGAGCTCCAGTCCCCTCCAGCCCAGCCCTTCACGCACATGTAGGCACTCACTGTGAGATGTGGGTGATGGGTGTCAGCAGAGTC
TCCCCTTCCAGGTCCAGGTCCTCGGCGAAGCTGTTGGTTCTCATGAAATGGGGCATGCTCACTTCCAGCAGTGTTGGGCTCTTCCTTACTGAGGAGAA
AGTGTGGGGACAGCACGTTAGGCCTCCCACCCAACCTGCCAGTGCTGTGGAGCCAGGCCTGTGAGCTCCCAGGGCCAGGTGAGGAGTGGAGCTTGT
GCCTGGAAGCTCCAGCCCTGACAATCAGTTTTATAGTTGGGTGTTGGGGTGGGTGGGTGTTTAAGATGGCCCCAAAGTGCCTGACACCTCAGTGTCTG
AGGAGAATAAAGGCCAGTGAATCATCTTCGGAGTGGTAACTGTGCCTGGGGCTGTGCTCAGGTGTCCAAGACTCTCCCCACCTCAGGTTCTGGGCCTC
TTCCCATGTTCACAACTGACACTTGGGTCAAGAATAAAACAGAGGTGACAGGCACAGGCCTGGTAAGGGACAACAGCTCCCACCACCATACATTGTC
ACACTCATACATTGACAGAGTAGTGGGTCAGGAAAGATGGGGTGATAGGACCTCTGACCCATGAGGAATGGCAAGCCTGGGAGGTGGGGGCAGCCAC
AGAACTCCTTCCTCAAAACACGATGCTACATCAACCAGTTGTACCCCCAATGAGGCACAGGAGTCCCTCCAACAGCTGGCTTCACATTATGATTCCTGG
GCCCACCTCTGCAACTTTGACACAGGAGCTCAGGATACTGATCCTTATCACTCCCCACTCTCTGTTGACACTATCAGTATTTGCTCTTGGCTTATATTTGT
CTTCATTTAAATATTTTATTGCAAAAGGATTAAGTACTCATCTGTGAGAAGTGGAATAATTCAAAGATTCACAATGGCTTTCCTCCCCCAAGGTCATCAG
TGTTAACTGCCAACTAGATCCCTAGCCCACCCTCTCTGCTCATCCGAGCAGCAGATGCCAGGTGGGAGTTGCTTTTATGCACATGGGATGTCACTGTGA
CTCTGTTCCTGTCACTCTGTAACATAAGCTCATTTCATTTTTCTCTAGGTATATAGGCATGGATTTAATTATTTTAGTCATTACATAATAGTCCACAGTTTAG
ATTGGCCACAGCTAACCATTCTGTATATCATGGACATTCAGGCTCTTATAGTTTTAAGCTGCTTAATTATTCAGACTATGATGCCATATTCATCTTTATTCTT
AAAAGCTGACATACTGGAGCAGGGTGAGAGGGTGTGTCCTTAGTAATTTTAATAAATGTTGCTAGATTGCTTTCCCAAGAAGTTGTAGCAATTCAGGCT
CCCCCAGCTTTCCCACATCCATGCCTGAATGAAGTGTTGAATGAAGCCCGAATGAAGGTTGTCAGCACCTTTTGCAGGGACTATCAAGCATGGATGAA
AACTGAGGCATTGCTGTTATTTCCATCTGTGTTGAATACCTTTTATGGGCTTGGCATCTGTTTGGGTTTCCTCTTGCAGAATTGCCTGTTCATGTCCTTTG
CTCACTTTTGCTTATCACTTTGTAGGAATTTTCTATACATTACAGACATTAACTCCTTGTTATATGAGGCATAGATAGCTTCCCCAGTCTGTTTTTTCACCT
GAATTATGTTGCTTTTATCCTAGAAAATGTTTTATGTTTTTAAAGTGATACATCTTTTGCTTTATAAAAAAGGAAGGTTTTCCCACATCAGCGTAATACAAT
TCTCTCTTAAATTTATTCCTAATTTCACTATTTGAATGTGTTGTATGTATACATATGTATGTGTGTAAAAGTTTTAATGCATTTAGTATCTATGCCTGATAGGA
CAGATGATCAACAATTAGAAAATGAAAATTTAAAGGGTACTACATACAAACTTACTGAAAAAAAATCTAAAATAAAACATAAAACCATAAAATATCTTG
AAGAAAACATAAGAGAAAATCTCCAGGACCTTGGATTTGGCAGTGAATTCTGAAATACGAAACCAAAAGTTTAACTCGTAAGAGAAAAAGAATCAAC
AAATTGGACTTTATCAAAATTAAAAGCTTTTGCTGTGCAACAGACACTGCTAATGGAATGAAAAGACAAAGCACAGACTTGGAAAGAATATTTGCATAT
CACCTATCAGGTCAAGGACTTGCACTCAGAATATATAAAGAATGCTTACAACTTGACAATAAAGTGAACAATCCAATTTTAAAAATGGATTATAAATAAG

CACATGAAAAGATACATAATTAACATAAATATGGAAATGCAAATTAGAATCACAATGAAATTCCATAAATTAAAAAAAAAATTCAAAACCTGACAATACC
AACCACTCTTGAGGATGTGGAACATGTAGAACTCTCATATGTTACTGGTGGGAATGCAAAATGGCACAGTGACTTTAGGAAATAGTTTAGCAATTTCTT
ATTAAGTTAAACATACACATACCACATGACCTATCGGTTCCTTTCCCACCCAAGTGAATTGAATTTTTATGTTCGCAAAAACCTGTACATAAATGTGTATA
GTAACTCTGCTCATAATTACCAAATACTGGAAACAATGAAGATGTCCTTCAACAGGTGAATGCATAAACAAAATGTATATATTCATGCAATGGAATACCA
CCCAGCAATAAAAAGAAATTAACTAATGATTTATGCAACAACATGGATGAATCTCAAATGCATTTTGCTAAGCAGTGAAAGAAGCCAGATGCAAAAGA
ATACATATTATAGGATCCCATTTATAGGACATTCTAGAAAAACAAAACTACAGGGACATAAAACAGATCAATAGCTGCCAGTGGTTAGGGGAGAAGTAC
TTGACTTCAAAAGACTCACCCCAGGGTATTTTTAGGGGTATGGAACTTCTGTGAATGATACTGGATTGTGGACACATGACTCCAGACATCTGTCAAAAC
CCACAGAACTGAATCTCAAAGAGTGGATTTTACTGTATGCAAATTTAAAAAATTAACCCGGATGTTGGAGGACCCTACGATGAAAAGCAGACTATGATG
AGCGTGTTACAAACAAATGACATAGCAGTGAAAGGAAAGGAGGAGGAGGAGCTGGCATATGTTGCTTTCAAAAACACTGTTTTGACTGGACACTCTA
TGGTTAAAGCAAAAGTCTGTCCTAAACACTCTAATCTAGTAGGTAAATTTGCTTCACATAGGGTACTACGTGTACAATAGGGTTACACAAATAAGTAAAT
GAACTGTAGGTAATGGGAGCCAGGTTTCTAGCTGTCAGAGAAAAAGCTATAAATAAGTTGTGGGGGAGGGGGCAAGGATGAACTCTGGTCAGAAATG
TCAGTATGAACTCATGGTTAGCTTTATATAGATACAGATATGTAAAGATGTATGTGTACTCAGGTTACATACATACATATATTTCCTAGCTCTGTCCACTGA
GAGGAGACTGAAGCAATGATACCCTAGTAGCCACGAGCATACCCCGTGCCTAGATCCAGGTTTTCTATTCATTCTCCAGGGAGCCAGGGCTCTCTGGAT
GAATGGATGATTCTAGGGCTAGGGTAGGGAAAATAAAAGATGAGCCTGAAGTATTGTCAAGTGCTAGAAAAGAAATGCTTAAAAATAAATGAAAAGGA
TGGCGGGGGGCACATCAAAAGGACACATGAACCCACCAGTGGCCACAGCTTGAACAATTTGAGCAACAAAATAAATAATATTGGACTATAACTCAAAA
AATAAATACTCCCAAATCCACACTGATAAAATTAAATAAACAATTGGAGGAGAGGGACAAATCTTCCTTATAAAGGAATTCCAAATAAAAAATGTAGAA
GGAATGAGGAAAATAGAAGTACATCACGGTAATATTTGCTGCAGGAAAACGATCCATGAATGAATGCTGAAATTAGTTAGTGAAACTCAGAAACTTCA
CAGTTTCTTCCTTGAAATGTATTTTAATTGCTGTGGTAGTTTTAACATATTTGCAGATTTTTTTTGATACTCTCCTGCAGGAGGTAGAGTTGAACCCCTCC
CTTCCCCACCTTGAGTGTGGGCTATACTTAGTGACTCGCTTCTAAAGACTAAGCTATGGAAAGGGAAAAACAGCTATTCACTTACAGTGGAGGAGCCT
GGCCGACACCACTTTAAACGAGTGGCTCAAGGTTAATATTACCAGTGATAAACCATGTTGATGTCATGTTCCACAATGAGGAAAGCATCTCACCTCACT
GGCCTCCTTCCCCTAAACCTATAACCCTGGTCTAGTCCTGAGAAAACACCAGACAAATCCAAACTGAGGGACAATGACACCTGACTGGTATTCTTCAA
AAGTGTCAAGTTAATGAAGAACAAGACTGAGAAGCCCGTCACCGATCAGAGATGACAATAAATGTAATGATCGATCCTAGATGGGATCTTGGAACAGA
AAAAGGACATTAGTAGTTAAACTGGGGAAATCCAAATAAATTTATAACTCTAGCTTAATAGTGTACCAATGTTAATTTTTTAGTTTTGGTCAATGTACCAT
GGTTATATAAGATGCTAACATTAGGGGAAGCTGGTGAAAGGGATGTGGGTACTCGGTACTATTTTTGTTTCTCTTCTGTAAATAAAAAATGTGAGGTAAC
AAGAGAAAACAGAACCTGTATACAATAGCACAAAAGAATATTAATTATCTAACTAAGTATATGCAAGAACTCTGGAGAAAATTGTAAAACTTTAGTAAG
AGACAATGAAGAAGATATAAATGGAGAAATATGCCTTCATTCCAATGACTAGAAGACTCAATACTGAAAAGATTATCTCAAAATCTGAATAAATCTCTCC

AAATCTTAATAAAAATCTCAAGGCTTCTGTGTGAGAACTTTGGCAAGCTGATTCTAACTGCGACTTGGAAGTGCAAAAAGCCAAGAATTGGCAATATTC
TTGAAGAAGAACAAGGTGGGAGAACTTGCTGTACCAAACATCAGTACTTCTTCCAAGGCAGTGGTCATTAGGGCAATGTGGTATTGACACAGGGAGA
GACAAATAGGCCAATGGGACAGGATTGAGCATTGGGAAACAGCCCACACAAATATGGAAGCTTGCTGTGTATCAGAGGTGGCCTGCACATCCTGGGA
AGAGCATGGACTTTTCAATCAAATGTTCAGGAGCAGTTGGATACCCACACTGGAAAAACATTAAGTTGGACCCTTAATTACCTCACATCATATACCAAA
AATTCATTCTTGTCATTCTAGGGGATTGAAGACCTAACTATGAAAAGCATAACTATAAAGGTCACATGACTGAATATAGAAGAATATCTTAATGACATCA
GCATTAGGATGGTTTTTTTTAAAAGAAGCAATAACCATGAGAGAGAAGACTGAGTAATTTGACATTAGAGTTAAGACCCAATCATCAAAATACACCTTA
AAGAGTGAAAAGACAGTGCCCACAAAATGCAAAGGACTCCTACAAATCAATAGGAAAAATATAAATGACTTAATAGAAAATGGGCAATAGACAAAGT
AGCACATCACAAAAGAGGAATCCAAAAGGCTGATGAGCATGTGGAAAGGTGTTGCGCCTGACTAGCAATCAGGGAAATGTAAATAAACTCAGGAGCT
CATGGCCCGTGGGGGCGAGTGGCAGACTTCCAGCAGGAAACCACACAGACATTACTTAGTCACAGCCAGGAGAAGGGCTCAGAGTGAAAGGGCTCT
GTGCCCATGACATCCTTAGAGAGACCAGGGAAGGACCTGTAAGGCTTGAAGAATGTGCAGATTTGGAGACCCACAGGTCTGGGACCTGGCAGAAGCA
TGTTACTGCCCCCATTTTAGAGATTTGGGGCTGAGATTCAAGTACCCTAGGTTATATAATGCAGTTGTCAAGTGACAGAGCTAAAACTTCATCCAGGATT
CAATTTTTTTTAAAGGAAGGAAGAAAGAAAAAACATATTTTGATGTTTGCATCACTTGTATGTAGAAGGAACGATGGAGCACAGGCTAGAACGTAGAG
TTGCTTTCCAAACACCCATGATGACAAGTGCCCATCTCCAAAGCCTGAGGGCCAAGGGTGAGCCACCTGACCCCTGAGGAAGGTTCTGTCCTGGTGTT
TTAGGAAATTAAAATGTGCACATCAGTTGGCTGCCCAGAGTGGGTGGGACCTGCACCTGCCAGAGAGGAATGTGTGCTTTTTGGTTTAATCCTGGGAA
CGGCATATGGGGCCCCTATAACAAGGGGCAGAGAAGGCCTCCACAGATTTGTTTTTCCAATTCAAGAGGAGAGGTTATTGTCTGCTGGCTTGTGTGTTG
GAGACTGTGGGGCGTGCTTTCTCCACCACAGGGAGGCAGTGACCAGAGCTGCCACCTCTTCCCTGTTTTCACCTGTGGTCGTAGCCAGGTCAGGCATC
TAGGAGAGCATGGAGGGGAGCTGGGGGTGGGTGGGTGGGGATTCAGCTCTTTCCAGACACACTGGTGCTGCCTCTTTCAGGCCAGGGCTTGAGGGCA
AGTGCTGTGCCCTAACAGGTCTTGCTTGTCACCCCCACCTCTGAAGTGGGTATTTCACAGCCTGAACTCACAGCTGCAGAGAAGGTTGAGCCCATGGT
GTACTTGAAAGGGCAGTTCATCTGGATATCCAGGCTCTGCAGGAGGTACCGGAGCAATGCCAGGTGCCCTTTGCTGAACTGCCTAGAGTGGCTGGCCC
TGGGGACAAAGAGCACAGCATCCTGGTGACAATGAAGTCACACTTTCCTAAGACAGTCCTGGTTTTTGATGCTTTAATTCTTTGGCTGCCTTTGCTTTG
TCAAGGCCAGGATCTGGGCCTAAATTTTCAGGGCAGAACAAAACAATTTTGTTTTCTAGGAAAGTGGGAGGTCTGGGATGGCAAAGGCAGCCTCGGG
CGTGGCGACTACCCCAGCTGGGGACGCTGGCTGCCAAGTCTCACCCTCTCAAGAGAACAGGAATCAAACTCAGCACAGCATCCTCCTTTCTAGTCAGT
GGTCCTGCTCGATAGGGGGATTTGAATTTTATTTTTTTAAAAATACAGGCACAGAAAAAAGACTAGAAGGAAACAGATCCAAATGTTAACTTTGTCTTT
GGGTGATTTTCATTTCCTTCCTTATTGTGTCTGTGTTTTCTATTTTGCATGATACTTTTCCAAGTCAAGAAACAATAATTTGTGGAAGTCTCCTGTATCTGT
GCCTTGCTCTCTGGCCCACGACCTTGCACATCTTATCCTAGAGTTGCCAGAATATGTCCCTCCACCTGGCCTGGAGCTGCCCATGAGGCTACATAGAGC
TCTCCTATCATGAAAGTCTTTGAGGCTTTCTGTTCCCAAGAAATTCTGGTCTCACTTCCTCTGCCTTCTCATCGAGCCCCAGACCCCTCCCTGCCCAGAT

TAGGTCAGCATTCAAGCCCCAAGTCCAGCCTATGACAAAGCTCAAAGCTCCAGCCCATCTCTGCTGCCCTCTGCCCTCAAGAGACTCTCGCCAGTGTC
TGGGCCCTTCCTAGTAACTTCACCCAACCACTCACCCCCTGCCCTTCCCCTCCTGATTGATTAAAATCCGGCCTAATCTCCCAGGTGTAACCCAAGCCCT
CTTCCACAAGCTTTCCAGGGCTCCGGAGATTGCCCCCTCCCCTGTACGGCTGCAGGGCTTATGTGTGCCTGGCGCTCAGCACGGGAACCTTGCCTTCT
GCCTCACCCATGTCTCCCCAGGGTTTGGCACAGGCGAGTTCCCTGAGCCAGCCTACTTCACAAGGATGAACAGAAGCTCTCCCTATCCTTTGCAAGTA
GAAGCCCCCTCCCTGGCCCCCTCAAGAAATCTCCCCCTCTCAGTGGATTCACCAAAGCCCTGCGGATCCTGCACTGTGAAGGAGAAGCCCTCCCCAGG
GGCATCCGGACAGCTGCAGCCAGGCCCTGCGTGACATTGCTGAGCCGCAGCCCCACAGCCTGTGCCAGCTGCCCATGCCCGGCTCATAGACCCTGAG
CCAGAGTGGCCCCGCCACCACCTGTGTCTCCACTCAGAGGCGAGAAGTAAGGGACATGGATGGAAAGTGTAAAAGCCATTCCCACAACAGCTGCCAG
GAACAAGGCGAGGAATGGAGTGAAAATGTATATTCACAGCTGGCACCCAGTTCCGCTTAGGAAAATACCTTGCTTGAGTGGACACCTGTTGTTTTTGC
CTGCCAGCAGCCAGGCCTGGCTTCTGGGAACAGCTTCTCAAATCTTCCTTAGGGGGCTCTTCCTTTCCCACTCCTAGTGTGCAGTGTTTAGGTACCCTC
TGGCCATCCCCAAAGGGGTCCCTGTGGACCTTGCCTCAGCCATCGGAGGTACAGCTGCAGGTCCAAGGATGGCTTGTACCCCAGCTGAGGCCAATAAG
AACCTGGCCTTAGATTCTGGCAGGAATCCTGGAAGAGATGACCTCGTTCTGCTGGGCTTCCTGAGCTGTTGAATGCAAGCTGGTGGCCATTTTGCCCCT
TTTAGAAAGTAGCTGCCTAAGGATACAGCCATCCCATGGGGCCAATGGATAGAGAGCAAGGGAAAGGGAGAACAAAAGAGGGAACGGGAAGGGAGA
AGGGGAGTGCAAGAGGGGAGAGTGAGAGCAAGTGAGCAAGATCATCTCAGCTCTGGGGTCCAGCTGTGTCTGAAACAGCTGGGTCCAGCTGTGTGA
GCCAAAAAATGTCCCTTTTGTGCTTAAGCTTGTTTGAGTTGGGGTTCCATCATTTGTTACCAAGAGAGTCTTCAGGAATGTGCATGACAAAGTTGGGGG
TGAGGTGTGAGCCTACGGACATGGGCCTGGGGGCAGTCAAGGTTACAAAATAGCTAAACTGGTCTGCACTGCCCCCACTAGTTTTCAGGGCAGAGGG
CAGGGAGAAGGCTTGTGGGAAAGAAGCTGGCCTGAGGCCAAGGCCCTGGCCCTAGCCCTGCAACAGCACAGGGAACTCTTGGAAAGGGATTTCCTA
AGGTGGGACATTGGCTTTCTACTGCTGTGATGCAAGTCCTCATCTCCCCAAACCTTCAGTGAGCCTCTCTCCACACCCTGCTGTATGAGGATGTTTGAT
GCAAATTATGGAACATGTTGGATCTCACATTGGATCAAAGGTGTGCAGAATAGATTTGCCTCAAGCATGACAGTGATGTGACACAGTCAAAGATGACTT
CCGGAATCCTGAGAAGTCCCAGGGAGGATGCTGGGCTTTCCAAAACCTAAGTTTAGGATACTGAGGACCACACCCCACACCCCAAACACTTCTCCTTA
GAATAAGTATAGCAAGCCTCAGCATACACAAATCTCAGAGTGAACGGAAATGTCTCTGCAGGCCCAAGGGTGGGGTCTGAGTAGCGGGGGCGCTGAG
GAAGACAGGCAGTGACAGAGCCAGGACAGGGTCAACCCTGAGCGTGAGTGAGCCCAGGAAACGGAAAGAGAGCAACACAGCCACCCACACAACTA
AAATCGACCCACCCGGGTCTCCATGCTGCAGGAAGTCACTGTCTAGGTCTCCCAAGACACATTTTCAGGGGAAGGTTCCAGGCAGCAAGAGCAGCCC
TAGGAAACTTTAATATACAATGGAATCTGGGTTAAGCACTATCTTCTCCAAGAGTTGAGCTCACAGGACATTCCCCAGAAACCTCTGGAGAGGTGGGG
AGGAAAGTTTCTGGAGGTCTTTGGATGGAGAAAACAAAAAAGTGGCCTTGATTTTCAAGACAAAATTACACAGGAAGAAAAAAATGTTTTTAATCTAA
TTAAGAGCGATCTTTTCACCTTCATCACAACATCGCGGAAATGTGCCAGTTCTTAACTTCACACAATAATCAAAGCAATTAGTGAGGGAGAAGCCTTGG
AAACCGGTGCAGATGTTCTCAGAATGTTTAATGGGGAAATGAGTTGCAGGCCTCATTCTCAGCATCCGGCTCCAGGCCCTGGCTACCAAATCCAGACAT

CTGCTGGGGCCCCACCCCCAGGCCCCGAGGGCGCGGGCTTGAGAAACACAGGTCCCTTCCCAGGCCACCTCCCAGCCAGGCCAGAACTGGGGCCGA
GAGGCTCCCCATAGCTTCTCAAATTGGAGCCTGGACACAGTCAGAGCCACCCCAGAACTGGGGGGAGGCTCAACATCAAAATTATGTTAACAACTGAT
GTCAAAAGTAATCAACTCAGCTTGGCTCCCCCACTGCTGCTGGCTGCAGGAGGGCTGTGAGTAGGAGGAGAGCAAGCGGCCCCAGAGGGACCCTCG
GCTATGACCCCCTCAGCCTGAACACTACTTCAAGAAAAGATGTGGTGGCATCTGTCTGGTATGGTCCTGTGGGCTCCATTCCAATGCTAATGGACTCTT
AAGGGGCCCAGGAATCAAAATGGTCAACACTTGAGGAATTTCCAGTAATCAGAGAAAACGTATAGAAATGAGGATGATGCTTACTTAGGAAATGGCCT
ATTACAGGAAACAGAGGGCCACGTGGCTAATGTCAGTGAGCTGTGGGAGAGACAGAGACCCCAGGTGGGGCAGGCAGATCAGCAAATCTTGGAGGA
CCTGCTATGTGGCAGGCACTGGAGACATGGAGGTAAAGAACACAATATCCATGTTCTCAGGGACACACTCATATGCCCTTAGCATTCACGTTCTTCACA
CTGACAGCTTCTTAATGCAAGCCCACCAGATACTTTCATAAGCACTACCTCTGACTTCAGGCACTTGCTGAGCTGCACACAGGGCAGGCCAAAAGGAA
TGGAAAGTGAGCCACCACCGGGGCAGCACTCACTAGTGAAAGATAAATACCCCAGCTCTCTCAGCCGCTTGTAGAACTCTGAGGAGTGTTCTGTGCA
GCTTCCCAGCTGCCCACCATGAAGCTCTTTCTCAATAAGTGACCAGCACAGCTTAATTCCTGAATTAAGCTGTGTGTGCTGTGAGACTGACACAAGAC
AGGGTGACAGGAAGCTGTCGGAACATTTGAGTAGGGAAGCAGCAAGATCCACTGTACATTTAAAGAACTCCCTTGAGCTGTTATGTGAAGAATGGAGT
ACAGGAAGCAAGAGTGGAAATCAAGAGACCAGTAATGAGGTATGGACAACAATGTAGGCAGAGAAATGGTGGTGGTTTTAACCAGGGAGATAGCTG
GAGAGACTAGGTGTGGGCAAATCCAGAGAGTTGATAAGACATACTGATAGATTAGAAAGGGGTACTAAGAAAAAAGGATAATTTAAAAAAGACTTTCA
GCCAGGCTTGGAGAAAAATAGCATGCTGCTCCTCATCCCCTCTGACTATAAATCATCTCACTCATCCTCCCCTAAAAGCATAAAATCAGTAAGTGCAAAT
AAAACCACATGACCCATGCTGTTAGCAGTATTAGGAGAAAGAATACCTTCAAATTGCCTGTGGGTAGCAAAGTGAGCCAAATTCCAATAGCATTCTAGT
CCCCTGCCCAGCACTGCAAAACTGCATACAGAATCAGAGAAGAGGCTTAAAATGCTTTGTCAAAAAGTTTGAAGGTAGTTAGCAACATCAGCAAAAAT
AGAGGAGTAAGTACCTCTAAGGAGCCTTCTTGCCTTCACAGACATCCTAAGCACCAAGCAGCCTAAGCCACTGATGAACTTGCAGATACCACTGATGT
GGATTATAGTCTAATTAATTGTAGAGACACCACACTTCTGCACTGAACCAGAACCAAAGCCAAAGCACCCTACCCAAATGATACTACAGACGCGTGTAC
AGGAAAAGTCCTTACTAAGGCTACTCCATAAAATTGGAAGTCTCTGTTATTCCAGATATGCAGATATCAACTTGGGGACACAAAAAACTTGGGGGGAA
AAAAACAAGAAAACATGACTTGCCAAAAGAACACAATAATTCTCCAATAATAGACTCCAAAGTAAAGGAAATCTTAAGGAAACCCAATGAGATACAA
GAGAACACAAATAGACAATACAAACATATCAGGAAAACAATTCATTATCTAAATGGGAAATCCAGCAGAGTTAGATATGAAAAAGAACCAAACAAAAA
TCCTGGAGGTGATAAATTAAATGAATGAAATAAAAAATACAATTGAAAGCTTCAAAAGCCGGCTAAGTCAAGCAGAAGAAATAATTTCTAAATTTGAA
GACAGGTGTTTTGCAATAAACCAGACAGATTTAAAAAATAACAATAAAAAAGAATGAAGAATGCCTGCGTGACATATGAGACACTATTAAGCTAACAAT
ATTTCACATTTTGGGAGTTCCAGAAGAAGTGTTGGGATAAGGACCAGAAAATCTATTTAAGAAGATAATACCTGAAAACTGCCAAAGTTTGGGAGAGA
AAGAGACTAACAGATAAAGGAAGCTCACAAATCCCTAAATAGATTCAACCCTAAAAGGGCCTCTCTGAGGCACATTTTCATCAAACTGTCAAAAATCA
AAGAGAGAATTTAAAAAACAAGAGAGAAGCATGAAGTCACATATAAGGGAATCAACAGCAGATTAACAGATTTTTCAGCAGAAACCTTCTAGGCCAG

GAAAGAATTGAATGCCATATTAAAAGTGCTGCAAGGTGGGGAAAAGCTCACAAAATTGCTAGCCACAAATACTATACCCAGCAAAGCTATCCTTCAGA
AATGAAGCAGAATGAGGGAAGAAATTTTGCCTCCCTCAGACAAGCAAAAACTGAGGGAATTCATTACTACTAAATCAGCCCCACAAGAAATGCTTAAG
GGAGTACTACATCTGGAAGTAGGAGGATGATATCTACCATCATGAAACTACAAAAGCATAAAACACTAGTAGAGCAGATACACAAATGAGAAAGGAAA
AGGAATAAAAGATTATCACTACAGAAAACCAAAAAATTGCAAAGAAAAACAATGAGAGGAAACAAAAGTATACAAATCAGTCAGAAAACAATAAAAT
GACAGGAGTAAGTCCTCACCTATTGATAACAATCTTGAATGTAGGTAATTTAAATTTCCCAAATGAAAGATAGATACTGGCTAAATGAATTAAAAAAATA
AAACCCAATTACATGCTGCCTATAAGAAACTGACTTCACCTGTAAAGACACCCATAGACTGTAAGGGAATGGATAGAAAAAGATATTCCACACAAACA
GACACCAAAAGCATGCAGGAGTAGACAGACAAAAAATACTGAGTCAAAAAAAAAAAAAAAAAAAAAAAGAAAAAGAAAAAAGAGACAAAGGAGG
TCATTATATAATTATAAATGGATCAATTCAGCAAGAAGATATAACAATTATATATGCAACCAACAACACTGGAGTACCCAGGTATATTAAACAAATATTATT
AGTGCTAAAGAGATAGACCCCAATACAATAATAGTTAGGGATGGCAACATTTCATACTCAGCATTGGACAATTCATTTGGATGGAAAAATATCAACAAG
GAAATATTAGATTTGAACTGCTGCTTCGTTAGCACACAGCACATTCTCCAGGATATACCATATGTTAGGACACAAAACGGGTCTCAATAAATTTTTAAAA
GTCAAAATCTTATCAAGTATCTTCTCAGACCACAATGGAATAAAACTGGAAATCAATAACAAGAGGAACTTCTGAAATTGAACAGATACACGGAAATC
AAACTACATGTTCCTGAATGACCACTGTGTCTATGAAGAAATTGATTTTAAAAATTTAAAAATTCTTTGAAACAAATGAAAATAGAAACACAGCATACA
AAAATGTATAGGGTACAACAAAAGAAGTGCTATGAGGGACATTTATTTCAATAAACACCCACATCAATAAGGTAGAAAGTTTTTAAACAAATAACCTAA
TAAACGCATCTCAAGGAACTAGAAAAGCAAGAACAAATCAAACCTAAAATTAGAAGGAAATAAATAGTAAAGATCAGAGCAGATCTAAATGAAATAG
AGATAAAAGGTAAAAAAGACAAAAGATCAATGAAATGAGGCCTCCCCCCACCCCCCGAGACACGGTCTCACTCTGTCACCCAGGCTAGAGCGCAGT
GGTGTGATCTTGGCTCACTGTAACCTCTGCTTCCTGGGCTCAAGCAATCCTCCTGCCTCAGCTTCTGAGTAGATGTGCAGCACCACACCTCGCTAATTT
TTTTTTTTTTTTTTGGTAGAGATGGGGGTTTGCCATGTTGCTCAGGCTGGTCTCAAACTCCTGGACTCAAGCGATCGGCCTGCCTTGGCCTTCCAAAGT
ACTGGGTTTACAGGTGTGAGCCAACAAACCCAGCCAAAAAACTGATTTTTTGAAAAGAGAAAATCAATAAACCATTAGCTAGACTAACCAAGAAGAC
CCAATAAATAAAATCAGAAATGAAAAAGGAGACTTACAATGGACACCACAGAAATACAAAAGATTATTAGAGACCATTATGAACAACTATATGTCAACA
ATTAGAAAACCTAGAGGAAAGAGATAAATTCCTGGACATATATAACCTCCCAAGATTGAACCAGGAAGAAAAAAAATCTGAACAGAGCAATAACAAG
GAATGAGATTGTATCAGTAATAAAGTCTCCCAGTAAAGAAAACCTCAGGATTGCACGGGTTTTCTACTGAATTCTACCAAACCTTTAAAGAACTAACAT
GAATTCTTCTCAGACAATTCCAAAAGACTGAAGAGAAAGGAATTCTCCCTAACTCATTCTGTGAGGCAAGCAGTATCTTGATACCAAAACCAGATAAG
GACACAATAACAGCAAAAAAGAAAACTACAGGCCAATCTCCCTGATGAACATAGATGCAGAATCCTCAGAAACAAAACACACACACAGACACACAA
ACTAGCAATCTGAATCCAATCGCACATCAAAAAGATAATACACCATAATCAAGTGGGATTTATCCCAGGGATTCAAGGATGGTTCAACATATACAAATCA
ATAAATGTGATACATCACATTAACAGAAGGAAGGACAAAAAAATACATGATCATCTCAATAGATGCAGAAAAAGCATCTGATAAAATTCAAAATCTCTT
CATGATAAAAAAAAAAAGCTCATCACATAATGTCTAGAAGGAACATAACTCAATGAAGGTCATATATGACAAACCCACAGCCACCTGGGGAAAAGCTAA

ACACCTTCTCTCTTAGAACTAGAACAAGACAAGGATGCCCACTTTCACTATTCTTATTCAGCAGAGTAAGAAAAGTCCTCACCAGAACAATTAGGCAA
GTAAAAGAAAAAGGGTATCCAAATTGGAAAAGAGAATGTCATATTGTCCTTTGCAGATGGCATAATCTTATACATAGAAAAACCTAAAGATTCCACCAA
AATCTTTTAGAACTGGTAAAGGAATTCAATAAAGTTGCAGGATAAAAAAATCAACAAACAAAAATTAGTAGGTGTGGACATGATGGCTCATGCTTGTAA
TTTCAGCACTTTGAAAGGATGAGGCGGGTGGATTGCTTGAGCCCAGGAGTTTGAGACTGTCCTGGGCAACATGGCAAAACCTCATCAGTACAAAAAT
GAGCTGGGCGTGGTGGCCTGCACCTGAAGTTCAGCTACTCAGGAGGCTGAGATGGGAGGATCACTTGAACCTCGGAGGTGAAGGTTGCAGTGAGCA
GAAATTGCACCATTGCACCCCAGCCTGGGCAACAGAGTGAGACCCTGTCTGCACTCCCCTGCCTCCCCCAAAAAATCAGTAGCATTTCTGTGAAACAG
CAAATTAGCCAAAAGAGAAATGAAGAAAGCAATCCCATTTGCAATAGCTACAAAAAAAAGCAAAGCTTAACCAAGGAGGTGAAAGATTTCCACAATG
AAAACTATAAAATACTGATAACAGTAATTGAAGAGGCTACAAAATTTTTTGAGACATCCCATGCTCATGGATTGAAAGAAAAATGTTAAAATGATCATAT
TTACCTATAGCAATATACAGATGCTATGAAATCCCTATCCAGATATCAGCAGGGGCTTCAAGATGGCTGACTAGAGGCATCTGGCACTCACCTCCTCCAC
AAAATATAGCAAGTAGGTAACCACAGTTTATATAGATCGCCTCTGAGAGAATGCTGGAATTCAACTGAGAAGTAATAAGAAAAACCCAATGCAAGGGAT
AAGAGGGGAGTCAGGCAGTCTGCTTCTATGGGACTGGCTGGGATCATGAAGAGACTCCTCGATGTGCAGAAAGGTAAGTAAGTGAGCCCCTAGTGGT
CCACATTCCCACAGCAGACTCCTGAAATCCTAGTCAGAGAACCCCCTGACCCATAGATGCCTTGAGACTAACAAGAGGCTACCTACAGACTGTGAAAT
GGCAATGAACAGCTCCCAGAGAGGGAGATCACCCTGAATCCTCAGGCAATTCACACATCCCCTGAATCCTCAGGCAACTTCAGGAAAGTCCCATTTTG
ATAGCCCAACCCCCACCAGACTGCATCTGCTGTGGGCCCCACATCCCTCTATCTCATCCCTGGAGCCCCATGGACATCCCTTACACAAAGCTGGTTGCC
ACTCCTGACTGCTCCCTCCAGGCCAAAGCATGAGCCATTGGCAATACTCTGCTACCTCCGAAGCAAGGCTGCCATGAGGAAAGGTGACCCTGCCCATA
GTCACCACCTGGGGCCGAAGCATGCATTGCCCAACTGCCTGTTTATAGCTGCTGCCACTGAAAGCAATCCCACTCAACTTCCCCAGGAGTAGGGCTGC
AATAGAGTTGCTGCTGCCCCAACATGAGCATTCAGCAGGGTATACAGGGATCTTCCTGTGCCTGCCTACCACAGTCAGTACATTCTTGCATTACTGGAG
GGCCTCAGGACAGGCCCACCTGGCCTGGTCCCACCCTCTTTTCTCCCAGTGCCAGCGCATACCATCTCTGGGCCTAGGGATCACCCTGCCCTATCCAGT
ATTGTTGGCACCTGAGCACTCCTGGGGGCTTGAGGACTGCCCTACCCAACTGACCATTACCACCACAACTGGCATTCACTTGAATTTGCCACCTGTGG
GCCTTGGGACAAGCTCATCCAGCTTATCCCAGCCATCCCAATACCAGCATGGACACCTTGGGAGGCAGAAGATTGTTCTGCCACTGCTACTGCCATTGC
CCAAGCCATATCTGCTGCTCAGAGCTCAAGGACCTACCCACCCTGTGGCCCAGCTCTGTCATCATTGGCACCTGAGCAAGTTGCCTGGAGGCCCAAAA
TTGGTTTCCCTGGACTTGCTAACACTGGTACCAGCATACACTACCATTGGGCCCAAGGATAAGCATTCGGGGCCAGCCACTGCTGCCACTGGGGCCTG
AGGACTGGCCCAGCTGGTGTCCCTATCCCCAGCCAGACTTTACCACAGCCTCCACCAACAACTGCATCCTAAACTACAGAGGAACTCCCAGACACTAC
TGGTGCTGTTTACAGCTGAAAAAAATTCCATGGAGATCACACTACTGCAGGCACCCAGAATCAAAACCAAAGTGCCCTGCCCAAAGAACACCATAGAT
ATATCTTCAGGAACAAGACTCTCTTAGGAAAGACAGATCAAAAAATTGGAAGAAACTACTGTTACACCAGATGTGCAGATACCAGTGTAAGCACACAA
GAAACATGAAAAGGCAAAACATGACAACTCCTAAGGAATGCAATAATTCTCCAACAGATTCCAGTGAAAAAGAAATTGATGAAACCTTGAAAAATTC

AATATAATATTAAAGAAGATCTGATAGACTATACAAAGAAATCAGAAAAAACAATTCGGGGTATGAATGAGAAATTTGCCCAAGAGATAGATATAAAAA
GAACCAAACAGATATTCTGGAATTGAATAATAGATTGAATGAAATAAAAAACATATTTGAAAGCTTCAATAACAGACCACAACAAGCAGAAGAAAGAA
TTTCACACCTTGAAGATAAGTCTTTTGAAATGACTCAGACAAAAATATAGAAAAAATAGAAAAGAATGAACAAAGGCTATGTGACTATAGGATGCCATA
AGGTGACACCTTCAGTGTCCCAGAAGGTGAAGAGCAAACCAAAAGATTATAAAACCTATTTAATGAAATAATACCTGAACACTTCCCAATCTCCCAAG
AGATTTAGATCCAAATATAGGAATCTCAGAACCCTAAATAGATACAATTCAGAAAGGTCTTTTCCACAGCACATTATAGCAAATTGCCCATCCCAAGACA
AAGAGAGAATCATAAAACCAGCGATAAAAGAGGTTGTAGACACTTACAAGGGAACAACCTGCAGACTAACAGATTTTTCAGCAGAAACTTTACAGGC
CAGGAGGATGGGATTATATACGCAGAGTGCTGAAAGAAAAAACAAAACCAAAAACCTGCCAGCCAGGAATACTGTACTTAGCAAAGTTATATTCGTCA
GAAATGAAGGAGAAATAAAGTATTTCCCTTAAAAGTAAAAGCTCAGGGAATTAATTGCCACTACACTGGCACTACAAAAAATGCTTAAGGGAGTCTTA
CAGGAAGAAGTGAAAGGACAACTTCATGAAAGCACACAAAATAATCAGAAATCAATTAATGACAGGAATAAGCCCTCACATATCAACAATAACCTTGC
ATATAAATAGATTAAACTTTCTACTTAAAAGACAGACTGGCTGAATGGACTTAAAAACATGACCCATCTAAATGCTATCTACAAGAAACGCATCTCACCT
GTAAAGACACATAGTTGAAAGAAAGGAATGGGAAAAGATATTACATGCAAAAGGAAACAAAAACCAAACAGGAGTAGCTATACTTTCATCAGATAAA
ACAAAGGTTAAGCTAAAAACACTAAAAAGAGACGAAGTCATTATATAGTGACAGAGGGATCAATTGACCAAGAGGATATAACAATTCTAAACATATATG
CACCCAACACCAAAGCACCCAGATATATAAGGCAAACATCAAATCTGAAGGGAAAGGTAGACTTCAACACAATAATCTTGGGAACTTCAATTCTCCAT
TTCCAGTATTAGACAGATCATCTAGACAAAAAATTAACAAAGAAACATTGGATTTAAACTGCACTTTATACCAAATGGACCTAACAGACATTTACAGAC
CAGTTCATAAAACAGCTGCAGAATATACACTCTTCTCATCAGTACATGGAACACTCTCCAGGATACATTATATGTTAGGACACTAAGTATGATTTTAAATA
TATATATATTTTAAATTGGATTCATATCACATATCTTCTCAGACCACAATAGCATAAAACTATAAATAAATAAGAGGAGCATTCAAAACTGTATAAATAAAT
GGAAATTAACATGTTCCTGAATGACCAATGGGTCAAAGAAGAAATTAAGGCAGAAATAAAAAATTTACTGAAACAAGTGAAAATAGAAGCACAACAT
TCTGAAACTTAAGGGATACTGCAAAAGCAATGCTAAGAGGAAAGTTTATTGTAATAAATGCCTCCATTAAAAAAGGTAGAAAAGATCTTAAATAATCTA
ATGATACACCTGAAGGAACCAGAAAAGCAAGAACAAACCATACTAAACATAAAATTAGTAGAAGGAAAGAAATAACGAAGATCAGAGCAGAACTAA
ACAAGAAACTAAATAAATATAAAGGATCAATGAAATGCAAAATCGGTTTTTGAAAAGAAACAAAATAACCAGTGGCTAAACTATCCAAGAAGAAAAG
AGAGAAGACCTACATGAATAAAATCAAAAATGAAAAAGGGGATATTACAACTCATACCATGAAATACAAAAATCAAAACTCTTCAGTCTGATCAATAAA
TTGATTACAGTTACAGGATTTAAAAATCAGGCTTATTTCTATATACCAATAAAGAACCAGCTGAGAAAGAAATCAAGAAGGCATTATCTTTTACAACCAC
TACAAAAAATCTAGGAATGAGTTTAACCAAAGAGGTGAGAAACCTCTAACAAGGAAAACTACAAAAGTCTGATGAAAGAAATTGAAAAGAACACAC
AAAAAACATCCCATTCTCATGGATTGGGAGAATTAACAAAATTAAAATGACCATACTACCCAAAGCAATCTACAGATTCAATGCAGTCTCTCTCAAAAT
ACCAATGCCATTTTTCATGGAAATAGAAAAAATCCTAAAATTCAGATGGAACCAAAAAAGAGCTGGAATAGCCACAGCAATTCTGAGCAAAAATAACA
AAGTTGGATGCATCACACTGCCTGATTTAAAAATATAAGGCTATAGCATTAAAAGCAGCATGGTATTGGAATAAAAACTGATAGACCAATGGAACAGAG

TAGAACCTGGAAGTAAATCCATGTATTTACAGCCAACTGATTTTTGACAAAGATGCCAAGAACATACACTGGGAAAAGAGCACCCTCTTCACTTAATGG
AGCTGGGAAAATAAGATATCCATGTGAAAAATGAAACCGTTTCTATCTCTTGCTATACTAAAATCAACTCAAGATGGGTTAAAGACTTAAACATGAGGC
ATGAAACTATAACATCACTATTAATGAAGGAGACATTGGGGAAACACTTTAGGACATTGGTCTAGGGAAAGATTTTATGGATAAGACCTCAAAAACACA
GACAAAAACAAAAATAGACAAATGGGACTATATTAAATGATAAAGCTTGTACACAACAAGGGAAACAATCACCAGAGTGAAGAGACAGCCTGTTGAA
TGGGAGAAAATATTTGCAAACTACTCATCTGACAAGGTACTAATACCAGAATATACTAGGAACTCCAATAACTCAATAGTAAACAACAAATTAATTCCAA
TAAAAAGTGAGCAAAAGACATGGATAGATATTTCTCAAAAGAAAACATACAAGTAGCCAACAGGTATATTTAAAAAAAAAAGCCCAACATCAGTAATT
ATAAGGGAAATGCAAGCCAAAACCATAGTGAGAATGGCTATTTTAAAAAGACAAAAATTAACCAATGCTGGAGAGGATGTGGAGGGAACTCTTATACA
CTGTTGGTGGGAATGTAAATTAATACTATAGAAAACAGTTTGATTTCTGAAAAACCTAAAAAGAGAAATATGACATAATCCAGCAATCTCACTACTGGAT
ATTTATCTAAAGGAAAAGATATCAGTATACTAAAAATGTACCTGCATCCCCGTGTTTATTGCAGCACTATTCACAACAGCAAAGATATGGACTCAGGAAC
CTAAGTGTACATCAGTGGATAAATGGAAAAAGAAAATGAGGTAAATATATACAATGGAATACTAATTGACCAAAGAAGAATGAAATCATGTCCTTTGCA
GCAACATGAATGGAGCTGGAGGTCATTATTTTAAGCCAGGCACAGAAAGATAATTATCTTATGTTATTATCATATGTGGGAGCTATAAAAGTTGATCTCAT
GGAGGTAGAGAGTAGAAGGATAGATACCAAAGGCTGTGAAAGGTATGTCTGGAGGGTGAAGAGAGGTTGGTTAGTGGGTAAAAATACACAGCTAGTT
AGAATAAATAATTTATAATGTTTGATAAGAGTTGGTTGACTATATTTAACAATATAATGTATATTTCAAAATAAAAAAGAGGACTTGAAATGTTCCTAACTA
TAGAGATGATAAATTGAGGGTGATGGATACCCTGACTTGATCATTACACATTCGATGCATGTAACAATAGCACATGTACCCCATAAGTATTATGTATCGGT
CAAAAAAAAAAAAAAAAAGGAAATCCCAGTGCTTTGGGGGGCCAAGGCAGAAGGATCGCTTGAGCCCAGGAATTCAAGACCAGCCTGGGCAACAATG
CACGACCCTGTCTCTACAAAAAATTAAGAAGTTATCCAGGCATGGTGGTGCATGCCTGTAGTCCCAGCTACTTGAGGGGTCAAGGATGGAGAATGACT
TGAGCCTGGGAGTTAAAGATTGCAGTAAGTGGGGTGGTTCCAAGATGGCCGAATAGGAGGAGCTCCAGTCTATAGCTCCCAGCGTGAGTGACGCAGA
AGATGGGTGATTTCTGCATTTCCAACTGAGGTAGTGGGTTCATCTCACTGGGGCTCGTCAAGACAGTGGGTACAGGACAGTGGGTGCAGCCCACTGAG
TGTGAGACGAAGTAGGGCGAGGCATCGCCTCACCTGGGAAGCACAAGGGGTCAGGGAATTCCCTTTCCTAGCCAACGGAAGGGGTGACAGACGGCA
CCTGGAAAACTGGATCACTCCCACCCTAATACTGTGCTTTTCCAATGGTCTTAGCAAACGGCACACCAGGAGATTATATCCCGCGCCTGGCTCGGAGGG
TCCCACGCCCATGGAGCCTCGCTCATTGCTAGCACAGCAGTCTGAGATCGAACTGCAAGGCGGCAGCGAGGCTGGGGGAGCCTGCCATTGCTGAGGC
TTGAGTAGGTAAACAAAGTGGCCAGGAAGCTCGAACTGGGTGGAGTCCACCACAGCTCAAGGAGGCCTGCCTGCCTCTGTAGACTCCACCTCTGGGG
ACAGAGCATAGCCGAACAAAAGGCAGCAGAAACCTCTGCAGACTTAAATGTCCCTGTCTCACAGCTTTGAAAAGAGTAGTGGTTCTCCCAGCACGGA
GTTTGAGATCTAAGAATGGATAGACTGCCTCCTCAAGTGGGTCCCTGACCCCCGAGTAGCCTAACTGGGAGGCACTCCCCAGTAGGGGCAGACTGACA
CCTCACACGGCTGGGTACTCCTCTGAGACGAAGCTTCCAGAGGAACAATCAGGCAGCAACATTTGGTGTTCAGCAGTATTCGCTGTTCTGCAGCCTCT
GCTGCTGATACCCAGGCAAACAGGGTCTGGAGTGGACCTCCAGCAAACTCCAACAGACCTGCAGCTGAGGGTCCTGACTGTTAGAAGGAAAACTAA

CAAACAGAAAGGACATCCACACCAAAACTCCATCTATACGTCACCATCATCAAAGACCAAAGGTAGATAAAACCACAAAGATGGGGAAAAAACAGCA
GAAAAGCAGAAAATTCTAAAACTCAGAGCACCTCTCCCCATCCAAAGGAACGCAGCCACTCGCCAGCAATGGAACAAAGCTGGACGGAGAATGACT
TTGACAAGTTGAGAGAGGAAGGCTTCAGACAATCAAACTTCTCCGAGCTAAAGGAGGAAGTTCGAACCCAAAGCAAAGAAGCTAAAAACCTTGAAA
AAAGATTAGACGAATGGCTAACTAGAATAACCAGTGTAGAGAAGTCCTTAAATGACATGATGGAGCAGAAAACCATGGCACAAGAACTACGTGACAA
ATGCACAAGCTTCAGTAGCCGATTTGATCAACTGGAAGAAAGGATATCGGTGATTGAAGATCAAATGAATGAAATGAAGTGAGAAGAGAAGTTCAGA
GAAAAAAGAGTAAAAACAAACGAACAAAGCCTCCAAGAAATATGGGACTATGTGAAAAGACCAAATCTACATCTGACTGGTGTACATGAAAATGACA
GGGAGAATGGAACCAAGTTGGAAAACACTCTTCAGGATATTATCCAGGAGAACTTCCCCAACCTAGCAAGGCAGGATATTATCCAGGAGAACTTCCCC
AACCTAGCAAGGCAGGCCAACATTCAAATTCAGGAAATACAGAGAATGCCACAAAGATACTCCTCAAGAAGAGCAACTCCAAGACACATAATTGTCA
GATTCACCAAAGTTGAAATGAAGGAAAAAATGTTAAGGGCAGCCAGAGAGAAAGGTTGGGTTACCCACAAAGGGAAACCCATCAGACTAACGGCGG
ATCTCTCAGCAGAAACTCTACAAGCCAGAAGAGAGTGGGGGCCAATATTCAACATTCTTAAAGAAAAGAATTTTCAACCCAGAATTTCATATCCAGCC
AAACTAAGCTTCATAAGTGAAGGAGAAATAAAATCCTTTATAGACAAGCAAATGCTGAGAGATGTTATAACCACCAGGCCTGCCCTACAAGAGCTCCT
GAAGGAAGCACTAAACATGGAAAGGAACAATTGGTACCAGCCACTGCAAAAACATGACAAATTGTAAAGACCATAGATGCTAGGAAGAAACTGCATC
AACTAACGAGCAAAATAACCAGCTAACATCATAATGGATCAAATTCACACATAACAATATTAACCTTAAATGTAAATAGGCTAAAGGCTCCAATTAAAAG
ACACAGACTGGCAAATTGGATAAAGAGTCAAGACCCATCAGTGTGCTGTATTCAGGAGACCCATCTCACGTGCAGAGACACACATAGGCTCAAAATAA
AGGGATGGAGGAAGATCTACCAAGCAAATGGAAAATAAAAAAAGGCAGGGGTTGCAATCCTAGTCTCTGATAAAACAGACTTTAAACCAATAAAGAT
CAAAAGAGACAAGGTCATTACATAATGGTAAAGGGATCAATTCAACAAGAAGAGCTAACTATCTTAAATATATATGCACCCAATACAGGAGCACCCAGA
TTCATAAAGCAAGTCCTTAGAGATCTACAAAGAGACAGACTCCCACACAATAATAATGGGAGACTTTAACACCCCACTGTTAACATTAGACAAATCAAC
AAGACCGGAAGTTAACAAGGATATCCAGGAATTGAACTCAGCTCTGCACCAAGTGGACCTAATAGACATCTACAGAACATTCCAACCCAAATCAACAG
AATATACATTCTTCTCAGCATCACATCGCACTTATTCCAAAATTGACCACATAATTGGAAGTAAAGCACTCCTCAGCAAATGTAAAAGAACAGAAACTAT
AACAAACTGTCTCTCAGACCACAGTGCAATCAAACTAGAACTCAGGATTAAGAAACTCACTCAAAACCGCTCAACGACATGGAAACTGAACCACCTG
CTCCTGAATGACTACTGGGTACATAACGAAATGAAGGCAGAGAAATAAAGATGTTCTTTGAAACCAATGAGAACAAAGACACAACATACCAGAATCTC
TGGGACACATTTAAAGCAGTGTGTAGAGGGAAATTTATAGCACTAAATGCCCACAAGAGAAAGCAGGAAAAATCTAAAATTGATGCCCTAACATCACA
ATTAAAAGAACTAGAGAAGCAAGAGCAAACACATTCAAAAGCTAGCAGAAGGCAAGAAATAACTAAGATCAGAGAAGAATTGAAGGAGATAGAGAC
ACAAAAAACCCTTCAAAAAATCAATGAATCCAGGAGGTGGTTTTTTTGAAAAGATCAACAAAATTGATAGGCCTCTAGCAAGACTAATAAAGAAGAG
AGAAGAATCAAATAGATGCAATAAAAAATGATAAAGGGGATATCACCACCGATCCCACAGAAATACAAACTACCATCAGAGAATACTATAAACACCTCT
ATGCAAATAAACTAGAAAATCTAGAAGAAATGGATAAATTCCTGGAGACATACACCCTTCCAAGACTAAACCAGGAAGAAGTTGAATCCCTGAAAAGA

CCAATAACAGGCTCTGAAATTTAGGCAATAATTAATAGTCTACCAACCAAAAAAAGTCCAGGACCAGACGGATTCACAGCCGAATTCTACCAGAGGTA
CAAGGAGGAGCTGGTACTATGCCTTCTGAAACTATTCCAAACAATAGAAAAAGAGGGAATCCTCCCTAACTCATTTTATGAGGCCAGCATCATCCTGAT
ACCAAAGCCTGGCAGAGACACAACAAAAAAAGAGAATTTTAGACCAATATCCCTGATGAACATTGATGCAAAAATCCTCAATAAAATACTGGCAAACC
GAATCCAGCAGCACATCAAAAAGCTTATCCACCATGATCAAGTGGGCTTCATCCCTGGGATGCAAGGCTGGTTCAACATATGCAAATCAATAAACATTA
TCCAGCATATAAACAGAACCAAAGACAAAAACCACATGATTATCTCAATAGATGCAGAAAAGGCCTTTGACAAAATTCAACAGCCCTTCATGCTAAAA
ACTCTCAATAAATTAGGTATTGATGGGATGTATCTCAAAATAATAAGAGCTATTTATGACAAACCCACAGCCAATATCATACTGAATGAGCAAAAACTGG
AAGCATTCCCTTTGAAAACTGGCACAAGACAGGGATGCCCTCTCTCACCACTCCTATTCAACATAGTGTTGGAAGTTCTGGCCAAGGCAATCAGGCGG
GAGAAAGAAAGAAAGGATATTCAATTAGGAAAAGAGGAAGTCAAATTGTCCCTGTTTGCAGATGACATGATTGTATATTTAGAAAACCCCATCATCTCA
GCCCAAAATCTCCTTAAGCTGATAAGCAACTTCAACAAAGTCTCAGGATACAAAATCAATGTGCAAAACATCACAAGCATTCTTATACACCAATAACAG
ACAAACAGAGAGCCAAATCATGAGTGAACTCCCATTCACAATTGCTTCAAAGAGAATAAAATACCTAGGAATCCAACTTACAAGGGATGTGAAGGACC
TCTTCAAGGAGAAGTACAAACCACTGCTGAACAAAATAAAAGAGGATACAAACAAATGGAAGAACATTCCATGCTCATGGATAGGGAGAATCAGTATC
ATGAAAATGGCCATACTGCCCAAGGTAATTTATAAATTCAATGCCATCCCCATCAAGCTACCAATGACTTTCTTCACAGAATTGGAAAAAAACTACTTTA
AAGTTCATATGGAACCAAAAAAGAGCCCGCATTGCCAAGACAATCCTAAGCCAAAAGAACAAAGCTGGAGGCATCACGCTACCTGACTTCAAACTAT
ACTACAAGGCTACAGTCACCAAAACAGCATGGTACTGGTACCAAAACAGAGATATAGACCAATGGAACAGAACAGAGCCCTCAGAAATAATACCACA
CATCTACAATCATCTGATCTTTGACAAAGCTGACAGAAACAAGAAATGGGGAAAGGATTCCCTATTTAATAAATGGTGCTGGGAAAACTGGCTAGCCAT
ATGTAGAAAGCTGAAACTGGATCCCTTCCTTACACCTTATACAAAAATTAATTCAAGATGGATTAAAGACTTACATGTTAGACCTAAAACCGTAAAAAC
CCTAGAAGAAAACCTAGGCAATACCATTCAGGACATAGGCATGGGCAAGGACTTCATGTCTAAAACACCAAAAGCAATGGCAACAAAAGCCAAAATT
GACAAATGGGATCTAATTAAACTAAAGAGCTTCTGCACAGCAAAAGAAACTACCGTCAGAGTGAATAGGCAACCTACAGAATGGGAGAAATTTTTTGC
AATCTACTCATCTGATAAAAGGCTAATATCCAGAATCTACAAAGAACTCAAACAAATTTACAAGAAAAAACAAACAACCCTATCAAAAAGTGGGCAAA
GGTTATGAACAGACACTTCTCAAAAGAAGACATTTATGCAGCCAAAAGACACATGAAAAAATGCTCATCATCACTGGCCATCAGAGAAAGGCAAATCA
AAAAACCACAATGAGATACCATCTCACACCAGTTAGAATGGCAATCATTAAAAATTCAGGAAACAACAGGTGCTGGAGAAGATGTGGAGAAATAGGA
ACGCTTTTACACTGTTGGTGGGACTGTAAACTAGTTCAACCATTGTAGAAGACAGTGTGGTGATTCCTCAAGGATCTAGAACTAGAAATACCATTTGAT
CCAGCCATCCCCTTACTGGGTATATACCCAAAGGACTATAAGTCATGCTGCTATAAAGACACATGCACACGTATGTTTATTGCAGCACTATTCACGATAGC
AAAGACTTGGAACCAACCCAAATGTCCATCAATGATAGACTGGATTAAGAAAATGTGGCACATACACACCATGGAATACTATGCAGCCATAAAAAAAG
GATGAGTTCACGTCCTTTGTAGGGACATGGATGAAGCTGGAAACCATCATTC
TCAGCAAACTATAGCAAGAATGAAAAACCAAACACCGCATGTTCTCACTCGTAGGTGGGAACTGAACAATGAGAACACTTGGACGTAGGAAGGGGAA

CATCACCCACCAGGGCCTGTTGTGGGGTGGGGGGGGAGGGGGGAGGGGGGAGGGAAAGCATTAGGAGATATACCTAAGGTAAATGACAAGGTAATCA
GTGCAGCACACCAACATGGCACATGTATACATATGTAACAAACCTGCACGTTGTGCACATGTACCCTAGAACTTAAAGTATAATAAAAAAAAGGGAGA
GAGAGAGAAAAGAGAAATCTAAAAAAAAAAAAAATACCAAAGACAATCTTCACCGAAATAGCAAAAACACAATCCTTAAACTTGCATGGAACCACAA
AAGACCACAAATAGGACAAACAATACTGAGCAGGCAGAATGAAGCTGGAGGCACTACACTGCCTGACATCAAAATACAGTCGACTCTTCAACAACAT
AGATTTGAACTGTATAGGTTCATTTATACGTGGATTTTCCCCATAATCTAACAAGGATAGAATGCAATATTCACAAAAGTGAAAACTATGTATACAGATGA
CTGACATTTTGTATATGCGCATTACACAGGGCTGACTCTGGGACTTGAATATACATGGCTTTTGGTATATACAGGGGGTCTCGGAAGCAATCCCCCTTGTA
TACAAAACAATGGTAACCAAATTAGCATGCAATGGGCATAAAACAGACATATAGACCAATGGAACAGAATAGAGAACCCAGAAGTAAATAAATTTATTT
ACAGACAACTGGTTTTTGACAAAGGTGCCAAGAACACACTCTGGGGAAAGGACACCATGTTGAACACATCATGTTGGGAAAACAGGATATCATATGC
AGAAGAATGAAACTATACTATCTCTCAGCATATAAAAAAATCAACTCAAAATTGATTAGAGACTTAAACATGAGACCTGAAGCAAACAGTACTAGAAG
AAAACAGAAGGGAAACCCTTCAGGATATGGGTCTAGCGAAGATTTTATGGGTAAGACTTTATAAGCACAGGCAACAAAAACAAAAATAGACAAATGG
GACTGTATCAAACTAAAAACCTACACATAAAAGGAAATAATAAACTATTAAGAGATATCCTGTAGAATGGGAGAAAATATTCATCTGACAAGGGATTAAT
ATCCGGAATATACAAGGAATTCAAACAATTCAACAGCAAAAAACACCAGATTATCTGATTTAGAAATGTGCAAAATAGAACAACTGGCCAACAGGTAT
GTGAAAAAATGCTCAACATCTCTCATCACAGAAATGCAAGTCAAAGCCACAGTGAGACATTATTTCACCTCAGTTAGAATGGCTGTTTTCAAAAAGAC
AAAACATAACAAATGCTGGCAAGGATGCAGAGAAAAGGGAAATAATACACTATTTGTGGGACTATAAATTAGTGAGTCATTATGGAAAATACTGTGGTG
TTTTGTCAAAAAAACTAAAAGTAGACTATCATACAGTTTGACAATCCACTACCGGGTATTGACTCAAAGGAAAGGAAATCAGTATATAAAAAAGATATT
TGCATTCTCATGTTTATTGCAGTACTGTTCACAACAGTTAAGATATGGAATCAACTTAAATCTCCATCAGCAGATGCAGAGAAAGAATGTGGTATGTATA
CACAATGGAATATTATTCAGTCATAATAAAGCATTAAGTCCTGTCATTCACAGCAACATGGATGAACTTGGATAATATGTTAAATGAAATTAGGCACAGA
AAGATGGAGGATTTTGACTGCTCACAAAACAGAGAAATGATATTTGAGTTGATGAATATGCTAATTACCCTGATTTGATTATGACACATTATATACATGTA
TCACAATATCACTGTGTATATCCCATAAATATCTACAATTATTACATGTTGACTAAAAATAAAAGGACAAAGAAAAAGACTCCTAGAAGAAAAACCCAC
AAGGAGCATCATAATGGTGAAAAGACTAAGAGCTTTTCCTCTAAGATCAGGAATAAAGCAAGATGCGCATTTTAGCCATTATATTAAACATAGTAGTGG
CTTTCTAGCCAAAGAATATGGAAAAGAAATAAAGGCATCATTTAGAAAGGAAGAATTAAAATTATGTTTGTTCATAGATATGATCATATTTGTAGAAATC
CCTGAAAACTCACCACAAAAACTGAATAAATTAATTCAGCAAAGTAGCAGGATAGTAAGTCAGTACACAAAATTCAGTTGCATTTCTTTTTTTTTTTTTT
TTTTTTTGAGGCAGAGTCTTGGTCTGTCGCCCAGGCTAGAGTGTAGTGGCGCGATCTCGGCTCACTGCAAGCTCTGCCTCCCGAGTTCACGCCATTCTC
CTGCTTCAGCCTCCCAAGTAGCTGGGACTACAGGCACACGCTGCTATGCCTGGCTAATGTTTTTGTATTTTTAGTAGAGATGGGGTTTCACCATGTTAGC
CAGGATGGTCTCGATCTCCTGACCTTGTGATCTGCCCTCCTCAGCCTCCCAAAGTGCTGGGATTACAGGCGTGAGCCACTGCGCCCAGCTTTCAGTTGC
ATTTCTATACATTAATCACAAACAATCTGAAAGGATATTATTAACACAACTCTATTTACACGACAGCATCAGGACACTCAGCCTTGGCCACCAAGGACC

TTTGTAATGTTTGCCACTGCTGACCTCAGCTGATGGAGCTGCAAGAAGAGTATTCCATTATATCCTCATCTGGGCTACAGCTGCTGCACCCTACCTACTT
AACACTCCATCAAGTGAGCAGATTTCAATTCATTGTAACAAGAAACAGAAAAACCAAGGAAACATAGCACCCACCTGAGAAATACAATAATTTTCAGG
GACTGACTCCCAAGAAATTGGGATACATTAATTGCCTTACAGAAAATTAAAAACGACTGATTTAAGGAAGTTCAGTGTGCTACCAGAGAACAGAGAGA
CAACCTAATGAAATCATTAATACACAAAAGGAGACATTCAATAAAGAAATAGAAATCCTAAAAAAAAAACAAACATTTTGGAGGTGAAGAATATAGTG
CATGAAATGAAAAGTGAAATAGAAAGCATCAGCAGCAGACTTGATCAAATAGAAGGAGGAATTCATCAACTGGAGGATAAATTATTTGAAAATAACCA
GTAGAGGAGAAAAATAAAGAATGAAAAGGTAACAAGGGAAGTTTACAGGATTTATGGGACACCATCAGGAGAGTTAATGTTCACACAGGATTTATAGA
AGGAAAGTAGAGAGGTAAAGGGTAGAAAGTTAATTCAGAGAAATAATGGCTGAAAACTTCCCATATCTGGGGCTCTTCCCAGATTGATGGACATCTAG
GTACATGAAGCTCAAAGAATTACAATTAGTTTCCATCTAAAAGAGGTTTCACCTAGATATATTGCCATCAAAATGTAAAAAATGGAAGACAAGATAATTT
TGAAAGCGGCAAGATAAATAAAGTTCTTACATCTGCAGGAACCTCCATAAGGCTATCAGTGGATTTCTGAGCAAAAACTTTCCAGGCCAGGAAACAAT
AGGATTATATATTCAAAGTTCTGAAAGATAAAAAACTGCCATCTAAGAATACTTTACACAGCAAAGCTGTCCTTCAGATATGAAACAGATACAAGACTT
CCTCAGGTAAACAAAAGTTGAAGGAGTTTATCAGCAACTACTCTTGCCTTACAAGATATGCCAGAGGGAGTTATTCAGGCTTAAATGGAAGGATGATAA
TTAGTAACATGAAAACATATGAAAGTACAAAATTCACTGATAAATAAATATACGGGCAACTTAAGAATACTCTAATAGTGTAATGGTAGTATATAAATCAC
ATAACTCTAGTATAAAGGTTAAAAGAAAAATAATTAAAAATTACTATTGTTTTCATAATTTCTCAATGGATATGCAATATTAAAAGGTGTACCTTGGGGCA
TCAAAAATAAAATGTAGCGGGGGAAGTATAAGATCAAAATATTCCCCCCAAACAAAGAGGGAACAATCCCAACTTATTTTACAAGGCTAGCATTGCCCT
CATACTAAGGCCAGACAAGGACAATATAAGAAAAGAAAATAACTGGCTGATATTTTTTTATGACTATACATTTAAAAATTTTCAACAAAATATGATCAAA
CTGAATTTAACAGCACATTAAAAAAATCATATATCACAAACATGGGATTTATCCTTAGTACATAAGGACAGTTAAGTGTACATATACAAGAAATGGGATA
CTTTAATAAAATCAAAGATAAAAATCATACAATCATTTTGATAAGTGCAAATAATGCAGTTCACATAATTCAACATCCTTCCATGATAAAAACTATGAACA
AATTAGGTATAGAAGGAATGTACTGCAACACAATAAAGGCCATATATGAATAGCCCACAGCTAACATCATACTCAATGCTGAAAAGCTGAATGCCTCTCC
TCTAAGATGAGGAACAAGAAAAAGATATTCTCACCACTTCTATTCAACACAGTACTGGAAGTCCTAGCCAGAGGAATTGGGCAAGAAAAATAAATAAA
TACACTCAAATCAGAAAGAAGGAAGTAAAATTGTCTCTGTTTGCAGATGACATCATATATGTAGAAAATGCTACAGACTCCACAAAAAAAAACCTGTTAG
AAGTAATAATCTAACTCAGTAAAGTGGCAGGATACAAAATCAGCATTCAGAAGTCATTTGCGTATCTATACACTAATAATAAACAACCTGAAAAACAAA
TTAAGAAAACAATTTCATTTAAAATAGCATCAAAAAGAATAGAGAGCTCCCAAATAAATCCTTTCATATATGAACGCATGATTTCTGACAAGCATGCCAT
GTCCATTCAATGGCAAATGGATTCTCTTTTCAACAAATGGTGCCAGGAAAACTGGATATCCTCACGTGAGAGAATGAAGTCGAGACCTTTACCTAACTC
CATATACAAAAATTAACTCAAAGTGGACCACAGACCTAAATGTAAGACCTAAAACTATAAAACTCTTAGAAGAAAACATGGGGCAAAAACTGCATGAC
ATTGGATTTGGTATTGACTTCATTTGGATATAATATGAAAGGCACAGATAAAATAAAAAATAGAGAAATTGGACTGCATAACATTTAGGCATACATTTAAC
CAAGGAGATAAAAGACCTAGACACTGAAAACTATGAAACATTGAACAAATTCTAAGATGATACAAATAAATGGAAAGATATCTGGTGTTCATAGATCAA

TAGAATAAATATTGCCGAAATAGCTATACTACTCAAAGTGATCTACAGATTCAAGGCAATGCCTATCAAATTTCCAGTGACTTTTTTTCACAGATATAGAC
AAAAAAAATCCTAAAATTTGAAAGAAACTACAAAAGACCTTAAAGATCTAAAGCATACTTGAGCAATAAGAACAAAGTGAAAGATATTACACATGCTT
ATTTTGAATTATATTTCAAAGTTATGGTACTGGTATAGAAATAAACCAATGGAACAGAATAGAAAGCATAGAAATAAACCCACACATTTATGGTCAACTA
ATCTTTGACAAAGACACCAAGAGTACACAATGGGGAAAGGGTACTCTCTTGAATAAATGGCACTGTGAAACCAGGATATCCACATGCAAAAAAATTAA
ATTGGAACCTTACCCTACACTGTAGAGAAAAGTGAATAAGATTTAAACATATGACCTGAAACCATAAAACCTATAAAACAGGGAAAAGCTCCTTGCCAT
TGGTTTTTGTGACAATTTTTTAGATGTGACATCAAAAGCACAAGGAATAAAACGCAAAAATAAATTAGAACTACATCAAACAAAAAAGTGTCTGCACA
ACAAAGGAAACAACAAAATGAAAAGGCAGCACATGTAGCGGGAGAAAATATTTCCAACCATATATCTGATAAGCTATTAATATCCAATATATATAAGGAA
CTCATATAACTAGCAAAAAAACTTAATAACCCAATTAATAACCTGTTAAGATGGACAAGGGACTGAATAGACATTTATTCAAAGAAGACATACAGATGG
TCAACAGGTATATAAAAAGGTGCTTAACATCACTTATCATCAGGGAAATGCAAATGAAAACCACACTGAGATATCACCTCATAACTGTTAGAATCGCTAT
TATCAAAAAGTCAAAAGATAGCAAGTGTTGGTGAGGATGTGGAGAAAAGGTGTCCCTTATACATTTTTGTTAGGAATGTAAATTGATACAGTCATTATG
GAAAACACGATAGATTTTCTTTAAAAAATTAAAATTGGAACTGCTATATGATCCAGCAGTCTCAGTTCCGGGTATATCTAAGGAAAACAAAGTCAGTATC
TTGAAGAGGTATCTGCACTCCCATGTTCATTGTAGTGTTACAGTAGTCAAGAAATGAGCAACATAGTATTCATAGGCATGGTGGCTTGTACCTGTAATCC
CAGTACTTGGGGGTGGCCAAGGTGAAGGATAGCTTGAACACAGGAGTTTGAGACCAGCCTAGGAAACATAGTGATACCCTATCTCTACATAAAATTTTA
AAAATTAGCTGGGCATGGGGGTGCATGACTGTGGTCCCAGCTACTCAGGAGGCTGAGGCAGGAGGATCACTTGAGCCCATGAGGTTGAGGCTACAGT
GAACTGTGATTGTGCCACTGTACTCCTGCCTGGATGACAGATGGAGACCCTGTCTCAAAAAGAAAGGAAGAAAATAGTGTGTGTGTGTGTATCACCAA
ATTCCCAAATAAACACATCACATACATATACATACAGACACACACACACACATATATACACACACACAATGAAATATTCATCCTTTAAAAAGCAGGAAAT
CATGCAATTTATGACAACATAGATGAACCTGGAGGATATTATGCTAAGTGAAATAGCCAGACACAGAAAGACAAATACTGCACTATCCCAGTTACATATG
GAATTCTAAAAAGCTTGAACTCAGAGAAACGGAGAGTAGAAGGGTGGTTACTAGGGGTCAGGGAGTAGGGGAAATAGGAAATGTTGAAGGGTACAA
ACATAGAGTTATCAGATGAATAAGTTCTTGACATAACGTCCAGATTGGTGACTATAGTTAATATATTCTATACTTGGAATTTGCTAAGAGAGTATAGCTTAG
GTATTCTGACCACACACACGCACACACATGGCAACTATGTAAGGTATATGTTAATTGGCTTGATTGTAGTAATCATTTCACAGTAGGTCAAATATGTTCTT
AAATATATGCACTTTTAATTTGTCAATTATACCTCAATAAAAGCTGAAAAAAGCATCAAGAATAACAAACTTAGGAACCAACTTTACCAAGGAGGTGAA
AGTCTTGTACAATGAAAATTACAAAACAATGCTGAAAGAAATTAAAGTAGACATAAAGGGAAAACACCTCCTATGTTCATGGATTGGAAGGCCAATATT
GTTAAGGTGTCAGTACCACACAAAACAATCTACAAATCCAAGGTAATCCCAATAAAAATCCTAATGATGTTATTTGCAGAAAGAAGAAACCCCATCCTA
AAATTCACATGGAATCTCAAGGGACCTTGAATAGCTAAAACATTCTTGAAAATGATGAACAACATTGGAGGACTCACACTTTCTGATTTGAAAACTTAC
TACAGGCCAGGTACAATGGCCTACACCTGTAATCTCAGCACTTTTGAAGGCTGAGGTGGGAGGACTTCTGAGTCCAGGAGTTTGAGACCAGCCTGGG
GAACACAGGGAGACCTCATCTCTACAAAAAATAAAATAATTGGCCAGGTGTAATGGTACACACTTCTATTCCCAGCTACTCAAGAAGCCGAGGTGGGA

GGATTGCTTGAGCCTGGGAGATTGAGGTGGCAGTAAGCAGTGATAATGCCACTGTACTCCAGCGTGGGCAACAGAGTAAGAATGTCTGAAAAACAAA
CAAAAACCCCAACTTATTACGAAGCTACAGTAATCAAAACTATGTGGTATTGACATAAAGATGTAGAGACCAATGGAATTGAATAGAGAGCCCAAAAAT
AAACCCTTGTATACATGATCAAATGATTCCTGAGAAGAGTGCCAAGACCATTCAATAGGGAAAGTGCTGTATTTTCAACAAATGGTGCCAGGATAACTG
AAGTTGAGACCCTTACCTAATACCATACACAAAAATTAACTCAAGAGAATGAAGTTGAGATCCTTACCTAACCATGTATAAAAGAGAATGAAGTTGAGA
CCCTTACCTAACACTGTGTACAAAAACTTACTCAAAATGGGTCAAAGTCCTAAAACTATGAAGTCTTATAAGGAAACATAGGGGAAAAACTTTACATCA
TTGGCAGTGCTTCTTGTATGCGACACCAAAGGCACAGGCAATAAAAGTAAAAATAGATAAATTGTACTTGCACAGGCAATAAAAGTAAAACTAGATAA
ATTTTAAAAATTTACATTGTGTGCATTAAAATAATATCAACTAAGCAATAAGACAACCTACAGAATGGGAGAAAAATTTGCAAATCACATACCTGATAAG
GGATTAATGTCCAGATTATACAGAGAACTTCTAAAAGTCATCACCAAAAAACAATGCACCTGAAAAATGGGCAAAAGGGCCAGGCACGGTGGCTCAC
GCCTGTAATCCCAGTACTTTGGGAGGCTGAGGCGGGAGGATCATGAAGTCAGGAGATTGAGACCATCCTGGCTAACAGGGTGAAACCCCGTCTCTACT
AAAAATACAAAAAATTAGCCAGGCGTGGTGGTGGGCGCCTGTAGTCCCAGCTACTCGGGAGGCAGGAGAATGATGTGAACCCAGGAGGCAGAGGTT
GCAGTAAGCCGAGATGGCACCACTGCACTCAGCTTGGGCAACAGAGTGAGACTCCATCTCAAAAAAAAAAAAAAAAAGGGCAAAGGACTTGGACAG
ACGTATCTCCAAAGAAGATATACAAATGGATAATAGTACGTGAAAAGTTGTTTAACATCTCTAGTCATTAGGAAATACAAGTCAAAACCACAACCTTACA
TACACTCAGATGACTACTATTAAAAAAATAAACACAGCCTAGGCAACATAGTGAGACCCATCTCTAGTATCAGCTACGTGGGAGGTTAAGGTGGGAGGA
TAGCCTAAGCCCAGAGGTCTAGGTTGAGGCTGCAGTGTGCCATGATCATGCCACTGCATTCCAGCCTAAGTGACAGAGCCAGATCTTGTCTCAAAAAA
ATCCCACATAAACAGAAAATAACCACTGCTGGAAGGGACATGGAGAAATTGGAATCCTTGTGCACTGTTAGTGGGAATGCAAAATGGTACAGCCTCTG
TGTTAAACAGTTTGGTGTTCCTTCAAAAATTAAAAACAGAATTATCTGATCCATCAACTCCACCTCTGTGTATACACCCAGAAGGATTAAAAGCAGTCT
CAAAGATATATTTGTATACCCACGATTGTAGTGGCATTATTTACAACAGTTAAAACATCAATGCAACCCAAATGTCCATGGAAGAATGGATAAGCAAAAT
GTGGTATATACATACAATGTAATATTATTCAGCTTTAAAAAAAAATAGAAATTTTGACACATGCCACAACATGGATGAACCTCGAGGCCATTATGTTAAATG
AAATACAGCAGTCACAAGAAGAGAAGTACTGTATGTTTCCACTTATATGAGGTATGTAGAATAGTCAAATCAGAGACATAGAAAGTACAAGGTGGTTTC
CAAGAGATGGCGGTGGTGGGGGGGTTGGGGAATTATTGTTTAATGGATATAGAGTTTCATAATTACAAATGACAAGTGTTATGGAGATGGATGATAGTGA
TGATTGCACAGCATTGTGAACGTATCTAATACATTCACTGAATTGTACACTAAAATTGGTTAGGATGGTAATTTTTATGTTAAGTGTATTTTGCCACAATA
AAAGATTTGGAAATCAACAACAAATAGAAATCCCAGAAGATAACATAGGAAGAAATCTAGATGATCTTTAGTTTGGTGATGGCTTTTTAGATATGACAC
CAAAGGCATGATTCATGAAAGAAATAATTGATAAGCTAGACTTTGTTAAAAATAAAAGTTTCTGCTCTGTAAAACATACTGTCAAGAGGATGAAAGGAC
AGGCCACAGAAGGGAGACAATACTTGTAAAAGATATACCTGATAAAGGACTCATCCAAAATATATAAAGAACCTTAAAAACATAACAATTAGAAAACA
AACAATCTGATTACAAAATGGACTGAAGACCTTAACAGACATCTCACCAATGAAGATATACAGATGGCAACTAAGTATATGAAAAGATGCCCCACATCT
TACGTCATTAGGGAAATGGAAATTAAAACAAGAAATGTGATATATCTACACGCTTATTAGAATGGCCAAAATTCAAAACACTGACAATACCAAATACTTG

CCAATATGTGGGGCAACAGGAACACTCATCCATTGCTGGTGGGAATGCAAAATGGTACAGCCACTTTGACATATACCATTAATGGGAATGTAAAGTGGT
GCAGCCCTGGTGGAGAAGAGTTTGGTGGTTCTTCAAAGAATTAAAAATGGAATTATCATAGGATCCATCAATTCCACTTCTGAATGTGTATACATATCCC
CCGAAGAATCCAGAGTATATCGAAATCAGTGGTTTCTTATAAAATTAAACATATTCTTACCATGCAGTTCAGCAATCACATCCTTGGTATTTACTCAAAGG
AGGTGAAGACTTATATTCACATAAAAATCTGCACAGAGATGTTTACAGAAGCTTTATTTGTGATTGTTGAAATTGGAAGCAACCGAGATGTCCTTCCATA
GGTGAGTAGACAGATAAACTGTAGTACATCCAGGCAATGAAATATTATTCATCACTAAATAGAAATGAGCTATCAAGCCATGAAAAGATATGGAGGTAAT
TAAAAAACATATTACTATGTGAAATAAGGCAATCTGAAGAAACTACATACTGTATGATTCCAATCATATTGCATTTTAGAAAAGGCAATGCTATGGAGGC
AGTAAAAAGTTCAGTGGTTGCCAAGGGTTGTTGGGGAAGAAGGGTTGAATAGGCAGTGCACAGAGTACTTTTAGGGCAGTGAAAATGTACAAATATAT
GTAAATATACGTATCTGTATGATACTATAATGGTAGATACATGCATATTAGTCCGTTCTCACAGTGCTATAAAGAACTACCTGAGACTGGGTAATTTGTGAG
AAAAGAGGTTTAATTGACTCACAGTTTTGTAGGCTGTACAGTAAGCATGGCTGGCAGGCCTCAGAAACTTAAAATCATGGCGGAAGGCAAAGGAGAA
GCAGGCACATCTTTACCATGGCAGAACAGGAGAGGGAAAGAAAGTGAGATGGGAAGTGCCACACACGTTTAAACAACCAGATCTCATGAGAACTCA
CTATCATGAGAACAGCAAGGGGGAAGTTTGCCCCCCACCAGGCCCCTCCCCTGACACATGGGGATTACAGTTCAAGATGAGATTTGGGTGGAACAC
AGAGCCAAACCATATCAACATGTTACACATTTGTAGAAACCCACAGAATGTACACCCCAACAGTGAGCCCTAATGTTAACTAGGGACTTTGATAATAAT
GTGTAAATGCAGATTCATAAGTTGTAACAAACGTGCCACTCTGGTGGGAGAGTTGGTAATGGGAAAAGCTCTGTATGTATGGGTGCAGTGGGTATACGG
GAAATCTCTGTACTTACCTCTCAATTTTGCTGGAACCCTAAAACTGCTCTAAAAATATAAAGTCTTTAAAATACACATACACACATACAGACAATCGAAT
AGAGAATCATAATGGTACACTATCAGAAAATCATCAAACACAAAAAAAGGCAGTAAGGGAGGAAATGTTGTATTTAAAAAATGTAAGGCAAAAGAAA
AGGCACAAAATGTCAGAACTTCTTTTTTATAAGTAAGTACTTTAAATGTAAGTCGATTAAATTCTCCAAAAAGCAGAGGTTGGCAGAATGGATTTAAAA
GAAAACCATCAACCTATGCTGTATATAACAAACTCACTTTTGGTCTAAAGACACAAGTAGATTGAAAGTGAAAGAATGAAAAATGATATTTCATACAAA
TAGTAACCTAAAGAAAGCTGGAGGTAATTATACTAATAAAAGAAATAAACTTGAATTTAAAAATTGTTATAGGAGACAAAGGAGGACATTACATACTGA
GAAAATGGAAAATTACTCAAGACTTCGCAATTGGAAACGTATACACACCAAAATATAGAGCCCCAGAATATAAGAAAATATACTGACAGAATTGGAGTG
AGAAAGACAGTTCTACAATAATACTTAGGGACTTCAATAACGTACTTTCAGTAATGGGTAGAGTCAAAGAAGAAATGAAAACAGAAACTTAAAAATAT
CTTCAGACAAATGACAGTGGGAACACAACATACCAAAAGCAAAACATGCAGCAAAAGCAGTTCTAAGAGGGAAGTTTACAGCAATAAATGCCTTGCC
TACATTAAAAAAGAAGAGATATTTCAAATAGCCCAAAATTGTACCTCAAGGAATTAGAAAAAGAAGAACAAACTAAAGCCAAAGTTAACAGAAGGAA
ATAATAAAGATCAGAACAGAGACAAGTTAGAAAGAAAAACCATAGGGGAAAAAAGTCAGTAAAACTAAGACTTCAATTTTTGAAACAAAGAATTGAT
TTTTGAAAAATAAAATCAACAAACTCTTGGACTAAGAAAGAGGACAAAATCAGAAATGAAAATGGAGAATATATTACAACAGGTACTTCAGAAATAAA
AAGGATCATAAGGGACTTTTATGAACAATTATATCCCAACAAATTGGATAACCTAGAGGAAATGGACAAATTCCTAGAAAAAAACCAACCTGCTAAGAT
TAAATCAGGAATAGAAAGCCTGAACAGACCAATAGCAAAGAGATCACAGTGGTAAACTAAAGAGCTTCTGCATAGCAAAAGAAATTATCAACAGAGT

AAACAGACAACATATAGAATGGGAGAAAAAATATTTGCAAACTATTCATCTGGCAAAGGTCCTATGTCCAGAATCCATAAGGAACTTAAACCAATCAAC
AAGCAAAAAACGAATAATCCAATTGAATTGGCAAAGGACAGGAACATACATTTCTTAAAAGAAAATACAGAAGTAGCCAAACAAAAAAACATTCTCA
TGATCACTAATAATCAGAAATGCAAATAATGAGGCCAGGCACGGTGGCTCATGCCTGTAGTCCTAGCACTTTGGGAGGCTGAGGTGCGTGGATCATCTG
GGGTCAGGTATTCGAGACCATCCTGGCCAACATGGTGAAACCCCGTCTCTACTAAAAACACAAAAATTAGCCAGGTATGGTGGCGGTCACCTGTAATC
CCAGCTACTCAGGAGTCTGAGACAGGAGAATTGCTTGAACCCAGGAGGTGGAGGTTGCAGTGAGCCGAGATTGCGCCACTGCACTCCAGCCTGGGA
GACAGAGCAAGACTCTTTCTCAAAAAACAAACAAACAAACAAAAAAGCAAAAAACAGACAAAAAAAAACAAAACAAAACAACAACAACAAAAAA
ACACCAAACCATAATGGGATGCCATCTCACACCAGTCAGAATGGCAATTATTAAAAAGTCAAAAAAATAAAAGTTGCTGGGAAGGCTGCAGAGAAAA
GGGAACGCTTATACACTGTTGGCAGGAATACAAATTAGTTCAGCCACTGTGCTAAGCAGTTTGGAGTTTTCTCAAATAACTGAAAACAGAACTACCATC
TGACCCAGCAATCCCATTACTGGATATATATTCAAAGGAAAATAAATTGTTCTATCAAAAAGACACATGCATTTGTATGTTCATCACAGCACTATTCACAA
CAGCAAAGACATGGAATCCACTTAGATGCCCATCAGTGGTGGATTGGATAAAGAAAATGTGGTACATGTACACCATGGAATACTATGCAGCCATAAAAA
TGAATGAATTCGGCCAGGTGCGGTAGCTCTCACCTGTAATCCCAGCACTTTGGGAGGCCAAGGCGGGTGGATCACCTGAGGTCAGGAGTTCGAGACC
AGCCTGACCAACATGGAAAAACCCTGTCTCTACTAAAACTACAAAATTAGCTGGACGTGGTGGCGCATGCCTCTAATCCCAGCTACTCAGGAGGCTGA
GGCAGGAGAATCACTTGAACCCGGTAGGCGGAGGCTGCAGTGAGCCGAGATCATGCCATTGCCCTCCAGCCTGGGCAACAAGAGCGAAACTCCGTCT
AAAAAAAAAATAAAAAAAAAAAAATATATATATATACATGAATGAACTTACGTCCTTTGCAGCAACATGGATGCAGCTAGAGGCCACTATCTTAAGCAAA
TTAATGCAGGAACAGAAAACCAAATACTGCATGTTACCACTTGCAAGTGGGACCTAAACACTGAGTAAACATGGACATAAAGATGATCAGTAGACACT
GGGGACTACTTGGGGGAGGAATGGAGAGGTGGGCTGAAAACCTACCTATTGGGTACTATGCTCACTACCTGGGTGATAGGCTTATCCACACTCCAAAC
CTCATCATCATGCAGTATACTCCATATACCATGCAACATATAGACCTGTACATATACCCTCTAAATCAAAAATAAAAGTTAAAAAAACCCCAAAACCTCA
AACAAAGAAAAGCCCAGGACCAGATAGCTTCATGACTGAATACTACCCAATATTCAAAGAATACCAATACTTCTTAAACTCTTCCAAAAAAAATGCAGCT
ATAGGGAATACTTCCAAAAAAAAAAAAAATGCAGCTAAAGGGAATACTTCCTAACACATTTTATGAGGCCAGCATCACCTTGATACCCAGGTGAGCCAA
AGGCATCACAAGAAAAGGAAACTACAGGCCAACATAACCCAAGAATACCGATGCAAAAATCCTTAATAAAATATTGGCAGACTAAATTTAACAATACAT
CAAAAAGATTATATATCATGTCTATGTGGAATTAATTTCTGGCATGCAAATCTTATTTAATATACATGTAGCAATCAATGTGATACTAACAGACTGAAAGAT
GAAAACCACATGATCATCTCAATTGACACACAAAAAGTATTTGATGAAGTTGAACATCCTTTGTTGATAAATTCTAACAGTTCAGGTATAGAAGGAACA
CTCCTCAATATAACGAATGCCATTTATGAAAAGCCCACAGCTAACGTTATAATCAATGAGGGAAAACAGAAGCCCTTCTACTATGAACCAGTACAAGGC
AAGGATGCCCACTCTAGCTACTACTACTCAACATAGTACTGGAAGTACTAGTACAACCAAACAGACAAGAAAAAGAAATGAAAGACATTCAAATCAGG
AAGTAAGAAGTAATATTATCTTTATATGAAGATGACATGATCCTACATATAGAAAACCCCGAAGACTCTGACAAAGCAACCTGTTAGAACTAATAAATGA
GTTCAGTAAAGTTGTAGGATATAAAATCAATATAAAACAGTAGTTGCATTTCTATACACAAATAATGACCTGGCCAAAAAAGAAATCAAGAAAACAATC

CCATTTATGATAGCATCAAAAAAATTGCTTAGGAATAAATTTAGCCAAAAAGATGAAAGACCTGTACACTGAAAACTATGAATCATTGATGAAAGAAAT
TAAACAAGATACAAATAAATGGAAAGATATCCCATGCTCATGAATCTGAATAATATTGTTAAAATGTCAATAATATCCAAATCAATATACAGATTGAATAA
AATCCCTATCAAAATCCCATGGAAAATTTTTTTAAATTCTAAAGTTCATTTGGAATAGCAAGAACCTTAAGTAGCCAACGAAATTCTGAAAATTCTGTTC
TGAAAAACAAAGGGAGACTTCATACTTTCTGGTTTATTTTACAAAACTATTGATCAAACAGTATGGTCCTGGCATAAAAAACAAACATGTAGACCAGTG
GAACAGAATAGAGAGCCCAGAAAGAAATTCAAAGATATATGATCAACCAATTTTCCACAAGAACACGAAGAAAACAATGCAGAAAGATAGTCTCTCT
CATAAATGACGTTAGGAAACCCAGTTCTGTGGCTTGTCCAACCCATGGCCCACAGACCACATGTAAGCCCAGGATGGCTTTAATGAGGCCCAACACAA
ATTTGTAAACTTTGTTAAAACATTGAATTTTTTTGCATTTTTTTAGCCCATCAGCTGTTGTTATTGTTAGTGTATTTTATGTGTGGCCTAAGACAATTGTTC
TTACAGTGTGGCCCCGGGAAGCCAAAAGATTTGATATCGCTGCCTTACACCATACATAGAAATAAACTCAAAATGGATAAAAGACCTAAACATAAGACC
AAAACCCACAACCAGAAGAAAACATAGGGGAAAAGCTCGACATGGCTCTGGCAGTGATTTTTTGGAGATCACACCAAAAGCTCAGGCTACAAAAAC
AAATAATTGGTATACCATCAAACTAAAAAACTTCTGCACAGGAACGGAAACAGTCAACAAAAGGAAAAGGCAACCTATGGACTAGAAGAAAATATTT
GCCAACCATATATCTTATAAGAGGATAATATCCAAAATTTATAAGGAATGCATACAACTCAATAACAAGAAAATAGATAACCCAATTAAAAAATGGGCAG
TGGAACTGAGTAGACATTTTTGCAAAGAAGTTATAAAAATGGCAAACACATATATGAAAAGACATTTAACATCACTAATCGTCAGGGAAATGCAAATCA
AAATTACTATGCGATATCACCTCACACTCATGAGAATAGCTATTAAGTGTTGGCTAGGGTTAGGGAACTCTTGTACAGTGTTGGTGGGACTGTAGATTGG
TGCAGTCATAATGAAAAACAGAATGGTGGCTCCTAAAGAAATTGAAAATATACTATCATCTGACCCACTAATCCCTCTTCTAGGTATATTCCCAAAGAAA
ATGAAATCTCCACCTCATAAAGATATCTGCGCTCCTATATTCATTGTGGCATTATATGCATGAGTCACGATATGAAAACTTACGTGTCCATTGATGGATGAC
TAAACTGTGGTGTGTATGTCTACAATGGAATATTATTCCGCATTAAAAAAGGAGATCCTGCCACTTGCCACATGGATGAAACTGGAAGACATTGTGCTA
AGTTAAATAAGCCATATACAAATGAAAAGTTTTAAATAATCTCACCTATATGTAGAATCTTAAAAAGAAAAAAGTCAAATATACAGAGATAGAATAAACT
AGTGGTTACCATGGGCAAGGTGTGGGGGAAGAAAATGGAAAGATGTAGGTCAAAGGATACCAAGTAGCAGATATATAGGATGAACAAGTCTAGAGAT
CTCATGTAGAACATGAGAATAGTTAGTATATTGCATTTGGAATTTTTGCCAATTGAGTAGATCATAGCTTTTCTCACTCATACACACAAAAGAATAACTGT
GAGATGATGGACATATTAATTTGCATCACTATGTTAACGATTTAACTGTATGTTTTATACACTAAATATGCATATTTTTAAAATTAAAAAAAAAAAACACAA
CAAGATAATGTACAGCCAGACAGAAGCCCAATAAGAAAAGAGAGGACTTGAACAATACCATAAACCAACTAGACCTACAAACATATATGGACACTCCA
CCCAACTACACATTCTTCTCAAGTGCACATGGAACATTCCCTAGGAGGGATGATATGTTAGGCCACAAAAGAGTCTCAATAAATTTTAAAATATAGAAAT
CATACAAAATATCTTCTCTAACTACAATATAGTGAGGCCAGAAATCTGTAACAGAAGAAAAACCTAAAAATTAACAAATTCAGGGAAATAAAACTACAC
ACTCTTTAAAAATTATTGTGTCAAAGAAGACATCAGAAGGGAAATTATAAAATACTTTCAAAAAGTGAAAATGACGACACAACATACTCAGACTTATGG
GATGTAGTGAAAGAAGTGCTCAGAGGAAAAATTATAGCTGTGAATGCCCACACTGAAAAAGAAGAAGGATCCATAACCTAATATTATACCTTACGTAAC
CAGAGAAAGAAGAACGAAGTAAACCTAAAGTTAAAAGAGAGAATGAAATAATATGTATGAGTGGAGATAAAACTGAATTCGAGCTTAAATAAACAAC

AGAGATAATTTAACAAAACCAAAAGTTGCTTGTTTTTGAAAAGATCAAAGTTGAAAACCTTTAACTAGAATGACTAATAAAAAATTAGAGAAGTTGCA
CATATCTAAAATCATAAACAAAAGTAGGAACACAACAAAAAAAAAACAAAAATAAGAAAAAAAAAACAAAAGTGGGAACGATATAACTGATCTTACAGA
AATAAAAAGTGTGATAAAAGAATACTATGAATAACTGTATACCAACAAATTATATAACCTAGATAAGATGGACGAATTCACAAAATACTAATACAGACCA
AAACAGAAATTGAAATTATGAGCAGACCCATAACAAGTAGAAAGTTTGAATCAGTAACCAAAAACCTTCCAATGAAGAAAATGCTACACAGATGGCGT
CACTGTTGAATTATACCAAACACTTGAAGAATTCACACCAATCCCTCTCAAACTCTTCCAAAAAACAGAGGAGAAAAAACTTTGCAATACATTCTATGA
GGCCAGTATTACATGGATTCCAAAGCCAGACAAATATACCACAAGAAAAGACAAACTAGATACTAAAATCTTTTATTTAACAAAGTACTAGTAAGCCAA
ATTCAGCAGCATATTAAAAAGGATTATATATCATGACCACATGGAATTTATCTCAAGAATACAAGGGTGGTTCAGTACAAGAGTATCAATCAATGTAATAC
ACCACATTAATACAATGAAGGATAAAAAATCCATGCAATCATTTCAATTCACTCAGAACAAGCATTTGACAAAATTCTACAACCTTTCAGGATAAAAAC
ATGGAACTTTCAAAATGTGTTAAAGAGGATTTGTGAAAAATCCACAACTAATATACTTAATGAGAAAAAAAACTGAAAAATTTCTCCCATAACAAAGA
GTAAGATATGGCTGTATGCTTTACCCAGTGCTATTGAACAATGTACTGAAAGTTTTAACCATGACAATTAGGCAGATAAATAAACAAACAGGTAAATGAA
TAGCATCCAAATTGGAAAGGAAGCAGTAAAACTCTTTATTCAATAATGACATCTTCTATCTAGAAAATCAAAGAATCCACAAACTAGTAGAGCTAATAA
ACAAGTCCAGCAAAATTGCAGGGTACATGTTCAACATTTATTTTAAGAAAATCATTTGTGCCTCTATATACCAGGAATGAACAGCCCAAGAAGGAAGTC
AATTTTTAAAATTCCACTTACAATAGCATCAAAAAGAGTAAAATAGCTAGGAATAAATTAAAACAAGAAGGTGAAAGACTTCTGCACTAAAGACTACA
AAACACTGTTGACAAAAATTAAAGAAGACTTAAATAAATAGAACTATACATCCTGTGTTCACAGATTGGAAGATTAAATATTGTTAAGTTGGCGATATTA
CCCAGTGATCTACAGATTCAATGCAACCCTTATCAAAATCCCAAGGGCCTTTTTATTTGCAGAAATGTAAAAACTGATTTTAAAATGTAAATGGAATTTT
CCCAGGGCCCCAAACAATCTAAAATCATATTGAAAAAGAACAAAGTTGGAAGACTCAGGCCTGCCATTTTCAAAACTTTTACAAAGCTGCAGTAATCA
AAACAATGTGGTACTGACACAATGTGGTCTCTAGGACACAAGGATGGACATATAGACCAATGGAATATAATAGAGAATGTAGAAATAAACACTCACATA
TATGATCAATTCATTTTCAACAAATGTATCAAGAATTCAGTGGGAAGAGTAGTCTTTTAACAAATGGTGCTGGGGCAACTGGATCCTCATATATCAAAGA
ATGAAGTTGGACCCATAACTCATAGCATATAAAAATTATCTCAAAATGTATCAGAGACCAAAAAATAAGAGTTAAAGGCATAAATTTCTTAGTAGAAAAC
AGGAGTAAATCTTTATGACTTGAGATTGGAAATAAATTCTTAGAAAAGACACAAAAAACACAATATTGTAAATGTACTAAGTGACACTGAATTGTACAT
TTTGAAATGGTTTCTGTTACTTAAAAAATTTAAAGGGTGCACAGAAGACTAAAAAGCAGCACAGACAGAATTATCACCAGAAAAAGAAGACTCTATAT
TAAATGTCAACATGGAGAACACATGTCAGAAATAGGTAGTCCAGAGCAGCAGCTATAGTGGAGGCTCTTGCAAATGACTAAGATTTGAGGTGGCACCC
TCAGAGAATCACCTCTTGGGGGTAGATTCGATTTATGGAGTAGATGTATCTCAGAGACATGGTAGGAAAGCCAAAGAATGGTGAGCATAGCTGCCATCT
AGAAACCACCTACACATGCAAGCATAAGAGATTGGTTGAAAGGTTATAGCACAGTCACACTAAGGAATACTATGCAACTTTAGCAATGCAATGAGAAA
GATCTCTGTGAATGGATGTTGAGTGATTTCTAGGAGATGTTGTTAGGTGAGAAAAGCAAAGTTCAAAAGGGCATATACAGAATAGTACTTTTTTGGGAA
ATGAGAAAATAATCATGTCTTATAAAGAGCAAACACACACAAACTATAAACTAGAAAACACTGAAACTGGTTACCTATAATGGGTAGGGGGAATTAGGA

TGGAAGAGATATGGGTGAGAGGAAGATTTCTCTCAGTAGCTCCTTTTGTTTAGTATTGACTTTTGGAAGTATGTTAATGTTATATGTATTCAAAAAATAAA
ATTAACAAGATAGGGACCACTTCTGAGGTGGTGGAATGAGCACCTTGATATAACCACTCCCTGAAAAGCAATGATAAAACTGGACAAAGAAATATAAA
ATTGAAAAGTGCTAATTAAAGAAAAAATATTAAGCCTTCCTAAGAATAGCAGGGTTATGATTTAACGTGGGGCTGCCATCTCCCCACCCAAGCTCAGGG
GCACGGTAGGCTTGAGGACCAGCAACAAAGAAGGCTCATCTGATTTTGAATTCCATAGAAAACCCTATGCACTATGTTAAGCAACAAGGATTTCAGTG
GCAAATAATTGGAGGCAACCCACATCACAGCAACCTAATGTTTCAATACTGGTTGGGCAAGCGACAGATTAAGAAAAATTTTAAAAGGAATTCTGGAG
ATATGGAAGTTGTACATGCTTGCAGGAGAGATCAGAGAGGTTTCCTGCTATCTACTTATCCTTGGCTGACTGTGAGACCTTTTGCAAGCAGACAGTAAA
ATACAGGACAGAGTTATAAAGTGCCTGAACTTTGAATGTCCCAACCCACACATAGATCCCCTCAGCAGAGGGTGGAAGATTCACTGGCTCAAGATGTT
TAAACAAATGTCTGACTAATCATGGCTTGACCACTAGGTTATGCTGATCCAGGGGTGACACCAATGAAGCCAGGCTTACAAAACAAACATAAGAATTA
AAACAAACAAAACTGAGCAAAGACACCAGTGGCTGCACACTGCAGGAGAACACAGACTCTGCAGAATCCATGCAGAGAAGGCATACACAAGCGAG
CAAACAAATAAATGAGTAACAGAAATCATCCCTGGTTAGGGAGAAGAACTAGAATCCAGAGTTTCTACGATATATTATCTAAAGTAAGTTTTTAACAAA
AAAAATGAGATATGCAAAGAAACAGAAAATTGTGACCCTTGAACAAGAATAATGAGCAATAAATAGGAATTATCTCTGAAGGGCCCCAAATGTTAGGG
CGCGATTTCAAAGTAAGTATTATAACTATGTTCAAAGAACCAAAAGAAACCACATTAAAGAATGCAAGTGTGATGACAAAGACTCATCAAATAGATAAT
ATCAATATAGAGATAGAAATTAGAAACCAAATAGAAATTCTGTAGTTGTGAAATATAATAACTTAGGTGAAAAACTCACTAGTGCGGCTAAGCAAATTTG
ACCTGGCAGAAGAAAGAATCAATGAACTTCAACGCAGATCCATAAAGATGATTCAGACTGAAGAATAGAAAGAAAAATAATGAAGAAAAATGAAGAG
ATACCCATTGGATACCATAATATAGTGGTCCCCAACCTTTTTGGCATGAGGGACCAGTTTTGTGGAAGACAATTTTTCCACAGCCAGTTGGGGGAGGCT
TTAGTTAGATTCTCATACGGAGTGCACAACCTAGATCCCTCACATGCTCAGTTCACAATAGGATTTGCTCTCTGTGAGAATCTAATGCCATCACTGATCA
GACAGGAGGCAGAGCTCAGGCAGTAATGCTTGCTTGCCTGCTGCTCACCTCCTGCTGTGTAGCCCAGTTTCTAATAGGCCACAGACTGGTACCAGTCC
ATGGCCCTGGGGTTGGGAACCCCTGTCCTAACAGGATGTGTAACAATGTATGTGTAATTAAGTTGTAACATATATGTAATTAAGAGTAGAAAACATATGT
GGAATTAAGGAGTAACAACATATGTGTTAATAAGAGTACTGAGAAGGAGAGAATAGGGCAGACAAAAATATTTGAGTAGATTATTAACCTGAAGCAGA
TTGTTTTTGCTTAAGCTGCTTATTGTAACCTCCAGATTACACTTTAAAAATGGGGGAAATAAACTAACAAAGGAATTAGAATGGTACTCTAGAACATATC
TATTCAACCTGCAGAAGGCAGTAAAGGAGGAAGAGAGGAACAAAAGTGATATGAGACATATAGAAAATAAATAACAACATGGTAGATATAAATCCTTC
CATATCAATAATTATATTACATATAAGTGAATTAAAAACTCAATAAAGAGCAGAGATTGCCAGACTGCCTTAAAAGCAGGATCCAAATATATATGGTCTAT
AAGAGTCACATTCTAGATTCAAAGACATAGTTGAAAGTAAAATGATGGAAAAAGATGACATGCAAACAGTAATAAGAAAGCTAGAGTGACTGTACTAA
AATCAGACAAAATTGGCTGGGTGCAGTGGCTCACACCTGTAATCCCAGCATTTTGGGAGGCTGAGGCAGACAGATCACTCGAGGTCAGGAGTTCAAG
ACCAGCCTGGTCAACATGGTGAAACTCTGTCTCTACTAAAAATTAGCTGGGTATGGTGGCAAATGCCTGTAGTCCCAGCTACTTGGGATGCTGAGGCAA
GAGAACGGCTTGAATCCGGGAGGCAGAGGTTGCAGTGAGCCAAGGTCACGCCACTGCACTCCAGCCTGGGCAACAGAGTGAGACTCCATCCCAAAA

ATAAAATAAAAATAAATAAAATCAGACAAAATAGACTTTAAAAACAAACCATGTTATTAGAGATACTGAGAAGCATTTTACAATGACAAAAGGGAAAAT
TTAACAGGAAGAAATAGAAATCATAAACAGAAGTCATAAAGAAATAAAAATTTATAACAGCTAAAAACACAGCCCTGAAGTATATGAAATAAAACTGT
CAGAAATGAAGAGAGAAACAGACAACTGAACAATAATAGACAACTCAACAATAATAGTACTAACTCTTGTTGATTTTCTGTCTTGTTGTTCTATCAATTA
TTCAGAGTGAGTTATTGAACTATTACCCAACTACTATTCTTGGATGAAATGGTGGGAGGTGAGGCTATGTGGCACTCTGCTTAATACCCTCCCCTGTTCT
GGTGTCAGGGAGACAAATGTGGAGCTGACTTTTCGTCAGAAACAGTGGTACCCAGAAAGTAGTGGGGTAACAGATCCCAAGTGCTCAAAGTAAAAA
AAAAAAAAAAAAAAAAAAAAAAAAAGCCAACCAAGAATACTGTGTCCAGAAAAGTTTCTTTCAAAAAGAAGGCAAAATAAAAGCATTCCCAGATAAA
CAAAAACAGAATTCCTTGTTAGCAGACCAGTTTTGTAAGAACTACTAGAGGAAGTTCTTCAGGCAAAAAGGAAGTGCTCCCAGGAAGTTCACATATAT
CCAAAAGCAAAGAGCAAGGAGGTAAATAGGAGCAAAGCTGTTCTAGAGTAAGGAAATACCACCAGTTGGTAACTCAAATCCATAAAAACCAAAAAA
GTGGATCAAATATATGATATAAGGAGGTTAATAAAACAGAAACTAGAAATATATACTTGCTCTCCTTTCAGCTTCTTTAAAGATATATCATTATATAGTATG
ATCATTATAATAGCGCATAATAAAGATGTAATATATGTAACAATACCACAAAAAGTGGTAAAACTGAATAAACTCATACAGGAATAACTTCTGTATCTCAC
CAGGATTAATATAAATATGAAGTAAATTTTGAGACGATACATAAGGTAAGCCCTAGAGCAAACACTAATAACAAATAGAATATTGAAGAATCATAAAAGA
AATGAAAATTTATATTAGAAAGTAGTCACTAGATGGAAAAGAAAGCAGCAAAGGAGAAATAGAGGAACAAGAAATAGTACATAGAAAAGACCAAAAG
TAAAACTGTGGATATAAATCTAATTATATCAATAATAATATTAAATGTGAATGGATTAAACAATCTAATCAAAGGCAGGAATTATCAGACCAGATTTGAAA
AACAGGATCCATCTGTATTCCATCTATATGAGGCACACTTTAGATGATAAATGCCAACTGAAAGTAAAAGGATGGAAAAAGATATATCATGTAAGCAGCA
ACCATAAGTAACCTGAAGTGCTATAATAAAGTCAAAGGAGATTTTAAATTAAGAATGTTACTGAAGTTAAATATGAATATCTCATAATGATAAAATGTCAA
TTCATGAGAAGATACAATGATTATAAACATATATTCACCTAACAATAGAGAACTAAAACATAAGAAGCAAAAACTGACAGAACTGAACAGAGAAATAG
CCAATTCAACAATAGTAGTTGGAGACTTCAACACTCCACTTTCAATAATGTGTAGTACAAGAAAGAAGATCAATAAGGAAACAGAAGATATGAGCAAC
ACCATAAACCAACTAGACTCAAAAGGTAATTATAGAGCACTTCGCCCAACAACCAACATTGAATATACAGTATTTTCAAGTGCACATGAGACATTCTCC
AAGAAGGACCATATGCTAAGTCACGAAACAAACCTGCATAAATTTAAAAGGATTGAAATAATACAAGGTATCTTTTTCTGAATATAATGAAATAAAAGTA
GAACTCAGTAGCAAAGAAATTAGGGAAACATACACATGTGGAAATTAAACAACACTACATTCTTAAATAATCAATGGGTGAAAGAATTCACAAGGGAA
ATGGAACGTACTTTGAGATTAATGAAAATGAAGATACAACATAACAGAACTTATGGGATACATTTAAAGCAGTGTAAAGATGGAATTTTATAGCTATGAA
TGCCTATATCAAAAAATAAGGCTTCAAATCAGTAAGCCAACTCTCCACCTCAAGAAACTAGAAAAAGAAGAGCAAACTAAACCTAAAGCAAGTAGAA
AAAACAAAATAATAAATATTAGAGTAGAAATAAAATATAGAATATAAAAATAATAAAGTCAACAAATCCAAAATTTGGTTCTTTGAAATTAACAAACCTT
TGATAGATTGGTAAGAGAGAGGGAGAGGGAGAAGAAAAGAAGGGGAGGAAGAAAGAGGGGGAGGAGGAAAGACAGAGAGGGAAGTGCATGAGGC
AAAGGGGGAGAGAGAAAACTCAAATTACTAAAATCAGAAATGAATGACAGTTTTACAAATTAAAGCAAAAAAACGAGGCTAGCTGCAGTGGCTCACA
CCTGTAATCCCAGCACTTTGGGAGGCCAAGGCAGGAGAATTGCTTGTGGCCAGGAGTTCGAGAATTGCTTGTGGCCAGGAGTTCAAGAATAGCATGC

ACAACAAAGCAAGACCCTATCTCTACAAAATATTTTTTGAATAACAGCTGGTGTTGTGGTGCACACCTGTCATAGGCTAAGGGGCAAGGATCACTTGAG
ACTAGGAGATCGAGGTTACAGTGAGCTATGATTACACCACTCTATTCCAGCCTGGGCAACAGAGAGAGACTCTGTTTCTCAAAAAGAAAAGGAAAAA
GGAATGAATGAGGGAAGTATACTACCAACCTGACAGAAATAAAAAGGATTATGAAGGAACACTATGAACAATTTTGTGCCAATAAATTATAAAACTTAG
ATGAAATTGACAAATTCCTAGAAAGATGAAAACTATGAAAACTGACTA

>SNHG7

>ENST00000414282.5|ENSG00000233016.6|OTTHUMG00000020949.1|OTTHUMT00000055146.1|RP11-251M1.5-001|SNHG7|2357|

CTCTGCGTGCGCCGGAGGCTGCCGTGGCGGGTGGGCCGCCTGACTTCTCCTCCCGGCCAGTTCTCGAGCGCCTCACCGGGCCTCGCCCTGCAGCCTC
GCTCTCGCTGGCGCTGCGCGGCCTAGGGGACTGGGCTGCTGGCCTCCGGGTGCGGGGTGGGGGCAGGCTCCGACCTGGGGCGTCCTGGCCGCGCGA
GCCGCGGGATGGGGGCCCGGGCCGCGGAGGAGGCGCCGCTGGTGTGTCCCTTGGTGGAGAGGGCGCTGCCGGCCCTGCGCGGTTTCCAGCCAGGAA
GCTTCGGGAAGCCTGGACGTCTGCTCACTGGAGATGACACGTGCGTGGGGTGTTGGCATTCTTGTTATTTAACACGGGAAGGAGGTGACTTCGCCTGT
GATGGACTTCCAGTGTGAGCACTGGCCAGAGTGACCAGGCTGACCAGCACCAGCCCTGATCCAGATGCAGAGGCCAGGATGTGGGCCCAGCCCTGTG
CCAGGAGGCTGGCTGGAATAAAGAGTAACAAACCCCCTTGGAGGACTCTCCTGCCGGGATGTCCATGTCCGCCTTTGCTCCGAGCTGGGGTCTCATGT
CTGTGGTGCTGGAATCCAGAGCCCTGACGGGACAGCAGCAGCAGGAACTCGTTACGCTGCAGCAGGGCCCACACGGACTTGGCTTCCGCCAGGGAC
ACCCGGTTGTCCTTGTTGAAGTCAGCCATGAGCAGGACCTGGCCAACCAGCGCCGGCAGGGAAGGCAGGTCTCCCAGGTTCGCCTGGCGTTTGGGAC
CAAAAGAAAGGAAAAGCCAGCTGATTTGATGTCTGGGAGCACGACCTGGCCTTTACCTGGTCCTCCTGGCGGGGTGGTCTGCCGGCCAGACCAGCAC
ATCTGCCAGGTTAGGGGTCCCAGCCCACAGCTACACGGGTGGAGGACGCAGCCAGCAATGTGTCAGCTCACTCTGCTGGCCTGTGTGGCTGCTGCCA
GACAGACCTCCCGCCTCCTGGCAGCCTTGGGTGCACCTGGGGAGCTCGGGGGTGGGCACCTGGGCAGAGAAGCCTCAGAGCTTCCCTGAGGATGAG
AATCGGCACAGGGCAGAGGGCACGCACTGGCTGAAGCCCTGGGAAGCGGCCCCTTTCCACAAGCCCTGGTCTCCATTCGGGGCACCTGCCCTGCTGG
ACACACGGCCCTTGACTGCAGGAGCCTCCCCTCGACCTCAGCTGCCAGCCCACCGGCGCCCCCAACCCTTGCCCCCAGCCCTGGAGAGGCTCCTTCC
CTGGCAGGGCTCATGCCCAGGGGCTGGGGGATGGGAGGTCTGCCCAGGGTCCACTTGGGCCCATCTGTGCCTTTGCGCTGGGCATGTTGGTGCCAGG
GCCGCCTGCTGGGCATATGACCGTGGTGTGGACTCCTGCCCTGCCCCCTAGGAGAGCTGCCTGACCTTGAGGAAGCTGAGGGTCATCTCCCGGAATTC
CTTGATGGAGGTGCCCCGGGTGGGCTTGTCAAACAGTACCAGCTCCCGCCGGGGGGCCGCATCCGACCGGGCCTTGGAGTCGAGGGTCTCCTCAATG
CCACACTTGATGGTTACATCCTTGTCCCGCCAGAGCCCGCTGTACACCTGGACAGGGCCACAGAGGTCCTCCGTGCAGACATCCCCCTCCCACTGCAC
GGGCAGTATCAGCCCCAGGCCCACCCGCCCTGCCAGTGGCTATACCTGCTGGCCCGGGGCCACCGAGAGGCAGGTCCTCCACTCCACCATATGCAGCT
CACACAGGTCCTGGCAGACGGAGCCCGAGATGATCCCCTTGCGGTACTGGTCACACTGAGGAGACAGGTGCAGGCGTGCCGGGCGGGGCAGCCACA
CCCCTGCCCCACTGAGCCCCTGCCCACAGGCCCGAAGCTGGCAGGGGCCTCCACTTGCTGCAGTTGGAAAGCTGCCAGCCCCTCACACAGGCAGTGC

CGGGGCCCTGGGTCATGCCATTGGTGGCTGCAGGATGGGGCTGTCGGCTGCAGGGGCAGCCCCGCCAACCCTCCGGTCCGGTCCCGTCCCCACCATCC
TGGCTCCTGTAACAGGACGGCACAGCAAAGGCCACTGCCTGGACATGAGACACACACCACACCCAGTGTCGACCCCACGCCAGGGCCAGAGGCAGG
AACCTGGAGGCAGCTCTCCGCCCAGCCGACCCAGCTCTGGACCATCCAGGCATTGGCCGGTGAACTAGAATTCACACTAGTCCCTAATATCTACACCA
CCAGCTGCCACACGCGCGCTCTCTGCCTGACTCTTCATTCCTGCCTCGGGTGACGCCAGGAGGGAGGATGCACCCCCTGACTCATGGCGCCCTCCCTG
CCCGGAATAGTAAGTGAGACATTTCTG
>ENST00000416970.5|ENSG00000233016.6|OTTHUMG00000020949.1|OTTHUMT00000055148.1|RP11-251M1.5-003|SNHG7|789|
CTCTGCGTGCGCCGGAGGCTGCCGTGGCGGGTGGGCCGCCTGACTTCTCCTCCCGGCCAGTTCTCGAGCGCCTCACCGGGCCTCGCCCTGCAGCCTC
GCTCTCGCTGGCGCTGCGCGGCCTAGGGGACTGGGCTGCTGGCCTCCGGGTGCGGGGTGGGGGCAGGCTCCGACCTGGGGCGTCCTGGCCGCGCGA
GCCGCGGGATGGGGGCCCGGGCCGCGGAGGAGGCGCCGCTGGTGTGTCCCTTGGTGGAGAGGGCGCTGCCGGCCCTGCGCGGTTTCCAGCCAGGAA
GCTTCGGGAAGCCTGGACGTCTGCTCACTGGAGATGACACGTGCGTGGGGTGTTGGCATTCTTGTTATTTAACACGGGAAGGAGGTGACTTCGCCTGT
GATGGACTTCCAGTGTGAGCACTGGCCAGAGTGACCAGGCTGACCAGCACCAGCCCTGATCCAGATGCAGAGGCCAGGATGTGGGCCCAGCCCTGTG
CCAGGAGGCTGGCTGGAATAAAGGGATGGGCAGGCTGGCATGGGGGCAGCCGCTGCCCCTGCCTGGGTGTTGCTGTGTATTCCTGCCGGCCAGGGGC
CACTGCCAGGACCACGCCTCCCTTTTCATATCCCGATTCTTAAGTTCTGCTATTGTGGTATTCTGGTGGAGAAAAAAGAACCGCGTGGCTGTTTTTGAA
CTGCCTGGAACCTAAGACCCTGAATTCTTTTCCCCCCCAAGGGGAAAATCTATATGGAAAACATTTATTTTAAAATACAGGATGAAGTGAATTAAAAGA
TTTAAATGCA
>ENST00000436596.1|ENSG00000233016.6|OTTHUMG00000020949.1|OTTHUMT00000055149.1|RP11-251M1.5-004|SNHG7|509|
AGGGTTTGCTGGCGTCTCGGTTAATAGCAGTAACGGCAGCCGCTTGTGTTCTTGATTCTTTGCCAAGCATTCTTGTTATTTAACACGGGAAGGAGGTGA
CTTCGCCTGTGATGGACTTCCAGTGTGAGCACTGGCCAGAGTGACCAGGCTGACCAGCACCAGCCCTGATCCAGATGCAGAGGCCAGGATGTGGGCC
CAGCCCTGTGCCAGGAGGCTGGCTGGAATAAAGGGATGGGCAGGCTGGCATGGGGGCAGCCGCTGCCCCTGCCTGGGTGTTGCTGTGTATTCCTGCC
GGCCAGGGGCCACTGCCAGGACCACGCCTCCCTTTTCATATCCCGATTCTTAAGTTCTGCTATTGTGGTATTCTGGTGGAGAAAAAAGAACCGCGTGGC
TGTTTTTGAACTGCCTGGAACCTAAGACCCTGAATTCTTTTCCCCCCCAAGGGGAAAATCTATATGGAAAACATTTATTTTAAAATACAGGATGAAGTGA
ATTAAAAGATTTAAATG
>ENST00000447221.1|ENSG00000233016.6|OTTHUMG00000020949.1|OTTHUMT00000055147.1|RP11-251M1.5-002|SNHG7|2157|
CTCTGCGTGCGCCGGAGGCTGCCGTGGCGGGTGGGCCGCCTGACTTCTCCTCCCGGCCAGTTCTCGAGCGCCTCACCGGGCCTCGCCCTGCAGCCTC
GCTCTCGCTGGCGCTGCGCGGCCTAGGGGACTGGGCTGCTGGCCTCCGGGTGCGGGGTGGGGGCAGGCTCCGACCTGGGGCGTCCTGGCCGCGCGA
GCCGCGGGATGGGGGCCCGGGCCGCGGAGGAGGCGCCGCTGGTGTGTCCCTTGGTGGAGAGGGCGCTGCCGGCCCTGCGCGGTTTCCAGCCAGGAA

GCTTCGGGAAGCCTGGACGTCTGCTCACTGGAGATGACACGTGCGTGGGGTGTTGGGTAAGTTGGTGCATTGGAGCTGGAACAGGGTCAATCCTCCA
ATGTAACTAAGCCCTCCTGTGCAACATCTTAAGTGACTGGAGGGACCAGTCGGTCTTGTGTTCACATTGAGGCTAGAAGCTGTCTCCAGGGGAGTAAA
AACGGATTATTTAGTCTTCAACAGGACACTGCCCCTAGAGGCGTTGCAGCTGTGGCTGCCGTGTCACATCTGTGTCATTAGGTGGCAGAGATTAGAGA
GGCTATGTCTACGCTCAGCGTTCTGCCCCGTGAACGTTTGAATGTTTGATAGTCTCACACTCCTGTTTTGTGGACAGGCATCTGTTTTCCTTCGCTGGTA
TCTGGGAGAGAGGGTGGGCCCTGTGAGTGGGCTTTCTAACTCTCGCGCTGGGCTAGGCTGGCCTCTAGGGGTCTGAAGGGATAGTGGGAAGGTGGAG
GGTGCCTTCACCAGGAGCAGCCTTCATGAGTGAGTTTTAAGAACCTGTGAGGATCGGTCAGGTCAGGTGAGGTACGCGCTGGATCATGCCGTACACCG
TGTCAGGATCACGCAGGACAGGACTGGGGAAGGGTTTGCTGGCGTCTCGGTTAATAGCAGTAACGGCAGCCGCTTGTGTTCTTGATTCTTTGCCAAGC
ATTCTTGTTATTTAACACGGGAAGGAGGTGACTTCGCCTGTGATGGACTTCCAGTGTGAGCACTGGCCAGAGTGACCAGGCTGACCAGCACCAGCCCT
GATCCAGGTAAGAGGTGAGGCGGAGGCTTGCCCTCCTTCCCTGCCTGCCGGGTGCGTGTGAGCGGCTGCCCTGCGGATGCTGTCCTGGACCTGTTGGC
ACCACAGACAGTTGCTCTGCTGTGCCTGTGGCCTCGGGGCAAAGAGAAAGTGGCGATTTCTACACTCAGTGCTCGGGAACCAGTGGGCACTGAGAAT
GGTTTATGGCCTGACATTACTTGCAACCAAAGCCAGGCAGCATTTGGATTTTCGCTTTTGGAAACCACCTTACCTGGTTTGCTCCATGAGGGAGGGGCA
GCAACATCGCTGGGAACCCTGAGGTGTAGATATGTATAAATGAGCTGTGTGTGTATCTGAAGCTCGGGCACTTGTGGGGTTACCAGGAGTGTGCGTCTC
ACTCCCAGGCCTCGTTCTGGAAACGATTCTGTAGGTCTGTCTGGGAGGCCTTAGGCAAGTTTGATTAAGAACAGTCGAGCTGGATAAACAGGCTGTTC
CTTGTCCAGCACTAGTCATCCCAGGGGGGCGGGGGCGGGGCTGCAACTCCTGTGCACAAAGGAAGGGCCCTGGGTAGGGGTCAGGAGCTGCCTTGC
CCAGAGATGTGTCTGGGTCACTTTGGAAACAGGAATCCTCTCCCCAAAATGGGTAGCAATGGGGGTACAGAGGCCAAAGACGCAGCTGCATGTCCTG
CAGTGCCTGGGACGCCCCGTAGTGAAGAGTGATGTGGCCCAAATGTCAGCAGTGCCAGTGTCAGACTCCTGCTAAGAGACAGTATCAAGAAAGATAA
CTTCAAAATACACTTTCTGGAGGCCTGGCGCGGTGGCTCAGGCCTGTAATCCCAGCACTCTGGGAGGCCAATGCATGTGGATCACCTGAGGTCAGGAG
TTCGAGACCAGCCTGGCCAACTGCCTGAAACCCCATCTCTACTAAAAATACAAAAATTAGCCAGGTGTGGTGGCGCAAGCCTGTAATCCCAGCTATTTG
GGAGGCTGAGGCAGGAGAATCGCTTGAACCCGGGAGGCAGAGGTTGCAGTGAGCCGAGATCGCACCACTGCACTCCAGCCTGGACAACAGAGTGGG
ACTCTGTCTC
>DLEU2
>ENST00000621282.4|ENSG00000231607.10|OTTHUMG00000016927.5|OTTHUMT00000474800.1|RP11-34F20.2-010|DLEU2|3068|
GGAGCTTTGCTGAAACTGCACAAAAAATCGAGCCGGGGGGTTCCCTGGTCCCCGGCGATGGGGCGGGGAGCGCTGCGCCGGGGGAGGGGGCGGGCG
CGGCGGCGCGGGCCCCGCCGAGGGGGGACACCTGGCTGAGGCACAGCTGCCGCCGTGCCTTTCCGCGCGAGCCCAGAGCTCCGATCCCTGCGCGGG
CTCGAGAGCTCGCCCCGAACGGGGGTCTTCCTCCTCCGCCGTTGACAGGTTTTGACCTGTAGCAGAGAACCAATTCTGGAGAACAGCCTCACTTCTTT
GATTGAATACTTACATAATGCATTGGAACATGACATGAGATTAAGCTGGGAGCAGAGGCTGGGAGATCGATGCTGCTTGTCAGCTGTTGTGCAGAATAA

CATCAATATGCAATAATGGTGGCCAAAGGAGCCTGCACCCATCCGTTTTTACAAACCCAGTGTATAAAGCCAAATGTCCCATTTAAAGTTTGACATTTGC
AATAGCATTCTATTTAAAAGGTTTAATAATGATAGAATGAAGACCACAATAAAAGAGACCTCTACTTAGCTCAGCAATTCTTACCTTTCTTACCTATTTGA
TGAAGATGTCTTTTGAAAGGTGTACTGCAAGGAACAAAATGTTTGTAAATTCTCCTTTTACCAAGGTTTCTTTTTTAATTGATGCCAAAGAGTTCCAATA
TTGAACATCTTAAGTCTGTTACTTGGATTACGGATTGAGTTTGGAGCTTACTCAGAGGACTACAGGAGAGTATCCAGGAAGTGGATAATTACTGTACCTT
CCTCATGGAAAAAGTTTTATTTAAAGTGTTATTTCTCATTGAATACTATCAAAAAGGAAAAAAAAATGACCTAAACTTTTGAGATAGATTTGGCTCTAGT
AAGTATTTAGGCCTACTCTAGGTGCGGTACTTCACTATAGTTTATAAAAGATGGTCCCTGTCAGCAAAGAACTGTAACCTGTATGAGAATATTATACTAAC
ATACAAGAAACAAGTAGAGAATAATGGAATGTAAACTCTGGCTTGCTAACTTAAGTGTGGTAGAAGCTTGAAGGAAATGTGGACTTAAACTTATCAGA
GAAGACTTATAAGAGGATATGAAAGGTGTAAATTTTGATACCACCATTGCCGCCTTCCCCTCAAAAAAGGTGAGAACTGACTAAACTACTGGTACTTAG
ACCATGTGGAGAAGGAACTGAAAATGGAAACAGAAACACGTCTGTACAGAGCAGAGTGTCAGATAATAGCTCATTTTAGTAGAGGGCAATAAATGCC
ACATGAGATGATCACCTCAGCAGAAAACTGGGATGTGGAGACGCTGGTAGCGGGGCCAGGAAGGCTCCGGACGGAGCAGGTTTTCTGGACCACAGA
CACACTGCCCCGGCGCCCTCCGCAGGCCGCTCCTCCCCAGGCCCATGACAGTGCCCCGCTGGTTTCTGCCGCGCTGCCGGACTGGAGCTCAGACGGC
CTTCGCCAGCCCCCCCAACCCACGCAGATCCCTGCTGACCTGGGCAACTCCCCACCCTCGCTGAAGGTTCGAGGACCACCCCGCTTTCCCGAGAGGA
GCCGGGCGGCGGGTACTTATCTCCGACCTCCGGCTAGTGGGAAAGGCCGCGCGACCGCCCGTCCTCCAACTACAGGGACCGCACGAACAGCGACTGG
GAAACTGCCACTAGAAAAAGATGACTGTCCAACTAAAACCTGCATACACAGCTACATCCTTCCCTGGAAGAGCACAGTGGAACTAGATCCTAGTACAG
ATAGCACAGGGATTGTCAACATTCTTGTCACACTGAAATTTCCTTCTACTGATCTCCAAACTTCTAAAAATTTGCTGTTGAGACCAGCTGAAATGTTTGA
AAGAAGCAACTAAAGAAAGAAACTTTGAAAGAAGCTTCCAAAAGAAGCAACCAAGTCTACAAAGGCACAGTTTGACTCAAATGGATCCAACTGATT
AGCAAATGGAAAATAACATGGAGGAATATATTTTGCATGACAAGCATAGCAGCTCCAAGTCATCCCTAATGAAGCCAAAAATTTAAGTGCAACCCAGA
GAATGGATGGTTCAGAATCTTTTTTTTTTTTGAGATGGAGTCTCGCTCTGTCACCTAGGCTGGAGTGCGGTGGCATGATCTCGGCTCACTGCAAGCTCC
GCCTCCTGGGTTCACGCCATTCTCCTGCCTCAGCCTTCCGGGTAGCTGGGACTACAGGTGCCTGCCACCACGCCCGGCTAATTTTTTGTATTTTTAGTAG
AGACGGGGTTTCACCGTGTTAGCTAGGATGGTCTCAATCTCCTGACCTCGTGATCTGCCCGCCTCAGCCTCCCAAAGTGCTGGAATTACAGGCGTGAG
CCACCGCGCCCGGCCCAGAATCTTTTTTAAATTTTTTATTATTATTATTTTTTCTGAGATGGAGTCCGGCTCTGTGGCCAGGCTGGAGTGCAGTGGCACA
ATCTCAGCTCACTGCAACCTCTGACTCCCAGGTACAAGTGATTCTCCTGCCTCAGCCTCCTGAGTAGCTGGGATAACAGGCACGTGCCACCACGCCCA
GCTAATTTTTGTATTTTTAGTAGAGACGGGGTTTCACCATGTTGGCCAGGATGGTCTCGATCTGCCTACCTTGGCCTCCCAATGTACTGGGATTACAGGC
GTGAGCCACCGTACCCGGCCCAGAATCTTTGCCTTTTGTTTGCATAGTTTAATCTTCTAAACCAGTTGCTTTCCCAGAAAAGGTGACTTAAAGTCATAGC
CTTTGTGTTCATCACCATACTCTCCATAGTTTTACAAATTATTCAGTTTATTTTTAAAAGGGGTAATTTTTAAAATTTTTATTTTTAATTCCGAAGCATTCCT
TTTAATACATTCTCTTGAGGGAAAAAAAAAATCACACATAGTGATTGTGTGTGGTAGTGATTATGTGTGGCCTAAGTGGCAATTGATCAATACCCCAATC

TCAAGCCTGTACATTGTTACTGGTTGACTGGTTGGTTGCCTGGTGGGCTTTTTGTCCTTCCAGATGTTTTTCTACCTTCTGCCTTTTCCAATAAAGCTTCC
ATTAT
>ENST00000425586.5|ENSG00000231607.10|OTTHUMG00000016927.5|OTTHUMT00000044957.1|RP11-34F20.2-004|DLEU2|1267|
GGGGAGCAGAAGGCCGCGGGCGGGCGGCTGGCGCGCTGTGTACTTAGGTCGTGTGCTGGGGCTTTTCTCTCCCAGGAGCCGGCGGGGGGAGGGGAG
GGGGAGGGGCCACCGCTCCGCCTTCTCCTTTTCGCAATGTTGACGCAATCTATAAATAGTGGAACAAAAGGACCAACTTCCTCGGAGCTTTGCTGAAA
CTGCACAAAAAATCGAGCCGGGGGGTTCCCTGGTCCCCGGCGATGGGGCGGGGAGCGCTGCGCCGGGGGAGGGGGCGGGCGCGGCGGCGCGGGCC
CCGCCGAGGGGGGACACCTGGCTGAGGCACAGCTGCCGCCGTGCCTTTCCGCGCGAGCCCAGAGCTCCGATCCCTGCGCGGGCTCGAGAGCTCGCCC
CGAACGGGGGTCTTCCTCCTCCGCCGTTGACAGGTTTTGACCTGTAGCAGAGAACCAATTCTGGAGAACAGCCTCACTTCTTTGATTGAATACTTACAT
AATGCATTGGAACATGACATGAGATTAAGGTTTAATAATGATAGAATGAAGACCACAATAAAAGAGACCTCTACTTAGCTCAGCAATTCTTACCTTTCTT
ACCTATTTGATGAAGATGTCTTTTGAAAGGTGTACTGCAAGGAACAAAATGTTTGTAAATTCTCCTTTTACCAAGGTGGATAATTACTGTACCTTCCTCA
TGGAAAAAGTTTTATTTAAAGTGTTATTTCTCATTGAATACTATCAAAAAGGAAAAAAAAATGACCTAAACTTTTGAGATAGATTTGGCTCTAGGCCTAC
TCTAGGTGCGGTACTTCACTATAGTTTATAAAAGATGGTCCCTGTCAGCAAAGAACTGTAACCTGTATGAGAATATTATACTAACATACAAGAAACAAGT
AGAGAATAATGGAATGTAAACTCTGGCTTGCTAACTTAAGTGTGGTAGAAGCTTGAAGGAAATGTGGACTTAAACTTATCAGAGAAGACTTATAAGAG
GATATGAAAGGTGTAAATTTTGATACCACCATTGCCGCCTTCCCCTCAAAAAAGGTGAGAACTGACTAAACTACTGGTACTTAGACCATGTGGAGAAG
GAACTGAAAATGGAAACAGAAACACGTCTGTACAGAGCAGAGTGTCAGATAATAGCTCATTTAGTAGAGGGCAATAAATGCCACATGAGATGATCAC
CTCAGCAGAAAACTGTAAGTAACTGGGGACTTACAGGAGAATTGAACAGTTGTTTCTGCTGACTCAGTAAAAAGTCTTCATTTTATAA
>ENST00000235290.7|ENSG00000231607.10|OTTHUMG00000016927.5|-|DLEU2-201|DLEU2|1739|
GATGCCTGATCTCATCAATCTAGCGGGAGAGACAGGATAACCTGTCCGAGAGTATAGCGCCACTATGACTCCGCCGGAAAAATTACTTTAAAAATCGCC
AAAAATTACTTGGAGCAAAGGGCAGTCGGCGGAGCTTCGCCAAGGCTGGCGCAGTCGGTTTTGACCTGTAGCAGAGAACCAATTCTGGAGAACAGCC
TCACTTCTTTGATTGAATACTTACATAATGCATTGGAACATGACATGAGATTAAGGTTTAATAATGATAGAATGAAGACCACAATAAAAGAGACCTCTAC
TCTCAGCAATTCTTACCTTGTAAGTATTAACTGTCTTACCTATTTGATGAAGATGTCTTTTGAAAGGTGTACTGCAAGGAACAAAATGTTTGTAAATTCTC
CTTTTACCAAGGTGGATAATTACTGTACCTCCTCATGGAAAAAGTTTTATTTAAAGTGTTATTTCTCATTGAATACTATCAAAAAGGAAAAAAAAATGAC
CTAAACTTTTGAGATAGATTTGGCTCTAGTAAGTATTTAGTTATATCACTTGCATATCTGGGAGAAGAAATAAGAGACTATCATCAGTACATTCCCATCTA
CTAAAAAAATTTATTTTACACATGTCAAGGGATTACTTATAACTTCCATTTTATTACTAATAGCTTGAACCCTTTTAATGAAGACCTAACTCCTCCACCAG
AAATTTAAGTTTATGTTCTTACTTTGTTTACTTATAAAATACATCTCAGGTATTTCGGATGTCTTTTTTTTTTCTAAGCCTATATGAAATGAAAAATATATTG
GCAAAATAAATGTTTAAACCTTTTACGTTAAAATTACTTTGAAAGATGAAAAGTTAGTGCTGTTTTTGTCTACGTTATACTGAAATTAAATGTTTATAATTT

ATATTTTGGGTTTATGTATAAATCATGGAATTTATGCAAAAATATGAGTAGTACAGATTCTCCTCTAATTCTGTAGGACTTTGAATAATGTGATATTTTTCTT
ATAATTGGACCCTTGTGTTTTGAAGAAATGCCAACTGCTTGAAGAATCTCCTTGTTATTTGTATTATTTGCTATAGGGTTAGATGTTGAGAAATTCTGCTG
ACAAAAAATTTTAAGCCAGTTTTACACTAAATGTTCCTCAGTCTGATTAATTTGTTATTGGATGTATTCTGTATCTTTCTTTTGTAATTTGTAACTTTTATCC
ACTTAGCACGAATGATTCTATTAAAGAAAATCATTAGGAAGTGGTAGAAACTTTAAATCGCCCCAGAGTTTGCCTGTTTCCATATTTTATTATCTTATAAT
CTTCGGGAGTGCTTACACTTATGGAGCTAACATTTTCAGAGATACAGCTTCTTATAGTAACACTAAAACTTTCTTCCTCTTTGGACTGAATACCTATAATT
ATAACTATATGGTAGTTTAAGTTTCCTTGTGATTAGTCAAAAATACCATTTTAGTATGAAGCAATGAAGTCTATTATTTGTTGTCCCATAATTGAGAAAGCT
TAAATACACCTTTTATTAAGAGTTTGTAAATTCTAGCTTAGTCTACACAGATTTTTATATCAATTTGTTTATATTTTTATTAATGTCATTTCTGGAAGTGTGA
AAATGTTAATGTTCAACAAGCAACATTAAAAATAGATTTGAAACATTTATATATAGAGAGGTACACATTTATTTACTGTTTAGGTACTGAAGATTATCACT
TAATAAAAAATATATATCCC
>ENST00000458725.5|ENSG00000231607.10|OTTHUMG00000016927.5|OTTHUMT00000044961.2|RP11-34F20.2-008|DLEU2|1657|
GATGCCTGATCTCATCAATCTAGCGGGAGAGACAGGATAACCTGTCCGAGAGTATAGCGCCACTATGACTCCGCCGGAAAAATTACTTTAAAAATCGCC
AAAAATTACTTGGAGCAAAGGGCAGTCGGCGGAGCTTCGCCAAGGCTGGCGCAGTCGGTTTTGACCTGTAGCAGAGAACCAATTCTGGAGAACAGCC
TCACTTCTTTGATTGAATACTTACATAATGCATTGGAACATGACATGAGATTAAGGTTTAATAATGATAGAATGAAGACCACAATAAAAGAGACCTCTAC
TTAGCTCAGCAATTCTTACCTTTCTTACCTATTTGATGAAGATGTCTTTTGAAAGGTGTACTGCAAGGAACAAAATGTTTGTAAATTCTCCTTTTACCAA
GGTGGATAATTACTGTACCTTCCTCATGGAAAAAGTTTTATTTAAAGTGTTATTTCTCATTGAATACTATCAAAAAGGAAAAAAAAAATGACCTAAACTTT
TGAGATAGATTTGGCTCTAGTAAGTATTTAGTTATATCACTTGCATATCTGGGAGAAGAAATAAGAGACTATCATCAGTACATTCCCATCTACTAAAAAAA
TTTATTTTACACATGTCAAGGGATTACTTATAACTTCCATTTTATTACTAATAGCTTGAACCCTTTTAATGAAGACCTAACTCCTCCACCAGAAATTTAAGT
TTATGTTCTTACTTTGTTTACTTATAAAATACATCTCAGGTATTTCGGATGTCTTTTTTTTTTCTAAGCCTATATGAAATGAAAAATATATTGGCAAAATAAA
TGTTTAAACCTTTTACGTTAAAATTACTTTGAAAGATGAAAAGTTAGTGCTGTTTTTGTCTACGTTATACTGAAATTAAATGTTTATAATTTATATTTTGGG
TTTATGTATAAATCATGGAATTTATGCAAAAATATGAGTAGTACAGATTCTCCTCTAATTCTGTAGGACTTTGAATAATGTGATATTTTTCTTATAATTGGAC
CCTTGTGTTTTGAAGAAATGCCAACTGCTTGAAGAATCTCCTTGTTATTTGTATTATTTGCTATAGGGTTAGATGTTGAGAAATTCTGCTGACAAAAAATT
TTAAGCCAGTTTTACACTAAATGTTCCTCAGTCTGATTAATTTGTTATTGGATGTATTCTGTATCTTTCTTTTGTAATTTGTAACTTTTATCCACTTAGCACG
AATGATTCTATTAAAGAAAATCATTAGGAAGTGGTAGAAACTTTAAATCGCCCCAGAGTTTGCCTGTTTCCATATTTTATTATCTTATAATCTTCGGGAGT
GCTTACACTTATGGAGCTAACATTTTCAGAGATACAGCTTCTTATAGTAACACTAAAACTTTCTTCCTCTTTGGACTGAATACCTATAATTATAACTATATG
GTAGTTTAAGTTTCCTTGTGATTAGTCAAAAATACCATTTTAGTATGAAGCAATGAAGTCTATTATTTGTTGTCCCATAATTGAGAAAGCTTAAATACACC
TTTTATTAAGAGTTTGTAAATTCTAGCTTAGTCTACACAGATTTTTATATCAATTTGTTTATATTTTTATTAATGTCATTTCTGGAAGTGTGAAAATGTTAAT

GTTCAACAAGCAACATTAAAAATAGATTTGAAACATTTA
>ENST00000433070.6|ENSG00000231607.10|OTTHUMG00000016927.5|OTTHUMT00000044954.2|RP11-34F20.2-001|DLEU2|698|
CGCCATTTTCGAGTGATGCCTGATCTCATCAATCTAGCGGGAGAGACAGGATAACCTGTCCGAGAGTATAGCGCCACTATGACTCCGCCGGAAAAATTA
CTTTAAAAATCGCCAAAAATTACTTGGAGCAAAGGGCAGTCGGCGGAGCTTCGCCAAGGCTGGCGCAGTCGGTTTAATAATGATAGAATGAAGACCAC
AATAAAAGAGACCTCTACTTAGCTCAGCAATTCTTACCTTTCTTACCTATTTGATGAAGATGTCTTTTGAAAGGTGTACTGCAAGGAACAAAATGTTTGT
AAATTCTCCTTTTACCAAGGTGGATAATTACTGTACCTTCCTCATGGAAAAAGTTTTATTTAAAGTGTTATTTCTCATTGAATACTATCAAAAAGGAAAAA
AAAATGACCTAAACTTTTGAGATAGATTTGGCTCTAGTAAGTATTTAGTTATATCACTTGCATATCTGGGAGAAGAAATAAGAGACTATCATCAGTACATT
CCCATCTACTAAAAAAAATTTATTTTACACATGTCAAGGGATTACTTATAACTTCCATTTTATTACTAATAGCTTGAACCCTTTTAATGAAGACCTAACTCCT
CCACCAGAAATTTAAGTTTATGTTCTTACTTTGTTTACTTATAAAATACATCTCAGGTATTTCGGATGTCTTTTTTTTTTCTAAGCCTATATGAAAT
>ENST00000443587.5|ENSG00000231607.10|OTTHUMG00000016927.5|OTTHUMT00000044958.1|RP11-34F20.2-005|DLEU2|705|
TGATCTCATCAATCTAGCGGGAGAGACAGGATAACCTGTCCGAGAGTATAGCGCCACTATGACTCCGCCGGAAAAATTACTTTAAAAATCGCCAAAAAT
TACTTGGAGCAAAGGGCAGTCGGCGGAGCTTCGCCAAGGCTGGCGCAGTCGGTTTTGACCTGTAGCAGAGAACCAATTCTGGAGAACAGCCTCACTT
CTTTGATTGAATACTTACATAATGCATTGGAACATGACATGAGATTAAGTGTATAAAGCCAAATGTCCCATTTAAAGTTTGACATTTGCAATAGCATTCTA
TTTAAAAGGTTTAATAATGATAGAATGAAGACCACAATAAAAGAGACCTCTACTTAGCTCAGCAATTCTTACCTTTCTTACCTATTTGATGAAGATGTCTT
TTGAAAGGTGTACTGCAAGGAACAAAATGTTTGTAAATTCTCCTTTTACCAAGGTGGATAATTACTGTACCTTCCTCATGGAAAAAGTTTTATTTAAAGT
GTTATTTCTCATTGAATACTATCAAAAAGGAAAAAAAAAATGACCTAAACTTTTGAGATAGATTTGGCTCTAGTAAGTATTTAGTTATATCACTTGCATATC
TGGGAGAAGAAATAAGAGACTATCATCAGTACATTCCCATCTACTAAAAAAAATTTATTTTACACATGTCAAGGGATTACTTATAACTTCCATTTTATTACT
AATAG
>ENST00000421758.5|ENSG00000231607.10|OTTHUMG00000016927.5|OTTHUMT00000044955.1|RP11-34F20.2-002|DLEU2|692|
CTGATCTCATCAATCTAGCGGGAGAGACAGGATAACCTGTCCGAGAGTATAGCGCCACTATGACTCCGCCGGAAAAATTACTTTAAAAATCGCCAAAAA
TTACTTGGAGCAAAGGGCAGTCGGCGGAGCTTCGCCAAGGCTGGCGCAGTCGGTTTAATAATGATAGAATGAAGACCACAATAAAAGAGACCTCTACT
TAGCTCAGCAATTCTTACCTTTCTTACCTATTTGATGAAGATGTCTTTTGAAAGGTGTACTGCAAGGAACAAAATGTTTGTAAATTCTCCTTTTACCAAG
GTAAAGATCAAATTTTATAAATTTACTTGTTTGTTTATACAAGGAAAAATAACTTCATATATTGAATATATTCAAAAGTTTAAGCATTTAGTTGTATTGCCC
TGTTAAGTTGGCATAGCAAATAAATGCTTTTCTTTTCCTCATTTTATTCTTTGTGTTTCCTAACCTATAGCACTGTGCTGGGCACAGAATGGACTTCAGTT
AAGTTTTTGATGTAGAAATGTTTTATTATTCTACTTAAAATCTCCTTAAAAATAATTATGCATATTACATCAATGTTATAATGTTTAAACATAGATTTTTTTAC
ATGCATTCTTTTTTTCCTGAAAGAAAATATTTTTTATATTCTTTAGGCGCGAATGTGTGTTTAAAAAAAAATAAAACCTTGGAGTAAAG

>ENST00000449579.1|ENSG00000231607.10|OTTHUMG00000016927.5|OTTHUMT00000044956.1|RP11-34F20.2-003|DLEU2|791|
GATGCCTGATCTCATCAATCTAGCGGGAGAGACAGGATAACCTGTCCGAGAGTATAGCGCCACTATGACTCCGCCGGAAAAATTACTTTAAAAATCGCC
AAAAATTACTTGGAGCAAAGGGCAGTCGGCGGAGCTTCGCCAAGGCTGGCGCAGTCGGTTTTGACCTGTAGCAGAGAACCAATTCTGGAGAACAGCC
TCACTTCTTTGATTGAATACTTACATAATGCATTGGAACATGACATGAGATTAAGGTTTAATAATGATAGAATGAAGACCACAATAAAAGAGACCTCTAC
TTAGCTCAGCAATTCTTACCTTTCTTACCTATTTGATGAAGATGTCTTTTGAAAGGTGTACTGCAAGGAACAAAATGTTTGTAAATTCTCCTTTTACCAA
GGTAAAGATCAAATTTTATAAATTTACTTGTTTGTTTATACAAGGAAAAATAACTTCATATATTGAATATATTCAAAAGTTTAAGCATTTAGTTGTATTGCC
CTGTTAAGTTGGCATAGCAAATAAATGCTTTTCTTTTCCTCATTTTATTCTTTGTGTTTCCTAACCTATAGCACTGTGCTGGGCACAGAATGGACTTCAGT
TAAGTTTTTGATGTAGAAATGTTTTATTATTCTACTTAAAATCTCCTTAAAAATAATTATGCATATTACATCAATGTTATAATGTTTAAACATAGATTTTTTTA
CATGCATTCTTTTTTTCCTGAAAGAAAATATTTTTTATATTCTTTAGGCGCGAATGTGTGTTTAAAAAAAAATAAAACCTTGGAGTAAA
>SNHG1
>ENST00000539921.5|ENSG00000255717.6|OTTHUMG00000167743.1|OTTHUMT00000396015.1|RP11-727F15.10-006|SNHG1|1071|
GAAGGGTGTTATTTTTTCAGATGTTCAGCCCACAAGAGCTTACTGGTGAAGGAATGGGACAAGACCCATCTTTATGCAAAGCCAGCGTTACAGTAATGT
TCCAGCATCTCATAATCTATCCTGGGGAATTCAGCTGCCTCCCAGGGTGAATACAGGTATTCCTGATGACAGTCTGCCTCTATCTTACAGAGCAGCTTGT
TGCTATATACCATTGAAAAGCCTTCAGAGCTGAGAGGTACTACTAACCAATAACCTGCTTGGCTCAAAGGGCCAGCACCTTCTCTCTAAAGCCCAAGA
GGAGTTTGAGGAAAACTAGGTGTCTGTGTTCACTCCAGGCTGAAGTTACAGGTCTGAGCAAATAAGGTGTATAAAAAATGGAATCTGTCTTGGAGGAC
ATCAGAAGGTGAATTTTCCAAGTTCTTGGACAACCTAGCTGTTGAAAAGCTTTCTGGGTTTGGGGGGTATTTCAGATGTACCTTAAAGTGTTAGCAGAC
ACAGATTAAGACACTGGGAGCCAATGAAACAGCAGTTGAGGGTTTGCTGTGTATCACATTTCTGTATTTTATCACCCCCTTCCTGCAACATTATTTATCT
GGAATCTACCTGCCCTTTTGTTTTTTAGATACAAGGGCTTGGTTTTGTTACCCAGGCTGGTTTCAAGGCCATAGCTTTAAGAGATCCTCTCACCACAGAT
TTCCAAAGTGCTGGGATTGCAGGTGTGATTCATGGCACCCAGACTTTGCTGCCTTTCTTACATGATCCAGGCCCAGAACCCAAACTCAGGCACTGTATA
GATGACCACTTTCGTAAACTACTGACCTAGCTTGTTGCCAATTGTTGATTGAACTTCCCATAACTCCACTTCGTGTCTGTTCCTCTGTATACAGCCACCTT
CTGTTCCCGTCATGAGCCTTTAGGTCTCCATTTGCATATTGCAAATACTATGTTCCATGTAGGTAGCTCATTCAGGGCCTTGCTCTTCACTTCAAAAAAGG
TTCCCTTGAGGACTGGCTGTCAATTTGTGTTGCTGTGTTGGTTGTTGATGAAAATAATAAAATGATTGATTACATA
>ENST00000540725.5|ENSG00000255717.6|OTTHUMG00000167743.1|OTTHUMT00000396016.1|RP11-727F15.10-001|SNHG1|1374|
GCTGCCTGGCCAGGGCGACTGGCGGATAAGGTCTTGTGCGTGGCCTCGAGGCTTAAAAGTAGCAGTGGGGCTTTGTGAAGGACAAAATGGCGATGGC
GGGCCGTGTAGGTCCCCCTTCCTATGATGAGGACCTTTTCACAGACCTGTACTGAGCTCCGTGAGGATAAATAACTCTGAGGAGATGGGCCCTGCAAG
CCTCTTGCTTAGCCGTCTGTTCAGAAAATAGCGTTTTCGAAATGCCCTGAGTTGACCTAATGTCTTATTGGGCTCCTGTCTGCAGGATTTACGCGCACGT

TGGAACCGAAGAGAGCTCTGTTGTTGCAATGTTCAGCCCACAAGAGCTTACTGGTGAAGGAATGGGACAAGACCCATCTTTATGCAAAGCCAGCGTT
ACAGTAATGTTCCAGCATCTCATAATCTATCCTGGGGAATTCAGCTGCCTCCCAGGGTGAATACAGGTATTCCTGATGACAGTCTGCCTCTATCTTACAG
AGCAGCTTGTTGCTATATACCATTGAAAAGCCTTCAGAGCTGAGAGGTACTACTAACCAATAACCTGCTTGGCTCAAAGGGCCAGCACCTTCTCTCTAA
AGCCCAAGAGGAGTTTGAGGAAAACTAGGTGTCTGTGTTCACTCCAGGCTGAAGTTACAGGTCTGAGCAAATAAGGTGTATAAAAAATGGAATCTGTC
TTGGAGGACATCAGAAGGTGAATTTTCCAAGTTCTTGGACAACCTAGCTGTTGAAAAGCTTTCTGGGTTTGGGGGGTATTTCAGATGTACCTTAAAGTG
TTAGCAGACACAGATTAAGACACTGGGAGCCAATGAAACAGCAGTTGAGGGTTTGCTGTGTATCACATTTCTGTATTTTATCACCCCCTTCCTGCAACA
TTATTTATCTGGAATCTACCTGCCCTTTTGTTTTTTAGATACAAGGGCTTGGTTTTGTTACCCAGGCTGGTTTCAAGGCCATAGCTTTAAGAGATCCTCTC
ACCACAGATTTCCAAAGTGCTGGGATTGCAGGTGTGATTCATGGCACCCAGACTTTGCTGCCTTTCTTACATGATCCAGGCCCAGAACCCAAACTCAG
GCACTGTATAGATGACCACTTTCGTAAACTACTGACCTAGCTTGTTGCCAATTGTTGATTGAACTTCCCATAACTCCACTTCGTGTCTGTTCCTCTGTATA
CAGCCACCTTCTGTTCCCGTCATGAGCCTTTAGGTCTCCATTTGCATATTGCAAATACTATGTTCCATGTAGGTAGCTCATTCAGGGCCTTGCTCTTCACT
TCAAAAAAGGTTCCCTTGAGGACTGGCTGTCAATTTGTGTTGCTGTGTTGGTTGTTGATGAAAATAATAAAATGATTGATTACATA
>ENST00000537925.5|ENSG00000255717.6|OTTHUMG00000167743.1|OTTHUMT00000396017.1|RP11-727F15.10-002|SNHG1|1487|
GTGGTAAGTGGCTTCGTGGTCTTTATAGCTGTTACTCTTTTGTACTTTGTCTTTTTCTTTTATTTTCTTTTGAGCGATTGTGCGAACATAGCATAGCACGCA
CTATGCCTTCTGTGTTGTAGCTGCCTGGCCAGGGCGACTGGCGGATAAGGTCTTGTGCGTGGCCTCGAGGCTTAAAAGTAGCAGTGGGGCTTTGTGAA
GGACAAAATGGCGATGGCGGGCCGTGTAGGTCCCCCTTCCTATGATGAGGACCTTTTCACAGACCTGTACTGAGCTCCGTGAGGATAAATAACTCTGA
GGAGATGGGCCCTGCAAGCCTCTTGCTTAGCCGTCTGTTCAGAAAATAGCGTTTTCGAAATGCCCTGAGTTGACCTAATGTCTTATTGGGCTCCTGTCT
GCAGGATTTACGCGCACGTTGGAACCGAAGAGAGCTCTGTTGTTGCACCCACAAGAGCTTACTGGTGAAGGAATGGGACAAGACCCATCTTTATGCA
AAGCCAGCGTTACAGTAATGTTCCAGCATCTCATAATCTATCCTGGGGAATTCAGCTGCCTCCCAGGGTGAATACAGGTATTCCTGATGACAGTCTGCCT
CTATCTTACAGAGCAGCTTGTTGCTATATACCATTGAAAAGCCTTCAGAGCTGAGAGGTACTACTAACCAATAACCTGCTTGGCTCAAAGGGCCAGCAC
CTTCTCTCTAAAGCCCAAGAGGAGTTTGAGGAAAACTAGGTGTCTGTGTTCACTCCAGGCTGAAGTTACAGGTCTGAGCAAATAAGGTGTATAAAAAA
TGGAATCTGTCTTGGAGGACATCAGAAGGTGAATTTTCCAAGTTCTTGGACAACCTAGCTGTTGAAAAGCTTTCTGGGTTTGGGGGGTATTTCAGATGT
ACCTTAAAGTGTTAGCAGACACAGATTAAGACACTGGGAGCCAATGAAACAGCAGTTGAGGGTTTGCTGTGTATCACATTTCTGTATTTTATCACCCCC
TTCCTGCAACATTATTTATCTGGAATCTACCTGCCCTTTTGTTTTTTAGATACAAGGGCTTGGTTTTGTTACCCAGGCTGGTTTCAAGGCCATAGCTTTAA
GAGATCCTCTCACCACAGATTTCCAAAGTGCTGGGATTGCAGGTGTGATTCATGGCACCCAGACTTTGCTGCCTTTCTTACATGATCCAGGCCCAGAAC
CCAAACTCAGGCACTGTATAGATGACCACTTTCGTAAACTACTGACCTAGCTTGTTGCCAATTGTTGATTGAACTTCCCATAACTCCACTTCGTGTCTGT
TCCTCTGTATACAGCCACCTTCTGTTCCCGTCATGAGCCTTTAGGTCTCCATTTGCATATTGCAAATACTATGTTCCATGTAGGTAGCTCATTCAGGGCCT

TGCTCTTCACTTCAAAAAAGGTTCCCTTGAGGACTGGCTGTCAATTTGTGTTGCTGTGTTGGTTGTTGATGAAAATAATAAAATGATTGATTACATA
>ENST00000537068.5|ENSG00000255717.6|OTTHUMG00000167743.1|OTTHUMT00000396018.1|RP11-727F15.10-025|SNHG1|629|
TTTTTCTACTGCTCGTGGATTTACGCGCACGTTGGAACCGAAGAGAGCTCTGTTGTTGCAATGTTCAGCCCACAAGAGCTTACTGGTGAAGGAATGGG
ACAAGACCCATCTTTATGCAAAGCCAGCGTTACAGTAATGTTCCAGGTATTCCTGATGACAGTCTGCCTCTATCTTACAGAGCAGCTTGTTGCTATATAC
CATTGAAAAGCCTTCAGAGCTGAGAGGTACTACTAACCAATAACCTGCTTGGCTCAAAGGGCCAGCACCTTCTCTCTAAAGCCCAAGAGGAGTTTGAG
GAAAACTAGGTGTCTGTGTTCACTCCAGGCTGAAGTTACAGGTGAGCACTGTATAGATGACCACTTTCGTAAACTACTGACCTAGCTTGTTGCCAATTG
TTGATTGAACTTCCCATAACTCCACTTCGTGTCTGTTCCTCTGTATACAGCCACCTTCTGTTCCCGTCATGAGCCTTTAGGTCTCCATTTGCATATTGCAA
ATACTATGTTCCATGTAGGTAGCTCATTCAGGGCCTTGCTCTTCACTTCAAAAAAGGTTCCCTTGAGGACTGGCTGTCAATTTGTGTTGCTGTGTTGGTT
GTTGATGAAAATAATAAAATGATTGATTACATA
>ENST00000538654.5|ENSG00000255717.6|OTTHUMG00000167743.1|OTTHUMT00000396019.1|RP11-727F15.10-003|SNHG1|2768|
CTCATTTTTCTACTGCTCGTGGTAAGTGGCTTCGTGGTCTTTATAGCTGTTACTCTTTTGTACTTTGTCTTTTTCTTTTATTTTCTTTTGAGCGATTGTGCG
AACATAGCATAGCACGCACTATGCCTTCTGTGTTGTAGCTGCCTGGCCAGGGCGACTGGCGGATAAGGTCTTGTGCGTGGCCTCGAGGCTTAAAAGTA
GCAGTGGGGCTTTGTGAAGGACAAAATGGCGATGGCGGGCCGTGTAGGTCCCCCTTCCTATGATGAGGACCTTTTCACAGACCTGTACTGAGCTCCGT
GAGGATAAATAACTCTGAGGAGATGGGCCCTGCAAGCCTCTTGCTTAGCCGTCTGTTCAGAAAATAGCGTTTTCGAAATGCCCTGAGTTGACCTAATGT
CTTATTGGGCTCCTGTCTGCAGGATTTACGCGCACGTTGGAACCGAAGAGAGCTCTGTTGTTGCAGTAAGTTCTTACGGCCATTTCTTAATCTCTGCTCT
TTCGTTGAGTGTGTGGAAGTTGCTACGGGGATGATTTTACGAACTGAACTCTCTCTTTCTGATGGATTAGTGGAGAAAACAGAAAATTCTGAGTAGCAC
TGTACTGTACGCAACAAATGTCAGGGCCCTATTGATTTGTCTGAGGTGTTAGTGAAGGGTGTTATTTTTTCAGATGTTCAGCCCACAAGAGCTTACTGGT
GAAGGAATGGGACAAGACCCATCTTTATGCAAAGCCAGCGTTACAGTAATGTTCCAGGTAGGTGTACATGGTTTATGCTCTTACAGAGGAGACCTTGTA
GATAACCACTCCATGATGAACACAAAATGACAAGCATATGGCTGAACTTTCAAGTGATGTCATCTTACTACTGAGAAGTGAGAGAGAGGTCTTAAGGG
GTCTTTGAATGACTATTTTTAGGTACATAAAATGCTTTCCTCTGTTGTCTACAGCATCTCATAATCTATCCTGGGGAATTCAGCTGCCTCCCAGGGTGAAT
ACAGGTATTCCTGATGACAGTCTGCCTCTATCTTACAGAGCAGCTTGTTGCTATATACCATTGAAAAGCCTTCAGAGCTGAGAGGTTAGTTGATATTTTT
TGTTCCTTACAGCTTATGCCACCAAGTAGGCAGTTTCTATGATGAATCAAACTAGCTCACTATGACCGACAGTGAAAATACATGAACACCTGAGAAACT
GGAGAACGCAGGGAGTGGGGGGTAACCATGTCTGAGGAATCTTTCACCCACAGCTTTGTTTTTCTCTAGGTACTACTAACCAATAACCTGCTTGGCTCA
AAGGGCCAGCACCTTCTCTCTAAAGCCCAAGAGGAGTTTGAGGTAAATGGCTTTGCAATAGTTACCATCAATGGCTGCTATATAAAATTTTCTGTGATTT
TTGTGTGTGATAGCACTGTGGTCTGGGTGAATGTACACAGACATAACTGGCTTAACCCAAAGTCTTTGATCTCCTGAACCACCAGTGATGAATTGCTGC
TCACCAGTGATGAGTTGAATACCGCCCCAGTCTGATCAATGTGTGACTGAAAGGTATTTTCTGAGCTGTGAGCCTGCCTTCCAGTGACATGTTCTAAAA

ATTGCAAGTTATTTGAGGAGGCTTTACAGCCAATAGGAAGTTCTTGGGCTAAGTAGTGTTTCCTATAAAATGTGCCCTGAAACTTCTTTCTGCCAAGCAA
TAGATACAATTGAGAGATTGTAAAATGTGACATAGAATGAAAGTCTCTCAAGACTACATTTTTCTTTCTTGATTTTGCAGGAAAACTAGGTGTCTGTGTT
CACTCCAGGCTGAAGTTACAGGTGAGTAAACCTAAATGTAAGGTGGACTATGCTAAAAATTCCCAATGAAGAAACTTTCACATGTCTTACTCTCTGTCC
TAGTCCCAGAGCCTGTAAAGGTGAACCCACTGGGACTGGCTGGGGGAGAAGAGGAAGATTTGTTCCAGAAGGAACTGTCTGAGGGATGATAAAGATT
TCTATACAGAGAAAGGGAGTAATCATCACTTGTTGAAAACATTGGTTTTATTTTTTCCAGGTCTGAGCAAATAAGGTGTATAAAAAATGGAATCTGTCTT
GGAGGACATCAGAAGGTGAATTTTCCAAGTTCTTGGACAACCTAGCTGTTGAAAAGCTTTCTGGGTTTGGGGGGTATTTCAGATGTACCTTAAAGTGTT
AGCAGACACAGATTAAGACACTGGGAGCCAATGAAACAGCAGTTGAGGGTTTGCTGTGTATCACATTTCTGTATTTTATCACCCCCTTCCTGCAACATT
ATTTATCTGGAATCTACCTGCCCTTTTGTTTTTTAGATACAAGGGCTTGGTTTTGTTACCCAGGCTGGTTTCAAGGCCATAGCTTTAAGAGATCCTCTCAC
CACAGATTTCCAAAGTGCTGGGATTGCAGGTGTGATTCATGGCACCCAGACTTTGCTGCCTTTCTTACATGATCCAGGCCCAGAACCCAAACTCAGGC
ACTGTATAGATGACCACTTTCGTAAACTACTGACCTAGCTTGTTGCCAATTGTTGATTGAACTTCCCATAACTCCACTTCGTGTCTGTTCCTCTGTATACA
GCCACCTTCTGTTCCCGTCATGAGCCTTTAGGTCTCCATTTGCATATTGCAAATACTATGTTCCATGTAGGTAGCTCATTCAGGGCCTTGCTCTTCACTTC
AAAAAAGGTTCCCTTGAGGACTGGCTGTCAATTTGTGTTGCTGTGTTGGTTGTTGATGAAAATAATAAAATGATTGATTACAT
>ENST00000537869.5|ENSG00000255717.6|OTTHUMG00000167743.1|OTTHUMT00000396020.1|RP11-727F15.10-004|SNHG1|1038|
CGCGCACGTTGGAACCGAAGAGAGCTCTGTTGTTGCAATGTTCAGCCCACAAGAGCTTACTGGTGAAGGAATGGGACAAGACCCATCTTTATGCAAA
GCCAGCGTTACAGTAATGTTCCAGCATCTCATAATCTATCCTGGGGAATTCAGCTGCCTCCCAGGGTGAATACAGGTATTCCTGATGACAGTCTGCCTCT
ATCTTACAGTACTACTAACCAATAACCTGCTTGGCTCAAAGGGCCAGCACCTTCTCTCTAAAGCCCAAGAGGAGTTTGAGGAAAACTAGGTGTCTGTG
TTCACTCCAGGCTGAAGTTACAGGTCTGAGCAAATAAGGTGTATAAAAAATGGAATCTGTCTTGGAGGACATCAGAAGGTGAATTTTCCAAGTTCTTG
GACAACCTAGCTGTTGAAAAGCTTTCTGGGTTTGGGGGGTATTTCAGATGTACCTTAAAGTGTTAGCAGACACAGATTAAGACACTGGGAGCCAATGA
AACAGCAGTTGAGGGTTTGCTGTGTATCACATTTCTGTATTTTATCACCCCCTTCCTGCAACATTATTTATCTGGAATCTACCTGCCCTTTTGTTTTTTAGA
TACAAGGGCTTGGTTTTGTTACCCAGGCTGGTTTCAAGGCCATAGCTTTAAGAGATCCTCTCACCACAGATTTCCAAAGTGCTGGGATTGCAGGTGTGA
TTCATGGCACCCAGACTTTGCTGCCTTTCTTACATGATCCAGGCCCAGAACCCAAACTCAGGCACTGTATAGATGACCACTTTCGTAAACTACTGACCT
AGCTTGTTGCCAATTGTTGATTGAACTTCCCATAACTCCACTTCGTGTCTGTTCCTCTGTATACAGCCACCTTCTGTTCCCGTCATGAGCCTTTAGGTCTC
CATTTGCATATTGCAAATACTATGTTCCATGTAGGTAGCTCATTCAGGGCCTTGCTCTTCACTTCAAAAAAGGTTCCCTTGAGGACTGGCTGTCAATTTG
TGTTGCTGTGTTGGTTGTTGATGAAAATAATAAAATGATTGATTAC
>ENST00000539975.5|ENSG00000255717.6|OTTHUMG00000167743.1|OTTHUMT00000396021.1|RP11-727F15.10-005|SNHG1|955|
GAAGGGTGTTATTTTTTCAGATGTTCAGCCCACAAGAGCTTACTGGTGAAGGAATGGGACAAGACCCATCTTTATGCAAAGCCAGCGTTACAGTAATGT

TCCAGGAGCAGCTTGTTGCTATATACCATTGAAAAGCCTTCAGAGCTGAGAGGTACTACTAACCAATAACCTGCTTGGCTCAAAGGGCCAGCACCTTCT
CTCTAAAGCCCAAGAGGAGTTTGAGGAAAACTAGGTGTCTGTGTTCACTCCAGGCTGAAGTTACAGGTCTGAGCAAATAAGGTGTATAAAAAATGGAA
TCTGTCTTGGAGGACATCAGAAGGTGAATTTTCCAAGTTCTTGGACAACCTAGCTGTTGAAAAGCTTTCTGGGTTTGGGGGGTATTTCAGATGTACCTT
AAAGTGTTAGCAGACACAGATTAAGACACTGGGAGCCAATGAAACAGCAGTTGAGGGTTTGCTGTGTATCACATTTCTGTATTTTATCACCCCCTTCCT
GCAACATTATTTATCTGGAATCTACCTGCCCTTTTGTTTTTTAGATACAAGGGCTTGGTTTTGTTACCCAGGCTGGTTTCAAGGCCATAGCTTTAAGAGAT
CCTCTCACCACAGATTTCCAAAGTGCTGGGATTGCAGGTGTGATTCATGGCACCCAGACTTTGCTGCCTTTCTTACATGATCCAGGCCCAGAACCCAAA
CTCAGGCACTGTATAGATGACCACTTTCGTAAACTACTGACCTAGCTTGTTGCCAATTGTTGATTGAACTTCCCATAACTCCACTTCGTGTCTGTTCCTCT
GTATACAGCCACCTTCTGTTCCCGTCATGAGCCTTTAGGTCTCCATTTGCATATTGCAAATACTATGTTCCATGTAGGTAGCTCATTCAGGGCCTTGCTCT
TCACTTCAAAAAAGGTTCCCTTGAGGACTGGCTGTCAATTTGTGTTGCTGTGTTGGTTG
>ENST00000535076.5|ENSG00000255717.6|OTTHUMG00000167743.1|OTTHUMT00000396022.1|RP11-727F15.10-022|SNHG1|849|
TGACTTGCTGTTGAGACTCTGAAATCTGATTTTCTGAGAATGATGGGTGGGAACAACATAATGCGGGATATGAGAAGCACTACTGACTTGGTCTTCCTC
CTTTCAGGGCCAGCACCTTCTCTCTAAAGCCCAAGAGGAGTTTGAGGAAAACTAGGTGTCTGTGTTCACTCCAGGCTGAAGTTACAGGTCTGAGCAA
ATAAGGTGTATAAAAAATGGAATCTGTCTTGGAGGACATCAGAAGGTGAATTTTCCAAGTTCTTGGACAACCTAGCTGTTGAAAAGCTTTCTGGGTTTG
GGGGGTATTTCAGATGTACCTTAAAGTGTTAGCAGACACAGATTAAGACACTGGGAGCCAATGAAACAGCAGTTGAGGGTTTGCTGTGTATCACATTTC
TGTATTTTATCACCCCCTTCCTGCAACATTATTTATCTGGAATCTACCTGCCCTTTTGTTTTTTAGATACAAGGGCTTGGTTTTGTTACCCAGGCTGGTTTC
AAGGCCATAGCTTTAAGAGATCCTCTCACCACAGATTTCCAAAGTGCTGGGATTGCAGGTGTGATTCATGGCACCCAGACTTTGCTGCCTTTCTTACAT
GATCCAGGCCCAGAACCCAAACTCAGGCACTGTATAGATGACCACTTTCGTAAACTACTGACCTAGCTTGTTGCCAATTGTTGATTGAACTTCCCATAA
CTCCACTTCGTGTCTGTTCCTCTGTATACAGCCACCTTCTGTTCCCGTCATGAGCCTTTAGGTCTCCATTTGCATATTGCAAATACTATGTTCCATGTAGGT
AGCTCATTCAGGGCCTTGCTCTTCACTTCAAAAAAGGTTCCCTTGAGGACTGG
>ENST00000545440.5|ENSG00000255717.6|OTTHUMG00000167743.1|OTTHUMT00000396023.1|RP11-727F15.10-014|SNHG1|754|
CTTATTGGGCTCCTGTCTGCAGGATTTACGCGCACGTTGGAACCGAAGAGAGCTCTGTTGTTGCAATGTTCAGCCCACAAGAGCTTACTGGTGAAGGA
ATGGGACAAGACCCATCTTTATGCAAAGCCAGCGTTACAGTAATGTTCCAGGTATTCCTGATGACAGTCTGCCTCTATCTTACAGTACTACTAACCAATA
ACCTGCTTGGCTCAAAGGGCCAGCACCTTCTCTCTAAAGCCCAAGAGGAGTTTGAGGAAAACTAGGTGTCTGTGTTCACTCCAGGCTGAAGTTACAG
GTCTGAGCAAATAAGGTGTATAAAAAATGGAATCTGTCTTGGAGGACATCAGAAGGTGAATTTTCCAAGTTCTTGGACAACCTAGCTGTTGAAAAGCT
TTCTGGGTTTGGGGGGTATTTCAGATGTACCTTAAAGTGTTAGCAGACACAGATTAAGACACTGGGAGCCAATGAAACAGCAGTTGAGGGTTTGCTGT
GTATCACATTTCTGTATTTTATCACCCCCTTCCTGCAACATTATTTATCTGGAATCTACCTGCCCTTTTGTTTTTTAGATACAAGGGCTTGGTTTTGTTACCC

AGGCTGGTTTCAAGGCCATAGCTTTAAGAGATCCTCTCACCACAGATTTCCAAAGTGCTGGGATTGCAGGTGTGATTCATGGCACCCAGACTTTGCTGC
CTTTCTTACATGATCCAGGCCCAGAACCCAAACTCAGGCACTGTATAGATGACCACTTTCG
>ENST00000542112.5|ENSG00000255717.6|OTTHUMG00000167743.1|OTTHUMT00000396024.1|RP11-727F15.10-007|SNHG1|780|
ATTTAATCGTCTCCCCGGGGAGATTCGTTCTCATTTTTCTACTGCTCGTGATGTTCAGCCCACAAGAGCTTACTGGTGAAGGAATGGGACAAGACCCAT
CTTTATGCAAAGCCAGCGTTACAGTAATGTTCCAGGTATTCCTGATGACAGTCTGCCTCTATCTTACAGAGCAGCTTGTTGCTATATACCATTGAAAAGC
CTTCAGAGCTGAGAGGTACTACTAACCAATAACCTGCTTGGCTCAAAGGGCCAGCACCTTCTCTCTAAAGCCCAAGAGGAGTTTGAGGAAAACTAGG
TGTCTGTGTTCACTCCAGGCTGAAGTTACAGGTCTGAGCAAATAAGGTGTATAAAAAATGGAATCTGTCTTGGAGGACATCAGAAGGTGAATTTTCCA
AGTTCTTGGACAACCTAGCTGTTGAAAAGCTTTCTGGGTTTGGGGGGTATTTCAGATGTACCTTAAAGTGTTAGCAGACACAGATTAAGACACTGGGA
GCCAATGAAACAGCAGTTGAGGGTTTGCTGTGTATCACATTTCTGTATTTTATCACCCCCTTCCTGCAACATTATTTATCTGGAATCTACCTGCCCTTTTG
TTTTTTAGATACAAGGGCTTGGTTTTGTTACCCAGGCTGGTTTCAAGGCCATAGCTTTAAGAGATCCTCTCACCACAGATTTCCAAAGTGCTGGGATTGC
AGGTGTGATTCATGGCACCCAGACTTTGCTGCCTTTCTTACATGATCCAGGCCCAGAACCCAAACTCAGGCACTGTATAGATGACCA
>ENST00000541615.5|ENSG00000255717.6|OTTHUMG00000167743.1|OTTHUMT00000396025.1|RP11-727F15.10-020|SNHG1|748|
CTTATTGGGCTCCTGTCTGCAGGATTTACGCGCACGTTGGAACCGAAGAGAGCTCTGTTGTTGCAATGTTCAGCCCACAAGAGCTTACTGGTGAAGGA
ATGGGACAAGACCCATCTTTATGCAAAGCCAGCGTTACAGTAATGTTCCAGGTATTCCTGATGACAGTCTGCCTCTATCTTACAGAGCAGCTTGTTGCTA
TATACCATTGAAAAGCCTTCAGAGCTGAGAGGTACTACTAACCAATAACCTGCTTGGCTCAAAGGGCCAGCACCTTCTCTCTAAAGCCCAAGAGGAGT
TTGAGGAAAACTAGGTGTCTGTGTTCACTCCAGGCTGAAGTTACAGGTCTGAGCAAATAAGGTGTATAAAAAATGGACATCAGAAGGTGAATTTTCCA
AGTTCTTGGACAACCTAGCTGTTGAAAAGCTTTCTGGGTTTGGGGGGTATTTCAGATGTACCTTAAAGTGTTAGCAGACACAGATTAAGACACTGGGA
GCCAATGAAACAGCAGTTGAGGGTTTGCTGTGTATCACATTTCTGTATTTTATCACCCCCTTCCTGCAACATTATTTATCTGGAATCTACCTGCCCTTTTG
TTTTTTAGATACAAGGGCTTGGTTTTGTTACCCAGGCTGGTTTCAAGGCCATAGCTTTAAGAGATCCTCTCACCACAGATTTCCAAAGTGCTGGGATTGC
AGGTGTGATTCATGGCACCCAGACTTTGCTGCCTTTCTTACATGATCCAGGCCCA
>ENST00000541578.5|ENSG00000255717.6|OTTHUMG00000167743.1|OTTHUMT00000396026.1|RP11-727F15.10-021|SNHG1|588|
CTTATTGGGCTCCTGTCTGCAGGATTTACGCGCACGTTGGAACCGAAGAGAGCTCTGTTGTTGCAATGTTCAGCCCACAAGAGCTTACTGGTGAAGGA
ATGGGACAAGACCCATCTTTATGCAAAGCCAGCGTTACAGTAATGTTCCAGCATCTCATAATCTATCCTGGGGAATTCAGCTGCCTCCCAGGGTGAATAC
AGGTATTCCTGATGACAGTCTGCCTCTATCTTACAGAGCAGCTTGTTGCTATATACCATTGAAAAGCCTTCAGAGCTGAGAGGTACTACTAACCAATAAC
CTGCTTGGCTCAAAGGGCCAGCACCTTCTCTCTAAAGCCCAAGAGGAGTTTGAGGAAAACTAGGTGTCTGTGTTCACTCCAGGCTGAAGTTACAGGT
CTGAGCAAATAAGGTGTATAAAAAATGGAATCTGTCTTGGAGGACATCAGAAGATACAAGGGCTTGGTTTTGTTACCCAGGCTGGTTTCAAGGCCATA

GCTTTAAGAGATCCTCTCACCACAGATTTCCAAAGTGCTGGGATTGCAGGTGTGATTCATGGCACCCAGACTTTGCTGCCTTTCTTACATGATCC
>ENST00000538266.5|ENSG00000255717.6|OTTHUMG00000167743.1|OTTHUMT00000396027.1|RP11-727F15.10-019|SNHG1|347|
GGATTTACGCGCACGTTGGAACCGAAGAGAGCTCTGTTGTTGCAATGTTCAGCCCACAAGAGCTTACTGGTGAAGGAATGGGACAAGACCCATCTTTA
TGCAAAGCCAGCGTTACAGTAATGTTCCAGGTATTCCTGATGACAGTCTGCCTCTATCTTACAGTACTACTAACCAATAACCTGCTTGGCTCAAAGGGCC
AGCACCTTCTCTCTAAAGCCCAAGAGGAGTTTGAGGAAAACTAGGTGTCTGTGTTCACTCCAGGCTGAAGTTACAGGAATCTGTCTTGGAGGACATCA
GAAGGTGAATTTTCCAAGTTCTTGGACAACCTAGCTGTTGAAAAGCTTTCT
>ENST00000545688.5|ENSG00000255717.6|OTTHUMG00000167743.1|OTTHUMT00000396028.1|RP11-727F15.10-015|SNHG1|610|
CGCGCACGTTGGAACCGAAGAGAGCTCTGTTGTTGCAATGTTCAGCCCACAAGAGCTTACTGGTGAAGGAATGGGACAAGACCCATCTTTATGCAAA
GCCAGCGTTACAGTAATGTTCCAGCATCTCATAATCTATCCTGGGGAATTCAGCTGCCTCCCAGGGTGAATACAGGTATTCCTGATGACAGTCTGCCTCT
ATCTTACAGAGCAGCTTGTTGCTATATACCATTGAAAAGCCTTCAGAGCTGAGAGGTACTACTAACCAATAACCTGCTTGGCTCAAAGGGCCAGCACCT
TCTCTCTAAAGCCCAAGAGGAGTTTGAGGAAAACTAGGTGTCTGTGTTCACTCCAGGCTGAAGTTACAGTCCCAGAGCCTGTAAAGGTGAACCCACT
GGGACTGGCTGGGGGAGAAGAGGAAGATTTGTTCCAGAAGGAACTGTCTGAGGGATGATAAAGATTTCTATACAGAGAAAGGGAGTAATCATCACTT
GTTGAAAACATTGGTTTTATTTTTTCCAGGTCTGAGCAAATAAGGTGTATAAAAAATGGTAGGTATCTGTTTAAATATTTAACTGATTTTATAATAGCAAC
CTTTTGCTCCTAGGTTACA
>ENST00000544550.5|ENSG00000255717.6|OTTHUMG00000167743.1|OTTHUMT00000396029.1|RP11-727F15.10-018|SNHG1|636|
CTTATTGGGCTCCTGTCTGCAGGATTTACGCGCACGTTGGAACCGAAGAGAGCTCTGTTGTTGCAATGTTCAGCCCACAAGAGCTTACTGGTGAAGGA
ATGGGACAAGACCCATCTTTATGCAAAGCCAGCGTTACAGTAATGTTCCAGCATCTCATAATCTATCCTGGGGAATTCAGCTGCCTCCCAGGGTGAATAC
AGGTATTCCTGATGACAGTCTGCCTCTATCTTACAGAGCAGCTTGTTGCTATATACCATTGAAAAGCCTTCAGAGCTGAGAGGTACTACTAACCAATAAC
CTGCTTGGCTCAAAGGGCCAGCACCTTCTCTCTAAAGCCCAAGAGGAGTTTGAGGAAAACTAGGTGTCTGTGTTCACTCCAGGCTGAAGTTACAGGT
GAGTAAACCTAAATGTAAGGTGGACTATGCTAAAAATTCCCAATGAAGAAACTTTCACATGTCTTACTCTCTGTCCTAGTCCCAGAGCCTGTAAAGGTG
AACCCACTGGGACTGGCTGGGGGAGAAGAGGAAGATTTGTTCCAGAAGGAACTGTCTGAGGGATGATAAAGATTTCTATACAGAGAAAGGGAGTAAT
CATCACTTGTTGAAAACATTGGTTTTATTTTTTCCAGGTCTGAGC
>ENST00000545920.1|ENSG00000255717.6|OTTHUMG00000167743.1|OTTHUMT00000396030.1|RP11-727F15.10-024|SNHG1|625|
CACTGATCTGCCCAGGGTCAGACGAATGCTTGTCAGTTGTAGCTTTCCAGGATTCTGCTCCAATGAGGAGGAAACATCCTGCCTTACCCCTGTTTTAGC
CTGGGAACCAGTAATTGTGAACTCACCAGGGTTAACATGAAGAGGGCATGAGAGCTTATTCCATAAGGAATTGTCTGAGACATTTGGTTACCTTTTCTT
AGTTTGTCCATTAGCAACCGTAACTAAAATAGTTTTGGCTGTTTGGGATAATTAGCTAATTGAATGATTTTTTGATTCAGGAGCAGCTTGTTGCTATATAC

CATTGAAAAGCCTTCAGAGCTGAGAGGTTAGTTGATATTTTTTGTTCCTTACAGCTTATGCCACCAAGTAGGCAGTTTCTATGATGAATCAAACTAGCTC
ACTATGACCGACAGTGAAAATACATGAACACCTGAGAAACTGGAGAACGCAGGGAGTGGGGGGTAACCATGTCTGAGGAATCTTTCACCCACAGCTT
TGTTTTTCTCTAGGTACTACTAACCAATAACCTGCTTGGCTCAAAGGGCCAGCACCTTCTCTCTAAAGCCCAAGAGGAGTTTGAGGAAAACTAGGTGT
CTGTGTTCACTCCAGGCTGAAGTTACAGGTG
>ENST00000540865.5|ENSG00000255717.6|OTTHUMG00000167743.1|OTTHUMT00000396031.1|RP11-727F15.10-017|SNHG1|784|
CTTATTGGGCTCCTGTCTGCAGGATTTACGCGCACGTTGGAACCGAAGAGAGCTCTGTTGTTGCAATGTTCAGCCCACAAGAGCTTACTGGTGAAGGA
ATGGGACAAGACCCATCTTTATGCAAAGCCAGCGTTACAGTAATGTTCCAGCATCTCATAATCTATCCTGGGGAATTCAGCTGCCTCCCAGGGTGAATAC
AGGTATTCCTGATGACAGTCTGCCTCTATCTTACAGTACTACTAACCAATAACCTGCTTGGCTCAAAGGGCCAGCACCTTCTCTCTAAAGCCCAAGAGG
AGTTTGAGGTAAATGGCTTTGCAATAGTTACCATCAATGGCTGCTATATAAAATTTTCTGTGATTTTTGTGTGTGATAGCACTGTGGTCTGGGTGAATGTA
CACAGACATAACTGGCTTAACCCAAAGTCTTTGATCTCCTGAACCACCAGTGATGAATTGCTGCTCACCAGTGATGAGTTGAATACCGCCCCAGTCTGA
TCAATGTGTGACTGAAAGGTATTTTCTGAGCTGTGAGCCTGCCTTCCAGTGACATGTTCTAAAAATTGCAAGTTATTTGAGGAGGCTTTACAGCCAATA
GGAAGTTCTTGGGCTAAGTAGTGTTTCCTATAAAATGTGCCCTGAAACTTCTTTCTGCCAAGCAATAGATACAATTGAGAGATTGTAAAATGTGACATAG
AATGAAAGTCTCTCAAGACTACATTTTTCTTTCTTGATTTTGCAGGAAAACTAGGTGTCTGTGTTCACTCCAGGCTGAAGTTACAGGT
>ENST00000535689.5|ENSG00000255717.6|OTTHUMG00000167743.1|OTTHUMT00000396032.1|RP11-727F15.10-016|SNHG1|550|
CTTATTGGGCTCCTGTCTGCAGGATTTACGCGCACGTTGGAACCGAAGAGAGCTCTGTTGTTGCAATGTTCAGCCCACAAGAGCTTACTGGTGAAGGA
ATGGGACAAGACCCATCTTTATGCAAAGCCAGCGTTACAGTAATGTTCCAGCATCTCATAATCTATCCTGGGGAATTCAGCTGCCTCCCAGGGTGAATAC
AGGTATTCCTGATGACAGTCTGCCTCTATCTTACAGAGCAGCTTGTTGCTATATACCATTGAAAAGCCTTCAGAGCTGAGAGGTACTACTAACCAATAAC
CTGCTTGGCTCAAAGGGCCAGCACCTTCTCTCTAAAGCCCAAGAGGAGTTTGAGGTAAATGGCTTTGCAATAGTTACCATCAATGGCTGCTATATAAAA
TTTTCTGTGATTTTTGTGTGTGATAGCACTGTGGTCTGGGTGAATGTACACAGACATAACTGGCTTAACCCAAAGTCTTTGATCTCCTGAACCACCAGTG
ATGAATTGCTGCTCACCAGTGATGAGTTGAATACCGCCCCAGTCTGATCAATG
>ENST00000537024.5|ENSG00000255717.6|OTTHUMG00000167743.1|OTTHUMT00000396033.1|RP11-727F15.10-013|SNHG1|532|
CTCCTGTCTGCAGGATTTACGCGCACGTTGGAACCGAAGAGAGCTCTGTTGTTGCAATGTTCAGCCCACAAGAGCTTACTGGTGAAGGAATGGGACA
AGACCCATCTTTATGCAAAGCCAGCGTTACAGTAATGTTCCAGCATCTCATAATCTATCCTGGGGAATTCAGCTGCCTCCCAGGGTGAATACAGGTATTC
CTGATGACAGTCTGCCTCTATCTTACAGAGCAGCTTGTTGCTATATACCATTGAAAAGCCTTCAGAGCTGAGAGGTTAGTTGATATTTTTTGTTCCTTACA
GCTTATGCCACCAAGTAGGCAGTTTCTATGATGAATCAAACTAGCTCACTATGACCGACAGTGAAAATACATGAACACCTGAGAAACTGGAGAACGCA
GGGAGTGGGGGGTAACCATGTCTGAGGAATCTTTCACCCACAGCTTTGTTTTTCTCTAGGTACTACTAACCAATAACCTGCTTGGCTCAAAGGGCCAGC

ACCTTCTCTCTAAAGCCCAAGAGGAGTTTGAGGTAAA
>ENST00000539303.5|ENSG00000255717.6|OTTHUMG00000167743.1|OTTHUMT00000396034.1|RP11-727F15.10-023|SNHG1|240|
GACCCATCTTTATGCAAAGCCAGCGTTACAGTAATGTTCCAGCATCTCATAATCTATCCTGGGGAATTCAGCTGCCTCCCAGGGTGAATACAGGTATTCC
TGATGACAGTCTGCCTCTATCTTACAGAGCAGCTTGTTGCTATATACCATTGAAAAGCCTTCAGAGCTGAGAGCTTTGTTTTTCTCTAGGTACTACTAAC
CAATAACCTGCTTGGCTCAAAGGGCCAGCACCTTCTCTCT
>ENST00000541416.5|ENSG00000255717.6|OTTHUMG00000167743.1|OTTHUMT00000396035.1|RP11-727F15.10-008|SNHG1|996|
TCTTTTATTTTCTTTTGAGCGATTGTGCGAACATAGCATAGCACGCACTATGCCTTCTGTGTTGTAGCTGCCTGGCCAGGGCGACTGGCGGATAAGGTCT
TGTGCGTGGCCTCGAGGCTTAAAAGTAGCAGTGGGGCTTTGTGAAGGACAAAATGGCGATGGCGGGCCGTGTAGGTCCCCCTTCCTATGATGAGGACC
TTTTCACAGACCTGTACTGAGCTCCGTGAGGATAAATAACTCTGAGGAGATGGGCCCTGCAAGCCTCTTGCTTAGCCGTCTGTTCAGAAAATAGCGTTT
TCGAAATGCCCTGAGTTGACCTAATGTCTTATTGGGCTCCTGTCTGCAGGATTTACGCGCACGTTGGAACCGAAGAGAGCTCTGTTGTTGCAATGTTCA
GCCCACAAGAGCTTACTGGTGAAGGAATGGGACAAGACCCATCTTTATGCAAAGCCAGCGTTACAGTAATGTTCCAGCATCTCATAATCTATCCTGGGG
AATTCAGCTGCCTCCCAGGGTGAATACAGGTATTCCTGATGACAGTCTGCCTCTATCTTACAGTAAGTTGTTTTTCGAATTCTATGGGCCTATTATCTAAT
AGATAACTTAGCCATTTTGATATTTGTTACCAAAGCCTGCTTAACCCATAAAGTTTTCTGGTTCCTCTTGTTCTCTGTAGTGATGTTTCTAGATAGATGCCC
ACTGATATATGCCCCTTTTTTCTCAGATAGATGCCCACTGATCTGCCCAGGGTCAGACGAATGCTTGTCAGTTGTAGCTTTCCAGGATTCTGCTCCAATG
AGGAGGAAACATCCTGCCTTACCCCTGTTTTAGCCTGGGAACCAGTAATTGTGAACTCACCAGGGTTAACATGAAGAGGGCATGAGAGCTTATTCCATA
AGGAATTGTCTGAGACATTTGGTTACCTTTTCTTAGTTTGTCCATTAGCAACCGTAACTAAAATAGTTTTGGCTGTTTGGGATAATTAGCTAATTGAAT
>ENST00000537965.5|ENSG00000255717.6|OTTHUMG00000167743.1|OTTHUMT00000396036.1|RP11-727F15.10-011|SNHG1|578|
CTTATTGGGCTCCTGTCTGCAGGATTTACGCGCACGTTGGAACCGAAGAGAGCTCTGTTGTTGCAATGTTCAGCCCACAAGAGCTTACTGGTGAAGGA
ATGGGACAAGACCCATCTTTATGCAAAGCCAGCGTTACAGTAATGTTCCAGGTAGGTGTACATGGTTTATGCTCTTACAGAGGAGACCTTGTAGATAAC
CACTCCATGATGAACACAAAATGACAAGCATATGGCTGAACTTTCAAGTGATGTCATCTTACTACTGAGAAGTGAGAGAGAGGTCTTAAGGGGTCTTT
GAATGACTATTTTTAGGTACATAAAATGCTTTCCTCTGTTGTCTACAGCATCTCATAATCTATCCTGGGGAATTCAGCTGCCTCCCAGGGTGAATACAGGT
ATTCCTGATGACAGTCTGCCTCTATCTTACAGTAAGTTGTTTTTCGAATTCTATGGGCCTATTATCTAATAGATAACTTAGCCATTTTGATATTTGTTACCAA
AGCCTGCTTAACCCATAAAGTTTTCTGGTTCCTCTTGTTCTCTGTAGTGATGTTTCTAGATAGATGCCCACTGATATAT
>ENST00000545308.5|ENSG00000255717.6|OTTHUMG00000167743.1|OTTHUMT00000396037.1|RP11-727F15.10-012|SNHG1|646|
CTTATTGGGCTCCTGTCTGCAGGATTTACGCGCACGTTGGAACCGAAGAGAGCTCTGTTGTTGCAATGTTCAGCCCACAAGAGCTTACTGGTGAAGGA
ATGGGACAAGACCCATCTTTATGCAAAGCCAGCGTTACAGTAATGTTCCAGCATCTCATAATCTATCCTGGGGAATTCAGCTGCCTCCCAGGGTGAATAC

AGGTAATTATCCAAATTGGTGTATGTCATCTTTGTAAATGTTCAAGAAAACAAGTGGCCGGCGGAACAATGTTTTTTGGCGCCTTCCATCTTGTTCCTCT
TGAAATGATTTTCTGGGGAGGGAGATCGCAACCCGGTACTACAGTTCTTGTAAGGGTGTGCTGTTACCCTCATCCATATATCGATCACGTTACTGTCAGA
TGATTTGAATTGATAAGCTGATGTTCTGTGAGGTACAAAAGTTAATAGCATGTTAGAGTTCTGATGGCAGTTGTTGTTTATGAATAAGCTTCATTCCATCC
TAATACCTTAAAATAACTAATTTGCTTTCCTTTCCAGGTATTCCTGATGACAGTCTGCCTCTATCTTACAGTAAGTTGTTTTTCGAATTCTATGGGCCTATT
ATCTAATAGATAACTTAGCCATTTTGATATTTGTTACCAAAGCCT
>ENST00000540904.1|ENSG00000255717.6|OTTHUMG00000167743.1|OTTHUMT00000396038.1|RP11-727F15.10-010|SNHG1|594|
CTTATTGGGCTCCTGTCTGCAGGATTTACGCGCACGTTGGAACCGAAGAGAGCTCTGTTGTTGCACCCACAAGAGCTTACTGGTGAAGGAATGGGACA
AGACCCATCTTTATGCAAAGCCAGCGTTACAGTAATGTTCCAGGTAGGTGTACATGGTTTATGCTCTTACAGAGGAGACCTTGTAGATAACCACTCCATG
ATGAACACAAAATGACAAGCATATGGCTGAACTTTCAAGTGATGTCATCTTACTACTGAGAAGTGAGAGAGAGGTCTTAAGGGGTCTTTGAATGACTAT
TTTTAGGTACATAAAATGCTTTCCTCTGTTGTCTACAGCATCTCATAATCTATCCTGGGGAATTCAGCTGCCTCCCAGGGTGAATACAGGTAATTATCCAA
ATTGGTGTATGTCATCTTTGTAAATGTTCAAGAAAACAAGTGGCCGGCGGAACAATGTTTTTTGGCGCCTTCCATCTTGTTCCTCTTGAAATGATTTTCT
GGGGAGGGAGATCGCAACCCGGTACTACAGTTCTTGTAAGGGTGTGCTGTTACCCTCATCCATATATCGATCACGTTACTGTCAGATGATTTGAAT
>ENST00000544983.1|ENSG00000255717.6|OTTHUMG00000167743.1|OTTHUMT00000396039.1|RP11-727F15.10-009|SNHG1|870|
TTCTGTGTTGTAGCTGCCTGGCCAGGGCGACTGGCGGATAAGGTCTTGTGCGTGGCCTCGAGGCTTAAAAGTAGCAGTGGGGCTTTGTGAAGGACAA
AATGGCGATGGCGGGCCGTGTAGGTCCCCCTTCCTATGATGAGGACCTTTTCACAGACCTGTACTGAGCTCCGTGAGGATAAATAACTCTGAGGAGATG
GGCCCTGCAAGCCTCTTGCTTAGCCGTCTGTTCAGAAAATAGCGTTTTCGAAATGCCCTGAGTTGACCTAATGTCTTATTGGGCTCCTGTCTGCAGGATT
TACGCGCACGTTGGAACCGAAGAGAGCTCTGTTGTTGCAATGTTCAGCCCACAAGAGCTTACTGGTGAAGGAATGGGACAAGACCCATCTTTATGCAA
AGCCAGCGTTACAGTAATGTTCCAGGTAGGTGTACATGGTTTATGCTCTTACAGAGGAGACCTTGTAGATAACCACTCCATGATGAACACAAAATGACA
AGCATATGGCTGAACTTTCAAGTGATGTCATCTTACTACTGAGAAGTGAGAGAGAGGTCTTAAGGGGTCTTTGAATGACTATTTTTAGGTACATAAAATG
CTTTCCTCTGTTGTCTACAGCATCTCATAATCTATCCTGGGGAATTCAGCTGCCTCCCAGGGTGAATACAGGTAATTATCCAAATTGGTGTATGTCATCTT
TGTAAATGTTCAAGAAAACAAGTGGCCGGCGGAACAATGTTTTTTGGCGCCTTCCATCTTGTTCCTCTTGAAATGATTTTCTGGGGAGGGAGATCGCAA
CCCGGTACTACAGTTCTTGTAAGGGTGTGCTGTTACCCTCATCCATATATCGATCACGTTACTGTCAGATGATTTGA
>SNHG6
>ENST00000520348.5|ENSG00000245910.8|OTTHUMG00000164743.4|OTTHUMT00000380021.1|RP11-345I19.4-005|SNHG6|648|
CTTTCCCGCGCGACCGGCGAGGGAGGAAGAAGCGCGAAGAGCCGTTAGTCATGCCGGTGTGGTGGCGGCGGCGGAGACTGCGGGCCCGTAGCTGGG
CTCTGCGAGGTGCAAGAAAGCCTTTGAGGTGAAGGTGTATGAAAGTCATCATAACAGATGTTTTCCAAAAACTTGTAGAAGGTTGTGAAAAAACTACT

AGGATCACGCGGCATGTATTGAGGTGTGGCATGCAGCATTTTGGAAGGAAAATTGAAGACGTGTTCAAGAAAACATGAACAGAAGCAAATGATGAAA
ATGAGCATTTTACTTGATGTTGATAACATCACAATAAATTATGGAGAAAAATACATATTTGGCTAACTTTTAATTGCTGAACAATAAAGTGTTTTCTTTTA
AATCAACTCTAAATAGCTCCATTCTCATAGTCACTAGTCAGACCTGTTTTGAACATATTCGAAAGATTATAATCTTGTCAATAATTAGCTTATTTATGGGTG
GTGATTCTCATTGAGGCTGACAGCTGGGGAGACATTGCTTGTACCTCTAGGTTCCCTGTCTGGCTTCCCCTTCAGAGCCTGCTGTTGTACCAGGTGGTT
GAATCTTAAAACTCTTTAATACCAAATAGCAATCAAATTCCCCCTTACAGATAAA
>ENST00000520944.5|ENSG00000245910.8|OTTHUMG00000164743.4|OTTHUMT00000380022.1|RP11-345I19.4-003|SNHG6|638|
CGGAAGAGGCGGGGCCACCGGAGTGCCTAAGAGCTGTCTTCCGATGTCGCTCTTCCTTTCCCGCGCGACCGGCGAGGGAGGAAGAAGCGCGAAGAG
CCGTTAGTCATGCCGGTGTGGTGGCGGCGGCGGAGACTGCGGGCCCGTAGCTGGGCTCTGCGAGGTGCAAGAAAGCCTTTGAGGTGAAGGTGTATGA
AAGTCATCATAACAGATGTTTTCCAAAAACTTGTAGAAGGTTGTGAAAAAACTACTAGGATCACGCGGCATGTATTGAGCATATAGGTTGCTGTAGATG
AATGTTCTTAGCTGTCATGTTTAAAAATACTTCTGCTTCGTTACCTCAAGTGTGGCATGCAGCATTTTGGAAGGAAAATTGAAGACGTGTTCAAGAAAA
CATGAACAGAAGCAAATGATGAAAATGAGCATTTTACTTGATGTTGATAACATCACAATAAATTATGGAGAAAAATACATATTTGGCTAACTTTTAATTG
CTGAACAATAAAGTGTTTTCTTTTAAATCAACTCTAAATAGCTCCATTCTCATAGTCACTAGTCAGACCTGTTTTGAACATATTCGAAAGATTATAATCTT
GTCAATAATTAGCTTATTTATGGGTGGTGATTCTCATTGAGGCTGA
>ENST00000521399.5|ENSG00000245910.8|OTTHUMG00000164743.4|OTTHUMT00000380024.1|RP11-345I19.4-006|SNHG6|302|
CTTTCCCGCGCGACCGGCGAGGGAGGAAGAAGCGCGAAGAGCCGTTAGTCATGCCGGTGTGGTGGCGGCGGCGGAGACTGCGGGCCCGTAGCTGGG
CTCTGCGAGCATATAGGTTGCTGTAGATGAATGTTCTTAGCTGTCATGTTTAAAAATACTTCTGCTTCGTTACCTCAAGTGTGGCATGCAGCATTTTGGA
AGGAAAATTGAAGACGTGTTCAAGAAAACATGAACAGAAGCAAATGATGAAAATGAGCATTTTACTTGATGTTGATAACATCACAATAAATTATGGAG
AAAAATAC
>ENST00000520619.1|ENSG00000245910.8|OTTHUMG00000164743.4|OTTHUMT00000380025.1|RP11-345I19.4-004|SNHG6|479|
GGAGAGATGACCTATCTTTAAGTGAGTAGATTGTTTAATCATATGCTTTCATGTGTATTCTTTCTGGCAACAAGATCATAAGCCTTTCTGCTCCAGCATCA
AGCCCAGTGCTTTGCAGTCAGGATTCTATAATACAATGAGAAAAACAGAAGTAATAAGACGTGAAAATCAACAGCAAATTATAACATGTTCATAAACAA
GGTGCAAGAAAGCCTTTGAGGTGAAGGTGTATGAAAGTCATCATAACAGATGTTTTCCAAAAACTTGTAGAAGGTTGTGAAAAAACTACTAGGATCAC
GCGGCATGTATTGAGCATATAGGTTGCTGTAGATGAATGTTCTTAGCTGTCATGTTTAAAAATACTTCTGCTTCGTTACCTCAAGTGTGGCATGCAGCATT
TTGGAAGGAAAATTGAAGACGTGTTCAAGAAAACATGAACAGAAGCAAATGATGAAAATGAGCATTTTACTTGATGTTGA
>ENST00000521127.1|ENSG00000245910.8|OTTHUMG00000164743.4|OTTHUMT00000381157.1|RP11-345I19.4-007|SNHG6|560|
CTTTCCCGCGCGACCGGCGAGGGAGGAAGAAGCGCGAAGAGCCGTTAGTCATGCCGGTGTGGTGGCGGCGGCGGAGACTGCGGGCCCGTAGCTGGG

CTCTGCGAGGTGCAAGAAAGCCTTTGAGGTGAAGGTGTATGAAAGTCATCATAACAGATGTTTTCCAAAAACTTGTAGAAGGTTGTGAAAAAACTACT
AGGATCACGCGGCATGTATTGAGGTACCTAAATTTAATTAATGTTAGTTATGTGATGTATTCTTGACAGATAGGTTTAATAGCTGGCACAATGATGACTTA
AATTACTTTTTGCCGTTTACCCAGCTGAGGTTGTCTTTGAAGAAATAATTTTAAGACTGAGATGCCAGTACTGTACATTGATTAATTACTGACATGTATGT
AAGGCAATAATTTAATTAAGGGTGAATATTCTTTGTTTTTAGCATATAGGTTGCTGTAGATGAATGTTCTTAGCTGTCATGTTTAAAAATACTTCTGCTTCG
TTACCTCAAGTAAGTGATTTGTCAAGCAACTATGTCTGCTGACAGTTCAGAAAGTATCTGAT
>TUG1
>ENST00000646021.1|ENSG00000253352.9|OTTHUMG00000030444.6|OTTHUMT00000495120.1|RP3-430N8.2-012|TUG1|375|
AACAGTACCGGGGGCGGGCCGAGCGACGCAGCCGGGACGGTAGCTGCGGTGCGGACCGGAGGAGCCATCTTGTCTCGTCGCCGGGGAGTCAGGCCC
CTAAATCGAAGAAGCCCTGGCGCGCCCTCCCCCCCTCCCGGGTCTGGGCAGAGACAGATAATCTCACTTCCAGAGAAATGACTTGGAGAAAAAAAAG
TGTTGGTCTTTTTGCTCTTTTGTAATTAAATCCGGATGTACCTCAAAAGACTTAAGACTGTGGTGATAAGATGCTTTCCTCAGCAGAAAGGAGGGAAAA
AAAACAACTGGAACTCAAAGCTTGAAATTCTGTGGCAAAACATGAGATGTCCAGGATTGGAGGTTGAAAAGATTTCACTACAG
>ENST00000646496.1|ENSG00000253352.9|OTTHUMG00000030444.6|OTTHUMT00000494310.1|RP3-430N8.2-010|TUG1|4650|
GACGCAGCCGGGACGGTAGCTGCGGTGCGGACCGGAGGAGCCATCTTGTCTCGTCGCCGGGGAGTCAGGCCCCTAAATCGAAGAAGCCCTGGCGCG
CCCTCCCCCCCTCCCGGGTCTGATAGCAGACTCCTTGAAAGCAGGGTCCTTGTTTAGTGCATCTTTGCCCACATACACCACAACATATCAAGATGCATTT
ATTAGGAAGGAGGAGTTTAGAGAGCAGGCTATCAGAATAACCACTCATCCTGTGCCTCCTGATTGCTGAGTGTTCACCTGGACCTTCTGACTACCTTCC
CTGTGCTATTCCATCAGCCTACAGACCTGGTACCTGGATTTTTGCCCGAGATGATTCCTACCACCTTACTACTGACGAAGACACCCATTCCAGTGGACCA
CTGTGACCCAGGAGGCATTCAGCCATCATGATGTGGCCTTTACCTCCACTCCTGTCTTGTTCTACCCAGATTCAGCACAGCCCTTTATAGTGAAGTCAGA
GTCCTCAAGCCAAATAGCTAAAGCTGTTTTATCACAACAAAGGCCTAGTTTGTTCCATGAGTGTGCATTTCATTTCTTCAGTTAAAGCCTTCAGAGACA
CACAATAAATTTGGACCAGGGGATTTTTTAGTTATTAATGCTCTCTGAAGAAAGGCAACATCTTTTTGAGAGCAGCATTGGACCACACCCCACAATCTC
AAATGATTGAAATTCATGAACATCTAGGATCCCGTGAAGGTCACTGGACCCTGTTTTTTCTACTTCAAATCCTGTAGTAGCCTACTGAATGAGAAAACAT
ATTCTGACCCATTGGGATCAAATCAAAGGCACAGTGAACTCCTCATAGCATCTTCTTTGGAATTACTCAGGAACCAGAACTTTTTACACAAATGTAAGA
AATTCTACCAAGGAGTCCCCTTACCTAACAGCATCTCACAAGGCTGCACCAGATTCCAGAAAAGGCTTCTCTTGATACATCAAGCATTTTGTGACCGAC
TTATTCTTAGATCATTGGTTTTCCAAAGGCTTTGTGGCCATGAAGCCCTTTGAGTGAAAACTGTGCAGAAGCCCAGAGTAAAAGTGAAGCTGCTCTGG
ATGAAGTAGTGAAGCAAGAGTAGGGGCCTGAATCCTGCTACAACTATCTTCCTTTACCACCGTGGTGACACCTAAGGGGACTTCCTTACAACACCTTG
AACTCTTCCGAACACAGTTTGAAAACCACTGCCCCAGACAGCAATATGTTTGACCTGAATGGCATTCCAATCTTTTCTGTACCTCCACTCAGCACAGTT
CATGTTCAGTAGATGCTGAACATTCTTAGAAATACTGTGTGTGAACTTAGAAAAGTGCAAGAAGACAGGCATGTCTTTGACCCCAGGAATGATCATTTG

CTGAAGATGGTGTCAAGTGAACCTAGATTAACAGCCCTCCACTCCAGATGGATATCCAGTGATTCCTAGAATGGGATATAGCCAGAGAACAATTCTATG
CACCCTACACTGACAGACTCCCTTAAGCAACACCAGATGCTCTACTGGTACTTGAAGTACATGACTTTGAAGTCTTGACCCTCCATGAATACCTGAATT
ATCAGCAAGCGGGTTTTGAAGCTGGTGCCTCATTGAGGCCATATTAGAGCAACTTGTACATTTGACCTCTTGTTATCAGCCATGGTACTCTACTTCGTGT
GCAAGAGATAACTATGAAAGCCAAATTCAAATACTGGCAACATTTCCTAAAGGGGCTCAATATCTATCATTCGTCTTCTTTTCCAAACTACACATCACTG
TATGACTCAACCAGTAGCAGTTATATTGCCCCTTGGTTTTTATTCAGTTTAACTACTGTTTCCAAGATAAATGAGCTAATAAGCTTTAAAAAAAAAAAAA
AAAAAGGCTGAATTCTTTTTTCTTCATCACTGGCATATCTGCCTATTCTCCAGAATTATTATGACTATTCAGCTCACTTTAACAGTTGAACTTCAAGCGAC
AATCTTTGAACACCCCTTCTCATGTGATTTAAAATGAAACCATTTGGAAAAGTTTCTTCTAGCCAGTAATAGATTTTTTTTTAATTGCTCTGCCTTGTGC
CGAGAGATGTTCTTTTAAGATGAATCTTTTGATGTCTGATACCACCAAATATAGGTGGTAGGGAGAGTTGGAGGCTGGCCCTTTGAGCAGGCCATTAGC
TTACTTGCTGGGCATTTCCGATAGCTTATTGCCTACCTTTTTGCTGGAAACAAACTGATTTGAAAAACAAAATCTATGAAGACTGCAGCTAAGGATTTTA
TCGGTAGACTTAAGAGCTTTTGTCCTTGTGGATATTTTAGTGGAACCACATCAGTCTCAATACTGTCATTTTACACTGACTCAGAGCAGCTGACTTCATT
CCTTGCCATGATATATATTTAAGGCAGGCATTGTAACAGACATAAAGACAACTTATCTGTTTCAGCAGGAAGGATTCAGTTTATGAACTCTCAGACCAGA
TCATGTTGAACAAGGAGACTTTGATGTGTGTCATGAGAAAACTCATTCTTTACTTCCCAGTCAATTTAAAGGCCAGCTATCCTGAGCTACTCGAATGAAT
GCACTGGTTAAACATTGGAAATAGTTTGTTTATATCCTTGTCTCTCTCTAGGCCAATTGTGATTACATGACTCGACTCTACATCTCGTCAAACAAGGCCTA
GGTCTGGTTGCTGTAGACTGCTCGCCCTCAACAAATAAAATCTGGTTGACTAGCCTCCTTGTATATACAACTATTATTTGTTAAGAAGAAATTATCGTCAA
TTTTCTACTACCTTCCAATTGTCAGCTCTTTTTTTCCTCTCTGGTTTTTCCTATACTTTACAGAAAAAGACATTGATCTATACTGCCATTCCCTCTAATCCT
GCCATACTCAGTCAAAAGGAATGACTTAAGATGAAGATGATCATCTGCTCGAGTCTAAAATATACATTGTATATAAGAATTGGTGATTAGAAAAGCAAAA
AACCTAAAACTTAAATCTAGGAGTCTGTATACTGTCTCCATGTCTCCATGCCTCAGATCTCATCTAAATCTTTGAACAGCACCATTCAACCAATCTGAGG
CCTTGACTTGCTTGTAAGATGATTCTCAGAGATCGGCTGAGTTAAAAAAGATGACGACTTGATTACCAAAGAAAGTAGGGCCAACTTTGACAAATCTG
GCTCTGCTGACCCTGTCACTCCCAGATGTAGCATAGACTCCTAAACAGAACCTCAAGTCTGATTGAGGATAAGGCCTTCTCCTGAGCTGAAAGTTCTTT
GGCAGATGAGCAAGAAACTGAAAGCTGATGTACCTGACTGGCTCTGTAAGATCAGAAAACTGTATCCAGAATAAGCCCTATGGATTAACCCCTGAGTA
CCCAGAGTAAAAACTAATTTACAGAACTTCCTTATTGATCTGCTGGTTCTTCCAGATCATATTCTGGCTATTGGTATGGCTGGCCTTTCTGAAGGTACCCT
GCTTGTCTATTTTCCTGACTCAGCTCTTGCCTGCCTTTTTCACATGTTGCTGCAATTAGACTCACCGTGAGGACTACAGTCAATTTCAGTCTATCTTGTGC
CCAATACAACAAGGATTTTTAATAGTAACAACCCACACCTCACCCACTAGGACTCAATGTTCACAACAGGAAGGACCATTGCTGCATACTCCTTGACCA
GCAACTTTTTTGAAGATATTTTTAAGTGCAGAGTAGGCCTCTATTCCTGTATGTAATTGTTCATTTTCAGCACCTGGAACCTCATCTATCGGGTCTGGAAG
GAATACAGCAGTTCGAAAGCCGCGTCCATTTCTCTCCTTCAGTAGTGCAGAAATGAGTCCGATTCACCAGTACACACAGAACTGTACCAGTTCAACCT
AGCAAAAGAAGAAAAGTTTCCACTGTACTTAAAATTTACAGCTGACTCAAATTGCCTCACAGAATTATTTGATGTAGAAGGCTAGTTGTCTTACTTCAG

ATCAGCAGGACAGTTGGGCTCTCAGACTCATGACCACTGAGTTTGCTTGTGTTGAAACTGTGGTTTCATCCAACATATGCTATTGGACATGATTATTATT
CCATTCAAATGGATTACAGACTTCTTGAGGACAGGACAAACTTATCTCTCATGGTGTTTTTTTAGAATACTTTTATAACCAAGGAAGAAACCATGCCAGC
TGTTACCATTCAACTTCTTAAGCAGAGATTAAGCTTTTTCATATCTGTTCTTATCCTGGACATCAGTAGTTTTTAATTGCCCAGCATCCGTTCCATCTTGTA
ACAACTCCCTGATGTTTCTTAAAACCACCTCTTCCTATTTTCAGTCTGTGGTTTGGACAGTCTGACCCAACCTTGAGCTTTGTGGGTGAACATGTAATTC
AGACCTCATCAATCAGCAAATCCATCTGAACTGTGGAGGAGAAGCTCTCTTTACTGAGGGTGCTTTAGCTTTGTAGGATGAAAACCTCAAACTAACAG
GGCCTACCATGTAGAGAATGAAGCCAGTGCAGGGGAAAGCAGAGCCAAAATATGGAGAGACTTGAATCCTGATGACAGCGTTTGTGCCCCTGGATCC
AACCGTGCCTGAAGCTAGAATATCCCCTGGACTTTTCAGTTATGTGAACCAATAAATACCCTTTTTTGCTT
>ENST00000644773.1|ENSG00000253352.9|OTTHUMG00000030444.6|OTTHUMT00000431951.3|RP3-430N8.2-006|TUG1|7469|
ACGCAGCCGGGACGGTAGCTGCGGTGCGGACCGGAGGAGCCATCTTGTCTCGTCGCCGGGGAGTCAGGCCCCTAAATCGAAGAAGCCCTGGCGCGC
CCTCCCCCCCTCCCGGGTCTGGTAGGGCGAAGGAACGGGCGTGCGGTCGATCGAGCGATCGGTTGGCGGCTCTTTCTCCTGCTCTGGCATCCAGCTCT
TGGGGCGCAGGCCCGGCCGCCGCGGCGCGCGCCCGGTGGCCGTTGGCGCTCGCGCCGCGTCTTTCTTCTCGTACGCAGAACTCGGGCGGCGGCCTAT
GCGTTTGCGATTCGACGAGGAGTCGTCCGGGTGGTCGGCGGCGGCGGGCAGCTGCTCCGCCCCGCTCCGGGGGAGGCGGCGGCGGCAGCGGCCGCG
GGATTTGGAGCGGCCGGGGAGGCGGGGGTGGCCGGGGCCGGCTTGGAGGCCTGGCGCCACCCTTCGGGGCCTGCAAGGACCCAGTTGGGGGGGCA
GGAGGGGGCCGGAGGATGGTTGGTTGTGGGATTTCTACTTTGCCTTTTCCTCCTTATGCCGCCTTAGTGAGGGGCGGGAGCTCTGGCGGCAGCCCCGG
GGTGGGGAGACGAGCTCCGGAGTCGGAAGAGCTGGGTTTTCTTCCGGGCCTAGCCACCAGTTGGCGGAGTGACCTTAGGCGAGTCACTCTGTAATTT
GTCTGCGCCTCAGTTTCCTCCTCTGCCTATCAATGTGTGTGGGGTTGAAATCGCTTTGTAAACTATAAAGCGTGGGTGTACGTAAAGGATGGTTATTGTT
TATAATTTTTTTTGAGTTGTAAGAAAACTTAGCAGTTCCCCAATCCTTGGGTTTTGAACCTGGGAACCTTGGATTGGAGTTGGGGATCCCCAAACTTCCT
GAAATTGTGGGAATGTGCGGTTTGGGGGAATGATGGGAATTTGTGGGAATGTGCGTTTTAGGGGAATGATGATCCATCGCTAGCAAGTTTTCCAAGGG
GGCTGTGACCCAGAAGAGTTAAGAATCACAATTTCTTCATGCTACAGAGAGGAAACTGAGGCCTAGATGTCATTTGGGACCCTTCACAACCATTTTGA
AGCCCTGTTTGAGTCCCTGGGATATGTGAGCTGTTTCTATGCATAATGGATATTCGGGGTTAACAACAGTCCCCTGCTTGGCTTCTATTCTGAATCCTTTT
CTTTCACCATGGGGTGCCTGAAGGGTGGCTGATGCATATGGTACAATGGCACCCAGTGTAAAGCAGCTACAATTAGGAGTGGATGTGTTCTGTAGCATC
CTATTTAAATAAGCCTATTTTATCCTTTGGCCCGTCAACTCTGTTATCTGCTGCTTGTACTGGTGCCTGTACTTTTCTGACTCTCATTGACCATATTCCACG
ACCATGGTTGTCATCCATTACTTGATCCTACTTTACATGTCTAGGCTGTGTGGTTGGTGGTGAATAGGCTTCTTTTTACATGGTGCTGCCAGCCCAGCTAA
TTAATGGTGCACGTGGACTTTTAGCAAGCGGGCTCACTGGAAGAGACTGAACCTGGCATGGAATTCCTGAAGATGTTTGGGGTTTTTTTCTTTCTTAAT
CGAAAGTTAACATTGTCTGAAAAGTTTTGTTAGAACTACTGCGGAACCTCAAAATCAGTAGATTTGGAAGTGATTCAAAGCTAAACTTTTTCCTTGGCC
CTCCTTGTGTTCTAATTGCTTGCAAGTGTAATACTAGGATGTCCAAGATGCCAGTTTTTGCTTCTTTGTTAGTTGTCAGCTGCTTTTATCAAATTTCAGGC

CATTATCCAACAAACACTATAAAAATGTTTGAACAATTGGATTTCAAACATTTTCGTTTTGTGGAGTGGTGCTCACCAAGTGGTACAGCCCTAAGCAAG
TGAACACAAACACATTTAAGTGTATTTTGTCTGATTAGATGTTAGCCAGTTATGCTATTTCATTCAAATGTCTGAAAAAATCAATTGACTATTCCCTTTTC
CTAAAGGGCAGAGACAGATAATCTCACTTCCAGAGAAATGACTTGGAGAAAAAAAAGTGTTGGTCTTTTTGCTCTTTTGTAATTAAATCCGGATGTACC
TCAAAAGACTTAAGACTGTGGTGATAAGATGCTTTCCTCAGCAGAAAGGAGGGAAAAAAAACAACTGGAACTCAAAGCTTGAAATTCTGTGGCAAA
ACATGAGATGTCCAGGATTGGAGGTTGAAAAGATTTCACTACAGTGTTCTGCAATAGTTGGAGCAGATAACTTTCAGTGTAGCCACAGCCATGGACTCC
AGATTTCCAGATTTTCAAGACCTGGACCTGGAACCCGAAAGAGCTTGTCACGATGCGGCAGGAACACTGGAGGTAGATTTTTTTTATTTTTGAATTTT
GGGACTGTTGACCTTGCTGTGAGAAAAGAGACAACGACTGAGCAAGCACTACCACCAGCACTGTTACTGGGAATTAGAAGACCTGAGTTTCTGTCCA
GACCCTCAGTGCAAACTGAGGATGCTCCATCCAAAGTGAATTATGTCCTGTGCCTCCTGATTGCTGAGTGTTCACCTGGACCTTCTGACTACCTTCCCT
GTGCTATTCCATCAGCCTACAGACCTGGTACCTGGATTTTTGCCCGAGATGATTCCTACCACCTTACTACTGACGAAGACACCCATTCCAGTGGACCACT
GTGACCCAGGAGGCATTCAGCCATCATGATGTGGCCTTTACCTCCACTCCTGTCTTGTTCTACCCAGATTCAGCACAGCCCTTTATAGTGAAGTCAGAG
TCCTCAAGCCAAATAGCTAAAGCTGTTTTATCACAACAAAGGCCTAGTTTGTTCCATGAGTGTGCATTTCATTTCTTCAGTTAAAGCCTTCAGAGACAC
ACAATAAATTTGGACCAGGGGATTTTTTAGTTATTAATGCTCTCTGAAGAAAGGCAACATCTTTTTGAGAGCAGCATTGGACCACACCCCACAATCTCA
AATGATTGAAATTCATGAACATCTAGGATCCCGTGAAGGTCACTGGACCCTGTTTTTTCTACTTCAAATCCTGTAGTAGCCTACTGAATGAGAAAACATA
TTCTGACCCATTGGGATCAAATCAAAGGCACAGTGAACTCCTCATAGCATCTTCTTTGGAATTACTCAGGAACCAGAACTTTTTACACAAATGTAAGAA
ATTCTACCAAGGAGTCCCCTTACCTAACAGCATCTCACAAGGCTGCACCAGATTCCAGAAAAGGCTTCTCTTGATACATCAAGCATTTTGTGACCGACT
TATTCTTAGATCATTGGTTTTCCAAAGGCTTTGTGGCCATGAAGCCCTTTGAGTGAAAACTGTGCAGAAGCCCAGAGTAAAAGTGAAGCTGCTCTGGA
TGAAGTAGTGAAGCAAGAGTAGGGGCCTGAATCCTGCTACAACTATCTTCCTTTACCACCGTGGTGACACCTAAGGGGACTTCCTTACAACACCTTGA
ACTCTTCCGAACACAGTTTGAAAACCACTGCCCCAGACAGCAATATGTTTGACCTGAATGGCATTCCAATCTTTTCTGTACCTCCACTCAGCACAGTTC
ATGTTCAGTAGATGCTGAACATTCTTAGAAATACTGTGTGTGAACTTAGAAAAGTGCAAGAAGACAGGCATGTCTTTGACCCCAGGAATGATCATTTGC
TGAAGATGGTGTCAAGTGAACCTAGATTAACAGCCCTCCACTCCAGATGGATATCCAGTGATTCCTAGAATGGGATATAGCCAGAGAACAATTCTATGC
ACCCTACACTGACAGACTCCCTTAAGCAACACCAGATGCTCTACTGGTACTTGAAGTACATGACTTTGAAGTCTTGACCCTCCATGAATACCTGAATTAT
CAGCAAGCGGGTTTTGAAGCTGGTGCCTCATTGAGGCCATATTAGAGCAACTTGTACATTTGACCTCTTGTTATCAGCCATGGTACTCTACTTCGTGTGC
AAGAGATAACTATGAAAGCCAAATTCAAATACTGGCAACATTTCCTAAAGGGGCTCAATATCTATCATTCGTCTTCTTTTCCAAACTACACATCACTGTA
TGACTCAACCAGTAGCAGTTATATTGCCCCTTGGTTTTTATTCAGTTTAACTACTGTTTCCAAGATAAATGAGCTAATAAGCTTTAAAAAAAAAAAAAAA
AAAGGCTGAATTCTTTTTTCTTCATCACTGGCATATCTGCCTATTCTCCAGAATTATTATGACTATTCAGCTCACTTTAACAGTTGAACTTCAAGCGACAA
TCTTTGAACACCCCTTCTCATGTGATTTAAAATGAAACCATTTGGAAAAGTTTCTTCTAGCCAGTAATAGATTTTTTTTTAATTGCTCTGCCTTGTGCCG

AGAGATGTTCTTTTAAGATGAATCTTTTGATGTCTGATACCACCAAATATAGGTGGTAGGGAGAGTTGGAGGCTGGCCCTTTGAGCAGGCCATTAGCTT
ACTTGCTGGGCATTTCCGATAGCTTATTGCCTACCTTTTTGCTGGAAACAAACTGATTTGAAAAACAAAATCTATGAAGACTGCAGCTAAGGATTTTATC
GGTAGACTTAAGAGCTTTTGTCCTTGTGGATATTTTAGTGGAACCACATCAGTCTCAATACTGTCATTTTACACTGACTCAGAGCAGCTGACTTCATTCC
TTGCCATGATATATATTTAAGGCAGGCATTGTAACAGACATAAAGACAACTTATCTGTTTCAGCAGGAAGGATTCAGTTTATGAACTCTCAGACCAGATC
ATGTTGAACAAGGAGACTTTGATGTGTGTCATGAGAAAACTCATTCTTTACTTCCCAGTCAATTTAAAGGCCAGCTATCCTGAGCTACTCGAATGAATG
CACTGGTTAAACATTGGAAATAGTTTGTTTATATCCTTGTCTCTCTCTAGGCCAATTGTGATTACATGACTCGACTCTACATCTCGTCAAACAAGGCCTAG
GTCTGGTTGCTGTAGACTGCTCGCCCTCAACAAATAAAATCTGGTTGACTAGCCTCCTTGTATATACAACTATTATTTGTTAAGAAGAAATTATCGTCAAT
TTTCTACTACCTTCCAATTGTCAGCTCTTTTTTTCCTCTCTGGTTTTTCCTATACTTTACAGAAAAAGACATTGATCTATACTGCCATTCCCTCTAATCCTG
CCATACTCAGTCAAAAGGAATGACTTAAGATGAAGATGATCATCTGCTCGAGTCTAAAATATACATTGTATATAAGAATTGGTGATTAGAAAAGCAAAAA
ACCTAAAACTTAAATCTAGGAGTCTGTATACTGTCTCCATGTCTCCATGCCTCAGATCTCATCTAAATCTTTGAACAGCACCATTCAACCAATCTGAGGC
CTTGACTTGCTTGTAAGATGATTCTCAGAGATCGGCTGAGTTAAAAAAGATGACGACTTGATTACCAAAGAAAGTAGGGCCAACTTTGACAAATCTGG
CTCTGCTGACCCTGTCACTCCCAGATGTAGCATAGACTCCTAAACAGAACCTCAAGTCTGATTGAGGATAAGGCCTTCTCCTGAGCTGAAAGTTCTTTG
GCAGATGAGCAAGAAACTGAAAGCTGATGTACCTGACTGGCTCTGTAAGATCAGAAAACTGTATCCAGAATAAGCCCTATGGATTAACCCCTGAGTAC
CCAGAGTAAAAACTAATTTACAGAACTTCCTTATTGATCTGCTGGTTCTTCCAGATCATATTCTGGCTATTGGTATGGCTGGCCTTTCTGAAGGTACCCTG
CTTGTCTATTTTCCTGACTCAGCTCTTGCCTGCCTTTTTCACATGTTGCTGCAATTAGACTCACCGTGAGGACTACAGTCAATTTCAGTCTATCTTGTGCC
CAATACAACAAGGATTTTTAATAGTAACAACCCACACCTCACCCACTAGGACTCAATGTTCACAACAGGAAGGACCATTGCTGCATACTCCTTGACCAG
CAACTTTTTTGAAGATATTTTTAAGTGCAGAGTAGGCCTCTATTCCTGTATGTAATTGTTCATTTTCAGCACCTGGAACCTCATCTATCGGGTCTGGAAGG
AATACAGCAGTTCGAAAGCCGCGTCCATTTCTCTCCTTCAGTAGTGCAGAAATGAGTCCGATTCACCAGTACACACAGAACTGTACCAGTTCAACCTA
GCAAAAGAAGAAAAGTTTCCACTGTACTTAAAATTTACAGCTGACTCAAATTGCCTCACAGAATTATTTGATGTAGAAGGCTAGTTGTCTTACTTCAGA
TCAGCAGGACAGTTGGGCTCTCAGACTCATGACCACTGAGTTTGCTTGTGTTGAAACTGTGGTTTCATCCAACATATGCTATTGGACATGATTATTATTC
CATTCAAATGGATTACAGACTTCTTGAGGACAGGACAAACTTATCTCTCATGGTGTTTTTTTAGAATACTTTTATAACCAAGGAAGAAACCATGCCAGCT
GTTACCATTCAACTTCTTAAGCAGAGATTAAGCTTTTTCATATCTGTTCTTATCCTGGACATCAGTAGTTTTTAATTGCCCAGCATCCGTTCCATCTTGTAA
CAACTCCCTGATGTTTCTTAAAACCACCTCTTCCTATTTTCAGTCTGTGGTTTGGACAGTCTGACCCAACCTTGAGCTTTGTGGGTGAACATGTAATTCA
GACCTCATCAATCAGCAAATCCATCTGAACTGTGGAGGAGAAGCTCTCTTTACTGAGGGTGCTTTAGCTTTGTAGGATGAAAACCTCAAACTAACAGG
GCCTACCATGTAGAGAATGAAGCCAGTGCAGGGGAAAGCAGAGCCAAAATATGGAGAGACTTGAATCCTGATGACAGCGTTTGTGCCCCTGGATCCA
ACCGTGCCTGAAGCTAGAATATCCCCTGGACTTTTCAGTTATGTGAACCAATAAATACCCTTTTTTGCTTAAGTTACTTTGAGTTGGGTTTCTGTTACTTG

AAATTGAATCCACACTAATATATCTACCAACATTGAGACTTGACAGATCCAAGTATTTATTAAGCTAGAGGTCATGGTCACTGAAATTACTTTCCAAAGT
GGAAGACAAAATGAAACAGGAACTGAGGGAATATTTAAGATCCCACAGAAGCGTAAAAATGACATGGTAGAAAGTAATAGAAAACCTAAATGTCTGT
CATTAAAGGATAGGTTAAGGTGTGGTTCAGCCATATAGGAATATCTCGTATCTGTTAAAATGAATAAAGTACATTCATTGTGTATGGAAAAATGGCCATGA
TACATTAGGTGAAACAAGTTATTAATAGAAAAGTGTACAGTGTGAACTCATTTTAAAATGTGTGTGCTTATGTTTATAAATGCATAGAAAGGTCTATTCAC
AGCTTTCTTTGAACAGTGTAGATCACATGAAACTTTCAACTTTATACATTTCTGTATTAATATTTTACACTACCCACATTATTTTTAAACTTTATTTTAAATA
AAGAATTTTTAAAATTAAA
>ENST00000569384.2|ENSG00000253352.9|OTTHUMG00000030444.6|OTTHUMT00000431954.2|RP3-430N8.2-007|TUG1|4214|
GCAGCCGGGACGGTAGCTGCGGTGCGGACCGGAGGAGCCATCTTGTCTCGTCGCCGGGGAGTCAGGCCCCTAAATCGAAGAAGCCCTGGCGCGCCCT
CCCCCCCTCCCGGGTCTGTCCTGTGCCTCCTGATTGCTGAGTGTTCACCTGGACCTTCTGACTACCTTCCCTGTGCTATTCCATCAGCCTACAGACCTGG
TACCTGGATTTTTGCCCGAGATGATTCCTACCACCTTACTACTGACGAAGACACCCATTCCAGTGGACCACTGTGACCCAGGAGGCATTCAGCCATCAT
GATGTGGCCTTTACCTCCACTCCTGTCTTGTTCTACCCAGATTCAGCACAGCCCTTTATAGTGAAGTCAGAGTCCTCAAGCCAAATAGCTAAAGCTGTTT
TATCACAACAAAGGCCTAGTTTGTTCCATGAGTGTGCATTTCATTTCTTCAGTTAAAGCCTTCAGAGACACACAATAAATTTGGACCAGGGGATTTTTTA
GTTATTAATGCTCTCTGAAGAAAGGCAACATCTTTTTGAGAGCAGCATTGGACCACACCCCACAATCTCAAATGATTGAAATTCATGAACATCTAGGAT
CCCGTGAAGGTCACTGGACCCTGTTTTTTCTACTTCAAATCCTGTAGTAGCCTACTGAATGAGAAAACATATTCTGACCCATTGGGATCAAATCAAAGG
CACAGTGAACTCCTCATAGCATCTTCTTTGGAATTACTCAGGAACCAGAACTTTTTACACAAATGTAAGAAATTCTACCAAGGAGTCCCCTTACCTAAC
AGCATCTCACAAGGCTGCACCAGATTCCAGAAAAGGCTTCTCTTGATACATCAAGGCTTTGTGGCCATGAAGCCCTTTGAGTGAAAACTGTGCAGAAG
CCCAGAGTAAAAGTGAAGCTGCTCTGGATGAAGTAGTGAAGCAAGAGTAGGGGCCTGAATCCTGCTACAACTATCTTCCTTTACCACCGTGGTGACAC
CTAAGGGGACTTCCTTACAACACCTTGAACTCTTCCGAACACAGTTTGAAAACCACTGCCCCAGACAGCAATATGTTTGACCTGAATGGCATTCCAATC
TTTTCTGTACCTCCACTCAGCACAGTTCATGTTCAGTAGATGCTGAACATTCTTAGAAATACTGTGTGTGAACTTAGAAAAGTGCAAGAAGACAGGCAT
GTCTTTGACCCCAGGAATGATCATTTGCTGAAGATGGTGTCAAGTGAACCTAGATTAACAGCCCTCCACTCCAGATGGATATCCAGTGATTCCTAGAAT
GGGATATAGCCAGAGAACAATTCTATGCACCCTACACTGACAGACTCCCTTAAGCAACACCAGATGCTCTACTGGTACTTGAAGTACATGACTTTGAAG
TCTTGACCCTCCATGAATACCTGAATTATCAGCAAGCGGGTTTTGAAGCTGGTGCCTCATTGAGGCCATATTAGAGCAACTTGTACATTTGACCTCTTGT
TATCAGCCATGGTACTCTACTTCGTGTGCAAGAGATAACTATGAAAGCCAAATTCAAATACTGGCAACATTTCCTAAAGGGGCTCAATATCTATCATTCG
TCTTCTTTTCCAAACTACACATCACTGTATGACTCAACCAGTAGCAGTTATATTGCCCCTTGGTTTTTATTCAGTTTAACTACTGTTTCCAAGATAAATGA
GCTAATAAGCTTTAAAAAAAAAAAAAAAAAAAGGCTGAATTCTTTTTTCTTCATCACTGGCATATCTGCCTATTCTCCAGAATTATTATGACTATTCAGCTC
ACTTTAACAGTTGAACTTCAAGCGACAATCTTTGAACACCCCTTCTCATGTGATTTAAAATGAAACCATTTGGAAAAGTTTCTTCTAGCCAGTAATAGAT

TTTTTTTTTAATTGCTCTGCCTTGTGCCGAGAGATGTTCTTTTAAGATGAATCTTTTGATGTCTGATACCACCAAATATAGGTGGTAGGGAGAGTTGGAG
GCTGGCCCTTTGAGCAGGCCATTAGCTTACTTGCTGGGCATTTCCGATAGCTTATTGCCTACCTTTTTGCTGGAAACAAACTGATTTGAAAAACAAAATC
TATGAAGACTGCAGCTAAGGATTTTATCGGTAGACTTAAGAGCTTTTGTCCTTGTGGATATTTTAGTGGAACCACATCAGTCTCAATACTGTCATTTTACA
CTGACTCAGAGCAGCTGACTTCATTCCTTGCCATGATATATATTTAAGGCAGGCATTGTAACAGACATAAAGACAACTTATCTGTTTCAGCAGGAAGGAT
TCAGTTTATGAACTCTCAGACCAGATCATGTTGAACAAGGAGACTTTGATGTGTGTCATGAGAAAACTCATTCTTTACTTCCCAGTCAATTTAAAGGCC
AGCTATCCTGAGCTACTCGAATGAATGCACTGGTTAAACATTGGAAATAGTTTGTTTATATCCTTGTCTCTCTCTAGGCCAATTGTGATTACATGACTCGA
CTCTACATCTCGTCAAACAAGGCCTAGGTCTGGTTGCTGTAGACTGCTCGCCCTCAACAAATAAAATCTGGTTGACTAGCCTCCTTGTATATACAACTAT
TATTTGTTAAGAAGAAATTATCGTCAATTTTCTACTACCTTCCAATTGTCAGCTCTTTTTTTCCTCTCTGGTTTTTCCTATACTTTACAGAAAAAGACATTG
ATCTATACTGCCATTCCCTCTAATCCTGCCATACTCAGTCAAAAGGAATGACTTAAGATGAAGATGATCATCTGCTCGAGTCTAAAATATACATTGTATATA
AGAATTGGTGATTAGAAAAGCAAAAAACCTAAAACTTAAATCTAGGAGTCTGTATACTGTCTCCATGTCTCCATGCCTCAGATCTCATCTAAATCTTTGA
ACAGCACCATTCAACCAATCTGAGGCCTTGACTTGCTTGTAAGATGATTCTCAGAGATCGGCTGAGTTAAAAAAGATGACGACTTGATTACCAAAGAA
AGTAGGGCCAACTTTGACAAATCTGGCTCTGCTGACCCTGTCACTCCCAGATGTAGCATAGACTCCTAAACAGAACCTCAAGTCTGATTGAGGATAAG
GCCTTCTCCTGAGCTGAAAGTTCTTTGGCAGATGAGCAAGAAACTGAAAGCTGATGTACCTGACTGGCTCTGTAAGATCAGAAAACTGTATCCAGAAT
AAGCCCTATGGATTAACCCCTGAGTACCCAGAGTAAAAACTAATTTACAGAACTTCCTTATTGATCTGCTGGTTCTTCCAGATCATATTCTGGCTATTGGT
ATGGCTGGCCTTTCTGAAGGTACCCTGCTTGTCTATTTTCCTGACTCAGCTCTTGCCTGCCTTTTTCACATGTTGCTGCAATTAGACTCACCGTGAGGAC
TACAGTCAATTTCAGTCTATCTTGTGCCCAATACAACAAGGATTTTTAATAGTAACAACCCACACCTCACCCACTAGGACTCAATGTTCACAACAGGAA
GGACCATTGCTGCATACTCCTTGACCAGCAACTTTTTTGAAGATATTTTTAAGTGCAGAGTAGGCCTCTATTCCTGTATGTAATTGTTCATTTTCAGCACC
TGGAACCTCATCTATCGGGTCTGGAAGGAATACAGCAGTTCGAAAGCCGCGTCCATTTCTCTCCTTCAGTAGTGCAGAAATGAGTCCGATTCACCAGTA
CACACAGAACTGTACCAGTTCAACCTAGCAAAAGAAGAAAAGTTTCCACTGTACTTAAAATTTACAGCTGACTCAAATTGCCTCACAGAATTATTTGAT
GTAGAAGGCTAGTTGTCTTACTTCAGATCAGCAGGACAGTTGGGCTCTCAGACTCATGACCACTGAGTTTGCTTGTGTTGAAACTGTGGTTTCATCCAA
CATATGCTATTGGACATGATTATTATTCCATTCAAATGGATTACAGACTTCTTGAGGACAGGACAAACTTATCTCTCATGGTGTTTTTTTAGAATACTTTTA
TAACCAAGGAAGAAACCATGCCAGCTGTTACCATTCAACTTCTTAAGCAGAGATTAAGCTTTTTCATATCTGTTCTTATCCTGGACATCAGTAGTTTTTA
ATTGCCCAGCATCCGTTCCATCTTGTAACAACTCCCTGATGTTTCTTAAAACCACCTCTTCCTATTTTCAGTCTGTGGTTTGGACAGTCTGACCCAACCT
TGAGCTTTGTGGGTGAACATGTAATTCA
>ENST00000643920.1|ENSG00000253352.9|OTTHUMG00000030444.6|OTTHUMT00000495121.1|RP3-430N8.2-013|TUG1|4449|
GGGACGGTAGCTGCGGTGCGGACCGGAGGAGCCATCTTGTCTCGTCGCCGGGGAGTCAGGCCCCTAAATCGAAGAAGCCCTGGCGCGCCCTCCCCCC

CTCCCGGGTCTGCCTACAGACCTGGTACCTGGATTTTTGCCCGAGATGATTCCTACCACCTTACTACTGACGAAGACACCCATTCCAGTGGACCACTGT
GACCCAGGAGGCATTCAGCCATCATGATGTGGCCTTTACCTCCACTCCTGTCTTGTTCTACCCAGATTCAGCACAGCCCTTTATAGTGAAGTCAGAGTC
CTCAAGCCAAATAGCTAAAGCTGTTTTATCACAACAAAGGCCTAGTTTGTTCCATGAGTGTGCATTTCATTTCTTCAGTTAAAGCCTTCAGAGACACAC
AATAAATTTGGACCAGGGGATTTTTTAGTTATTAATGCTCTCTGAAGAAAGGCAACATCTTTTTGAGAGCAGCATTGGACCACACCCCACAATCTCAAA
TGATTGAAATTCATGAACATCTAGGATCCCGTGAAGGTCACTGGACCCTGTTTTTTCTACTTCAAATCCTGTAGTAGCCTACTGAATGAGAAAACATATT
CTGACCCATTGGGATCAAATCAAAGGCACAGTGAACTCCTCATAGCATCTTCTTTGGAATTACTCAGGAACCAGAACTTTTTACACAAATGTAAGAAAT
TCTACCAAGGAGTCCCCTTACCTAACAGCATCTCACAAGGCTGCACCAGATTCCAGAAAAGGCTTCTCTTGATACATCAAGGTAGAACCTCTATGCATT
TTGTGACCGACTTATTCTTAGATCATTGGTTTTCCAAAGGCTTTGTGGCCATGAAGCCCTTTGAGTGAAAACTGTGCAGAAGCCCAGAGTAAAAGTGA
AGCTGCTCTGGATGAAGTAGTGAAGCAAGAGTAGGGGCCTGAATCCTGCTACAACTATCTTCCTTTACCACCGTGGTGACACCTAAGGGGACTTCCTT
ACAACACCTTGAACTCTTCCGAACACAGTTTGAAAACCACTGCCCCAGACAGCAATATGTTTGACCTGAATGGCATTCCAATCTTTTCTGTACCTCCAC
TCAGCACAGTTCATGTTCAGTAGATGCTGAACATTCTTAGAAATACTGTGTGTGAACTTAGAAAAGTGCAAGAAGACAGGCATGTCTTTGACCCCAGG
AATGATCATTTGCTGAAGATGGTGTCAAGTGAACCTAGATTAACAGCCCTCCACTCCAGATGGATATCCAGTGATTCCTAGAATGGGATATAGCCAGAG
AACAATTCTATGCACCCTACACTGACAGACTCCCTTAAGCAACACCAGATGCTCTACTGGTACTTGAAGTACATGACTTTGAAGTCTTGACCCTCCATG
AATACCTGAATTATCAGCAAGCGGGTTTTGAAGCTGGTGCCTCATTGAGGCCATATTAGAGCAACTTGTACATTTGACCTCTTGTTATCAGCCATGGTAC
TCTACTTCGTGTGCAAGAGATAACTATGAAAGCCAAATTCAAATACTGGCAACATTTCCTAAAGGGGCTCAATATCTATCATTCGTCTTCTTTTCCAAAC
TACACATCACTGTATGACTCAACCAGTAGCAGTTATATTGCCCCTTGGTTTTTATTCAGTTTAACTACTGTTTCCAAGATAAATGAGCTAATAAGCTTTAA
AAAAAAAAAAAAAAAAGGCTGAATTCTTTTTTCTTCATCACTGGCATATCTGCCTATTCTCCAGAATTATTATGACTATTCAGCTCACTTTAACAGTTGA
ACTTCAAGCGACAATCTTTGAACACCCCTTCTCATGTGATTTAAAATGAAACCATTTGGAAAAGTTTCTTCTAGCCAGTAATAGATTTTTTTTTAATTGC
TCTGCCTTGTGCCGAGAGATGTTCTTTTAAGATGAATCTTTTGATGTCTGATACCACCAAATATAGGTGGTAGGGAGAGTTGGAGGCTGGCCCTTTGAG
CAGGCCATTAGCTTACTTGCTGGGCATTTCCGATAGCTTATTGCCTACCTTTTTGCTGGAAACAAACTGATTTGAAAAACAAAATCTATGAAGACTGCAG
CTAAGGATTTTATCGGTAGACTTAAGAGCTTTTGTCCTTGTGGATATTTTAGTGGAACCACATCAGTCTCAATACTGTCATTTTACACTGACTCAGAGCA
GCTGACTTCATTCCTTGCCATGATATATATTTAAGGCAGGCATTGTAACAGACATAAAGACAACTTATCTGTTTCAGCAGGAAGGATTCAGTTTATGAAC
TCTCAGACCAGATCATGTTGAACAAGGAGACTTTGATGTGTGTCATGAGAAAACTCATTCTTTACTTCCCAGTCAATTTAAAGGCCAGCTATCCTGAGC
TACTCGAATGAATGCACTGGTTAAACATTGGAAATAGTTTGTTTATATCCTTGTCTCTCTCTAGGCCAATTGTGATTACATGACTCGACTCTACATCTCGT
CAAACAAGGCCTAGGTCTGGTTGCTGTAGACTGCTCGCCCTCAACAAATAAAATCTGGTTGACTAGCCTCCTTGTATATACAACTATTATTTGTTAAGAA
GAAATTATCGTCAATTTTCTACTACCTTCCAATTGTCAGCTCTTTTTTTCCTCTCTGGTTTTTCCTATACTTTACAGAAAAAGACATTGATCTATACTGCCA

TTCCCTCTAATCCTGCCATACTCAGTCAAAAGGAATGACTTAAGATGAAGATGATCATCTGCTCGAGTCTAAAATATACATTGTATATAAGAATTGGTGAT
TAGAAAAGCAAAAAACCTAAAACTTAAATCTAGGAGTCTGTATACTGTCTCCATGTCTCCATGCCTCAGATCTCATCTAAATCTTTGAACAGCACCATTC
AACCAATCTGAGGCCTTGACTTGCTTGTAAGATGATTCTCAGAGATCGGCTGAGTTAAAAAAGATGACGACTTGATTACCAAAGAAAGTAGGGCCAAC
TTTGACAAATCTGGCTCTGCTGACCCTGTCACTCCCAGATGTAGCATAGACTCCTAAACAGAACCTCAAGTCTGATTGAGGATAAGGCCTTCTCCTGAG
CTGAAAGTTCTTTGGCAGATGAGCAAGAAACTGAAAGCTGATGTACCTGACTGGCTCTGTAAGATCAGAAAACTGTATCCAGAATAAGCCCTATGGATT
AACCCCTGAGTACCCAGAGTAAAAACTAATTTACAGAACTTCCTTATTGATCTGCTGGTTCTTCCAGATCATATTCTGGCTATTGGTATGGCTGGCCTTTC
TGAAGGTACCCTGCTTGTCTATTTTCCTGACTCAGCTCTTGCCTGCCTTTTTCACATGTTGCTGCAATTAGACTCACCGTGAGGACTACAGTCAATTTCA
GTCTATCTTGTGCCCAATACAACAAGGATTTTTAATAGTAACAACCCACACCTCACCCACTAGGACTCAATGTTCACAACAGGAAGGACCATTGCTGCA
TACTCCTTGACCAGCAACTTTTTTGAAGATATTTTTAAGTGCAGAGTAGGCCTCTATTCCTGTATGTAATTGTTCATTTTCAGCACCTGGAACCTCATCTA
TCGGGTCTGGAAGGAATACAGCAGTTCGAAAGCCGCGTCCATTTCTCTCCTTCAGTAGTGCAGAAATGAGTCCGATTCACCAGTACACACAGAACTGT
ACCAGTTCAACCTAGCAAAAGAAGAAAAGTTTCCACTGTACTTAAAATTTACAGCTGACTCAAATTGCCTCACAGAATTATTTGATGTAGAAGGCTAGT
TGTCTTACTTCAGATCAGCAGGACAGTTGGGCTCTCAGACTCATGACCACTGAGTTTGCTTGTGTTGAAACTGTGGTTTCATCCAACATATGCTATTGG
ACATGATTATTATTCCATTCAAATGGATTACAGACTTCTTGAGGACAGGACAAACTTATCTCTCATGGTGTTTTTTTAGAATACTTTTATAACCAAGGAAG
AAACCATGCCAGCTGTTACCATTCAACTTCTTAAGCAGAGATTAAGCTTTTTCATATCTGTTCTTATCCTGGACATCAGTAGTTTTTAATTGCCCAGCATC
CGTTCCATCTTGTAACAACTCCCTGATGTTTCTTAAAACCACCTCTTCCTATTTTCAGTCTGTGGTTTGGACAGTCTGACCCAACCTTGAGCTTTGTGGG
TGAACATGTAATTCAGACCTCATCAATCAGCAAATCCATCTGAACTGTGGAGGAGAAGCTCTCTTTACTGAGGGTGCTTTAGCTTTGTAGGATGAAAAC
CTCAAACTAACAGGGCCTACCATGTAGAGAATGAAGCCAGTGCAGGGGAAAGCAGAGCCAAAATATGGAGAGACTTGAATCCTGATGACAGCGTTTG
TGCCCCTGGATCCAACCGTGCCTGAAGCTAGAATATCCCCTGGACTTTTCAGTTATGTGAACCAATAAATAC
>ENST00000602971.2|ENSG00000253352.9|OTTHUMG00000030444.6|OTTHUMT00000495122.1|RP3-430N8.2-014|TUG1|1817|
GCGGACCGGAGGAGCCATCTTGTCTCGTCGCCGGGGAGTCAGGCCCCTAAATCGAAGAAGCCCTGGCGCGCCCTCCCCCCCTCCCGGGTCTGATAGC
AGACTCCTTGAAAGCAGGGTCCTTGTTTAGTGCATCTTTGCCCACATACACCACAACATATCAAGATGCATTTATTAGGAAGGAGGAGTTTAGAGAGCA
GGCTATCAGAATAACCACTCATCCTGTGCCTCCTGATTGCTGAGTGTTCACCTGGACCTTCTGACTACCTTCCCTGTGCTATTCCATCAGCCTACAGACC
TGGTACCTGGATTTTTGCCCGAGATGATTCCTACCACCTTACTACTGACGAAGACACCCATTCCAGTGGACCACTGTGACCCAGGAGGCATTCAGCCAT
CATGATGTGGCCTTTACCTCCACTCCTGTCTTGTTCTACCCAGATTCAGCACAGCCCTTTATAGTGAAGTCAGAGTCCTCAAGCCAAATAGCTAAAGCTG
TTTTATCACAACAAAGGCCTAGTTTGTTCCATGAGTGTGCATTTCATTTCTTCAGTTAAAGCCTTCAGAGACACACAATAAATTTGGACCAGGGGATTTT
TTAGTTATTAATGCTCTCTGAAGAAAGGCAACATCTTTTTGAGAGCAGCATTGGACCACACCCCACAATCTCAAATGATTGAAATTCATGAACATCTAG

GATCCCGTGAAGGTCACTGGACCCTGTTTTTTCTACTTCAAATCCTGTAGTAGCCTACTGAATGAGAAAACATATTCTGACCCATTGGGATCAAATCAAA
GGCACAGTGAACTCCTCATAGCATCTTCTTTGGAATTACTCAGGAACCAGAACTTTTTACACAAATGTAAGAAATTCTACCAAGGAGTCCCCTTACCTA
ACAGCATCTCACAAGGCTGCACCAGATTCCAGAAAAGGCTTCTCTTGATACATCAAGGTAGAACCTCTATGCATTTTGTGACCGACTTATTCTTAGATCA
TTGGTTTTCCAAAGGCTTTGTGGCCATGAAGCCCTTTGAGTGAAAACTGTGCAGAAGCCCAGAGTAAAAGTGAAGCTGCTCTGGATGAAGTAGTGAA
GCAAGAGTAGGGGCCTGAATCCTGCTACAACTATCTTCCTTTACCACCGTGGTGACACCTAAGGGGACTTCCTTACAACACCTTGAACTCTTCCGAACA
CAGTTTGAAAACCACTGCCCCAGACAGCAATATGTTTGACCTGAATGGCATTCCAATCTTTTCTGTACCTCCACTCAGCACAGTTCATGTTCAGTAGAT
GCTGAACATTCTTAGAAATACTGTGTGTGAACTTAGAAAAGTGCAAGAAGACAGGCATGTCTTTGACCCCAGGAATGATCATTTGCTGAAGATGGTGT
CAAGTGAACCTAGATTAACAGCCCTCCACTCCAGATGGATATCCAGTGATTCCTAGAATGGGATATAGCCAGAGAACAATTCTATGCACCCTACACTGA
CAGACTCCCTTAAGCAACACCAGATGCTCTACTGGTACTTGAAGTACATGACTTTGAAGTCTTGACCCTCCATGAATACCTGAATTATCAGCAAGCGGG
TTTTGAAGCTGGTGCCTCATTGAGGCCATATTAGAGCAACTTGTACATTTGACCTCTTGTTATCAGCCATGGTACTCTACTTCGTGTGCAAGAGATAACT
ATGAAAGCCAAATTCAAATACTGGCAACATTTCCTAAAGGGGCTCAATATCTATCATTCGTCTTCTTTTCCAAACTACACATCACTGTATGACTCAACCA
GTAGCAGTTATATTGCCCCTTGGTTTTTATTCA
>ENST00000519077.4|ENSG00000253352.9|OTTHUMG00000030444.6|OTTHUMT00000495123.1|RP3-430N8.2-011|TUG1|5017|
AGGCCCGGCCGCCGCGGCGCGCGCCCGGTGGCCGTTGGCGCTCGCGCCGCGTCTTTCTTCTCGGCAGAGACAGATAATCTCACTTCCAGAGAAATGA
CTTGGAGAAAAAAAAGTGTTGGTCTTTTTGCTCTTTTGTAATTAAATCCGGATGTACCTCAAAAGACTTAAGACTGTGGTGATAAGATGCTTTCCTCAG
CAGAAAGGAGGGAAAAAAAACAACTGGAACTCAAAGCTTGAAATTCTGTGGCAAAACATGAGATGTCCAGGATTGGAGGTTGAAAAGATTTCACTA
CAGTGTTCTGCAATAGTTGGAGCAGATAACTTTCAGTGTAGCCACAGCCATGGACTCCAGATTTCCAGATTTTCAAGACCTGGACCTGGAACCCGAAA
GAGCTTGTCACGATGCGGCAGGAACACTGGAGGTAGATTTTTTTTTATTTTTGAATTTTGGGACTGTTGACCTTGCTGTGAGAAAAGAGACAACGACT
GAGCAAGCACTACCACCAGCACTGTTACTGGGAATTAGAAGACCTGAGTTTCTGTCCAGACCCTCAGTGCAAACTGAGGATGCTCCATCCAAAGTGAA
TTATGTCCTGTGCCTCCTGATTGCTGAGTGTTCACCTGGACCTTCTGACTACCTTCCCTGTGCTATTCCATCAGCCTACAGACCTGGTACCTGGATTTTTG
CCCGAGATGATTCCTACCACCTTACTACTGACGAAGACACCCATTCCAGTGGACCACTGTGACCCAGGAGGCATTCAGCCATCATGATGTGGCCTTTAC
CTCCACTCCTGTCTTGTTCTACCCAGATTCAGCACAGCCCTTTATAGTGAAGTCAGAGTCCTCAAGCCAAATAGCTAAAGCTGTTTTATCACAACAAAG
GCCTAGTTTGTTCCATGAGTGTGCATTTCATTTCTTCAGTTAAAGCCTTCAGAGACACACAATAAATTTGGACCAGGGGATTTTTTAGTTATTAATGCTCT
CTGAAGAAAGGCAACATCTTTTTGAGAGCAGCATTGGACCACACCCCACAATCTCAAATGATTGAAATTCATGAACATCTAGGATCCCGTGAAGGTCA
CTGGACCCTGTTTTTTCTACTTCAAATCCTGTAGTAGCCTACTGAATGAGAAAACATATTCTGACCCATTGGGATCAAATCAAAGGCACAGTGAACTCC
TCATAGCATCTTCTTTGGAATTACTCAGGAACCAGAACTTTTTACACAAATGTAAGAAATTCTACCAAGGAGTCCCCTTACCTAACAGCATCTCACAAG

GCTGCACCAGATTCCAGAAAAGGCTTCTCTTGATACATCAAGGTAGAACCTCTATGCATTTTGTGACCGACTTATTCTTAGATCATTGGTTTTCCAAAGG
CTTTGTGGCCATGAAGCCCTTTGAGTGAAAACTGTGCAGAAGCCCAGAGTAAAAGTGAAGCTGCTCTGGATGAAGTAGTGAAGCAAGAGTAGGGGCC
TGAATCCTGCTACAACTATCTTCCTTTACCACCGTGGTGACACCTAAGGGGACTTCCTTACAACACCTTGAACTCTTCCGAACACAGTTTGAAAACCAC
TGCCCCAGACAGCAATATGTTTGACCTGAATGGCATTCCAATCTTTTCTGTACCTCCACTCAGCACAGTTCATGTTCAGTAGATGCTGAACATTCTTAGA
AATACTGTGTGTGAACTTAGAAAAGTGCAAGAAGACAGGCATGTCTTTGACCCCAGGAATGATCATTTGCTGAAGATGGTGTCAAGTGAACCTAGATT
AACAGCCCTCCACTCCAGATGGATATCCAGTGATTCCTAGAATGGGATATAGCCAGAGAACAATTCTATGCACCCTACACTGACAGACTCCCTTAAGCA
ACACCAGATGCTCTACTGGTACTTGAAGTACATGACTTTGAAGTCTTGACCCTCCATGAATACCTGAATTATCAGCAAGCGGGTTTTGAAGCTGGTGCC
TCATTGAGGCCATATTAGAGCAACTTGTACATTTGACCTCTTGTTATCAGCCATGGTACTCTACTTCGTGTGCAAGAGATAACTATGAAAGCCAAATTCA
AATACTGGCAACATTTCCTAAAGGGGCTCAATATCTATCATTCGTCTTCTTTTCCAAACTACACATCACTGTATGACTCAACCAGTAGCAGTTATATTGCC
CCTTGGTTTTTATTCAGTTTAACTACTGTTTCCAAGATAAATGAGCTAATAAGCTTTAAAAAAAAAAAAAAAAAAAGGCTGAATTCTTTTTTCTTCATCAC
TGGCATATCTGCCTATTCTCCAGAATTATTATGACTATTCAGCTCACTTTAACAGTTGAACTTCAAGCGACAATCTTTGAACACCCCTTCTCATGTGATTT
AAAATGAAACCATTTGGAAAAGTTTCTTCTAGCCAGTAATAGATTTTTTTTTTAATTGCTCTGCCTTGTGCCGAGAGATGTTCTTTTAAGATGAATCTTTT
GATGTCTGATACCACCAAATATAGGTGGTAGGGAGAGTTGGAGGCTGGCCCTTTGAGCAGGCCATTAGCTTACTTGCTGGGCATTTCCGATAGCTTATTG
CCTACCTTTTTGCTGGAAACAAACTGATTTGAAAAACAAAATCTATGAAGACTGCAGCTAAGGATTTTATCGGTAGACTTAAGAGCTTTTGTCCTTGTG
GATATTTTAGTGGAACCACATCAGTCTCAATACTGTCATTTTACACTGACTCAGAGCAGCTGACTTCATTCCTTGCCATGATATATATTTAAGGCAGGCAT
TGTAACAGACATAAAGACAACTTATCTGTTTCAGCAGGAAGGATTCAGTTTATGAACTCTCAGACCAGATCATGTTGAACAAGGAGACTTTGATGTGTG
TCATGAGAAAACTCATTCTTTACTTCCCAGTCAATTTAAAGGCCAGCTATCCTGAGCTACTCGAATGAATGCACTGGTTAAACATTGGAAATAGTTTGTT
TATATCCTTGTCTCTCTCTAGGCCAATTGTGATTACATGACTCGACTCTACATCTCGTCAAACAAGGCCTAGGTCTGGTTGCTGTAGACTGCTCGCCCTC
AACAAATAAAATCTGGTTGACTAGCCTCCTTGTATATACAACTATTATTTGTTAAGAAGAAATTATCGTCAATTTTCTACTACCTTCCAATTGTCAGCTCTT
TTTTTCCTCTCTGGTTTTTCCTATACTTTACAGAAAAAGACATTGATCTATACTGCCATTCCCTCTAATCCTGCCATACTCAGTCAAAAGGAATGACTTAA
GATGAAGATGATCATCTGCTCGAGTCTAAAATATACATTGTATATAAGAATTGGTGATTAGAAAAGCAAAAAACCTAAAACTTAAATCTAGGAGTCTGTA
TACTGTCTCCATGTCTCCATGCCTCAGATCTCATCTAAATCTTTGAACAGCACCATTCAACCAATCTGAGGCCTTGACTTGCTTGTAAGATGATTCTCAG
AGATCGGCTGAGTTAAAAAAGATGACGACTTGATTACCAAAGAAAGTAGGGCCAACTTTGACAAATCTGGCTCTGCTGACCCTGTCACTCCCAGATGT
AGCATAGACTCCTAAACAGAACCTCAAGTCTGATTGAGGATAAGGCCTTCTCCTGAGCTGAAAGTTCTTTGGCAGATGAGCAAGAAACTGAAAGCTGA
TGTACCTGACTGGCTCTGTAAGATCAGAAAACTGTATCCAGAATAAGCCCTATGGATTAACCCCTGAGTACCCAGAGTAAAAACTAATTTACAGAACTT
CCTTATTGATCTGCTGGTTCTTCCAGATCATATTCTGGCTATTGGTATGGCTGGCCTTTCTGAAGGTACCCTGCTTGTCTATTTTCCTGACTCAGCTCTTGC

CTGCCTTTTTCACATGTTGCTGCAATTAGACTCACCGTGAGGACTACAGTCAATTTCAGTCTATCTTGTGCCCAATACAACAAGGATTTTTAATAGTAAC
AACCCACACCTCACCCACTAGGACTCAATGTTCACAACAGGAAGGACCATTGCTGCATACTCCTTGACCAGCAACTTTTTTGAAGATATTTTTAAGTGC
AGAGTAGGCCTCTATTCCTGTATGTAATTGTTCATTTTCAGCACCTGGAACCTCATCTATCGGGTCTGGAAGGAATACAGCAGTTCGAAAGCCGCGTCCA
TTTCTCTCCTTCAGTAGTGCAGAAATGAGTCCGATTCACCAGTACACACAGAACTGTACCAGTTCAACCTAGCAAAAGAAGAAAAGTTTCCACTGTAC
TTAAAATTTACAGCTGACTCAAATTGCCTCACAGAATTATTTGATGTAGAAGGCTAGTTGTCTTACTTCAGATCAGCAGGACAGTTGGGCTCTCAGACT
CATGACCACTGAGTTTGCTTGTGTTGAAACTGTGGTTTCATCCAACATATGCTATTGGACATGATTATTATTCCATTCAAATGGATTACAGACTTCTTGAG
GACAGGACAAACTTATCTCTCATGGTGTTTTTTTAGAATACTTTTATAACCAAGGAAGAAACCATGCCAGCTGTTACCATTCAACTTCTTAAGCAGAGAT
TAAGCTTTTTCATATCTGTTCTTATCCTGGACATCAGTAGTTTTTAATTGCCCAGCATCCGTTCCATCTTGTAACAACTCCCTGATGTTTCTTAAAACCACC
TCTTCCTATTTTCAGTCTGTGGTTTGGACAGTCTGACCCAACCTTGAGCTTTGTGGGTGAACATGTAATTCAGACCTCATCAATCAGCAAATCCATCTGA
ACTGTGGAGGAGAAGCTCTCTTTACTGAGGGTGCTTTAGCTTTGTAGGATGAAAACCTCAAACTAACAGGGCCTACCATGTAGAGAATGAAGCCAGTG
CAGGGGAAAGCAGAGCCAAAATATGGAGAGACTTGAATCCTGATGACAGCGTTTGTGCCCCTGGATCCAACCGTGCCTGAAGCTAGAATATCCCCTGG
ACTTTTCAGTTATGTGAACCAATAAATACCCTTTTTTGCTTAAGTT
>ENST00000643280.1|ENSG00000253352.9|OTTHUMG00000030444.6|OTTHUMT00000075332.4|RP3-430N8.2-001|TUG1|3419|
GGGGAGACGAGCTCCGGAGTCGGAAGAGCTGGGTTTTCTTCCGGGCCTAGCCACCAGTTGGCGGAGTGACCTTAGGCGAGTCACTCTGTAATTTGTC
TGCGCCTCAGTTTCCTCCTCTGCCTATCAATGTGTGTGGGGTTGAAATCGCTTTGTAAACTATAAAGCGTGGGTGTACGTAAAGGATGGTTATTGTTTAT
AATTTTTTTTGAGTTGTAAGAAAACTTAGCAGTTCCCCAATCCTTGGGTTTTGAACCTGGGAACCTTGGATTGGAGTTGGGGATCCCCAAACTTCCTGA
AATTGTGGGAATGTGCGGTTTGGGGGAATGATGGGAATTTGTGGGAATGTGCGTTTTAGGGGAATGATGATCCATCGCTAGCAAGTTTTCCAAGGGGGC
TGTGACCCAGAAGAGTTAAGAATCACAATTTCTTCATGCTACAGAGAGGAAACTGAGGCCTAGATGTCATTTGGGACCCTTCACAACCATTTTGAAGC
CCTGTTTGAGTCCCTGGGATATGTGAGCTGTTTCTATGCATAATGGATATTCGGGGTTAACAACAGTCCCCTGCTTGGCTTCTATTCTGAATCCTTTTCTT
TCACCATGGGGTGCCTGAAGGGTGGCTGATGCATATGGTACAATGGCACCCAGTGTAAAGCAGCTACAATTAGGAGTGGATGTGTTCTGTAGCATCCTA
TTTAAATAAGCCTATTTTATCCTTTGGCCCGTCAACTCTGTTATCTGCTGCTTGTACTGGTGCCTGTACTTTTCTGACTCTCATTGACCATATTCCACGACC
ATGGTTGTCATCCATTACTTGATCCTACTTTACATGTCTAGGCTGTGTGGTTGGTGGTGAATAGGCTTCTTTTTACATGGTGCTGCCAGCCCAGCTAATTA
ATGGTGCACGTGGACTTTTAGCAAGCGGGCTCACTGGAAGAGACTGAACCTGGCATGGAATTCCTGAAGATGTTTGGGGTTTTTTTCTTTCTTAATCGA
AAGTTAACATTGTCTGAAAAGTTTTGTTAGAACTACTGCGGAACCTCAAAATCAGTAGATTTGGAAGTGATTCAAAGCTAAACTTTTTCCTTGGCCCTC
CTTGTGTTCTAATTGCTTGCAAGTGTAATACTAGGATGTCCAAGATGCCAGTTTTTGCTTCTTTGTTAGTTGTCAGCTGCTTTTATCAAATTTCAGGCCAT
TATCCAACAAACACTATAAAAATGTTTGAACAATTGGATTTCAAACATTTTCGTTTTGTGGAGTGGTGCTCACCAAGTGGTACAGCCCTAAGCAAGTGA

ACACAAACACATTTAAGTGTATTTTGTCTGATTAGATGTTAGCCAGTTATGCTATTTCATTCAAATGTCTGAAAAAATCAATTGACTATTCCCTTTTCCTAA
AGGGCAGAGACAGATAATCTCACTTCCAGAGAAATGACTTGGAGAAAAAAAAGTGTTGGTCTTTTTGCTCTTTTGTAATTAAATCCGGATGTACCTCAA
AAGACTTAAGACTGTGGTGATAAGATGCTTTCCTCAGCAGAAAGGAGGGAAAAAAAACAACTGGAACTCAAAGCTTGAAATTCTGTGGCAAAACATG
AGATGTCCAGGATTGGAGGTTGAAAAGATTTCACTACAGTGTTCTGCAATAGTTGGAGCAGATAACTTTCAGTGTAGCCACAGCCATGGACTCCAGATT
TCCAGATTTTCAAGACCTGGACCTGGAACCCGAAAGAGCTTGTCACGATGCGGCAGGAACACTGGAGGTAGATTTTTTTTATTTTTGAATTTTGGGAC
TGTTGACCTTGCTGTGAGAAAAGAGACAACGACTGAGCAAGCACTACCACCAGCACTGTTACTGGGAATTAGAAGACCTGAGTTTCTGTCCAGACCC
TCAGTGCAAACTGAGGATGCTCCATCCAAAGTGAATTATGCCTACAGACCTGGTACCTGGATTTTTGCCCGAGATGATTCCTACCACCTTACTACTGACG
AAGACACCCATTCCAGTGGACCACTGTGACCCAGGAGGCATTCAGCCATCATGATGTGGCCTTTACCTCCACTCCTGTCTTGTTCTACCCAGATTCAGC
ACAGCCCTTTATAGTGAAGTCAGAGTCCTCAAGCCAAATAGCTAAAGCTGTTTTATCACAACAAAGGCCTAGTTTGTTCCATGAGTGTGCATTTCATTTC
TTCAGTTAAAGCCTTCAGAGACACACAATAAATTTGGACCAGGGGATTTTTTAGTTATTAATGCTCTCTGAAGAAAGGCAACATCTTTTTGAGAGCAGC
ATTGGACCACACCCCACAATCTCAAATGATTGAAATTCATGAACATCTAGGATCCCGTGAAGGTCACTGGACCCTGTTTTTTCTACTTCAAATCCTGTAG
TAGCCTACTGAATGAGAAAACATATTCTGACCCATTGGGATCAAATCAAAGGCACAGTGAACTCCTCATAGCATCTTCTTTGGAATTACTCAGGAACCA
GAACTTTTTACACAAATGTAAGAAATTCTACCAAGGAGTCCCCTTACCTAACAGCATCTCACAAGGCTGCACCAGATTCCAGAAAAGGCTTCTCTTGAT
ACATCAAGCATTTTGTGACCGACTTATTCTTAGATCATTGGTTTTCCAAAGGCTTTGTGGCCATGAAGCCCTTTGAGTGAAAACTGTGCAGAAGCCCAG
AGTAAAAGTGAAGCTGCTCTGGATGAAGTAGTGAAGCAAGAGTAGGGGCCTGAATCCTGCTACAACTATCTTCCTTTACCACCGTGGTGACACCTAAG
GGGACTTCCTTACAACACCTTGAACTCTTCCGAACACAGTTTGAAAACCACTGCCCCAGACAGCAATATGTTTGACCTGAATGGCATTCCAATCTTTTC
TGTACCTCCACTCAGCACAGTTCATGTTCAGTAGATGCTGAACATTCTTAGAAATACTGTGTGTGAACTTAGAAAAGTGCAAGAAGACAGGCATGTCTT
TGACCCCAGGAATGATCATTTGCTGAAGATGGTGTCAAGTGAACCTAGATTAACAGCCCTCCACTCCAGATGGATATCCAGTGATTCCTAGAATGGGAT
ATAGCCAGAGAACAATTCTATGCACCCTACACTGACAGACTCCCTTAAGCAACACCAGATGCTCTACTGGTACTTGAAGTACATGACTTTGAAGTCTTG
ACCCTCCATGAATACCTGAATTATCAGCAAGCGGGTTTTGAAGCTGGTGCCTCATTGAGGCCATATTAGAGCAACTTGTACATTTGACCTCTTGTTATCA
GCCATGGTACTCTACTTCGTGTGCAAGAGATAACTATGAAAGCCAAATTCAAATACTGGCAACATTTCCTAAAGGGGCTCAATATCTATCATTCGTCTTC
TTTTCCAAACTACACATCACTGTATGACTCAACCAGTAGCAGT
>ENST00000643877.1|ENSG00000253352.9|OTTHUMG00000030444.6|OTTHUMT00000495124.1|RP3-430N8.2-016|TUG1|3028|
GTCACTCTGTAATTTGTCTGCGCCTCAGTTTCCTCCTCTGCCTATCAATGTGTGTGGGGTTGAAATCGCTTTGTAAACTATAAAGCGTGGGTGTACGTAA
AGGATGGTTATTGTTTATAATTTTTTTTGAGTTGTAAGAAAACTTAGCAGTTCCCCAATCCTTGGGTTTTGAACCTGGGAACCTTGGATTGGAGTTGGGG
ATCCCCAAACTTCCTGAAATTGTGGGAATGTGCGGTTTGGGGGAATGATGGGAATTTGTGGGAATGTGCGTTTTAGGGGAATGATGATCCATCGCTAGC

AAGTTTTCCAAGGGGGCTGTGACCCAGAAGAGTTAAGAATCACAATTTCTTCATGCTACAGAGAGGAAACTGAGGCCTAGATGTCATTTGGGACCCTT
CACAACCATTTTGAAGCCCTGTTTGAGTCCCTGGGATATGTGAGCTGTTTCTATGCATAATGGATATTCGGGGTTAACAACAGTCCCCTGCTTGGCTTCT
ATTCTGAATCCTTTTCTTTCACCATGGGGTGCCTGAAGGGTGGCTGATGCATATGGTACAATGGCACCCAGTGTAAAGCAGCTACAATTAGGAGTGGAT
GTGTTCTGTAGCATCCTATTTAAATAAGCCTATTTTATCCTTTGGCCCGTCAACTCTGTTATCTGCTGCTTGTACTGGTGCCTGTACTTTTCTGACTCTCAT
TGACCATATTCCACGACCATGGTTGTCATCCATTACTTGATCCTACTTTACATGTCTAGGCTGTGTGGTTGGTGGTGAATAGGCTTCTTTTTACATGGTGC
TGCCAGCCCAGCTAATTAATGGTGCACGTGGACTTTTAGCAAGCGGGCTCACTGGAAGAGACTGAACCTGGCATGGAATTCCTGAAGATGTTTGGGGT
TTTTTTCTTTCTTAATCGAAAGTTAACATTGTCTGAAAAGTTTTGTTAGAACTACTGCGGAACCTCAAAATCAGTAGATTTGGAAGTGATTCAAAGCTAA
ACTTTTTCCTTGGCCCTCCTTGTGTTCTAATTGCTTGCAAGTGTAATACTAGGATGTCCAAGATGCCAGTTTTTGCTTCTTTGTTAGTTGTCAGCTGCTTT
TATCAAATTTCAGGCCATTATCCAACAAACACTATAAAAATGTTTGAACAATTGGATTTCAAACATTTTCGTTTTGTGGAGTGGTGCTCACCAAGTGGTA
CAGCCCTAAGCAAGTGAACACAAACACATTTAAGTGTATTTTGTCTGATTAGATGTTAGCCAGTTATGCTATTTCATTCAAATGTCTGAAAAAATCAATT
GACTATTCCCTTTTCCTAAAGGGCAGAGACAGATAATCTCACTTCCAGAGAAATGACTTGGAGAAAAAAAAGTGTTGGTCTTTTTGCTCTTTTGTAATT
AAATCCGGATGTACCTCAAAAGACTTAAGACTGTGGTGATAAGATGCTTTCCTCAGCAGAAAGGAGGGAAAAAAAACAACTGGAACTCAAAGCTTGA
AATTCTGTGGCAAAACATGAGATGTCCAGGATTGGAGGTTGAAAAGATTTCACTACAGTGTTCTGCAATAGTTGGAGCAGATAACTTTCAGTGTAGCCA
CAGCCATGGACTCCAGATTTCCAGATTTTCAAGACCTGGACCTGGAACCCGAAAGAGCTTGTCACGATGCGGCAGGAACACTGGAGGTAGATTTTTTT
TTATTTTTGAATTTTGGGACTGTTGACCTTGCTGTGAGAAAAGAGACAACGACTGAGCAAGCACTACCACCAGCACTGTTACTGGGAATTAGAAGACC
TGAGTTTCTGTCCAGACCCTCAGTGCAAACTGAGGATGCTCCATCCAAAGTGAATTATGCCTACAGACCTGGTACCTGGATTTTTGCCCGAGATGATTC
CTACCACCTTACTACTGACGAAGACACCCATTCCAGTGGACCACTGTGACCCAGGAGGCATTCAGCCATCATGATGTGGCCTTTACCTCCACTCCTGTC
TTGTTCTACCCAGATTCAGCACAGCCCTTTATAGTGAAGTCAGAGTCCTCAAGCCAAATAGCTAAAGCTGTTTTATCACAACAAAGGCCTAGTTTGTTC
CATGAGTGTGCATTTCATTTCTTCAGTTAAAGCCTTCAGAGACACACAATAAATTTGGACCAGGGGATTTTTTAGTTATTAATGCTCTCTGAAGAAAGGC
AACATCTTTTTGAGAGCAGCATTGGACCACACCCCACAATCTCAAATGATTGAAATTCATGAACATCTAGGATCCCGTGAAGGTCACTGGACCCTGTTT
TTTCTACTTCAAATCCTGTAGTAGCCTACTGAATGAGAAAACATATTCTGACCCATTGGGATCAAATCAAAGGCACAGTGAACTCCTCATAGCATCTTCT
TTGGAATTACTCAGGAACCAGAACTTTTTACACAAATGTAAGAAATTCTACCAAGGAGTCCCCTTACCTAACAGCATCTCACAAGGCTGCACCAGATTC
CAGAAAAGGCTTCTCTTGATACATCAAGGTAGAACCTCTATGGTAAATCCCTCTGTTTATAATGCACTTTACAGTTTCAAAGCACTTGTACGTACATTTAT
CTCAATTATCAAAATAACAGTGAATTATAATCCCTTTTTGGAAATGAGGGAATGTAAATTTTAAAAGTTAAGCAATTATCCAAGGTGACACAGCTGTGAA
AGTGGTAGAGGCCAGGCTTTCTGACACCCAGTCCAGTACTCTCCTTGGAATTAGCCTCCATTTTACCCACAGTGTTAAAATGGCTGGGTATTGGGAACC
TCAGGAAATTTTCCAGCTTCCTGATCTGGTGCCCCTAGTCACATAGGATGTGTGATCACGTGATAGCTACATCTGGACCAGAATCCCATGGCAATATCCC

ACGGTTTAAGGGCATAATATCTCCGTGGTGGTAGATTGTCAAGTCAAAAACGGGACATGTTCTAACAGCAAAGCTACCCAATATGAAACACAAAGCTA
CTTTTAGATTTTTTAAGACTTACTGATATTCCTGGGCCCTAAGAGC
>ENST00000643071.1|ENSG00000253352.9|OTTHUMG00000030444.6|OTTHUMT00000495125.1|RP3-430N8.2-017|TUG1|6154|
GTTTCCTCCTCTGCCTATCAATGTGTGTGGGGTTGAAATCGCTTTGTAAACTATAAAGCGTGGGTGTACGTAAAGGATGGTTATTGTTTATAATTTTTTTT
GAGTTGTAAGAAAACTTAGCAGTTCCCCAATCCTTGGGTTTTGAACCTGGGAACCTTGGATTGGAGTTGGGGATCCCCAAACTTCCTGAAATTGTGGG
AATGTGCGGTTTGGGGGAATGATGGGAATTTGTGGGAATGTGCGTTTTAGGGGAATGATGATCCATCGCTAGCAAGTTTTCCAAGGGGGCTGTGACCC
AGAAGAGTTAAGAATCACAATTTCTTCATGCTACAGAGAGGAAACTGAGGCCTAGATGTCATTTGGGACCCTTCACAACCATTTTGAAGCCCTGTTTG
AGTCCCTGGGATATGTGAGCTGTTTCTATGCATAATGGATATTCGGGGTTAACAACAGTCCCCTGCTTGGCTTCTATTCTGAATCCTTTTCTTTCACCATG
GGGTGCCTGAAGGGTGGCTGATGCATATGGTACAATGGCACCCAGTGTAAAGCAGCTACAATTAGGAGTGGATGTGTTCTGTAGCATCCTATTTAAATA
AGCCTATTTTATCCTTTGGCCCGTCAACTCTGTTATCTGCTGCTTGTACTGGTGCCTGTACTTTTCTGACTCTCATTGACCATATTCCACGACCATGGTTGT
CATCCATTACTTGATCCTACTTTACATGTCTAGGCTGTGTGGTTGGTGGTGAATAGGCTTCTTTTTACATGGTGCTGCCAGCCCAGCTAATTAATGGTGCA
CGTGGACTTTTAGCAAGCGGGCTCACTGGAAGAGACTGAACCTGGCATGGAATTCCTGAAGATGTTTGGGGTTTTTTTCTTTCTTAATCGAAAGTTAAC
ATTGTCTGAAAAGTTTTGTTAGAACTACTGCGGAACCTCAAAATCAGTAGATTTGGAAGTGATTCAAAGCTAAACTTTTTCCTTGGCCCTCCTTGTGTT
CTAATTGCTTGCAAGTGTAATACTAGGATGTCCAAGATGCCAGTTTTTGCTTCTTTGTTAGTTGTCAGCTGCTTTTATCAAATTTCAGGCCATTATCCAAC
AAACACTATAAAAATGTTTGAACAATTGGATTTCAAACATTTTCGTTTTGTGGAGTGGTGCTCACCAAGTGGTACAGCCCTAAGCAAGTGAACACAAA
CACATTTAAGTGTATTTTGTCTGATTAGATGTTAGCCAGTTATGCTATTTCATTCAAATGTCTGAAAAAATCAATTGACTATTCCCTTTTCCTAAAGGGCA
GAGACAGATAATCTCACTTCCAGAGAAATGACTTGGAGAAAAAAAAGTGTTGGTCTTTTTGCTCTTTTGTAATTAAATCCGGATGTACCTCAAAAGACT
TAAGACTGTGGTGATAAGATGCTTTCCTCAGCAGAAAGGAGGGAAAAAAAACAACTGGAACTCAAAGCTTGAAATTCTGTGGCAAAACATGAGATGT
CCAGGATTGGAGGTTGAAAAGATTTCACTACAGTGTTCTGCAATAGTTGGAGCAGATAACTTTCAGTGTAGCCACAGCCATGGACTCCAGATTTCCAGA
TTTTCAAGACCTGGACCTGGAACCCGAAAGAGCTTGTCACGATGCGGCAGGAACACTGGAGGTAGATTTTTTTTATTTTTGAATTTTGGGACTGTTGA
CCTTGCTGTGAGAAAAGAGACAACGACTGAGCAAGCACTACCACCAGCACTGTTACTGGGAATTAGAAGACCTGAGTTTCTGTCCAGACCCTCAGTG
CAAACTGAGGATGCTCCATCCAAAGTGAATTATGCCTACAGACCTGGTACCTGGATTTTTGCCCGAGATGATTCCTACCACCTTACTACTGACGAAGAC
ACCCATTCCAGTGGACCACTGTGACCCAGGAGGCATTCAGCCATCATGATGTGGCCTTTACCTCCACTCCTGTCTTGTTCTACCCAGATTCAGCACAGC
CCTTTATAGTGAAGTCAGAGTCCTCAAGCCAAATAGCTAAAGCTGTTTTATCACAACAAAGGCCTAGTTTGTTCCATGAGTGTGCATTTCATTTCTTCAG
TTAAAGCCTTCAGAGACACACAATAAATTTGGACCAGGGGATTTTTTAGTTATTAATGCTCTCTGAAGAAAGGCAACATCTTTTTGAGAGCAGCATTGG
ACCACACCCCACAATCTCAAATGATTGAAATTCATGAACATCTAGGATCCCGTGAAGGTCACTGGACCCTGTTTTTTCTACTTCAAATCCTGTAGTAGCC

TACTGAATGAGAAAACATATTCTGACCCATTGGGATCAAATCAAAGGCACAGTGAACTCCTCATAGCATCTTCTTTGGAATTACTCAGGAACCAGAACT
TTTTACACAAATGTAAGAAATTCTACCAAGGAGTCCCCTTACCTAACAGCATCTCACAAGGCTGCACCAGATTCCAGAAAAGGCTTCTCTTGATACATC
AAGGTAGAACCTCTATGCATTTTGTGACCGACTTATTCTTAGATCATTGGTTTTCCAAAGGCTTTGTGGCCATGAAGCCCTTTGAGTGAAAACTGTGCA
GAAGCCCAGAGTAAAAGTGAAGCTGCTCTGGATGAAGTAGTGAAGCAAGAGTAGGGGCCTGAATCCTGCTACAACTATCTTCCTTTACCACCGTGGTG
ACACCTAAGGGGACTTCCTTACAACACCTTGAACTCTTCCGAACACAGTTTGAAAACCACTGCCCCAGACAGCAATATGTTTGACCTGAATGGCATTC
CAATCTTTTCTGTACCTCCACTCAGCACAGTTCATGTTCAGTAGATGCTGAACATTCTTAGAAATACTGTGTGTGAACTTAGAAAAGTGCAAGAAGACA
GGCATGTCTTTGACCCCAGGAATGATCATTTGCTGAAGATGGTGTCAAGTGAACCTAGATTAACAGCCCTCCACTCCAGATGGATATCCAGTGATTCCTA
GAATGGGATATAGCCAGAGAACAATTCTATGCACCCTACACTGACAGACTCCCTTAAGCAACACCAGATGCTCTACTGGTACTTGAAGTACATGACTTT
GAAGTCTTGACCCTCCATGAATACCTGAATTATCAGCAAGCGGGTTTTGAAGCTGGTGCCTCATTGAGGCCATATTAGAGCAACTTGTACATTTGACCTC
TTGTTATCAGCCATGGTACTCTACTTCGTGTGCAAGAGATAACTATGAAAGCCAAATTCAAATACTGGCAACATTTCCTAAAGGGGCTCAATATCTATCA
TTCGTCTTCTTTTCCAAACTACACATCACTGTATGACTCAACCAGTAGCAGTTATATTGCCCCTTGGTTTTTATTCAGTTTAACTACTGTTTCCAAGATAA
ATGAGCTAATAAGCTTTAAAAAAAAAAAAAAAAAAAGGCTGAATTCTTTTTTCTTCATCACTGGCATATCTGCCTATTCTCCAGAATTATTATGACTATTCA
GCTCACTTTAACAGTTGAACTTCAAGCGACAATCTTTGAACACCCCTTCTCATGTGATTTAAAATGAAACCATTTGGAAAAGTTTCTTCTAGCCAGTAAT
AGATTTTTTTTTTAATTGCTCTGCCTTGTGCCGAGAGATGTTCTTTTAAGATGAATCTTTTGATGTCTGATACCACCAAATATAGGTGGTAGGGAGAGTTG
GAGGCTGGCCCTTTGAGCAGGCCATTAGCTTACTTGCTGGGCATTTCCGATAGCTTATTGCCTACCTTTTTGCTGGAAACAAACTGATTTGAAAAACAA
AATCTATGAAGACTGCAGCTAAGGATTTTATCGGTAGACTTAAGAGCTTTTGTCCTTGTGGATATTTTAGTGGAACCACATCAGTCTCAATACTGTCATTT
TACACTGACTCAGAGCAGCTGACTTCATTCCTTGCCATGATATATATTTAAGGCAGGCATTGTAACAGACATAAAGACAACTTATCTGTTTCAGCAGGAA
GGATTCAGTTTATGAACTCTCAGACCAGATCATGTTGAACAAGGAGACTTTGATGTGTGTCATGAGAAAACTCATTCTTTACTTCCCAGTCAATTTAAA
GGCCAGCTATCCTGAGCTACTCGAATGAATGCACTGGTTAAACATTGGAAATAGTTTGTTTATATCCTTGTCTCTCTCTAGGCCAATTGTGATTACATGAC
TCGACTCTACATCTCGTCAAACAAGGCCTAGGTCTGGTTGCTGTAGACTGCTCGCCCTCAACAAATAAAATCTGGTTGACTAGCCTCCTTGTATATACAA
CTATTATTTGTTAAGAAGAAATTATCGTCAATTTTCTACTACCTTCCAATTGTCAGCTCTTTTTTTCCTCTCTGGTTTTTCCTATACTTTACAGAAAAAGAC
ATTGATCTATACTGCCATTCCCTCTAATCCTGCCATACTCAGTCAAAAGGAATGACTTAAGATGAAGATGATCATCTGCTCGAGTCTAAAATATACATTGT
ATATAAGAATTGGTGATTAGAAAAGCAAAAAACCTAAAACTTAAATCTAGGAGTCTGTATACTGTCTCCATGTCTCCATGCCTCAGATCTCATCTAAATC
TTTGAACAGCACCATTCAACCAATCTGAGGCCTTGACTTGCTTGTAAGATGATTCTCAGAGATCGGCTGAGTTAAAAAAGATGACGACTTGATTACCAA
AGAAAGTAGGGCCAACTTTGACAAATCTGGCTCTGCTGACCCTGTCACTCCCAGATGTAGCATAGACTCCTAAACAGAACCTCAAGTCTGATTGAGGA
TAAGGCCTTCTCCTGAGCTGAAAGTTCTTTGGCAGATGAGCAAGAAACTGAAAGCTGATGTACCTGACTGGCTCTGTAAGATCAGAAAACTGTATCCA

GAATAAGCCCTATGGATTAACCCCTGAGTACCCAGAGTAAAAACTAATTTACAGAACTTCCTTATTGATCTGCTGGTTCTTCCAGATCATATTCTGGCTAT
TGGTATGGCTGGCCTTTCTGAAGGTACCCTGCTTGTCTATTTTCCTGACTCAGCTCTTGCCTGCCTTTTTCACATGTTGCTGCAATTAGACTCACCGTGA
GGACTACAGTCAATTTCAGTCTATCTTGTGCCCAATACAACAAGGATTTTTAATAGTAACAACCCACACCTCACCCACTAGGACTCAATGTTCACAACA
GGAAGGACCATTGCTGCATACTCCTTGACCAGCAACTTTTTTGAAGATATTTTTAAGTGCAGAGTAGGCCTCTATTCCTGTATGTAATTGTTCATTTTCAG
CACCTGGAACCTCATCTATCGGGTCTGGAAGGAATACAGCAGTTCGAAAGCCGCGTCCATTTCTCTCCTTCAGTAGTGCAGAAATGAGTCCGATTCACC
AGTACACACAGAACTGTACCAGTTCAACCTAGCAAAAGAAGAAAAGTTTCCACTGTACTTAAAATTTACAGCTGACTCAAATTGCCTCACAGAATTAT
TTGATGTAGAAGGCTAGTTGTCTTACTTCAGATCAGCAGGACAGTTGGGCTCTCAGACTCATGACCACTGAGTTTGCTTGTGTTGAAACTGTGGTTTCA
TCCAACATATGCTATTGGACATGATTATTATTCCATTCAAATGGATTACAGACTTCTTGAGGACAGGACAAACTTATCTCTCATGGTGTTTTTTTAGAATA
CTTTTATAACCAAGGAAGAAACCATGCCAGCTGTTACCATTCAACTTCTTAAGCAGAGATTAAGCTTTTTCATATCTGTTCTTATCCTGGACATCAGTAGT
TTTTAATTGCCCAGCATCCGTTCCATCTTGTAACAACTCCCTGATGTTTCTTAAAACCACCTCTTCCTATTTTCAGTCTGTGGTTTGGACAGTCTGACCCA
ACCTTGAGCTTTGTGGGTGAACATGTAATTCAGACCTCATCAATCAGCAAATCCATCTGAACTGTGGAGGAGAAGCTCTCTTTACTGAGGGTGCTTTAG
CTTTGTAGGATGAAAACCTCAAACTAACAGGGCCTACCATGTAGAGAATGAAGCCAGTGCAGGGGAAAGCAGAGCCAAAATATGGAGAGACTTGAAT
CCTGATGACAGCGTTTGTGCCCCTGGATCCAACCGTGCCTGAAGCTAGAATATCCCCTGGACTTTTCAGTTATGTGAACCAA
>ENST00000540687.6|ENSG00000253352.9|OTTHUMG00000030444.6|OTTHUMT00000495126.1|RP3-430N8.2-020|TUG1|3308|
TGGGTTTTGAACCTGGGAACCTTGGATTGGAGTTGGGGATCCCCAAACTTCCTGAAATTGTGGGAATGTGCGGTTTGGGGGAATGATGGGAATTTGTG
GGAATGTGCGTTTTAGGGGAATGATGATCCATCGCTAGCAAGTTTTCCAAGGGGGCTGTGACCCAGAAGAGTTAAGAATCACAATTTCTTCATGCTACA
GAGAGGAAACTGAGGCCTAGATGTCATTTGGGACCCTTCACAACCATTTTGAAGCCCTGTTTGAGTCCCTGGGATATGTGAGCTGTTTCTATGCATAAT
GGATATTCGGGGTTAACAACAGTCCCCTGCTTGGCTTCTATTCTGAATCCTTTTCTTTCACCATGGGGTGCCTGAAGGGTGGCTGATGCATATGGTACAA
TGGCACCCAGTGTAAAGCAGCTACAATTAGGAGTGGATGTGTTCTGTAGCATCCTATTTAAATAAGCCTATTTTATCCTTTGGCCCGTCAACTCTGTTATC
TGCTGCTTGTACTGGTGCCTGTACTTTTCTGACTCTCATTGACCATATTCCACGACCATGGTTGTCATCCATTACTTGATCCTACTTTACATGTCTAGGCTG
TGTGGTTGGTGGTGAATAGGCTTCTTTTTACATGGTGCTGCCAGCCCAGCTAATTAATGGTGCACGTGGACTTTTAGCAAGCGGGCTCACTGGAAGAGA
CTGAACCTGGCATGGAATTCCTGAAGATGTTTGGGGTTTTTTTCTTTCTTAATCGAAAGTTAACATTGTCTGAAAAGTTTTGTTAGAACTACTGCGGAAC
CTCAAAATCAGTAGATTTGGAAGTGATTCAAAGCTAAACTTTTTCCTTGGCCCTCCTTGTGTTCTAATTGCTTGCAAGTGTAATACTAGGATGTCCAAGA
TGCCAGTTTTTGCTTCTTTGTTAGTTGTCAGCTGCTTTTATCAAATTTCAGGCCATTATCCAACAAACACTATAAAAATGTTTGAACAATTGGATTTCAAA
CATTTTCGTTTTGTGGAGTGGTGCTCACCAAGTGGTACAGCCCTAAGCAAGTGAACACAAACACATTTAAGTGTATTTTGTCTGATTAGATGTTAGCCA
GTTATGCTATTTCATTCAAATGTCTGAAAAAATCAATTGACTATTCCCTTTTCCTAAAGGGCAGAGACAGATAATCTCACTTCCAGAGAAATGACTTGGA

GAAAAAAAAGTGTTGGTCTTTTTGCTCTTTTGTAATTAAATCCGGATGTACCTCAAAAGACTTAAGACTGTGGTGATAAGATGCTTTCCTCAGCAGAAA
GGAGGGAAAAAAAACAACTGGAACTCAAAGCTTGAAATTCTGTGGCAAAACATGAGATGTCCAGGATTGGAGGTTGAAAAGATTTCACTACAGTGTT
CTGCAATAGTTGGAGCAGATAACTTTCAGTGTAGCCACAGCCATGGACTCCAGATTTCCAGATTTTCAAGACCTGGACCTGGAACCCGAAAGAGCTTG
TCACGATGCGGCAGGAACACTGGAGGTAGATTTTTTTTATTTTTGAATTTTGGGACTGTTGACCTTGCTGTGAGAAAAGAGACAACGACTGAGCAAG
CACTACCACCAGCACTGTTACTGGGAATTAGAAGACCTGAGTTTCTGTCCAGACCCTCAGTGCAAACTGAGGATGCTCCATCCAAAGTGAATTATGTCC
TGTGCCTCCTGATTGCTGAGTGTTCACCTGGACCTTCTGACTACCTTCCCTGTGCTATTCCATCAGCCTACAGACCTGGTACCTGGATTTTTGCCCGAGA
TGATTCCTACCACCTTACTACTGACGAAGACACCCATTCCAGTGGACCACTGTGACCCAGGAGGCATTCAGCCATCATGATGTGGCCTTTACCTCCACT
CCTGTCTTGTTCTACCCAGATTCAGCACAGCCCTTTATAGTGAAGTCAGAGTCCTCAAGCCAAATAGCTAAAGCTGTTTTATCACAACAAAGGCCTAGT
TTGTTCCATGAGTGTGCATTTCATTTCTTCAGTTAAAGCCTTCAGAGACACACAATAAATTTGGACCAGGGGATTTTTAGTTATTAATGCTCTCTGAAG
AAAGGCAACATCTTTTTGAGAGCAGCATTGGACCACACCCCACAATCTCAAATGATTGAAATTCATGAACATCTAGGATCCCGTGAAGGTCACTGGAC
CCTGTTTTTTCTACTTCAAATCCTGTAGTAGCCTACTGAATGAGAAAACATATTCTGACCCATTGGGATCAAATCAAAGGCACAGTGAACTCCTCATAGC
ATCTTCTTTGGAATTACTCAGGAACCAGAACTTTTTACACAAATGTAAGAAATTCTACCAAGGAGTCCCCTTACCTAACAGCATCTCACAAGGCTGCAC
CAGATTCCAGAAAAGGCTTCTCTTGATACATCAAGCATTTTGTGACCGACTTATTCTTAGATCATTGGTTTTCCAAAGGCTTTGTGGCCATGAAGCCCTT
TGAGTGAAAACTGTGCAGAAGCCCAGAGTAAAAGTGAAGCTGCTCTGGATGAAGTAGTGAAGCAAGAGTAGGGGCCTGAATCCTGCTACAACTATCT
TCCTTTACCACCGTGGTGACACCTAAGGGGACTTCCTTACAACACCTTGAACTCTTCCGAACACAGTTTGAAAACCACTGCCCCAGACAGCAATATGT
TTGACCTGAATGGCATTCCAATCTTTTCTGTACCTCCACTCAGCACAGTTCATGTTCAGTAGATGCTGAACATTCTTAGAAATACTGTGTGTGAACTTAG
AAAAGTGCAAGAAGACAGGCATGTCTTTGACCCCAGGAATGATCATTTGCTGAAGATGGTGTCAAGTGAACCTAGATTAACAGCCCTCCACTCCAGAT
GGATATCCAGTGATTCCTAGAATGGGATATAGCCAGAGAACAATTCTATGCACCCTACACTGACAGACTCCCTTAAGCAACACCAGATGCTCTACTGGT
ACTTGAAGTACATGACTTTGAAGTCTTGACCCTCCATGAATACCTGAATTATCAGCAAGCGGGTTTTGAAGCTGGTGCCTCATTGAGGCCATATTAGAG
CAACTTGTACATTTGACCTCTTGTTATCAGCCATGGTACTCTACTTCGTGTGCAAGAGATAACTATGAAAGCCAAATTCAAATACTGGCAACATTTCCTA
AAGGGGCTCAATATCTATCATTCGTCTTCTTTTCCAAACTACACATCACTGTATGACTCAACCAGTAGCAGTTATATTGCCCCTTGGTTTTTATTCAGTTTA
ACTACTGTTTCCAAGATAAATGAGCTAATAA
>ENST00000644027.1|ENSG00000253352.9|OTTHUMG00000030444.6|OTTHUMT00000467407.2|RP3-430N8.2-009|TUG1|7084|
GGAATTTGTGGGAATGTGCGTTTTAGGGGAATGATGATCCATCGCTAGCAAGTTTTCCAAGGGGGCTGTGACCCAGAAGAGTTAAGAATCACAATTTCT
TCATGCTACAGAGAGGAAACTGAGGCCTAGATGTCATTTGGGACCCTTCACAACCATTTTGAAGCCCTGTTTGAGTCCCTGGGATATGTGAGCTGTTTC
TATGCATAATGGATATTCGGGGTTAACAACAGTCCCCTGCTTGGCTTCTATTCTGAATCCTTTTCTTTCACCATGGGGTGCCTGAAGGGTGGCTGATGCAT

ATGGTACAATGGCACCCAGTGTAAAGCAGCTACAATTAGGAGTGGATGTGTTCTGTAGCATCCTATTTAAATAAGCCTATTTTATCCTTTGGCCCGTCAA
CTCTGTTATCTGCTGCTTGTACTGGTGCCTGTACTTTTCTGACTCTCATTGACCATATTCCACGACCATGGTTGTCATCCATTACTTGATCCTACTTTACAT
GTCTAGGCTGTGTGGTTGGTGGTGAATAGGCTTCTTTTTACATGGTGCTGCCAGCCCAGCTAATTAATGGTGCACGTGGACTTTTAGCAAGCGGGCTCA
CTGGAAGAGACTGAACCTGGCATGGAATTCCTGAAGATGTTTGGGGTTTTTTTCTTTCTTAATCGAAAGTTAACATTGTCTGAAAAGTTTTGTTAGAAC
TACTGCGGAACCTCAAAATCAGTAGATTTGGAAGTGATTCAAAGCTAAACTTTTTCCTTGGCCCTCCTTGTGTTCTAATTGCTTGCAAGTGTAATACTAG
GATGTCCAAGATGCCAGTTTTTGCTTCTTTGTTAGTTGTCAGCTGCTTTTATCAAATTTCAGGCCATTATCCAACAAACACTATAAAAATGTTTGAACAAT
TGGATTTCAAACATTTTCGTTTTGTGGAGTGGTGCTCACCAAGTGGTACAGCCCTAAGCAAGTGAACACAAACACATTTAAGTGTATTTTGTCTGATTA
GATGTTAGCCAGTTATGCTATTTCATTCAAATGTCTGAAAAAATCAATTGACTATTCCCTTTTCCTAAAGGGCAGAGACAGATAATCTCACTTCCAGAGA
AATGACTTGGAGAAAAAAAAGTGTTGGTCTTTTTGCTCTTTTGTAATTAAATCCGGATGTACCTCAAAAGACTTAAGACTGTGGTGATAAGATGCTTTC
CTCAGCAGAAAGGAGGGAAAAAAAACAACTGGAACTCAAAGCTTGAAATTCTGTGGCAAAACATGAGATGTCCAGGATTGGAGGTTGAAAAGATTT
CACTACAGTGTTCTGCAATAGTTGGAGCAGATAACTTTCAGTGTAGCCACAGCCATGGACTCCAGATTTCCAGATTTTCAAGACCTGGACCTGGAACCC
GAAAGAGCTTGTCACGATGCGGCAGGAACACTGGAGGTAGATTTTTTTTATTTTTGAATTTTGGGACTGTTGACCTTGCTGTGAGAAAAGAGACAAC
GACTGAGCAAGCACTACCACCAGCACTGTTACTGGGAATTAGAAGACCTGAGTTTCTGTCCAGACCCTCAGTGCAAACTGAGGATGCTCCATCCAAA
GTGAATTATGGTACTTGCCATTTTCCAAAATGCCTTATCCTTTACCATCTCTGCACTTTTGTTCATACTCTCATTCTACTTTGGAACTGCTGCTCTGTGGCT
TTTCATCTGTCAAAACTGCCATTTTCTCAGTATCCAACTCTTATGCCCTCTTTTCCATGAGTCTCCTAACTAGCCAGAATAGAGCTTTAAAGTTTTATGAC
ATTTCGTTATGTATCCTCTATCTGTATACAAAATCCTGTAAAATAGTTACTTGCCTGCATTTACTGTCTTTGCAGATAGCAGACTCCTTGAAAGCAGGGTC
CTTGTTTAGTGCATCTTTGCCCACATACACCACAACATATCAAGATGCATTTATTAGGAAGGAGGAGTTTAGAGAGCAGGCTATCAGAATAACCACTCA
GTAAGTGTTTTCTTAATTGCTATGTGATAACTTACATTACTCTTAATGAGGAGAAAAGTCGCTAGATATGGATCATGTTTATGTTTTAATGTTTTTTAATTCT
AAATTTTGATCTAGGGAGCCCTCAGACATAAGGAGAAACCAATATGTTGAATGATGGGGTATTACATACAGAAGGACCAAGACCTTATTTTCTCCTACTT
AAGATGGAATCTTGTTAAGATGAAAGGACAGCTTTTTAAAAGGAGAAGGTCATAAGACAGTTTGAGGAAGGCATTGGAAGAGGAAGAGGGGCAATGT
CTCCTTTGTTTTATTGTTGGTATATAAACTTAAAATCTCAGTTCTTTTTATGGCACTTGTGGAGCCACTTCCTCTCCTCACCAAAAAATGCCCAGTACCCT
GAATCCGATCAAATTACTCTCCTAAAGTATAAGGCTTAGTTTCTGTGTGCTGCTTCGCGAGACAGTTCCCACAAATCGAGAGTGTTAATCAGACTTTTGT
GTTTTCTTTTTTGGATTGCTGTTGTTTGTTTTCATTTATCATTTGTCTTTGCCTAAGCCAGGCTCATCAAGAATTAACAGCCATCAGGCTGCCGATGTGCT
GACAGCAGACCCACTTAGAGTCCTGTGTTTGTAATTCCATGCATTTGTTATTTTACCTGTTTTGTCCCTGCCCCTTCTAGTCCTGTGCCTCCTGATTGCTG
AGTGTTCACCTGGACCTTCTGACTACCTTCCCTGTGCTATTCCATCAGCCTACAGACCTGGTACCTGGATTTTTGCCCGAGATGATTCCTACCACCTTACT
ACTGACGAAGACACCCATTCCAGTGGACCACTGTGACCCAGGAGGCATTCAGCCATCATGATGTGGCCTTTACCTCCACTCCTGTCTTGTTCTACCCAG

ATTCAGCACAGCCCTTTATAGTGAAGTCAGAGTCCTCAAGCCAAATAGCTAAAGCTGTTTTATCACAACAAAGGCCTAGTTTGTTCCATGAGTGTGCAT
TTCATTTCTTCAGTTAAAGCCTTCAGAGACACACAATAAATTTGGACCAGGGGATTTTTTAGTTATTAATGCTCTCTGAAGAAAGGCAACATCTTTTTGA
GAGCAGCATTGGACCACACCCCACAATCTCAAATGATTGAAATTCATGAACATCTAGGATCCCGTGAAGGTCACTGGACCCTGTTTTTTCTACTTCAAA
TCCTGTAGTAGCCTACTGAATGAGAAAACATATTCTGACCCATTGGGATCAAATCAAAGGCACAGTGAACTCCTCATAGCATCTTCTTTGGAATTACTCA
GGAACCAGAACTTTTTACACAAATGTAAGAAATTCTACCAAGGAGTCCCCTTACCTAACAGCATCTCACAAGGCTGCACCAGATTCCAGAAAAGGCTT
CTCTTGATACATCAAGCATTTTGTGACCGACTTATTCTTAGATCATTGGTTTTCCAAAGGCTTTGTGGCCATGAAGCCCTTTGAGTGAAAACTGTGCAGA
AGCCCAGAGTAAAAGTGAAGCTGCTCTGGATGAAGTAGTGAAGCAAGAGTAGGGGCCTGAATCCTGCTACAACTATCTTCCTTTACCACCGTGGTGAC
ACCTAAGGGGACTTCCTTACAACACCTTGAACTCTTCCGAACACAGTTTGAAAACCACTGCCCCAGACAGCAATATGTTTGACCTGAATGGCATTCCA
ATCTTTTCTGTACCTCCACTCAGCACAGTTCATGTTCAGTAGATGCTGAACATTCTTAGAAATACTGTGTGTGAACTTAGAAAAGTGCAAGAAGACAGG
CATGTCTTTGACCCCAGGAATGATCATTTGCTGAAGATGGTGTCAAGTGAACCTAGATTAACAGCCCTCCACTCCAGATGGATATCCAGTGATTCCTAGA
ATGGGATATAGCCAGAGAACAATTCTATGCACCCTACACTGACAGACTCCCTTAAGCAACACCAGATGCTCTACTGGTACTTGAAGTACATGACTTTGA
AGTCTTGACCCTCCATGAATACCTGAATTATCAGCAAGCGGGTTTTGAAGCTGGTGCCTCATTGAGGCCATATTAGAGCAACTTGTACATTTGACCTCTT
GTTATCAGCCATGGTACTCTACTTCGTGTGCAAGAGATAACTATGAAAGCCAAATTCAAATACTGGCAACATTTCCTAAAGGGGCTCAATATCTATCATT
CGTCTTCTTTTCCAAACTACACATCACTGTATGACTCAACCAGTAGCAGTTATATTGCCCCTTGGTTTTTATTCAGTTTAACTACTGTTTCCAAGATAAAT
GAGCTAATAAGCTTTAAAAAAAAAAAAAAAAAAAGGCTGAATTCTTTTTTCTTCATCACTGGCATATCTGCCTATTCTCCAGAATTATTATGACTATTCAG
CTCACTTTAACAGTTGAACTTCAAGCGACAATCTTTGAACACCCCTTCTCATGTGATTTAAAATGAAACCATTTGGAAAAGTTTCTTCTAGCCAGTAATA
GATTTTTTTTTTAATTGCTCTGCCTTGTGCCGAGAGATGTTCTTTTAAGATGAATCTTTTGATGTCTGATACCACCAAATATAGGTGGTAGGGAGAGTTGG
AGGCTGGCCCTTTGAGCAGGCCATTAGCTTACTTGCTGGGCATTTCCGATAGCTTATTGCCTACCTTTTTGCTGGAAACAAACTGATTTGAAAAACAAA
ATCTATGAAGACTGCAGCTAAGGATTTTATCGGTAGACTTAAGAGCTTTTGTCCTTGTGGATATTTTAGTGGAACCACATCAGTCTCAATACTGTCATTTT
ACACTGACTCAGAGCAGCTGACTTCATTCCTTGCCATGATATATATTTAAGGCAGGCATTGTAACAGACATAAAGACAACTTATCTGTTTCAGCAGGAA
GGATTCAGTTTATGAACTCTCAGACCAGATCATGTTGAACAAGGAGACTTTGATGTGTGTCATGAGAAAACTCATTCTTTACTTCCCAGTCAATTTAAA
GGCCAGCTATCCTGAGCTACTCGAATGAATGCACTGGTTAAACATTGGAAATAGTTTGTTTATATCCTTGTCTCTCTCTAGGCCAATTGTGATTACATGAC
TCGACTCTACATCTCGTCAAACAAGGCCTAGGTCTGGTTGCTGTAGACTGCTCGCCCTCAACAAATAAAATCTGGTTGACTAGCCTCCTTGTATATACAA
CTATTATTTGTTAAGAAGAAATTATCGTCAATTTTCTACTACCTTCCAATTGTCAGCTCTTTTTTTCCTCTCTGGTTTTTCCTATACTTTACAGAAAAAGAC
ATTGATCTATACTGCCATTCCCTCTAATCCTGCCATACTCAGTCAAAAGGAATGACTTAAGATGAAGATGATCATCTGCTCGAGTCTAAAATATACATTGT
ATATAAGAATTGGTGATTAGAAAAGCAAAAAACCTAAAACTTAAATCTAGGAGTCTGTATACTGTCTCCATGTCTCCATGCCTCAGATCTCATCTAAATC

TTTGAACAGCACCATTCAACCAATCTGAGGCCTTGACTTGCTTGTAAGATGATTCTCAGAGATCGGCTGAGTTAAAAAAGATGACGACTTGATTACCAA
AGAAAGTAGGGCCAACTTTGACAAATCTGGCTCTGCTGACCCTGTCACTCCCAGATGTAGCATAGACTCCTAAACAGAACCTCAAGTCTGATTGAGGA
TAAGGCCTTCTCCTGAGCTGAAAGTTCTTTGGCAGATGAGCAAGAAACTGAAAGCTGATGTACCTGACTGGCTCTGTAAGATCAGAAAACTGTATCCA
GAATAAGCCCTATGGATTAACCCCTGAGTACCCAGAGTAAAAACTAATTTACAGAACTTCCTTATTGATCTGCTGGTTCTTCCAGATCATATTCTGGCTAT
TGGTATGGCTGGCCTTTCTGAAGGTACCCTGCTTGTCTATTTTCCTGACTCAGCTCTTGCCTGCCTTTTTCACATGTTGCTGCAATTAGACTCACCGTGA
GGACTACAGTCAATTTCAGTCTATCTTGTGCCCAATACAACAAGGATTTTTAATAGTAACAACCCACACCTCACCCACTAGGACTCAATGTTCACAACA
GGAAGGACCATTGCTGCATACTCCTTGACCAGCAACTTTTTTGAAGATATTTTTAAGTGCAGAGTAGGCCTCTATTCCTGTATGTAATTGTTCATTTTCAG
CACCTGGAACCTCATCTATCGGGTCTGGAAGGAATACAGCAGTTCGAAAGCCGCGTCCATTTCTCTCCTTCAGTAGTGCAGAAATGAGTCCGATTCACC
AGTACACACAGAACTGTACCAGTTCAACCTAGCAAAAGAAGAAAAGTTTCCACTGTACTTAAAATTTACAGCTGACTCAAATTGCCTCACAGAATTAT
TTGATGTAGAAGGCTAGTTGTCTTACTTCAGATCAGCAGGACAGTTGGGCTCTCAGACTCATGACCACTGAGTTTGCTTGTGTTGAAACTGTGGTTTCA
TCCAACATATGCTATTGGACATGATTATTATTCCATTCAAATGGATTACAGACTTCTTGAGGACAGGACAAACTTATCTCTCATGGTGTTTTTTTAGAATA
CTTTTATAACCAAGGAAGAAACCATGCCAGCTGTTACCATTCAACTTCTTAAGCAGAGATTAAGCTTTTTCATATCTGTTCTTATCCTGGACATCAGTAGT
TTTTAATTGCCCAGCATCCGTTCCATCTTGTAACAACTCCCTGATGTTTCTTAAAACCACCTCTTCCTATTTTCAGTCTGTGGTTTGGACAGTCTGACCCA
ACCTTGAGCTTTGTGGGTGAACATGTAATTCAGACCTCATCAATCAGCAAATCCATCTGAACTGTGGAGGAGAAGCTCTCTTTACTGAGGGTGCTTTAG
CTTTGTAGGATGAAAACCTCAAACTAACAGGGCCTACCATGTAGAGAATGAAGCCAGTGCAGGGGAAAGCAGAGCCAAAATATGGAGAGACTTGAAT
CCTGATGACAGCGTTTGTGCCCCTGGATCCAACCGTGCCTGAAGCTAGAATATCCCCTGGACTTTTCAGTTATGTGAACCAATAAATACCCTTTTTTGCT
TAAGT
>ENST00000647354.1|ENSG00000253352.9|OTTHUMG00000030444.6|OTTHUMT00000431950.3|RP3-430N8.2-003|TUG1|5875|
GAGGCCTAGATGTCATTTGGGACCCTTCACAACCATTTTGAAGCCCTGTTTGAGTCCCTGGGATATGTGAGCTGTTTCTATGCATAATGGATATTCGGGG
TTAACAACAGTCCCCTGCTTGGCTTCTATTCTGAATCCTTTTCTTTCACCATGGGGTGCCTGAAGGGTGGCTGATGCATATGGTACAATGGCACCCAGTG
TAAAGCAGCTACAATTAGGAGTGGATGTGTTCTGTAGCATCCTATTTAAATAAGCCTATTTTATCCTTTGGCCCGTCAACTCTGTTATCTGCTGCTTGTAC
TGGTGCCTGTACTTTTCTGACTCTCATTGACCATATTCCACGACCATGGTTGTCATCCATTACTTGATCCTACTTTACATGTCTAGGCTGTGTGGTTGGTG
GTGAATAGGCTTCTTTTTACATGGTGCTGCCAGCCCAGCTAATTAATGGTGCACGTGGACTTTTAGCAAGCGGGCTCACTGGAAGAGACTGAACCTGG
CATGGAATTCCTGAAGATGTTTGGGGTTTTTTTCTTTCTTAATCGAAAGTTAACATTGTCTGAAAAGTTTTGTTAGAACTACTGCGGAACCTCAAAATCA
GTAGATTTGGAAGTGATTCAAAGCTAAACTTTTTCCTTGGCCCTCCTTGTGTTCTAATTGCTTGCAAGTGTAATACTAGGATGTCCAAGATGCCAGTTTT
TGCTTCTTTGTTAGTTGTCAGCTGCTTTTATCAAATTTCAGGCCATTATCCAACAAACACTATAAAAATGTTTGAACAATTGGATTTCAAACATTTTCGTT

TTGTGGAGTGGTGCTCACCAAGTGGTACAGCCCTAAGCAAGTGAACACAAACACATTTAAGTGTATTTTGTCTGATTAGATGTTAGCCAGTTATGCTATT
TCATTCAAATGTCTGAAAAAATCAATTGACTATTCCCTTTTCCTAAAGGGCAGAGACAGATAATCTCACTTCCAGAGAAATGACTTGGAGAAAAAAAA
GTGTTGGTCTTTTTGCTCTTTTGTAATTAAATCCGGATGTACCTCAAAAGACTTAAGACTGTGGTGATAAGATGCTTTCCTCAGCAGAAAGGAGGGAAA
AAAAACAACTGGAACTCAAAGCTTGAAATTCTGTGGCAAAACATGAGATGTCCAGGATTGGAGGTTGAAAAGATTTCACTACAGTGTTCTGCAATAGT
TGGAGCAGATAACTTTCAGTGTAGCCACAGCCATGGACTCCAGATTTCCAGATTTTCAAGACCTGGACCTGGAACCCGAAAGAGCTTGTCACGATGCG
GCAGGAACACTGGAGGTAGATTTTTTTTTATTTTTGAATTTTGGGACTGTTGACCTTGCTGTGAGAAAAGAGACAACGACTGAGCAAGCACTACCACC
AGCACTGTTACTGGGAATTAGAAGACCTGAGTTTCTGTCCAGACCCTCAGTGCAAACTGAGGATGCTCCATCCAAAGTGAATTATGTCCTGTGCCTCCT
GATTGCTGAGTGTTCACCTGGACCTTCTGACTACCTTCCCTGTGCTATTCCATCAGCCTACAGACCTGGTACCTGGATTTTTGCCCGAGATGATTCCTAC
CACCTTACTACTGACGAAGACACCCATTCCAGTGGACCACTGTGACCCAGGAGGCATTCAGCCATCATGATGTGGCCTTTACCTCCACTCCTGTCTTGT
TCTACCCAGATTCAGCACAGCCCTTTATAGTGAAGTCAGAGTCCTCAAGCCAAATAGCTAAAGCTGTTTTATCACAACAAAGGCCTAGTTTGTTCCATG
AGTGTGCATTTCATTTCTTCAGTTAAAGCCTTCAGAGACACACAATAAATTTGGACCAGGGGATTTTTTAGTTATTAATGCTCTCTGAAGAAAGGCAACA
TCTTTTTGAGAGCAGCATTGGACCACACCCCACAATCTCAAATGATTGAAATTCATGAACATCTAGGATCCCGTGAAGGTCACTGGACCCTGTTTTTTCT
ACTTCAAATCCTGTAGTAGCCTACTGAATGAGAAAACATATTCTGACCCATTGGGATCAAATCAAAGGCACAGTGAACTCCTCATAGCATCTTCTTTGG
AATTACTCAGGAACCAGAACTTTTTACACAAATGTAAGAAATTCTACCAAGGAGTCCCCTTACCTAACAGCATCTCACAAGGCTGCACCAGATTCCAG
AAAAGGCTTCTCTTGATACATCAAGGTAGAACCTCTATGCATTTTGTGACCGACTTATTCTTAGATCATTGGTTTTCCAAAGGCTTTGTGGCCATGAAGC
CCTTTGAGTGAAAACTGTGCAGAAGCCCAGAGTAAAAGTGAAGCTGCTCTGGATGAAGTAGTGAAGCAAGAGTAGGGGCCTGAATCCTGCTACAACT
ATCTTCCTTTACCACCGTGGTGACACCTAAGGGGACTTCCTTACAACACCTTGAACTCTTCCGAACACAGTTTGAAAACCACTGCCCCAGACAGCAAT
ATGTTTGACCTGAATGGCATTCCAATCTTTTCTGTACCTCCACTCAGCACAGTTCATGTTCAGTAGATGCTGAACATTCTTAGAAATACTGTGTGTGAAC
TTAGAAAAGTGCAAGAAGACAGGCATGTCTTTGACCCCAGGAATGATCATTTGCTGAAGATGGTGTCAAGTGAACCTAGATTAACAGCCCTCCACTCC
AGATGGATATCCAGTGATTCCTAGAATGGGATATAGCCAGAGAACAATTCTATGCACCCTACACTGACAGACTCCCTTAAGCAACACCAGATGCTCTAC
TGGTACTTGAAGTACATGACTTTGAAGTCTTGACCCTCCATGAATACCTGAATTATCAGCAAGCGGGTTTTGAAGCTGGTGCCTCATTGAGGCCATATTA
GAGCAACTTGTACATTTGACCTCTTGTTATCAGCCATGGTACTCTACTTCGTGTGCAAGAGATAACTATGAAAGCCAAATTCAAATACTGGCAACATTTC
CTAAAGGGGCTCAATATCTATCATTCGTCTTCTTTTCCAAACTACACATCACTGTATGACTCAACCAGTAGCAGTTATATTGCCCCTTGGTTTTTATTCAGT
TTAACTACTGTTTCCAAGATAAATGAGCTAATAAGCTTTAAAAAAAAAAAAAAAAAAAGGCTGAATTCTTTTTTCTTCATCACTGGCATATCTGCCTATTC
TCCAGAATTATTATGACTATTCAGCTCACTTTAACAGTTGAACTTCAAGCGACAATCTTTGAACACCCCTTCTCATGTGATTTAAAATGAAACCATTTGG
AAAAGTTTCTTCTAGCCAGTAATAGATTTTTTTTTTAATTGCTCTGCCTTGTGCCGAGAGATGTTCTTTTAAGATGAATCTTTTGATGTCTGATACCACCA

AATATAGGTGGTAGGGAGAGTTGGAGGCTGGCCCTTTGAGCAGGCCATTAGCTTACTTGCTGGGCATTTCCGATAGCTTATTGCCTACCTTTTTGCTGGA
AACAAACTGATTTGAAAAACAAAATCTATGAAGACTGCAGCTAAGGATTTTATCGGTAGACTTAAGAGCTTTTGTCCTTGTGGATATTTTAGTGGAACC
ACATCAGTCTCAATACTGTCATTTTACACTGACTCAGAGCAGCTGACTTCATTCCTTGCCATGATATATATTTAAGGCAGGCATTGTAACAGACATAAAG
ACAACTTATCTGTTTCAGCAGGAAGGATTCAGTTTATGAACTCTCAGACCAGATCATGTTGAACAAGGAGACTTTGATGTGTGTCATGAGAAAACTCAT
TCTTTACTTCCCAGTCAATTTAAAGGCCAGCTATCCTGAGCTACTCGAATGAATGCACTGGTTAAACATTGGAAATAGTTTGTTTATATCCTTGTCTCTCT
CTAGGCCAATTGTGATTACATGACTCGACTCTACATCTCGTCAAACAAGGCCTAGGTCTGGTTGCTGTAGACTGCTCGCCCTCAACAAATAAAATCTGG
TTGACTAGCCTCCTTGTATATACAACTATTATTTGTTAAGAAGAAATTATCGTCAATTTTCTACTACCTTCCAATTGTCAGCTCTTTTTTTCCTCTCTGGTTT
TTCCTATACTTTACAGAAAAAGACATTGATCTATACTGCCATTCCCTCTAATCCTGCCATACTCAGTCAAAAGGAATGACTTAAGATGAAGATGATCATCT
GCTCGAGTCTAAAATATACATTGTATATAAGAATTGGTGATTAGAAAAGCAAAAAACCTAAAACTTAAATCTAGGAGTCTGTATACTGTCTCCATGTCTC
CATGCCTCAGATCTCATCTAAATCTTTGAACAGCACCATTCAACCAATCTGAGGCCTTGACTTGCTTGTAAGATGATTCTCAGAGATCGGCTGAGTTAAA
AAAGATGACGACTTGATTACCAAAGAAAGTAGGGCCAACTTTGACAAATCTGGCTCTGCTGACCCTGTCACTCCCAGATGTAGCATAGACTCCTAAAC
AGAACCTCAAGTCTGATTGAGGATAAGGCCTTCTCCTGAGCTGAAAGTTCTTTGGCAGATGAGCAAGAAACTGAAAGCTGATGTACCTGACTGGCTCT
GTAAGATCAGAAAACTGTATCCAGAATAAGCCCTATGGATTAACCCCTGAGTACCCAGAGTAAAAACTAATTTACAGAACTTCCTTATTGATCTGCTGGT
TCTTCCAGATCATATTCTGGCTATTGGTATGGCTGGCCTTTCTGAAGGTACCCTGCTTGTCTATTTTCCTGACTCAGCTCTTGCCTGCCTTTTTCACATGTT
GCTGCAATTAGACTCACCGTGAGGACTACAGTCAATTTCAGTCTATCTTGTGCCCAATACAACAAGGATTTTTAATAGTAACAACCCACACCTCACCCA
CTAGGACTCAATGTTCACAACAGGAAGGACCATTGCTGCATACTCCTTGACCAGCAACTTTTTTGAAGATATTTTTAAGTGCAGAGTAGGCCTCTATTCC
TGTATGTAATTGTTCATTTTCAGCACCTGGAACCTCATCTATCGGGTCTGGAAGGAATACAGCAGTTCGAAAGCCGCGTCCATTTCTCTCCTTCAGTAGT
GCAGAAATGAGTCCGATTCACCAGTACACACAGAACTGTACCAGTTCAACCTAGCAAAAGAAGAAAAGTTTCCACTGTACTTAAAATTTACAGCTGAC
TCAAATTGCCTCACAGAATTATTTGATGTAGAAGGCTAGTTGTCTTACTTCAGATCAGCAGGACAGTTGGGCTCTCAGACTCATGACCACTGAGTTTGC
TTGTGTTGAAACTGTGGTTTCATCCAACATATGCTATTGGACATGATTATTATTCCATTCAAATGGATTACAGACTTCTTGAGGACAGGACAAACTTATCT
CTCATGGTGTTTTTTTAGAATACTTTTATAACCAAGGAAGAAACCATGCCAGCTGTTACCATTCAACTTCTTAAGCAGAGATTAAGCTTTTTCATATCTGT
TCTTATCCTGGACATCAGTAGTTTTTAATTGCCCAGCATCCGTTCCATCTTGTAACAACTCCCTGATGTTTCTTAAAACCACCTCTTCCTATTTTCAGTCTG
TGGTTTGGACAGTCTGACCCAACCTTGAGCTTTGTGGGTGAACATGTAATTCAGACCTCATCAATCAGCAAATCCATCTGAACTGTGGAGGAGAAGCT
CTCTTTACTGAGGGTGCTTTAGCTTTGTAGGATGAAAACCTCAAACTAACAGGGCCTACCATGTAGAGAATGAAGCCAGTGCAGGGGAAAGCAGAGC
CAAAATATGGAGAGACTTGAATCCTGATGACAGCGTTTGTGCCCCTGGATCCAACCGTGCCTGAAGCTAGAATATCCCCTGGACTTTTCAGTTATGTGA
>ENST00000643553.1|ENSG00000253352.9|OTTHUMG00000030444.6|OTTHUMT00000495127.1|RP3-430N8.2-018|TUG1|5412|

CTGTGTGGTTGGTGGTGAATAGGCTTCTTTTTACATGGTGCTGCCAGCCCAGCTAATTAATGGTGCACGTGGACTTTTAGCAAGCGGGCTCACTGGAAG
AGACTGAACCTGGCATGGAATTCCTGAAGATGTTTGGGGTTTTTTTCTTTCTTAATCGAAAGTTAACATTGTCTGAAAAGTTTTGTTAGAACTACTGCGG
AACCTCAAAATCAGTAGATTTGGAAGTGATTCAAAGCTAAACTTTTTCCTTGGCCCTCCTTGTGTTCTAATTGCTTGCAAGTGTAATACTAGGATGTCCA
AGATGCCAGTTTTTGCTTCTTTGTTAGTTGTCAGCTGCTTTTATCAAATTTCAGGCCATTATCCAACAAACACTATAAAAATGTTTGAACAATTGGATTTC
AAACATTTTCGTTTTGTGGAGTGGTGCTCACCAAGTGGTACAGCCCTAAGCAAGTGAACACAAACACATTTAAGTGTATTTTGTCTGATTAGATGTTAG
CCAGTTATGCTATTTCATTCAAATGTCTGAAAAAATCAATTGACTATTCCCTTTTCCTAAAGGGCAGAGACAGATAATCTCACTTCCAGAGAAATGACTT
GGAGAAAAAAAAGTGTTGGTCTTTTTGCTCTTTTGTAATTAAATCCGGATGTACCTCAAAAGACTTAAGACTGTGGTGATAAGATGCTTTCCTCAGCAG
AAAGGAGGGAAAAAAAACAACTGGAACTCAAAGCTTGAAATTCTGTGGCAAAACATGAGATGTCCAGGATTGGAGGTTGAAAAGATTTCACTACAG
TGTTCTGCAATAGTTGGAGCAGATAACTTTCAGTGTAGCCACAGCCATGGACTCCAGATTTCCAGATTTTCAAGACCTGGACCTGGAACCCGAAAGAG
CTTGTCACGATGCGGCAGGAACACTGGAGGTAGATTTTTTTTATTTTTGAATTTTGGGACTGTTGACCTTGCTGTGAGAAAAGAGACAACGACTGAG
CAAGCACTACCACCAGCACTGTTACTGGGAATTAGAAGACCTGAGTTTCTGTCCAGACCCTCAGTGCAAACTGAGGATGCTCCATCCAAACCTACAGA
CCTGGTACCTGGATTTTTGCCCGAGATGATTCCTACCACCTTACTACTGACGAAGACACCCATTCCAGTGGACCACTGTGACCCAGGAGGCATTCAGCC
ATCATGATGTGGCCTTTACCTCCACTCCTGTCTTGTTCTACCCAGATTCAGCACAGCCCTTTATAGTGAAGTCAGAGTCCTCAAGCCAAATAGCTAAAGC
TGTTTTATCACAACAAAGGCCTAGTTTGTTCCATGAGTGTGCATTTCATTTCTTCAGTTAAAGCCTTCAGAGACACACAATAAATTTGGACCAGGGGATT
TTTTAGTTATTAATGCTCTCTGAAGAAAGGCAACATCTTTTTGAGAGCAGCATTGGACCACACCCCACAATCTCAAATGATTGAAATTCATGAACATCTA
GGATCCCGTGAAGGTCACTGGACCCTGTTTTTTCTACTTCAAATCCTGTAGTAGCCTACTGAATGAGAAAACATATTCTGACCCATTGGGATCAAATCAA
AGGCACAGTGAACTCCTCATAGCATCTTCTTTGGAATTACTCAGGAACCAGAACTTTTTACACAAATGTAAGAAATTCTACCAAGGAGTCCCCTTACCT
AACAGCATCTCACAAGGCTGCACCAGATTCCAGAAAAGGCTTCTCTTGATACATCAAGGTAGAACCTCTATGCATTTTGTGACCGACTTATTCTTAGATC
ATTGGTTTTCCAAAGGCTTTGTGGCCATGAAGCCCTTTGAGTGAAAACTGTGCAGAAGCCCAGAGTAAAAGTGAAGCTGCTCTGGATGAAGTAGTGA
AGCAAGAGTAGGGGCCTGAATCCTGCTACAACTATCTTCCTTTACCACCGTGGTGACACCTAAGGGGACTTCCTTACAACACCTTGAACTCTTCCGAAC
ACAGTTTGAAAACCACTGCCCCAGACAGCAATATGTTTGACCTGAATGGCATTCCAATCTTTTCTGTACCTCCACTCAGCACAGTTCATGTTCAGTAGA
TGCTGAACATTCTTAGAAATACTGTGTGTGAACTTAGAAAAGTGCAAGAAGACAGGCATGTCTTTGACCCCAGGAATGATCATTTGCTGAAGATGGTG
TCAAGTGAACCTAGATTAACAGCCCTCCACTCCAGATGGATATCCAGTGATTCCTAGAATGGGATATAGCCAGAGAACAATTCTATGCACCCTACACTG
ACAGACTCCCTTAAGCAACACCAGATGCTCTACTGGTACTTGAAGTACATGACTTTGAAGTCTTGACCCTCCATGAATACCTGAATTATCAGCAAGCGG
GTTTTGAAGCTGGTGCCTCATTGAGGCCATATTAGAGCAACTTGTACATTTGACCTCTTGTTATCAGCCATGGTACTCTACTTCGTGTGCAAGAGATAAC
TATGAAAGCCAAATTCAAATACTGGCAACATTTCCTAAAGGGGCTCAATATCTATCATTCGTCTTCTTTTCCAAACTACACATCACTGTATGACTCAACC

AGTAGCAGTTATATTGCCCCTTGGTTTTTATTCAGTTTAACTACTGTTTCCAAGATAAATGAGCTAATAAGCTTTAAAAAAAAAAAAAAAAAAAGGCTGA
ATTCTTTTTTCTTCATCACTGGCATATCTGCCTATTCTCCAGAATTATTATGACTATTCAGCTCACTTTAACAGTTGAACTTCAAGCGACAATCTTTGAACA
CCCCTTCTCATGTGATTTAAAATGAAACCATTTGGAAAAGTTTCTTCTAGCCAGTAATAGATTTTTTTTTTAATTGCTCTGCCTTGTGCCGAGAGATGTTC
TTTTAAGATGAATCTTTTGATGTCTGATACCACCAAATATAGGTGGTAGGGAGAGTTGGAGGCTGGCCCTTTGAGCAGGCCATTAGCTTACTTGCTGGG
CATTTCCGATAGCTTATTGCCTACCTTTTTGCTGGAAACAAACTGATTTGAAAAACAAAATCTATGAAGACTGCAGCTAAGGATTTTATCGGTAGACTTA
AGAGCTTTTGTCCTTGTGGATATTTTAGTGGAACCACATCAGTCTCAATACTGTCATTTTACACTGACTCAGAGCAGCTGACTTCATTCCTTGCCATGATA
TATATTTAAGGCAGGCATTGTAACAGACATAAAGACAACTTATCTGTTTCAGCAGGAAGGATTCAGTTTATGAACTCTCAGACCAGATCATGTTGAACA
AGGAGACTTTGATGTGTGTCATGAGAAAACTCATTCTTTACTTCCCAGTCAATTTAAAGGCCAGCTATCCTGAGCTACTCGAATGAATGCACTGGTTAA
ACATTGGAAATAGTTTGTTTATATCCTTGTCTCTCTCTAGGCCAATTGTGATTACATGACTCGACTCTACATCTCGTCAAACAAGGCCTAGGTCTGGTTGC
TGTAGACTGCTCGCCCTCAACAAATAAAATCTGGTTGACTAGCCTCCTTGTATATACAACTATTATTTGTTAAGAAGAAATTATCGTCAATTTTCTACTAC
CTTCCAATTGTCAGCTCTTTTTTTCCTCTCTGGTTTTTCCTATACTTTACAGAAAAAGACATTGATCTATACTGCCATTCCCTCTAATCCTGCCATACTCAG
TCAAAAGGAATGACTTAAGATGAAGATGATCATCTGCTCGAGTCTAAAATATACATTGTATATAAGAATTGGTGATTAGAAAAGCAAAAAACCTAAAAC
TTAAATCTAGGAGTCTGTATACTGTCTCCATGTCTCCATGCCTCAGATCTCATCTAAATCTTTGAACAGCACCATTCAACCAATCTGAGGCCTTGACTTGC
TTGTAAGATGATTCTCAGAGATCGGCTGAGTTAAAAAAGATGACGACTTGATTACCAAAGAAAGTAGGGCCAACTTTGACAAATCTGGCTCTGCTGAC
CCTGTCACTCCCAGATGTAGCATAGACTCCTAAACAGAACCTCAAGTCTGATTGAGGATAAGGCCTTCTCCTGAGCTGAAAGTTCTTTGGCAGATGAGC
AAGAAACTGAAAGCTGATGTACCTGACTGGCTCTGTAAGATCAGAAAACTGTATCCAGAATAAGCCCTATGGATTAACCCCTGAGTACCCAGAGTAAA
AACTAATTTACAGAACTTCCTTATTGATCTGCTGGTTCTTCCAGATCATATTCTGGCTATTGGTATGGCTGGCCTTTCTGAAGGTACCCTGCTTGTCTATTT
TCCTGACTCAGCTCTTGCCTGCCTTTTTCACATGTTGCTGCAATTAGACTCACCGTGAGGACTACAGTCAATTTCAGTCTATCTTGTGCCCAATACAACA
AGGATTTTTAATAGTAACAACCCACACCTCACCCACTAGGACTCAATGTTCACAACAGGAAGGACCATTGCTGCATACTCCTTGACCAGCAACTTTTTT
GAAGATATTTTTAAGTGCAGAGTAGGCCTCTATTCCTGTATGTAATTGTTCATTTTCAGCACCTGGAACCTCATCTATCGGGTCTGGAAGGAATACAGCA
GTTCGAAAGCCGCGTCCATTTCTCTCCTTCAGTAGTGCAGAAATGAGTCCGATTCACCAGTACACACAGAACTGTACCAGTTCAACCTAGCAAAAGAA
GAAAAGTTTCCACTGTACTTAAAATTTACAGCTGACTCAAATTGCCTCACAGAATTATTTGATGTAGAAGGCTAGTTGTCTTACTTCAGATCAGCAGGA
CAGTTGGGCTCTCAGACTCATGACCACTGAGTTTGCTTGTGTTGAAACTGTGGTTTCATCCAACATATGCTATTGGACATGATTATTATTCCATTCAAATG
GATTACAGACTTCTTGAGGACAGGACAAACTTATCTCTCATGGTGTTTTTTTAGAATACTTTTATAACCAAGGAAGAAACCATGCCAGCTGTTACCATTC
AACTTCTTAAGCAGAGATTAAGCTTTTTCATATCTGTTCTTATCCTGGACATCAGTAGTTTTTAATTGCCCAGCATCCGTTCCATCTTGTAACAACTCCCT
GATGTTTCTTAAAACCACCTCTTCCTATTTTCAGTCTGTGGTTTGGACAGTCTGACCCAACCTTGAGCTTTGTGGGTGAACATGTAATTCAGACCTCATC

AATCAGCAAATCCATCTGAACTGTGGAGGAGAAGCTCTCTTTACTGAGGGTGCTTTAGCTTTGTAGGATGAAAACCTCAAACTAACAGGGCCTACCAT
GTAGAGAATGAAGCCAGTGCAGGGGAAAGCAGAGCCAAAATATGGAGAGACTTGAATCCTGATGACAGCGTTTGTGCCCCTGGATCCAACCGTGCCT
GAAGCTAGAATATCCCCTGGACTTTTCAGTTATGTGAACCA
>ENST00000569149.2|ENSG00000253352.9|OTTHUMG00000030444.6|OTTHUMT00000495128.1|RP3-430N8.2-015|TUG1|4207|
GTGGACTTTTAGCAAGCGGGCTCACTGGAAGAGACTGAACCTGGCATGGAATTCCTGAAGATGTTTGGGGTTTTTTTCTTTCTTAATCGAAAGTTAACA
TTGTCTGAAAAGTTTTGTTAGAACTACTGCGGAACCTCAAAATCAGTAGATTTGGAAGTGATTCAAAGCTAAACTTTTTCCTTGGCCCTCCTTGTGTTCT
AATTGCTTGCAAGTGTAATACTAGGATGTCCAAGATGCCAGTTTTTGCTTCTTTGTTAGTTGTCAGCTGCTTTTATCAAATTTCAGGCCATTATCCAACAA
ACACTATAAAAATGTTTGAACAATTGGATTTCAAACATTTTCGTTTTGTGGAGTGGTGCTCACCAAGTGGTACAGCCCTAAGCAAGTGAACACAAACA
CATTTAAGTGTATTTTGTCTGATTAGATGTTAGCCAGTTATGCTATTTCATTCAAATGTCTGAAAAAATCAATTGACTATTCCCTTTTCCTAAAGGGCAGA
GACAGATAATCTCACTTCCAGAGAAATGACTTGGAGAAAAAAAAGTGTTGGTCTTTTTGCTCTTTTGTAATTAAATCCGGATGTACCTCAAAAGACTTA
AGACTGTGGTGATAAGATGCTTTCCTCAGCAGAAAGGAGGGAAAAAAAACAACTGGAACTCAAAGCTTGAAATTCTGTGGCAAAACATGAGATGTCC
AGGATTGGAGGTTGAAAAGATTTCACTACAGTGTTCTGCAATAGTTGGAGCAGATAACTTTCAGTGTAGCCACAGCCATGGACTCCAGATTTCCAGATT
TTCAAGACCTGGACCTGGAACCCGAAAGAGCTTGTCACGATGCGGCAGGAACACTGGAGGTAGATTTTTTTTATTTTTGAATTTTGGGACTGTTGACC
TTGCTGTGAGAAAAGAGACAACGACTGAGCAAGCACTACCACCAGCACTGTTACTGGGAATTAGAAGACCTGAGTTTCTGTCCAGACCCTCAGTGCA
AACTGAGGATGCTCCATCCAAAGTGAATTATGTCCTGTGCCTCCTGATTGCTGAGTGTTCACCTGGACCTTCTGACTACCTTCCCTGTGCTATTCCATCA
GCCTACAGACCTGGTACCTGGATTTTTGCCCGAGATGATTCCTACCACCTTACTACTGACGAAGACACCCATTCCAGTGGACCACTGTGACCCAGGAG
GCATTCAGCCATCATGATGTGGCCTTTACCTCCACTCCTGTCTTGTTCTACCCAGATTCAGCACAGCCCTTTATAGTGAAGTCAGAGTCCTCAAGCCAAA
TAGCTAAAGCTGTTTTATCACAACAAAGGCCTAGTTTGTTCCATGAGTGTGCATTTCATTTCTTCAGTTAAAGCCTTCAGAGACACACAATAAATTTGGA
CCAGGGGATTTTTTAGTTATTAATGCTCTCTGAAGAAAGGCAACATCTTTTTGAGAGCAGCATTGGACCACACCCCACAATCTCAAATGATTGAAATTC
ATGAACATCTAGGATCCCGTGAAGGTCACTGGACCCTGTTTTTTCTACTTCAAATCCTGTAGTAGCCTACTGAATGAGAAAACATATTCTGACCCATTGG
GATCAAATCAAAGGCACAGTGAACTCCTCATAGCATCTTCTTTGGAATTACTCAGGAACCAGAACTTTTTACACAAATGTAAGAAATTCTACCAAGGAG
TCCCCTTACCTAACAGCATCTCACAAGGCTGCACCAGATTCCAGAAAAGGCTTCTCTTGATACATCAAGGTAGAACCTCTATGGTAAATCCCTCTGTTTA
TAATGCACTTTACAGTTTCAAAGCACTTGTACGTACATTTATCTCAATTATCAAAATAACAGTGAATTATAATCCCTTTTTGGAAATGAGGGAATGTAAAT
TTTAAAAGTTAAGCAATTATCCAAGGTGACACAGCTGTGAAAGTGGTAGAGGCCAGGCTTTCTGACACCCAGTCCAGTACTCTCCTTGGAATTAGCCT
CCATTTTACCCACAGTGTTAAAATGGCTGGGTATTGGGAACCTCAGGAAATTTTCCAGCTTCCTGATCTGGTGCCCCTAGTCACATAGGATGTGTGATCA
CGTGATAGCTACATCTGGACCAGAATCCCATGGCAATATCCCACGGTTTAAGGGCATAATATCTCCGTGGTGGTAGATTGTCAAGTCAAAAACGGGACA

TGTTCTAACAGCAAAGCTACCCAATATGAAACACAAAGCTACTTTTAGATTTTTTAAGACTTACTGATATTCCTGGGCCCTAAGAGCTTGTCTATGGTCC
TATCTTTGCAAACTAGTATTTACACAAAATGAGCCACCTGGTTTTTTTAAAGTCAGTATTGTGTGTGTGTGTGTAAATATGACATGGAGAAAGCTTCTGT
TACCTAGTAAGATAGTAAAGTATGTAAGACATGGCCCTTCCCCCAGTCTACATTGAGGTATAGTGTATTAAAGGATCTCAGGAGACTTGCAGCAAATTAC
TACTGCTTCTGTGCTTAAATTCAGAAGTCTGAAAAAAAAAAAAAAAAGACTTCTAGTAAAGAGTTCCATTTTGCTGTTTAACCCTTTGTGTCCTAAGTTT
AATCAAACACAGAAGGATATTTTTTTGCATCATTCCATCTAATGGAATGCAGAAACTGCTCTAGAGCAGTATAACCAGTGCTTCCTGATTGCCAGCAGAT
TGCCCTGGACACCCCAGAATAAACAGCATCTTCAAAATCCCCCTTTCTCATACCTTCTTATTAATGGTTGAATTAGTATGTCTCTGTCATCTGAGAGTCTT
GCATCTTGACCATTACCTTTATTTTCTGTCAAGGGAATCCCTAGAAGATGTATGGGGAGACATAGGAAAGGAATGGAAGAAAGTTAATTCACCCCAAAT
TGGATAAATTCCATGACATTTAGCAATATACTTCTGTATCCCTAGAATTGTATCATAACTTTAGTTCAAGGACATTGCATCTCAGGTGGGTTAAGGAGGTT
GGGAAGGGGAGAAAAGGGATGAGGAGATATTTTCTTGAGATTTATCTCCTTGAGATAATGCTTGAGAAGTCATCCAGCCATGGACTTCTTGGAGTAATA
CTTACAAAGAATTGGATGCATTTTGCATCCTTAGGCAAAAACCCCAGTGCTGTTACAACTGAAAATCATTAGCCTTGTTCTCTGATAGATACTTACAAGC
CCAACTGCAGGCAATCAAAATTTATAGCAGATGAGTTCTAACTCCATCAACCTGTAATGTGGTCCATCTTAGGGAAAGAATAGGAAAACACCTTGGTTT
ACCACAGGCTTTGCTTACAGTTTCTGCTTTCTGTGACCTATTTTTTGTATTTCAGCATTTTGTGACCGACTTATTCTTAGATCATTGGTTTTCCAAAGGCTT
TGTGGCCATGAAGCCCTTTGAGTGAAAACTGTGCAGAAGCCCAGAGTAAAAGTGAAGCTGCTCTGGATGAAGTAGTGAAGCAAGAGTAGGGGCCTG
AATCCTGCTACAACTATCTTCCTTTACCACCGTGGTGACACCTAAGGGGACTTCCTTACAACACCTTGAACTCTTCCGAACACAGTTTGAAAACCACTG
CCCCAGACAGCAATATGTTTGACCTGAATGGCATTCCAATCTTTTCTGTACCTCCACTCAGCACAGTTCATGTTCAGTAGATGCTGAACATTCTTAGAAA
TACTGTGTGTGAACTTAGAAAAGTGCAAGAAGACAGGCATGTCTTTGACCCCAGGAATGATCATTTGCTGAAGATGGTGTCAAGTGAACCTAGATTAA
CAGCCCTCCACTCCAGATGGATATCCAGTGATTCCTAGAATGGGATATAGCCAGAGAACAATTCTATGCACCCTACACTGACAGACTCCCTTAAGCAAC
ACCAGATGCTCTACTGGTACTTGAAGTACATGACTTTGAAGTCTTGACCCTCCATGAATACCTGAATTATCAGCAAGCGGGTTTTGAAGCTGGTGCCTC
ATTGAGGCCATATTAGAGCAACTTGTACATTTGACCTCTTGTTATCAGCCATGGTACTCTACTTCGTGTGCAAGAGATAACTATGAAAGCCAAATTCAAA
TACTGGCAACATTTCCTAAAGGGGCTCAATATCTATCATTCGTCTTCTTTTCCAAACTACACATCACTGTATGACTCAACCAGTAGCAGTTATATTGCCCC
TTGGTTTTTATTCAGTTTAACTACTGTTTCC
>ENST00000563812.2|ENSG00000253352.9|OTTHUMG00000030444.6|OTTHUMT00000495129.1|RP3-430N8.2-019|TUG1|2787|
TAAAAATGTTTGAACAATTGGATTTCAAACATTTTCGTTTTGTGGAGTGGTGCTCACCAAGTGGTACAGCCCTAAGCAAGTGAACACAAACACATTTAA
GTGTATTTTGTCTGATTAGATGTTAGCCAGTTATGCTATTTCATTCAAATGTCTGAAAAAATCAATTGACTATTCCCTTTTCCTAAAGGGCAGAGACAGAT
AATCTCACTTCCAGAGAAATGACTTGGAGAAAAAAAAAGTGTTGGTCTTTTTGCTCTTTTGTAATTAAATCCGGATGTACCTCAAAAGACTTAAGACTGT
GGTGATAAGATGCTTTCCTCAGCAGAAAGGAGGGAAAAAAAACAACTGGAACTCAAAGCTTGAAATTCTGTGGCAAAACATGAGATGTCCAGGATTG

GAGGTTGAAAAGATTTCACTACAGTGTTCTGCAATAGTTGGAGCAGATAACTTTCAGTGTAGCCACAGCCATGGACTCCAGATTTCCAGATTTTCAAGA
CCTGGACCTGGAACCCGAAAGAGCTTGTCACGATGCGGCAGGAACACTGGAGGTAGATTTTTTTTATTTTTGAATTTTGGGACTGTTGACCTTGCTGT
GAGAAAAGAGACAACGACTGAGCAAGCACTACCACCAGCACTGTTACTGGGAATTAGAAGACCTGAGTTTCTGTCCAGACCCTCAGTGCAAACTGA
GGATGCTCCATCCAAAGTGAATTATGGTACTTGCCATTTTCCAAAATGCCTTATCCTTTACCATCTCTGCACTTTTGTTCATACTCTCATTCTACTTTGGAA
CTGCTGCTCTGTGGCTTTTCATCTGTCAAAACTGCCATTTTCTCAGTATCCAACTCTTATGCCCTCTTTTCCATGAGTCTCCTAACTAGCCAGAATAGAGC
TTTAAAGTTTTATGACATTTCGTTATGTATCCTCTATCTGTATACAAAATCCTGTAAAATAGTTACTTGCCTGCATTTACTGTCTTTGCAGATAGCAGACTC
CTTGAAAGCAGGGTCCTTGTTTAGTGCATCTTTGCCCACATACACCACAACATATCAAGATGCATTTATTAGGAAGGAGGAGTTTAGAGAGCAGGCTAT
CAGAATAACCACTCATCCTGTGCCTCCTGATTGCTGAGTGTTCACCTGGACCTTCTGACTACCTTCCCTGTGCTATTCCATCAGCCTACAGACCTGGTAC
CTGGATTTTTGCCCGAGATGATTCCTACCACCTTACTACTGACGAAGACACCCATTCCAGTGGACCACTGTGACCCAGGAGGCATTCAGCCATCATGAT
GTGGCCTTTACCTCCACTCCTGTCTTGTTCTACCCAGATTCAGCACAGCCCTTTATAGTGAAGTCAGAGTCCTCAAGCCAAATAGCTAAAGCTGTTTTAT
CACAACAAAGGCCTAGTTTGTTCCATGAGTGTGCATTTCATTTCTTCAGTTAAAGCCTTCAGAGACACACAATAAATTTGGACCAGGGGATTTTTTAGTT
ATTAATGCTCTCTGAAGAAAGGCAACATCTTTTTGAGAGCAGCATTGGACCACACCCCACAATCTCAAATGATTGAAATTCATGAACATCTAGGATCCC
GTGAAGGTCACTGGACCCTGTTTTTTCTACTTCAAATCCTGTAGTAGCCTACTGAATGAGAAAACATATTCTGACCCATTGGGATCAAATCAAAGGCAC
AGTGAACTCCTCATAGCATCTTCTTTGGAATTACTCAGGAACCAGAACTTTTTACACAAATGTAAGAAATTCTACCAAGGAGTCCCCTTACCTAACAGC
ATCTCACAAGGCTGCACCAGATTCCAGAAAAGGCTTCTCTTGATACATCAAGCATTTTGTGACCGACTTATTCTTAGATCATTGGTTTTCCAAAGGCTTT
GTGGCCATGAAGCCCTTTGAGTGAAAACTGTGCAGAAGCCCAGAGTAAAAGTGAAGCTGCTCTGGATGAAGTAGTGAAGCAAGAGTAGGGGCCTGA
ATCCTGCTACAACTATCTTCCTTTACCACCGTGGTGACACCTAAGGGGACTTCCTTACAACACCTTGAACTCTTCCGAACACAGTTTGAAAACCACTGC
CCCAGACAGCAATATGTTTGACCTGAATGGCATTCCAATCTTTTCTGTACCTCCACTCAGCACAGTTCATGTTCAGTAGATGCTGAACATTCTTAGAAAT
ACTGTGTGTGAACTTAGAAAAGTGCAAGAAGACAGGCATGTCTTTGACCCCAGGAATGATCATTTGCTGAAGATGGTGTCAAGTGAACCTAGATTAAC
AGCCCTCCACTCCAGATGGATATCCAGTGATTCCTAGAATGGGATATAGCCAGAGAACAATTCTATGCACCCTACACTGACAGACTCCCTTAAGCAACA
CCAGATGCTCTACTGGTACTTGAAGTACATGACTTTGAAGTCTTGACCCTCCATGAATACCTGAATTATCAGCAAGCGGGTTTTGAAGCTGGTGCCTCAT
TGAGGCCATATTAGAGCAACTTGTACATTTGACCTCTTGTTATCAGCCATGGTACTCTACTTCGTGTGCAAGAGATAACTATGAAAGCCAAATTCAAATA
CTGGCAACATTTCCTAAAGGGGCTCAATATCTATCATTCGTCTTCTTTTCCAAACTACACATCACTGTATGACTCAACCAGTAGCAGTTATATTGCCCCTT
GGTTTTTATTCAGTTTAACTACTGTTTCCAAGATAAATGAGCTAATAAGCTTTAAAAAAAAAAAAAAAAAAAGGCTGAATTCTTTTTTCTTCATCACTGGC
ATAT
>ENST00000566220.2|ENSG00000253352.9|OTTHUMG00000030444.6|OTTHUMT00000431953.2|RP3-430N8.2-005|TUG1|932|

GCTATTTCATTCAAATGTCTGAAAAAATCAATTGACTATTCCCTTTTCCTAAAGGGCAGAGACAGATAATCTCACTTCCAGAGAAATGACTTGGAGAAA
AAAAAGTGTTGGTCTTTTTGCTCTTTTGTAATTAAATCCGGATGTACCTCAAAAGACTTAAGACTGTGGTGATAAGATGCTTTCCTCAGCAGAAAGGAG
GGAAAAAAAACAACTGGAACTCAAAGCTTGAAATTCTGTGGCAAAACATGAGATGTCCAGGATTGGAGGTTGAAAAGATTTCACTACAGTGTTCTGC
AATAGTTGGAGCAGATAACTTTCAGTGTAGCCACAGCCATGGACTCCAGATTTCCAGATTTTCAAGACCTGGACCTGGAACCCGAAAGAGCTTGTCAC
GATGCGGCAGGAACACTGGAGTCCTGTGCCTCCTGATTGCTGAGTGTTCACCTGGACCTTCTGACTACCTTCCCTGTGCTATTCCATCAGCCTACAGAC
CTGGTACCTGGATTTTTGCCCGAGATGATTCCTACCACCTTACTACTGACGAAGACACCCATTCCAGTGGACCACTGTGACCCAGGAGGCATTCAGCCA
TCATGATGTGGCCTTTACCTCCACTCCTGTCTTGTTCTACCCAGATTCAGCACAGCCCTTTATAGTGAAGTCAGAGTCCTCAAGCCAAATAGCTAAAGCT
GTTTTATCACAACAAAGGCCTAGTTTGTTCCATGAGTGTGCATTTCATTTCTTCAGTTAAAGCCTTCAGAGACACACAATAAATTTGGACCAGGGGATTT
TTTAGTTATTAATGCTCTCTGAAGAAAGGCAACATCTTTTTGAGAGCAGCATTGGACCACACCCCACAATCTCAAATGATTGAAATTCATGAACATCTAG
GATCCCGTGAAGGTCACTGGACCCTGTTTTTTCTACTTCAA
>ENST00000521091.6|ENSG00000253352.9|OTTHUMG00000030444.6|OTTHUMT00000158310.3|RP3-430N8.2-002|TUG1|2110|
ATAAGATGCTTTCCTCAGCAGAAAGGAGGGAAAAAAAACAACTGGAACTCAAAGCTTGAAATTCTGTGGCAAAACATGAGATGTCCAGGATTGGAGG
TTGAAAAGATTTCACTACAGTGTTCTGCAATAGTTGGAGCAGATAACTTTCAGTGTAGCCACAGCCATGGACTCCAGATTTCCAGATTTTCAAGACCTG
GACCTGGAACCCGAAAGAGCTTGTCACGATGCGGCAGGAACACTGGAGGTAGATTTTTTTTTATTTTTGAATTTTGGGACTGTTGACCTTGCTGTGAGA
AAAGAGACAACGACTGAGCAAGCACTACCACCAGCACTGTTACTGGGAATTAGAAGACCTGAGTTTCTGTCCAGACCCTCAGTGCAAACTGAGGATG
CTCCATCCAAAGTGAATTATGATAGCAGACTCCTTGAAAGCAGGGTCCTTGTTTAGTGCATCTTTGCCCACATACACCACAACATATCAAGATGCATTTA
TTAGGAAGGAGGAGTTTAGAGAGCAGGCTATCAGAATAACCACTCACCTACAGACCTGGTACCTGGATTTTTGCCCGAGATGATTCCTACCACCTTACT
ACTGACGAAGACACCCATTCCAGTGGACCACTGTGACCCAGGAGGCATTCAGCCATCATGATGTGGCCTTTACCTCCACTCCTGTCTTGTTCTACCCAG
ATTCAGCACAGCCCTTTATAGTGAAGTCAGAGTCCTCAAGCCAAATAGCTAAAGCTGTTTTATCACAACAAAGGCCTAGTTTGTTCCATGAGTGTGCAT
TTCATTTCTTCAGTTAAAGCCTTCAGAGACACACAATAAATTTGGACCAGGGGATTTTTTAGTTATTAATGCTCTCTGAAGAAAGGCAACATCTTTTTGA
GAGCAGCATTGGACCACACCCCACAATCTCAAATGATTGAAATTCATGAACATCTAGGATCCCGTGAAGGTCACTGGACCCTGTTTTTTCTACTTCAAA
TCCTGTAGTAGCCTACTGAATGAGAAAACATATTCTGACCCATTGGGATCAAATCAAAGGCACAGTGAACTCCTCATAGCATCTTCTTTGGAATTACTCA
GGAACCAGAACTTTTTACACAAATGTAAGAAATTCTACCAAGGAGTCCCCTTACCTAACAGCATCTCACAAGGCTGCACCAGATTCCAGAAAAGGCTT
CTCTTGATACATCAAGGTAGAACCTCTATGCATTTTGTGACCGACTTATTCTTAGATCATTGGTTTTCCAAAGGCTTTGTGGCCATGAAGCCCTTTGAGT
GAAAACTGTGCAGAAGCCCAGAGTAAAAGTGAAGCTGCTCTGGATGAAGTAGTGAAGCAAGAGTAGGGGCCTGAATCCTGCTACAACTATCTTCCTTT
ACCACCGTGGTGACACCTAAGGGGACTTCCTTACAACACCTTGAACTCTTCCGAACACAGTTTGAAAACCACTGCCCCAGACAGCAATATGTTTGACC

TGAATGGCATTCCAATCTTTTCTGTACCTCCACTCAGCACAGTTCATGTTCAGTAGATGCTGAACATTCTTAGAAATACTGTGTGTGAACTTAGAAAAGT
GCAAGAAGACAGGCATGTCTTTGACCCCAGGAATGATCATTTGCTGAAGATGGTGTCAAGTGAACCTAGATTAACAGCCCTCCACTCCAGATGGATATC
CAGTGATTCCTAGAATGGGATATAGCCAGAGAACAATTCTATGCACCCTACACTGACAGACTCCCTTAAGCAACACCAGATGCTCTACTGGTACTTGAA
GTACATGACTTTGAAGTCTTGACCCTCCATGAATACCTGAATTATCAGCAAGCGGGTTTTGAAGCTGGTGCCTCATTGAGGCCATATTAGAGCAACTTGT
ACATTTGACCTCTTGTTATCAGCCATGGTACTCTACTTCGTGTGCAAGAGATAACTATGAAAGCCAAATTCAAATACTGGCAACATTTCCTAAAGGGGCT
CAATATCTATCATTCGTCTTCTTTTCCAAACTACACATCACTGTATGACTCAACCAGTAGCAGTTATATTGCCCCTTGGTTTTTATTCAGTTTAACTACTGT
TTCCAAGATAAATGAGCTAATAAGCTTT
>ENST00000643077.1|ENSG00000253352.9|OTTHUMG00000030444.6|OTTHUMT00000431952.2|RP3-430N8.2-004|TUG1|4752|
AACATGAGATGTCCAGGATTGGAGGTTGAAAAGATTTCACTACAGTGTTCTGCAATAGTTGGAGCAGATAACTTTCAGTGTAGCCACAGCCATGGACT
CCAGATTTCCAGATTTTCAAGACCTGGACCTGGAACCCGAAAGAGCTTGTCACGATGCGGCAGGAACACTGGAGGTAGATTTTTTTTTATTTTTGAATT
TTGGGACTGTTGACCTTGCTGTGAGAAAAGAGACAACGACTGAGCAAGCACTACCACCAGCACTGTTACTGGGAATTAGAAGACCTGAGTTTCTGTC
CAGACCCTCAGTGCAAACTGAGGATGCTCCATCCAAAGTGAATTATGATAGCAGACTCCTTGAAAGCAGGGTCCTTGTTTAGTGCATCTTTGCCCACAT
ACACCACAACATATCAAGATGCATTTATTAGGAAGGAGGAGTTTAGAGAGCAGGCTATCAGAATAACCACTCATCCTGTGCCTCCTGATTGCTGAGTGT
TCACCTGGACCTTCTGACTACCTTCCCTGTGCTATTCCATCAGCCTACAGACCTGGTACCTGGATTTTTGCCCGAGATGATTCCTACCACCTTACTACTG
ACGAAGACACCCATTCCAGTGGACCACTGTGACCCAGGAGGCATTCAGCCATCATGATGTGGCCTTTACCTCCACTCCTGTCTTGTTCTACCCAGATTC
AGCACAGCCCTTTATAGTGAAGTCAGAGTCCTCAAGCCAAATAGCTAAAGCTGTTTTATCACAACAAAGGCCTAGTTTGTTCCATGAGTGTGCATTTCA
TTTCTTCAGTTAAAGCCTTCAGAGACACACAATAAATTTGGACCAGGGGATTTTTTAGTTATTAATGCTCTCTGAAGAAAGGCAACATCTTTTTGAGAG
CAGCATTGGACCACACCCCACAATCTCAAATGATTGAAATTCATGAACATCTAGGATCCCGTGAAGGTCACTGGACCCTGTTTTTTCTACTTCAAATCCT
GTAGTAGCCTACTGAATGAGAAAACATATTCTGACCCATTGGGATCAAATCAAAGGCACAGTGAACTCCTCATAGCATCTTCTTTGGAATTACTCAGGA
ACCAGAACTTTTTACACAAATGTAAGAAATTCTACCAAGGAGTCCCCTTACCTAACAGCATCTCACAAGGCTGCACCAGATTCCAGAAAAGGCTTCTC
TTGATACATCAAGCATTTTGTGACCGACTTATTCTTAGATCATTGGTTTTCCAAAGGCTTTGTGGCCATGAAGCCCTTTGAGTGAAAACTGTGCAGAAG
CCCAGAGTAAAAGTGAAGCTGCTCTGGATGAAGTAGTGAAGCAAGAGTAGGGGCCTGAATCCTGCTACAACTATCTTCCTTTACCACCGTGGTGACAC
CTAAGGGGACTTCCTTACAACACCTTGAACTCTTCCGAACACAGTTTGAAAACCACTGCCCCAGACAGCAATATGTTTGACCTGAATGGCATTCCAATC
TTTTCTGTACCTCCACTCAGCACAGTTCATGTTCAGTAGATGCTGAACATTCTTAGAAATACTGTGTGTGAACTTAGAAAAGTGCAAGAAGACAGGCAT
GTCTTTGACCCCAGGAATGATCATTTGCTGAAGATGGTGTCAAGTGAACCTAGATTAACAGCCCTCCACTCCAGATGGATATCCAGTGATTCCTAGAAT
GGGATATAGCCAGAGAACAATTCTATGCACCCTACACTGACAGACTCCCTTAAGCAACACCAGATGCTCTACTGGTACTTGAAGTACATGACTTTGAAG

TCTTGACCCTCCATGAATACCTGAATTATCAGCAAGCGGGTTTTGAAGCTGGTGCCTCATTGAGGCCATATTAGAGCAACTTGTACATTTGACCTCTTGT
TATCAGCCATGGTACTCTACTTCGTGTGCAAGAGATAACTATGAAAGCCAAATTCAAATACTGGCAACATTTCCTAAAGGGGCTCAATATCTATCATTCG
TCTTCTTTTCCAAACTACACATCACTGTATGACTCAACCAGTAGCAGTTATATTGCCCCTTGGTTTTTATTCAGTTTAACTACTGTTTCCAAGATAAATGA
GCTAATAAGCTTTAAAAAAAAAAAAAAAAAAAAGGCTGAATTCTTTTTTCTTCATCACTGGCATATCTGCCTATTCTCCAGAATTATTATGACTATTCAGCTC
ACTTTAACAGTTGAACTTCAAGCGACAATCTTTGAACACCCCTTCTCATGTGATTTAAAATGAAACCATTTGGAAAAGTTTCTTCTAGCCAGTAATAGAT
TTTTTTTTTAATTGCTCTGCCTTGTGCCGAGAGATGTTCTTTTAAGATGAATCTTTTGATGTCTGATACCACCAAATATAGGTGGTAGGGAGAGTTGGAG
GCTGGCCCTTTGAGCAGGCCATTAGCTTACTTGCTGGGCATTTCCGATAGCTTATTGCCTACCTTTTTGCTGGAAACAAACTGATTTGAAAAACAAAATC
TATGAAGACTGCAGCTAAGGATTTTATCGGTAGACTTAAGAGCTTTTGTCCTTGTGGATATTTTAGTGGAACCACATCAGTCTCAATACTGTCATTTTACA
CTGACTCAGAGCAGCTGACTTCATTCCTTGCCATGATATATATTTAAGGCAGGCATTGTAACAGACATAAAGACAACTTATCTGTTTCAGCAGGAAGGAT
TCAGTTTATGAACTCTCAGACCAGATCATGTTGAACAAGGAGACTTTGATGTGTGTCATGAGAAAACTCATTCTTTACTTCCCAGTCAATTTAAAGGCC
AGCTATCCTGAGCTACTCGAATGAATGCACTGGTTAAACATTGGAAATAGTTTGTTTATATCCTTGTCTCTCTCTAGGCCAATTGTGATTACATGACTCGA
CTCTACATCTCGTCAAACAAGGCCTAGGTCTGGTTGCTGTAGACTGCTCGCCCTCAACAAATAAAATCTGGTTGACTAGCCTCCTTGTATATACAACTAT
TATTTGTTAAGAAGAAATTATCGTCAATTTTCTACTACCTTCCAATTGTCAGCTCTTTTTTTCCTCTCTGGTTTTTCCTATACTTTACAGAAAAAGACATTG
ATCTATACTGCCATTCCCTCTAATCCTGCCATACTCAGTCAAAAGGAATGACTTAAGATGAAGATGATCATCTGCTCGAGTCTAAAATATACATTGTATATA
AGAATTGGTGATTAGAAAAGCAAAAAACCTAAAACTTAAATCTAGGAGTCTGTATACTGTCTCCATGTCTCCATGCCTCAGATCTCATCTAAATCTTTGA
ACAGCACCATTCAACCAATCTGAGGCCTTGACTTGCTTGTAAGATGATTCTCAGAGATCGGCTGAGTTAAAAAAGATGACGACTTGATTACCAAAGAA
AGTAGGGCCAACTTTGACAAATCTGGCTCTGCTGACCCTGTCACTCCCAGATGTAGCATAGACTCCTAAACAGAACCTCAAGTCTGATTGAGGATAAG
GCCTTCTCCTGAGCTGAAAGTTCTTTGGCAGATGAGCAAGAAACTGAAAGCTGATGTACCTGACTGGCTCTGTAAGATCAGAAAACTGTATCCAGAAT
AAGCCCTATGGATTAACCCCTGAGTACCCAGAGTAAAAACTAATTTACAGAACTTCCTTATTGATCTGCTGGTTCTTCCAGATCATATTCTGGCTATTGGT
ATGGCTGGCCTTTCTGAAGGTACCCTGCTTGTCTATTTTCCTGACTCAGCTCTTGCCTGCCTTTTTCACATGTTGCTGCAATTAGACTCACCGTGAGGAC
TACAGTCAATTTCAGTCTATCTTGTGCCCAATACAACAAGGATTTTTAATAGTAACAACCCACACCTCACCCACTAGGACTCAATGTTCACAACAGGAA
GGACCATTGCTGCATACTCCTTGACCAGCAACTTTTTTGAAGATATTTTTAAGTGCAGAGTAGGCCTCTATTCCTGTATGTAATTGTTCATTTTCAGCACC
TGGAACCTCATCTATCGGGTCTGGAAGGAATACAGCAGTTCGAAAGCCGCGTCCATTTCTCTCCTTCAGTAGTGCAGAAATGAGTCCGATTCACCAGTA
CACACAGAACTGTACCAGTTCAACCTAGCAAAAGAAGAAAAGTTTCCACTGTACTTAAAATTTACAGCTGACTCAAATTGCCTCACAGAATTATTTGAT
GTAGAAGGCTAGTTGTCTTACTTCAGATCAGCAGGACAGTTGGGCTCTCAGACTCATGACCACTGAGTTTGCTTGTGTTGAAACTGTGGTTTCATCCAA
CATATGCTATTGGACATGATTATTATTCCATTCAAATGGATTACAGACTTCTTGAGGACAGGACAAACTTATCTCTCATGGTGTTTTTTTAGAATACTTTTA

TAACCAAGGAAGAAACCATGCCAGCTGTTACCATTCAACTTCTTAAGCAGAGATTAAGCTTTTTCATATCTGTTCTTATCCTGGACATCAGTAGTTTTTA
ATTGCCCAGCATCCGTTCCATCTTGTAACAACTCCCTGATGTTTCTTAAAACCACCTCTTCCTATTTTCAGTCTGTGGTTTGGACAGTCTGACCCAACCT
TGAGCTTTGTGGGTGAACATGTAATTCAGACCTCATCAATCAGCAAATCCATCTGAACTGTGGAGGAGAAGCTCTCTTTACTGAGGGTGCTTTAGCTTT
GTAGGATGAAAACCTCAAACTAACAGGGCCTACCATGTAGAGAATGAAGCCAGTGCAGGGGAAAGCAGAGCCA
>ENST00000602393.1|ENSG00000253352.9|OTTHUMG00000030444.6|OTTHUMT00000467406.1|RP3-430N8.2-008|TUG1|2233|
GTTTTTTAATTCTAAATTTTGATCTAGGGAGCCCTCAGACATAAGGAGAAACCAATATGTTGAATGATGGGGTATTACATACAGAAGGACCAAGACCTTA
TTTTCTCCTACTTAAGATGGAATCTTGTTAAGATGAAAGGACAGCTTTTTAAAAGGAGAAGGTCATAAGACAGTTTGAGGAAGGCATTGGAAGAGGAA
GAGGGGCAATGTCTCCTTTGTTTTATTGTTGGTATATAAACTTAAAATCTCAGTTCTTTTTATGGCACTTGTGGAGCCACTTCCTCTCCTCACCAAAAAAT
GCCCAGTACCCTGAATCCGATCAAATTACTCTCCTAAAGTATAAGGCTTAGTTTCTGTGTGCTGCTTCGCGAGACAGTTCCCACAAATCGAGAGTGTTA
ATCAGACTTTTGTGTTTTCTTTTTTGGATTGCTGTTGTTTGTTTTCATTTATCATTTGTCTTTGCCTAAGCCAGGCTCATCAAGAATTAACAGCCATCAGG
CTGCCGATGTGCTGACAGCAGACCCACTTAGAGTCCTGTGTTTGTAATTCCATGCATTTGTTATTTTACCTGTTTTGTCCCTGCCCCTTCTAGTCCTGTGC
CTCCTGATTGCTGAGTGTTCACCTGGACCTTCTGACTACCTTCCCTGTGCTATTCCATCAGCCTACAGACCTGGTACCTGGATTTTTGCCCGAGATGATT
CCTACCACCTTACTACTGACGAAGACACCCATTCCAGTGGACCACTGTGACCCAGGAGGCATTCAGCCATCATGATGTGGCCTTTACCTCCACTCCTGT
CTTGTTCTACCCAGATTCAGCACAGCCCTTTATAGTGAAGTCAGAGTCCTCAAGCCAAATAGCTAAAGCTGTTTTATCACAACAAAGGCCTAGTTTGTT
CCATGAGTGTGCATTTCATTTCTTCAGTTAAAGCCTTCAGAGACACACAATAAATTTGGACCAGGGGATTTTTTAGTTATTAATGCTCTCTGAAGAAAGG
CAACATCTTTTTGAGAGCAGCATTGGACCACACCCCACAATCTCAAATGATTGAAATTCATGAACATCTAGGATCCCGTGAAGGTCACTGGACCCTGTT
TTTTCTACTTCAAATCCTGTAGTAGCCTACTGAATGAGAAAACATATTCTGACCCATTGGGATCAAATCAAAGGCACAGTGAACTCCTCATAGCATCTTC
TTTGGAATTACTCAGGAACCAGAACTTTTTACACAAATGTAAGAAATTCTACCAAGGAGTCCCCTTACCTAACAGCATCTCACAAGGCTGCACCAGATT
CCAGAAAAGGCTTCTCTTGATACATCAAGGTAGAACCTCTATGCATTTTGTGACCGACTTATTCTTAGATCATTGGTTTTCCAAAGGCTTTGTGGCCATG
AAGCCCTTTGAGTGAAAACTGTGCAGAAGCCCAGAGTAAAAGTGAAGCTGCTCTGGATGAAGTAGTGAAGCAAGAGTAGGGGCCTGAATCCTGCTAC
AACTATCTTCCTTTACCACCGTGGTGACACCTAAGGGGACTTCCTTACAACACCTTGAACTCTTCCGAACACAGTTTGAAAACCACTGCCCCAGACAG
CAATATGTTTGACCTGAATGGCATTCCAATCTTTTCTGTACCTCCACTCAGCACAGTTCATGTTCAGTAGATGCTGAACATTCTTAGAAATACTGTGTGTG
AACTTAGAAAAGTGCAAGAAGACAGGCATGTCTTTGACCCCAGGAATGATCATTTGCTGAAGATGGTGTCAAGTGAACCTAGATTAACAGCCCTCCAC
TCCAGATGGATATCCAGTGATTCCTAGAATGGGATATAGCCAGAGAACAATTCTATGCACCCTACACTGACAGACTCCCTTAAGCAACACCAGATGCTCT
ACTGGTACTTGAAGTACATGACTTTGAAGTCTTGACCCTCCATGAATACCTGAATTATCAGCAAGCGGGTTTTGAAGCTGGTGCCTCATTGAGGCCATAT
TAGAGCAACTTGTACATTTGACCTCTTGTTATCAGCCATGGTACTCTACTTCGTGTGCAAGAGATAACTATGAAAGCCAAATTCAAATACTGGCAACATT

TCCTAAAGGGGCTCAATATCTATCATTCGTCTTCTTTTCCAAACTACACATCACTGTATGACTCAACCAGTAGCAGTTATATTGCCCCTTGGTTTTTATTCA
GTTTAACTACTGTTTCCAAGATAAATGAGCTAATAAGCTTT
>SNHG12
>ENST00000464612.5|ENSG00000197989.13|OTTHUMG00000003654.2|OTTHUMT00000010362.1|RP4-669K10.5-007|SNHG12|719|
GGTCAAAAAGGAGCCCAGAGTGACAGTTTTCCTTGACGGTCGCCGTTCTGTTTGTTGTAACTGATCTGCAACATTTTGGGAAAATACAGTTCCATTGTA
CCTGCCACCTTTCAGCTGTAGCCAGAGACCTTTATCAAAATGGGCTTGTTTCCACAGAACAGCAGAACGGTTGGGGGTTTGCATGTACAGTTAACCAG
CATAAGACTCGTACTGGGAAAATCCAGCTGGGATGTGACACAGCTTAACTGATAGCTTAAGGCATCAGTATTGGGACCAAAGGCTGGTCAGATTTGTAT
CATTCTGAGGACCAAATGATGGGAACAATAAAATTGTTCATGACAGTTGTTCTCATTTTGCTGTCCAGATGAAGACTCTTAAGATGACAGAAGGTGATT
TTTCTGGTGATCGAGGACTTCCGGGGTAATGACAGTGATGAAATGCAGGGGACCTGGTTGCCCCCAAGTTTCCTGGCAGTGTGTGATACTGAGGAGGT
GAGCTTGTTTCTGGAGCTGTGCTTTAAGATTCATGTTACATGTAAAGCTGTCCTCATTTGTGACTATGGACCTATGGAGTTGGGACAATCTCTATGGGAA
GCAGAAGGCAAGGACCCCGGTCATTTTAGGTAGAAACAACAGCATGCTAATGCAAAAAATTATGCAGTGTGCTACTGAACTTCAGAGGTGATCAATAA
AAGAAGAATAAAAAGACTAATAAAAGTA
>ENST00000475441.5|ENSG00000197989.13|OTTHUMG00000003654.2|OTTHUMT00000010366.1|RP4-669K10.5-011|SNHG12|444|
GCGGATAAAACGGTCCCATCAAGACTGAGAAAAAGCACACCAGCTATTGGCACAGCGTGGGCAGTGGGGCCTACAGGATGACTGACTTAGTCTACAG
AGATCCCGGCGTACTTAAGCAGATGAAGACTCTTAAGATGACAGAAGGTGATTTTTCTGGTGATCGAGGACTTCCGGGGTAATGACAGTGATGAAATG
CAGGGGACCTGGTTGCCCCCAAGTTTCCTGGCAGTGTGTGATACTGAGGAGATTCATGTTACATGTAAAGCTGTCCTCATTTGTGACTATGGACCTATG
GAGTTGGGACAATCTCTATGGGAAGCAGAAGGCAAGGACCCCGGTCATTTTAGGTAGAAACAACAGCATGCTAATGCAAAAAATTATGCAGTGTGCTA
CTGAACTTCAGAGGTGATCAATAAAAGAAGAATAAAAAGACTAATAAAAGTA
>ENST00000531126.5|ENSG00000197989.13|OTTHUMG00000003654.2|OTTHUMT00000010356.1|RP4-669K10.5-001|SNHG12|719|
TGCAAGCCTCTGCCTGCCTTCCTGCGCGCCGTTCCCCGCTAGTCGCTGCTGCTGGCGCGCACTCGCCGGGTTTTTCCTCCCACGGCCTCGAGATGGTG
GTGAATGTGGCACGGAGGAGCCGGGCCTTCCAACCCGGTGGGCCCGAGCTCCGAAAGGCCCCCTCGGCAGTGAGAGGGGCGGGAGCCCGCGGGGG
CCGCGCCCTTCTCTCGCTTCGGACTGCGCAACGCTGCGCTCTGGGCTGACAGGCGGATAAAACGGTCCCATCAAGACTGAGAAAAAGCACACCAGCT
ATTGGCACAGCGTGGGCAGTGGGGCCTACAGGATGACTGACTTAGTCTACAGAGATCCCGGCGTACTTAAGCAGATGAAGACTCTTAAGATGACAGAA
GGTGATTTTTCTGGTGATCGAGGACTTCCGGGGTAATGACAGTGATGAAATGCAGGGGACCTGGTTGCCCCCAAGTTTCCTGGCAGTGTGTGATACTG
AGGAGGTGAGCTTGTTTCTGGAGCTGTGCTTTAAGATTCATGTTACATGTAAAGCTGTCCTCATTTGTGACTATGGACCTATGGAGTTGGGACAATCTCT
ATGGGAAGCAGAAGGCAAGGACCCCGGTCATTTTAGGTAGAAACAACAGCATGCTAATGCAAAAAATTATGCAGTGTGCTACTGAACTTCAGAGGTGA

TCAATAAAAGAAGAATAAAAAGACTAATAAAAGTA
>ENST00000461448.5|ENSG00000197989.13|OTTHUMG00000003654.2|OTTHUMT00000092197.1|RP4-669K10.5-012|SNHG12|1629|
TTACTAGCTGCAAGCCTCTGCCTGCCTTCCTGCGCGCCGTTCCCCGCTAGTCGCTGCTGCTGGCGCGCACTCGCCGGGTTTTTCCTCCCACGGCCTCGA
GATGGTGGTGAATGTGGCACGGAGGAGCCGGGCCTTCCAACCCGGTGGGCCCGAGCTCCGAAAGGCCCCCTCGGCAGTGAGAGGGGCGGGAGCCCG
CGGGGGCCGCGCCCTTCTCTCGCTTCGGACTGCGCAACGCTGCGCTCTGGGCTGACAGGCGGATAAAACGGTCCCATCAAGACTGAGAAAAAGCACA
CCAGCTATTGGCACAGCGTGGGCAGTGGGGCCTACAGGATGACTGACTTAGTCTACAGAGATCCCGGCGTACTTAAGCAGATGAAGACTCTTAAGATG
ACAGAAGGTGATTTTTCTGGTGATCGAGGACTTCCGGGGTAATGACAGTGATGAAATGCAGGGGACCTGGTAGGTTTCTTTTGGAGACTAACTGGGGG
CTGCCTGGTGGGACAGAACAGCATGTTTCCAAGGGCTGTGGCTGGTCATAGCCATGGGATCTCCAACTGCATGCAAGAGCAACCTGGAAAGACTTTG
ACAGCGCAGGTCAGTACAATACCTGCAAGCTGCCACTCAGCTTTCCTATAATGTTTCAGGACCAGGGCTGGAAGCCTTCCATGTGTCAACTTCAATGTA
GATGGGTTTTTTTTTTTTTTTTTTGAGACGGAGTCTTGCTCTGTCACCCAGGCTGCTGGAGTGCAGTGGCATGATCTCGGCTCATGGCAACCTCTGC
CTCCTGGGTTCAAGCGACTCTCCTACCTCATCCTCCCGAGTAGCTGGGATTACAGGTGCCCACCATCACACCCAGCTAATTTTCTTGTATTTTTAGTAGA
GATGGTTTTCACCATGTTGGCTAGATGGGTCTCGAACTTCTGACCTCGGGTGATCCGCCCGACTCGGCCTCCCCTTCCAAAGTGCTGGGATTACAGGCA
TGAGCCACCGCTCCTGGCCAGTTTAGTTTTCACGCTAAGCTTTTTGTTCTGCAGGTTGCCCCCAAGTTTCCTGGCAGTGTGTGATACTGAGGAGGTGAG
CTTGTTTCTGGAGCTGTGCTTTAAGGTAAAGTTGATCAGCTTAATCCTCCTGATCCCTTTCCCATCGGATCTGAACACTGGTCTTGGTGGTCGTAAAAGG
AGGAAAAGTAATAGTGAAGCTGGCCTAAATGTTGTAATCTGGTATATGGCATGTGGGCTAGTTTCAGACAGGTTTCAGAGATGGTTGGATCTCTGAAAT
TGTAAAATGAAGTATAATCTTAGGCTAAGGGAAGGATGCGTGTGAAGCTCTGGAGGTTGGTATAGTAATAGCTGACCTATTACTGCATTTGGGAGGGAT
CTGTCATAGCTTCCTTGCCTCTTAATTAAGGGTGGTGTTTTTTTCTTTTAGATTCATGTTACATGTAAAGCTGTCCTCATTTGTGACTATGGACCTATGGAG
TTGGGACAATCTCTATGGGAAGCAGAAGGCAAGGACCCCGGTCATTTTAGGTAGAAACAACAGCATGCTAATGCAAAAAATTATGCAGTGTGCTACTG
AACTTCAGAGGTGATCAATAAAAGAAGAATAAAAAGACTAATAAAAGTA
>ENST00000470977.5|ENSG00000197989.13|OTTHUMG00000003654.2|OTTHUMT00000010358.1|RP4-669K10.5-003|SNHG12|1098|
CTTTCTCCCCGCCGCATTCCCGGTGTCGACTTACTAGCTGCAAGCCTCTGCCTGCCTTCCTGCGCGCCGTTCCCCGCTAGTCGCTGCTGCTGGCGCGCA
CTCGCCGGGTTTTTCCTCCCACGGCCTCGAGATGGTGGTGAATGTGGCACGGAGGAGCCGGGCCTTCCAACCCGGTGGGCCCGAGCTCCGAAAGGCC
CCCTCGGCAGTGAGAGGGGCGGGAGCCCGCGGGGGCCGCGCCCTTCTCTCGCTTCGGACTGCGCAACGCTGCGCTCTGGGCTGACAGGCGGATAAA
ACGGTCCCATCAAGACTGAGAAAAAGCACACCAGCTATTGGCACAGCGTGGGCAGTGGGGCCTACAGGATGACTGACTTAGTCTACAGAGATCCCGG
CGTACTTAAGCAGGTAGTAATGATGGACAGATGAAGACTCTTAAGATGACAGAAGGTGATTTTTCTGGTGATCGAGGACTTCCGGGGTAATGACAGTG
ATGAAATGCAGGGGACCTGGTTGCCCCCAAGTTTCCTGGCAGTGTGTGATACTGAGGAGGTGAGCTTGTTTCTGGAGCTGTGCTTTAAGGTAAAGTTG

ATCAGCTTAATCCTCCTGATCCCTTTCCCATCGGATCTGAACACTGGTCTTGGTGGTCGTAAAAGGAGGAAAAGTAATAGTGAAGCTGGCCTAAATGTT
GTAATCTGGTATATGGCATGTGGGCTAGTTTCAGACAGGTTTCAGAGATGGTTGGATCTCTGAAATTGTAAAATGAAGTATAATCTTAGGCTAAGGGAAG
GATGCGTGTGAAGCTCTGGAGGTTGGTATAGTAATAGCTGACCTATTACTGCATTTGGGAGGGATCTGTCATAGCTTCCTTGCCTCTTAATTAAGGGTGG
TGTTTTTTTCTTTTAGATTCATGTTACATGTAAAGCTGTCCTCATTTGTGACTATGGACCTATGGAGTTGGGACAATCTCTATGGGAAGCAGAAGGCAAG
GACCCCGGTCATTTTAGGTAGAAACAACAGCATGCTAATGCAAAAAATTATGCAGTGTGCTACTGAACTTCAGAGGTGATCAATAAAAGAAGAATAAA
AAGACTAATAAAAGTA
>ENST00000474814.1|ENSG00000197989.13|OTTHUMG00000003654.2|OTTHUMT00000010359.1|RP4-669K10.5-004|SNHG12|1903|
TTTCTCCCCGCCGCATTCCCGGTGTCGACTTACTAGCTGCAAGCCTCTGCCTGCCTTCCTGCGCGCCGTTCCCCGCTAGTCGCTGCTGCTGGCGCGCAC
TCGCCGGGTTTTTCCTCCCACGGCCTCGAGATGGTGGTGAATGTGGCACGGAGGAGCCGGGCCTTCCAACCCGGTGGGCCCGAGCTCCGAAAGGCCC
CCTCGGCAGTGAGAGGGGCGGGAGCCCGCGGGGGCCGCGCCCTTCTCTCGCTTCGGACTGCGCAACGCTGCGCTCTGGGCTGACAGGTGAGTGTTGC
AGGCAGGCGGCCGGGTTTACGGAAGGGGTGGGGGTTTGGGAACCGGTCTCCTGGGGGATGCGGGGATGAATCCCTGGGCCTGAAGTGTCTTAATTCA
GGGGGAGGCCGCATGCTGGTGTCAGGATGGCCTCCAGTTGAATCTGTGACATTTACCAGCTAGGTGATTTTGGGACTGGTGACTACGCGCGAGACCTC
TGGAGGTCGCCCTGGTAGACACGGTTTTCAGAAGGACGGGTTTTAGGCATTCAGACCCTTGCGTGCATCCCAGCTTGTTGCTTTCAGCCTCGCTAAGT
GACTAAGCGACCTTCAGGAAGTAACTTAATCGGTCTAAACCTCTTACCTGTAATAGGCGGATAAAACGGTCCCATCAAGACTGAGAAAAAGCACACCA
GCTATTGGCACAGCGTGGGCAGTGGGGCCTACAGGATGACTGACTTAGTCTACAGAGATCCCGGCGTACTTAAGCAGGTAGTAATGATGGACAGGTGA
GGCTTTAGGCGGCCGCCCTGGGATCTGAATTGCCCCTTGGCCCTTATCGAAGCTGCAGCTGCTTCCGCATAGCTGCTGTGGTCAAAAAGGAGCCCAGA
GTGACAGTTTTCCTTGACGGTCGCCGTTCTGTTTGTTGTAACTGATCTGCAACATTTTGGGAAAATACAGTTCCATTGTACCTGCCACCTTTCAGCTGTA
GCCAGAGACCTTTATCAAAATGGGCTTGTTTCCACAGAACAGCAGAACGGTTGGGGGTTTGCATGTACAGTTAACCAGCATAAGACTCGTACTGGGAA
AATCCAGCTGGGATGTGACACAGCTTAACTGATAGCTTAAGGCATCAGTATTGGGACCAAAGGCTGGTCAGATTTGTATCATTCTGAGGACCAAATGAT
GGGAACAATAAAATTGTTCATGACAGTTGTTCTCATTTTGCTGTCCAGATGAAGACTCTTAAGATGACAGAAGGTGATTTTTCTGGTGATCGAGGACTT
CCGGGGTAATGACAGTGATGAAATGCAGGGGACCTGGTTGCCCCCAAGTTTCCTGGCAGTGTGTGATACTGAGGAGGTGAGCTTGTTTCTGGAGCTGT
GCTTTAAGGTAAAGTTGATCAGCTTAATCCTCCTGATCCCTTTCCCATCGGATCTGAACACTGGTCTTGGTGGTCGTAAAAGGAGGAAAAGTAATAGTG
AAGCTGGCCTAAATGTTGTAATCTGGTATATGGCATGTGGGCTAGTTTCAGACAGGTTTCAGAGATGGTTGGATCTCTGAAATTGTAAAATGAAGTATAA
TCTTAGGCTAAGGGAAGGATGCGTGTGAAGCTCTGGAGGTTGGTATAGTAATAGCTGACCTATTACTGCATTTGGGAGGGATCTGTCATAGCTTCCTTGC
CTCTTAATTAAGGGTGGTGTTTTTTTCTTTTAGATTCATGTTACATGTAAAGCTGTCCTCATTTGTGACTATGGACCTATGGAGTTGGGACAATCTCTATG
GGAAGCAGAAGGCAAGGACCCCGGTCATTTTAGGTAGAAACAACAGCATGCTAATGCAAAAAATTATGCAGTGTGCTACTGAACTTCAGAGGTGATC

AATAAAAGAAGAATAAAAAGACTAATAAAAGT
>ENST00000481368.5|ENSG00000197989.13|OTTHUMG00000003654.2|OTTHUMT00000010360.1|RP4-669K10.5-005|SNHG12|1588|
GAGGGGCGGGAGCCCGCGGGGGCCGCGCCCTTCTCTCGCTTCGGACTGCGCAACGCTGCGCTCTGGGCTGACAGGCGGATAAAACGGTCCCATCAAG
ACTGAGAAAAAGCACACCAGCTATTGGCACAGCGTGGGCAGTGGGGCCTACAGGATGACTGACTTAGTCTACAGAGATCCCGGCGTACTTAAGCAGG
TAGTAATGATGGACAGGTGAGGCTTTAGGCGGCCGCCCTGGGATCTGAATTGCCCCTTGGCCCTTATCGAAGCTGCAGCTGCTTCCGCATAGCTGCTGT
GGTCAAAAAGGAGCCCAGAGTGACAGTTTTCCTTGACGGTCGCCGTTCTGTTTGTTGTAACTGATCTGCAACATTTTGGGAAAATACAGTTCCATTGTA
CCTGCCACCTTTCAGCTGTAGCCAGAGACCTTTATCAAAATGGGCTTGTTTCCACAGAACAGCAGAACGGTTGGGGGTTTGCATGTACAGTTAACCAG
CATAAGACTCGTACTGGGAAAATCCAGCTGGGATGTGACACAGCTTAACTGATAGCTTAAGGCATCAGTATTGGGACCAAAGGCTGGTCAGATTTGTAT
CATTCTGAGGACCAAATGATGGGAACAATAAAATTGTTCATGACAGTTGTTCTCATTTTGCTGTCCAGATGAAGACTCTTAAGATGACAGAAGGTGATT
TTTCTGGTGATCGAGGACTTCCGGGGTAATGACAGTGATGAAATGCAGGGGACCTGGTAGGTTTCTTTTGGAGACTAACTGGGGGCTGCCTGGTGGGA
CAGAACAGCATGTTTCCAAGGGCTGTGGCTGGTCATAGCCATGGGATCTCCAACTGCATGCAAGAGCAACCTGGAAAGACTTTGACAGCGCAGGTCA
GTACAATACCTGCAAGCTGCCACTCAGCTTTCCTATAATGTTTCAGGACCAGGGCTGGAAGCCTTCCATGTGTCAACTTCAATGTAGATGGGTTTTTTTT
TTTTTTTTTTTTTGAGACGGAGTCTTGCTCTGTCACCCAGGCTGCTGGAGTGCAGTGGCATGATCTCGGCTCATGGCAACCTCTGCCTCCTGGGTTCAA
GCGACTCTCCTACCTCATCCTCCCGAGTAGCTGGGATTACAGGTGCCCACCATCACACCCAGCTAATTTTCTTGTATTTTTAGTAGAGATGGTTTTCACC
ATGTTGGCTAGATGGGTCTCGAACTTCTGACCTCGGGTGATCCGCCCGACTCGGCCTCCCCTTCCAAAGTGCTGGGATTACAGGCATGAGCCACCGCTC
CTGGCCAGTTTAGTTTTCACGCTAAGCTTTTTGTTCTGCAGGTTGCCCCCAAGTTTCCTGGCAGTGTGTGATACTGAGGAGGTGAGCTTGTTTCTGGAG
CTGTGCTTTAAGATTCATGTTACATGTAAAGCTGTCCTCATTTGTGACTATGGACCTATGGAGTTGGGACAATCTCTATGGGAAGCAGAAGGCAAGGAC
CCCGGTCATTTTAGGTAGAAACAACAGCATGCTAATGCAAAAAATTATGCAGTGTGCTACTGAACTTCAGAGGTGATCAATAAAAGAAGAATAAAAAG
ACTAATAAAAG
>ENST00000481220.5|ENSG00000197989.13|OTTHUMG00000003654.2|OTTHUMT00000010361.1|RP4-669K10.5-006|SNHG12|751|
CCGGTGTCGACTTACTAGCTGCAAGCCTCTGCCTGCCTTCCTGCGCGCCGTTCCCCGCTAGTCGCTGCTGCTGGCGCGCACTCGCCGGGTTTTTCCTCC
CACGGCCTCGAGATGGTGGTGAATGTGGCACGGAGGAGCCGGGCCTTCCAACCCGGTGGGCCCGAGCTCCGAAAGGCCCCCTCGGCAGTGAGAGGG
GCGGGAGCCCGCGGGGGCCGCGCCCTTCTCTCGCTTCGGACTGCGCAACGCTGCGCTCTGGGCTGACAGATGAAGACTCTTAAGATGACAGAAGGTG
ATTTTTCTGGTGATCGAGGACTTCCGGGGTAATGACAGTGATGAAATGCAGGGGACCTGGTAGGTTTCTTTTGGAGACTAACTGGGGGCTGCCTGGTG
GGACAGAACAGCATGTTTCCAAGGGCTGTGGCTGGTCATAGCCATGGGATCTCCAACTGCATGCAAGAGCAACCTGGAAAGACTTTGACAGCGCAGG
TTGCCCCCAAGTTTCCTGGCAGTGTGTGATACTGAGGAGGTGAGCTTGTTTCTGGAGCTGTGCTTTAAGATTCATGTTACATGTAAAGCTGTCCTCATTT

GTGACTATGGACCTATGGAGTTGGGACAATCTCTATGGGAAGCAGAAGGCAAGGACCCCGGTCATTTTAGGTAGAAACAACAGCATGCTAATGCAAAA
AATTATGCAGTGTGCTACTGAACTTCAGAGGTGATCAATAAAAGAAGAATAAAAAGACTAATAAAA
>ENST00000488745.5|ENSG00000197989.13|OTTHUMG00000003654.2|OTTHUMT00000010357.1|RP4-669K10.5-002|SNHG12|1365|
CTGCCTCAGCCTCCCGGGTAGCTGGGACTACAGGCGCCCGCGACCACGCCCGGCTAATTTTTTTTTGTATTTTTTTTTAGTAGAGACCGGGTTTCGCCGT
GTTAGCCAGGATGGTCTCGATCTCCTGACCTCGTGATCCACCCGCCTTGGCCTCCCAAAGTGCTGGGATTACAGGCGTGAGTAACCGCACCCGGCAGT
AGTACCTACTTTTTAAAGTGTAAGCAAGGAAAGTTGGCCCGCACCTGTAGTCCCATCTACCTGGGAGGCTGAGGTGGAAGAATTGCTTGAGCCCAGTT
GGTCGAGGCTGCAATGAGCCTTGATCACGCCACTGCACTCCAGCCTGGGCAACAGAACAAGACCCTGTCTCAAACAAAAAAGTGTATGTAAGAATAA
AAATTAATAGATTAGGCACAGGATGGGTGTTTAATTCTCGGCAATCACCACTAAGTTTTCATACTGAGGCTTATTCATTCCATGGGCAAAACATTGCGCT
ATGTGTGGTGCCTGGGGTGGGCTCCAAATTCCAGTCCTCTCCTCGCTTGCGATAGCTGTGTGATCCATTCCTAAGTTTCCTTATTTGTCCACAGAGCGTA
CACAGGGTTTTTATGAAAACTAGATCAGATAATCAGACACAGTTAAAATAGCACGCGCTCAGTAAAATTACATATTAGTGGAAGAGACAAGCCTTGAAT
AAATCGCCTTTCGGGGAGTATGACCCCCTCCCCGAAACCCACTCAGGCGCTGCTCGCTCGCACGCCCACTGAGCTCGGAGCGGAGCCCGAGCCCTTT
CCCAGCACACAGGGTTCATTTCCAGACTGAAAGACATCCCAGAGACAACTCCAGAGGCCAAGGCGGGTCGGCTTCCTGCGTGGGCCCAGCGCCGGG
CACTGAAAGGCGGATAAAACGGTCCCATCAAGACTGAGAAAAAGCACACCAGCTATTGGCACAGCGTGGGCAGTGGGGCCTACAGGATGACTGACTT
AGTCTACAGAGATCCCGGCGTACTTAAGCAGATGAAGACTCTTAAGATGACAGAAGGTGATTTTTCTGGTGATCGAGGACTTCCGGGGTAATGACAGT
GATGAAATGCAGGGGACCTGGTTGCCCCCAAGTTTCCTGGCAGTGTGTGATACTGAGGAGGTGAGCTTGTTTCTGGAGCTGTGCTTTAAGATTCATGTT
ACATGTAAAGCTGTCCTCATTTGTGACTATGGACCTATGGAGTTGGGACAATCTCTATGGGAAGCAGAAGGCAAGGACCCCGGTCATTTTAGGTAGAAA
CAACAGCATGCTAATGCAAAAAATTATGCAGTGTGCTACTGAACTTCAGAGGTGATCAATAAAAGAAGAATAAAAAGACTAATAAAA
>ENST00000483436.5|ENSG00000197989.13|OTTHUMG00000003654.2|OTTHUMT00000010365.2|RP4-669K10.5-010|SNHG12|682|
CCTTCCTGCGCGCCGTTCCCCGCTAGTCGCTGCTGCTGGCGCGCACTCGCCGGGTTTTTCCTCCCACGGCCTCGAGATGGTGGTGAATGTGGCACGGA
GGAGCCGGGCCTTCCAACCCGGTGGGCCCGAGCTCCGAAAGGCCCCCTCGGCAGTGAGAGGGGCGGGAGCCCGCGGGGGCCGCGCCCTTCTCTCGC
TTCGGACTGCGCAACGCTGCGCTCTGGGCTGACAGGCGGATAAAACGGTCCCATCAAGACTGAGAAAAAGCACACCAGCTATTGGCACAGCGTGGGC
AGTGGGGCCTACAGGATGACTGACTTAGTCTACAGAGATCCCGGCGTACTTAAGCAGGTAGTAATGATGGACAGATGAAGACTCTTAAGATGACAGAA
GGTGATTTTTCTGGTGATCGAGGACTTCCGGGGTAATGACAGTGATGAAATGCAGGGGACCTGGTTGCCCCCAAGTTTCCTGGCAGTGTGTGATACTG
AGGAGATTCATGTTACATGTAAAGCTGTCCTCATTTGTGACTATGGACCTATGGAGTTGGGACAATCTCTATGGGAAGCAGAAGGCAAGGACCCCGGTC
ATTTTAGGTAGAAACAACAGCATGCTAATGCAAAAAATTATGCAGTGTGCTACTGAACTTCAGAGGTGATCAATAAAAGAAGAATAAAAAGACTAA
>ENST00000464115.1|ENSG00000197989.13|OTTHUMG00000003654.2|OTTHUMT00000010364.1|RP4-669K10.5-009|SNHG12|712|

CCGGTGTCGACTTACTAGCTGCAAGCCTCTGCCTGCCTTCCTGCGCGCCGTTCCCCGCTAGTCGCTGCTGCTGGCGCGCACTCGCCGGGTTTTTCCTCC
CACGGCCTCGAGATGGTGGTGAATGTGGCACGGAGGAGCCGGGCCTTCCAACCCGGTGGGCCCGAGCTCCGAAAGGCCCCCTCGGCAGTGAGAGGG
GCGGGAGCCCGCGGGGGCCGCGCCCTTCTCTCGCTTCGGACTGCGCAACGCTGCGCTCTGGGCTGACAGGCGGATAAAACGGTCCCATCAAGACTGA
GAAAAAGCACACCAGCTATTGGCACAGCGTGGGCAGTGGGGCCTACAGGATGACTGACTTAGTCTACAGAGATCCCGGCGTACTTAAGCAGGTAGTA
ATGATGGACAGATGAAGACTCTTAAGATGACAGAAGGTGATTTTTCTGGTGATCGAGGACTTCCGGGGTAATGACAGTGATGAAATGCAGGGGACCTG
GTAGGTTTCTTTTGGAGACTAACTGGGGGCTGCCTGGTGGGACAGAACAGCATGTTTCCAAGGGCTGTGGCTGGTCATAGCCATGGGATCTCCAACTG
CATGCAAGAGCAACCTGGAAAGACTTTGACAGCGCAGGTCAGTACAATACCTGCAAGCTGCCACTCAGCTTTCCTATAATGTTTCAGGACCAGGGCTG
GAAGCCTTCCATGTGTCAACTTCAATGTA
>ENST00000461832.1|ENSG00000197989.13|OTTHUMG00000003654.2|OTTHUMT00000010363.1|RP4-669K10.5-008|SNHG12|675|
ATTCCCGGTGTCGACTTACTAGCTGCAAGCCTCTGCCTGCCTTCCTGCGCGCCGTTCCCCGCTAGTCGCTGCTGCTGGCGCGCACTCGCCGGGTTTTTC
CTCCCACGGCCTCGAGATGGTGGTGAATGTGGCACGGAGGAGCCGGGCCTTCCAACCCGGTGGGCCCGAGCTCCGAAAGGCCCCCTCGGCAGTGAG
AGGGGCGGGAGCCCGCGGGGGCCGCGCCCTTCTCTCGCTTCGGACTGCGCAACGCTGCGCTCTGGGCTGACAGGCGGATAAAACGGTCCCATCAAGA
CTGAGAAAAAGCACACCAGCTATTGGCACAGCGTGGGCAGTGGGGCCTACAGGATGACTGACTTAGTCTACAGAGATCCCGGCGTACTTAAGCAGAT
GAAGACTCTTAAGATGACAGAAGGTGATTTTTCTGGTGATCGAGGACTTCCGGGGTAATGACAGTGATGAAATGCAGGGGACCTGGTAGGTTTCTTTT
GGAGACTAACTGGGGGCTGCCTGGTGGGACAGAACAGCATGTTTCCAAGGGCTGTGGCTGGTCATAGCCATGGGATCTCCAACTGCATGCAAGAGCA
ACCTGGAAAGACTTTGACAGCGCAGGTCAGTACAATACCTGCAAGCTGCCACTCAGCTTTCCTATAATGTTTCAGGACCAGGGCTGGAAGC
>NEAT1
>ENST00000499732.3|ENSG00000245532.8|OTTHUMG00000166321.7|OTTHUMT00000389141.3|RP11-867O8.8-002|NEAT1|3441|
GCAAAAGTTGTGGCAAGTCCAGCCGGAGTTAGCGACAGGGAGGGATGCGCGCCTGGGTGTAGTTGTGGGGGAGGAAGTGGCTAGCTCAGGGCTTCA
GGGGACAGACAGGGAGAGATGACTGAGTTAGATGAGACGAGGGGGCGGGCTGGGGGTGCGAGAAGGAAGCTTGGCAAGGAGACTAGGTCTAGGGG
GACCACAGTGGGGCAGGCTGCATGGAAAATATCCGCAGGGTCCCCCAGGCAGAACAGCCACGCTCCAGGCCAGGCTGTCCCTACTGCCTGGTGGAGG
GGGAACTTGACCTCTGGGAGGGCGCCGCTCTTGCATAGCTGAGCGAGCCCGGGTGCGCTGGTCTGTGTGGAAGGAGGAAGGCAGGGAGAGGTAGAA
GGGGTGGAGGAGTCAGGAGGAATAGGCCGCAGCAGCCCTGGAAATGATCAGGAAGGCAGGCAGTGGGTGCAGGGCTGCAGGAGGGCCGGGAGGGC
TAATCTTCAACTTGTCCATGCCAGCAGCCCCTTTTTTTCCAGACCAAGGGCTGTGAACCCGCCTGGGGATGAGGCCTGGTCTTGTGGAACTGAACTTA
GCTCGACGGGGCTGACCGCTCTGGCCCAGGGTGGTGGCAGTGCTCCTTTTGGACTTTTCTCTAGGTTTGGCGCTAAACTCTTCTTGTGAGCTCACTCCA
CCCCTTCTTCCTCCCTTTAACTTATCCATTCACTTAAAACATTACCTGGTCATCTGGTAAGCCCGGGACAGTAAGCCGAGTGGCTGTTGGAGTCGGTATT

GTTGGTAATGGTGGAGGAAGAGAGGCCTTCCCGCTGAGGCTGGGGTGGGGCGGATCGGTGTTGCTTGCCTGCAGAGAGGGTGGGGAGTGAATGTGC
ACCCTTGGGTGGGCCTGCAGCCATCCAGCTGAAAGTTACAAAAATGCTTCATGGACCGTGGTTTGTTACTATAGTGTTCCTCATGGCGAGCAGATGGAA
CCGGGAGACATGGAGTCCCTGGCCAGTGTGAGTCCTAGCATTGCAGGAGGGGAGACCCTGGAGGAGAGAGCCCGCCTCAATTGATGCCTGCAGATTG
AATTTCCAGAGGCTTAGGAGGAGGAAGTTCTCCAATGTTCTGTTTCCAGGCCTTGCTCAGGAAGCCCTGTATTCAGGAGGCTACCATTTAAAGTTTGCA
GATGAGCTTATGGGGGGCAATCTTAAAAAGTCCACAGCAGATGCATCCGGCTCGAGGGGCCATCAGCTTTGAATAAATGCTTGTTCCAGAGCCCATGA
ATGCCAGCAGGCACCCCTCCTTTCCTGGGGTAAAGGTTTTCAGATGCTGCATCTTCTAAATTGAGCCTCCGGTCATACTAGTTTTGTGCTTGGAACCTTG
CTTCAAGAAGATCCCTAAGCTGTAGAACATTTTAACGTTGATGCCACAACGCAGATTGATGCCTTGTAGATGGAGCTTGCAGATGGAGCCCCGTGACCT
CTCACCTACCCACCTGTTTGCCTGCCTTCTTGTGCGTTTCTCGGAGAAGTTCTTAGCCTGATGAAATAACTTGGGGCGTTGAAGAGCTGTTTAATTTTAA
ATGCCTTAGACTGGGGATATATTAGAGGAAGCAGATTGTCAAATTAAGGGTGTCATTGTGTTGTGCTAAACGCTGGGAGGGTACAAGTTGGTCATTCCT
AAATCTGTGTGTGAGAAATGGCAGGTCTAGTTTGGGCATTGTGATTGCATTGCAGATTACTAGGAGAAGGGAATGGTGGGTACACCGGTAGTGCTCTTT
TGTTCTTGCTTCGTTTTTTTAAACTTGAACTTTACTTCGTTAGATTTCATAATACTTTCTTGGCATTCTAGTAAGAGGACCCTGAGGTGGGAGTTGTGGGG
GACGGGGAGAAGGGGACAGCTTGGCACCGGTCCCGTGGGCGTTGCAGTGTGGGGGATGGGGGTATGCAGCTTGGCACTGGTACTGGGAGGGATGAG
GGTGAAGAAGGGGAGAGGGTTGGTTAGAGATACAGTGTGGGTGGTGGGGGTGGTAGGAAATGCAGGTTGAAGGGAATTCTCTGGGGCTTTGGGGAA
TTTAGTGCGTGGGTGAGCCAAGAAAATACTAATTAATAATAGTAAGTTGTTAGTGTTGGTTAAGTTGTTGCTTGGAAGTGAGAAGTTGCTTAGAAACTT
TCCAAAGTGCTTAGAACTTTAAGTGCAAACAGACAAACTAACAAACAAAAATTGTTTTGCTTTGCTACAAGGTGGGGAAGACTGAAGAAGTGTTAAC
TGAAAACAGGTGACACAGAGTCACCAGTTTTCCGAGAACCAAAGGGAGGGGTGTGTGATGCCATCTCACAGGCAGGGGAAATGTCTTTACCAGCTTC
CTCCTGGTGGCCAAGACAGCCTGTTTCAGAGGGTTGTTTTGTTTGGGGTGTGGGTGTTATCAAGTGAATTAGTCACTTGAAAGATGGGCGTCAGACTT
GCATACGCAGCAGATCAGCATCCTTCGCTGCCCCTTAGCAACTTAGGTGGTTGATTTGAAACTGTGAAGGTGTGATTTTTTCAGGAGCTGGAAGTCTTA
GAAAAGCCTTGTAAATGCCTATATTGTGGGCTTTTAACGTATTTAAGGGACCACTTAAGACGAGATTAGATGGGCTCTTCTGGATTTGTTCCTCATTTGTC
ACAGGTGTCTTGTGATTGAAAATCATGAGCGAAGTGAAATTGCATTGAATTTCAAGGGAATTTAGTATGTAAATCGTGCCTTAGAAACACATCTGTTGT
CTTTTCTGTGTTTGGTCGATATTAATAATGGCAAAATTTTTGCCTATCTAGTATCTTCAAATTGTAGTCTTTGTAACAACCAAATAACCTTTTGTGGTCACT
GTAAAATTAATATTTGGTAGACAGAATCCATGTACCTTTGCTAAGGTTAGAATGAATAATTTATTGTATTTTTAATTTGAATGTTTGTGCTTTTTAAATGAG
CCAAGACTAGAGGGGAAACTATCACCTAAAATCAGTTTGGAAAACAAGACCTAAAAAGGGAAGGGGATGGGGATTGTGGGGAGAGAGTGGGCGAGG
TGCCTTTACTACATGTGTGATCTGAAAACCCTGCTTGGTTCTGAGCTGCGTCTATTGAATTGGTAAAGTAATACCAATGGCTTTTTATCATTTCCTTCTTC
CCTTTAAGTTTCACTTGAAATTTTAAAAATCATGGTTATTTTTATCGTTGGGATCTTTCTGTCTTCTGGGTTCCATTTTTTAAATGTTTAAAAATATGTTGA
CATGGTAGTTCAGTTCTTAACCAATGACTTGGGGATGATGCAAACAATTACTGTCGTTGGGATTTAGAGTGTATTAGTCACGCATGTATGGGGAAGTAGT

CTCGGGTATGCTGTTGTGAAATTGAAACTGTAAAAGTAGATGGTTGAAAGTACTGGTATGTTGCTCTGTATGGTAAGAACTAATTCTGTTACG
>ENST00000501122.2|ENSG00000245532.8|OTTHUMG00000166321.7|OTTHUMT00000389142.3|RP11-867O8.8-001|NEAT1|22743|
GGAGTTAGCGACAGGGAGGGATGCGCGCCTGGGTGTAGTTGTGGGGGAGGAAGTGGCTAGCTCAGGGCTTCAGGGGACAGACAGGGAGAGATGACT
GAGTTAGATGAGACGAGGGGGCGGGCTGGGGGTGCGAGAAGGAAGCTTGGCAAGGAGACTAGGTCTAGGGGGACCACAGTGGGGCAGGCTGCATG
GAAAATATCCGCAGGGTCCCCCAGGCAGAACAGCCACGCTCCAGGCCAGGCTGTCCCTACTGCCTGGTGGAGGGGGAACTTGACCTCTGGGAGGGCG
CCGCTCTTGCATAGCTGAGCGAGCCCGGGTGCGCTGGTCTGTGTGGAAGGAGGAAGGCAGGGAGAGGTAGAAGGGGTGGAGGAGTCAGGAGGAATA
GGCCGCAGCAGCCCTGGAAATGATCAGGAAGGCAGGCAGTGGGTGCAGGGCTGCAGGAGGGCCGGGAGGGCTAATCTTCAACTTGTCCATGCCAGC
AGCCCCTTTTTTTCCAGACCAAGGGCTGTGAACCCGCCTGGGGATGAGGCCTGGTCTTGTGGAACTGAACTTAGCTCGACGGGGCTGACCGCTCTGG
CCCAGGGTGGTATGTAATTTTCGCTCGGCCTGGGACGGGGCCCAGGCCGGGCCCAGCCTGGTGGAGCGTCCAGGTCTGGGTGCGAAGCCAGGCCCCT
GGGCGGAGGTGAGGGGTGGTCTGAGGAGTGATGTGGAGTTAAGGCGCCATCCTCACCGGTGACTGGTGCGGCACCTAGCATGTTTGACAGGCGGGGA
CTGCGAGGCACGCTGCTCGGGTGTTGGGGACAACATTGACCAACGCTTTATTTTCCAGGTGGCAGTGCTCCTTTTGGACTTTTCTCTAGGTTTGGCGCT
AAACTCTTCTTGTGAGCTCACTCCACCCCTTCTTCCTCCCTTTAACTTATCCATTCACTTAAAACATTACCTGGTCATCTGGTAAGCCCGGGACAGTAAG
CCGAGTGGCTGTTGGAGTCGGTATTGTTGGTAATGGTGGAGGAAGAGAGGCCTTCCCGCTGAGGCTGGGGTGGGGCGGATCGGTGTTGCTTGCCTGC
AGAGAGGGTGGGGAGTGAATGTGCACCCTTGGGTGGGCCTGCAGCCATCCAGCTGAAAGTTACAAAAATGCTTCATGGACCGTGGTTTGTTACTATAG
TGTTCCTCATGGCGAGCAGATGGAACCGGGAGACATGGAGTCCCTGGCCAGTGTGAGTCCTAGCATTGCAGGAGGGGAGACCCTGGAGGAGAGAGC
CCGCCTCAATTGATGCCTGCAGATTGAATTTCCAGAGGCTTAGGAGGAGGAAGTTCTCCAATGTTCTGTTTCCAGGCCTTGCTCAGGAAGCCCTGTATT
CAGGAGGCTACCATTTAAAGTTTGCAGATGAGCTTATGGGGGGCAATCTTAAAAAGTCCACAGCAGATGCATCCGGCTCGAGGGGCCATCAGCTTTGA
ATAAATGCTTGTTCCAGAGCCCATGAATGCCAGCAGGCACCCCTCCTTTCCTGGGGTAAAGGTTTTCAGATGCTGCATCTTCTAAATTGAGCCTCCGGT
CATACTAGTTTTGTGCTTGGAACCTTGCTTCAAGAAGATCCCTAAGCTGTAGAACATTTTAACGTTGATGCCACAACGCAGATTGATGCCTTGTAGATGG
AGCTTGCAGATGGAGCCCCGTGACCTCTCACCTACCCACCTGTTTGCCTGCCTTCTTGTGCGTTTCTCGGAGAAGTTCTTAGCCTGATGAAATAACTTG
GGGCGTTGAAGAGCTGTTTAATTTTAAATGCCTTAGACTGGGGATATATTAGAGGAAGCAGATTGTCAAATTAAGGGTGTCATTGTGTTGTGCTAAACG
CTGGGAGGGTACAAGTTGGTCATTCCTAAATCTGTGTGTGAGAAATGGCAGGTCTAGTTTGGGCATTGTGATTGCATTGCAGATTACTAGGAGAAGGGA
ATGGTGGGTACACCGGTAGTGCTCTTTTGTTCTTGCTTCGTTTTTTTAAACTTGAACTTTACTTCGTTAGATTTCATAATACTTTCTTGGCATTCTAGTAAG
AGGACCCTGAGGTGGGAGTTGTGGGGGACGGGGAGAAGGGGACAGCTTGGCACCGGTCCCGTGGGCGTTGCAGTGTGGGGGATGGGGGTATGCAGC
TTGGCACTGGTACTGGGAGGGATGAGGGTGAAGAAGGGGAGAGGGTTGGTTAGAGATACAGTGTGGGTGGTGGGGGTGGTAGGAAATGCAGGTTGA
AGGGAATTCTCTGGGGCTTTGGGGAATTTAGTGCGTGGGTGAGCCAAGAAAATACTAATTAATAATAGTAAGTTGTTAGTGTTGGTTAAGTTGTTGCTT

GGAAGTGAGAAGTTGCTTAGAAACTTTCCAAAGTGCTTAGAACTTTAAGTGCAAACAGACAAACTAACAAACAAAAATTGTTTTGCTTTGCTACAAG
GTGGGGAAGACTGAAGAAGTGTTAACTGAAAACAGGTGACACAGAGTCACCAGTTTTCCGAGAACCAAAGGGAGGGGTGTGTGATGCCATCTCACA
GGCAGGGGAAATGTCTTTACCAGCTTCCTCCTGGTGGCCAAGACAGCCTGTTTCAGAGGGTTGTTTTGTTTGGGGTGTGGGTGTTATCAAGTGAATTA
GTCACTTGAAAGATGGGCGTCAGACTTGCATACGCAGCAGATCAGCATCCTTCGCTGCCCCTTAGCAACTTAGGTGGTTGATTTGAAACTGTGAAGGT
GTGATTTTTTCAGGAGCTGGAAGTCTTAGAAAAGCCTTGTAAATGCCTATATTGTGGGCTTTTAACGTATTTAAGGGACCACTTAAGACGAGATTAGATG
GGCTCTTCTGGATTTGTTCCTCATTTGTCACAGGTGTCTTGTGATTGAAAATCATGAGCGAAGTGAAATTGCATTGAATTTCAAGGGAATTTAGTATGTA
AATCGTGCCTTAGAAACACATCTGTTGTCTTTTCTGTGTTTGGTCGATATTAATAATGGCAAAATTTTTGCCTATCTAGTATCTTCAAATTGTAGTCTTTGT
AACAACCAAATAACCTTTTGTGGTCACTGTAAAATTAATATTTGGTAGACAGAATCCATGTACCTTTGCTAAGGTTAGAATGAATAATTTATTGTATTTTT
AATTTGAATGTTTGTGCTTTTTAAATGAGCCAAGACTAGAGGGGAAACTATCACCTAAAATCAGTTTGGAAAACAAGACCTAAAAAGGGAAGGGGATG
GGGATTGTGGGGAGAGAGTGGGCGAGGTGCCTTTACTACATGTGTGATCTGAAAACCCTGCTTGGTTCTGAGCTGCGTCTATTGAATTGGTAAAGTAAT
ACCAATGGCTTTTTATCATTTCCTTCTTCCCTTTAAGTTTCACTTGAAATTTTAAAAATCATGGTTATTTTTATCGTTGGGATCTTTCTGTCTTCTGGGTTCC
ATTTTTTAAATGTTTAAAAATATGTTGACATGGTAGTTCAGTTCTTAACCAATGACTTGGGGATGATGCAAACAATTACTGTCGTTGGGATTTAGAGTGTA
TTAGTCACGCATGTATGGGGAAGTAGTCTCGGGTATGCTGTTGTGAAATTGAAACTGTAAAAGTAGATGGTTGAAAGTACTGGTATGTTGCTCTGTATG
GTAAGAACTAATTCTGTTACGTCATGTACATAATTACTAATCACTTTTCTTCCCCTTTACAGCACAAATAAAGTTTGAGTTCTAAACTCATTAGAATTGTT
GTATTGCTATGTTACATTTCTCGACCCCTATCACATTGCCTTCATAACGACTTTGGATGTATCTTCATATTGTAGATTTAGGTCTAGATTTGCTAGCTCCAA
GTAATTAAGGCCATGTAGGAGAGCATGGTAACCACAGATAGAACTGGTATTATCCCAAGTGGTCTGCAGACTGCTGAGTGGGGATGGGATCTGCTCTCT
GTTGAGAGTTGGTAATCATTGGTTTGAAATGTGATGAAACCACTCAAGCCAATGAAGGTGGGTGTGTAGGTGGGGAGTACTTTGCCATAATATTTTAAA
ACATTACCTGGTTAGAGTTCTAAGTGGTACTTATTTTTGTTTGGTTAGGGGAAAGCCTGAATAAAAACAGAAATGGACACATAATATGCATATTCCATAG
TCTTTGGGAGGCTGGAATGTGCCTGGGATTTGGGTCTAAGTGTATGCGTAATTCTTACCTCACTAAAGAATTTGCCTTGTTTTTTTCCTTTTGGTGAGTG
ACTAAAACGTCTGGGCTTCCCTGTGTGCGTGCTACAGTAAGCAAGCAGAGGCTGTGCAAAGGTGTGAGCAGGATCACGTGGAATCTGGAGGATACAT
CTTGGCTTGCAAACTGCCTCTGTCTCCTGGGTGGGACTGTTCTGTCCTTGCACTGCTGTTCTGTGTTACCTCTTGGGGTGTAAGGTTTTGCTTACAGGA
GACAAACTTTGGGCGTAGAATGGAAGCCACTGCCAGCCTCTGTGCTGAGAAGGAAGGTGCTTGTTTCAAAGGGAGCAGCAAGGGAGGCTTGTTCTAC
TCACCTGGGCCTGTTTGCCTGAGAAGGGGAGATAAGGGCTGAACTGGGACTAGCCAGGGGGACCAACACAAATGGTGGGGGATCATGACCTGAAGG
ATTCTTTCCTTCCCATGAGCTGCAGGGCTGGTTGCCGTCCTTGCAACTGTGTCTTATTTGCCTGTGCCGTTATATCTTGGTGACCCCTCCACGTGTACACT
ACTGACAAACGGGTGGAGTGCTGGGGAGAAGTCACTGTGCCGCCCACCTAGTAAACCTTCTGTCTGTGCTCATGGCATCTCCAAGATGGGGCACTGCT
GTGTGCAGAATCCAGGGTCCTCTTTCTGCTTGCAACTCCTTTCCCTGGATGCCCCAGAAACAATCCAGGCCTCCTTTCCTATCTTACCCCTTTGCTTTGC

TTTTTACCCCAGCACCTCTATAACCGCCTTCTCTTCTTTTCAGAACTCCTTGTTTCTCGTCCTGTTTTTTATGATTACAAAACTCTTGCTTCCACCCTGGA
AGATAACTGCTATAGATGCCTGTATGTAAATGGTGCTGTCTCCAGCAACTGGCATGCTGAAGAAGAATTGATTCACGGGGTATAAATGTTGGGGATTGG
AAGTGGGGATGAAATGGCACTTGTTGATACAGGAGCAGAGAGGTGAGGCCGACTGCTGAAGACAGCTCGCCACCCTCCTTGCCTCCACTCCAATCCA
GGGGCTGGGGCCACATTCTTTGCCTTCATTTATCCTCAGATCAGGTGAGATCGACAGGAGGTGTTGATGGCAGTGCCAGCAATTATTGCTAATCCGTTT
GCATCCTTATGCATAGATCTGAATTCAGACTTTGTGAATTTCCAGAGGTGTGGGTAATATAATAGAATTCAGTGAGTGGGCATGGCTGATCTTGTGCAAA
TTAAAAGTTATGGGGCATAAGAATAGCAAAAGTTGAACTTCTTTTAAAAAGGAAAGTACCCTGAGAGCCAGTATTGGTTGAGGCTCTTCAGTATGCCCA
GGTTGGCAGCACTGAGAACCGCAGGAACGGCCTGTTGTTACAAAAAGGAGATTGACTCAGCTGCCCTTGGTGCATCTGACTGACTATGACTGCTGAG
AGATTCCAAGGACCCTTAATGCCAGGGCTAACCTCTCCATGTGCAGTGAGACCTCTGGAGGAAGTGTCATCCTCTGGCTTTGTGTGGTACTCATTATGG
TGCAGTGCGGGCATGAAATGAAGACACCCAAATAGGCTTACAGATACGATATGTTTTAAATGTTCGTATTTAACAAAAACATACTGACACTGTTTGGAA
ATGGCAACAGGAAGATAGCAAAATGAATACTAACATTACGAAAAGATGAACAGGTACATGTTCCAAGGCAGGTGGCTGTGAACTTCCTCTGAGTGAA
GGCATCCCCTCCAGCACCTTTCAGCCTGCTAGTTAGGACGACCCGCCGCCACCCTCCAGGACCTCCAGCCCTGCACTGCCTTTCCTCTCTTTTAAATAA
TTCTTCATTGAGTTCTAATATGTAAAAAAAAAAAAGTTTACTGTAAAGTTTGCAAATAAGGAAATTTTTTTTAAAAGTCCTCAGTAATCTTACCAGTAACA
ATTGTTATGGGCACATTTGCTTTTGGAAGATTTCTTTTGTATGCATGGGATAAGTACATTTTTAAACAAAAATGGGATTATGCCATAAATTCTATTTTGTGA
CTTTAATATATAGTGAACACCTTTTTTAATGATGACAGGATGTTCCCTTGCATGGCTGTATCAATTTAAACAATCTTGTTTCAATGGGCATACAGGGTATTT
TCTAGTTTTTTTTTCCTCTTAGAAAATAATACTTGCGATGACTTTCCTTGTAGCTCAGACTTTTTCACGTCTGTTGTTATCTCTTTGGGAATGCTGAATACA
TACATTTCGAGAAGGAAATGACTGTTAAACTCTTAAGACTTCAGGTTCATATTGCTAAACTGCCCAGCAGGGAGGGATTTTTTCAATTAGTGTTCTCACT
GGTGAGGCAAACCTGATGCCTTCCCCTCTTCCTCAGAACCGGCTTTATCACATTGAAAACCTTTGCTCCTCCGACGGATCGAGTCTGCTTTCCCTCTGG
ATGTGAGCATTGCTTTGTCTGCTGGTGACTGAACATCTCTACCTTGTGTCAATTGGCCATTTGTGGTGTGTGTGTGTGTGCGTGTGTGTGTGTGTGTGTG
TGTATGATTTTCTAATTCCTAGTCATTTTTCTATTGATTGTTTTGCAAAAGCCATTTACATCTTAAGGATATTGATAATCTTTTGTTATATTTGATGCAAATAT
TTTTTTCCAGTTTATAGGTTGCCTTTTAATTTTGTGTTTCAGGTAGATAAAAGTTAAACGATTTTCTTAGGTTAGTTTATCACTGTGGTTTCTGAACTTGTT
ATGTGTAGATCTTTTCCACCCCAAGAGTACATAAATATTAATCCATACTTTCTTATGGAACTTGTATGGTTTCGTTTTTTACATTTAAACCTTCTTCCCCGT
GGTGTGTGTTGTGGAATCTGTGTTTGTGTGAGGAGGGGCATGGTGCTCTCAGAACCCACCTCCTGTGGCCAGAGAGCCCTGTCCTGTGAGGGTGGTTG
TCACAGTGGCAGGGTTCAATTCAGAAGACCTTGAGGGCAGGCTGATGTTTCCTGAATGGGCCCCTGGTTGTTGCTTGTCCCTGACTCTCCATTTCCCCA
TCTGAGTGGATTTGGACCTAATAGGGCACTGGAGCTGGTTCGAATCCTGACTGGACTACTTGGCAACTTTATGTCTGGGAGCAAGTTACTTAACCTCCC
CAAGCCTGTGTCTGTGAAATGCGGGTAAATGAATGTAGATGTTTGGCAGCAGCTACTCCTTGTTGAGCTCTCACAGTGAACTCTCCTGCCTCTGCCCTC
CTTCCCCGCCTCCCCTGGTGCCTAGCGTCAGGTCTAGCCACTTCCTCCTGGGCCCCTCTCCCTTTTCTGTGGCTGGCTGCCTGCCCGCCTGGCGCTGGA

CCTTTCATGTAACGGGAATCAGCATGTATATTCTGGTCTGGTCTGTTTCTACACTTAATTTTGTTTCCAGTAGTATTTCCCTGTACCGGCAGAGTTCACAA
ACACATTTGAAGAGGCTTTTTCTCAGGATTCTTAACCTTCCCAAAGGAAGTCCCATGGATGGGTTTCTAGAAGTCTATAAATGCTCTGAAATTGTATTTT
TCTGTGGAAAGCATAACTTTCATCTGCTTGTTCGTGCTCAAAAAAGATCATGAATGAATGATTGCATGATTTTATGCCATTGTGCTTATACTAAAGGATAT
GTAGCCCATCTCTTGAGCTGTTAAACTGTTTTGACTACTTTAAATCGTGCAGCTGTGAGCATCTCTGTAAATTTAGTGTACACATGTATCCCCTGGAGTG
GCATTGCCTCGGCAGTGAGCACTTATGGTTTTATAACTCTCTTCACAGACTCAAATGACTCCAGAAAGCTACACTTCCTGTTGTGAGTATATGATATCCA
TTTCCCTACATAGCCACTAACATCAGGTTTTTACAATTTTATTTATTTCTTGCTACTTTAAGAAATTTTTGTGGTGAAATACATATAATAGAAGTTGACTATC
TGAATCATTTTTAAGTATACATTCAGTAGTGTTAAGTATGTCGCCATTGTTGTACAACCAATCTCCAGAACTTTTTCATCTTGCAAAACAAACTCTGTACC
CATTAAATAACATTAAACATTCCATTCCCTCCAGCCTCAGCAACCCCATTCTACTTTCTGTTTCTGTGAGTTTGACTATTCCAAGCACTTCATATCAGTTA
AATCATGAAGTATTTGTCTGTCTGTGACTGGCTTATTTCTCTGAGCACAGTGTCCTCGAGATGCGTCTATGTTGTAGCATATGTCAGAATTTCCTTCCTTT
TTAAAAGATCCAAATAATATTCTTATTTTATATCTTTTTTTTATCCATTCATCCATTAGTGGACACTTGGGTTGCTTTTGGCTATTGTAAATAATGGTGCTAT
GTACAAATATCTATATTATTGTATTTACAAGTATAATGCTGTAATGTACACACATCTTTTTGAGATCCTACCTTCAGTTCTTTTGAGTATATAGCCAGAAGTG
GTATTACTAAATCTTACGATATTTCTATTTTTAATTTATTGAGGAACCACTGTAGTTTTTCATAGCAACTGCACCATTTTACGTTCTCACCAAGAGTGCACA
AGGGTTCCGAGGTTCCCACATCCTCCCCAACACTTGTTATTTTCTGCTTTTTTTAGATTGCAGCCATCATAGTGGGTGTGAGGTGACATTTCATTGTGGT
TTTGATTTGCATTTCCCTAATGAGGAGTGATGCTGAGCATCTTTTCATATGCTTACTGGTCATTTGTATGTTGTCTTTGGAAAAATGTCTATTCAAGTCCTT
TGACTATTTTAAAAATTGGGTTATTAGAGTTATCGTTGTTGTTGACTTGTAGGAGTTTCTTTCTATATTCTGGATATTAATCCCCTATCAGATATATGATTTG
CAAATATCTTCTCTTATTCCATAAGGTTACTTTTTCACTTTGTTGATTGTGTTCTTTGATGTATAGAAGTTTTTAGTTTTGAAATAGTCTAATTTATCTGTTT
TTACTTTTGTGGTCTGTGCTTTTGGTGTCATATCCAAGAAATCCTTGCCAAATCCAACGTTATAAGGTACTTTTAAGGTATTTTAGTTGTCTTAGTCTATAT
TTCTGTACTCACCTTTCTTTATCCACTCATCAGTTGATGGGCATGTAGGTTGGTTCCATATCTTTGCAATTCTGAATTGTGCTATGATCAGGTGTCTTTTTA
GTATAATGATTTACTCTCCTTTGGGTAGATACCCAGTAGTGGGATTGCTGGATCGAATGGTTTTTATAATTTTCTATTTTACCACAGTTTCTCTCTGCATTTT
TCCTCTTTGACCACTAACCATGTGAAATTCTCATATTGACCTTTATAATGATCATGAACTCTTAGTATCATTGGGAAGGCCACATTTGCCACTTATGATTGT
AAACCTTATCCTCCATTTTTCCTGTTATTGTTGGTGCAAAAAGCACCTATTATACCAGGACTTTAAAAATCAGTCTGATAAGTCTTTGATAAGTCTAATAA
TAATAACTGATAAGTCCATTGAATTTGCTTCTGATTACTTTTTCTTTAGTAGCTAAACATGTATGTACTCCTATGATTACAATGAACACTCCTCTCCATTTA
AATTAATTATTTACATTGATGAAATAGCAAAATGTTAATGACTAAATACTGTCTTGGTTTTTTCGTTCCAGGTCAGTCAATATTAACTTCTTATAATTTTCTT
TTTTTTCTTTATGTGTGTGTGTGTGTGTATTTTTTTTTTTTTTAATTTCAATGGCTTTTGGGGTACAAATGGCTTTTGGTCATATAGATGAATTCTACAGTAGT
GAAGTCTGAGATTTTACTGCACCGGTCACCTGAGTAGTGTACATTGTACCCAATATGTGGTTTTTTATACCTTGCCCCCCTCTTACCCTCCCCACTTTGAG
TCTCTAGTGTCCATTATGTCACTCTGTATACCTTTTTGTACCCATAAGTTAGCTCTCACTTATAAGTGAGAACACACAGTATTTGGTTTTCCATTCCTGAGT

TGCTTCACTTAGAATAATATCCTCCAGCTCCATCCAAAATTGCTGCAAAAAAAAAAAAAAACCACAAACATTATTTTGTTCTTTTTTATTGCTAAGTCATAT
TCCATGGTGTAGAGATACCACATTTTATTTATCCACTCACTGGTTGATGGGTTGGTTCCACATCTTTGCAATTGTGACTTGTACTGCCATCAAGTGTCTTT
CTGGTATAATGACTTCTTTTCCTTTGGGTAGATACCCAGGAGTGGGATTGCTAGATCAAATGGTTCTTAACATTTTCTCTCTGGATCTATTTCTGGAAATT
TTAGGCTCCAGTTTTTGTTGTTGTTGTTAATAAAATGCAATGGAATGTAATGATCATCACTTTTCATTATGCTTTAAAATCTGGTAAATGGAGGCTAGAAC
ACTCCTGTAAGGCAAGAATATTCTCTCTGTTGGAACTCAAATACACAGAACTGGGTAAATCTCAATCTTAATCTTTGATTCAGGACACAACATGGCTCTC
TTTTACTTGCTTTCTTTAATTGTTTTTTAATAATGTGGTAAGCATTTCTGAATCTCCTATCCAATACAAAAACTAGGACAATACAGACAGTAACTCCTATGG
TTACAATGAACACTCCTCTCCACTTAAATTAATTATTTACACTGATGAAATTGAAATAGCAAAATTTTAATGACTAAATACTGTCTTTGATTTTTTGTTCCA
GGTCTGTCAATATTAACTTCTTATAATTTTCTTTTTTTTTCTTTATGTGTGTGTGTGTGTGTGTATATATATATATTTAATTTCAATGGCTTTTGGGGTACAAA
TGGCTTTTGGTCATATATATGAGTTCTACAGTAGTGAAGTCTGAGATTTTACTACACCTTCCACTTATGTGGTCCCACACCACCCGCCTCCCCTGCCGCCT
CCTGCCACCCCCTAGGCCAAGGTAATAATCATCCTGAATCCTGGGTTTATCTCTCACTTGCTTTCTTTTCATATAATTTTGCAAAAGAATCTGATCTAAAT
GTGTTTTTCAGAGTATATATTTATATTTTAGCTGTTCTTAGAGAAAATTTATTATTTTGCATGTAATCTTATGGAACATTCTCATTTAATACCATGGTAAGATT
CAGCCCTTGCCCAGGGGATAGTTCATTTAGTTTGTTTACTGGATAGAGCTCATCATGTGACTATACCTCAGTTAGTTTATCAGTTCTCCCATCCATGGTGA
CTAGGTTGCCTCTCAGCCTCTCAACAACACTGTTTCTCAGTGTCCTTGTAGAAGTGATATGTGGGTGTTTTCTCCTTACACAGAGTTGAAAGGTGACGA
CAACAACGTTGGCACTACCAATCCCCCACCCTCCAGAGGGGTAACCAGTGTTACCAGTTTGCTGTGTTTCCTGCTACACCTCGCCTTATTCACTTCCATT
TGTATCTGAAAAACGTGTTGCATGGTTTCTTTTCTATAGAAGTGGTAAAATGCTATTGTGTCCTGTACATTATTGATTACTTTTTTTCATTTAACAGTAGGG
AGATGCCTGGGAGTACACAGAGAACTGCCCTCATTGTTTTCAACTTCTGCACTGTATGTCTGTGAGTTTAGCCATTCTGCTGTTAATGGAAATTTACAGT
ATTCTAATCTTTTGATATTACAAACAGTTCTGTGCGATCATCGTCATACACAACCCCTTGTGCACAATGCATGAGTGTTTCTCAGGGTAGGTACCAAGAA
GTGAAATTCCTGGGTCATAGGGCGTGAGTCCGACATTTTTCTCCATTCTGCCCTGTTGCCCTCCAGAGTGGGTGTCCAGCTTTGCATACCTAAGTATGAG
AGTATCTGTTGTTCATATCCTCTACGACGCTCCATATATGAAACTTAAGTTTCTGCTAGTTGCCATCTTTGATCTATCATGTATGCAGTGACCTACTAAGAC
TGTAATTGGTACAGTAGATTCTTGTCATCTGTGTGTGAATTTAGCATTCATGGGCTTAATGCTGACAAGGCCCCCAGGGTCCAAGACATATAATCATGTAT
AATTTTGTCAAGGTATAATTTTTTAAATTGCTTTTGTCATGTGTCTGCTGGTGATGCCCAACCCAGTGCTCTGCACCCAGGTCACACTGTGGCTTTGTCC
TCTGCTTATGCCTGCATTGCAGCAACTGTCCTGAAGAGACCAAAATTATGCAGATTTAGGTAAGTCCATGGCTAATGTTATTATATTATGTGCTATTGTAAT
GGATGGGGCTGTGGAGTGTATGAATTTATAAATCACTGGTCTTGTAATTAAAATTCAAACACTATAGAAAAAGGCCATGTAGAAGATAAAAGTTCCTCTA
TAATCCCGGACCCCTAAGATAACTACTAATGACAACTTCATTTATATTCCTTCAGACATTTTCTGGCTGTGGATGTACTAAAATGTATCCTATTATTCTCTG
CCCTAAAATGGAATCATACAAGGTGTACTGTTATTTTTATGGCTCTATAACATGTCATATTGTACGTGTTGGTATGGTCATTTTAACCATTTTTCTAGTGAT
GGCTTTGAGGTTATTTGCAGTTTCCTAGCCATCTCAAAGTGTGCTGCGGGGATCTCTTTTGCATCCCTCTGGGTGCAGAGCTGAGGCACCCAGAGGCAG

TGTCCAGAGGAGGCAGCATCTGTAGGTGTCTTCACCTGCTCTGGCTCTTGGCACATCTGGTTGGTGACACTGTTTTGTGAGATGGGTTGAAAGCACGT
GCTGCCAAAATAGAATAATGTTGGTCCTCTCCTCATGTGCCGTGGAACTGGGGTAAAACTGCGTAGTGGCTGCAGCTGCCTGTCCATACCGGAATCGAG
TATAACACGGTGCCTGGCTTAGCACAAAACAGTAGTGGGTCCTGCAGGCCCCAGAGTCTAATTCCTGGTATTCTTTCCCCTACACAGATTAAATAAACC
AAAAACAAACTATTCTAGGAAAGCGTCTGTGACATTTGTAAAAAGTGGTATTTAATGATCTTTTATTCACTTGTCTGTTTAGTTTGTTGAAATCTTAAGTG
GCATCCTGGTCTGGGAAGGAGTGCTGTCTGCGCCTGCCCTCCGCTGGGCACAGCGTGGCTGCTTCAGGGGCTAAGCACACACTTTCTGTCTTCTAAAG
GGCCGCCACATGCCAGGAGCTCAGGTGTGAGCCCGGCTCTGGCTCTTACCTCATAGGGTCACTCATAGGGGCACAGGGAGCAGAACATTGTACACAG
CGAGGCACCACCCGGCTTGGCATCTGCCTCGGTGGACTTACTACCTCTAGAAGGAAATACCTGAGTTCCTCTGGCCTCAGCTCCTAGAGTGACTGGTG
TGCTGTCCCTGTTACTCTTCTGTCAAGGTGACAACTGTGTGACCCATCATCTGTGTGTCAAAGCAAGGCCCTGCCTGGGCCTCTGCTCCTGTGCTGACC
CCAAAGGCAAATGCTTTGCTAGTTTCCTTCCAGTTAATTTCACCTATGAATAGATGTGTGAAAACTGTTCAAAGCCATACCTGCACATGTTTGAACTTCA
AACCCTGTGGGTGATTCAGTGGCATCTTTCTCTAACCCCCAGCCTCCCTTCCCACAGAGGCCACCGTCATGGCCAGTTGCTGCAGTTTCTTTCCAGAGA
ACCTGTGTATGTGTAAAGCTGTACAGGCGTGGGTACACCACACAGCCTGTCTTGCACTGTGGACTGTTGAGTTACTAGTACATCTAGGTAAGCACCGCA
TATCTGTATTCATGTCTGCCTTGGTCTTTTCAACATCTGTGTGGTAGCCGTGTTTGAATTACCCATTCCCTTTTTGGGGAACCATTAAGTTGTTTCAGCAA
TTTTTACTGTAGATAAGGCTATACCGCATATCTGTGTACATGGGTTTTTATGTACATGGGCAAGTATATCTGTGAGAGAAAAGTTTCCTCAGGAGGAATTC
TGGGCACAGCATGTGTAAATTTCTAAATATGATGGACACCCCCAGCTTCCACCTCAAGGAGGTTGGTCCCATTGACATTTCCCCACACCTTCACCCAGG
CTGTGCCCTTAAACTTGGTTATTTGTCAATGTGAGAAGTGGAAAATAGTATTTAATTGTAGTTTGGATTTGTATTTCTATTGGGTTGTATACTTACTGATTA
ATAATAAGAGCTCTTTACATATTAAGGAAATTAACCCTTTTCAAATACATTCCTATTTCTCACTAATCTTTAAGTTTTATTGTAATATTTTGCTCTTTAGTTTA
TATATATATGTATATATATATATATGTATATATATATATATACATATATATATACATATATATATACTAATTTTCTTTTATGGTTCCTGGATTTTGTGAGTAGTTTGA
AAAGGCTAATCCAGCTGAAGATTTTGTTGTTGTTGTTAAACCCCATGTTTTCTCCTAACTCTTTTTATTTTTATTTTGGAGGACTCTATCTAGACTTAATTT
TAGCATAACAAGTGACAGGGTTAGTTAGCCTGTTGTCCTTACACCATTTTCTGGCTAATACAGCTATTAACTATTGATCTGTCTATTCACGTGCCAGTTCC
TAATGGTTTTACATAGTGTAATCTGCACTTCAAAATAGCGAAGGGAAGCCCTACCTCATTATTCTACTTTTCCAGAATTCTCCTGGCTATTCCAGGCTGCA
TGTTTACCTTAACCTTCCCTGTGATGTCTTCATGCCGTTGTCTTCTTATGCAAGAATAAGGTACGTCTTTCCATCCACTCACGTCTATTTAATTTGACTTTG
CATTACACAGAAAGCTGGTCTTGGTCTGTCTACCTCGGCATCTAGTTGTCCTCACTGCCCCCTAGCCGACCCCACCCCATCTGACTGACTACCCCATCA
CAGAGTACTTTTATTTACGTTTTGCTCTGCCTAATGGTTACTTGATACTGTCACGCCGACAGTGTCCAGTTCAGTGGTCTTTGCAGTTGAAATGCTCCCG
TACACACTGTCTTGTTAAAAATGCCAGTAAGTTCATACAAACCCAGCTTGCACCCAAGGTCACATTCAGAGAGCGTAGGGCTGGGATGGGTTGTTTTC
CAAGCTTCTGCCACTGTGTGGCTAGCTCTTCCCACTGGGAAGTTCTGTGTACCCGGAATGTCGGAGTGGAGTCCTGTTCTAGTGTCCAGCACCTGACC
CTGTGCCCAACCCCTCAACAGCCTATTCCTGCTGTCCACAGCCTGCTGGAACTTTTTACAAAATATGTTGCCATGCTGGACCCTGGGCACTGGACATAA

GCCCCCTGGCAGCCTTTTTCATGTCACCCAAAGGGGTAATTGTCCTACTGGTGGTCTGTAAGATGAGTTAGGGTGACTTGCTAATAGACATTGTAAATCT
TAATATTTATGTATGTATTTTATTATTACCGGTTTTCCATTTATGATGGTAATATTGTTTCTTCTAAGAATATTTATTTTTCCTTCTAAATATTGAGATAAAATT
CATGCTTTTGAAATGTTCTATTCAGTGGCTTTTAGTATATTTGCTATGTTGTGCAACCATCGACACTATCCATTTCTAGAACTTTTTCGTCATCCCAAACAG
ACGCTCTGTATTCATAAAAAAAATAACTTCCTACCTGTCTCTCCCCCTAGTCTTTGGTAACCTTTGTTATACTGGTAAACTTTGTTGTGCTCTCTGTCTGTG
TGAATTTGCCTATTCTAGGGGCCTCATATAAGTGTAATCATACAGTATTTGTCTTTTTGGGTCTGTCTGATTTCACTTAGCGGGTTTTCAGGGTTCATTCAT
GTTGCAGCATATAACAGTACTGCGTTCCTTTTTCTGGCTGAATAATATTCCACTGTATGGATAGACCCCATTTTGTTTATTCACACATCATTTGGACATTTG
GATTATTTCTGGTTTTTGGCTATTATGAACAATGGTGCTATGAACAGTTGCGTACAAGTTTTTGTGTGAACATATGTTTTCAATTCTCTCATTATATACCTA
GGAGTAGAATTACTGGGTCATATGGTAACTGTATATTTTTGAGGAACTGCCAAACTATTTTCCCACGTCCATGCACCATTTCACATTCCCACCAGTAAGT
AAGAGGGTTCCAATTTCTGCGCATTCTTGCCAACACTAGTTATTATCTGACTTTCTGGTTATAATCATTCTAATGAGTGTGAAGTAGCCTCTGGTGTCATT
TGGATTTGCATTTCTCTGATGAGTGATGCTATCAAGCACCTTTGCTGGTGCTGTTGGCCATATGTGTATGTTCCCTGGAGAAGTGTCTGTGCTGAGCCTT
GGCCCACTTTTTAATTAGGCGTTTGTCTTTTTATTACTGAGTTGTAAGAGTTCTTTATATATTCTGGATTCTAGACCCTTATCAGATACATGGTTTGCAAATA
TTTTCTCCCATTCTGTGGGTTGTGTTTTCACTTTATCGATAATGTCCTTAGACATATAATAAATTTGTATTTTAAAAGTGACTTGATTTGGCTGTGCAAGGT
GGCTCACGCTTGTAATCCCAGCACTTTGGGAGACTGAGGTGGGTGGATCATATGAGGAGGCTAGGAGTTCGAGGTCAGCCTGGCCAGCATAGCGAAA
ACTTGTCTCTACTAAAAATACAAAAATTAGTCAGGCATGGTGGTGCACGTCTGTAATACCAGCTTCTCAGGAGGCTGAGGCACGAGGATCACTTGAAC
CCAGGAGGAGGAGGTTGCAGTGAGCTGAGATCATGCCAGGGCAACAGAATGAGACTTTGTTTAAAAAAAAAAAAAAAGTGACTTGATTTAAGGGAAA
AAATGACTGGCTATATTCAGTCAGATATGGCAAAAAGTCTCAAGGTGTTAATGTGAATGATTAAGGTCTTGGGGGGGGTGTCCCCTATCAGACTACAGG
TGTTTAGAGGCACAGAAAAAGGTGCAGTTGGGTTCTTAATGTGAAATGATGAGAAGCACAACTCCAGTGTGTCTCTTTGTGTAGAATGTCAGCAGACA
CCCCCTGCTAGATGTGCTGGATCATGGGAAAGCATTTCCATTTGTTACTAGATTGTTCAGAAGTTTTAATTTATGATGGGTGTGGTGGCTCATGCCTGTAG
TCCCAGCACTGTGGGAGGCTGAGGCAGGAGGATCATCTGAGGCCAAGAGTTCAAGATCAGCCTGGGCAACATAGTGATACCCTATCTCTTAAAAAAGA
AGAAGTTTTTAAATTTGAAATAATAATAGGTACTGGATTTATGCAAATGTCTTTTCTGCGTCTTTTGAGATGAGTATCAGGTTTTTTTTTTCCTTTTATCA
TCTGATGATGAACTTAATGTTTCCATTTGTATTAATGGAATACTAAGTCCCTCTGTGATTTCTGAACCAAGCTATTCCTAGGCCTGAGTTTTATTTTGTTGA
CACAGAAATAAATTAGAAGGCCAAGCGTGGTGGCATGTGCCTGTAGTCCTAGTTGCTGAGGTAAGAGGATTGCTTGAGCCCAGGAGTTCAAGGCTGC
AGCAAGCTTTGATTGCGCCACTGCACTCCAGCCTTGGCGACAGACTAAGACGCTGTCTCAAAAAAAAAACAAAAACGACAAAAAAAAAAACAAAACAG
AAAAAATAAACTAAGGCAATGACAGTCCCTGGCAAATGCTGGGAGGGAGGCAGCAGTGGTCAGGGAAGGTAACCCTGAAGCAGGACTTGTAAAGCA
AATAAGATTGGGAGGCCAAGGTGGGTGGATCACGAGGTCAGGAGTTCGAGACCAGCCTGGCCAACATAGTGAAACCCCGTCTTTACTAAAAATACAA
AAAAATTAGCCAGGTGTGGTGGTGGGTGCCTGTAGTCCCAGCTACTTGGGAGGCTGAGGCAGGAGAATCTCGAACCCAGGAGGCGGAGGTTACAGTC

AGCTGAGACCGCACCATTGCACTCCAGCCTGGGTGACAGAGCAAGATTCCGTCTCAAAAAAAAAAAAAAAAAAAAAACCAAGAAGAAAAGGAATG
AATTAGAACTTCTTCTGCTTGGACTTAAGGGCATCATCAGGCAGGTTTTGGGTAGGATAGCAGGGGAGGCAGAGACATAGTCGGGGTCAGTGGTCATG
AGTGTGGCTTTGAGCCCAAAAACTTGGTTTCTGTTCCCTACTTTGCCACTCAGTAGTGCATGACTTTGGCCAAATTTCTTAAATTCATGAAGCAAGTTTC
CGGGTGAATGAAATGGGGATAAAAATAGTGTTCAAACCTATCCGTTGGTTTGTGTGAAACTGAAATGAATAGTATCGTGCAGGTACTTGTGAGCAAGG
GGAGCTGCTGTTTCCTGTCCCTTTATGATGGGAAATATCTAGACAAGTTCCCAACCCTCTGCACTGCAGGCTGCATGGCACGGAGGGTCTTGTAACACC
AGCTGGGGCTGGCCTTCTTTTAGGAGCTTCAGTGGTTCTGAAAACTTTTATTTGTTTGTTTGTTTTAGTAGATGTGGGGTCTTTCTGTGTTGCCCGGACT
GGTCTCAAACTTCTGGACTCAAGTGATCCTCCCCCGCTCAACCTCCCAAAGTGTTGGGATTACAGGTGTGAGCCACTGTGCCCAGCCTTGAAAACTTT
TTCAGGTTCTTCCAGGGTTACTGGGCTATTAAATATTTCTATTTCATTATAAGTCAGTTTTTCAAAGTTATATTATCTTAATTACCTTTTTTATATGTATTAGT
GTAGAGTAGCATTTTATATTTTGATATCCTCCTTATGCATAGTTTTTCACTTTTTATTCCTAGTTTTTCGTTTTTAATAAGACTTTCAAGAAATTTATTTTATT
GGCCTTTTGAAAAAAGCAGCTTTAGATAAAGTAAGCAGTTCTGCTTTCATTTTATAATTTATTTCTACTTTTGTTTCATTAATCTTTTCCTCCGGCATGCCT
TGGATTTTGTTGTGTTACTCTTTTTCTAGAGGCTCGCATTGTGTGTCTGGTTCACTTATGATCACGCTTGCCTACTTTTAAGAATGGAAGAGGGGAGGTG
GAGGGTGGCTGCACAGTCGAGGGTGTGAGGCAGTCTTGCTCTAGCCCCACCATGCCCTCAGCCCGCTGTGGCCACGCTGGTTCCTCAATTGCTGGGGC
GTGCAGTGTCTGTAAGGGAGGCTACTGATGCCATCCGAGGAAGATGTAAGGTTTCGTGTGGGCAGCGAGAGCCTAGCAGGCATGTGGGGTGCCCAGC
AAAGGGTAACAGTGGACAGTTGTTGCCTCATTCCACAGAGTTTTGATTTTTTTTTTTTTTTTAATGGTCACTCCATCAACATCCCCCATGGCCAGAGCCT
GAGCTGGTCCCCAGAGACACAGGCATTCAGCTGACAGCCTCGCCTTCACGCTGCTGCTGTTCTCATGGGGGACAGGCCTCAGGTGGCAATGCACAAA
TCATTAGTTAAGGGCAGTTGTGACAGTTACCAAGGAGTGTAGTCCCCCGCCCCCCGCCCAGTGAAAACAGCCCTAACCAGGGGTGGGGACCTTTGGG
CTCTGACCCGAAGGGTAGGAGAAGCTGGAAGGACAGCATTCCTGTCTGCGAAGGCAGGAGCAAAGCTGCCAGGCTATGAAGGAAATGGCTGGAGCC
TGAAGTCATGCAAGCTGGGGCTGGCAGGGACAGGGCCAACTTCCAGGCCTGGGGGCCACCATGAGGATTCAGGACGTGACCCCCAGGGCACATGAA
GGCCTTCCATCTGTATTTAAGAAAAGACTTTATCAGACGAGTATGGTGGCTCACGCCTGAATCTTAGCACTTTGGGAGGCTGAGGCAGGTGGATCACGA
GGTCAGGAGTTCAATACCAGCCTGGCCAATATGGTAAAACCCCATCTCTACTAAAACTACAAAAATTAGCCAGGCATGGTGGCGCACGCCTGTAGTCCC
AGCTACTCGGGAGGCTGAGGCAGAAGAATCACTTGAACCCGGGAGGTGGAGGTTACAGTGAGCCAAGATCGCGCCACTACACTCCAGCCTGGGTGA
CAGAGTGAGACTCCGTCTCAAAAAAACCAAAAGACTTTATCTTATTTCCTATATGTTTGTGGTTTCAGTCCTGATGTATAATTTGACCCTAGTTAGAATG
GTTATCTGAGGAAGTGGCCTGTACGATTTCTGCTTTTTTAAATGTGTGGCTCCCTTTCTTCATTGATTAACGTATGATTATTTTTATAAATGTTCCATGGCA
GTGGGAAGGGATTCTCTGTCACATTCCACATCTGGATCAGTTCCTCCCCATTTTGTTGGTCAAATCCGATCTGCCATATCCTGTGTAATGACAAGTGAGT
TGCATTCTCACCGTCACTCCTGGGGTCTCTCCGCTTCCCCTGAGCTGGCTCAGCAGTCTGCTCCATGTGTTTTGATGCAGGGTGACCCATTGGTATTCCC
GACACTAACGCCCCCGTCTGTGGACTGCTTGCTGCTTGGGCTTCACTGTGTCTGGTGTTGACAGTGCAGACCTAAAGGTGTGCACACATGTGCACACA

CACTCCGCTGTCTTCTTGTTTGCACTGGACTTAAATATCTATGAGGGTTATTTTCAACTGCTGAATTTGGAATGATTTTTATATCTTTTCTGCTTTCTGCCC
ATGTACATGTGTTTATTTTACACTGTTGTGATTGGTAGTTACTATGTGGGGACACAATTACTTGGGCTGAAATAATCCACCTGTTGTGGTTGGGGTCCTCT
GGGGCATTCCAGGGTGAGAGGTTGTCACTGCCACCTGGGCCATGTGGGCCGGCACCAGCATTTTGTGGTTACGAATTCTACAGTCACAAATATCTTTGG
GCAAATCCCCTTCTATACCTCAAGGCAGCTTTTGGTTTGCAACCCCACTGGCCAGAGGGAAGGGCCAGTCACTTGGCTCTCTCACTGCCCTGCGCCCC
AGATGGTTCTAGGGCTGCTGTTTTCCCTTGGCCCTGCCAACACCACTGTTTTTACTTCTGCTCATTGGCTGAGTGCAGTGGTTCCTGGAAGCCAGTGGC
ACGTTTCCCCGCGTAGCTCGCTTATCCCACAGCACACACCCAAGGGTTCTGTTGCTAACACGCTGAATTAATTCTTTGCTCATCTTACAGAGTGTGTTTT
GACTGCCCCCATTTCTGAGGCCTTGTAAGGCCAGAGCTTTGTTGCTTCATCGGCAGGTTGGGACTTAGATGGCCGTGAATGTTTCCTCTCTGCTGCTGC
AGTAAGTAAGTGCCCGCACCATAGTGTGTTTGGAGGCTGAAGTTGAAGCGAGGCTGTGAGGGGAGATGGACGTGTGAGGAGGGATGATGGGGCTTGA
GCAAAGTGGGGGAGGGGGCAAAGGCAGTTGGCCCAACACATTCCCCACCCCTTTGAGAGGTCTGAGGCCTGCAGACCTGGCTCGGAGCCCACCTGG
TAGTCCTCAGACTGTGTGTGTGTGTGTGTGTGTGTGTGTGTGTGTGTGTGTGTGTGTGTGTGTGTGTGTGTAAAAGAGAGAAGTTGTGGAGAAATGGG
GGGCTGATTCTGCTCAGATTCATCAGGATGAGTAGAAGGCACCCAGCTCTCACCCTGGCCTGACATGTGTGTCCCTGAGCAGGTTACAGTCCTCTCTGA
GCCTCTGCTTCCCATCTGGACCCTGCTGGGCAGGGCTTCTGAGCTCCTTAGCACTAGCAGGAGGGGCTCCAGGGGCCCTCCCTCCATGGCAGCCAGGA
CAGGACTCTCAAATGAGGACAGCAGAGCTCGTGGGGGGCTCCCACGGACCCGCCGTGGGCCCAGGGGAGGCAGAGCCTGAGCCAACAGCAGTGGT
GCTGTGGACCGTGGATCCTGAGGGTGGCCTGGGGCAAGTACCGGCTGAGGGTCCAGGTGGGCTTTGTGTACCTTTGGGTCCTGGGGCCCTGGTGACT
TGGACTCCAGGTTAGAGTCAAGTGACAGGAGAAAGGCTGGTGGGGCCCTGTGCTTCCGACTTCATTTCGAGTGATGGCAGTTCCCAGGAAGGAATCC
ACAGCTGACGGTGGCTGACAGATCAGAGAATGGAAGGCGAGGCAGGCGGGCGTCTGCGTGACCTCAGGTGCTTGGGGCCCAGCAGACCCAGAGAAC
CATTTCCACTAGGCCAGGGTGCCGGAAGTGTCCACAGGTCTTAGATTCCCTGTTCAGATGAAAAGATTTGTGCCTTTAATGATAAAAGTGATCTGCATA
GAGTCAAAAATTCAAGCCATGGGTATAAAATGCAAGTAAAATCCCTGCCCTCACCTATCCCACCCTACTACACAGAGATGTCCTCTCGAGTTTCCTAGA
CTCACTCTGGAAATTTCTGTATACACACAGAAGCTTGTGCCTCTGCTCGTGAAGGCAGAGGGAGGGAGAGCTGAAGGGCCAGCACCTTCTCACCTGT
GGGCCCCCTCAGTGCTCGGTCCCAGAGCATGCAGGACTGTGCCTCGTGTTCAGTTTGCTGGTCTGACTTCATGCTCCTTGGGCAGGATATGCATGTGCC
ATGCTAGGAGACATGTGGATGTGAAGCTGGGGGACAATGTCCCCTGGCTATGCCTTTACAAGGGAAGTAAGGAAGGTAGGAGGTGAGCCTGGGAGGG
AGGGAGGGAGGCGCGGAGCCGCCGCAGGTGTTTCTTTTACTGAGTGCAGCCCATGGCCGCACTCAGGTTTTGCTTTTCACCTTCCCATCTGTGAAAGA
GTGAGCAGGAAAAAGCAAAA
>ENST00000601801.3|ENSG00000245532.8|OTTHUMG00000166321.7|OTTHUMT00000476511.2|RP11-867O8.8-005|NEAT1|3524|
AGTTAGCGACAGGGAGGGATGCGCGCCTGGGTGTAGTTGTGGGGGAGGAAGTGGCTAGCTCAGGGCTTCAGGGGACAGACAGGGAGAGATGACTGA
GTTAGATGAGACGAGGGGGCGGGCTGGGGGTGCGAGAAGGAAGCTTGGCAAGGAGACTAGGTCTAGGGGGACCACAGTGGGGCAGGCTGCATGGA

AAATATCCGCAGGGTCCCCCAGGCAGAACAGCCACGCTCCAGGCCAGGCTGTCCCTACTGCCTGGTGGAGGGGGAACTTGACCTCTGGGAGGGCGCC
GCTCTTGCATAGCTGAGCGAGCCCGGGTGCGCTGGTCTGTGTGGAAGGAGGAAGGCAGGGAGAGGTAGAAGGGGTGGAGGAGTCAGGAGGAATAG
GCCGCAGCAGCCCTGGAAATGATCAGGAAGACCAAGGGCTGTGAACCCGCCTGGGGATGAGGCCTGGTCTTGTGGAACTGAACTTAGCTCGACGGG
GCTGACCGCTCTGGCCCAGGGTGGTATGTAATTTTCGCTCGGCCTGGGACGGGGCCCAGGCCGGGCCCAGCCTGGTGGAGCGTCCAGGTCTGGGTGC
GAAGCCAGGCCCCTGGGCGGAGGTGAGGGGTGGTCTGAGGAGTGATGTGGAGTTAAGGCGCCATCCTCACCGGTGACTGGTGCGGCACCTAGCATGT
TTGACAGGCGGGGACTGCGAGGCACGCTGCTCGGGTGTTGGGGACAACATTGACCAACGCTTTATTTTCCAGGTGGCAGTGCTCCTTTTGGACTTTTC
TCTAGGTTTGGCGCTAAACTCTTCTTGTGAGCTCACTCCACCCCTTCTTCCTCCCTTTAACTTATCCATTCACTTAAAACATTACCTGGTCATCTGGTAAG
CCCGGGACAGTAAGCCGAGTGGCTGTTGGAGTCGGTATTGTTGGTAATGGTGGAGGAAGAGAGGCCTTCCCGCTGAGGCTGGGGTGGGGCGGATCGG
TGTTGCTTGCCTGCAGAGAGGGTGGGGAGTGAATGTGCACCCTTGGGTGGGCCTGCAGCCATCCAGCTGAAAGTTACAAAAATGCTTCATGGACCGT
GGTTTGTTACTATAGTGTTCCTCATGGCGAGCAGATGGAACCGGGAGACATGGAGTCCCTGGCCAGTGTGAGTCCTAGCATTGCAGGAGGGGAGACCC
TGGAGGAGAGAGCCCGCCTCAATTGATGCCTGCAGATTGAATTTCCAGAGGCTTAGGAGGAGGAAGTTCTCCAATGTTCTGTTTCCAGGCCTTGCTCA
GGAAGCCCTGTATTCAGGAGGCTACCATTTAAAGTTTGCAGATGAGCTTATGGGGGGCAATCTTAAAAAGTCCACAGCAGATGCATCCGGCTCGAGGG
GCCATCAGCTTTGAATAAATGCTTGTTCCAGAGCCCATGAATGCCAGCAGGCACCCCTCCTTTCCTGGGGTAAAGGTTTTCAGATGCTGCATCTTCTAA
ATTGAGCCTCCGGTCATACTAGTTTTGTGCTTGGAACCTTGCTTCAAGAAGATCCCTAAGCTGTAGAACATTTTAACGTTGATGCCACAACGCAGATTG
ATGCCTTGTAGATGGAGCTTGCAGATGGAGCCCCGTGACCTCTCACCTACCCACCTGTTTGCCTGCCTTCTTGTGCGTTTCTCGGAGAAGTTCTTAGCC
TGATGAAATAACTTGGGGCGTTGAAGAGCTGTTTAATTTTAAATGCCTTAGACTGGGGATATATTAGAGGAAGCAGATTGTCAAATTAAGGGTGTCATTG
TGTTGTGCTAAACGCTGGGAGGGTACAAGTTGGTCATTCCTAAATCTGTGTGTGAGAAATGGCAGGTCTAGTTTGGGCATTGTGATTGCATTGCAGATT
ACTAGGAGAAGGGAATGGTGGGTACACCGGTAGTGCTCTTTTGTTCTTGCTTCGTTTTTTTAAACTTGAACTTTACTTCGTTAGATTTCATAATACTTTCT
TGGCATTCTAGTAAGAGGACCCTGAGGTGGGAGTTGTGGGGGACGGGGAGAAGGGGACAGCTTGGCACCGGTCCCGTGGGCGTTGCAGTGTGGGGG
ATGGGGGTATGCAGCTTGGCACTGGTACTGGGAGGGATGAGGGTGAAGAAGGGGAGAGGGTTGGTTAGAGATACAGTGTGGGTGGTGGGGGTGGTA
GGAAATGCAGGTTGAAGGGAATTCTCTGGGGCTTTGGGGAATTTAGTGCGTGGGTGAGCCAAGAAAATACTAATTAATAATAGTAAGTTGTTAGTGTTG
GTTAAGTTGTTGCTTGGAAGTGAGAAGTTGCTTAGAAACTTTCCAAAGTGCTTAGAACTTTAAGTGCAAACAGACAAACTAACAAACAAAAATTGTTT
TGCTTTGCTACAAGGTGGGGAAGACTGAAGAAGTGTTAACTGAAAACAGGTGACACAGAGTCACCAGTTTTCCGAGAACCAAAGGGAGGGGTGTGT
GATGCCATCTCACAGGCAGGGGAAATGTCTTTACCAGCTTCCTCCTGGTGGCCAAGACAGCCTGTTTCAGAGGGTTGTTTTGTTTGGGGTGTGGGTGTT
ATCAAGTGAATTAGTCACTTGAAAGATGGGCGTCAGACTTGCATACGCAGCAGATCAGCATCCTTCGCTGCCCCTTAGCAACTTAGGTGGTTGATTTGA
AACTGTGAAGGTGTGATTTTTTCAGGAGCTGGAAGTCTTAGAAAAGCCTTGTAAATGCCTATATTGTGGGCTTTTAACGTATTTAAGGACCACTTAAG

ACGAGATTAGATGGGCTCTTCTGGATTTGTTCCTCATTTGTCACAGGTGTCTTGTGATTGAAAATCATGAGCGAAGTGAAATTGCATTGAATTTCAAGG
GAATTTAGTATGTAAATCGTGCCTTAGAAACACATCTGTTGTCTTTTCTGTGTTTGGTCGATATTAATAATGGCAAAATTTTTGCCTATCTAGTATCTTCAA
ATTGTAGTCTTTGTAACAACCAAATAACCTTTTGTGGTCACTGTAAAATTAATATTTGGTAGACAGAATCCATGTACCTTTGCTAAGGTTAGAATGAATAA
TTTATTGTATTTTTAATTTGAATGTTTGTGCTTTTTAAATGAGCCAAGACTAGAGGGGAAACTATCACCTAAAATCAGTTTGGAAAACAAGACCTAAAAA
GGGAAGGGGATGGGGATTGTGGGGAGAGAGTGGGCGAGGTGCCTTTACTACATGTGTGATCTGAAAACCCTGCTTGGTTCTGAGCTGCGTCTATTGAA
TTGGTAAAGTAATACCAATGGCTTTTTATCATTTCCTTCTTCCCTTTAAGTTTCACTTGAAATTTTAAAAATCATGGTTATTTTTATCGTTGGGATCTTTCTG
TCTTCTGGGTTCCATTTTTTAAATGTTTAAAAATATGTTGACATGGTAGTTCAGTTCTTAACCAATGACTTGGGGATGATGCAAACAATTACTGTCGTTGG
GATTTAGAGTGTATTAGTCACGCATGTATGGGGAAGTAGTCTCGGGTATGCTGTTGTGAAATTGAAACTGTAAAAGTAGAT
>ENST00000612303.2|ENSG00000245532.8|OTTHUMG00000166321.7|OTTHUMT00000473553.2|RP11-867O8.8-003|NEAT1|1053|
AGCGACAGGGAGGGATGCGCGCCTGGGTGTAGTTGTGGGGGAGGAAGTGGCTAGCTCAGGGCTTCAGGGGACAGACAGGGAGAGATGACTGAGTTA
GATGAGACGAGGGGGCGGGCTGGGGGTGCGAGAAGGAAGCTTGGCAAGGAGACTAGGTCTAGGGGGACCACAGTGGGGCAGGCTGCATGGAAAAT
ATCCGCAGGGTCCCCCAGGCAGAACAGCCACGCTCCAGGCCAGGCTGTCCCTACTGCCTGGTGGAGGGGGAACTTGACCTCTGGGAGGGCGCCGCTC
TTGCATAGCTGAGCGAGCCCGGGTGCGCTGGTCTGTGTGGAAGGAGGAAGGCAGGGAGAGGTAGAAGGGGTGGAGGAGTCAGGAGGAATAGGCCG
CAGCAGCCCTGGAAATGATCAGGAAGGCAGGCAGTGGGTGCAGGGCTGCAGGAGGGCCGGGAGGGCTAATCTTCAACTTGTCCATGCCAGCAGCCC
CTTTTTTTCCAGACCAAGGGCTGTGAACCCGCCTGGGGATGAGGCCTGGTCTTGTGGAACTGAACTTAGCTCGACGGGGCTGACCGCTCTGGCCCAG
GGTGGTGGCAGTGCTCCTTTTGGACTTTTCTCTAGTGTTCCTCATGGCGAGCAGATGGAACCGGGAGACATGGAGTCCCTGGCCAGTGTGAGTCCTAG
CATTGCAGGAGGGGAGACCCTGGAGGAGAGAGCCCGCCTCAATTGATGCCTGCAGATTGAATTTCCAGAGGCTTAGGAGGAGGAAGTTCTCCAATGT
TCTGTTTCCAGGCCTTGCTCAGGAAGCCCTGTATTCAGGAGGCTACCATTTAAAGTTTGCAGATGAGCTTATGGGGGGCAATCTTAAAAAGTCCACAGC
AGATGCATCCGGCTCGAGGGGCCATCAGCTTTGAATAAATGCTTGTTCCAGAGCCCATGAATGCCAGCAGGCACCCCTCCTTTCCTGGGGTAAAGGTTT
TCAGATGCTGCATCTTCTAAATTGAGCCTCCGGTCATACTAGTTTTGTGCTTGGAACCTTGCTTCAAGAAGATCCCTAAGCTGT
>ENST00000646243.1|ENSG00000245532.8|OTTHUMG00000166321.7|OTTHUMT00000494370.1|RP11-867O8.8-006|NEAT1|2994|
ATAGCTGAGCGAGCCCGGACCAAGGGCTGTGAACCCGCCTGGGGATGAGGCCTGGTCTTGTGGAACTGAACTTAGCTCGACGGGGCTGACCGCTCTG
GCCCAGGGTGGTGGCAGTGCTCCTTTTGGACTTTTCTCTAGGTTTGGCGCTAAACTCTTCTTGTGAGCTCACTCCACCCCTTCTTCCTCCCTTTAACTTA
TCCATTCACTTAAAACATTACCTGGTCATCTGGTAAGCCCGGGACAGTAAGCCGAGTGGCTGTTGGAGTCGGTATTGTTGGTAATGGTGGAGGAAGAG
AGGCCTTCCCGCTGAGGCTGGGGTGGGGCGGATCGGTGTTGCTTGCCTGCAGAGAGGGTGGGGAGTGAATGTGCACCCTTGGGTGGGCCTGCAGCCA
TCCAGCTGAAAGTTACAAAAATGCTTCATGGACCGTGGTTTGTTACTATAGTGTTCCTCATGGCGAGCAGATGGAACCGGGAGACATGGAGTCCCTGG

CCAGTGTGAGTCCTAGCATTGCAGGAGGGGAGACCCTGGAGGAGAGAGCCCGCCTCAATTGATGCCTGCAGATTGAATTTCCAGAGGCTTAGGAGGA
GGAAGTTCTCCAATGTTCTGTTTCCAGGCCTTGCTCAGGAAGCCCTGTATTCAGGAGGCTACCATTTAAAGTTTGCAGATGAGCTTATGGGGGGCAATC
TTAAAAAGTCCACAGCAGATGCATCCGGCTCGAGGGGCCATCAGCTTTGAATAAATGCTTGTTCCAGAGCCCATGAATGCCAGCAGGCACCCCTCCTT
TCCTGGGGTAAAGGTTTTCAGATGCTGCATCTTCTAAATTGAGCCTCCGGTCATACTAGTTTTGTGCTTGGAACCTTGCTTCAAGAAGATCCCTAAGCTG
TAGAACATTTTAACGTTGATGCCACAACGCAGATTGATGCCTTGTAGATGGAGCTTGCAGATGGAGCCCCGTGACCTCTCACCTACCCACCTGTTTGCC
TGCCTTCTTGTGCGTTTCTCGGAGAAGTTCTTAGCCTGATGAAATAACTTGGGGCGTTGAAGAGCTGTTTAATTTTAAATGCCTTAGACTGGGGATATAT
TAGAGGAAGCAGATTGTCAAATTAAGGGTGTCATTGTGTTGTGCTAAACGCTGGGAGGGTACAAGTTGGTCATTCCTAAATCTGTGTGTGAGAAATGG
CAGGTCTAGTTTGGGCATTGTGATTGCATTGCAGATTACTAGGAGAAGGGAATGGTGGGTACACCGGTAGTGCTCTTTTGTTCTTGCTTCGTTTTTTTAA
ACTTGAACTTTACTTCGTTAGATTTCATAATACTTTCTTGGCATTCTAGTAAGAGGACCCTGAGGTGGGAGTTGTGGGGGACGGGGAGAAGGGGACAG
CTTGGCACCGGTCCCGTGGGCGTTGCAGTGTGGGGGATGGGGGTATGCAGCTTGGCACTGGTACTGGGAGGGATGAGGGTGAAGAAGGGGAGAGGG
TTGGTTAGAGATACAGTGTGGGTGGTGGGGGTGGTAGGAAATGCAGGTTGAAGGGAATTCTCTGGGGCTTTGGGGAATTTAGTGCGTGGGTGAGCCA
AGAAAATACTAATTAATAATAGTAAGTTGTTAGTGTTGGTTAAGTTGTTGCTTGGAAGTGAGAAGTTGCTTAGAAACTTTCCAAAGTGCTTAGAACTTTA
AGTGCAAACAGACAAACTAACAAACAAAAATTGTTTTGCTTTGCTACAAGGTGGGGAAGACTGAAGAAGTGTTAACTGAAAACAGGTGACACAGAG
TCACCAGTTTTCCGAGAACCAAAGGGAGGGGTGTGTGATGCCATCTCACAGGCAGGGGAAATGTCTTTACCAGCTTCCTCCTGGTGGCCAAGACAGC
CTGTTTCAGAGGGTTGTTTTGTTTGGGGTGTGGGTGTTATCAAGTGAATTAGTCACTTGAAAGATGGGCGTCAGACTTGCATACGCAGCAGATCAGCAT
CCTTCGCTGCCCCTTAGCAACTTAGGTGGTTGATTTGAAACTGTGAAGGTGTGATTTTTTCAGGAGCTGGAAGTCTTAGAAAAGCCTTGTAAATGCCTA
TATTGTGGGCTTTTAACGTATTTAAGGGACCACTTAAGACGAGATTAGATGGGCTCTTCTGGATTTGTTCCTCATTTGTCACAGGTGTCTTGTGATTGAA
AATCATGAGCGAAGTGAAATTGCATTGAATTTCAAGGGAATTTAGTATGTAAATCGTGCCTTAGAAACACATCTGTTGTCTTTTCTGTGTTTGGTCGATA
TTAATAATGGCAAAATTTTTGCCTATCTAGTATCTTCAAATTGTAGTCTTTGTAACAACCAAATAACCTTTTGTGGTCACTGTAAAATTAATATTTGGTAGA
CAGAATCCATGTACCTTTGCTAAGGTTAGAATGAATAATTTATTGTATTTTTAATTTGAATGTTTGTGCTTTTTAAATGAGCCAAGACTAGAGGGGAAACT
ATCACCTAAAATCAGTTTGGAAAACAAGACCTAAAAAGGGAAGGGGATGGGGATTGTGGGGAGAGAGTGGGCGAGGTGCCTTTACTACATGTGTGAT
CTGAAAACCCTGCTTGGTTCTGAGCTGCGTCTATTGAATTGGTAAAGTAATACCAATGGCTTTTTATCATTTCCTTCTTCCCTTTAAGTTTCACTTGAAAT
TTTAAAAATCATGGTTATTTTTATCGTTGGGATCTTTCTGTCTTCTGGGTTCCATTTTTTAAATGTTTAAAAATATGTTGACATGGTAGTTCAGTTCTTAAC
CAATGACTTGGGGATGATGCAAACAATTACTGTCGTTGGGATTTAGAGTGTATTAGTCACGCATGTATGGGGAAGTAGTCTCGGGTATGCTGTTGTGAAA
TTGAAACTGTAAAAGTAGATGGTTGAAAGTACTGGTATGTTGCTCTGTATGGTAAGAACTAATTCTGTTACGTCATGTACATAATTACTAATCACTTTTCT
TCCCCTTTACAGCACAAATAAAGTTTG

>ENST00000645023.1|ENSG00000245532.8|OTTHUMG00000166321.7|OTTHUMT00000494371.1|RP11-867O8.8-008|NEAT1|3300|
TGTGTGGAAGGAGGAAGGCAGGGAGAGGTAGAAGGGGTGGAGGAGTCAGGAGGAATAGGCCGCAGCAGCCCTGGAAATGATCAGGAAGGCAGGCA
ACCAAGGGCTGTGAACCCGCCTGGGGATGAGGCCTGGTCTTGTGGAACTGAACTTAGCTCGACGGGGCTGACCGCTCTGGCCCAGGGTGGTATGTAA
TTTTCGCTCGGCCTGGGACGGGGCCCAGGCCGGGCCCAGCCTGGTGGAGCGTCCAGGTCTGGGTGCGAAGCCAGGCCCCTGGGCGGAGGTGAGGGG
TGGTCTGAGGAGTGATGTGGAGTTAAGGCGCCATCCTCACCGGTGACTGGTGCGGCACCTAGCATGTTTGACAGGCGGGGACTGCGAGGCACGCTGC
TCGGGTGTTGGGGACAACATTGACCAACGCTTTATTTTCCAGGTGGCAGTGCTCCTTTTGGACTTTTCTCTAGGTTTGGCGCTAAACTCTTCTTGTGAG
CTCACTCCACCCCTTCTTCCTCCCTTTAACTTATCCATTCACTTAAAACATTACCTGGTCATCTGGTAAGCCCGGGACAGTAAGCCGAGTGGCTGTTGGA
GTCGGTATTGTTGGTAATGGTGGAGGAAGAGAGGCCTTCCCGCTGAGGCTGGGGTGGGGCGGATCGGTGTTGCTTGCCTGCAGAGAGGGTGGGGAGT
GAATGTGCACCCTTGGGTGGGCCTGCAGCCATCCAGCTGAAAGTTACAAAAATGCTTCATGGACCGTGGTTTGTTACTATAGTGTTCCTCATGGCGAGC
AGATGGAACCGGGAGACATGGAGTCCCTGGCCAGTGTGAGTCCTAGCATTGCAGGAGGGGAGACCCTGGAGGAGAGAGCCCGCCTCAATTGATGCCT
GCAGATTGAATTTCCAGAGGCTTAGGAGGAGGAAGTTCTCCAATGTTCTGTTTCCAGGCCTTGCTCAGGAAGCCCTGTATTCAGGAGGCTACCATTTAA
AGTTTGCAGATGAGCTTATGGGGGGCAATCTTAAAAAGTCCACAGCAGATGCATCCGGCTCGAGGGGCCATCAGCTTTGAATAAATGCTTGTTCCAGA
GCCCATGAATGCCAGCAGGCACCCCTCCTTTCCTGGGGTAAAGGTTTTCAGATGCTGCATCTTCTAAATTGAGCCTCCGGTCATACTAGTTTTGTGCTTG
GAACCTTGCTTCAAGAAGATCCCTAAGCTGTAGAACATTTTAACGTTGATGCCACAACGCAGATTGATGCCTTGTAGATGGAGCTTGCAGATGGAGCCC
CGTGACCTCTCACCTACCCACCTGTTTGCCTGCCTTCTTGTGCGTTTCTCGGAGAAGTTCTTAGCCTGATGAAATAACTTGGGGCGTTGAAGAGCTGTT
TAATTTTAAATGCCTTAGACTGGGGATATATTAGAGGAAGCAGATTGTCAAATTAAGGGTGTCATTGTGTTGTGCTAAACGCTGGGAGGGTACAAGTTG
GTCATTCCTAAATCTGTGTGTGAGAAATGGCAGGTCTAGTTTGGGCATTGTGATTGCATTGCAGATTACTAGGAGAAGGGAATGGTGGGTACACCGGTA
GTGCTCTTTTGTTCTTGCTTCGTTTTTTTAAACTTGAACTTTACTTCGTTAGATTTCATAATACTTTCTTGGCATTCTAGTAAGAGGACCCTGAGGTGGGA
GTTGTGGGGGACGGGGAGAAGGGGACAGCTTGGCACCGGTCCCGTGGGCGTTGCAGTGTGGGGGATGGGGGTATGCAGCTTGGCACTGGTACTGGG
AGGGATGAGGGTGAAGAAGGGGAGAGGGTTGGTTAGAGATACAGTGTGGGTGGTGGGGGTGGTAGGAAATGCAGGTTGAAGGGAATTCTCTGGGGC
TTTGGGGAATTTAGTGCGTGGGTGAGCCAAGAAAATACTAATTAATAATAGTAAGTTGTTAGTGTTGGTTAAGTTGTTGCTTGGAAGTGAGAAGTTGCT
TAGAAACTTTCCAAAGTGCTTAGAACTTTAAGTGCAAACAGACAAACTAACAAACAAAAATTGTTTTGCTTTGCTACAAGGTGGGGAAGACTGAAGA
AGTGTTAACTGAAAACAGGTGACACAGAGTCACCAGTTTTCCGAGAACCAAAGGGAGGGGTGTGTGATGCCATCTCACAGGCAGGGGAAATGTCTTT
ACCAGCTTCCTCCTGGTGGCCAAGACAGCCTGTTTCAGAGGGTTGTTTTGTTTGGGGTGTGGGTGTTATCAAGTGAATTAGTCACTTGAAAGATGGGC
GTCAGACTTGCATACGCAGCAGATCAGCATCCTTCGCTGCCCCTTAGCAACTTAGGTGGTTGATTTGAAACTGTGAAGGTGTGATTTTTTCAGGAGCTG
GAAGTCTTAGAAAAGCCTTGTAAATGCCTATATTGTGGGCTTTTAACGTATTTAAGGGACCACTTAAGACGAGATTAGATGGGCTCTTCTGGATTTGTTC

CTCATTTGTCACAGGTGTCTTGTGATTGAAAATCATGAGCGAAGTGAAATTGCATTGAATTTCAAGGGAATTTAGTATGTAAATCGTGCCTTAGAAACAC
ATCTGTTGTCTTTTCTGTGTTTGGTCGATATTAATAATGGCAAAATTTTTGCCTATCTAGTATCTTCAAATTGTAGTCTTTGTAACAACCAAATAACCTTTT
GTGGTCACTGTAAAATTAATATTTGGTAGACAGAATCCATGTACCTTTGCTAAGGTTAGAATGAATAATTTATTGTATTTTTAATTTGAATGTTTGTGCTTT
TTAAATGAGCCAAGACTAGAGGGGAAACTATCACCTAAAATCAGTTTGGAAAACAAGACCTAAAAAGGGAAGGGGATGGGGATTGTGGGGAGAGAG
TGGGCGAGGTGCCTTTACTACATGTGTGATCTGAAAACCCTGCTTGGTTCTGAGCTGCGTCTATTGAATTGGTAAAGTAATACCAATGGCTTTTTATCAT
TTCCTTCTTCCCTTTAAGTTTCACTTGAAATTTTAAAAATCATGGTTATTTTTATCGTTGGGATCTTTCTGTCTTCTGGGTTCCATTTTTTAAATGTTTAAAA
ATATGTTGACATGGTAGTTCAGTTCTTAACCAATGACTTGGGGATGATGCAAACAATTACTGTCGTTGGGATTTAGAGTGTATTAGTCACGCATGTATGG
GGAAGTAGTCTCGGGTATGCTGTTGTGAAATTGAAACTGTAAAAGTAGATGGTTGAAAGTACTGGTATGTTGCTCTGTATGGTAAGAACTAATTCTGTT
ACGTCATGTACATAATTACTAATCACTTTTCTTCCCCTTTACAGC
>ENST00000642367.1|ENSG00000245532.8|OTTHUMG00000166321.7|OTTHUMT00000494372.1|RP11-867O8.8-007|NEAT1|1145|
GCGGCACCTAGCATGTTTGACAGGCGGGGACTGCGAGGCACGCTGCTCGGGTGTTGGGGACAACATTGACCAACGCTTTATTTTCCAGGTGGCAGTG
CTCCTTTTGGACTTTTCTCTAGGTTTGGCGCTAAACTCTTCTTTGTTCCTCATGGCGAGCAGATGGAACCGGGAGACATGGAGTCCCTGGCCAGTGTGA
GTCCTAGCATTGCAGGAGGGGAGACCCTGGAGGAGAGAGCCCGCCTCAATTGATGCCTGCAGATTGAATTTCCAGAGGCTTAGGAGGAGGAAGTTCT
CCAATGTTCTGTTTCCAGGCCTTGCTCAGGAAGCCCTGTATTCAGGAGGCTACCATTTAAAGTTTGCAGATGAGCTTATGGGGGGCAATCTTAAAAAGT
CCACAGCAGATGCATCCGGCTCGAGGGGCCATCAGCTTTGAATAAATGCTTGTTCCAGAGCCCATGAATGCCAGCAGGCACCCCTCCTTTCCTGGGGT
AAAGGTTTTCAGATGCTGCATCTTCTAAATTGAGCCTCCGGTCATACTAGTTTTGTGCTTGGAACCTTGCTTCAAGAAGATCCCTAAGCTGTAGAACATT
TTAACGTTGATGCCACAACGCAGATTGATGCCTTGTAGATGGAGCTTGCAGATGGAGCCCCGTGACCTCTCACCTACCCACCTGTTTGCCTGCCTTCTT
GTGCGTTTCTCGGAGAAGTTCTTAGCCTGATGAAATAACTTGGGGCGTTGAAGAGCTGTTTAATTTTAAATGCCTTAGACTGGGGATATATTAGAGGAA
GCAGATTGTCAAATTAAGGGTGTCATTGTGTTGTGCTAAACGCTGGGAGGGTACAAGTTGGTCATTCCTAAATCTGTGTGTGAGAAATGGCAGGTCTAG
TTTGGGCATTGTGATTGCATTGCAGATTACTAGGAGAAGGGAATGGTGGGTACACCGGTAGTGCTCTTTTGTTCTTGCTTCGTTTTTTTAAACTTGAACT
TTACTTCGTTAGATTTCATAATACTTTCTTGGCATTCTAGTAAGAGGACCCTGAGGTGGGAGTTGTGGGGGACGGGGAGAAGGGGACAGCTTGGCACC
GGTCCCGTGGGCGTTGCAGTGTGGGGGATGGGGGTATGCAGCTTGGCACTGGTACTGGGA
>ENST00000616315.2|ENSG00000245532.8|OTTHUMG00000166321.7|OTTHUMT00000473554.2|RP11-867O8.8-004|NEAT1|512|
TGGAAGTCTTAGAAAAGCCTTGTAAATGCCTATATTGTGGGCTTTTAACGTATTTAAGGGACCACTTAAGACGAGATTAGATGGGCTCTTCTGGATTTGT
TCCTCATTTGTCACAGGTGTCTTGTGATTGAAAATCATGAGCGAAGTGAAATTGCATTGAATTTCAAGGGAATTTAGTATGTAAATCGTGCCTTTACTAC
ATGTGTGATCTGAAAACCCTGCTTGGTTCTGAGCTGCGTCTATTGAATTGGTAAAGTAATACCAATGGCTTTTTATCATTTCCTTCTTCCCTTTAAGTTTC

ACTTGAAATTTTAAAAATCATGGTTATTTTTATCGTTGGGATCTTTCTGTCTTCTGGGTTCCATTTTTTAAATGTTTAAAAATATGTTGACATGGTAGTTCA
GTTCTTAACCAATGACTTGGGGATGATGCAAACAATTACTGTCGTTGGGATTTAGAGTGTATTAGTCACGCATGTATGGGGAAGTAGTCTCGGGTATGCT
GTTGTGAAA

>SNHG5

>ENST00000623163.3|ENSG00000203875.10|OTTHUMG00000015144.5|OTTHUMT00000479176.1|RP11-33E24.2-019|SNHG5|851|

GGAGTGTGACATTTCTGCGAGAATGCTTAAATACCGATTTCCCGCAGGAACAATGGCGCTGTCTTCAGTGGCACAGTGGAGCAGCTCTGAAGATGCAA
AGATACACGAAAAAACTTCCAGAACATCTGGGAGAATATTTAATGGAAAATCGCTTGGTTAAAACCTGACACTTTTAACAGTGAGGGTCGCTGTCTGC
CCATTGATAGAGGCCAGATTGTCTTGGAAGTTCCAAAGTTGCAACGATTTCTTTAGTTGAATCCATGGACATGAAACCCACAGATATAGAGGGTTGGCC
CGGTGCAGTGGCTCACGCCTGTAATCCCAGCACTTTGGGAGGCCAAGGCGGGTGGATCACCTGAGGTCAGAAGCTTGAGACCAGCCTGGCCAACATG
GGTACTGCTTTCTCTTGCTGCAGCTTGCTTCCTCCCTCAGGTATGAAGTTAAGCTACTCGGGAGGCTGAGGCAGGAGAATCGCTTGAACCCGGGAGGC
GGAGGTTGCCATGAGCCGAGATTGCGCCACTACACTCCAGCCTGGGCAACAAAAGCGAAACTCCATCTCAAAAAAAAAAAAAAAGGCATATAAAATTA
TAGCTGGGTAGGAGGAATAAGTTCTAGTGTTCTACACCACTGTGGGATGACTATAGTTAACAATAATACATACTTTCAGGTAACATGAAGGAGGATATTG
AATGTTCCTAACACAAAGAAATGATAAATGTTTGAGATGGTGGATGTACTAATTGCCCTGATCTGATGACTATACATTATATATATTGAAACATCAGTATGT
ACCCCATGAATATGTACAATTATATGTCAACTTAAACACATTTAAAAGTAAAAAAAAAAAAA

>ENST00000623901.3|ENSG00000203875.10|OTTHUMG00000015144.5|OTTHUMT00000479177.1|RP11-33E24.2-020|SNHG5|711|

GGAGTGTGACATTTCTGCGAGAATGCTTAAATACCGATTTCCCGCAGGAACAATGGCGCTGTCTTCAGTGGCACAGTGGAGCAGCTCTGAAGATGCAA
AGATACACGAAAAAACTTCCAGAACATCTGGGAGAATATTTAATGGAAAATCGCTTGGTTAAAACCTGACACTTTTAACAGTGAACAGCGTTCTGAGT
GTGGACGAGTAGCCAGTGAAGATAATGAATGTCGAATGTGACTGACTAGCAGCTTCATTTTGAATGAGGGTCGCTGTCTGCCCATTGATAGAGGCCAGA
TTGTCTTGGAAGTTCCAAAGTTGCAACGATTTCTTTAGTTGAATCCATGGACATGAAACCCACAGATATAGAGGGTTGGCCCGGTGCAGTGGCTCACGC
CTGTAATCCCAGCACTTTGGGAGGCCAAGGCGGGTGGATCACCTGAGGTCAGAAGCTTGAGACCAGCCTGGCCAACATGGGTACTGCTTTCTCTTGCT
GCAGCTTGCTTCCTCCCTCAGGTATGAAGTTAAGGTAACATGAAGGAGGATATTGAATGTTCCTAACACAAAGAAATGATAAATGTTTGAGATGGTGGA
TGTACTAATTGCCCTGATCTGATGACTATACATTATATATATTGAAACATCAGTATGTACCCCATGAATATGTACAATTATATGTCAACTTAAACACATTTAA
AAGTAAAAAAAAAAAAAA

>ENST00000589187.5|ENSG00000203875.10|OTTHUMG00000015144.5|OTTHUMT00000458633.2|RP11-33E24.2-011|SNHG5|568|

CTTTTACGTCGGCCTTCGCGAGCGTCTGGGCGGGTGGTAGGAACAATGGCGCTGTCTTCAGTGGCACAGTGGAGCAGCTCTGAAGATGCAAAGATAC
ACGAAAAAACTTCCAGAACATCTGGGAGAATATTTAATGGAAAATCGCTTGGTTAAAACCTGACACTTTTAACAGTGAGGGTCGCTGTCTGCCCATTGA

TAGAGGCCAGATTGTCTTGGAAGTTCCAAAGTTGCAACGATTTCTTTAGTTGAATCCATGGACATGAAACCCACAGATATAGAGGGTTGGCCCGGTGCA
GTGGCTCACGCCTGTAATCCCAGCACTTTGGGAGGCCAAGGCGGGTGGATCACCTGAGGTCAGAAGCTTGAGACCAGCCTGGCCAACATGGGTACTG
CTTTCTCTTGCTGCAGCTTGCTTCCTCCCTCAGGTAACATGAAGGAGGATATTGAATGTTCCTAACACAAAGAAATGATAAATGTTTGAGATGGTGGATG
TACTAATTGCCCTGATCTGATGACTATACATTATATATATTGAAACATCAGTATGTACCCCATGAATATGTACAAT
>ENST00000622963.3|ENSG00000203875.10|OTTHUMG00000015144.5|OTTHUMT00000479178.1|RP11-33E24.2-022|SNHG5|753|
GGTCTTTTACGTCGGCCTTCGCGAGCGTCTGGGCGGGTGGTAGGAACAATGGCGCTGTCTTCAGTGGCACAGTGGAGCAGCTCTGAAGATGCAAAGT
GAGGGTCGCTGTCTGCCCATTGATAGAGGCCAGATTGTCTTGGAAGTTCCAAAGTTGCAACGATTTCTTTAGTTGAATCCATGGACATGAAACCCACAG
ATATAGAGGGTTGGCCCGGTGCAGTGGCTCACGCCTGTAATCCCAGCACTTTGGGAGGCCAAGGCGGGTGGATCACCTGAGGTCAGAAGCTTGAGAC
CAGCCTGGCCAACATGGTAAAACCCCATCTCTACTAATAATACAAAACAATTTGCTGGGCATGGTGGTGCACGCTTGTAATCCAAGCTACTCCAGAGGC
TGAGGCAGAAGAATCGCTTGAATCCGAGAGGCAAAGGTTGCAGTGAGCCCAGATCACACCACTGCTCTCCAACCTGGGCAACAAGAGCAAAACTCC
GTCAAAAAAAAAAGATACAGGGTCAACTGTACACATTAAACACTTCTATCAACTAGTGATGTCAGTCTTGTCCATTGTATTATTGCCTATCTTACTAAAC
AGTCATATTTCAGGAAGTACAATATACTCAAAAACTAAGTTAATGTTCTTTGTAGATTTATGGTTAATGGATGATTCATATAATAGAAACTTAAATTTATTT
GACCATTCTATTTAATATAGAATAAATACAACTAGCCAGGCATGGTGGCTCATGTCTATAATG
>ENST00000623650.3|ENSG00000203875.10|OTTHUMG00000015144.5|OTTHUMT00000479179.1|RP11-33E24.2-018|SNHG5|886|
GGCGGCGGAAAAACCACCTTAATTGGGGCGGAGGGTTAGTTTTAACAGCAAAGGGCCTTTACTAAAATGGCGAAGGCCTTCCGTCGGCGTTGTTTTAA
AATGGGAAGCCTCGACCCTGTATTGAAACTGAGCTGTTCGAAGGCGGCGTTGTGTGCAATTCGGATTAATGAAGGGGAAGGGTTTTGTGTGGAAAAA
CGCCTTGGAGTGTGACATTTCTGCGAGAATGCTTAAATACCGATTTCCCGCAGGAACAATGGCGCTGTCTTCAGTGGCACAGTGGAGCAGCTCTGAAG
ATGCAAAGATACACGAAAAAACTTCCAGAACATCTGGGAGAATATTTAATGGAAAATCGCTTGGTTAAAACCTGACACTTTTAACAGTTAGTTGAATCC
ATGGACATGAAACCCACAGATATAGAGGGTTGGCCCGGTGCAGTGGCTCACGCCTGTAATCCCAGCACTTTGGGAGGCCAAGGCGGGTGGATCACCT
GAGGTCAGAAGCTTGAGACCAGCCTGGCCAACATGGTAAAACCCCATCTCTACTAATAATACAAAACAATTTGCTGGGCATGGTGGTGCACGCTTGTA
ATCCAAGCTACTCCAGAGGCTGAGGCAGAAGAATCGCTTGAATCCGAGAGGCAAAGGTTGCAGTGAGCCCAGATCACACCACTGCTCTCCAACCTGG
GCAACAAGAGCAAAACTCCGTCAAAAAAAAAAGATACAGGGTCAACTGTACACATTAAACACTTCTATCAACTAGTGATGTCAGTCTTGTCCATTGTA
TTATTGCCTATCTTACTAAACAGTCATATTTCAGGAAGTACAATATACTCAAAAACTAAGTTAATGTTCTTTGTAGATTTATGGTTAATGGATGATTCATATA
A
>ENST00000624128.1|ENSG00000203875.10|OTTHUMG00000015144.5|OTTHUMT00000480.1|RP11-33E24.2-021|SNHG5|672|
CGGAAAAACCACCTTAATTGGGGCGGAGGGTTAGTTTTAACAGCAAAGGGCCTTTACTAAAATGGCGAAGGCCTTCCGTCGGCGTTGTTTTAAAATGG

GAAGCCTCGACCCTGTATTGAAACTGAGCTGTTCGAAGGCGGCGTTGTGTGCAATTCGGATTAATGAAGGGGAAGGGTTTTGTGTGGAAAAACGCCTT
GGAGTGTGACATTTCTGCGAGAATGCTTAAATACCGATTTCCCGCAGGAACAATGGCGCTGTCTTCAGTGGCACAGTGGAGCAGCTCTGAAGATGCAA
AGTTAGTTGAATCCATGGACATGAAACCCACAGATATAGAGGGTTGGCCCGGTGCAGTGGCTCACGCCTGTAATCCCAGCACTTTGGGAGGCCAAGGC
GGGTGGATCACCTGAGGTCAGAAGCTTGAGACCAGCCTGGCCAACATGGTAAAACCCCATCTCTACTAATAATACAAAACAATTTGCTGGGCATGGTG
GTGCACGCTTGTAATCCAAGCTACTCCAGAGGCTGAGGCAGAAGAATCGCTTGAATCCGAGAGGCAAAGGTTGCAGTGAGCCCAGATCACACCACTG
CTCTCCAACCTGGGCAACAAGAGCAAAACTCCGTCAAAAAAAAAAGATACAGGGTCAACTGTACACATTAAACACTTCTATCAAC
>ENST00000587692.5|ENSG00000203875.10|OTTHUMG00000015144.5|OTTHUMT00000458634.1|RP11-33E24.2-012|SNHG5|1029|
CTTCGCGAGCGTCTGGGCGGGTGGTAGGTGAGTGGGTATTGCGGGCTAGTATCCGAGCAAAAGATGGTGGCGCAGGCCGAGTTAAGAGCTTTAATCCT
GTGAAGACATCTTAGTGAAGAGTTTAGAGTGCTGAGAGTTGAAAGCTTGCACGTGGGAAACGTGCGGCCGGACTGCCACATGTACTGAGGTTGAGTC
GTGACGGCCACAGGCTCCGAGTTTTGGCGTGAGGAACCGCTGATCGGCCACGGGCGCCGAACTTGCTGGCCTCCGGCATGTGCCTGAGCGGCGGCGG
AAAAACCACCTTAATTGGGGCGGAGGGTTAGTTTTAACAGCAAAGGGCCTTTACTAAAATGGCGAAGGCCTTCCGTCGGCGTTGTTTTAAAATGGGAA
GCCTCGACCCTGTATTGAAACTGAGCTGTTCGAAGGCGGCGTTGTGTGCAATTCGGATTAATGAAGGGGAAGGGTTTTGTGTGGAAAAACGCCTTGGA
GTGTGACATTTCTGCGAGAATGCTTAAATACCGATTTCCCGCAGGAACAATGGCGCTGTCTTCAGTGGCACAGTGGAGCAGCTCTGAAGATGCAAAGA
TACACGAAAAAACTTCCAGAACATCTGGGAGAATATTTAATGGAAAATCGCTTGGTTAAAACCTGACACTTTTAACAGTGAGGGTCGCTGTCTGCCCAT
TGATAGAGGCCAGATTGTCTTGGAAGTTCCAAAGTTGCAACGATTTCTTTAGTTGAATCCATGGACATGAAACCCACAGATATAGAGGGTTGGCCCGGT
GCAGTGGCTCACGCCTGTAATCCCAGCACTTTGGGAGGCCAAGGCGGGTGGATCACCTGAGGTCAGAAGCTTGAGACCAGCCTGGCCAACATGGTAA
AACCCCATCTCTACTAATAATACAAAACAATTTGCTGGGCATGGTGGTGCACGCTTGTAATCCAAGCTACTCCAGAGGCTGAGGCAGAAGAATCGCTTG
AATCCGAGAGGCAAAGGTTGCAGTGAGCCCAGATCACACCACTGCTCTC
>ENST00000369605.8|ENSG00000203875.10|OTTHUMG00000015144.5|OTTHUMT00000041401.4|RP11-33E24.2-001|SNHG5|1032|
GGTCTTTTACGTCGGCCTTCGCGAGCGTCTGGGCGGGTGGTAGGTGAGTGGGTATTGCGGGCTAGTATCCGAGCAAAAGATGGTGGCGCAGGCCGAGT
TAAGAGCTTTAATCCTGTGAAGACATCTTAGTGAAGAGTTTAGAGTGCTGAGAGTTGAAAGCTTGCACGTGGGAAACGTGCGGCCGGACTGCCACAT
GTACTGAGGTTGAGTCGTGACGGCCACAGGCTCCGAGTTTTGGCGTGAGGAACCGCTGATCGGCCACGGGCGCCGAACTTGCTGGCCTCCGGCATGT
GCCTGAGCGGCGGCGGAAAAACCACCTTAATTGGGGCGGAGGGTTAGTTTTAACAGCAAAGGGCCTTTACTAAAATGGCGAAGGCCTTCCGTCGGCG
TTGTTTTAAAATGGGAAGCCTCGACCCTGTATTGAAACTGAGCTGTTCGAAGGCGGCGTTGTGTGCAATTCGGATTAATGAAGGGGAAGGGTTTTGTG
TGGAAAAACGCCTTGGAGTGTGACATTTCTGCGAGAATGCTTAAATACCGATTTCCCGCAGGAACAATGGCGCTGTCTTCAGTGGCACAGTGGAGCAG
CTCTGAAGATGCAAAGATACACGAAAAAACTTCCAGAACATCTGGGAGAATATTTAATGGAAAATCGCTTGGTTAAAACCTGACACTTTTAACAGTGA

ACAGCGTTCTGAGTGTGGACGAGTAGCCAGTGAAGATAATGAATGTCGAATGTGACTGACTAGCAGCTTCATTTTGAATGAGGGTCGCTGTCTGCCCA
TTGATAGAGGCCAGATTGTCTTGGAAGTTCCAAAGTTGCAACGATTTCTGGCTAGTGCCACGAGGTTTACTTGACTGTTGTGTGAAAAGCTGATAAGA
AAACCATCCAGAAAAAAGCTCTTCGTTTTACAAACATGAAAATAAAACATGTAATTTTGGATTATGTTCCTTTTTGTTATTACTTTTAAATAGGTCCTGAA
ATAACATGGGGAGCATTAAATGGAAAATCCACTAACCAGCCTTGTAATCAAA
>ENST00000427501.5|ENSG00000203875.10|OTTHUMG00000015144.5|OTTHUMT00000041405.1|RP11-33E24.2-005|SNHG5|680|
AATGGAAAATCGCTTGGTTAAAACCTGACACTTTTAACAGGTATGTGTTGTTTTAGTACTTTATGATTGAGCATAGCATTTAATCCACACCTAGACTAAAT
CAAATTTTTTTTGTCAGTGAACAGCGTTCTGAGTGTGGACGAGTAGCCAGTGAAGATAATGAATGTCGAATGTGACTGACTAGCAGCTTCATTTTGAAG
TAGGTTGTATGGCTTAAAAGTTCTGTAGTATTTGTACTATAATACTTGCCTTTTAGCATTACCTTGGTTTGTAGTCAGTGTCACAGAAGTGCAGTTTAATG
TATTATGTGTACATATACAAGGTCTGATTGGTCTAATCAATGATGAAACCTATCCCGAAGCTGATAACCTGAAGAAAAATAAGTACGGATTCGGCTTCTG
AGATTAAGACCAGTAATTCAGAGGTGGAGTAAATTTTGTTGCCGTGATTTTATAACAGTTGTGTTATAAAATCCTGGGTTTTTTTTTTTTCTGCCAACAGT
GAGGGTCGCTGTCTGCCCATTGATAGAGGCCAGATTGTCTTGGAAGTTCCAAAGTTGCAACGATTTCTGGCTAGTGCCACGAGGTTTACTTGACTGTTG
TGTGAAAAGCTGATAAGAAAACCATCCAGAAAAAAGCTCTTCGTTTTACAAACATGAAAATAAAACATGTAATTTTGGA
>ENST00000435947.5|ENSG00000203875.10|OTTHUMG00000015144.5|OTTHUMT00000041403.1|RP11-33E24.2-003|SNHG5|696|
GGCGCAGGCCGAGTTAAGAGCTTTAATCCTGTGAAGACATCTTAGTGAAGAGTTTAGAGTGCTGAGAGTTGAAAGCTTGCACGTGGGAAACGTGCGG
CCGGACTGCCACATGTACTGAGGTTGAGTCGTGACGGCCACAGGCTCCGAGTTTTGGCGTGAGGAACCGCTGATCGGCCACGGGCGCCGAACTTGCT
GGCCTCCGGCATGTGCCTGAGCGGCGGCGGAAAAACCACCTTAATTGGGGCGGAGGGTTAGTTTTAACAGCAAAGGGCCTTTACTAAAATGGCGAAG
GCCTTCCGTCGGCGTTGTTTTAAAATGGGAAGCCTCGACCCTGTATTGAAACTGAGCTGTTCGAAGGCGGCGTTGTGTGCAATTCGGATTAATGAAGG
GGAAGGGTTTTGTGTGGAAAAACGCCTTGGAGTGTGACATTTCTGCGAGAATGCTTAAATACCGATTTCCCGCAGGAACAATGGCGCTGTCTTCAGTG
GCACAGTGGAGCAGCTCTGAAGATGCAAAGTGAGGGTCGCTGTCTGCCCATTGATAGAGGCCAGATTGTCTTGGAAGTTCCAAAGTTGCAACGATTTC
TGGCTAGTGCCACGAGGTTTACTTGACTGTTGTGTGAAAAGCTGATAAGAAAACCATCCAGAAAAAAGCTCTTCGTTTTACAAACATGAAAATAAAAC
ATGTAATTTTGGA
>ENST00000624295.3|ENSG00000203875.10|OTTHUMG00000015144.5|OTTHUMT00000479181.1|RP11-33E24.2-015|SNHG5|632|
AGGGTTAGTTTTAACAGCAAAGGGCCTTTACTAAAATGGCGAAGGCCTTCCGTCGGCGTTGTTTTAAAATGGGAAGCCTCGACCCTGTATTGAAACTG
AGCTGTTCGAAGGCGGCGTTGTGTGCAATTCGGATTAATGAAGGGGAAGGGTTTTGTGTGGAAAAACGCCTTGGAGTGTGACATTTCTGCGAGAATGC
TTAAATACCGATTTCCCGCAGGAACAATGGCGCTGTCTTCAGTGGCACAGTGGAGCAGCTCTGAAGATGCAAAGATACACGAAAAAACTTCCAGAAC
ATCTGGGAGAATATTTAATGGAAAATCGCTTGGTTAAAACCTGACACTTTTAACAGACTAAATCAAATTTTTTTTGTCAGTGAACAGCGTTCTGAGTGT

GGACGAGTAGCCAGTGAAGATAATGAATGTCGAATGTGACTGACTAGCAGCTTCATTTTGAATGAGGGTCGCTGTCTGCCCATTGATAGAGGCCAGATT
GTCTTGGAAGTTCCAAAGTTGCAACGATTTCTGGCTAGTGCCACGAGGTTTACTTGACTGTTGTGTGAAAAGCTGATAAGAAAACCATCCAGAAAAAA
GCTCTTCGTTTTACAAACATGAAAATAAAACATGTAATTTTGG
>ENST00000625175.3|ENSG00000203875.10|OTTHUMG00000015144.5|OTTHUMT00000479182.1|RP11-33E24.2-017|SNHG5|257|
ATACCGATTTCCCGCAGGAACAATGGCGCTGTCTTCAGTGGCACAGTGGAGCAGCTCTGAAGATGCAAAGATACACGAAAAAACTTCCAGAACATCT
GGGAGAATATTTAATGGAAAATCGCTTGGTTAAAACCTGACACTTTTAACAGGGCTAGTGCCACGAGGTTTACTTGACTGTTGTGTGAAAAGCTGATAA
GAAAACCATCCAGAAAAAAGCTCTTCGTTTTACAAACATGAAAATAAAACATGTAATTTTG
>ENST00000623910.3|ENSG00000203875.10|OTTHUMG00000015144.5|OTTHUMT00000479183.1|RP11-33E24.2-013|SNHG5|631|
GGCGGCGGAAAAACCACCTTAATTGGGGCGGAGGGTTAGTTTTAACAGCAAAGGGCCTTTACTAAAATGGCGAAGGCCTTCCGTCGGCGTTGTTTTAA
AATGGGAAGCCTCGACCCTGTATTGAAACTGAGCTGTTCGAAGGCGGCGTTGTGTGCAATTCGGATTAATGAAGGGGAAGGGTTTTGTGTGGAAAAA
CGCCTTGGAGTGTGACATTTCTGCGAGAATGCTTAAATACCGATTTCCCGCAGGAACAATGGCGCTGTCTTCAGTGGCACAGTGGAGCAGCTCTGAAG
ATGCAAAGATACACGAAAAAACTTCCAGAACATCTGGGAGAATATTTAATGGAAAATCGCTTGGTTAAAACCTGACACTTTTAACAGCGTTCTGAGTG
TGGACGAGTAGCCAGTGAAGATAATGAATGTCGAATGTGACTGACTAGCAGCTTCATTTTGAATGAGGGTCGCTGTCTGCCCATTGATAGAGGCCAGAT
TGTCTTGGAAGTTCCAAAGTTGCAACGATTTCTGGCTAGTGCCACGAGGTTTACTTGACTGTTGTGTGAAAAGCTGATAAGAAAACCATCCAGAAAAA
AGCTCTTCGTTTTACAAACATGAAAATAAAACATGTAATTTTG
>ENST00000428833.5|ENSG00000203875.10|OTTHUMG00000015144.5|OTTHUMT00000041404.2|RP11-33E24.2-004|SNHG5|641|
GGCCACAGGCTCCGAGTTTTGGCGTGAGGAACCGCTGATCGGCCACGGGCGCCGAACTTGCTGGCCTCCGGCATGTGCCTGAGCGGCGGCGGAAAA
ACCACCTTAATTGGGGCGGAGGGTTAGTTTTAACAGCAAAGGGCCTTTACTAAAATGGCGAAGGCCTTCCGTCGGCGTTGTTTTAAAATGGGAAGCCT
CGACCCTGTATTGAAACTGAGCTGTTCGAAGGCGGCGTTGTGTGCAATTCGGATTAATGAAGGGGAAGGGTTTTGTGTGGAAAAACGCCTTGGAGTGT
GACATTTCTGCGAGAATGCTTAAATACCGATTTCCCGCAGGAACAATGGCGCTGTCTTCAGTGGCACAGTGGAGCAGCTCTGAAGATGCAAAGATACA
CGAAAAAACTTCCAGAACATCTGGGAGAATATTTAATGGAAAATCGCTTGGTTAAAACCTGACACTTTTAACAGTGAGGGTCGCTGTCTGCCCATTGAT
AGAGGCCAGATTGTCTTGGAAGTTCCAAAGTTGCAACGATTTCTGGCTAGTGCCACGAGGTTTACTTGACTGTTGTGTGAAAAGCTGATAAGAAAACC
ATCCAGAAAAAAGCTCTTCGTTTTACAAACATGAAAATAAAACATGTAATTTTG
>ENST00000453754.5|ENSG00000203875.10|OTTHUMG00000015144.5|OTTHUMT00000041406.1|RP11-33E24.2-006|SNHG5|401|
CACGTGGGAAACGTGCGGCCGGACTGCCACATGTACTGAGGTTGAGTCGTGACGGCCACAGGCTCCGAGTTTTGGCGTGAGGAACCGCTGATCGGCC
ACGGGCGCCGAACTTGCTGGCCTCCGGCATGTGCCTGAGCGGCGGCGGAAAAACCACCTTAATTGGGGCGGAGGGAACAATGGCGCTGTCTTCAGTG

GCACAGTGGAGCAGCTCTGAAGATGCAAAGTGAGGGTCGCTGTCTGCCCATTGATAGAGGCCAGATTGTCTTGGAAGTTCCAAAGTTGCAACGATTTC
TGGCTAGTGCCACGAGGTTTACTTGACTGTTGTGTGAAAAGCTGATAAGAAAACCATCCAGAAAAAAGCTCTTCGTTTTACAAACATGAAAATAAAAC
ATGTAATTTTG
>ENST00000420199.5|ENSG00000203875.10|OTTHUMG00000015144.5|OTTHUMT00000314640.1|RP11-33E24.2-010|SNHG5|1002|
GCCTTCGCGAGCGTCTGGGCGGGTGGTAGGTGAGTGGGTATTGCGGGCTAGTATCCGAGCAAAAGATGGTGGCGCAGGCCGAGTTAAGAGCTTTAATC
CTGTGAAGACATCTTAGTGAAGAGTTTAGAGTGCTGAGAGTTGAAAGCTTGCACGTGGGAAACGTGCGGCCGGACTGCCACATGTACTGAGGTTGAG
TCGTGACGGCCACAGGCTCCGAGTTTTGGCGTGAGGAACCGCTGATCGGCCACGGGCGCCGAACTTGCTGGCCTCCGGCATGTGCCTGAGCGGCGGC
GGAAAAACCACCTTAATTGGGGCGGAGGGTTAGTTTTAACAGCAAAGGGCCTTTACTAAAATGGCGAAGGCCTTCCGTCGGCGTTGTTTTAAAATGGG
AAGCCTCGACCCTGTATTGAAACTGAGCTGTTCGAAGGCGGCGTTGTGTGCAATTCGGATTAATGAAGGGGAAGGGTTTTGTGTGGAAAAACGCCTTG
GAGTGTGACATTTCTGCGAGAATGCTTAAATACCGATTTCCCGCAGGAACAATGGCGCTGTCTTCAGTGGCACAGTGGAGCAGCTCTGAAGATGCAAA
GATACACGAAAAAACTTCCAGAACATCTGGGAGAATATTTAATGGAAAATCGCTTGGTTAAAACCTGACACTTTTAACAGGTATGTGTTGTTTTAGTAC
TTTATGATTGAGCATAGCATTTAATCCACACCTAGACTAAATCAAATTTTTTTTGTCAGTGAACAGCGTTCTGAGTGTGGACGAGTAGCCAGTGAAGATA
ATGAATGTCGAATGTGACTGACTAGCAGCTTCATTTTGAATGAGGGTCGCTGTCTGCCCATTGATAGAGGCCAGATTGTCTTGGAAGTTCCAAAGTTGC
AACGATTTCTGGCTAGTGCCACGAGGTTTACTTGACTGTTGTGTGAAAAGCTGATAAGAAAACCATCCAGAAAAAAGCTCTTCGTTTTACAAACATGA
AAATAAAACATGTAATTTTG
>ENST00000414002.5|ENSG00000203875.10|OTTHUMG00000015144.5|OTTHUMT00000041402.2|RP11-33E24.2-002|SNHG5|430|
CTTTTACGTCGGCCTTCGCGAGCGTCTGGGCGGGTGGTAGGAACAATGGCGCTGTCTTCAGTGGCACAGTGGAGCAGCTCTGAAGATGCAAAGATAC
ACGAAAAAACTTCCAGAACATCTGGGAGAATATTTAATGGAAAATCGCTTGGTTAAAACCTGACACTTTTAACAGTGAACAGCGTTCTGAGTGTGGAC
GAGTAGCCAGTGAAGATAATGAATGTCGAATGTGACTGACTAGCAGCTTCATTTTGAATGAGGGTCGCTGTCTGCCCATTGATAGAGGCCAGATTGTCT
TGGAAGTTCCAAAGTTGCAACGATTTCTGGCTAGTGCCACGAGGTTTACTTGACTGTTGTGTGAAAAGCTGATAAGAAAACCATCCAGAAAAAAGCTC
TTCGTTTTACAAACATGAAAATAAAACATGTAATTTTG
>ENST00000623267.3|ENSG00000203875.10|OTTHUMG00000015144.5|OTTHUMT00000479184.1|RP11-33E24.2-016|SNHG5|374|
TGGGGCGGAGGGTTAGTTTTAACAGCAAAGGGCCTTTACTAAAATGGCGAAGGCCTTCCGTCGGCGTTGTTTTAAAATGGGAAGCCTCGACCCTGTAT
TGAAACTGAGCTGTTCGAAGGCGGCGTTGTGTGCAATTCGGATTAATGAAGGGGAAGGGTTTTGTGTGGAAAAACGCCTTGGAGTGTGACATTTCTGC
GAGAATGCTTAAATACCGATTTCCCGCAGGAACAATGGCGCTGTCTTCAGTGGCACAGTGGAGCAGCTCTGAAGATGCAAAGGGCTAGTGCCACGAG
GTTTACTTGACTGTTGTGTGAAAAGCTGATAAGAAAACCATCCAGAAAAAAGCTCTTCGTTTTACAAACATGAAAATAAAA

>ENST00000623001.3|ENSG00000203875.10|OTTHUMG00000015144.5|OTTHUMT00000479185.1|RP11-33E24.2-014|SNHG5|598|
TGGGGCGGAGGGTTAGTTTTAACAGCAAAGGGCCTTTACTAAAATGGCGAAGGCCTTCCGTCGGCGTTGTTTTAAAATGGGAAGCCTCGACCCTGTAT
TGAAACTGAGCTGTTCGAAGGCGGCGTTGTGTGCAATTCGGATTAATGAAGGGGAAGGGTTTTGTGTGGAAAAACGCCTTGGAGTGTGACATTTCTGC
GAGAATGCTTAAATACCGATTTCCCGCAGGAACAATGGCGCTGTCTTCAGTGGCACAGTGGAGCAGCTCTGAAGATGCAAAGATACACGAAAAAACT
TCCAGAACATCTGGGAGAATATTTAATGGAAAATCGCTTGGTTAAAACCTGACACTTTTAACAGTGAACAGCGTTCTGAGTGTGGACGAGTAGCCAGT
GAAGATAATGAATGTCGAATGTGACTGACTAGCAGCTTCATTTTGAAGTAGTGAGGGTCGCTGTCTGCCCATTGATAGAGGCCAGATTGTCTTGGAAGT
TCCAAAGTTGCAACGATTTCTGGCTAGTGCCACGAGGTTTACTTGACTGTTGTGTGAAAAGCTGATAAGAAAACCATCCAGAAAAAAGCTCTTCGTTT
TACAAACATG
>ENST00000431043.1|ENSG00000203875.10|OTTHUMG00000015144.5|OTTHUMT00000314639.1|RP11-33E24.2-009|SNHG5|998|
CTTTTACGTCGGCCTTCGCGAGCGTCTGGGCGGGTGGTAGGAACAATGGCGCTGTCTTCAGTGGCACAGTGGAGCAGCTCTGAAGATGCAAAGATAC
ACGAAAAAACTTCCAGAACATCTGGGAGAATATTTAATGGAAAATCGCTTGGTTAAAACCTGACACTTTTAACAGGTATGTGTTGTTTTAGTACTTTATG
ATTGAGCATAGCATTTAATCCACACCTAGACTAAATCAAATTTTTTTTGTCAGTGAACAGCGTTCTGAGTGTGGACGAGTAGCCAGTGAAGATAATGAA
TGTCGAATGTGACTGACTAGCAGCTTCATTTTGAAGTAGGTTGTATGGCTTAAAAGTTCTGTAGTATTTGTACTATAATACTTGCCTTTTAGCATTACCTT
GGTTTGTAGTCAGTGTCACAGAAGTGCAGTTTAATGTATTATGTGTACATATACAAGGTCTGATTGGTCTAATCAATGATGAAACCTATCCCGAAGCTGAT
AACCTGAAGAAAAATAAGTACGGATTCGGCTTCTGAGATTAAGACCAGTAATTCAGAGGTGGAGTAAATTTTGTTGCCGTGATTTTATAACAGTTGTGT
TATAAAATCCTGGGTTTTTTTTTTTTCTGCCAACAGTGAGGGTCGCTGTCTGCCCATTGATAGAGGCCAGATTGTCTTGGAAGTTCCAAAGTTGCAACG
ATTTCTGTAAGTGGAGTTTTTCTGTTTGCTTAGAGATCAGTGAATATTGTGTCCTTGGTCTTATCTGTGATGATCTTATCCCGAACCTGAACTTCTGTTGA
AAAAAAAAAACTTTTACGGATCTGGCTTCTGAGATGGACCGTTATAAGGACAATATTTTTTTTTAATACTTTTAATGCTTTTACATATGTTGTAATGTTTGT
AGTCTTGTAAGAATCTCGTGTTTTTCCTTTTCTAGGGCTAGTGCCACGAGGTTTACTTGACTGTTGTGTGAAAAGCTGATAAGAAAACCATCCAGAAAA
>ENST00000433843.1|ENSG00000203875.10|OTTHUMG00000015144.5|OTTHUMT00000041407.2|RP11-33E24.2-007|SNHG5|388|
ATTTCCCGCAGGAACAATGGCGCTGTCTTCAGTGGCACAGTGGAGCAGCTCTGAAGATGCAAAGATACACGAAAAAACTTCCAGAACATCTGGGAGA
ATATTTAATGGAAAATCGCTTGGTTAAAACCTGACACTTTTAACAGTGAACAGCGTTCTGAGTGTGGACGAGTAGCCAGTGAAGATAATGAATGTCGAA
TGTGACTGACTAGCAGCTTCATTTTGAATGAGGGTCGCTGTCTGCCCATTGATAGAGGCCAGATTGTCTTGGAAGTTCCAAAGTTGCAACGATTTCTGT
AAGTGGAGTTTTTCTGTTTGCTTAGAGATCAGTGAATATTGTGTCCTTGGTCTTATCTGTGATGATCTTATCCCGAACCTGAACTTCTGTTGA
>ENST00000425170.1|ENSG00000203875.10|OTTHUMG00000015144.5|OTTHUMT00000041408.1|RP11-33E24.2-008|SNHG5|489|
GGGTTTTGTGTGGAAAAACGCCTTGGAGTGTGACATTTCTGCGAGAATGCTTAAATACCGATTTCCCGCAGGAACAATGGCGCTGTCTTCAGTGGCAC

AGTGGAGCAGCTCTGAAGATGCAAAGGTAAGAGCTTAGTTAAGCTTAGTTTCCAAACTAAAGGAGTAAACCTGTTGATTTACAGGAATAGGAACTGTT
GCATCGTTTGAAATTTACTTTTTTTTGTTAGATACACGAAAAAACTTCCAGAACATCTGGGAGAATATTTAATGGAAAATCGCTTGGTTAAAACCTGACA
CTTTTAACAGGTATGTGTTGTTTTAGTACTTTATGATTGAGCATAGCATTTAATCCACACCTAGACTAAATCAAATTTTTTTTGTCAGTGAACAGCGTTCT
GAGTGTGGACGAGTAGCCAGTGAAGATAATGAATGTCGAATGTGACTGACTAGCAGCTTCATTTTGAATGAGGGTCGCTGTCTGCCCATTGA
>H19
>ENST00000442037.5|ENSG00000130600.18|OTTHUMG00000012477.6|OTTHUMT00000142908.4|AC051649.8-015|H19|798|
AACACCTTAGGCTGGTGGGGCTGCGGCAAGAAGCGGGTCTGTTTCTTTACTTCCTCCACGGAGTCGGCACACTATGGCTGCCCTCTGGGCTCCCAGAA
CCCACAACATGAAAGCTTGGAAATGAATATGCTGCACTTTACAACCACTGCACTACCTGACTCAGGAATCGGCTCTGGAAGGTGAAGCTAGAGGAACC
AGACCTCATCAGCCCAACATCAAAGACACCATCGGAACAGCAGCGCCCGCAGCACCCACCCCGCACCGGCGACTCCATCTTCATGGCCACCCCCTGC
GGCGGACGGTTGACCACCAGCCACCACATCATCCCAGAGCTGAGCTCCTCCAGCGGGATGACGCCGTCCCCACCACCTCCCTCTTCTTCTTTTTCATCC
TTCTGTCTCTTTGTTTCTGAGCTTTCCTGTCTTTCCTTTTTTCTGAGAGATTCAAAGCCTCCACGACTCTGTTTCCCCCGTCCCTTCTGAATTTAATTTGC
ACTAAGTCATTTGCACTGGTTGGAGTTGTGGAGACGGCCTTGAGTCTCAGTACGAGTGTGCGTGAGTGTGAGCCACCTTGGCAAGTGCCTGTGCAGG
GCCCGGCCGCCCTCCATCTGGGCCGGGTGACTGGGCGCCGGCTGTGTGCCCGAGGCCTCACCCTGCCCTCGCCTAGTCTGGAAGCTCCGACCGACATC
ACGGAGCAGCCTTCAAGCATTCCATTACGCCCCATCTCGCTCTGTGCCCCTCCCCACCAGGGCTTCAGCAGGAGCCCTGGACTCATCATCAATAAACAC
TGTTACAGCAA
>ENST00000417089.6|ENSG00000130600.18|OTTHUMG00000012477.6|OTTHUMT00000495009.1|AC051649.8-016|H19|1090|
ACAGCATCCAGGGGAGTCAAGGGCATGGGGCGAGACCAGACTAGGCGAGGCGGGCGGGGCGGAGTGAATGAGCTCTCAGGAGGGAGGATGGTGCA
GGCAGGGGTGAGGAGCGCAGCGGGCGGCGAGCGGGAGGCACTGGCCTCCAGAGCCCGTGGCCAAGGCGGGCCTCGCGGGCGGCGACGGAGCCGG
GATCGGTGCCTCAGCGTTCGGGCTGGAGACGAGGCCAGGTCTCCAGCTGGGGTGGACGTGCCCACCAGCTGCCGAAGGCCAAGACGCCAGGTCCGG
TGGACGTGACAAGCAGGACATGACATGGTCCGGTGTGACGGCGAGGACAGAGGAGGCGCGTCCGGCCTTCCTGAACACCTTAGGCTGGTGGGGCTG
CGGCAAGAAGCGGGTCTGTTTCTTTACTTCCTCCACGGAGTCGGCACACTATGGCTGCCCTCTGGGCTCCCAGAACCCACAACATGAAAGGTGAAGCT
AGAGGAACCAGACCTCATCAGCCCAACATCAAAGACACCATCGGAACAGCAGCGCCCGCAGCACCCACCCCGCACCGGCGACTCCATCTTCATGGCC
ACCCCCTGCGGCGGACGGTTGACCACCAGCCACCACATCATCCCAGAGCTGAGCTCCTCCAGCGGGATGACGCCGTCCCCACCACCTCCCTCTTCTTC
TTTTTCATCCTTCTGTCTCTTTGTTTCTGAGCTTTCCTGTCTTTCCTTTTTTCTGAGAGATTCAAAGCCTCCACGACTCTGTTTCCCCCGTCCCTTCTGAA
TTTAATTTGCACTAAGTCATTTGCACTGGTTGGAGTTGTGGAGACGGCCTTGAGTCTCAGTACGAGTGTGCGTGAGTGTGAGCCACCTTGGCAAGTGC
CTGTGCAGGGCCCGGCCGCCCTCCATCTGGGCCGGGTGACTGGGCGCCGGCTGTGTGCCCGAGGCCTCACCCTGCCCTCGCCTAGTCTGGAAGCTCC

GACCGACATCACGGAGCAGCCTTCAAGCATTCCATTACGCCCCATCTCGCTCTGTGCCCCTCCCCACCAGGGCTTCAGCAGGAGCCCTGGACTCATCAT
CAATAAACACTGTTACAGCAA
>ENST00000411861.5|ENSG00000130600.18|OTTHUMG00000012477.6|OTTHUMT00000142897.3|AC051649.8-004|H19|1771|
ATGGGCCCGTTCCAGGCAGAAAGAGCAAGAGGGCAGGGAGGGAGCACAGGGGTGGCCAGCGTAGGGTCCAGCACGTGGGGTGGTACCCCAGGCCT
GGGTCAGACAGGGACATGGCAGGGGACACAGGACAGAGGGGTCCCCAGCTGCCACCTCACCCACCGCAATTCATTTAGTAGCAGGCACAGGGGCAG
CTCCGGCACGGCTTTCTCAGGCCTATGCCGGAGCCTCGAGGGCTGGAGAGCGGGAAGACAGGCAGTGCTCGGGGAGTTGCAGCAGGACGTCACCAG
GAGGGCGAAGCGGCCACGGGAGGGGGGCCCCGGGACATTGCGCAGCAAGGAGGCTGCAGGGGCTCGGCCTGCGGGCGCCGGTCCCACGAGGCACT
GCGGCCCAGGGTCTGGTGCGGAGAGGGCCCACAGTGGACTTGGTGACGCTGTATGCCCTCACCGCTCAGCCCCTGGGGCTGGCTTGGCAGACAGTAC
AGCATCCAGGGGAGTCAAGGGCATGGGGCGAGACCAGACTAGGCGAGGCGGCGGGGCGGAGTGAATGAGCTCTCAGGAGGGAGGATGGTGCAGG
CAGGGGTGAGGAGCGCAGCGGGCGGCGAGCGGGAGGCACTGGCCTCCAGAGCCCGTGGCCAAGGCGGGCCTCGCGGGCGGCGACGGAGCCGGGAT
CGGTGCCTCAGCGTTCGGGCTGGAGACGAGGCCAGGTCTCCAGCTGGGGTGGACGTGCCCACCAGCTGCCGAAGGCCAAGACGCCAGGTCCGGTGG
ACGTGACAAGCAGGACATGACATGGTCCGGTGTGACGGCGAGGACAGAGGAGGCGCGTCCGGCCTTCCTGAACACCTTAGGCTGGTGGGGCTGCGG
CAAGAAGCGGGTCTGTTTCTTTACTTCCTCCACGGAGTCGGCACACTATGGCTGCCCTCTGGGCTCCCAGAACCCACAACATGAAAGAAATGGTGCTA
CCCAGCTCAAGCCTGGGCCTTTGAATCCGGACACAAAACCCTCTAGCTTGGAAATGAATATGCTGCACTTTACAACCACTGCACTACCTGACTCAGGA
ATCGGCTCTGGAAGGTGAGCACCAGCGCTCCTTCCGGAAGCCTCCAGGCCCCCGAGCACCCTGCCCCCATCCCACCCACGTGTCGCTATCTCTAGGTG
AAGCTAGAGGAACCAGACCTCATCAGCCCAACATCAAAGACACCATCGGAACAGCAGCGCCCGCAGCACCCACCCCGCACCGGCGACTCCATCTTCA
TGGCCACCCCCTGCGGCGGACGGTTGACCACCAGCCACCACATCATCCCAGAGCTGAGCTCCTCCAGCGGGATGACGCCGTCCCCACCACCTCCCTCT
TCTTCTTTTTCATCCTTCTGTCTCTTTGTTTCTGAGCTTTCCTGTCTTTCCTTTTTTCTGAGAGATTCAAAGCCTCCACGACTCTGTTTCCCCCGTCCCTTC
TGAATTTAATTTGCACTAAGTCATTTGCACTGGTTGGAGTTGTGGAGACGGCCTTGAGTCTCAGTACGAGTGTGCGTGAGTGTGAGCCACCTTGGCAA
GTGCCTGTGCAGGGCCCGGCCGCCCTCCATCTGGGCCGGGTGACTGGGCGCCGGCTGTGTGCCCGAGGCCTCACCCTGCCCTCGCCTAGTCTGGAAG
CTCCGACCGACATCACGGAGCAGCCTTCAAGCATTCCATTACGCCCCATCTCGCTCTGTGCCCCTCCCCACCAGGGCTTCAGCAGGAGCCCTGGACTC
ATCATCAATAAACACTGTTACAGCAA
>ENST00000439725.5|ENSG00000130600.18|OTTHUMG00000012477.6|OTTHUMT00000142896.3|AC051649.8-003|H19|1929|
CCTGGGCAACGGAGGTGTAGCTGGCAGCAGCGGGCAGGTGAGGACCCCATCTGCCGGGCAGGTGAGTCCCTTCCCTCCCCAGGCCTCGCTTCCCCAG
CCTTCTGAAAGAAGGAGGTTTAGGGGATCGAGGGCTGGCGGGGAGAAGCAGACACCCTCCCAGCAGAGGGGCAGGATGGGGGCAGGAGAGTTAGC
AAAGGTGACATCTTCTCGGGGGGAGCCGAGACTGCGCAAGGCTGGGGGGTTATGGGCCCGTTCCAGGCAGAAAGAGCAAGAGGGCAGGGAGGGAG

CACAGGGGTGGCCAGCGTAGGGTCCAGCACGTGGGGTGGTACCCCAGGCCTGGGTCAGACAGGGACATGGCAGGGGACACAGGACAGAGGGGTCC
CCAGCTGCCACCTCACCCACCGCAATTCATTTAGTAGCAGGCACAGGGGCAGCTCCGGCACGGCTTTCTCAGGCCTATGCCGGAGCCTCGAGGGCTGG
AGAGCGGGAAGACAGGCAGTGCTCGGGGAGTTGCAGCAGGACGTCACCAGGAGGGCGAAGCGGCCACGGGAGGGGGGCCCCGGGACATTGCGCAG
CAAGGAGGCTGCAGGGGCTCGGCCTGCGGGCGCCGGTCCCACGAGGCACTGCGGCCCAGGGTCTGGTGCGGAGAGGGCCCACAGTGGACTTGGTGA
CGCTGTATGCCCTCACCGCTCAGCCCCTGGGGCTGGCTTGGCAGACAGTACAGCATCCAGGGGAGTCAAGGGCATGGGGCGAGACCAGACTAGGCGA
GGCGGGCGGGGCGGAGTGAATGAGCTCTCAGGAGGGAGGATGGTGCAGGCAGGGGTGAGGAGCGCAGCGGGCGGCGAGCGGGAGGCACTGGCCTC
CAGAGCCCGTGGCCAAGGCGGGCCTCGCGGGCGGCGACGGAGCCGGGATCGGTGCCTCAGCGTTCGGGCTGGAGACGAGGGTCTCCAGCTGGGGTG
GACGTGCCCACCAGCTGCCGAAGGCCAAGACGCCAGGTCCGGTGGACGTGACAAGCAGGACATGACATGGTCCGGTGTGACGGCGAGGACAGAGG
AGGCGCGTCCGGCCTTCCTGAACACCTTAGGCTGGTGGGGCTGCGGCAAGAAGCGGGTCTGTTTCTTTACTTCCTCCACGGAGTCGGCACACTATGGC
TGCCCTCTGGGCTCCCAGAACCCACAACATGAAAGAAATGGTGCTACCCAGCTCAAGCCTGGGCCTTTGAATCCGGACACAAAACCCTCTAGCTTGG
AAATGAATATGCTGCACTTTACAACCACTGCACTACCTGACTCAGGAATCGGCTCTGGAAGGTGAAGCTAGAGGAACCAGACCTCATCAGCCCAACAT
CAAAGACACCATCGGAACAGCAGCGCCCGCAGCACCCACCCCGCACCGGCGACTCCATCTTCATGGCCACCCCCTGCGGCGGACGGTTGACCACCAG
CCACCACATCATCCCAGAGCTGAGCTCCTCCAGCGGGATGACGCCGTCCCCACCACCTCCCTCTTCTTCTTTTTCATCCTTCTGTCTCTTTGTTTCTGAG
CTTTCCTGTCTTTCCTTTTTTCTGAGAGATTCAAAGCCTCCACGACTCTGTTTCCCCCGTCCCTTCTGAATTTAATTTGCACTAAGTCATTTGCACTGGTT
GGAGTTGTGGAGACGGCCTTGAGTCTCAGTACGAGTGTGCGTGAGTGTGAGCCACCTTGGCAAGTGCCTGTGCAGGGCCCGGCCGCCCTCCATCTGG
GCCGGGTGACTGGGCGCCGGCTGTGTGCCCGAGGCCTCACCCTGCCCTCGCCTAGTCTGGAAGCTCCGACCGACATCACGGAGCAGCCTTCAAGCAT
TCCATTACGCCCCATCTCGCTCTGTGCCCCTCCCCACCAGGGCTTCAGCAGGAGCCCTGGACTCATCATCAATAAACACTGTTACAGCAA
>ENST00000412788.5|ENSG00000130600.18|OTTHUMG00000012477.6|OTTHUMT00000142895.3|AC051649.8-002|H19|2281|
GGGGTAACGGGGGAAACTGGGGAAGTGGGGAACCGAGGGGCAACCAGGGGAAGATGGGGTGCTGGAGGAGAGCTTGTGGGAGCCAAGGAGCACC
TTGGACATCTGGAGTCTGGCAGGAGTGATGACGGGTGGAGGGGCTAGCTCGAGGCAGGGCTGGTGGGGCCTGAGGCCAGTGAGGAGTGTGGAGTAG
GCGCCCAGGCATCGTGCAGACAGGGCGACATCAGCTGGGGACGATGGGCCTGAGCTAGGGCTGGAAAGAAGGGGGAGCCAGGCATTCATCCCGGTC
ACTTTTGGTTACAGGACGTGGCAGCTGGTTGGACGAGGGGAGCTGGTGGGCAGGGTTTGATCCCAGGGCCTGGGCAACGGAGGTGTAGCTGGCAGC
AGCGGGCAGGTGAGGACCCCATCTGCCGGGCAGGTGAGTCCCTTCCCTCCCCAGGCCTCGCTTCCCCAGCCTTCTGAAAGAAGGAGGTTTAGGGGAT
CGAGGGCTGGCGGGGAGAAGCAGACACCCTCCCAGCAGAGGGGCAGGATGGGGGCAGGAGAGTTAGCAAAGGTGACATCTTCTCGGGGGGAGCCG
AGACTGCGCAAGGCTGGGGGGTTATGGGCCCGTTCCAGGCAGAAAGAGCAAGAGGGCAGGGAGGGAGCACAGGGGTGGCCAGCGTAGGGTCCAGC
ACGTGGGGTGGTACCCCAGGCCTGGGTCAGACAGGGACATGGCAGGGGACACAGGACAGAGGGGTCCCCAGCTGCCACCTCACCCACCGCAATTCA

TTTAGTAGCAGGCACAGGGGCAGCTCCGGCACGGCTTTCTCAGGCCTATGCCGGAGCCTCGAGGGCTGGAGAGCGGGAAGACAGGCAGTGCTCGGG
GAGTTGCAGCAGGACGTCACCAGGAGGGCGAAGCGGCCACGGGAGGGGGGCCCCGGGACATTGCGCAGCAAGGAGGCTGCAGGGGCTCGGCCTGC
GGGCGCCGGTCCCACGAGGCACTGCGGCCCAGGGTCTGGTGCGGAGAGGGCCCACAGTGGACTTGGTGACGCTGTATGCCCTCACCGCTCAGCCCCT
GGGGCTGGCTTGGCAGACAGTACAGCATCCAGGGGAGTCAAGGGCATGGGGCGAGACCAGACTAGGCGAGGCGGGCGGGGCGGAGTGAATGAGCT
CTCAGGAGGGAGGATGGTGCAGGCAGGGGTGAGGAGCGCAGCGGGCGGCGAGCGGGAGGCACTGGCCTCCAGAGCCCGTGGCCAAGGCGGGCCTC
GCGGGCGGCGACGGAGCCGGGATCGGTGCCTCAGCGTTCGGGCTGGAGACGAGGCCAGGTCTCCAGCTGGGGTGGACGTGCCCACCAGCTGCCGAA
GGCCAAGACGCCAGGTCCGGTGGACGTGACAAGCAGGACATGACATGGTCCGGTGTGACGGCGAGGACAGAGGAGGCGCGTCCGGCCTTCCTGAAC
ACCTTAGGCTGGTGGGGCTGCGGCAAGAAGCGGGTCTGTTTCTTTACTTCCTCCACGGAGTCGGCACACTATGGCTGCCCTCTGGGCTCCCAGAACCC
ACAACATGAAAGAAATGGTGCTACCCAGCTCAAGCCTGGGCCTTTGAATCCGGACACAAAACCCTCTAGCTTGGAAATGAATATGCTGCACTTTACAA
CCACTGCACTACCTGACTCAGGAATCGGCTCTGGAAGCTAGAGGAACCAGACCTCATCAGCCCAACATCAAAGACACCATCGGAACAGCAGCGCCCG
CAGCACCCACCCCGCACCGGCGACTCCATCTTCATGGCCACCCCCTGCGGCGGACGGTTGACCACCAGCCACCACATCATCCCAGAGCTGAGCTCCTC
CAGCGGGATGACGCCGTCCCCACCACCTCCCTCTTCTTCTTTTTCATCCTTCTGTCTCTTTGTTTCTGAGCTTTCCTGTCTTTCCTTTTTTCTGAGAGATT
CAAAGCCTCCACGACTCTGTTTCCCCCGTCCCTTCTGAATTTAATTTGCACTAAGTCATTTGCACTGGTTGGAGTTGTGGAGACGGCCTTGAGTCTCAG
TACGAGTGTGCGTGAGTGTGAGCCACCTTGGCAAGTGCCTGTGCAGGGCCCGGCCGCCCTCCATCTGGGCCGGGTGACTGGGCGCCGGCTGTGTGCC
CGAGGCCTCACCCTGCCCTCGCCTAGTCTGGAAGCTCCGACCGACATCACGGAGCAGCCTTCAAGCATTCCATTACGCCCCATCTCGCTCTGTGCCCCT
CCCCACCAGGGCTTCAGCAGGAGCCCTGGACTCATCATCAATAAACACTGTTACAGCAA
>ENST00000414790.6|ENSG00000130600.18|OTTHUMG00000012477.6|OTTHUMT00000034770.3|AC051649.8-001|H19|2348|
AGTTAGAAAAAGCCCGGGCTAGGACCGAGGAGCAGGGTGAGGGAGGGGGTGGGATGGGTGGGGGGTAACGGGGGAAACTGGGGAAGTGGGGAAC
CGAGGGGCAACCAGGGGAAGATGGGGTGCTGGAGGAGAGCTTGTGGGAGCCAAGGAGCACCTTGGACATCTGGAGTCTGGCAGGAGTGATGACGG
GTGGAGGGGCTAGCTCGAGGCAGGGCTGGTGGGGCCTGAGGCCAGTGAGGAGTGTGGAGTAGGCGCCCAGGCATCGTGCAGACAGGGCGACATCAG
CTGGGGACGATGGGCCTGAGCTAGGGCTGGAAAGAAGGGGGAGCCAGGCATTCATCCCGGTCACTTTTGGTTACAGGACGTGGCAGCTGGTTGGACG
AGGGGAGCTGGTGGGCAGGGTTTGATCCCAGGGCCTGGGCAACGGAGGTGTAGCTGGCAGCAGCGGGCAGGTGAGGACCCCATCTGCCGGGCAGGT
GAGTCCCTTCCCTCCCCAGGCCTCGCTTCCCCAGCCTTCTGAAAGAAGGAGGTTTAGGGGATCGAGGGCTGGCGGGGAGAAGCAGACACCCTCCCAG
CAGAGGGGCAGGATGGGGGCAGGAGAGTTAGCAAAGGTGACATCTTCTCGGGGGGAGCCGAGACTGCGCAAGGCTGGGGGGTTATGGGCCCGTTCC
AGGCAGAAAGAGCAAGAGGGCAGGGAGGGAGCACAGGGGTGGCCAGCGTAGGGTCCAGCACGTGGGGTGGTACCCCAGGCCTGGGTCAGACAGGG
ACATGGCAGGGGACACAGGACAGAGGGGTCCCCAGCTGCCACCTCACCCACCGCAATTCATTTAGTAGCAGGCACAGGGGCAGCTCCGGCACGGCTT

TCTCAGGCCTATGCCGGAGCCTCGAGGGCTGGAGAGCGGGAAGACAGGCAGTGCTCGGGGAGTTGCAGCAGGACGTCACCAGGAGGGCGAAGCGG
CCACGGGAGGGGGGCCCCGGGACATTGCGCAGCAAGGAGGCTGCAGGGGCTCGGCCTGCGGGCGCCGGTCCCACGAGGCACTGCGGCCCAGGGTCT
GGTGCGGAGAGGGCCCACAGTGGACTTGGTGACGCTGTATGCCCTCACCGCTCAGCCCCTGGGGCTGGCTTGGCAGACAGTACAGCATCCAGGGGAG
TCAAGGGCATGGGGCGAGACCAGACTAGGCGAGGCGGGCGGGGCGGAGTGAATGAGCTCTCAGGAGGGAGGATGGTGCAGGCAGGGGTGAGGAGC
GCAGCGGGCGGCGAGCGGGAGGCACTGGCCTCCAGAGCCCGTGGCCAAGGCGGGCCTCGCGGGCGGCGACGGAGCCGGGATCGGTGCCTCAGCGT
TCGGGCTGGAGACGAGGCCAGGTCTCCAGCTGGGGTGGACGTGCCCACCAGCTGCCGAAGGCCAAGACGCCAGGTCCGGTGGACGTGACAAGCAG
GACATGACATGGTCCGGTGTGACGGCGAGGACAGAGGAGGCGCGTCCGGCCTTCCTGAACACCTTAGGCTGGTGGGGCTGCGGCAAGAAGCGGGTC
TGTTTCTTTACTTCCTCCACGGAGTCGGCACACTATGGCTGCCCTCTGGGCTCCCAGAACCCACAACATGAAAGAAATGGTGCTACCCAGCTCAAGCC
TGGGCCTTTGAATCCGGACACAAAACCCTCTAGCTTGGAAATGAATATGCTGCACTTTACAACCACTGCACTACCTGACTCAGGAATCGGCTCTGGAA
GGTGAAGCTAGAGGAACCAGACCTCATCAGCCCAACATCAAAGACACCATCGGAACAGCAGCGCCCGCAGCACCCACCCCGCACCGGCGACTCCAT
CTTCATGGCCACCCCCTGCGGCGGACGGTTGACCACCAGCCACCACATCATCCCAGAGCTGAGCTCCTCCAGCGGGATGACGCCGTCCCCACCACCTC
CCTCTTCTTCTTTTTCATCCTTCTGTCTCTTTGTTTCTGAGCTTTCCTGTCTTTCCTTTTTTCTGAGAGATTCAAAGCCTCCACGACTCTGTTTCCCCCGTC
CCTTCTGAATTTAATTTGCACTAAGTCATTTGCACTGGTTGGAGTTGTGGAGACGGCCTTGAGTCTCAGTACGAGTGTGCGTGAGTGTGAGCCACCTTG
GCAAGTGCCTGTGCAGGGCCCGGCCGCCCTCCATCTGGGCCGGGTGACTGGGCGCCGGCTGTGTGCCCGAGGCCTCACCCTGCCCTCGCCTAGTCTG
GAAGCTCCGACCGACATCACGGAGCAGCCTTCAAGCATTCCATTACGCCCCATCTCGCTCTGTGCCCCTCCCCACCAGGGCTTCAGCAGGAGCCCTGG
ACTCATCATCAATAAACACTGTTACAGCAA
>ENST00000428066.6|ENSG00000130600.18|OTTHUMG00000012477.6|OTTHUMT00000142905.4|AC051649.8-012|H19|989|
CATCAGAGGACCATGGCCCCGTATCACCTGGGTCAGGCACTGAAGCTGGGACAGGAGAGCAGAGACTTCCAAAATGAGGGATCCCTGTGTTCTGAGG
TGATCATGACTGGGACCCAAGGACTCAAGCGCATGCTCCAGAGGGAATCGTTTCCCACAAGGCCTTTGGCAGGAACAGGGATCCTGGGAGCCTGCCA
AGCAGAGCGCACAGTGTTCCTGGAGTCTCGCTGCCCAGATGCCACGGAATCAGTTGAAGCCAGGTCTCCAGCTGGGGTGGACGTGCCCACCAGCTGC
CGAAGGCCAAGACGCCAGGTCCGGTGGACGTGACAAGCAGGACATGACATGGTCCGGTGTGACGGCGAGGACAGAGGAGGCGCGTCCGGCCTTCCT
GAACACCTTAGGCTGGTGGGGCTGCGGCAAGAAGCGGGTCTGTTTCTTTACTTCCTCCACGGAGTCGGCACACTATGGCTGCCCTCTGGGCTCCCAGA
ACCCACAACATGAAAGAAATGGTGCTACCCAGCTCAAGCCTGGGCCTTTGAATCCGGACACAAAACCCTCTAGCTTGGAAATGAATATGCTGCACTTT
ACAACCACTGCACTACCTGACTCAGGAATCGGCTCTGGAAGGTGAAGCTAGAGGAACCAGACCTCATCAGCCCAACATCAAAGACACCATCGGAACA
GCAGCGCCCGCAGCACCCACCCCGCACCGGCGACTCCATCTTCATGGCCACCCCCTGCGGCGGACGGTTGACCACCAGCCACCACATCATCCCAGAG
CTGAGCTCCTCCAGCGGGATGACGCCGTCCCCACCACCTCCCTCTTCTTCTTTTTCATCCTTCTGTCTCTTTGTTTCTGAGCTTTCCTGTCTTTCCTTTTT

TCTGAGAGATTCAAAGCCTCCACGACTCTGTTTCCCCCGTCCCTTCTGAATTTAATTTGCACTAAGTCATTTGCACTGGTTGGAGTTGTGGAGACGGCC
TTGAGTCTCAGT
>ENST00000436715.5|ENSG00000130600.18|OTTHUMG00000012477.6|OTTHUMT00000142906.3|AC051649.8-013|H19|861|
GCGGGCGGCGACGGAGCCGGGATCGGTGCCTCAGCGTTCGGGCTGGAGACGAGGCCAGGTCTCCAGCTGGGGTGGACGTGCCCACCAGCTGCCGAA
GGCCAAGACGCCAGGTCCGGTGGACGTGACAAGCAGGACATGACATGGTCCGGTGTGACGGCGAGGACAGAGGAGGCGCGTCCGGCCTTCCTGAAC
ACCTTAGGCTGGTGGGGCTGCGGCAAGAAGCGGGTCTGTTTCTTTACTTCCTCCACGGAGTCGGCACACTATGGCTGCCCTCTGGGCTCCCAGAACCC
ACAACATGAAAGGTGAGGGGCTTCCTGCCACACTTGGGGTGGGGGCACGCGAGAGGAGCTGAGTGGGACCTCACTCCTTCCCCATCCACAGAAAT
GGTGCTACCCAGCTCAAGCCTGGGCCTTTGAATCCGGACACAAAACCCTCTAGCTTGGAAATGAATATGCTGCACTTTACAACCACTGCACTACCTGA
CTCAGGAATCGGCTCTGGAAGGTGAAGCTAGAGGAACCAGACCTCATCAGCCCAACATCAAAGACACCATCGGAACAGCAGCGCCCGCAGCACCCA
CCCCGCACCGGCGACTCCATCTTCATGGCCACCCCCTGCGGCGGACGGTTGACCACCAGCCACCACATCATCCCAGAGCTGAGCTCCTCCAGCGGGAT
GACGCCGTCCCCACCACCTCCCTCTTCTTCTTTTTCATCCTTCTGTCTCTTTGTTTCTGAGCTTTCCTGTCTTTCCTTTTTTCTGAGAGATTCAAAGCCTC
CACGACTCTGTTTCCCCCGTCCCTTCTGAATTTAATTTGCACTAAGTCATTTGCACTGGTTGGAGTTGTGGAGACGGCCTTG
>ENST00000447298.2|ENSG00000130600.18|OTTHUMG00000012477.6|OTTHUMT00000142907.4|AC051649.8-014|H19|592|
ACAACCAACACGTTCTCCCCACACGACTCTCTCGTTCTCCCCACAGCCAGGTCTCCAGCTGGGGTGGACGTGCCCACCAGCTGCCGAAGGCCAAGAC
GCCAGGTCCGGTGGACGTGACAAGCAGGACATGACATGGTCCGGTGTGACGGCGAGGACAGAGGAGGCGCGTCCGGCCTTCCTGAACACCTTAGGC
TGGTGGGGCTGCGGCAAGAAGCGGGTCTGTTTCTTTACTTCCTCCACGGAGTCGGCACACTATGGCTGCCCTCTGGGCTCCCAGAACCCACAACATGA
AAGAAATGGTGCTACCCAGCTCAAGCCTGGGCCTTTGAATCCGGACACAAAACCCTCTAGCTTGGAAATGAATATGCTGCACTTTACAACCACTGCAC
TACCTGACTCAGGAATCGGCTCTGGAAGGTGAAGCTAGAGGAACCAGACCTCATCAGCCCAACATCAAAGACACCATCGGAACAGCAGCGCCCGCAG
CACCCACCCCGCACCGGCGACTCCATCTTCATGGCCACCCCCTGCGGCGGACGGTTGACCACCAGCCACCACATCATCCCAGAGCTGAGCTCCTCCAG
CGGGATGA
>ENST00000431095.6|ENSG00000130600.18|OTTHUMG00000012477.6|OTTHUMT00000495010.1|AC051649.8-017|H19|489|
GTGTCTGCCCTCCCTGCGTCAGGACGCGGCCCTGCCCAGACCGCCCCGCCGGGCCACCATCTCACTGCCCCGACCTCTGTCTTCTACAGAACACCTTA
GGCTGGTGGGGCTGCGGCAAGAAGCGGGTCTGTTTCTTTACTTCCTCCACGGAGTCGGCACACTATGGCTGCCCTCTGGGCTCCCAGAACCCACAACA
TGAAAGAAATGGTGCTACCCAGCTCAAGCCTGGGCCTTTGAATCCGGACACAAAACCCTCTAGCTTGGAAATGAATATGCTGCACTTTACAACCACTG
CACTACCTGACTCAGGAATCGGCTCTGGAAGGTGAAGCTAGAGGAACCAGACCTCATCAGCCCAACATCAAAGACACCATCGGAACAGCAGCGCCCG
CAGCACCCACCCCGCACCGGCGACTCCATCTTCATGGCCACCCCCTGCGGCGGACGGTTGACCACCAGCCACCACATCATCCCAGAGCTGAGCTCCTC

>ENST00000422826.2|ENSG00000130600.18|OTTHUMG00000012477.6|OTTHUMT00000142900.4|AC051649.8-007|H19|554|
AGTGTTCCTGGAGTCTCGCTGCCCAGATGCCACGGAATCAGTTGAAGGACGTGGCAGCTGGTTGGACGAGGGGAGCTGGTGGGCAGGGTTTGATCCC
AGGGCCTGGGCAACGGAGGTGTAGCTGGCAGCAGCGGGCAGGTGAGGACCCCATCTGCCGGGCAGGTGAGTCCCTTCCCTCCCCAGGCCTCGCTTCC
CCAGCCTTCTGAAAGAAGGAGGTTTAGGGGATCGAGGGCTGGCGGGGAGAAGCAGACACCCTCCCAGCAGAGGGGCAGGATGGGGGCAGGAGAGT
TAGCAAAGGTGACATCTTCTCGGGGGGAGCCGAGACTGCGCAAGGCTGGGGGGTTATGGGCCCGTTCCAGGCAGAAAGAGCAAGAGGGCAGGGAGG
GAGCACAGGGGTGGCCAGCGTAGGGTCCAGCACGTGGGGTGGTACCCCAGGCCTGGGTCAGACAGGGACATGGCAGGGGACACAGGACAGAGGGG
TCCCCAGCTGCCACCTCACCCACCGCAATTCATTTAGTAGCAGGCACAGGGGCAGCTCCGGCACGGCTTTCTCA
>ENST00000643292.1|ENSG00000130600.18|OTTHUMG00000012477.6|OTTHUMT00000142901.4|AC051649.8-008|H19|475|
AGTTAGAAAAAGCCCGGGCTAGGACCGAGGAGCAGGGACGTGGCAGCTGGTTGGACGAGGGGAGCTGGTGGGCAGGGTTTGATCCCAGGGCCTGG
GCAACGGAGGTGTAGCTGGCAGCAGCGGGCAGGTGAGGACCCCATCTGCCGGGCAGGTGAGTCCCTTCCCTCCCCAGGCCTCGCTTCCCCAGCCTTC
TGAAAGAAGGAGGTTTAGGGGATCGAGGGCTGGCGGGGAGAAGCAGACACCCTCCCAGCAGAGGGGCAGGATGGGGGCAGGAGAGTTAGCAAAGG
TGACATCTTCTCGGGGGGAGCCGAGACTGCGCAAGGCTGGGGGGTTATGGGCCCGTTCCAGGCAGAAAGAGCAAGAGGGCAGGGAGGGAGCACAG
GGGTGGCCAGCGTAGGGTCCAGCACGTGGGGTGGTACCCCAGGCCTGGGTCAGACAGGGACATGGCAGGGGACACAGGACAGAGGGGTCCCCA
>GAS5
>ENST00000449289.5|ENSG00000234741.7|OTTHUMG00000037216.5|OTTHUMT00000090584.1|RP5-1198E17.2-008|GAS5|542|
CTTTTCGAGGTAGGAGTCGACTCCTGTGAGGTATGGTGCTGGGTGCAGATGCAGTGTGGCTCTGGATAGCACCTTATGGACAGTTGTGTCCCCAAGGA
AGGATGAGAATAGCTACTGAAGTCCTAAAGAGCAAGCCTAACTCAAGCCATTGGCACACAGGCATTAGACAGAAAGCTGGAAGTTGAAATGGTGGAG
TCCAACTTGCCTGGACCAGCTTAATGGTTCTGCTCCTGGTAACGTTTTTATCCATGGATGACTTGCTTGGGACTCAGAATTCATGATTGAAGAAATGCAG
GCAGACCTGTTATCCTAAACTAGGGTTTTTAATGACCACAACAAGCAAGCATGCAGCTTACTGCTTGAAAGGGTCTTGCCTCACCCAAGCTAGAGTGC
AGTGGCCTTTGAAGCTTACTACAGCCTCAAACTTCTGGGCTCAAGTGATCCTCAGCCTCCCAGTGGTCTTTGTAGACTGCCTGATGGAGTCTCATGGCA
CAAGAAGATTAAAACAGTGTCTCCAATTTTAATAAATTTTTGCAATCCAT
>ENST00000414075.5|ENSG00000234741.7|OTTHUMG00000037216.5|OTTHUMT00000090604.1|RP5-1198E17.2-028|GAS5|413|
GCATTAGACAGAAAGCTGGAAGTTGAAATGGTGGAGTCCAACTTGCCTGGACCAGCTTAATGGTTCTGCTCCTGGTAACGTTTTTATCCATGGATGACT
TGCTTGGGTATGGAGAGTCGGCTTGACTACACTGTGTGGAGCAAGTTTTAAAGAAGCAAAGGACTCAGAATTCATGATTGAAGAAATGCAGGGTTTTT
AATGACCACAACAAGCAAGCATGCAGCTTACTGCTTGAAAGGGTCTTGCCTCACCCAAGCTAGAGTGCAGTGGCCTTTGAAGCTTACTACAGCCTCAA
ACTTCTGGGCTCAAGTGATCCTCAGCCTCCCAGTGGTCTTTGTAGACTGCCTGATGGAGTCTCATGGCACAAGAAGATTAAAACAGTGTCTCCAATTTT

AATAAATTTTTGCAATCCA
>ENST00000448718.5|ENSG00000234741.7|OTTHUMG00000037216.5|OTTHUMT00000090579.1|RP5-1198E17.2-003|GAS5|565|
GTATGGTGCTGGGTGCAGATGCAGTGTGGCTCTGGATAGCACCTTATGGACAGTTGTGTCCCCAAGGAAGGATGAGAATAGCTACTGAAGTCCTAAAG
AGCAAGCCTAACTCAAGCCATTGGCACACAGGCATTAGACAGAAAGCTGGAAGTTGAAATGGTGGAGTCCAACTTGCCTGGACCAGCTTAATGGTTC
TGCTCCTGGTAACGTTTTTATCCATGGATGACTTGCTTGGGTATGGAGAGTCGGCTTGACTACACTGTGTGGAGCAAGTTTTAAAGAAGCAAAGGACTC
AGAATTCATGATTGAAGAAATGCAGGCAGACCTGTTATCCTAAACTAGGGTTTTTAATGACCACAACAAGCAAGCATGCAGCTTACTGCTTGAAAGGG
TCTTGCCTCACCCAAGCTAGAGTGCAGTGGCCTTTGAAGCTTACTACAGCCTCAAACTTCTGGGCTCAAGTGATCCTCAGCCTCCCAGTGGTCTTTGTA
GACTGCCTGATGGAGTCTCATGGCACAAGAAGATTAAAACAGTGTCTCCAATTTTAATAAATTTTTGCAATCCA
>ENST00000436285.5|ENSG00000234741.7|OTTHUMG00000037216.5|OTTHUMT00000090593.1|RP5-1198E17.2-017|GAS5|772|
GTATGGTGCTGGGTGCAGATGCAGTGTGGCTCTGGATAGCACCTTATGGACAGTTGTGTCCCCAAGGAAGGATGAGAATAGCTACTGAAGTCCTAAAG
AGCAAGCCTAACTCAAGCCATTGGCACACAGGCATTAGACAGAAAGCTGGAAGTTGAAATGGTGGAGTCCAACTTGCCTGGACCAGCTTAATGGTTC
TGCTCCTGGTAACGTTTTTATCCATGGATGACTTGCTTGGGTAAGGACATGAAGACAGTTCCTGTCATACCTTTTAAAGGTATGGAGAGTCGGCTTGACT
ACACTGTGTGGAGCAAGTTTTAAAGAAGCAAAGGACTCAGAATTCATGATTGAAGAAATGCAGGTTAGTTTAAACTTTGAAGGAAATTTTTAAGGTGG
CAAAAGGTTTTGGTGGCATATACACCTTAATCTGTAGATGGGAGTGATTAGCTGTTTAAAAGTTAAAATGTGACTGAGAAGGAAATTGAGTAGGGCAAA
TTTTAAATGGGTATTATTTTTCATCTTCAAACAGGCAGACCTGTTATCCTAAACTAGGGTTTTTAATGACCACAACAAGCAAGCATGCAGCTTACTGCTT
GAAAGGGTCTTGCCTCACCCAAGCTAGAGTGCAGTGGCCTTTGAAGCTTACTACAGCCTCAAACTTCTGGGCTCAAGTGATCCTCAGCCTCCCAGTGG
TCTTTGTAGACTGCCTGATGGAGTCTCATGGCACAAGAAGATTAAAACAGTGTCTCCAATTTTAATAAATTTTTGCAATCCA
>ENST00000456812.6|ENSG00000234741.7|OTTHUMG00000037216.5|OTTHUMT00000090605.2|RP5-1198E17.2-029|GAS5|723|
GTATGGTGCTGGGTGCAGATGCAGTGTGGCTCTGGATAGCACCTTATGGACAGTTGTGTCCCCAAGGAAGGATGAGAATAGCTACTGAAGTCCTAAAG
AGCAAGCCTAACTCAAGCCATTGGCACACAGGCATTAGACAGAAAGCTGGAAGTTGAAATGGTGGAGTCCAACTTGCCTGGACCAGCTTAATGGTTC
TGCTCCTGGTAACGTTTTTATCCATGGATGACTTGCTTGGGTATGGAGAGTCGGCTTGACTACACTGTGTGGAGCAAGTTTTAAAGAAGCAAAGGACTC
AGAATTCATGATTGAAGAAATGCAGGGTTTTTAATGACCACAACAAGCAAGCATGCAGCTTACTGCTTGAAAGGTGAGGATTGGAAATGTTGGGACTA
TTATAATTGCAGAATACATGATGATCTCAATCCAACTTGAACTCTCTCACTGATTACTTGATGACAATAAAATATCTGATATTCTGCATTCCCATGTAGCAT
TTTAATTGAAGTCTGTAAATGTGGCTAAAAGTCTTGTCTTATTTTTTGAGACAGGGTCTTGCCTCACCCAAGCTAGAGTGCAGTGGCCTTTGAAGCTTA
CTACAGCCTCAAACTTCTGGGCTCAAGTGATCCTCAGCCTCCCAGTGGTCTTTGTAGACTGCCTGATGGAGTCTCATGGCACAAGAAGATTAAAACAG
TGTCTCCAATTTTAATAAATTTTTGCAATCCA

>ENST00000452197.5|ENSG00000234741.7|OTTHUMG00000037216.5|OTTHUMT00000090589.1|RP5-1198E17.2-013|GAS5|483|
GAGGTAGGAGTCGACTCCTGTGAGGTATGGTGCTGGGTGCAGATGCAGTGTGGCTCTGGATAGCACCTTATGGACAGTTGTGTCCCCAAGGAAGGATG
AGAATAGCTACTGAAGTCCTAAAGAGCAAGCCTAACTCAAGCCATTGGCACACAGGTATGGAGAGTCGGCTTGACTACACTGTGTGGAGCAAGTTTTA
AAGAAGCAAAGGACTCAGAATTCATGATTGAAGAAATGCAGGCAGACCTGTTATCCTAAACTAGGGTTTTTAATGACCACAACAAGCAAGCATGCAGC
TTACTGCTTGAAAGGGTCTTGCCTCACCCAAGCTAGAGTGCAGTGGCCTTTGAAGCTTACTACAGCCTCAAACTTCTGGGCTCAAGTGATCCTCAGCC
TCCCAGTGGTCTTTGTAGACTGCCTGATGGAGTCTCATGGCACAAGAAGATTAAAACAGTGTCTCCAATTTTAATAAATTTTTGCAATCCA
>ENST00000450589.5|ENSG00000234741.7|OTTHUMG00000037216.5|OTTHUMT00000090577.1|RP5-1198E17.2-001|GAS5|632|
TTTCGAGGTAGGAGTCGACTCCTGTGAGGTATGGTGCTGGGTGCAGATGCAGTGTGGCTCTGGATAGCACCTTATGGACAGTTGTGTCCCCAAGGAAG
GATGAGAATAGCTACTGAAGTCCTAAAGAGCAAGCCTAACTCAAGCCATTGGCACACAGGCATTAGACAGAAAGCTGGAAGTTGAAATGGTGGAGTC
CAACTTGCCTGGACCAGCTTAATGGTTCTGCTCCTGGTAACGTTTTTATCCATGGATGACTTGCTTGGGTAAGGACATGAAGACAGTTCCTGTCATACCT
TTTAAAGGTATGGAGAGTCGGCTTGACTACACTGTGTGGAGCAAGTTTTAAAGAAGCAAAGGACTCAGAATTCATGATTGAAGAAATGCAGGCAGAC
CTGTTATCCTAAACTAGGGTTTTTAATGACCACAACAAGCAAGCATGCAGCTTACTGCTTGAAAGGGTCTTGCCTCACCCAAGCTAGAGTGCAGTGGC
CTTTGAAGCTTACTACAGCCTCAAACTTCTGGGCTCAAGTGATCCTCAGCCTCCCAGTGGTCTTTGTAGACTGCCTGATGGAGTCTCATGGCACAAGAA
GATTAAAACAGTGTCTCCAATTTTAATAAATTTTTGCAATCCA
>ENST00000412059.5|ENSG00000234741.7|OTTHUMG00000037216.5|OTTHUMT00000090598.1|RP5-1198E17.2-022|GAS5|979|
TTTCGAGGTAGGAGTCGACTCCTGTGAGGTATGGTGCTGGGTGCAGATGCAGTGTGGCTCTGGATAGCACCTTATGGACAGTTGTGTCCCCAAGGAAG
GATGAGAATAGCTACTGAAGTCCTAAAGAGCAAGCCTAACTCAAGCCATTGGCACACAGGCATTAGACAGAAAGCTGGAAGTTGAAATGGTGGAGTC
CAACTTGCCTGGACCAGCTTAATGGTTCTGCTCCTGGTAACGTTTTTATCCATGGATGACTTGCTTGGGTAAGGACATGAAGACAGTTCCTGTCATACCT
TTTAAAGGTATGGAGAGTCGGCTTGACTACACTGTGTGGAGCAAGTTTTAAAGAAGCAAAGGACTCAGAATTCATGATTGAAGAAATGCAGGTTAGTT
TAAACTTTGAAGGAAATTTTTAAGGTGGCAAAAGGTTTTGGTGGCATATACACCTTAATCTGTAGATGGGAGTGATTAGCTGTTTAAAAGTTAAAATGT
GACTGAGAAGGAAATTGAGTAGGGCAAATTTTAAATGGGTATTATTTTTCATCTTCAAACAGGCAGACCTGTTATCCTAAACTAGGTGAGTCAGCTTTT
GGTACATGTGATGATTTTCAGTGTAACCAATGATGTAATGATTCTGCCAAATGAAATATAATGATATCACTGTAAAACCGTTCCATTTTGATTCTGAGGTT
ACTCTACTAACAAGCATCACACATTTGTATTTTGCCCTGATTAATATGTTGGCTTCGCTTTCAGGGTTTTTAATGACCACAACAAGCAAGCATGCAGCTT
ACTGCTTGAAAGGGTCTTGCCTCACCCAAGCTAGAGTGCAGTGGCCTTTGAAGCTTACTACAGCCTCAAACTTCTGGGCTCAAGTGATCCTCAGCCTC
CCAGTGGTCTTTGTAGACTGCCTGATGGAGTCTCATGGCACAAGAAGATTAAAACAGTGTCTCCAATTTTAATAAATTTTTGCAATCCA
>ENST00000431268.5|ENSG00000234741.7|OTTHUMG00000037216.5|OTTHUMT00000090578.1|RP5-1198E17.2-002|GAS5|1698|

CTTTTCGAGGTAGGAGTCGACTCCTGTGAGGTATGGTGCTGGGTGCAGATGCAGTGTGGCTCTGGATAGCACCTTATGGACAGTTGTGTCCCCAAGGA
AGGATGAGAATAGCTACTGAAGTAAGTTGAAAATTCCCTCTCAAAAAGGTTTAAAGCCATTGGATGTGCCACAATGATGACAGTTTATTTGCTACTCTT
GAGTGCTAGAATGATGAGGATCTTAACCACCATTATCTTAACTGAGGCACCCAAAATGGTGAGTTGGGGAACATAGAGAGTACACCTAAGTTCACATG
AAGTTGTTTCTTCCCAGGTCCTAAAGAGCAAGCCTAACTCAAGCCATTGGCACACAGGCATTAGACAGAAAGCTGGAAGTTGAAATGGTGGAGTCCA
ACTTGCCTGGACCAGCTTAATGGTTCTGCTCCTGGTAACGTTTTTATCCATGGATGACTTGCTTGGGTAAGGACATGAAGACAGTTCCTGTCATACCTTT
TAAAGGTATGGAGAGTCGGCTTGACTACACTGTGTGGAGCAAGTTTTAAAGAAGCAAAGGTATAGCAGTTCCAAGTATTTTTTTTTTTTTTTTAGACAA
GAGTCTAGCTCTTGCCCAGAATGGAGTGCAGCGGCACTATCAGTTCACTGCAACCTCTGCCTCCCAGGTTCAAGGAATTCTCCTGCCTCAGCCTCTTGA
GTAGCTGGGATTACAGGCATGTGACACCATGCCTGGCTAATTTTGTACAGCTATGTTGTCCAGGCTGGTCTCGAACTCTTGACCTCAAGTGATACTGCCC
GCACTGACCTCCCAAAGTGCTGGGATTACAGGCGTGAGCCACCATGCCCCGCCTCAAGTCTGGTTTTTAAGTGTTGTAAAGCCGATACAATGATGATAA
CATAGTTCAGCAGACTAACGCTGATGAGCAATATTAAGTCTTTCGCTCCTATCTGATGTATCTGGCGGTAACATTCTAGTTTATGCCCCGAAAAGGGGAA
TATAGCCATTCTATAATGTTTGGAGATTTTGGATTACTCCTAATTGTATGCAAGTTGTCTTACTGTGTATTGTCCCTTAATTTCAGGACTCAGAATTCATGA
TTGAAGAAATGCAGGTTAGTTTAAACTTTGAAGGAAATTTTTAAGGTGGCAAAAGGTTTTGGTGGCATATACACCTTAATCTGTAGATGGGAGTGATTA
GCTGTTTAAAAGTTAAAATGTGACTGAGAAGGAAATTGAGTAGGGCAAATTTTAAATGGGTATTATTTTTCATCTTCAAACAGGCAGACCTGTTATCCTA
AACTAGGTGAGTCAGCTTTTGGTACATGTGATGATTTTCAGTGTAACCAATGATGTAATGATTCTGCCAAATGAAATATAATGATATCACTGTAAAACCGT
TCCATTTTGATTCTGAGGTTACTCTACTAACAAGCATCACACATTTGTATTTTGCCCTGATTAATATGTTGGCTTCGCTTTCAGGGTTTTTAATGACCACA
ACAAGCAAGCATGCAGCTTACTGCTTGAAAGGGTCTTGCCTCACCCAAGCTAGAGTGCAGTGGCCTTTGAAGCTTACTACAGCCTCAAACTTCTGGGC
TCAAGTGATCCTCAGCCTCCCAGTGGTCTTTGTAGACTGCCTGATGGAGTCTCATGGCACAAGAAGATTAAAACAGTGTCTCCAATTTTAATAAATTTTT
GCAATCCA
>ENST00000436656.5|ENSG00000234741.7|OTTHUMG00000037216.5|OTTHUMT00000090580.1|RP5-1198E17.2-004|GAS5|822|
CTTTTCGAGGTAGGAGTCGACTCCTGTGAGGTATGGTGCTGGGTGCAGATGCAGTGTGGCTCTGGATAGCACCTTATGGACAGTTGTGTCCCCAAGGA
AGGATGAGAATAGCTACTGAAGTCCTAAAGAGCAAGCCTAACTCAAGCCATTGGCACACAGGCATTAGACAGAAAGCTGGAAGTTGAAATGGTGGAG
TCCAACTTGCCTGGACCAGCTTAATGGTTCTGCTCCTGGTAACGTTTTTATCCATGGATGACTTGCTTGGGTAAGGACATGAAGACAGTTCCTGTCATAC
CTTTTAAAGGTACATGTTTTATTGATGTTAACGTTAATTGATTGAGCTACTGTTAGTGATGATTTTAAAATTAAAGCAGATGGGAATCTCTCTGAGAAAGA
AAATGGAGATTAATCTTAAACTGAAACAGTAGTTGGGAAATCTTTTAGAAATCCACCTATTACTACCTATTGGTAAAGGAGATTAAATTTCTACAGGTAT
GGAGAGTCGGCTTGACTACACTGTGTGGAGCAAGTTTTAAAGAAGCAAAGGACTCAGAATTCATGATTGAAGAAATGCAGGCAGACCTGTTATCCTAA
ACTAGGGTTTTTAATGACCACAACAAGCAAGCATGCAGCTTACTGCTTGAAAGGGTCTTGCCTCACCCAAGCTAGAGTGCAGTGGCCTTTGAAGCTTA

CTACAGCCTCAAACTTCTGGGCTCAAGTGATCCTCAGCCTCCCAGTGGTCTTTGTAGACTGCCTGATGGAGTCTCATGGCACAAGAAGATTAAAACAG
TGTCTCCAATTTTAATAAATTTTTGCAATCCA
>ENST00000430245.5|ENSG00000234741.7|OTTHUMG00000037216.5|OTTHUMT00000090603.2|RP5-1198E17.2-027|GAS5|723|
CAGCACTTGAGCAGCTTTCTTCTGCGTTAGGAAGCCTGGGGAGAGGGGAACTGGCTAAAGGGCAAAATGTCACTGAGGAACTTCGGAGAAGATGGG
GACAAGGCGTCGGGCGGGGGAGCCAGCGCTCAGGAGCCCAGGCACCATACTGTCTTTTCGAGGTATGGTGCTGGGTGCAGATGCAGTGTGGCTCTGG
ATAGCACCTTATGGACAGTTGTGTCCCCAAGGAAGGATGAGAATAGCTACTGAAGTCCTAAAGAGCAAGCCTAACTCAAGCCATTGGCACACAGGCAT
TAGACAGAAAGCTGGAAGTTGAAATGGTGGAGTCCAACTTGCCTGGACCAGCTTAATGGTTCTGCTCCTGGTAACGTTTTTATCCATGGATGACTTGCT
TGGGTATGGAGAGTCGGCTTGACTACACTGTGTGGAGCAAGTTTTAAAGAAGCAAAGGACTCAGAATTCATGATTGAAGAAATGCAGGCAGACCTGTT
ATCCTAAACTAGGGTTTTTAATGACCACAACAAGCAAGCATGCAGCTTACTGCTTGAAAGGGTCTTGCCTCACCCAAGCTAGAGTGCAGTGGCCTTTG
AAGCTTACTACAGCCTCAAACTTCTGGGCTCAAGTGATCCTCAGCCTCCCAGTGGTCTTTGTAGACTGCCTGATGGAGTCTCATGGCACAAGAAGATTA
AAACAGTGTCTCCAATTTTAATAAATTTTTGCAATCCA
>ENST00000454813.1|ENSG00000234741.7|OTTHUMG00000037216.5|OTTHUMT00000090602.1|RP5-1198E17.2-026|GAS5|621|
GCTGATGAGCAATATTAAGTCTTTCGCTCCTATCTGATGTATCTGGCGGTAACATTCTAGTTTATGCCCCGAAAAGGGGAATATAGCCATTCTATAATGTTT
GGAGATTTTGGATTACTCCTAATTGTATGCAAGTTGTCTTACTGTGTATTGTCCCTTAATTTCAGGACTCAGAATTCATGATTGAAGAAATGCAGGCAGA
CCTGTTATCCTAAACTAGGTGAGTCAGCTTTTGGTACATGTGATGATTTTCAGTGTAACCAATGATGTAATGATTCTGCCAAATGAAATATAATGATATCA
CTGTAAAACCGTTCCATTTTGATTCTGAGGTTACTCTACTAACAAGCATCACACATTTGTATTTTGCCCTGATTAATATGTTGGCTTCGCTTTCAGGGTTT
TTAATGACCACAACAAGCAAGCATGCAGCTTACTGCTTGAAAGGGTCTTGCCTCACCCAAGCTAGAGTGCAGTGGCCTTTGAAGCTTACTACAGCCTC
AAACTTCTGGGCTCAAGTGATCCTCAGCCTCCCAGTGGTCTTTGTAGACTGCCTGATGGAGTCTCATGGCACAAGAAGATTAAAACAGTGTCTCCAATT
TTAATAAATTTTTGCAATCC
>ENST00000422183.5|ENSG00000234741.7|OTTHUMG00000037216.5|OTTHUMT00000090595.1|RP5-1198E17.2-019|GAS5|745|
GTATGGTGCTGGGTGCAGATGCAGTGTGGCTCTGGATAGCACCTTATGGACAGTTGTGTCCCCAAGGAAGGATGAGAATAGCTACTGAAGTCCTAAAG
AGCAAGCCTAACTCAAGCCATTGGCACACAGGCATTAGACAGAAAGCTGGAAGTTGAAATGGTGGAGTCCAACTTGCCTGGACCAGCTTAATGGTTC
TGCTCCTGGTAACGTTTTTATCCATGGATGACTTGCTTGGGTATGGAGAGTCGGCTTGACTACACTGTGTGGAGCAAGTTTTAAAGAAGCAAAGGACTC
AGAATTCATGATTGAAGAAATGCAGGCAGACCTGTTATCCTAAACTAGGGTTTTTAATGACCACAACAAGCAAGCATGCAGCTTACTGCTTGAAAGGT
GAGGATTGGAAATGTTGGGACTATTATAATTGCAGAATACATGATGATCTCAATCCAACTTGAACTCTCTCACTGATTACTTGATGACAATAAAATATCTG
ATATTCTGCATTCCCATGTAGCATTTTAATTGAAGTCTGTAAATGTGGCTAAAAGTCTTGTCTTATTTTTTGAGACAGGGTCTTGCCTCACCCAAGCTAGA

GTGCAGTGGCCTTTGAAGCTTACTACAGCCTCAAACTTCTGGGCTCAAGTGATCCTCAGCCTCCCAGTGGTCTTTGTAGACTGCCTGATGGAGTCTCAT
GGCACAAGAAGATTAAAACAGTGTCTCCAATTTTAATAAATTTTTGCAATCC
>ENST00000422207.5|ENSG00000234741.7|OTTHUMG00000037216.5|OTTHUMT00000090599.1|RP5-1198E17.2-023|GAS5|643|
GATACAATGATGATAACATAGTTCAGCAGACTAACGCTGATGAGCAATATTAAGTCTTTCGCTCCTATCTGATGTATCTGGCGGTAACATTCTAGTTTATG
CCCCGAAAAGGGGAATATAGCCATTCTATAATGTTTGGAGATTTTGGATTACTCCTAATTGTATGCAAGTTGTCTTACTGTGTATTGTCCCTTAATTTCAG
GACTCAGAATTCATGATTGAAGAAATGCAGGTTAGTTTAAACTTTGAAGGAAATTTTTAAGGTGGCAAAAGGTTTTGGTGGCATATACACCTTAATCTG
TAGATGGGAGTGATTAGCTGTTTAAAAGTTAAAATGTGACTGAGAAGGAAATTGAGTAGGGCAAATTTTAAATGGGTATTATTTTTCATCTTCAAACAG
GCAGACCTGTTATCCTAAACTAGGGTTTTTAATGACCACAACAAGCAAGCATGCAGCTTACTGCTTGAAAGGGTCTTGCCTCACCCAAGCTAGAGTGC
AGTGGCCTTTGAAGCTTACTACAGCCTCAAACTTCTGGGCTCAAGTGATCCTCAGCCTCCCAGTGGTCTTTGTAGACTGCCTGATGGAGTCTCATGGCA
CAAGAAGATTAAAACAGTGTCTCCAATTTTAATAAATTTTTGCAAT
>ENST00000454068.5|ENSG00000234741.7|OTTHUMG00000037216.5|OTTHUMT00000090590.1|RP5-1198E17.2-014|GAS5|688|
ATGGTGCATGCGTGACCTAAGCTGCGACTATGTTAGAGTAGAACTGCGGAGAAGCCTCGGCTCTCGTGCCCTGCCTCTGATGAAGCCTGTGTTGGTATG
GTGCTGGGTGCAGATGCAGTGTGGCTCTGGATAGCACCTTATGGACAGTTGTGTCCCCAAGGAAGGATGAGAATAGCTACTGAAGTCCTAAAGAGCAA
GCCTAACTCAAGCCATTGGCACACAGGCATTAGACAGAAAGCTGGAAGTTGAAATGGTGGAGTCCAACTTGCCTGGACCAGCTTAATGGTTCTGCTCC
TGGTAACGTTTTTATCCATGGATGACTTGCTTGGGTAAGGACATGAAGACAGTTCCTGTCATACCTTTTAAAGGTATGGAGAGTCGGCTTGACTACACT
GTGTGGAGCAAGTTTTAAAGAAGCAAAGGACTCAGAATTCATGATTGAAGAAATGCAGGCAGACCTGTTATCCTAAACTAGGGTTTTTAATGACCACA
ACAAGCAAGCATGCAGCTTACTGCTTGAAAGGGTCTTGCCTCACCCAAGCTAGAGTGCAGTGGCCTTTGAAGCTTACTACAGCCTCAAACTTCTGGGC
TCAAGTGATCCTCAGCCTCCCAGTGGTCTTTGTAGACTGCCTGATGGAGTCTCATGGCACAAGAAGATTAAAACAGTGTCTCCAATTTTAATAAATTT
>ENST00000444470.5|ENSG00000234741.7|OTTHUMG00000037216.5|OTTHUMT00000090601.1|RP5-1198E17.2-025|GAS5|424|
ATACAATGATGATAACATAGTTCAGCAGACTAACGCTGATGAGCAATATTAAGTCTTTCGCTCCTATCTGATGTATCTGGCGGTAACATTCTAGTTTATGC
CCCGAAAAGGGGAATATAGCCATTCTATAATGTTTGGAGATTTTGGATTACTCCTAATTGTATGCAAGTTGTCTTACTGTGTATTGTCCCTTAATTTCAGG
ACTCAGAATTCATGATTGAAGAAATGCAGGCAGACCTGTTATCCTAAACTAGGGTTTTTAATGACCACAACAAGCAAGCATGCAGCTTACTGCTTGAAA
GGGTCTTGCCTCACCCAAGCTAGAGTGCAGTGGCCTTTGAAGCTTACTACAGCCTCAAACTTCTGGGCTCAAGTGATCCTCAGCCTCCCAGTGGTCTT
TGTAGACTGCCTGATGGAGTCTCAT
>ENST00000434796.5|ENSG00000234741.7|OTTHUMG00000037216.5|OTTHUMT00000090600.1|RP5-1198E17.2-024|GAS5|575|
AATGATGATAACATAGTTCAGCAGACTAACGCTGATGAGCAATATTAAGTCTTTCGCTCCTATCTGATGTATCTGGCGGTAACATTCTAGTTTATGCCCCG

AAAAGGGGAATATAGCCATTCTATAATGTTTGGAGATTTTGGATTACTCCTAATTGTATGCAAGTTGTCTTACTGTGTATTGTCCCTTAATTTCAGGACTC
AGAATTCATGATTGAAGAAATGCAGGCAGACCTGTTATCCTAAACTAGGGTTTTTAATGACCACAACAAGCAAGCATGCAGCTTACTGCTTGAAAGGT
GAGGATTGGAAATGTTGGGACTATTATAATTGCAGAATACATGATGATCTCAATCCAACTTGAACTCTCTCACTGATTACTTGATGACAATAAAATATCTG
ATATTCTGCATTCCCATGTAGCATTTTAATTGAAGTCTGTAAATGTGGCTAAAAGTCTTGTCTTATTTTTTGAGACAGGGTCTTGCCTCACCCAAGCTAGA
GTGCAGTGGCCTTTGAAGCTTACTACAGCCTCAAACTTCTGGGCTCAAGTGATCCTCAGCCTCCCAGTGGTCT
>ENST00000449589.5|ENSG00000234741.7|OTTHUMG00000037216.5|OTTHUMT00000090586.1|RP5-1198E17.2-010|GAS5|712|
CTTTTCGAGGTAGGAGTCGACTCCTGTGAGGTATGGTGCTGGGTGCAGATGCAGTGTGGCTCTGGATAGCACCTTATGGACAGTTGTGTCCCCAAGGA
AGGATGAGAATAGCTACTGAAGTAAGTTGAAAATTCCCTCTCAAAAAGGTTTAAAGCCATTGGATGTGCCACAATGATGACAGTTTATTTGCTACTCTT
GAGTGCTAGAATGATGAGGATCTTAACCACCATTATCTTAACTGAGGCACCCAAAATGGTGAGTTGGGGAACATAGAGAGTACACCTAAGTTCACATG
AAGTTGTTTCTTCCCAGGTCCTAAAGAGCAAGCCTAACTCAAGCCATTGGCACACAGGCATTAGACAGAAAGCTGGAAGTTGAAATGGTGGAGTCCA
ACTTGCCTGGACCAGCTTAATGGTTCTGCTCCTGGTAACGTTTTTATCCATGGATGACTTGCTTGGGTAAGGACATGAAGACAGTTCCTGTCATACCTTT
TAAAGGTATGGAGAGTCGGCTTGACTACACTGTGTGGAGCAAGTTTTAAAGAAGCAAAGGACTCAGAATTCATGATTGAAGAAATGCAGGCAGACCT
GTTATCCTAAACTAGGGTTTTTAATGACCACAACAAGCAAGCATGCAGCTTACTGCTTGAAAGGGTCTTGCCTCACCCAAGCTAGAGTGCAGTGGCCTT
TGAAGCTTACTACAGCCTCAAACT
>ENST00000451607.5|ENSG00000234741.7|OTTHUMG00000037216.5|OTTHUMT00000090591.2|RP5-1198E17.2-015|GAS5|1007|
CTGTGTTGGTAGGGACATCTGAGAGTAATGATGAATGCCAACCGCTCTGATGGTGGCACATGCCGAGTCACCCGAGTAAGCTATTGTTAAGGGCCGTG
ACCCGAGCCTCCATCAGCCGTCCGCTCTCATGAAAGGCTGTCGGTGGTAGTCCACGTGCTTAAGTGCCTGCATTCCGCAGTGTCACCAATATTTCCATT
AGTGTTTCTTTTTTCTTTTTTGAGACCGAGCCTCGTTCTGTCACCCAGGCTGGAATGCAGTGGCTCGACATCGGTTAATGGCAACCTCCGCGTCCCGAG
TTAAGCAGTTCTGCCTCAGCCTCCCAAGTAACTGGGACTACAGGCGCGCGCCACCATGCTAGGCTTTTATATTTTTAGTAGAGACGGGGTTTCACCATG
TTGGCGAGACTGGTCTTGAACTCCTGACCTCAGGTGATCCACCAGCCGTGGTCCCCCAACATACTGGGATTACAAGCCGTGAGCCACCGCGCCCGGCC
GCCATCGGTGGTTCTTAACTGCGGGTGCAGTGCTTCTTTGTAACATTAAGTGTATCCTTTACCTGTCGCTAGATAATGAATGGTATGGTGCTGGGTGCAG
ATGCAGTGTGGCTCTGGATAGCACCTTATGGACAGTTGTGTCCCCAAGGAAGGATGAGAATAGCTACTGAAGTCCTAAAGAGCAAGCCTAACTCAAGC
CATTGGCACACAGGCATTAGACAGAAAGCTGGAAGTTGAAATGGTGGAGTCCAACTTGCCTGGACCAGCTTAATGGTTCTGCTCCTGGTAACGTTTTT
ATCCATGGATGACTTGCTTGGGTATGGAGAGTCGGCTTGACTACACTGTGTGGAGCAAGTTTTAAAGAAGCAAAGGACTCAGAATTCATGATTGAAGA
AATGCAGGCAGACCTGTTATCCTAAACTAGGGTTTTTAATGACCACAACAAGCAAGCATGCAGCTTACTGCTTGAAAGGGTCTTGCCTCACCCAAGCT
AGAGTGCAGTGGCCTTTGAAGC

>ENST00000422008.5|ENSG00000234741.7|OTTHUMG00000037216.5|OTTHUMT00000090597.1|RP5-1198E17.2-021|GAS5|497|
TGCAGATGCAGTGTGGCTCTGGATAGCACCTTATGGACAGTTGTGTCCCCAAGGAAGGATGAGAATAGCTACTGAAGTCCTAAAGAGCAAGCCTAACT
CAAGCCATTGGCACACAGGCATTAGACAGAAAGCTGGAAGTTGAAATGGTGGAGTCCAACTTGCCTGGACCAGCTTAATGGTTCTGCTCCTGGTAACG
TTTTTATCCATGGATGACTTGCTTGGGTATGGAGAGTCGGCTTGACTACACTGTGTGGAGCAAGTTTTAAAGAAGCAAAGGACTCAGAATTCATGATTG
AAGAAATGCAGGTTAGTTTAAACTTTGAAGGAAATTTTTAAGGTGGCAAAAGGTTTTGGTGGCATATACACCTTAATCTGTAGATGGGAGTGATTAGCT
GTTTAAAAGTTAAAATGTGACTGAGAAGGAAATTGAGTAGGGCAAATTTTAAATGGGTATTATTTTTCATCTTCAAACAGGCAGACCTGTTATCCTAAAC
TAG
>ENST00000442067.5|ENSG00000234741.7|OTTHUMG00000037216.5|OTTHUMT00000090594.1|RP5-1198E17.2-018|GAS5|1114|
GTATGGTGCTGGGTGCAGATGCAGTGTGGCTCTGGATAGCACCTTATGGACAGTTGTGTCCCCAAGGAAGGATGAGAATAGCTACTGAAGTCCTAAAG
AGCAAGCCTAACTCAAGCCATTGGCACACAGGCATTAGACAGAAAGCTGGAAGTTGAAATGGTGGAGTCCAACTTGCCTGGACCAGCTTAATGGTTC
TGCTCCTGGTAACGTTTTTATCCATGGATGACTTGCTTGGGTAAGGACATGAAGACAGTTCCTGTCATACCTTTTAAAGGTACATGTTTTATTGATGTTAA
CGTTAATTGATTGAGCTACTGTTAGTGATGATTTTAAAATTAAAGCAGATGGGAATCTCTCTGAGAAAGAAAATGGAGATTAATCTTAAACTGAAACAGT
AGTTGGGAAATCTTTTAGAAATCCACCTATTACTACCTATTGGTAAAGGAGATTAAATTTCTACAGGTATGGAGAGTCGGCTTGACTACACTGTGTGGAG
CAAGTTTTAAAGAAGCAAAGGTATAGCAGTTCCAAGTATTTTTTTTTTTTTTTAGACAAGAGTCTAGCTCTTGCCCAGAATGGAGTGCAGCGGCACTA
TCAGTTCACTGCAACCTCTGCCTCCCAGGTTCAAGGAATTCTCCTGCCTCAGCCTCTTGAGTAGCTGGGATTACAGGCATGTGACACCATGCCTGGCTA
ATTTTGTACAGCTATGTTGTCCAGGCTGGTCTCGAACTCTTGACCTCAAGTGATACTGCCCGCACTGACCTCCCAAAGTGCTGGGATTACAGGCGTGAG
CCACCATGCCCCGCCTCAAGTCTGGTTTTTAAGTGTTGTAAAGCCGATACAATGATGATAACATAGTTCAGCAGACTAACGCTGATGAGCAATATTAAGT
CTTTCGCTCCTATCTGATGTATCTGGCGGTAACATTCTAGTTTATGCCCCGAAAAGGGGAATATAGCCATTCTATAATGTTTGGAGATTTTGGATTACTCCT
AATTGTATGCAAGTTGTCTTACTGTGTATTGTCCCTTAATTTCAGGACTCAGAATTCATGATTGAAGAAATGCAGGTTAGTTTAAACTTTGAAGGAAATT
TTTAAGGTGGCAAAAGGTT
>ENST00000455838.5|ENSG00000234741.7|OTTHUMG00000037216.5|OTTHUMT00000090585.1|RP5-1198E17.2-009|GAS5|632|
CTTTTCGAGGTAGGAGTCGACTCCTGTGAGGTATGGTGCTGGGTGCAGATGCAGTGTGGCTCTGGATAGCACCTTATGGACAGTTGTGTCCCCAAGGA
AGGATGAGAATAGCTACTGAAGTCCTAAAGAGCAAGCCTAACTCAAGCCATTGGCACACAGGCATTAGACAGAAAGCTGGAAGTTGAAATGGTGGAG
TCCAACTTGCCTGGACCAGCTTAATGGTTCTGCTCCTGGTAACGTTTTTATCCATGGATGACTTGCTTGGGTAAGGACATGAAGACAGTTCCTGTCATAC
CTTTTAAAGGTATGGAGAGTCGGCTTGACTACACTGTGTGGAGCAAGTTTTAAAGAAGCAAAGGTATAGCAGTTCCAAGTATTTTTTTTTTTTTTTAGA
CAAGAGTCTAGCTCTTGCCCAGAATGGAGTGCAGCGGCACTATCAGTTCACTGCAACCTCTGCCTCCCAGGTTCAAGGAATTCTCCTGCCTCAGCCTC

TTGAGTAGCTGGGATTACAGGCATGTGACACCATGCCTGGCTAATTTTGTACAGCTATGTTGTCCAGGCTGGTCTCGAACTCTTGACCTCAAGTGATACT
GCCCGCACTGACCTCCCAAAGTGCTGGGATTACAGGCGT
>ENST00000416952.5|ENSG00000234741.7|OTTHUMG00000037216.5|OTTHUMT00000090588.1|RP5-1198E17.2-012|GAS5|799|
TTTTCGAGGTAGGAGTCGACTCCTGTGAGGTATGGTGCTGGGTGCAGATGCAGTGTGGCTCTGGATAGCACCTTATGGACAGTTGTGTCCCCAAGGAA
GGATGAGAATAGCTACTGAAGTAAGTTGAAAATTCCCTCTCAAAAAGGTTTAAAGCCATTGGATGTGCCACAATGATGACAGTTTATTTGCTACTCTTG
AGTGCTAGAATGATGAGGATCTTAACCACCATTATCTTAACTGAGGCACCCAAAATGGTGAGTTGGGGAACATAGAGAGTACACCTAAGTTCACATGA
AGTTGTTTCTTCCCAGGTCCTAAAGAGCAAGCCTAACTCAAGCCATTGGCACACAGGCATTAGACAGAAAGCTGGAAGTTGAAATGGTGGAGTCCAA
CTTGCCTGGACCAGCTTAATGGTTCTGCTCCTGGTAACGTTTTTATCCATGGATGACTTGCTTGGGTAAGGACATGAAGACAGTTCCTGTCATACCTTTT
AAAGGTACATGTTTTATTGATGTTAACGTTAATTGATTGAGCTACTGTTAGTGATGATTTTAAAATTAAAGCAGATGGGAATCTCTCTGAGAAAGAAAAT
GGAGATTAATCTTAAACTGAAACAGTAGTTGGGAAATCTTTTAGAAATCCACCTATTACTACCTATTGGTAAAGGAGATTAAATTTCTACAGGTATGGAG
AGTCGGCTTGACTACACTGTGTGGAGCAAGTTTTAAAGAAGCAAAGGTATAGCAGTTCCAAGTATTTTTTTTTTTTTTTAGACAAGAGTCTAGCTCTT
GCCCAGAA
>ENST00000432536.5|ENSG00000234741.7|OTTHUMG00000037216.5|OTTHUMT00000090592.2|RP5-1198E17.2-016|GAS5|959|
ACCGCTCTGATGGTGGCACATGCCGAGTCACCCGAGTAAGCTATTGTTAAGGGCCGTGACCCGAGCCTCCATCAGCCGTCCGCTCTCATGAAAGGCTG
TCGGTGGTAGTCCACGTGCTTAAGTGCCTGCATTCCGCAGTGTCACCAATATTTCCATTAGTGTTTCTTTTTTCTTTTTTGAGACCGAGCCTCGTTCTGTC
ACCCAGGCTGGAATGCAGTGGCTCGACATCGGTTAATGGCAACCTCCGCGTCCCGAGTTAAGCAGTTCTGCCTCAGCCTCCCAAGTAACTGGGACTAC
AGGCGCGCGCCACCATGCTAGGCTTTTATATTTTTAGTAGAGACGGGGTTTCACCATGTTGGCGAGACTGGTCTTGAACTCCTGACCTCAGGTGATCCA
CCAGCCGTGGTCCCCCAACATACTGGGATTACAAGCCGTGAGCCACCGCGCCCGGCCGCCATCGGTGGTTCTTAACTGCGGGTGCAGTGCTTCTTTGT
AACATTAAGTGTATCCTTTACCTGTCGCTAGATAATGAATGGTATGTTACCTGCATCATTGGTTTAAAAAGACGAACCGTTTTTTTAAAGAACACTCTTTA
AAAAAAAGAACCGTGGAACAATGAATTAAAATCTGTACCTGATCTCTTTAGGTATGGTGCTGGGTGCAGATGCAGTGTGGCTCTGGATAGCACCTTATG
GACAGTTGTGTCCCCAAGGAAGGATGAGAATAGCTACTGAAGTCCTAAAGAGCAAGCCTAACTCAAGCCATTGGCACACAGGCATTAGACAGAAAGC
TGGAAGTTGAAATGGTGGAGTCCAACTTGCCTGGACCAGCTTAATGGTTCTGCTCCTGGTAACGTTTTTATCCATGGATGACTTGCTTGGGTAAGGACA
TGAAGACAGTTCCTGTCATACCTTTTAAAGGTATGGAGAGTCGGCTTGACTACACTGTGTGGAGCAAGT
>ENST00000456293.5|ENSG00000234741.7|OTTHUMG00000037216.5|OTTHUMT00000090583.1|RP5-1198E17.2-007|GAS5|583|
GTCTTTTCGAGGTAGGAGTCGACTCCTGTGAGGTATGGTGCTGGGTGCAGATGCAGTGTGGCTCTGGATAGCACCTTATGGACAGTTGTGTCCCCAAG
GAAGGATGAGAATAGCTACTGAAGTCCTAAAGAGCAAGCCTAACTCAAGCCATTGGCACACAGGCATTAGACAGAAAGCTGGAAGTTGAAATGGTAA

GTGAAACTGTATCCAAGTAAGCAGGTAACTGGGCAAACTTCCTACGGCACAAATGGCTTTTTAGTTACCTCCTAGTGCTGAATGCATTAAATAAATGGC
GGATTCTTGTCTTGTTATGATTAATAAGAAAGTTTGTAAATGCAGCCTGGATGATGATAAGCAAATGCTGACTGAACATGAAGGTCTTAATTAGCTCTAA
CTGACTAAAGGCATTTGTTAGTTTTGGCAGGGGGTGAACACTCATCTGTGGCTATTCTAAGACCACTCTTATTTCTTAGGTGGAGTCCAACTTGCCTGG
ACCAGCTTAATGGTTCTGCTCCTGGTAACGTTTTTATCCATGGATGACTTGCTTGGGTATGGAGAGTCGGCTTGACTACACTGTGTGGAG
>ENST00000443799.5|ENSG00000234741.7|OTTHUMG00000037216.5|OTTHUMT00000090587.2|RP5-1198E17.2-011|GAS5|897|
CTTTTCGAGGTAGGAGTCGACTCCTGTGAGGTATGGTGCTGGGTGCAGATGCAGTGTGGCTCTGGATAGCACCTTATGGACAGTTGTGTCCCCAAGGA
AGGATGAGAATAGCTACTGAAGTAAGTTGAAAATTCCCTCTCAAAAAGGTTTAAAGCCATTGGATGTGCCACAATGATGACAGTTTATTTGCTACTCTT
GAGTGCTAGAATGATGAGGATCTTAACCACCATTATCTTAACTGAGGCACCCAAAATGGTGAGTTGGGGAACATAGAGAGTACACCTAAGTTCACATG
AAGTTGTTTCTTCCCAGGTCCTAAAGAGCAAGCCTAACTCAAGCCATTGGCACACAGGCATTAGACAGAAAGCTGGAAGTTGAAATGGTAAGTGAAA
CTGTATCCAAGTAAGCAGGTAACTGGGCAAACTTCCTACGGCACAAATGGCTTTTTAGTTACCTCCTAGTGCTGAATGCATTAAATAAATGGCGGATTCT
TGTCTTGTTATGATTAATAAGAAAGTTTGTAAATGCAGCCTGGATGATGATAAGCAAATGCTGACTGAACATGAAGGTCTTAATTAGCTCTAACTGACTA
AAGGCATTTGTTAGTTTTGGCAGGGGGTGAACACTCATCTGTGGCTATTCTAAGACCACTCTTATTTCTTAGGTGGAGTCCAACTTGCCTGGACCAGCTT
AATGGTTCTGCTCCTGGTAACGTTTTTATCCATGGATGACTTGCTTGGGTAAGGACATGAAGACAGTTCCTGTCATACCTTTTAAAGGTACATGTTTTATT
GATGTTAACGTTAATTGATTGAGCTACTGTTAGTGATGATTTTAAAATTAAAGCAGATGGGAATCTCTCTGAGAAAGAAAATGGAGATTAATCTTAAACT
GAAA
>ENST00000421068.5|ENSG00000234741.7|OTTHUMG00000037216.5|OTTHUMT00000090582.1|RP5-1198E17.2-006|GAS5|1060|
TTTCGAGGTAGGAGTCGACTCCTGTGAGGTATGGTGCTGGGTGCAGATGCAGTGTGGCTCTGGATAGCACCTTATGGACAGTTGTGTCCCCAAGGAAG
GATGAGAATAGCTACTGAAGTAAGTTGAAAATTCCCTCTCAAAAAGGTTTAAAGCCATTGGATGTGCCACAATGATGACAGTTTATTTGCTACTCTTGA
GTGCTAGAATGATGAGGATCTTAACCACCATTATCTTAACTGAGGCACCCAAAATGGTGAGTTGGGGAACATAGAGAGTACACCTAAGTTCACATGAA
GTTGTTTCTTCCCAGGTCCTAAAGAGCAAGCCTAACTCAAGCCATTGGCACACAGGTGAGACACCTCTATTTTGTACTTCTCACTTTTAAGGGATTAGA
AAATAGCCAAAGCAATGATGATTATCTATGTTAGTGCTTCTCTCCCCTCTTTTCAAATGAGAATTTTGCTCTCATATTGATACTAAGTTTAATACTGAAGA
AAATGTGAAAACAGATACTATGATGGTTGCATAGTTCAGCAGATTTAATCATGAAGAGATGTACTATCTGTCTGATGTATCTGGGGTAGTTGTGGTTTGCT
GTTAATGGTTAAGCAGTGTACCACCAATCTACCATTAAAATATTTTTTGCTGACAATTTTGTATTAAAATTACAGGCATTAGACAGAAAGCTGGAAGTTG
AAATGGTAAGTGAAACTGTATCCAAGTAAGCAGGTAACTGGGCAAACTTCCTACGGCACAAATGGCTTTTTAGTTACCTCCTAGTGCTGAATGCATTAA
ATAAATGGCGGATTCTTGTCTTGTTATGATTAATAAGAAAGTTTGTAAATGCAGCCTGGATGATGATAAGCAAATGCTGACTGAACATGAAGGTCTTAAT
TAGCTCTAACTGACTAAAGGCATTTGTTAGTTTTGGCAGGGGGTGAACACTCATCTGTGGCTATTCTAAGACCACTCTTATTTCTTAGGTGGAGTCCAAC

TTGCCTGGACCAGCTTAATGGTTCTGGTAAGTATTAATGAAAACAGTAGATAGACTTAATGAAAA
>ENST00000425771.5|ENSG00000234741.7|OTTHUMG00000037216.5|OTTHUMT00000090596.1|RP5-1198E17.2-020|GAS5|242|
TGGTGCTGGGTGCAGATGCAGTGTGGCTCTGGATAGCACCTTATGGACAGTTGTGTCCCCAAGGAAGGATGAGAATAGCTACTGAAGTCCTAAAGAGC
AAGCCTAACTCAAGCCATTGGCACACAGGCATTAGACAGAAAGCTGGAAGTTGAAATGTTTTGGCAGGGGGTGAACACTCATCTGTGGCTATTCTAAG
ACCACTCTTATTTCTTAGGTGGAGTCCAACTTGCCTGGACCAGCTT
>ENST00000458220.1|ENSG00000234741.7|OTTHUMG00000037216.5|OTTHUMT00000090581.1|RP5-1198E17.2-005|GAS5|469|
TTTCGAGGTAGGAGTCGACTCCTGTGAGGTATGGTGCTGGGTGCAGATGCAGTGTGGCTCTGGATAGCACCTTATGGACAGTTGTGTCCCCAAGGAAG
GATGAGAATAGCTACTGAAGTCCTAAAGAGCAAGCCTAACTCAAGCCATTGGCACACAGGTGAGACACCTCTATTTTGTACTTCTCACTTTTAAGGGAT
TAGAAAATAGCCAAAGCAATGATGATTATCTATGTTAGTGCTTCTCTCCCCTCTTTTCAAATGAGAATTTTGCTCTCATATTGATACTAAGTTTAATACTGA
AGAAAATGTGAAAACAGATACTATGATGGTTGCATAGTTCAGCAGATTTAATCATGAAGAGATGTACTATCTGTCTGATGTATCTGGGGTAGTTGTGGTT
TGCTGTTAATGGTTAAGCAGTGTACCACCAATCTACCATTAAAATATTTTTTGCTGACAATTTTGTATTA
>MIR155HG
>ENST00000456917.1|ENSG00000234883.5|OTTHUMG00000078367.1|OTTHUMT00000171187.1|AP000223.5-001|MIR155HG|1600|
TACACACACGCAATGACCCACGAGAAAGGGAAAGGGGAAAACACCAACTACCCGGGCGCTGGGCTTTTTCGACTTTTCCTTTAAAAAGAAAAAAGTT
TTTCAAGCTGCGCGGGCTTCCTGTGCGCGGCCGAGCCCGGGCCCAGCGCCGCCTGCAGCCTCGGGAAGGGAGCGGATAGCGGAGCCCCGAGCCGCC
CGCAGAGCAAGCGCGGGGAACCAAGGAGACGCTCCTGGCACTGCAGATAACTTGTCTGCATTTCAAGAACAACCTACCAGAGACCTTACCTGTCACC
TTGGCTCTCCCACCCAATGGAGATGGCTCTAATGGTGGCACAAACCAGGAAGGGGAAATCTGTGGTTTAAATTCTTTATGCCTCATCCTCTGAGTGCTG
AAGGCTTGCTGTAGGCTGTATGCTGTTAATGCTAATCGTGATAGGGGTTTTTGCCTCCAACTGACTCCTACATATTAGCATTAACAGTGTATGATGCCTGT
TACTAGCATTCACATGGAACAAATTGCTGCCGTGGGAGGATGACAAAGAAGCATGAGTCACCCTGCTGGATAAACTTAGACTTCAGGCTTTATCATTTT
TCAATCTGTTAATCATAATCTGGTCACTGGGATGTTCAACCTTAAACTAAGTTTTGAAAGTAAGGTTATTTAAAAGATTTATCAGTAGTATCCTAAATGCA
AACATTTTCATTTAAATGTCAAGCCCATGTTTGTTTTTATCATTAACAGAAAATATATTCATGTCATTCTTAATTGCAGGTTTTGGCTTGTTCATTATAATGT
TCATAAACACCTTTGATTCAACTGTTAGAAATGTGGGCTAAACACAAATTTCTATAATATTTTTGTAGTTAAAAATTAGAAGGACTACTAACCTCCAGTTA
TATCATGGATTGTCTGGCAACGTTTTTTAAAAGATTTAGAAACTGGTACTTTCCCCCAGGTAACGATTTTCTGTTCAGGCAACTTCAGTTTAAAATTAATA
CTTTTATTTGACTCTTAAAGGGAAACTGAAAGGCTATGAAGCTGAATTTTTTTAATGAAATATTTTTAACAGTTAGCAGGGTAAATAACATCTGACAGCT
AATGAGATATTTTTTCCATACAAGATAAAAAGATTTAATCAAAAAATTTCATATTTGAAATGAAGTCCCAAATCTAGGTTCAAGTTCAATAGCTTAGCCA
CATAATACGGTTGTGCGAGCAGAGAATCTACCTTTCCACTTCTAAGCCTGTTTCTTCCTCCATATGGGGATAATACTTTACAAGGTTGTTGTGAGGCTTA

GATGAGATAGAGAATTATTCCATAAGATAATCAAGTGCTACATTAATGTTATAGTTAGATTAATCCAAGAACTAGTCACCCTACTTTATTAGAGAAGAGAA
AAGCTAATGATTTGATTTGCAGAATATTTAAGGTTTGGATTTCTATGCAGTTTTTCTAAATAACCATCACTTACAAATATGTAACCAAACGTAATTGTTAGT
ATATTTAATGTAAACTTGTTTTAACAACTCTTCTCAACATTTTGTCCAGGTTATTCACTGTAACCAAATAAATCTCATGAGTCTTTAGTTGATTTAAAATAA

>HOTAIR

>ENST00000424518.5|ENSG00000228630.5|OTTHUMG00000152934.1|OTTHUMT00000328662.1|AC012531.8-001|HOTAIR|2421|

CCAGTTCTCAGGCGAGAGCCGCGGCTGACAGGGTCTGGGACAGAAGGAAAGCCCTCCAGCCTCCAGGCCCTGCCTTCTGCCTGCACATTCTGCCCTG
ATTTCCGGAACCTGGAAGCCTAGGCAGGCAGTGGGGAACTCTGACTCGCCTGTGCTCTGGAGCTTGATCCGAAAGCTTCCACAGTGAGGACTGCTCC
GTGGGGGTAAGAGAGCACCAGGCACTGAGGCCTGGGAGTTCCACAGACCAACACCCCTGCTCCTGGCGGCTCCCACCCGGGACTTAGACCCTCAGG
TCCCTAATATCCCGGAGGTGCTCTCAATCAGAAAGGTCCTGCTCCGCTTCGCAGTGGAATGGAACGGATTTAGAAGCCTGCAGTAGGGGAGTGGGGAG
TGGAGAGAGGGAGCCCAGAGTTACAGACGGCGGCGAGAGGAAGGAGGGGCGTCTTTATTTTTTTAAGGCCCCAAAGAGTCTGATGTTTACAAGACCA
GAAATGCCACGGCCGCGTCCTGGCAGAGAAAAGGCTGAAATGGAGGACCGGCGCCTTCCTTATAAGTATGCACATTGGCGAGAGAAGTGCTGCAACC
TAAACCAGCAATTACACCCAAGCTCGTTGGGGCCTAAGCCAGTACCGACCTGGTAGAAAAAGCAACCACGAAGCTAGAGAGAGAGCCAGAGGAGGG
AAGAGAGCGCCAGACGAAGGTGAAAGCGAACCACGCAGAGAAATGCAGGCAAGGGAGCAAGGCGGCAGTTCCCGGAACAAACGTGGCAGAGGGC
AAGACGGGCACTCACAGACAGAGGTTTATGTATTTTTATTTTTTAAAATCTGATTTGGTGTTCCATGAGGAAAAGGGAAAATCTAGGGAACGGGAGTAC
AGAGAGAATAATCCGGGTCCTAGCTCGCCACATGAACGCCCAGAGAACGCTGGAAAAACCTGAGCGGGTGCCGGGGCAGCACCCGGCTCGGGTCAG
CCACTGCCCCACACCGGGCCCACCAAGCCCCGCCCCTCGCGGCCACCGGGGCTTCCTTGCTCTTCTTATCATCTCCATCTTTATGATGAGGCTTGTTAAC
AAGACCAGAGAGCTGGCCAAGCACCTCTATCTCAGCCGCGCCCGCTCAGCCGAGCAGCGGTCGGTGGGGGGACTGGGAGGCGCTAATTAATTGATTC
CTTTGGACTGTAAAATATGGCGGCGTCTACACGGAACCCATGGACTCATAAACAATATATCTGTTGGGCGTGAGTGCACTGTCTCTCAAATAATTTTTCC
ATAGGCAAATGTCAGAGGGTTCTGGATTTTTAGTTGCTAAGGAAAGATCCAAATGGGACCAATTTTAGGAGGCCCAAACAGAGTCCGTTCAGTGTCAG
AAAATGCTTCCCCAAAGGGGTTGGGAGTGTGTTTTGTTGGAAAAAAGCTTGGGTTATAGGAAAGCCTTTCCCTGCTACTTGTGTAGACCCAGCCCAAT
TTAAGAATTACAAGGAAGCGAAGGGGTTGTGTAGGCCGGAAGCCTCTCTGTCCCGGCTGGATGCAGGGGACTTGAGCTGCTCCGGAATTTGAGAGGA
ACATAGAAGCAAAGGTCCAGCCTTTGCTTCGTGCTGATTCCTAGACTTAAGATTCAAAAACAAATTTTTAAAAGTGAAACCAGCCCTAGCCTTTGGAA
GCTCTTGAAGGTTCAGCACCCACCCAGGAATCCACCTGCCTGTTACACGCCTCTCCAAGACACAGTGGCACCGCTTTTCTAACTGGCAGCACAGAGCA
ACTCTATAATATGCTTATATTAGGTCTAGAAGAATGCATCTTGAGACACATGGGTAACCTAATTATATAATGCTTGTTCCATACAGGAGTGATTATGCAGTG
GGACCCTGCTGCAAACGGGACTTTGCACTCTAAATATAGACCCCAGCTTGGGACAAAAGTTGCAGTAGAAAAATAGACATAGGAGAACACTTAAATAA
GTGATGCATGTAGACACAGAAGGGGTATTTAAAAGACAGAAATAATAGAAGTACAGAAGAACAGAAAAAAAATCAGCAGATGGAGATTACCATTCCC

AATGCCTGAACTTCCTCCTGCTATTAAGATTGCTAGAGAATTGTGTCTTAAACAGTTCATGAACCCAGAAGAATGCAATTTCAATGTATTTAGTACACAC
ACAGTATGTATATAAACACAACTCACAGAATATATTTTCCATACATTGGGTAGGTATGCACTTTGTGTATATATAATAATGTATTTTCCATGCAGTTTTAAAA
TGTAGATATATTAATATCTGGATGCATTTTCTGTGCACTGGTTTTATATGCCTTATGGAGTATATACTCACATGTAGCTAAATAGACTCAGGACTGCACATTC
CTTGTGTAGGTTGTGTGTGTGTGGTGGTTTTATGCATAAATAAAGTTTTACATGTGGTGAATATA
>ENST00000455246.5|ENSG00000228630.5|OTTHUMG00000152934.1|OTTHUMT00000328663.1|AC012531.8-002|HOTAIR|918|
AGGCGAGAGCCGCGGCTGACAGGGTCTGGGACAGAAGGAAAGCCCTCCAGCCTCCAGGCCCTGCCTTCTGCCTGCACATTCTGCCCTGATTTCCGGA
ACCTGGAAGCCTAGGCAGGCAGTGGGGAACTCTGACTCGCCTGTGCTCTGGAGCTTGATCCGAAAGCTTCCACAGTGAGGACTGCTCCGTGGGGGTA
AGAGAGCACCAGGCACTGAGGCCTGGGAGTTCCACAGACCAACACCCCTGCTCCTGGCGGCTCCCACCCGGGACTTAGACCCTCAGGTCCCTAATAT
CCCGGAGGTGCTCTCAATCAGAAAGGTCCTGCTCCGCTTCGCAGTGGAATGGAACGGATTTAGAAGCCTGCAGTAGGGGAGTGGGGAGTGGAGAGA
GGGAGCCCAGAGTTACAGACGGCGGCGAGAGGAAGGAGGGGCGTCTTTATTTTTTTAAGGCCCCAAAGAGTCTGATGTTTACAAGACCAGAAATGCC
ACGGCCGCGTCCTGGCAGAGAAAAGGCTGAAATGGAGGACCGGCGCCTTCCTTATAAGCTCGTTGGGGCCTAAGCCAGTACCGACCTGGTAGAAAAA
GCAACCACGAAGCTAGAGAGAGAGCCAGAGGAGGGAAGAGAGCGCCAGACGAAGGTGAAAGCGAACCACGCAGAGAAATGCAGGCAAGGGAGCA
AGGCGGCAGTTCCCGGAACAAACGTGGCAGAGGGCAAGACGGGCACTCACAGACAGAGGTTTATGTATTTTTATTTTTTAAAATCTGATTTGGTGTTC
CATGAGGAAAAGGGAAAATCTAGGGAACGGGAGTACAGAGAGAATAATCCGGGTCCTAGCTCGCCACATGAACGCCCAGAGAACGCTGGAAAAACC
TGAGCGGGTGCCGGGGCAGCACCCGGCTCGGGTCAGCCACTGCCCCACA
>ENST00000425595.5|ENSG00000228630.5|OTTHUMG00000152934.1|OTTHUMT00000328666.1|AC012531.8-005|HOTAIR|572|
ATCTTTCTTTGCTAGCTGCCCGGGTCCTGCTCCGCTTCGCAGTGGAATGGAACGGATTTAGAAGCCTGCAGTAGGGGAGTGGGGAGTGGAGAGAGGG
AGCCCAGAGTTACAGACGGCGGCGAGAGGAAGGAGGGGCGTCTTTATTTTTTTAAGGCCCCAAAGAGTCTGATGTTTACAAGACCAGAAATGCCACG
GCCGCGTCCTGGCAGAGAAAAGGCTGAAATGGAGGACCGGCGCCTTCCTTATAAGCTCGTTGGGGCCTAAGCCAGTACCGACCTGGTAGAAAAAGCA
ACCACGAAGCTAGAGAGAGAGCCAGAGGAGGGAAGAGAGCGCCAGACGAAGGTGAAAGCGAACCACGCAGAGAAATGCAGGCAAGGGAGCAAGG
CGGCAGTTCCCGGAACAAACGTGGCAGAGGGCAAGACGGGCACTCACAGACAGAGGTTTATGTATTTTTATTTTTTAAAATCTGATTTGGTGTTCCATG
AGGAAAAGGGAAAATCTAGGGAACGGGAGTACAGAGAGAATAATCCGGGTCCTAGCTCGCCACATGAACGCCCAGAGAACGCTGGAAA
>ENST00000453875.5|ENSG00000228630.5|OTTHUMG00000152934.1|OTTHUMT00000328665.1|AC012531.8-004|HOTAIR|562|
AGACCAACACCCCTGCTCCTGGCGGCTCCCACCCGGGACTTAGACCCTCAGGTCCCTAATATCCCGGAGGTGCTCTCAATCAGAAAGGTCCTGCTCCG
CTTCGCAGTGGAATGGAACGGATTTAGAAGCCTGCAGTAGGGGAGTGGGGAGTGGAGAGAGGGAGCCCAGAGTTACAGACGGCGGCGAGAGGCCCC
AAAGAGTCTGATGTTTACAAGACCAGAAATGCCACGGCCGCGTCCTGGCAGAGAAAAGGCTGAAATGGAGGACCGGCGCCTTCCTTATAAGTATGCA

CATTGGCGAGAGAAGTGCTGCAACCTAAACCAGCAATTACACCCAAGCTCGTTGGGGCCTAAGCCAGTACCGACCTGGTAGAAAAAGCAACCACGAA
GCTAGAGAGAGAGCCAGAGGAGGGAAGAGAGCGCCAGACGAAGGTGAAAGCGAACCACGCAGAGAAATGCAGGCAAGGGAGCAAGGCGGCAGTT
CCCGGAACAAACGTGGCAGAGGGCAAGACGGGCACTCACAGACAGAGGTTTATGTATTTTTATTTTTTAAAATCTGATTT
>ENST00000439545.1|ENSG00000228630.5|OTTHUMG00000152934.1|OTTHUMT00000328664.1|AC012531.8-003|HOTAIR|560|
CTAATCAATCCAAAAGGAAAAAGAAAAGAGAGGGGTGGGAAGGCATGGGGTGAAAAACTTCAGGTGACACAAAGCAAAAAGATTCCAGTCACCAA
AAACAAAAACAAAAAAAAATCTTTTGCCCCCAGCAAGAATCATTTGTTTGGGATCTGTTCCAGCCTCCAGGCCCTGCCTTCTGCCTGCACATTCTGCCC
TGATTTCCGGAACCTGGAAGCCTAGGCAGGCAGTGGGGAACTCTGACTCGCCTGTGCTCTGGAGCTTGATCCGAAAGCTTCCACAGTGAGGACTGCT
CCGTGGGGTCCTGCTCCGCTTCGCAGTGGAATGGAACGGATTTAGAAGCCTGCAGTAGGGGAGTGGGGAGTGGAGAGAGGGAGCCCAGAGTTACAG
ACGGCGGCGAGAGGAGGGGCGTCTTTATTTTTTTAAGGCCCCAAAGAGTCTGATGTTTACAAGACCAGAAATGCCACGGCCGCGTCCTGGCAGAGAA
AAGGCTGAAATGGAGGACCGGCGCCTTCCTTATAAGCTCGTTGGGGCCTAAGCCAGTACCGACCTGGTAGAAAAAG
>CASC2
>ENST00000414722.5|ENSG00000177640.15|OTTHUMG00000019127.8|OTTHUMT00000050581.1|CTA-287C20.1-005|CASC2|679|
CAGGGCTCCCCGACTTCCCTATGGCTGATGTCAAAACGCCTCGCGGGGGCAGCCCAGGGGCACGGCCGCTCCGGGGGTCCCCTTTCGTCTGGAGAAA
CACAGAGGCCCACCATCACCTGGCCGCGCCGTGCAGGCCTCTGCGCGGACCCCCGCCCCTGTCTCCACCTTCCCCAGGCCTGCGGGGCACGTGAGGC
CTGGCCACACGGACCCGGGCGGAGGGCTGCGGGGGTGAAGGGAGCCCCCGCCCCAATTCTGCACCCCTGGCCTCCCGCCTGCCTCCTTGCGGCCGAG
CAGCCTCCGCTGCCTCCGCGCCTCCCGCCCGCCCGGCGTTCGTCCGCAGGCTCGGCCCGGCTCCTCCTCCCGACCCCGATGGAGATTCAGAAACATAA
GGACAACAAGAAACTTCCCCAAGGTATCATTATAGTCTTTAGACTTCAGACACACACCACACCTCAAATATATACACAACTGAAAGGAAAATTAAGGA
AGTTTTTCAAAGAACCCTATTCCGAGTAAGAAGTGTGTTGCATGAATTTCTAAGAGCCAGAAAATGCATGACACAGGAGAAGATGTACCCTCATCTGTT
CAGTGAGAGATGTGCAAATCAACATCAACACAGAACTGCTGAAGAAAAAAAAATATGTGTGTGGATTATATATATACATATAAGTGTGTGTATA
>ENST00000435944.5|ENSG00000177640.15|OTTHUMG00000019127.8|OTTHUMT00000050577.5|CTA-287C20.1-001|CASC2|3270|
GGGCTGCAGGGCTGCGGGCGCTTGGTTCGGCCTGGCCCGGCCGGCGGCTCCTAACACCGGGCGGGCGGCTGCGGCGGCACTTCCGGGTTGGCCTTCT
CCATGTTGGTCTCGGGAACGTGAAGGGGCGGGGCCTCGAGGTCAGGGGTCAGCACCTCCCTGGTTGCCGTGGCGACTGGAGGCGCGCGACGCGGGC
GCAATGGCGGGGACCCGGGGCTTGATGCTGCTTGGGCCTGGCCCAGTGGCGGGTCCTAGGGACGTGGGTACCTGCAGAGGCCGGCAGATGGAGATTC
AGAAACATAAGGACAACAAGAAACTTCCCCAAGGTATCATTATAGTCTTTAGACTTCAGACACACACCACACCTCAAATATATACACAACTGAAAGGA
AAATTAAGGAAGTTTTTCAAAGAACCCTATTCCGAGTAAGAAGTGTGTTGCATGAATTTCTAAGAGCCAGAAAATGCATGACACAGGAGAAGATGTAC
CCTCATCTGTTCAGTGAGAGATGTGCAAATCAACATCAACACAGAACTGCTGAAGAAAAAAAAATATGTCTCTGAAAAGCAACTTATTCACTGGAGATG

TGAGGAGCCATCCGCACATCACAATTCTATAGACATCAAACGCATGAAGCATTTCGGATCTGCTTTAAGACTGAGGCAGACTTTCCATCTGGACACAGC
CGACCATCCATGTGTCATTACAATGAATCCAGCACTTCCCTGGAAGCTGGAAGGGTCAAATTCAACTTCAACTCTGCCACTTCCTGCTTAACTTGCTGT
TTAACCTTGGGCAAGCTGCCATCCCACACGAGGCCGAAGGTGTCGGTTTTTAAAATACAGAAATTTGGTTCCATGAGGGATTATCAATATACTGCTGGT
GGGCCAAATTCCATCTGCTACTTGCCTTTGTAAATAAAGTTTTATTGAAACACAAACATGCCCATTAGTTTACTCTTGTCTGTGACTACAAGGGCAAAGT
TGAATAGTTGTTACAGAGACCATCAGGCTCACAAAGCCTAGGTTATTTACTATCTGCCCATTTACAGAGTAAGTTTGCCAGCCCTGGACTGGATAATTTT
TTGAGTTCTTTCTAATTCTAGAATTCTTTATATATTTTATGAAACCCCTAATGTTTATATATTAATTATTTCAATACCCGTGTCTTTACTGAGTCCCTCCTATG
AAGCATACATCAAGACATATGTGTGATACATTAACTAAACTTTTAACAGTATCTTAAATAGGATTTCAATGACTAACAGCTCTTGGAAATATCCAAGGTAA
CATTTGGGGGCAAATGTCTTATGATTTGATTATCATTGGTAAATAGAGCATATCTTTTTTTCCACTAACAATGTTTAAGAAGTTAAAAAAAAATCAAGAAA
ACCCCGTATGGCACTAGAGACTTATTGCATTAAGACATCATCTTAACAGATTGTTTAGTTTTGGTAATATAATTTGACATCTAAACATAAGCTGAGAGGGA
TCTCCCTCAAAACCTTTGATAATATGATTCATTGTCAAATGTTTGTTTTGCCTATCTGGCACTCATTAATTAATTCTTTTTAATATAGCTTGGATTTACTGAT
TTTGAAAAAGAGAATTATTCTTATTGATGTTGCTAAAATATCCAGGTGGCTTGAATTTCACCAACTAGATAGTTTAATCTAGCACATTTGTGTAGCCCTAA
TCAAATCTTTACTAACAGATGTGAGAAGCCCAGTACTGGAGCTAATGTTTGTGTCTCAATGATAATTAAATTAGCTTAATAAGGAAAATAAATGAACAAT
AATCACCAAAATTTTTCCCTAAACCTGTGTTATAGTCTTAAAAACATTTTTTCCCCTATGGAGGCTTTTGGTAATAAAACACAAATTGAGGGATTTTAATT
AAAAGAAATTTTTTTTTCAAGAACTGGTCATGTACTAAATTGCTTATAATCAAATAATCAAGTAGCATTTCTTTTATTATAAGAGCAAAACCTATAATTAA
GCCAAAATTCAAATTTGTTTTTAAAGGTTGAATAATCTTTTGTTTTGGCCAATAGTATGTGGATTTTTTTTTTAAGTATTATCTCTTTGTAGTATTTTCTAAT
GGATAGGAAATGCCTTTCTCAGGTTGAATGATTATTTCCACATAGAATTAGTTCCTGCTTACAACAGTGCTCCACTAATGTAATGGCTCGGTGAATTGGC
TTTATAATTCATTAATCATAGCTCACATTGAGCTTTTGGCTCCAGGTGACAGTGCCCACCACAGGCCAAATAACTTCTCATTTAAAGGCAATTTGTACAA
ATAGGGAAGGTGAGAATTCTCACAGTCCCAGAGGGATTGAAGAGTTTTAAAAAACCAAGATAAGGCATTTTATGGAAGACAGGTTGATGGAAGAAAA
GTGGCCATTGGAGGATGAGGCAGATGGCTTTCCAGGGAGGCCCTGGAGCTGCCACCATGGGGAAGGCCATGTTGCAGCTGCTGATCAGAGCACATTG
GACGGTGTTTCCGTGTGAACATGAAGACAATGCTGCCTCTGTTTCTGTGACGCTGTGCTCTGATCTTGCTGGAGGCGAAGTGGTGTCAGCTGTGCTTA
CAGGACAGTCAGTGGTGGTAGAAAAGAGGCCAGTGTGACCTGTGAAGGACTGGGCCCCTCTCACTCAACCTGAGTGCCTGCAGTTGTGGCCACCTTT
ATTTGTGTATTCTACTGTATTTATTTATTTGCCTGGCTAATTAGCTCTGTGCCACATTCCTAAGCTAGATGTGTCCCTTCATGTAAGTGAGAGGGAGACAG
AAAGAAAGACTGAGGTGGGGGGTAGGAGAAAGAGAGGAAAGCAAGCAAGAAGAAAGAGAAAGAGAGAAGAAGAAAGGAAGAGGAAAGAAAGA
GAGAAAGAGAAAGAGAAGGAAGGAAGGAGAAAGAGAAGGAAAGAAGGAGCCTTCTAATTTTATTTCATTTCTCCAATAAATGTTCCTATATTAAGACT
TTAGACAAAGATTAGAAATATGATTTGACAGAAAGTGTATGGATGTGTTGTTCTATTGCATTGTAAGTAAAGTATCCATGGCAATATACAAATAATACAGC
TCCTACCCCTATTCTCTGTTGTTACTTTCATGTGTTGTCTTAACTTTTTAAATTTCAAGATAATATTGCGCTTATTTATTTTTCCATGAGGAGGCCTAGGATT

TAGCTTGATTAGAAAAGCTGTGGAAAATGAATGTGCTAATTACTTTAAATCAAATTGAAAATAAATTTTATCCTGTTTCATGCAAAACA
>ENST00000426021.5|ENSG00000177640.15|OTTHUMG00000019127.8|OTTHUMT00000050579.1|CTA-287C20.1-003|CASC2|3232|
GACTGGAGGCGCGCGACGCGGGCGCAATGGCGGGGACCCGGGGCTTGATGCTGCTTGGGCCTGGCCCAGTGGCGGGTCCTAGGGACGTGGGTACCT
GCAGAGGCCGGCAGATGGAGATTCAGAAACATAAGGACAACAAGAAACTTCCCCAAGGTATCATTATAGTCTTTAGACTTCAGACACACACCACACCT
CAAATATATACACAACTGAAAGGAAAATTAAGGAAGTTTTTCAAAGAACCCTATTCCGAGTAAGAAGTGTGTTGCATGAATTTCTAAGAAATGTATTTC
TCACAAGTCTGGAGATGGGAAGTCAAAGACCAAAGTACCAGCAGAATGGTTGTCTAGTGAGGGCCCTCTTCCTCCTAGATGACAGTCTTTGCACTGTG
TCCTCAAGGGTAGAAGGAATGAGCCAGCTCTCTGGGGTCTCTTTTATAAAAGTGCTAATCCCAGTCAAGAGGGCTCTGCCAGGGGCCCTGCCTCCCAA
TATTATCACCTTGGTGCTAACCAATATTTTCTAACTTCCATTTGCTACTCTAATGTTAACTCTGTTATCTCAATTTTAATAATAATAATAGCTGACATTTGCT
GATGACATGCCTTACACTGCATTCATTAACATCTCAATCTCATTTAATTTGAAAGAGATAAAGCAACAGCCAATACACACATTACCACTTTTTTTCCTATC
CTGTTACAAAGGAGATGAGGTTGTGGGAGAACTCTCTTTAGGCATTGTTCACATCAGGACACTTGAGAAACAGATCCTTCATATCCTTTGGACAACTCT
TGTACTTCAGTTGGCTTTAAGCATTATCAGAGCATCCTTGACATGATGCCAGGTCCTAACACCGCTTCAGTGAAAGCTCAGAGACAAGCAAAAGATAAT
TGATCTATTTTGTGCCGCATGACATACTCGATGAGCAGCAACATATACTGTGGTGTTTGGCCTCCATAAACTGCAGAGAAATTGAAATATAGTAATAAAC
TTTATGCACGGACAAAGGGTGACCTAGGATATCACTGAACTCTTCTGCCTTCATAACCAAATGTCAAAATGCTAATAGTCTGTTCCTTGTGGAAAAATGT
TGAGTTTTCATTCGGGAGATCAGGACTTACACACTAACTCAACCTACAGTAGACACTTGCTAGAGAATAAAACACAATAATGCACGTAGAATCGTTCTG
TAAACTGTAAAGAATTTTACAAATATGAAATTTTATTATGGTTTTAGTCTTGTATCTATTAAATGTATACCTATTAGCATGCACACTTTTTTCTTCTAGCATC
AAAGGTTGCTTAAAATAACAGTGGAACATTTAAAAGCAAGAGAATTCTAATTTACCATCATTCAAATTATGTTAAATCCAACTCAACTTTATAAGCTAAG
GAACATGTGAAGGATAAATAAAAATTTGATAGTTAAGAAGATAAATATATGCAGATAAATTATGTGATGAGTCAAATTGATTATTACAGGACAATATACTG
TATTAATAAAACAGACTAAATTTAAATGTATTGTGCAATGGCAAACCTAGTGCCTTCTTGGAAAAACATGCCTTAACAAGCTCATGTGGTTGCAAGGTT
GATAATATCTTGATAATAACTTATGTTGTTCTTATTCCTAGTAATTGCTGGCCTCCTTATGACCTTCCGCCTAGTTTCAGGCAGTAGCTAGGAGAGCTAGAC
TAACTGCTCCAGAGAAGTGAGGCCAAGGCATGCAACAGAGTCACTTCTCCGCTTTTCCATTTCAAAGTAGACAATAGGGGAAGATATAACAAAAGCCA
AAATTTATGCAGTAAAGAAAAAATTGGGGAAGAATGCAATTAATGAAAGACCTACACTATACAAGGATTTCACTGGACGAAGTGAAGAATAGGGAGCT
TGTAAAGAAGATCAAGATAGTTGGACAGGTGGAATGTCTCCAAGACCTTTCCACTCGCCTCCCCACTTGTTGAGCATGGGAGCTGGGCAAATGGATGA
TAGACCATCCAGATAAATTTTGGCATCTAAAAGTAATTGTCACTTGCATCTTGTTTCTCAAGAAGGACCTTGAAAGTAAGCAAACTCCTAGAGCTCCCT
AATCCCATGCACCACATTGGCTTGGAGCAGCAACAGTAGCAGTAGAATAGCAGAGCAGGGGGACTCAAGGCCAAGACGGTCTTGTTACAGGCAACTC
CCTGAGCCTCCTTCCATCCGAGAGGTACCTGAGAACCTCTAAAAATTTCATTTCCCCCTGAAAGGTGAGAAAGTGACTGAGAGAGGGCTGATAAACTA
GGGTCCTCCAGGTCGGGATTGGGAAAATTCAGAATATACATGGATAAAGAACCAGGGACTGGCCATGATAGGACCTACACCTATTACTTCATATGACTT

GCTAATAGGAGTCAATGCTGACAGCCATAGACCTTCCTGGGCTTCTCCCTGGGGCCTCAATTTCAGTCCAGCGTTAAGAACTTAGTTTTGGTCTCAGGG
AGAAGGATAGGGAGATGGAAATTTTACCTGAGTTTAAAAACCTGAATTTATGCAGATATGACATTTTCTGGATACATTTTGACAAGAGTAGAACTAGGG
GAATCAAAATAGAGATTATGTTGAGTAACAGGGAAATAGAGTTACAGTTCTTGCCTATCATCTGAGTATATGGACTGAAATACTGTACCTGCTACATACA
TTGTAAGCAAAGTGAGGTCATGGCCAGAGCCCAAGGGTTTGAGGAGCCTTCTATGACGTGATGAGATGGTCTTGGGTCCCCTATATGATTAGCTTGTGG
TGGTACATTAGGACTAAATATTCCAAATGCATTAACATAAAGCTGAAGTGATGTCCTTAGTGAACTTGGTCTTAAAAATTAAGAGCATTTTGGTTGGGTG
ACCTACCAACAACATACAGGGAAGAGATACAGGATCTTGGTTGATTCTCACTGCAGTAGACATTGATTTTGTGGTGTAGCAGCTTGTAAAATTAATGCA
AATTTGAGATGAACTAATAAAAATATAGTTCATGAAGGTGCCCCACTACTTGCCCTACCTCTCCTCTCCTGTTACAATAGATCACCCATCTTTGTTCCCAT
CTGAGGACTCAGGTAATGGACTTCATCTCTTCTTGTCTTCTCAAGGATTTTGCATCTGCCATTATCCTTTCTCCCTCCTGTATCCTCAGTTGTTTTCTTCTC
TACTGGATCAATGTCATCAACATACAAATATTATCTAGTATTTCTCATATT
>ENST00000454781.5|ENSG00000177640.15|OTTHUMG00000019127.8|OTTHUMT00000050578.1|CTA-287C20.1-002|CASC2|2216|
GCGCGACGCGGGCGCAATGGCGGGGACCCGGGGCTTGATGCTGCTTGGGCCTGGCCCAGTGGCGGGTCCTAGGGACGTGGGTACCTGCAGAGGCCG
GCAGATGGAGATTCAGAAACATAAGGACAACAAGAAACTTCCCCAAGGTATCATTATAGTCTTTAGACTTCAGACACACACCACACCTCAAATATATAC
ACAACTGAAAGGAAAATTAAGGAAGTTTTTCAAAGAACCCTATTCCGAGTAAGAAGTGTGTTGCATGAATTTCTAAGGTCAAAGATGTTGATTCATTCA
TAGTGTGAGGCCCAGAAATCGGCATTTTTAAACAAGTACACTGGACATTTCTAAAGCTGGCTAAAGTTTGAGAGACACTATCCAAGGGAAATTTCTCC
GTAACTCTTTTTCTCAGAAGAAAAGTAAACTAAAAGTAAAAATAAGCAGTACCACTAAGTTTCAAGTGATCTTCTCCTAAAAGAAGAAAAGGCCTATA
TTCAGGCACAAGAAAAGTATATTCTCTTTTATCCATGCAGAAAACAGCTAGTGGTTGAAAATCAACACTTCAGACCTCTTGTCTACATAAAGCCAACTG
CCCCTTTGTTTTACTGATGTCTTCAATGATTTTACTTAGATTTTTCTACGGCATTTACTGAATTAGCTAAGCAGACACAGATCCAGTCCTTCATGAATATGG
TTTGGAGACTCTCTACTGAATCAGCACTCTTAGCTTCACCGACATAGTCTCTTGGTAGACCAGAAGGTGTATCTTATCTGGAAAGACTGCTATATCTACA
CCCACAAAAAGCATTATTTATCTGAACACTAAATTCTGACTTCCTTGCCATTTTAGGGAGCCACTTTAAAACATTTTCAATATTTGCTATTTTGTAGCAGC
AAATCCTTCAGAAGGAGGTCAAGTGATGGCCTTTATCTGTTCCCAGTGTCTTGCCCTTAGGTCCTCAGGGCACCCCCTGGACAGCAGCCTTAGGAACC
AGGTACAAAGCACAAGCACTGCTGGTCCGCATGGTAAGGAATCACTTCCAGACTGGGCCTTCCCATGGGCTTCCCTGAGCTTCCTGGACTCAAATCAT
AGTCCTTCTTTTCCTCTCCCCTTTGGACTTGATAAACGCAGTCCCATTCATTTAATCTCCAACTTAGAAATCCGGAGAATGTGAGCCCTGGGATTTACTTT
CAGACACCCTCGTCGATAACCTTGGCCACTTAGGTTGCTGCAGCCTGTCCCCAGGGAGACAGTTAGAATGGAACAATGACACAAACAACACCAACAA
TAACAACAGCAGCAGAAGCAGAAGCAACAATAGTAGAACGAGAAATAATTTATAACATGCTTACTACATGCCAGGCACTAAGTGCTTACCCATATTAAC
TCAGTTAATTCTTATAACAACCAGATGAGATAGATTTTCTTATTATCCCGATTTTACAAATGAGCAAACAGAGGCACAAGCAGGTTAAGAAACCTCCCCA
AGGTTAAACAGTGAGAAACAGCAAAGATTTGAACAGTGAGAAACAGCAAAGATTTGAAACAGCAAAGATTTGAACCTATTGTTCGGGAGTCCATGCT

GTTACTCATTTCTTTATGCTGCCATAATCTAATATAAAATTGAATAACAATAAATGCACCTTATATTTGTATAGCTATACACACTAATTTACATGATCTTCATA
AGAACTATGAAATGGCTGTGATTATCCCCATTTTTAAAATGATACTATAAAGGCATAAAATGCTGTATTTATGGTATGCAACCCTTTGGTGAACAACCCTA
TAAATATTTAATTACTTATATTGATGTTCTTCCTACGTCTAAGAAAAATGTTATTTCCATTCCCAAGATGTTAGTAGAGACGAAGATTTTTCAATTTGTCTC
CTTGATTTTGTTGGATTATAGCATTAAAGTTATTTTTACAAAGCCATCTACGGTAACCCAGTAGAAAGGGTAAGCCTGTTGGCTTAGGAAAACTGAATGG
CATTCAGTTTCTTCAAAGAAATTTGGGACAGATGTGGGTTTTTTTTCTTCTTCTTCTTCAACAAATCCAGAAGATGAAAAATGTATTTTATTTCTTGTGAA
ATGCATTTTTCTGTTCATCTCAGTATGTGGGACTGTTGAAGTTTATAAGTTTGGCAGTGTTCTAACATGGCCATCCTGTTCTCCTTTAGCAACTTATTAAAT
GTATTTTTTTAATGAAAACAGAA
>ENST00000454857.5|ENSG00000177640.15|OTTHUMG00000019127.8|OTTHUMT00000050582.1|CTA-287C20.1-006|CASC2|720|
CGGGGACCCGGGGCTTGATGCTGCTTGGGCCTGGCCCAGTGGCGGGTCCTAGGGACGTGGGTACCTGCAGAGGCCGGCAGATGGAGATTCAGAAACA
TAAGGACAACAAGAAACTTCCCCAAGGTATCATTATAGTCTTTAGACTTCAGACACACACCACACCTCAAATATATACACAACTGAAAGGAAAATTAAG
GAAGTTTTTCAAAGAACCCTATTCCGAGTAAGAAGTGTGTTGCATGAATTTCTAAGAGCCAGAAAATGCATGACACAGGAGAAGATGTACCCTCATCT
GTTCAGTGAGAGATGTGCAAATCAACATCAACACAGAACTGCTGAAGAAAAAAAATATGTGTGTGGATTATATATATACATATAAGTGTGTGTATATATAT
ATATATATATATTTACATATATAGTATGGGCTATTTCACCTTTGCAAGCTGGGAGCCATTAAGTAATGGCTACCACCACTCCCCCACCCCCTTCAGTAAAAC
ACATCCTGAACATTGGGCCCATGTTTTCAAGAAGAATGTGCCCCACTGCCCCTTGGAAACTCCATTTCATTTACTTGGTTCAGTTAGTTAATGACGGTCC
ATTTTGACTCAAATATCCAGTTCCCTTTCATCAATCATGTCTTTCTGGACTGTCAGTTTTAGGGCAAGGATTACAAAGCCCTTCCATGCAGATATCCACAG
AGAAAGACCCAGACCCACATCC
>ENST00000439517.1|ENSG00000177640.15|OTTHUMG00000019127.8|OTTHUMT00000050580.1|CTA-287C20.1-004|CASC2|455|
AAAGGAAAATTAAGGAAGTTTTTCAAAGAACCCTATTCCGAGTAAGAAGTGTGTTGCATGAATTTCTAAGGTCTCTGAAAAGCAACTTATTCACTGGA
GATGTGAGGAGCCATCCGCACATCACAATTCTATAGACATCAAACGCATGAAGCATTTCGGATCTGCTTTAAGACTGAGGCAGACTTTCCATCTGGACA
CAGCCGACCATCCATGTGTCATTACAATGAATCCAGCACTTCCCTGGAAGCTGGAAGGGTCAAATTCAACTTCAACTCTGCCACTTCCTGCTTAACTTG
CTGTTTAACCTTGGGCAAGCTGCCATCCCACACGAGGCCGAAGGTGTCGGTTTTTAAAATACAGAAATTTGGTTCCATGAGGGATTATCAATATACTGCT
GGTGGGCCAAATTCCATCTGCTACTTGCCTTTGTAAATAAAGTTTTATTGAAACACAAA
>ENST00000614455.4|ENSG00000177640.15|OTTHUMG00000019127.8|OTTHUMT00000476179.1|CTA-287C20.1-008|CASC2|489|
AATATTATATCCTGAACTCATTTCAACTGCGTGTGTAAATCACCACCTGTATCTGCAGAGAAAGTGTTTTAACCCAGCCTGCTGCCCCCAACCAACATTA
TTTCCAGCTTTGAACTTATCCTGGGATTGCTTTTGCTAGCCAGCTACTCTGCAGCTAAAGTGCTATTGTTCCCTTTAATAATAGCCTAAGCAACTGTTCCT
TTTTAATGCAAAGTCCTGGAACTTAACAAGTTGATTACAAGATAGTGACTCACTTTTTATCTCTTTCTGTGCCTCCACCCCACCTAGATCTTTCTGCTCCT

GCAAATTTTAGTGAGTGGGTTACCTAGAACACCAGTCCAATAAAGGTTGGAGTAGAATACCAGACAAGAAAAGGATGAGCTCATTTCTCCTCTATGCT
GTCAGTCTTTGAACACAGTGAGACCTATTAGCCAGAAAATGCATGACACAGGAGAAGATGTACCCTCATCTGTTCAGTGAGAGATGTGC
>ENST00000622752.1|ENSG00000177640.15|OTTHUMG00000019127.8|OTTHUMT00000476177.1|CTA-287C20.1-007|CASC2|762|
TCCCACACACTGGCTGTGAACTTGTTCCCAACAAATGAGTCTGTGGGTTGGGAATGTCATCTATCTGAGCAGATATCTGCCCATATACTATCTCAGTACC
AGCAAAACTCTGAGCCTAAAGGTGACACATGGCCAAGGGCTGCTCTGCTGTTAACCATGCACAATCGATGGCAGCAAAGGTTGAAGTGAGCATAATCT
GCAACCTTTCCATGGAAGTCTTAAACCTTTCTCACAGTGGTTCTGCCCATTGTCAGACAATTATATTGGAGAAAGGTTTTCATTTTCATAGCATGTTCAG
ATTTTGGAGGGAAATGCAAAGAGCAGTGAAGGAGGAAAGGCAAACCCTTCCTCCCAGGCTTCAACAGAGTCAACCCGGGAATCAATGAGATGACAG
ATGTTGGAGACAGATGCCTGAATATCCACTCAATAGTCAGGCTTAATGAACTAACACAGGAAGAGCAATTAGAGAATTCATTAATTATACTCTAAGTTCA
CTTTCATTGTTAATTCTAATCCTTTATTTGGGCTAATGACTTCAGCTTTTCTTATAATAATTTGTGCCCGTCAGTGAATGATAACATCTTCAAATTTCCATAA
TTGTGTAAAATTCAAACTATATGTGTAGCTCTAAATTGGACCTAAAGTAGCTTCTAAGGGTACATAAGACACTTCAAGATAACATAAATGAGGAGAAAA
ACAAAAAGCCAGAAAATGCATGACACAGGAGAAGATGTACCCTCATCTGTTCAGTGAGAGATGTGC
>SNHG10
>ENST00000500370.2|ENSG00000247092.6|OTTHUMG00000171635.1|OTTHUMT00000414545.1|RP11-1070N10.2-001|SNHG10|1531|
ATCAGGGTCACGCTTGCAGGGCAACGAGAGGCAAAACAAGAGGGAAGACGACTTTCCTCCTGTGAGCCAATGAGGCCAGCTGGACTACGCCGAGAC
AACTGGGAGAGGCGCGGGACTCGCCCGTTCCGCGGAACGCCGGGAAGGGGTCACCTCCTGATGAAGTTTCCGGTTCCGGTGTCAGCGGCGGTTGAAT
TGCCATGGCAATGCGGTGGGCGCGCGCTTGTCGTGTTGGTCTCTTGGGAGGTAGCGGGGCTAGGCCGGGCGGGTATCCGCCTCTCCCAGCTTAGGTGA
GCGTCCCCGGGCGCCTCCGGAGCGCCGCGGCCGCATGCAGTTCGTCGTGGCGGGGAGCCGGAGCCTGACCGGGGTTCCAGCGCTCGGGCCGTAGCCT
TGGCTCCTGGACTTTCCCTGGCTCCGCCGCCACGTGGGAGCTGAGGCTCTGGGGCTTCCGCCTCCGGCGCGCGATTATTTCTCTAGAACAGTTTTCATT
TTTAAAATTTGTAAAGCGCTTTTGCCTGTGTGATTTCCTCTGGGTTTTTTTTTTTTTTCTTCCTTTTTGTAGAGACGGAATTGGCGGCGGGGGCGGGGGG
TCGATGTCTCACTTTTTTGCCCAGGCTGGTCTCGAACTCCTGGCTTCAAGGGATCCTCCTGCCTCGGCCTCTTAAAGTGCTGGGATTACAGGCGTGAGC
CACCGCCCCCGGCCGCCTCTGAGTTTCCAGCCTCGTTGGCCCTCCAGCCTTTTAACCTGTTGGGCCTAGGATCAGGAAAGATTCATTGATTCCAGGTAA
ATCAGAGGAACAAGCAACATGAACAGAAATATGTAGAAAAAGCTATTATGCAGAAGCATAATTGTTGTTTCAGAAGTCCAGCATCTGGTGCACTTAAC
AATAGAGAATATATTAAACTCTTTCCAAAATAACCTGTTGTTCCTACCTGTGTTGTAAGTGTTTTGTTTGTTTTTAAGTAAATATTGGTGATTTGTTGCCAA
AAACCTAAACTGTTTGTTGCTGTCAACTTAAATAACGAACAGAGAGAGGCTCTCTAAAAGAAAATGGCATTTATTCAGGAATAGAGCACTGCAATGAG
AAAATGCATGCGATAGTTAACTGTGTGTATCCTGGGAGATAGAGGAAGACAAAGGTCTTTAAAGGAAAAATGAGGAGGGTTACATAATTGTTTTGAGAT
AATTATTTTTGACTACAAGGATCAGTAACAAAGGTGACACCAGTCCCAGGTTGGACAGGCAGGTGCTCGGCAGGCATTGCAGAAGTATTTTTTGTGTA

AGATTGAGATGGCCTTTGTGCAGGATTGTGGTTTTGGAATCTTCTGTGATCGTTTTTGCTATCAGGCATTTATGCTTGCGAACTCTTTATGGCCTTCCCTG
TGTCTATTTTGATTCCACCTTTCTCATTGCCCACTCTAAACTGTAATTCTGCTTTTATGTTGACTGAGATCAGGAGAAGAATTTGCAAACTTATATCCTGA
GACAGAACAGTAATGAGGAGTATCGTGTTCCTTACCTAATGTATAATAT
>ENST00000554169.1|ENSG00000247092.6|OTTHUMG00000171635.1|OTTHUMT00000414546.1|RP11-1070N10.2-002|SNHG10|981|
GTAGAGACGGAATTGGCGGCGGGGGCGGGGGGTCGATGTCTCACTTTTTTGCCCAGGCTGGTCTCGAACTCCTGGCTTCAAGGGATCCTCCTGCCTCG
GCCTCTTAAAGTGCTGGGATTACAGGCGTGAGCCACCGCCCCCGGCCGCCTCTGAGTTTCCAGCCTCGTTGGCCCTCCAGCCTTTTAACCTGTTGGGC
CTAGGATCAGGAAAGGTTTGTTGAATGGGGAACTAAGAAGTGAATTCGTTCGTTCGACAAACGTTTCCTGAGCAGCCGCTGGGTGCTAGGCGCAGTG
CCAGCGCGGAATGTCCAGGGAGACCTGGTGCCCAAAGCTTGGACCCATCGTGAGAAATGAGAAGCAGATACAAAGCAGTGTGGGAGTGCAGAGGAG
ACAAAGCAAGCCTCATCAGGCCCATTGCTTGCTCTGCTCTCCCTTGTACTTACCAGTGCTTGACAATATACAGTTATTTACTAGCTTGGTTATTGACTTCC
TACCCAGCACTCAGTTTTATTCACTGCTGTATCCTCAGTGCCTAGGACGATGCTTGGAACGTGATTCATTGATTCCAGGTAAATCAGAGGAACAAGCAA
CATGAACAGAAATATGTAGAAAAAGCTATTATGCAGAAGCATAATTGTTGTTTCAGAAGTCCAGCATCTGGTGCACTTAACAATAGAGAATATATTAAAC
TCTTTCCAAAATAACCTGTTGTTCCTACCTGTGTTGTAAGTGTTTTGTTTGTTTTTAAGTAAATATTGGTGATTTGTTGCCAAAAACCTAAACTGTTTGTT
GCTGTCAACTTAAATAACGAACAGAGAGAGGCTCTCTAAAAGAAAATGGCATTTATTCAGGAATAGAGCACTGCAATGAGAAAATGCATGCGATAGTT
AACTGTGTGTATCCTGGGAGATAGAGGAAGACAAAGGTCTTTAAAGGAAAAATGAGGAGGGTTACATAATTGTTTTGAGATAATTATTTTTGA
>ENST00000555866.5|ENSG00000247092.6|OTTHUMG00000171635.1|OTTHUMT00000414547.1|RP11-1070N10.2-003|SNHG10|221|
GCGCGCTTGTCGTGTTGGTCTCTTGGGAGGTAGCGGGGCTAGGCCGGGCGGGTATCCGCCTCTCCCAGCTTAGATTCATTGATTCCAGGTAAATCAGAG
GAACAAGCAACATGAACAGAAATATGTAGAAAAAGCTATTATGCAGAAGCATAATTGTTGTTTCAGAAGTCCAGCATCTGGTGCACTTAACAATAGAG
AATATATTAAACTCTTTCCAAAAT
>ENST00000553559.1|ENSG00000247092.6|OTTHUMG00000171635.1|OTTHUMT00000414548.1|RP11-1070N10.2-004|SNHG10|428|
AGCCTCATCCTACTGCCTTACTATTGGTCGTCGGCAACCGCTTTGTTAGTTAATTGGAGAGACTGGATGTCCATCAGGGTCACGCTTGCAGGGCAACGA
GAGGCAAAACAAGAGGGAAGACGACTTTCCTCCTGTGAGCCAATGAGGCCAGCTGGACTACGCCGAGACAACTGGGAGAGGCGCGGGACTCGCCC
GTTCCGCGGAACGCCGGGAAGGGGTCACCTCCTGATGAAGTTTCCGGTTCCGGTGTCAGCGGCGGTTGAATTGCCATGGCAATGCGATTCATTGATTC
CAGGTAAATCAGAGGAACAAGCAACATGAACAGAAATATGTAGAAAAAGCTATTATGCAGAAGCATAATTGTTGTTTCAGAAGTCCAGCATCTGGTGC
ACTTAACAATAGAGAATATATTAAACTCTTTCCAAAAT
>MIR17HG
>ENST00000582141.5|ENSG00000215417.12|OTTHUMG00000017195.3|OTTHUMT00000442497.1|RP11-121J7.2-002|MIR17HG|2845|

GAAGCTCTCCTCGCGGGGCGGGCCGGCCGGCCGCACCCCCGGCCTGGGGCCTCCGGTCGTAGTAAAGCGCAGGCGGGCGGGGAGGCGGGAGCAGG
AGCCCGCGGCCGGCCAGCCGAAGATGGTGGCGGCTACTCCTCCTGTCATACACGTGGACCTAACTGCACCAGTAGCTTTTCTGAGAATACTTGCTGAA
AAGGAAGTTTTCTGGAATGGGTAAGTGTATTCTGATTTTCTTGAACTTTTCTTAAAAACAAATTTTTCTTGCTATTAAAGTTGAATAAATAGGATTGGTTT
CTTAGAGAGTAAAAGTAGGTGTTTCTTTCTTTAGACAATGTACCTTTTCTGAAAAACTAACTCATTAAGTACGGATTTGCTAATTTTAAGGTAGTAAAATT
ACAGTGTAAATATTCCTGTACATTTTTGGAAACTGGCTTATGCAGTTTACGAAATATAATTTTAGACCCTCTTTTAAGTTGGGTGATAAAGTAGATATAAC
CTGAGATGATAGATTTAAACAGGATATTTACGTTCTGCTACAATTGACTGATAACACTTGAAGTGTAGTCTGAACAGTAATTTTGTTAATCATTTCAACAA
GTATTTGCTAAGTGGAAGCCAGAAGAGGAGGAAAATGTTTTGCCACGTGGATGTGAAGATTTCCTCTAAAAGGTACACATGGACTAAATTGCCTTTAAA
TGTTCCAAAATTAGTTCTCATTTATTTGCAGTCTCATTTTGTTTTGTTTTTTTTCTCTATGTGTCAATCCATTTGGGAGAGGCCAGCCATTGGAAGAGCCA
CCACTTCCAGTGCTAGTTGGATGGTTGGTTATGATTGCCTTCTGTAAAGAATTCTTAAGGCATAAATACGTGTCTAAATGGACCTCATATCTTTGAGATAA
TTAAACTAATTTTTTCTTCCCCATTAGGGATTATGCTGAATTTGTATGGTTTATAGTTGTTAGAGTTTGAGGTGTTAATTCTAATTATCTATTTCAAATTTAG
CAGGAAAAAAGAGAACATCACCTTGTAAAACTGAAGATTGTGACCAGTCAGAATAATGTCAAAGTGCTTACAGTGCAGGTAGTGATATGTGCATCTAC
TGCAGTGAAGGCACTTGTAGCATTATGGTGACAGCTGCCTCGGGAAGCCAAGTTGGGCTTTAAAGTGCAGGGCCTGCTGATGTTGAGTGCTTTTTGTT
CTAAGGTGCATCTAGTGCAGATAGTGAAGTAGATTAGCATCTACTGCCCTAAGTGCTCCTTCTGGCATAAGAAGTTATGTATTCATCCAATAATTCAAGCC
AAGCAAGTATATAGGTGTTTTAATAGTTTTTGTTTGCAGTCCTCTGTTAGTTTTGCATAGTTGCACTACAAGAAGAATGTAGTTGTGCAAATCTATGCAA
AACTGATGGTGGCCTGCTATTTCCTTCAAATGAATGATTTTTACTAATTTTGTGTACTTTTATTGTGTCGATGTAGAATCTGCCTGGTCTATCTGATGTGAC
AGCTTCTGTAGCACTAAAGTGCTTATAGTGCAGGTAGTGTTTAGTTATCTACTGCATTATGAGCACTTAAAGTACTGCTAGCTGTAGAACTCCAGCTTCG
GCCTGTCGCCCAATCAAACTGTCCTGTTACTGAACACTGTTCTATGGTTAGTTTTGCAGGTTTGCATCCAGCTGTGTGATATTCTGCTGTGCAAATCCAT
GCAAAACTGACTGTGGTAGTGAAAAGTCTGTAGAAAAGTAAGGGAAACTCAAACCCCTTTCTACACAGGTTGGGATCGGTTGCAATGCTGTGTTTCTG
TATGGTATTGCACTTGTCCCGGCCTGTTGAGTTTGGTGGGGATTGTGACCAGAAGATTTTGAAAATTAAATATTACTGAAGATTTCGACTTCCACTGTTA
AATGTACAAGATACATGAAATATTAAAGAAAATGTGTAACTTTTTGTGTAAATACATCTTGTCTTGTTTTCATTCAAAAACATTTCACTTTTGGGGTTGCG
TGTCAGATTTGGCAGTATAAATTCTGGCTATATTTTTTGTTGTTAGATTTATTTGGCTGTTAAGTATTGCGATATGACTAAACATACTGTATACCTGATGATC
ATCTGTAAAGTTAGAGTATATCTTTTTGCTTTCTTTGGAGTTAGTGTTATTCCAGGATATTTTACTTAATCTAAAAGTTAATTTATGTTGCTCATATATTACTC
AAGTATTTAAATTTAGAGAGAATGCCGCTCTGTTTAAAGCAATGTGTAAAGATGAGTTTTTTTAAGCATGGAATTTAGGGTTGGGGTACAATTTGTTTCT
ATTAAGCAAGTACCAGTTTACCAATACATGAGTAACTGAAGTGTAACTGTTAAATGCTTGTATACTAGTTTTTCTTTCTGATTGTCAGTGATTTATAAGCT
ATAAATGACCAAGGTCCTCAGACTGCTTTTAGCATCTGCAACTTAAAAAAAATGGGAGTTAGAAAAAGAACAAATGCTAAATAGAGTAACAGTTAAATG
TATGTGTACACTCTTCCCAAATGCCAAGAGTGCAGCGGTGGGGTGAGATTCAGATATTCATTTATTTCTAAGTCTGTAGTTAACATTTATGTTCCCTACTC

CCTACGTAAGCCAGACTTTGGCAACAGTGATAGTTGATTCCAGGCTTATTTGACTTAAAGTCACTGAAGTGGAAACTAAGAAGTGGCAGTTAGTGTTTT
ACCCAGCATTTCTGCCTTCTCTCTTTTCTTCATGTGTTTTTGTCTCTAGCCTATGTGTATTTGTGTAGAATAATGTGGGATACCTGAATAATAGATTTAAAA
GGACCAAGTGGTAAAATTGGGCCCAAGCTGAAGTACAG
>ENST00000400282.6|ENSG00000215417.12|OTTHUMG00000017195.3|OTTHUMT00000045448.3|RP11-121J7.2-001|MIR17HG|931|
GAAGCTCTCCTCGCGGGGCGGGCCGGCCGGCCGCACCCCCGGCCTGGGGCCTCCGGTCGTAGTAAAGCGCAGGCGGGCGGGGAGGCGGGAGCAGG
AGCCCGCGGCCGGCCAGCCGAAGATGGTGGCGGCTACTCCTCCTGTCATACACGTGGACCTAACTGCACCAGTAGCTTTTCTGAGAATACTTGCTGAA
AAGGAAGTTTTCTGGAATGGTATTTGCTAAGTGGAAGCCAGAAGAGGAGGAAAATGTTTTGCCACGTGGATGTGAAGATTTCCTCTAAAAGGCAGAC
CTGTCTAACTACAAGCCAGACTTGGGTTTTCTCCTGTAGTTTGAAGACACACTGACTCCTGACAAAATGCAGCCTGCAACTTCCTGGAGAACAACTCA
GTGTCACATTAAAGTTTATTATGTATTTAATGATACACTGTTTAATTGACAGTTTTGCATAGTTTGTCTAACTTTAGAGAATTAAGAGCCTCTCAACTGAG
CAGTAAAGGTAAGGAGAGCTCAATCTGCACAGAGCCAGTTTTTAGTGTTTGATGGAAATAAGATCATCATGCCCACTTGAGACTTCAGATTATTCTTTA
GCTTAGTGGTTGTATGAGTTACATCTTATTAAAGTCGAAATTAATGTAGTTTTCTGCCTTGATAACATTTCATATGTGGTATTAGTTTTAAAGGGTCATTAG
GAAAATGCACATATTCCATGAATTTTAAGACCCATAGAAAAGTTGAAGAATGCTTAATTTTCTTATCCAGTAATGTAAACACAGAGACAGAACATTGAG
ATGTGCCTAGTTCTGTATTTACAGTTTGGTCTGGCTGTTTGAGTTCTAGCGCATTTAATGTTAATAAATAAAATACTGCATTTTAAAGCTGTTAAGAAATT
GTCCAGAACGAGAATATTGAAATAAAAACTTCAAGGTTATT
>ENST00000581816.1|ENSG00000215417.12|OTTHUMG00000017195.3|OTTHUMT00000442498.1|RP11-121J7.2-003|MIR17HG|2032|
TGGCGGCTACTCCTCCTGTCATACACGTGGACCTAACTGCACCAGTAGCTTTTCTGAGAATACTTGCTGAAAAGGAAGTTTTCTGGAATGGTATTTGCT
AAGTGGAAGCCAGAAGAGGAGGAAAATGTTTTGCCACGTGGATGTGAAGATTTCCTCTAAAAGGTACACATGGACTAAATTGCCTTTAAATGTTCCAA
AATTAGTTCTCATTTATTTGCAGTCTCATTTTGTTTTGTTTTTTTTCTCTATGTGTCAATCCATTTGGGAGAGGCCAGCCATTGGAAGAGCCACCACTTCC
AGTGCTAGTTGGATGGTTGGTTATGATTGCCTTCTGTAAAGAATTCTTAAGGCATAAATACGTGTCTAAATGGACCTCATATCTTTGAGATAATTAAACTA
ATTTTTTCTTCCCCATTAGGGATTATGCTGAATTTGTATGGTTTATAGTTGTTAGAGTTTGAGGTGTTAATTCTAATTATCTATTTCAAATTTAGCAGGAAA
AAAGAGAACATCACCTTGTAAAACTGAAGATTGTGACCAGTCAGAATAATGTCAAAGTGCTTACAGTGCAGGTAGTGATATGTGCATCTACTGCAGTG
AAGGCACTTGTAGCATTATGGTGACAGCTGCCTCGGGAAGCCAAGTTGGGCTTTAAAGTGCAGGGCCTGCTGATGTTGAGTGCTTTTTGTTCTAAGGT
GCATCTAGTGCAGATAGTGAAGTAGATTAGCATCTACTGCCCTAAGTGCTCCTTCTGGCATAAGAAGTTATGTATTCATCCAATAATTCAAGCCAAGCAA
GTATATAGGTGTTTTAATAGTTTTTGTTTGCAGTCCTCTGTTAGTTTTGCATAGTTGCACTACAAGAAGAATGTAGTTGTGCAAATCTATGCAAAACTGAT
GGTGGCCTGCTATTTCCTTCAAATGAATGATTTTTACTAATTTTGTGTACTTTTATTGTGTCGATGTAGAATCTGCCTGGTCTATCTGATGTGACAGCTTCT
GTAGCACTAAAGTGCTTATAGTGCAGGTAGTGTTTAGTTATCTACTGCATTATGAGCACTTAAAGTACTGCTAGCTGTAGAACTCCAGCTTCGGCCTGTC

GCCCAATCAAACTGTCCTGTTACTGAACACTGTTCTATGGTTAGTTTTGCAGGTTTGCATCCAGCTGTGTGATATTCTGCTGTGCAAATCCATGCAAAAC
TGACTGTGGTAGTGAAAAGTCTGTAGAAAAGTAAGGGAAACTCAAACCCCTTTCTACACAGGTTGGGATCGGTTGCAATGCTGTGTTTCTGTATGGTAT
TGCACTTGTCCCGGCCTGTTGAGTTTGGTGGGGATTGTGACCAGAAGATTTTGAAAATTAAATATTACTGAAGATTTCGACTTCCACTGTTAAATGTACA
AGATACATGAAATATTAAAGAAAATGTGTAACTTTTTGTGTAAATACATCTTGTCTTGTTTTCATTCAAAAACATTTCACTTTTGGGGTTGCGTGTCAGAT
TTGGCAGTATAAATTCTGGCTATATTTTTTGTTGTTAGATTTATTTGGCTGTTAAGTATTGCGATATGACTAAACATACTGTATACCTGATGATCATCTGTAA
AGTTAGAGTATATCTTTTTGCTTTCTTTGGAGTTAGTGTTATTCCAGGATATTTTACTTAATCTAAAAGTTAATTTATGTTGCTCATATATTACTCAAGTATTT
AAATTTAGAGAGAATGCCGCTCTGTTTAAAGCAATGTGTAAAGATGAGTTTTTTTAAGCATGGAATTTAGGGTTGGGGTACAATTTGTTTCTATTAAGCA
AGTACCAGTTTACCAATACATGAGTAACTGAAGTGTAACTGTTAAATGCTTGTATACTAGTTTTTCTTTCTGATTGTCAGTGATTTATAAGCTATAAATGA
CCAAGGTCCTCAGACTGCTTTTAGCATCTGCAACTTAAAAAAATGGGAGTTAGAAAAAGAACAAATGCTAAATAGAGTAACAGTTAAATGTATGTGTA
CACTCTTCCCAAATGCCAAGAGTGCA

>PVT1
>ENST00000504719.6|ENSG00000249859.9|OTTHUMG00000164871.1|OTTHUMT00000380693.1|CTD-2267H22.1-005|PVT1|754|
CTCCGGGCAGAGCGCGTGTGGCGGCCGAGCACATGGGCCCGCGGGCCGGGCGGGCTCGGGGCGGCCGGGACGAGGAGGGGCGACGACGAGCTGCG
AGCAAAGATGTGCCCCGGGACCCCCGGCACCTTCCAGTGGATTTCCTTGCGGAAAGGATGTTGGCGGTCCCTGTGACCTGTGGAGACACGGCCAGAT
CTGCCCTCCAGACCATAGCTGTCTGCAGTGCAGGAAGCCAACTATTAAGGGGAAACAAAAGTGTTCTTAGGAGTCCTGCTGTCACTGTGGATTGAGCC
GGTGAAGCCCTGAGGGATTTCATCGCTGAGGTGGATGGAGAAGCAGCTGGGGGCCTTGGCTGTAAGGACCCCTAAGGGCCTGATCTTTTGGCCAGAA
GGAGATTAAAAAGATGCCCCTCAAGATGGCTGTGCCTGTCAGCTGCATGGAGCTTCGTTCAAGTATTTTCTGAGCCTGATGGATTTACAGTGATCTTCA
GTGGTCTGGGGAATAACGCTGGTGGAACCATGCACTGGAATGACACACGCCCGGCACATTTCAGGATACTAAAAGTGGTTTTAAGGGAGGCTGTGGCT
GAATGCCTCATGGATTCTTACAGCTTGGATGTCCATGGGGGACGAAGGACTGCAGCTGGCTGAGAGGGTTGAGATCTCTGTTTACTTAGATCTCTGCCA
ACTTCCTTTGGGTCTCCCTATGGAATGTAAGACCCCGACTCTTCCTGGTGAAGCATCTGATGCACGTTCCA
>ENST00000524165.5|ENSG00000249859.9|OTTHUMG00000164871.1|OTTHUMT00000380694.1|CTD-2267H22.1-001|PVT1|458|
CCGAGCACATGGGCCCGCGGGCCGGGCGGGCTCGGGGCGGCCGGGACGAGGAGGGGCGACGACGAGCTGCGAGCAAAGATGTGCCCCGGGACCCC
CGGCACCTTCCAGTGGATTTCCTTGCGGAAAGGATGTTGGCGGTCCCTGTGACCTGTGGAGACACGGCCAGATCTGCCCTCCAGATTTATTAGGAGAA
AAACCTTCCCGGAAGCTGCAGAAGGACAAATACAGAATCCGTGTCTGGGAGAAACCTCGTGGCCTGGTCTCCATTATTTGAGATGAGTTACATCTTGG
AGGACCATAGCTGTCTGCAGTGCAGGAAGCCAACTATTAAGGGGAAACAAAAGTGTTCTTAGGAGTCCTGCTGTCACTGTGGATTGAGCCGGTGAAG

CCCTGAGGGATTTCATCGCTGAGGTGGATGGAGAAGCAGCTGGGGGCCTTGGCTGTAAGGACCCCTAAGG
>ENST00000523328.5|ENSG00000249859.9|OTTHUMG00000164871.1|OTTHUMT00000380695.1|CTD-2267H22.1-003|PVT1|343|
CCGAGCACATGGGCCCGCGGGCCGGGCGGGCTCGGGGCGGCCGGGACGAGGAGGGGCGACGACGAGCTGCGAGCAAAGATGTGCCCCGGGACCCC
CGGCACCTTCCAGTGGATTTCCTTGCGGAAAGGATGTTGGCGGTCCCTGTGACCTGTGGAGACACGGCCAGATCTGCCCTCCAGACCATAGCTGTCTG
CAGTGCAGGAAGCCAACTATTAAGGGGAAACAAAAGTGTTCTTAGGAGTCCTGCTGTCACTGTGGATTGAGCCGGTGAAGCCCTGAGGGATTTCATC
GCTGAGGTGGATGGAGAAGCAGCTGGGGGCCTTGGCTGTAAGGACCCCTAAGG
>ENST00000521951.1|ENSG00000249859.9|OTTHUMG00000164871.1|OTTHUMT00000380696.1|CTD-2267H22.1-002|PVT1|1535|
CCGAGCACATGGGCCCGCGGGCCGGGCGGGCTCGGGGCGGCCGGGACGAGGAGGGGCGACGACGAGCTGCGAGCAAAGATGTGCCCCGGGACCCC
CGGCACCTTCCAGTGGATTTCCTTGCGGAAAGGATGTTGGCGGTCCCTGTGACCTGTGGAGACACGGCCAGATCTGCCCTCCAGCCTGATCTTTTGGC
CAGAAGGAGATTAAAAAGATGCCCCTCAAGATGGCTGTGCCTGTCAGCTGCATGGAGCTTCGTTCAAGTATTTTCTGAGCCTGATGGATTTACAGTGAT
CTTCAGTGGTCTGGGGAATAACGCTGGTGGAACCATGCACTGGAATGACACACGCCCGGCACATTTCAGGATACTAAAAGTGGTTTTAAGGGAGGCTG
TGGCTGAATGCCTCATGGATTCTTACAGCTTGGATGTCCATGGGGGACGAAGGACTGCAGCTGGCTGAGAGGGTTGAGATCTCTGTTTACTTAGATCTC
TGCCAACTTCCTTTGGGTCTCCCTATGGAATGTAAGACCCCGACTCTTCCTGGTGAAGCATCTGATGCACGTTCCATCCGGCGCTCAGCTGGGCTTGAG
CTGACCATACTCCCTGGAGCCTTCTCCCGAGGTGCGCGGGTGACCTTGGCACATACAGCCATCATGATGGTACTTTAAGTGGAGGCTGAATCATCTCCC
CTTTGAGCTGCTTGGCACGTGGCTCCCTTGGTGTTCCCCTTTTACTGCCAGGACACTGAGATTTGGAGAGGTAAGTGGCTTACCTGAGGCCATGTGCTA
ACAGAGAAGATGAAGAGATGATTGAAACAGGCCTAAGACCAGACCTAAGGGTCTGTACATTTTCCACATACTTTCCATATCTTTAGAGGCCTGACCAA
AGCAGATCTTTTCCTTTCTTCTAGGTAAGTCCAAAGGCACCTGCCTGCTGGGCCCACTGTTTTCTAACTTTCCTAACTTTCTGATCCCTTGGAGGTGATA
ATCAAATATTCTAGTCTGAGGCATTGGGATACATGGTGCTAGGTTCTGAGACTCTGCGTCAGGCCTGAACCCTGCATTTTGTGGAGGTGGGTGGGAGAA
TGTTCCCCTGGGGAACATGCCTAGACACGGGGGACAACAGTTGCCCTCATGGGGAGGTACCTGTTTACTCGCTGTTATGGGACCGCTTTCACAAAACC
ACTGCAGGTGAGTGAGTTCCTGCTGAATATCAGGCCTGGTGTCTCTAGACTCATTATTTCCCCCACCCAACCCCTATGTTAGTTCATCTCGAGCCACATT
TTTATTGCCATAATCCAGGCCTGGACAGGCCAAGATCTTTTAACAATTTTAATTACTGAAAATAATAACTGCATTTTTTTTAAAGCCCAACTTTTGGTAGA
GTCAGCCCAAAATACAGTCTTTGTGTTGCCATCTGGGAACTGGATTTGGAATTGTTCTTCCATGAGACTGCAGAGCAGAACGGCAGGGCCAGAGGTCC
CACGAGCTGGTCAGACCCGGTTCTGCTCCTTGCTGGCTGAGTGACCTTGGGCATT
>ENST00000517525.1|ENSG00000249859.9|OTTHUMG00000164871.1|OTTHUMT00000380697.1|CTD-2267H22.1-006|PVT1|392|
AGCACATGGGCCCGCGGGCCGGGCGGGCTCGGGGCGGCCGGGACGAGGAGGGGCGACGACGAGCTGCGAGCAAAGATGTGCCCCGGGACCCCCGG
CACCTTCCAGTGGATTTCCTTGCGGAAAGGATGTTGGCGGTCCCTGTGACCTGTGGAGACACGGCCAGATCTGCCCTCCAGATTTATTAGGAGAAAAA

CCTTCCCGGAAGCTGCAGAAGGACAAATACAGAATCCGTGTCTGGGAGAAACCTCGTGGCCTGGTCTCCATTATTTGAGATGAGTTACATCTTGGAGC
TGACCATACTCCCTGGAGCCTTCTCCCGAGGTGCGCGGGTGACCTTGGCACATACAGCCATCATGATGGTACTTTAAGTGGAGGCTGAATCATCTCCCC
TT
>ENST00000523427.1|ENSG00000249859.9|OTTHUMG00000164871.1|OTTHUMT00000380698.1|CTD-2267H22.1-011|PVT1|938|
GGGCCGGGCGGGCTCGGGGCGGCCGGGACGAGGAGGGGCGACGACGAGCTGCGAGCAAAGATGTGCCCCGGGACCCCCGGCACCTTCCAGTGGAT
TTCCTTGCGGAAAGGATGTTGGCGGTCCCTGTGACCTGTGGAGACACGGCCAGATCTGCCCTCCAGTGGGAAGTGAGTGATGACCCTGAAGTGAGGA
CTCATCTCTAGATCTCCAAGGGCTGCAGCTCAGCCAGCACTTTACAAGGGTGATCTGGAGCCAAACTGGCCTGTTGGCTGACCATAGGTGACTCTGGG
TAGCCCATACCCAGGCTCAGCAGCAGTTGGGGAGCTGCCTCGATTTCTGGTTACAGAATTCCTGGAACTGAGTCACTGCAGTAATTGCTGTGATGAATT
GTGTTTACTTTGTGTGGGATTCCAAACTGTAGCAGCAGTGACTACAGCTGGAAGACAGCATGATCAGCAGCTTCCAAGGCAGAGCCTGGCGTCAGAA
AGCTGCATTGCGCTAATGCTGAAGCCTGTGGGAGCCTGTTGGAGAGACACTTGGATGTTTAGCGAGCTGGTGACTCTCCTTGTCATGAGCCTGATCTTT
TGGCCAGAAGGAGATTAAAAAGATGCCCCTCAAGATGGCTGTGCCTGTCAGCTGCATGGAGCTTCGTTCAAGTATTTTCTGAGCCTGATGGATTTACAG
TGATCTTCAGTGGTCTGGGGAATAACGCTGGTGGAACCATGCACTGGAATGACACACGCCCGGCACATTTCAGGATACTAAAAGTGGTTTTAAGGGAG
GCTGTGGCTGAATGCCTCATGGATTCTTACAGCTTGGATGTCCATGGGGGACGAAGGACTGCAGCTGGCTGAGAGGGTTGAGATCTCTGTTTACTTAGA
TCTCTGCCAACTTCCTTTGGGTCTCCCTATGGAATGTAAGACCCCGACTCTTCCTGG
>ENST00000517790.1|ENSG00000249859.9|OTTHUMG00000164871.1|OTTHUMT00000380699.1|CTD-2267H22.1-007|PVT1|599|
ATTTTGCATACTGGCAGCGACAAGTTGAGACTTGTTCAACTTGACACAGTCCTGTGGTCATAGCGAATCTTTCTAAAGCTCTGATCAGTCAAGAAGGG
GGTTGTATCAATCCTCAGAACCCTGAGTGGAACTTTCTACAGGATTTATTAGGAGAAAAACCTTCCCGGAAGCTGCAGAAGGACAAATACAGAATCCG
TGTCTGGGAGAAACCTCGTGGCCTGGTCTCCATTATTTGAGATGAGTTACATCTTGGAGGCCTGATCTTTTGGCCAGAAGGAGATTAAAAAGATGCCCC
TCAAGATGGCTGTGCCTGTCAGCTGCATGGAGCTTCGTTCAAGTATTTTCTGAGCCTGATGGATTTACAGTGATCTTCAGTGGTCTGGGGAATAACGCT
GGTGGAACCATGCACTGGAATGACACACGCCCGGCACATTTCAGGATACTAAAAGTGGTTTTAAGGGAGGCTGTGGCTGAATGCCTCATGGATTCTTA
CAGCTTGGATGTCCATGGGGGACGAAGGACTGCAGCTGGCTGAGAGGGTTGAGATCTCTGTTTACTTAGATCTCTGCCAACTTCCTTTGGGTCTCCCTA
TGGAATGT
>ENST00000522963.5|ENSG00000249859.9|OTTHUMG00000164871.1|OTTHUMT00000380700.1|CTD-2267H22.1-008|PVT1|568|
ATAGCGAATCTTTCTAAAGCTCTGATCAGTCAAGAAGGGGGTTGTATCAATCCTCAGAACCCTGAGTGGAACTTTCTACAGGATTTATTAGGAGAAAAA
CCTTCCCGGAAGCTGCAGAAGGACAAATACAGAATCCGTGTCTGGGAGAAACCTCGTGGCCTGGTCTCCATTATTTGAGATGAGTTACATCTTGGAGG
ACCATAGCTGTCTGCAGTGCAGGAAGCCAACTATTAAGGGGAAACAAAAGTGTTCTTAGGAGTCCTGCTGTCACTGTGGATTGAGCCGGTGAAGCCCT

GAGGGATTTCATCGCTGAGGTGGATGGAGAAGCAGCTGGGGGCCTTGGCTGTAAGGACCCCTAAGGGCCTGATCTTTTGGCCAGAAGGAGATTAAAA
AGATGCCCCTCAAGATGGCTGTGCCTGTCAGCTGCATGGAGCTTCGTTCAAGTATTTTCTGAGCCTGATGGATTTACAGTGATCTTCAGTGGTCTGGGG
AATAACGCTGGTGGAACCATGCACTGGAATGACACACGCCCGGCACATTTCAGGATACTAAAAGTGGTTTTAAGGGA
>ENST00000518528.1|ENSG00000249859.9|OTTHUMG00000164871.1|OTTHUMT00000380701.1|CTD-2267H22.1-009|PVT1|624|
ATCTTTCTAAAGCTCTGATCAGTCAAGAAGGGGGTTGTATCAATCCTCAGAACCCTGAGTGGAACTTTCTACAGGATTTATTAGGAGAAAAACCTTCCC
GGAAGCTGCAGAAGGACAAATACAGAATCCGTGTCTGGGAGAAACCTCGTGGCCTGGTCTCCATTATTTGAGATGAGTTACATCTTGGAGGTGAGGAC
GTGCCTCGTGGTCTAAAGCTTCGGCACAAGGGCCCAACTGGAATTCCACTTACGGGCCTGATCTTTTGGCCAGAAGGAGATTAAAAAGATGCCCCTCA
AGATGGCTGTGCCTGTCAGCTGCATGGAGCTTCGTTCAAGTATTTTCTGAGCCTGATGGATTTACAGTGATCTTCAGTGGTCTGGGGAATAACGCTGGT
GGAACCATGCACTGGAATGACACACGCCCGGCACATTTCAGGATACTAAAAGTGGTTTTAAGGGAGGCTGTGGCTGAATGCCTCATGGATTCTTACAG
CTTGGATGTCCATGGGGGACGAAGGACTGCAGCTGGCTGAGAGGGTTGAGATCTCTGTTTACTTAGATCTCTGCCAACTTCCTTTGGGTCTCCCTATGG
AATGTAAGACCCCGACTCTTCCTGGTGAAGCAT
>ENST00000523068.1|ENSG00000249859.9|OTTHUMG00000164871.1|OTTHUMT00000380702.1|CTD-2267H22.1-010|PVT1|844|
AGCTTCGGCACAAGGGCCCAACTGGAATTCCACTTACGGACGGAGTCTCCTTCTGTCGCCCAGGCTGGAGTGCAGTGGCGAGATCTCGGCTCACTGC
AAGCTCCGCCTCCCGGGTTCACGCCATTCTCCTGCCTCAGCCTCCCGAGTAGCTGGGACTACAGGCACCCGCCCACCACGCCCGGCTAAGTTTTTGTAT
TTTTAGTAGAGACGAGGTTTCACCATGTTAGCCAGGATGGTTTCGATCTCCTGACCTCGTGATCCGCCCGCCTTGGCCTCCCAAAGTGCTGGGATTACA
GGCGTGAGCCGCCGCGCCCAGCCCCAGGCGGAAATTATTTCAACTGGGTCCTGAGAACTGGGCACTTTTCAAGGATTGTCAGCAAGGAGCAACTTCC
CAAATATACATCTAAAAAGATGACAAACTCATGTGGAATGTGGATTTAAGGAGGAAGGCCTGATCTTTTGGCCAGAAGGAGATTAAAAAGATGCCCCT
CAAGATGGCTGTGCCTGTCAGCTGCATGGAGCTTCGTTCAAGTATTTTCTGAGCCTGATGGATTTACAGTGATCTTCAGTGGTCTGGGGAATAACGCTG
GTGGAACCATGCACTGGAATGACACACGCCCGGCACATTTCAGGATACTAAAAGTGGTTTTAAGGGAGGCTGTGGCTGAATGCCTCATGGATTCTTAC
AGCTTGGATGTCCATGGGGGACGAAGGACTGCAGCTGGCTGAGAGGGTTGAGATCTCTGTTTACTTAGATCTCTGCCAACTTCCTTTGGGTCTCCCTAT
GGAATGTAAGACCCCGACTCTTCCTGGTGAAGCATCTGATGCACGTTCCATCCGGCGC
>ENST00000521122.1|ENSG00000249859.9|OTTHUMG00000164871.1|OTTHUMT00000380703.1|CTD-2267H22.1-012|PVT1|221|
AGGGCTGGGTCGCGTGTCCTGGCAGGGCCACAGGAAGAGCATACACATCTCCTGCGTCTTCGTATTTTGCCTGATCTTTTGGCCAGAAGGAGATTAAA
AAGATGCCCCTCAAGATGGCTGTGCCTGTCAGCTGCATGGAGCTTCGTTCAAGTATTTTCTGAGCCTGATGGATTTACAGTGATCTTCAGTGGTCTGGG
GAATAACGCTGGTGGAACCATGCA
>ENST00000615442.1|ENSG00000249859.9|OTTHUMG00000164871.1|-|PVT1-223|PVT1|165|

GACCATAGCTGTCTGCAGTGCAGGAAGCCAACTATTAAGGGGAAACAAAAGTGTTCTTAGGAGTCCTGCTGTCACTGTGGATTGAGCCGGTGAAGCC
CTGAGGGATTTCATCGCTGAGGTGGATGGAGAAGCAGCTGGGGGCCTTGGCTGTAAGGACCCCTAAGG
>ENST00000513868.6|ENSG00000249859.9|OTTHUMG00000164871.1|OTTHUMT00000380704.1|CTD-2267H22.1-004|PVT1|1699|
CTCAAGATGGCTGTGCCTGTCAGCTGCATGGAGCTTCGTTCAAGTATTTTCTGAGCCTGATGGATTTACAGTGATCTTCAGTGGTCTGGGGAATAACGC
TGGTGGAACCATGCACTGGAATGACACACGCCCGGCACATTTCAGGATACTAAAAGTGGTTTTAAGGGAGGCTGTGGCTGAATGCCTCATGGATTCTT
ACAGCTTGGATGTCCATGGGGGACGAAGGACTGCAGCTGGCTGAGAGGGTTGAGATCTCTGTTTACTTAGATCTCTGCCAACTTCCTTTGGGTCTCCCT
ATGGAATGTAAGACCCCGACTCTTCCTGGTGAAGCATCTGATGCACGTTCCATCCGGCGCTCAGCTGGGCTTGAGCTGACCATACTCCCTGGAGCCTTC
TCCCGAGGTGCGCGGGTGACCTTGGCACATACAGCCATCATGATGGTACTTTAAGTGGAGGCTGAATCATCTCCCCTTTGAGCTGCTTGGCACGTGGCT
CCCTTGGTGTTCCCCTTTTACTGCCAGGACACTGAGATTTGGAGAGAGTCTCACTCTGTGGTCCAGGCTGAAGTACAGTGGCATGATCCCAGGTCACT
GCAACCCCCACCTCCCGGGTTCAAGTGATCCTCCTGCCTCAGCCTCCCGAGTAGCTGGTATTACAGGCGTGTGCCACAAAGCCTGGCTAAGTTTTGTAT
TTTTAGTAGAGACGGGGTTTCACCATGTTGGCCAGGTTGGTCTCGAACTCCTGACCTCAAGTGATCCACTCACTTTGGCCTTTCAACGTGCTGGGATTA
CAGGCGAGAGTCACCGCACCCGGACGACTCTGACATTTTTGAAGAGTCCAGAATCCTGTTACACCTGGGATTTAGGCACTTTCAATCTGAAAAAATAC
ATATCCTTTCAGCACTCTGGACGGACTTGAGAACTGTCCTTACGTGACCTAAAGCTGGAGTATTTTGAGATTGGAGAATTAAGAGCCAGTCTTGGTGCT
CTGTGTTCACCTGGTTCATCTGAGGAGCTGCATCTACCCTGCCCATGCCATAGATCCTGCCCTGTTTGCTTCTCCTGTTGCTGCTAGTGGACATGAGAAG
GACAGAATAACGGGCTCCCAGATTCACAAGCCCCACCAAGAGGATCACCCCAGGAACGCTTGGAGGCTGAGGAGTTCACTGAGGCTACTGCATCTTG
AGACTCAGGATGAAGACCCAGCTTGGGGCTGTCAAAGAGGCCTGAAGAGGCAGAACACCCCAGAGGAGCCTGGGGCCACCACCCAGCATCACTGTG
GGAAAACGGCAGCAGGAAATGTCCTCTCGCCTGCGTGCTCCACCTCGGTCCACGCCTTCCCTCCTTCTGGAAGCCTTGCCTGACCACTGGCCTGCCCC
TTCTATGGGAATCACTACTGACCTTGCAGCTTATTATAGACTTATATGTTTTTTGCATGTCTGACACCCATGACTCCACCTGGACCTTATGGCTCCACCCA
GAAGCAATTCAGCCCAACAGGAGGACAGCTTCAACCCATTACGATTTCATCTCTGCCCCAACCACTCAGCAGCAAGCACCTGTTACCTGTCCACCCCC
ACCCCTTCCCCCAAACTGCCTTTGAAAAATCCCTAACCTATGAGCTTTGAATAAGATGAGTACGAACTTCATCGCCCACGTGGCGTGGCCGGCCTCGTG
TCTATTAAATTCTTTTTCTACTA
>ENST00000520913.1|ENSG00000249859.9|OTTHUMG00000164871.1|OTTHUMT00000380705.1|CTD-2267H22.1-013|PVT1|584|
GTCAGCTGCATGGAGCTTCGTTCAAGTATTTTCTGAGCCTGATGGATTTACAGTGATCTTCAGTGGTCTGGGGAATAACGCTGGTGGAACCATGCACTG
GAATGACACACGCCCGGCACATTTCAGGATACTAAAAGTGGTTTTAAGGGAGGCTGTGGCTGAATGCCTCATGGATTCTTACAGCTTGGATGTCCATGG
GGGACGAAGGACTGCAGCTGGCTGAGAGGGTTGAGATCTCTGTTTACTTAGATCTCTGCCAACTTCCTTTGGGTCTCCCTATGGAATGTAAGACCCCGA
CTCTTCCTGGTGAAGCATCTGATGCACGTTCCATCCGGCGCTCAGCTGGGCTTGAGGACTTCGCAGGTGAGCAGTAACGGAAATCAGCCCCGTTTATTC

CTCCCAGCACCTGCCTTATCCAACTCCCCACGCTGTGGCTGAGTCCCAGCCTGCTATGGAAGCATCACTGGACTCCCATTGAACTCTGTGCAGATTCGC
TGTTCGTAGACATGGTACCTGATGGACACCAAGCTACGTACAGCTTCAAGGCCCCTTCTTTCTTTGCTAATAAAAAATAAAAAATAAAA
>ENST00000519481.5|ENSG00000249859.9|OTTHUMG00000164871.1|OTTHUMT00000380706.1|CTD-2267H22.1-014|PVT1|542|
TCTGAGCCTGATGGATTTACAGTGATCTTCAGTGGTCTGGGGAATAACGCTGGTGGAACCATGCACTGGAATGACACACGCCCGGCACATTTCAGGAT
ACTAAAAGTGGTTTTAAGGGAGGCTGTGGCTGAATGCCTCATGGATTCTTACAGCTTGGATGTCCATGGGGGACGAAGGACTGCAGCTGGCTGAGAGG
GTTGAGATCTCTGTTTACTTAGATCTCTGCCAACTTCCTTTGGGTCTCCCTATGGAATGTAAGACCCCGACTCTTCCTGGTGAAGCATCTGATGCACGTT
CCATCCGGCGCTCAGCTGGGCTTGAGAATCCTGTTACACCTGGGATTTAGGCACTTTCAATCTGAAAAAATACATATCCTTTCAGCACTCTGGACGGAC
TTGAGAACTGTCCTTACGTGACCTAAAGCTGGAGTATTTTGAGATTGGAGAATTAAGAGTGTGGGCACCCTGGCTTCAAGCTCACGAGAAACCAGGTC
GGGATTTAAACAATGTTGGGTTAAAGCAAAGTTTCATAAAGACAGAATC
>ENST00000517838.5|ENSG00000249859.9|OTTHUMG00000164871.1|OTTHUMT00000380707.1|CTD-2267H22.1-015|PVT1|518|
AGGGTTGAGATCTCTGTTTACTTAGATCTCTGCCAACTTCCTTTGGGTCTCCCTATGGAATGTAAGACCCCGACTCTTCCTGGTGAAGCATCTGATGCAC
GTTCCATCCGGCGCTCAGCTGGGCTTGAGCTGACCATACTCCCTGGAGCCTTCTCCCGAGGTGCGCGGGTGACCTTGGCACATACAGCCATCATGATGG
TACTTTAAGTGGAGGCTGAATCATCTCCCCTTTGAGCTGCTTGGCACGTGGCTCCCTTGGTGTTCCCCTTTTACTGCCAGGACACTGAGATTTGGAGAG
AATCCTGTTACACCTGGGATTTAGGCACTTTCAATCTGAAAAAATACATATCCTTTCAGCACTCTGGACGGACTTGAGAACTGTCCTTACGTGACCTAA
AGCTGGAGTATTTTGAGATTGGAGAATTAAGAGTGTGGGCACCCTGGCTTCAAGCTCACGAGAAACCAGGTCGGGATTTAAACAATGTTGGGTTAAAG
CAAAGTTTCATAAAGACAGAATC
>ENST00000612011.1|ENSG00000249859.9|OTTHUMG00000164871.1|-|PVT1-221|PVT1|169|
CTGACCATACTCCCTGGAGCCTTCTCCCGAGGTGCGCGGGTGACCTTGGCACATACAGCCATCATGATGGTACTTTAAGTGGAGGCTGAATCATCTCCC
CTTTGAGCTGCTTGGCACGTGGCTCCCTTGGTGTTCCCCTTTTACTGCCAGGACACTGAGATTTGGAGAG
>ENST00000617087.1|ENSG00000249859.9|OTTHUMG00000164871.1|-|PVT1-225|PVT1|130|
AATCCTGTTACACCTGGGATTTAGGCACTTTCAATCTGAAAAAATACATATCCTTTCAGCACTCTGGACGGACTTGAGAACTGTCCTTACGTGACCTAA
AGCTGGAGTATTTTGAGATTGGAGAATTAAG
>ENST00000512617.6|ENSG00000249859.9|OTTHUMG00000164871.1|OTTHUMT00000380708.1|CTD-2267H22.1-016|PVT1|383|
AGGCACTTTCAATCTGAAAAAATACATATCCTTTCAGCACTCTGGACGGACTTGAGAACTGTCCTTACGTGACCTAAAGCTGGAGTATTTTGAGATTGG
AGAATTAAGGCTACAGAGAAACAAGGAGACGGGAGAGCCTTGAAATGGACCTTCAAGATTACAATGTGTAGCTGACATTGTCAGTTAAGTTGGGCAG
ATCTGACTTCGCTGATTAAGTGGATGTATGAGCCGATCCTCCCCAGAAGCTGGGATCAGCTTCCCCATCTGATGAGCCAGTCTTGGTGCTCTGTGTTCAC

CTGGTTCATCTGAGGAGCTGCATCTACCCTGCCCATGCCATAGATCCTGCCCTGTTTGCTTCTCCTGTTGCTGCTAGTGGACATGAG
>ENST00000521600.5|ENSG00000249859.9|OTTHUMG00000164871.1|OTTHUMT00000380709.1|CTD-2267H22.1-018|PVT1|408|
TCAGCACTCTGGACGGACTTGAGAACTGTCCTTACGTGACCTAAAGCTGGAGTATTTTGAGATTGGAGAATTAAGGCTACAGAGAAACAAGGAGACG
GGAGAGCCTTGAAATGGACCTTCAAGATTACAATGTGTAGCTGACATTGTCAGTTAAGTTGGGCAGATCTGACTTCGCTGATTAAGTGGATGTATGAGC
CGATCCTCCCCAGAAGCTGGGATCAGCTTCCCCATCTGATGAGCCAGTCTTGGTGCTCTGTGTTCACCTGGTTCATCTGAGGAGCTGCATCTACCCTGC
CCATGCCATAGATCCTGCCCTGTTTGCTTCTCCTGTTGCTGCTAGTGGACATGAGGACAGAATAACGGGCTCCCAGATTCACAAGCCCCACCAAGAGGA
TCACCCCAGGAACG
>ENST00000522875.5|ENSG00000249859.9|OTTHUMG00000164871.1|OTTHUMT00000380710.1|CTD-2267H22.1-019|PVT1|922|
AGCTGGAGTATTTTGAGATTGGAGAATTAAGAGTGTGGGCACCCTGGCTTCAAGCTCACGAGAAACCAGAAGGAAGTCACTGTGCATAGCCATCCCCT
AAGAAGAGGGCATTCCTGCTCTTCCTTCTCCATGGCTAGAGGATCTACATGAACTATTTAGATTTTTTCTACCTGGGAGATTTAACTCCTCTCTCCTATTT
ATTTATTTATATATCAGCATGGACTTGCAGGCCAACAGAGATTTTGAGAAACACATTGAAGGATCTGTTAACACTTGATATACCCAATAAAAGCAGTGGT
TGTGCCAGTGCTGATCTGTCTTGATGTGAATGTGAACAATGGGAACCTGAGCTGAGCAGTTAATGCTAGGGTGACAGAAACTGGACCTCTCCCAAGAC
ATGTGACAGAGTAATACAGCAGCCAACTTCTTCGCCAAATTAAAGTTTTACAAGATTTAACCTGTCATCAAGACCTGGGATTTTGGTGAGCCAGTCTTG
GTGCTCTGTGTTCACCTGGTTCATCTGAGGAGCTGCATCTACCCTGCCCATGCCATAGATCCTGCCCTGTTTGCTTCTCCTGTTGCTGCTAGTGGACATG
AGGTAACCACAGCCTAGTTCTGTGATAAGCTCTTCACACCTGTACCCATATGGACTGTGGGAGAGTTTCTCTGATATGGACTGTGATGCGGATCTTGGA
CATGATACCTGGATGTGCAGCAGCCATCTGGTAATTATGAGGTGACAAGTCAATAGACTGAGATGGTGGAGCAGCAAAGATAGAAAAGAAGGACAGA
ATAACGGGCTCCCAGATTCACAAGCCCCACCAAGAGGATCACCCCAGGAACGCTTGGAGGCTGAGGAGTTCACTGAGGCTACTGCATCTTGAGACTC
AGGATGAAGACCCAGCTTGGGGCTGTCAAAGGT
>ENST00000523190.5|ENSG00000249859.9|OTTHUMG00000164871.1|OTTHUMT00000380711.1|CTD-2267H22.1-017|PVT1|443|
GTTGTGCCAGTGCTGATCTGTCTTGATGTGAATGTGAACAATGGGAACCTGAGCTGAGCAGTTAATGCTAGGGTGACAGAAACTGGACCTCTCCCAAG
ACATGTGACAGAGTAATACAGCAGCCAACTTCTTCGCCAAATTAAAGTTTTACAAGATTTAACCTGTCATCAAGACCTGGGATTTTGGTGAAGGACAG
AATAACGGGCTCCCAGATTCACAAGCCCCACCAAGAGGATCACCCCAGGAACGCTTGGAGGCTGAGGAGTTCACTGAGGCTACTGCATCTTGAGACT
CAGGATGAAGACCCAGCTTGGGGCTGTCAAAGGTTTTTTGCATGTCTGACACCCATGACTCCACCTGGACCTTATGGCTCCACCCAGAAGCAATTCAG
CCCAACAGGAGGACAGCTTCAACCCATTACGATTTCATCTCTGCCCCAACCA
>ENST00000616386.1|ENSG00000249859.9|OTTHUMG00000164871.1|-|PVT1-224|PVT1|113|
AGCCAGTCTTGGTGCTCTGTGTTCACCTGGTTCATCTGAGGAGCTGCATCTACCCTGCCCATGCCATAGATCCTGCCCTGTTTGCTTCTCCTGTTGCTGC

TAGTGGACATGAG
>ENST00000613916.1|ENSG00000249859.9|OTTHUMG00000164871.1|-|PVT1-222|PVT1|137|
AAGGACAGAATAACGGGCTCCCAGATTCACAAGCCCCACCAAGAGGATCACCCCAGGAACGCTTGGAGGCTGAGGAGTTCACTGAGGCTACTGCATC
TTGAGACTCAGGATGAAGACCCAGCTTGGGGCTGTCAAAG
>ENST00000522414.1|ENSG00000249859.9|OTTHUMG00000164871.1|OTTHUMT00000380712.1|CTD-2267H22.1-020|PVT1|654|
AGGACAGAATAACGGGCTCCCAGATTCACAAGCCCCACCAAGAGGATCACCCCAGGAACGCTTGGAGGCTGAGGAGTTCACTGAGGCTACTGCATCT
TGAGACTCAGGATGAAGACCCAGCTTGGGGCTGTCAAAGAGGCCTGAAGAGGCAGAACACCCCAGAGGAGCCTGGGGCCACCACCCAGCATCACTG
TGGGAAAACGGCAGCAGGAAATGTCCTCTCGCCTGCGTGCTCCACCTCGGTCCACGCCTTCCCTCCTTCTGGAAGCCTTGCCTGACCACTGGCCTGCC
CCTTCTATGGGAATCACTACTGACCTTGCAGCTTATTATAGACTTATATGTATGCAGAAATATAATTTTAATTACTTTTATATTGCTCCGAGAGTGATACCAA
ATCATGATAGAAAATTTAAATAAACAAAATAGCCAAATTAATACCAGTAATCACCCCAGTCAAATATACCCAGCTCACATTTTGATGTAAAATCTTCCAGT
ACAGTTTCTATGTACATATATATATTTTTAGGAAGGAGATTATATTTTACATGCTGTTTTCTAGAACGTTCTTTTAAAACTGTATCATAAACATCTTGCCTTA
TAATTCAATAGTCTTCTACAACAAACTTTGCTAATGAATTTTTTTTTTTATCATGG
>TTTY15
>ENST00000457658.5|ENSG00000233864.7|OTTHUMG00000036468.1|OTTHUMT00000088701.1|AC004810.2-002|TTTY15|630|
TTTTTTTTTTTTTTTTTGTCAGCCGGGTTCAATGCGCCATATTGAAGAGGACGGGTCTAATAGATCGCTGGAGACACAATTTAACTGAACCCCGCCCGTT
GTGGACTGACTTTGATGCTCTGAGTCCCTCCCTCCTTCACGCCGCTAGCAGGCCCTGATGTAGATTGCCTTTGTCTTACTTGGGACGTTTACCTGAGCGC
TTGGTGCTGGTGTCGGGACCGGGAGATAGGAGTGTCTCAGGAGAGACCTGGCCGAAAACCGCGAGAAAGAAAAGTGAAGCCTAGTGAAACTGCCTT
TGCAGTGACTCAAGAAAAACTCATCACCTGGAGTCCGTGTAAGCTCGGCGACAGCCCTAGCAGCGAGGCCAAAACAGTTTGGGAAGAAAGAAAACC
TAAAGTATTTGCCGTTGGTGATTCAAGGGAATCAAACTTGACGTATGGAGCCAAGAAAGCCCTTGGAAAAACTGGCCTCATATTTTGTGTACACAGTCC
CTGTACAGGGTTTCTGACCTGTGTTCAGTCTTTACAGAAGCTGACGAGAGATTTCCTTGTATTTTATGGTGGTATGATTGGCCTTAGAGCACTTCAGTTT
TGAGATCTCTGCTGTATTTGTATACAAGTATTTTGGAAG
>ENST00000440408.5|ENSG00000233864.7|OTTHUMG00000036468.1|OTTHUMT00000088700.1|AC004810.2-001|TTTY15|5284|
TTTTTTTTTTTTTTTTTGTCAGCCGGGTTCAATGCGCCATATTGAAGAGGACGGGTCTAATAGATCGCTGGAGACACAATTTAACTGAACCCCGCCCGTT
GTGGACTGACTTTGATGCTCTGAGTCCCTCCCTCCTTCACGCCGCTAGCAGGCCCTGATGTAGATTGCCTTTGTCTTACTTGGGACGTTTACCTGAGCGC
TTGGTGCTGGTGTCGGGACCGGGAGATAGGAGTGTCTCAGGAGAGACCTGGCCGAAAACCGCGAGAAAGAAAAGTGAAGCCTAGTGAAACTGCCTT
TGCAGTGACTCAAGAAAAACTCATCACCTGGAGTCCGTGTAAGCTCGGCGACAGCCCTAGCAGCGAGGCCAAAACAGTTTGGGAAGAAAGAAAACC

TAAAGTATTTGCCGTTGGTGATTCAAGGGAATCAAACTTGACGTATGGAGCCAAGAAAGCCCTTGGAAAAACTGGCCTCATATTTTGTGTACACAGTCC
CTGTACAGGGTTTCTGACCTGTGAGTCACTGAAAACTAAGCTGCGCTTTCCTAAAGTCCTGCGAACTGAAGCCAGACAACTTAAACCTCAGAAGAAA
ATAACAGCAACCTATTTATATACATAAGCCACTTTCATACCTGCCTACTGTTGTATAGACTTCAGAGTAATGTGGCCTGTATCGATTTTCCAGGAGTATTCT
TTTGTGTGTTGTTTTTTCTCAATTCCTCCTATTTTCTCTTTACAGGATGTGAGACTTCACAACCTGCTAAAAATGAGCTTTCAGGACCTACCCATATAGGA
ATAAACCATCCTAGCCATGAGAGATCAGATGAAACCTGAGACCAGAGAGACTCATTTGTTTCAAAATAGTTTCTCCAAAAGATTTTATAAAAGAAAAG
GCTGGGGGGAGTGGGATATGAAAGGAAAATGAATCTTGGGGCCCCCAAATCACTAAGCTCAAGGGATAAGTCAAGTTAGAAACTGTTCAGGGCCAAC
TTACCTTGCATTCTATTCAAATTCACCCCTCTGCTCACTTAGATGCATATCTGATTGTAATCAGAAACTCAAAAGAATGCAGCAGTTTGTCTCTCACCTAT
CTATGACCTGGAAGCCCCCTTCCCCGTTTGAGTCTTCCTGCCTTTGCTTCACTTTATCCCTGCCTTTCTAGACTGAACCAACATACTTCTTAGATATATTG
ATTGATGTCTCATGTCTCCCTAAAATGTATAAAACTAAGCTGGGCCCCAACCACCTTGGGCACATGTCGTTAGGACTTCCTGAGACTGTGTCACAAGTT
TGTGTCCACAACTTTGACAAAATAAACTTTTTAAATTAACTGAGACCTGTCCTAAATTTTTAGGGTTCCATCAGATATGCATTTCTCTCACCTGAGCATCA
GAGGGATGATTTCGAGTTCTCTGTGTTTTTTGTCCACAGGAATTTCCTTGTGGGCAAATTCTGAGGGAGGGATGTAGCTTTTTTATGTTTGGAGCTATTT
TATTTAGAAATAAAATGGGAGGCAGGTTTGCCTGAGTCAGTTGCCTGCTTGACTTCCTTTGGCTTAGTCATTTTGGAGTCCTGAGATTTATTTTCTTTTCA
CATACCTGTTTTATTTATGATGGTTTATGGTCATTGAACCTTGTAACATTGATTACATTTCTTTTCACTGGTCTTTTTTCTTAAGTAGATAAACTTATTTTTA
ACTTGGGCACAAGAGAGCATAAAAATTATATGAGGTACATAGCCTGCTTAAATAGAATATTTAAAATACGTTTTGTCCACAGAGCTGGAATTCTTTGTAA
ATGAAAACCAAATTTTACTTAATTTTTAGAGATAGGATCTTGGCTTTGTCGCCTAGGCTAGAGTGCTGTGGGGGCAATCATAGCCCACTGCAACCTCGAA
TTCCTGGCTTAAGTGATCCTCCCATCTCAGCCTCCTGGGTGCTTGGACAACAGACATGACACCTTACCTGGCTAGTTAAAAAAAAATTTTTTTTGTAGAG
ATGGGCTTTGCCTGTGTGGCCCAGGCTGCTCTTGAACTCCTAGGTTCAAGTGATCCTCCAGCTTGGGCCTTCCGAAGTGCTGGGATTACATTGCTTCCA
GCTTTAGTTTTTTAAATGTTTAACTTGTTTAACTTATTTAACTTGCTGTTTATTATTTAATGGAGAGCTTTTAGTGTTTGTCTTTTGAGGTGGGATCTTGCCA
TGCCCAGACTAGCCTTGAAGTCCTACAGTCAAGTGATCTTTGCATCTCAGTCTCCCAAGAAGTAGTGGTAGAGAATTTTTATGTTACATTCCTTTCTCTG
TGTATATGTTTGTGGGAGGACATATGTCCCTTTGTATGTTTGGGCATATATATTTTTTGTGTGAAAGTTATGCATGTTATTGTTGATCAATACAAGAGGTTT
AGAGGCCAGAGAAGAGAAATAAAATGGGAAAAACTACAAACATTCTACCTCCCATGATACCATGCTATAATGGCAATCATAATATTTATGTATTTTTTACT
TTTTTGTGTTCATTTTTAAAAATTAGATAAAAATAGTTTAAGCTAGAGGCTTCTCTCTTTCATCTTTTTTTTTCTAATTTAAGTTTTCCCTAAGTAGGAATT
TTGGTAATACTTCATTATATTGATGTATCAAGATTTCTTTAGTTTCTCCTTTGTTGTCTTTAGGTTGTCTACAATATTTTTTGTCATTGTGAATATGCTGTCAT
GAACATTTTGATGCTGATTATTACCAGATTAGTGTGTTGTGTCAAAGTTTTCATCAGTTGGATTATTTTTCAGTTTAGTAAGTGATGCAAGCCAAAACTAA
AACTCTAAGGCACCTTCTCCCCACCCAACCAGTCATCTGAGTAGACTTCCTCCTCAGCCAGGGCAGTCATAGCCCACTGCAACCAGCCAGCCCCTTTC
CATTCCAGTATCCCTTTCCCTTTAATAAAATTTAAGGCCAAACATGGTGGTTCAATGCCTGTAATGCCAGCACTTTGGGAGGCTGAGGTGGGCGGATCAT

GAGGTCAGGAGATAAAGACCATTCTGGGAAACATGGTGAAACACTGTCTCTACTAAAAGTATAAAAATTAGCTGGATGTGGTGGCATGTGGCTGTGGT
CTCAGCTACTCGGGAGGCTGCTGAGGCAGGAGAATCGCTTGAACCTGGGGAGGCAGAGGTTGCAGTGAGCAGAGATCATGCCACTACACTTTAGTCT
GGTGACAGAATGAGACTCCATCTCAAAAAAAAAAAAAATGATTTGTGAGATGAATTTGTTTTGAAAATATTAGCTTGTTTTCAGTTGTGATACATTTGAA
GTTGGTACAGAGTCCATTTTCTTTTATGTGTTAATGTATTGACAATATAGTTGTGTTGACAACTTCTTTTCTTATTTCCAGTTCAGTCTTTACAGAAGCTGA
CGAGAGATTTCCTTGTATTTTATGGTGGTATGATTGGCCTTAGAGCACTTCAGTTTTGAGATCTCTGCTGTATTTGTATACAAGTATTTTGGAAGGTCAGT
TGATAGGATGAATGAAAAACAAAAATTGGAAGTAATGGTACTAGTAATAAGTGGGGATTTTCATGTAGGTAAAGGAACTGATAAGTCATAGGGAATCCG
TGAGTTCTTAATCTTACTGAGTTTAGTTGTGTTCTTTGATTATTTTGGATAGCTTTGTTTAACGGTGAATGAAATGATTAAAATGGAAAAATTAAGTGCAG
AAAATATGTTTAACATTATATAGGATGTCTTGTTTTAGGCATTTATTGCTAGATAACATATGTCCATTCTTGTATTGCTATAAAGAAATATTGGATACTGGCT
AATTTATAAATAAAAGAGATTTATTGGCTCATGATTCTGCAGGCTGTACAGGAAATATGATTCTGGAATCCGCTTGGCTTCTGGAAAGGCCTCAGGAAAC
TTAGAATGATGACACAAGGCAGAGGGGAAGCAGGCACATCTTACATGGCAGGAGCAGGGAGCAAGAAAGAGTGAAGCGGGAGGTGCTACACACTTT
TAATAATCCAGATCTGAGTCAGGCATAGCAGCTTATGCCTATAATCCCAGCACTTTTGGGAGGCCAAGGCAGGCAGATCACCTGAGGGTCAGGAGTTCA
AGGCCAGCCTGGCCAACATGGTGAAACCTCATGTCTACTAAAAGTACAAAAATTAGCTGGGTGTGGTTGCACCTGCTTATAATCCCAGCTGCTCAGGA
GGCTGAGGCAGGAGAATTGCTTGAACCCGGGAGGCAGATGTTTCAGTGAACCAAGATTGCACCACTGCACTCCAGCCTGGGTGAACAGAGTAAGACT
CTGCCTTAAAAGTAAAATAAAATTAAATTAAAATTAAAAACCAGATCTTGCAAGAACTCACTGTCAGAAGAACAGCACCAAGGGTATGGTGCTGAACC
ATTCATGAAGGAGCCATCCTCAAGATCCAGTCATCTCATACCAGGTCCACCTCCATCTAATATTGGGAATTACAATTCATCATGAGGGTTGGTGGAGACA
TGGATCCAAGCCATGTCACATATTATCCTGACTGTAGTGGTTTAAGATAACAATTTTTATCTCACAATTTATTTTGAATACAGGCATGATTTAGCTGTGTCC
TTTGGTTCAGTATCTCTTCAAAGCTGTAATCAGGTTTTTCATGTGTTTGTCATCAACTGAAAGATTGACTGAGTATGGTTCTGAGATCTCTCAGGTGATTA
TTGGCAGAATTAAGTTCCTTTTCGATTCTTGGTCTTATTTCTTCACCCATCTACTATAGTGCATTCTTGCCATGCAGCCCTTTTTATGGAGCAAGTCACAAT
ACAGCAGCTTGCTTCATTAGGGCAAGCAAGCAAGACAAGCTGCAGCAAATGCAAGTAACATGGAAGTCTTTATAATCTAATCATGGAATTGACATAGTT
AAAAACAAATCATTAGGTAGGCTCCAAATTGATCAGAACATGGTTATTGGACAAAACCATGACTGTCAGGAGGCTGCATCATGGGGAGCCATTTTACA
AGCAGCACCATGGGTGTTATGGGGGATTTTATTACATTTGTTCTGCTCTTAAGAGTTGAAAGTCTTTAAAAATGTGTAAGTCTGTCGTTTGTTCTTGACT
TCTGTCATGTTTTCAAGAATGCATTATGCAATGATGTAGAATACTGTTTGTAAAGTAGTTGTCTAGACTCTAGTGAAAATAATTACAGATAATCTCAGTTC
ATCAACGAATCGGTATATTAATGTCATATTTAACAGTTATAGGAATAAACTAAGCATAATAATAAATGATGATTTGAATGTTATTATAATTA
>ENST00000417071.1|ENSG00000233864.7|OTTHUMG00000036468.1|OTTHUMT00000088702.1|AC004810.2-003|TTTY15|882|
GTGCTGGTGTCGGGACCGGGAGATAGGAGTGTCTCAGGAGAGACCTGGCCGAAAACCGCGAGAAAGAAAAGTGAAGCCTAGTGAAACTGCCTTTGC
AGTGACTCAAGAAAAACTCATCACCTGGAGTCCGTGTAAGCTCGGCGACAGCCCTAGCAGCGAGGCCAAAACAGTTTGGGAAGAAAGAAAACCTAA

AGTATTTGCCGTTGGTGATTCAAGGGAATCAAACTTGACGTATGGAGCCAAGAAAGCCCTTGGAAAAACTGGCCTCATATTTTGTGTACACAGTCCCTG
TACAGGGTTTCTGACCTGTGGATGTGAGACTTCACAACCTGCTAAAAATGAGCTTTCAGGACCTACCCATATAGGAATAAACCATCCTAGCCATGAGAG
ATCAGATGAAACCTGAGACCAGAGAGACTCATTTGTTTCAAAATAGTTTCTCCAAAAGATTTTATAAAAGAAAAGGCTGGGGGGAGTGGGATATGAAA
GGAAAATGAATCTTGGGGCCCCCAAATCACTAAGCTCAAGGGATAAGTCAAGTTAGAAACTGTTCAGGGCCAACTTACCTTGCATTCTATTCAAATTCA
CCCCTCTGCTCACTTAGATGCATATCTGATTGTAATCAGAAACTCAAAAGAATGCAGCAGTTTGTCTCTCACCTATCTATGACCTGGAAGCCCCCTTCCC
CGTTTGAGTCTTCCTGCCTTTGCTTCACTTTATCCCTGCCTTTCTAGACTGAACCAACATACTTCTTAGATATATTGATTGATGTCTCATGTCTCCCTAAAA
TGTATAAAACTAAGCTGGGCCCCAACCACCTTGGGCACATGTCGTTAGGACTTCCTGAGACTGTGTCACAAGTTTGTGTCCACAACTTTGACA
>TP53TG1
>ENST00000359941.9|ENSG00000182165.17|OTTHUMG00000130822.5|OTTHUMT00000253374.3|AC004023.2-001|TP53TG1|855|
GCCTGCGCGGTAGTGGGACCCGACCCTGTCTCCAGTGGGCGTCTTGGGCCCCGGCTCTATTCTGGGCTGCGGGCCTGGGAAGGGCTCGCCGGGTGCC
AAATGAGCTGTCCTAACTCTGCGGGGCTGCAGCTTCCTGCATGATGCTGGGGAGCTTGGCGCCTGACCCAGGATCTAGAAGGCACTCTGGGCAGGCCG
CGCTCCGCCCACGAAGGTACCCAACCCTCTGGGATAGATGCAGGAAGCGATGGTTAAGACCCATTTTCACCCAACTTCTCGCCGCAGGTCTGGCTTAC
CACACGCTCCTCCCCATTCCCAGTGAGCCGCTTTTTGCAGCACCAGGCGAACACTTACACCAGTGCTTTGTAAAGGAATCTTATTGTCCACCCCGTGTC
TTGGCAAAAGAACAGTGATCACACAGATTCCTACTTGGGCTCTTTCCTTTAATCTTCGGAGGCTGAGTTTGCCCAACTCAGGTTTAACCACCAAGGACT
CTGAGAGCTGGCAGGTCTGAGTAACCCTGGTAACAATTCTCTTCACCTTATCAAAACCTGAGCTAAAACCAATGCATCAGCTGATGATGACAGCAGAG
AGTGGCAGGGCTGAGGACCCAAAGTCATTTCCCAGGCTGGCGGAGAATAAACTGCCAGGGAGAAGAATGAGAAGACAGGAGACAAACTGTTTGGAA
AGCTAAATCTTCCCTCTTAATGAATAAAGGTTTTTGCCTTGTCTTAAAGTTTCTGAGTCCTAATTTTGGCAAAATAAATAACTTATGGTAATCTGGAATTG
TATTTTGTAATATTAAGTGTTTTGAACCTCTAACATTTACTTCCCAAAATCAAACTTCAGTTTCAAAAT
>ENST00000416560.5|ENSG00000182165.17|OTTHUMG00000130822.5|OTTHUMT00000334987.2|AC004023.2-005|TP53TG1|710|
GCCTGCGCGGTAGTGGGACCCGACCCTGTCTCCAGTGGGCGTCTTGGGCCCCGGCTCTATTCTGGGCTGCGGGCCTGGGAAGGGCTCGCCGGGTGCC
AAATGAGCTGTCCTAACTCTGCGGGGCTGCAGCTTCCTGCATGATGCTGGGGAGCTTGGCGCCTGACCCAGGATCTAGAAGGCACTCTGGGCAGGCCG
CGCTCCGCCCACGAAGGTCTGGCTTACCACACGCTCCTCCCCATTCCCAGTGAGCCGCTTTTTGCAGCACCAGGCGAACACTTACACCAGTGCTTTGT
AAAGGAATCTTATTGTCCACCCCGTGTCTTGGCAAAAGAACAGTGATCACACAGATTCCTACTTGGGCTCTTTCCTTTAATCTTCGGAGGCTGAGTTTG
CCCAACTCAGGTTTAACCACCAAGGACTCTGAGAGCTGGCAGGTCTGAGTAACCCTGGTAACAATTCTCTTCACCTTATCAAAACCTGAGCTAAAACC
AATGCATCAGCTGATGATGACAGCAGAGAGTGGCAGGGCTGAGGACCCAAAGTCATTTCCCAGGCTGGCGGAGAATAAACTGCCAGGGAGAAGAAT
GAGAAGACAGGAGACAAACTGTTTGGAAAGCTAAATCTTCCCTCTTAATGAATAAAGGTTTTTGCCTTGTCTTAAAGTTTCTGAGTCCTAATTTTGGCA

AAATAAATAACTTATGGTAATCTGG
>ENST00000610086.1|ENSG00000182165.17|OTTHUMG00000130822.5|OTTHUMT00000471664.1|AC004023.2-006|TP53TG1|849|
GGCTCTATTCTGGGCTGCGGGCCTGGGAAGGGCTCGCCGGGTGCCAAATGAGCTGTCCTAACTCTGCGGGGCTGCAGCTTCCTGCATGATGCTGGGGA
GCTTGGCGCCTGACCCAGGATCTAGAAGGCACTCTGGGCAGGCCGCGCTCCGCCCACGAAGGTACCCAACCCTCTGGGATAGATGCAGGAAGCGATG
GTTAAGACCCATTTTCACCCAACTTCTCGCCGCAGGTCTGGCTTACCACACGCTCCTCCCCATTCCCAGTGAGCCGCTTTTTGCAGCACCAGGCGAACA
CTTACACCAGTGCTTTGTAAAGGAATCTTATTGTCCACCCCGTGTCTTGGCAAAAGAACAGTGATCACACAGATTCCTACTTGGGCTCTTTCCTTTAATC
TTCGGAGGCTGAGTTTGCCCAACTCAGGTTTAACCACCAAGGACTCTGAGAGCTGGCAGGTCTGAGTAACCCTGGTAACAATTCTCTTCACCTTATCA
AAACCTGAGCTAAAACCAATGCATCAGCTGATGATGACAGCAGAGAGTGGCAGGGCTGAGGACCCAAAGTCATTTCCCAGGCTGGCGGAGAGTGAGT
AAATGAGATTCTAAAACTAAACAAGGGAGGTCAGAGACTCTTTCCAACCTTACCTGATGGCTTCTGGCCAAAGCAAGGAAGTGTCTTGGAAGGTGTT
GGTTGGTGATGGTGCGAAAAGACACTTGAGAAGGAGGGAAGAAAAAGCAGCGCCCCTTGATAAAGATAAACTGCCAGGGAGAAGAATGAGAAGAC
AGGAGACAAACTGTTTGGAAAGCTAAATCTTCCCTCTTAATGAATAAAGGTTTTTGCCTTGTCTTAAA
>ENST00000432193.5|ENSG00000182165.17|OTTHUMG00000130822.5|OTTHUMT00000253375.3|AC004023.2-002|TP53TG1|510|
CTCCAGTGGGCGTCTTGGGCCCCGGCTCTATTCTGGGCTGCGGGCCTGGGAAGGGCTCGCCGGGTGCCAAATGAGCTGTCCTAACTCTGCGGGGCTGC
AGCTTCCTGCATGATGCTGGGGAGCTTGGCGCCTGACCCAGGATCTAGAAGGCACTCTGGGCAGGCCGCGCTCCGCCCACGAAGGTACCCAACCCTC
TGGGATAGATGCAGGAAGCGATGGTTAAGACCCATTTTCACCCAACTTCTCGCCGCAGGTTTAACCACCAAGGACTCTGAGAGCTGGCAGGTCTGAGT
AACCCTGGTAACAATTCTCTTCACCTTATCAAAACCTGAGCTAAAACCAATGCATCAGCTGATGATGACAGCAGAGAGTGGCAGGGCTGAGGACCCAA
AGTCATTTCCCAGGCTGGCGGAGAATAAACTGCCAGGGAGAAGAATGAGAAGACAGGAGACAAACTGTTTGGAAAGCTAAATCTTCCCTCTTAATGA
ATAAAGGTTTTTGCCTTGTCTT
>ENST00000421293.1|ENSG00000182165.17|OTTHUMG00000130822.5|OTTHUMT00000253376.3|AC004023.2-003|TP53TG1|439|
CTCCAGTGGGCGTCTTGGGCCCCGGCTCTATTCTGGGCTGCGGGCCTGGGAAGGGCTCGCCGGGTGCCAAATGAGCTGTCCTAACTCTGCGGGGCTGC
AGCTTCCTGCATGATGCTGGGGAGCTTGGCGCCTGACCCAGGATCTAGAAGGCACTCTGGGCAGGCCGCGCTCCGCCCACGAAGGTTTAACCACCAA
GGACTCTGAGAGCTGGCAGGTCTGAGTAACCCTGGTAACAATTCTCTTCACCTTATCAAAACCTGAGCTAAAACCAATGCATCAGCTGATGATGACAG
CAGAGAGTGGCAGGGCTGAGGACCCAAAGTCATTTCCCAGGCTGGCGGAGAATAAACTGCCAGGGAGAAGAATGAGAAGACAGGAGACAAACTGT
TTGGAAAGCTAAATCTTCCCTCTTAATGAATAAAGGTTTTTGCCTTGTCTT
>ENST00000542586.2|ENSG00000182165.17|OTTHUMG00000130822.5|-|TP53TG1-205|TP53TG1|685|
GCCTGCGCGGTAGTGGGACCCGACCCTGTCTCCAGTGGGCGTCTTGGGCCCCGGCTCTATTCTGGGCTGCGGGCCTGGGAAGGGCTCGCCGGGTGCC

AAATGAGCTGTCCTAACTCTGCGGGGCTGCAGCTTCCTGCATGATGCTGGGGAGCTTGGCGCCTGACCCAGGATCTAGAAGGCACTCTGGGCAGGCCG
CGCTCCGCCCACGAAGGTCTGGCTTACCACACGCTCCTCCCCATTCCCAGTGAGCCGCTTTTTGCAGCACCAGGCGAACACTTACACCAGTGCTTTGT
AAAGGAATCTTATTGTCCACCCCGTGTCTTGGCAAAAGAACAGTGATCACACAGATTCCTACTTGGGCTCTTTCCTTTAATCTTCGGAGGCTGAGTTTG
CCCAACTCAGGTTTAACCACCAAGGACTCTGAGAGCTGGCAGGTCTGAGTAACCCTGGTAACAATTCTCTTCACCTTATCAAAACCTGAGCTAAAACC
AATGCATCAGCTGATGATGACAGCAGAGAGTGGCAGGGCTGAGGACCCAAAGTCATTTCCCAGGCTGGCGGAGAGTGAGTAAATGAGATTCTAAAAC
TAAACAAGGGAGGTCAGAGACTCTTTCCAACCTTACCTGATGGCTTCTGGCCAAAGCAAGGAAGTGTCTTGGAAGGTGTTGGTTGGTGATGGTGCGA
A
>ENST00000616845.1|ENSG00000182165.17|OTTHUMG00000130822.5|-|TP53TG1-207|TP53TG1|468|
CTCCAGTGGGCGTCTTGGGCCCCGGCTCTATTCTGGGCTGCGGGCCTGGGAAGGGCTCGCCGGGTGCCAAATGAGCTGTCCTAACTCTGCGGGGCTGC
AGCTTCCTGCATGATGCTGGGGAGCTTGGCGCCTGACCCAGGATCTAGAAGGCACTCTGGGCAGGCCGCGCTCCGCCCACGAAGGTACCCAACCCTC
TGGGATAGATGCAGGAAGCGATGGTTAAGACCCATTTTCACCCAACTTCTCGCCGCAGGTTTAACCACCAAGGACTCTGAGAGCTGGCAGGTCTGAGT
AACCCTGGTAACAATTCTCTTCACCTTATCAAAACCTGAGCTAAAACCAATGCATCAGCTGATGATGACAGCAGAGAGTGGCAGGGCTGAGGACCCAA
AGTCATTTCCCAGGCTGGCGGAGAGTGAGTAAATGAGATTCTAAAACTAAACAAGGGAGGTCAGAGACTCTTTCCAA
>EMX2OS
>ENST00000412075.6|ENSG00000229847.8|OTTHUMG00000019125.6|OTTHUMT00000050575.2|CTA-109P11.3-005|EMX2OS|1025|
GGAAAGAGTAAACAAAACCAGGAAAGTAATCCATTGATAAAACGCCAAGTGATGTTGTTCTGCGGAGCCAGAGTGGGGCCACCTGGGCACAATTCTC
CCACAAAGGAGCCAACTCTACTGGGGCTGAGACCCAGAGCCATCTGGACTCCCAGGCCAGCCCCAGAGCATTGAGCAGCGGGTTAGGATACTTAAGG
AGAGGCAAGTCAGGCAAGAAGCAGAAAGACAGGAAATGGAGAGATAGGAAGCAGAGAGACAGGAAGTGGAGGGACAGGAAGTGGAGAATCCAGC
AGGGGCCAATGTTGGACAACACTCCTTTGCCAGCTGTGCTCCCCATGCAACGGGATGAGGAGATTTTAAGCCTCTACCAGAAAAACTCAAATCTGGAG
ATAATTTAATTTTTCCTGGGTCTCTGCCAGCCATCTCCAGAGTTTTGTCAAATTTTCCACCTACTTCCTCTGTGTGTGGAAACTCAGCCTCCATTTTAGAA
CTCCATTCCCAACAGTGGTTCTGCACCCCCACCCATGTTTTTTTGTTTGTTTGTTTGTTTGTTTGTTTTTGGTTATCCTGGAGGTTTTGTGGATTAAGATG
TGTGAGATGAGGGGTTTATAGGGACTGGCTGGAGGTATGCCCCCCCTTCCACCCACACACCCACACACACATACACACGCACACGCTGTTGTGCAGAA
GATGTTTCTTGGGTGGCTGAGCAAAGAATCAGGTATTTGGATGTCCTGATTTGACCCTGAGAAAAATAGCAGCCATCCACAGAGCTCTGAAACATACTT
CCCACTGCTTGGAATGGCAGCTCCTCCTCAGAATACAAGCAAGGAAACGTGTGGCATCTGGCTACCATGAGCTGAAGACAAAGCCTACATGGGGAAA
AGTGCAAAACCAGGAGAATGGGAGAGAAATGGAGCCACGCACTGATGCTGTTGTGAAACATCTCAATCAAACTGCACCTGAAGACCGCCCTATCCCT
GCATTTTTCATTTACATGAGCCAATAAACTCTCTCATTTAAGTCAA

>ENST00000450314.6|ENSG00000229847.8|OTTHUMG00000019125.6|OTTHUMT00000050572.2|CTA-109P11.3-002|EMX2OS|520|
CCGCCTCCTCCTCCACCAGCACCACTCCCTCCGGGGCACCCCGGTCCCTGCAGAGTCCCTGGCCGGCGCCGCCTCCGCTGCGGCCCCCTGAATCCCGA
GCCTGCCTCGCCCAAGCTGGTAGGACAGAAGGACAGACAGATTCCTCTAGCCTAGCGCTCCGCCGCTGCTGCCTTACGCGGCCCCGCGTCGGGAGAA
CTGGGATCGCCCCAAGAGCACCGCGAGGGGTCATCTCAGGCTGGCTGCATGCCTCAGCTGAAGATCCCAGCTCCTGTCAATGCCACCTCTCTGCTTGA
CTGTCTCCTTCCAGATTCGATACAAGCAAGGAAACGTGTGGCATCTGGCTACCATGAGCTGAAGACAAAGCCTACATGGGGAAAAGTGCAAAACCAG
GAGAATGGGAGAGAAATGGAGCCACGCACTGATGCTGTTGTGAAACATCTCAATCAAACTGCACCTGAAGACCGCCCTATCCCTGCATTTTTCATTTAC
ATGAGCCAATAAACTCTCTCATTTAAGTCAA
>ENST00000424371.2|ENSG00000229847.8|OTTHUMG00000019125.6|OTTHUMT00000050574.2|CTA-109P11.3-004|EMX2OS|462|
GAAGGAGCCTTCGGAGGGAACATGCACTGCTGATTCCTTGATTCCAGATTTCTGACCTCCAGAAGCATGGGAGAACAGATTTCCCTTGTTTGAAGGCA
CCCAGTCTGTGGTCCTTCATCATGGCAGCCCTCAGGAATGGATACAGGCCCTTCCCTGCCTGCCTGGTGGAGCAGCCTCCAAGGGGAGCCTTTGCTAG
CAGGAAGAGGCATCGCATGGAGCTCAGACCCGCCTCAGAGGAACTTCTCTCAGCCTCCTCTCTCCGCACACCCGACCAAGATACAAGCAAGGAAACG
TGTGGCATCTGGCTACCATGAGCTGAAGACAAAGCCTACATGGGGAAAAGTGCAAAACCAGGAGAATGGGAGAGAAATGGAGCCACGCACTGATGC
TGTTGTGAAACATCTCAATCAAACTGCACCTGAAGACCGCCCTATCCCTGCATTTTTCATTTACATGAGCCAA
>ENST00000611404.1|ENSG00000229847.8|OTTHUMG00000019125.6|OTTHUMT00000476175.1|CTA-109P11.3-007|EMX2OS|588|
AGGAAGTGGAGGGACAGGAAGTGGAGAATCCAGCAGGGGCCAATGTTGGACAACACTCCTTTGCCAGCTGTGCTCCCCATGCAACGGGATGAGGAG
ATTTTAAGCCTCTACCAGAAAAACTCAAATCTGGAGATAATTTAATTTTTCCTGGGTCTCTGCCAGCCATCTCCAGAGTTTTGTCAAATTTTCCACCTAC
TTCCTCTGTGTGTGGAAACTCAGCCTCCATTTTAGAACTCCATTCCCAACAGTGGTTCTGCACCCCCACCCATGTTTTTTTGTTTGTTTGTTTGTTTGTTT
GTTTTTGGTTATCCTGGAGGTTTTGTGGATTAAGATGTGTGAGATGAGGGGTTTATAGGGACTGGCTGGAGGTATGCCCCCCCTTCCACCCACACACCC
ACACACACATACACACGCACACGCTGTTGTGCAGAAGATGTTTCTTGGGTGGCTGAGCAAAGAATCAGATACAAGCAAGGAAACGTGTGGCATCTGG
CTACCATGAGCTGAAGACAAAGCCTACATGGGGAAAAGTGCAAAACCAGGAGAATGGGAGAGAAATGGAGCCACGCACTGATGCTGTTGTGAAAC
>ENST00000440007.5|ENSG00000229847.8|OTTHUMG00000019125.6|OTTHUMT00000406743.2|CTA-109P11.3-001|EMX2OS|7105|
TCTCCGCGCCTTCGCCCCTCGCTCCCAGCTCACTCGCTCCTGCGGCGCCCGCTGCGGCTTCTCCTCCTCCTTCGCCTCCTCCTCTTCCTCCAGTTTCTTC
TTCTCCTCCTCCTCCTCTTCTTCCTCCTCCTCCTCTTCTTCCTCCTCCTCTTTCCCCACCTACTCCCCCTCAGCCACCGCCTCCTCCTCCACCAGCACCAC
TCCCTCCGGGGCACCCCGGTCCCTGCAGAGTCCCTGGCCGGCGCCGCCTCCGCTGCGGCCCCCTGAATCCCGAGCCTGCCTCGCCCAAGCTGGTAGG
ACAGAAGGACAGACAGATTCCTCTAGCCTAGCGCTCCGCCGCTGCTGCCTTACGCGGCCCCGCGTCGGGAGAACTGGGATCGCCCCAAGAGCACCGC
GAGGGGTCATCTCAGGCTGGCTGCATGCCTCAGCTGAAGATCCCAGCTCCTGTCAATGCCACCTCTCTGCTTGACTGTCTCCTTCCAGATTCGAGCAGG

TATGAGCTGGGAAGAATGAAGGCAGGGCATGCCCGTGTGCCAGCTCTGCACAGCTGGATAGCTGAGGAAAGATGTGGAGGAGAAGCCGGGGATTGTG
TGGAAGTCTAAGGGTGTTGTTTGCCCTTTGGGTTCCAGAAGATGCATGCCAGGACCCTGGGTGGCACTGCCAGGAAGCAACAGAGAGGAGATAAAAC
TCACAGCAGACAGACTTGCCTTAACAACAACTCCCTTGAATTAAAACACGCTTTTCAAGAAAACAAATTATCAGTTCGATCAGCAAACAGCAGAGAA
GTTTCTCTCATAATGGCAAAGAAGGGCCGGGTTGCTGACCAGTGAAAGAGCTTCAGAAAAGGAGAGGGGAGATGAGATGGCCAGAAGGAGCAAGAG
CACCGTACATCCCTGGACAACCTCATTCTAATGGGTCAGGGGCTGGGACGTGCATTTTGGAGTGCAGGAGAAGTGGCAACTCACAAATGCTAGATTTT
CTTCTAGAGATGACCAAGCTGTAGTTCTTAAAGCAGTGGCACTAGGGCAGAAAACTCTCACACTTTGATGTGCACACACAGCCCCTGGGGATCTTGTT
GACATGTAGATTCTGATTCCGTAAGTCGGGCTGAGATTCTGCATTTCCAACAAGCTCCTAGATGAGGTCCATTTTGCTGGTCCATGAAACACACTTAGAA
TAAGTAGCAAGGTATAGGAGGATACTGACTTTGCTCAGTGATGCTTGGGCTTCCGTCCAAACTAAAATAAAACAAAAGCAGACATAAATGGCCCAATT
CAACAGCCTGAGAAGTTTGGTGATAATGACCCAAGCCCTGGCCTGGTGACCAAGTGGCTGCTCAGAGAGCTCTATCTCCAAACTCCTTCCCTTCCTGC
CTCCCCTCCAAGAGTGAGTAATGTGCCAGAGTTCATTGGGGTCTGCTGTCCCCAGGGAAAGGTGGCCCAGTTGCCAAGTGCAAGTGTATAGAACGGCT
TACCTGTGCAAAGCTATCAGCCCTGGACCTATGGGCCTAGAAACATTACTTGCCACTCACTCACTCACTATCATTCATTCATTTCTGCAACAAACACTTC
CTGAGCATATGCTATACACCAGGCCCTGAGCTCAGCTCTGGGGATACAGGATGAATGGCAGATTGTGCCATCAGGAAGCCGACTGCACAGTCAGCCCG
CTGGAGCACATATGAAAAACAATACGGATGTTTTGATTTATTATCATATTGATCATTATGATGGTAGCGTATACTTTTGGAAGGCTTGCCATACATTAGGCA
TTGTGCTAAGCACATTATATGGTTAATCTCATCAATCTTCACAACCTATGTGGCAGGTACTATTATTAAACCCACTTCAAAGAGGAGGGAACTGAACCTC
AGAGAGGGTAAGTGACTTGCACAAGGACACAAGCTAGTGAGGAGAGGAACTGAGAGGAATGGCCAGACAGGCCATCAGCCTCTGTCCCCACATGCT
GGGTCTGAGTGTCTGACGGTGGACACATTCCCTTCCTGGAGGGAGAGCTCCTAGGGAGCTGATGGCAGCACTGAGTCTGGAATGTGATGGGAGTGCA
TTAGATGACGTGGGTGATCTGATGAGTACAGAGGCGTCACACTGCAATGACTTCATGCACATATCTCCCCTTGAATGCACAGAGATTCAGCAGAGGCCA
GGAGCTCAAGTTTCTGGCCCAGTGTCCCCCTCTTCCCCTGGGGAAAGCCAGGGACACACTGCCTGGCTTTGCTACACCTTTCTAGGCAAAGGCAGTAT
GCTCTCCCTCTCCTTGGCCTCCCTGGCTGTCCACACATCCACACCCACTCACACACACATGCACATGATCATACACATTACACAAGCCCACATGCCCAC
AGACATGCTCACACTCACACTCACATGCATACACTGCCATACACAACACACATACATACATTCACACACACTTGCACACACACACACACACACAGTTCT
GATGGCCAATCTTTTGGGGCTATGTTGACACTGTGAAGCAGGTGAAACAAAGTCTTTGCCACTCCCTGCTTTGGGCCTATTATGTTTGTTTTATAGTTTA
ACTCTCAAAAGACAACTTTATTTTCCAAAATTATTCAGGTTCCCAGCCAGGTGCAGTGGCTCACACCTGTAATCCCAGTACTTTGGGAGGCCAAGGCG
GGCAGATCACCTGAGGTCAGGAGTTTGAGACCAGCTTGGCCAACATGGTGAAACCCAGTCTCTACTAAAAATACAAAAATTAGCCAGACATGGTGGC
ACACACATGTAATCCCAGCTATTCGGGAGGCTGAGGCAGGAGAATCGCTTGAACCCGGGAGGCGGAGGTTGCAGTGGGCCAAGATCGTGCCACTGCA
CTCCAGCCTGGGCAACAGAGCAAGACTCCATCTCAAAAAACAAAAACAAAATCATTCAGGTTCTGTAGGGGACCCTCACTGTGTCCTGGATTCCATTC
CACTTTTAAAGTCCCAAACAGCTTGTATGGCCACTTCCCTTGACACCCAAACCAGATAGGAGTCTTAAATGACACAGAGTATTGATGGTTTCTTTAAAA

GGAAATCCACTCTCAATGGGAAGCTGGATAACTTCGCATCCAGATTTCATCCATCTTGGGCTTTGGTGGCTGCTGTTTGTTTTTGTGCAGGAGAATCTCT
CCCAGCCAACGTCGATTTCACTAACACAGTGGTTAACCTTTGCTCTCTACTCCTGCATCAATCTTCCGCCTACCGCATGTTGAGCCATGCTGCTTTCTGG
CATTCCTTTTGGATTTTCAGTTTACTACCCAACTACCAGTCCTAATAACATCAGATAAAAGGGCTTCCGCTGTGCTGCACTTGTTTTCAAAAACTATAAAT
GCCATCGACCCAGAGTTAAGTCCAGCCTCAGAGAGACAGAATATAAATACCCAAGTGGCCCTGGGCCACTGAGTTCCTCAGACATTATGTGCTATGGG
AGTTCCAGAAAGGGGGATTGCCTGGTTTTGAGTGCAGAAGGCTTCCTGGAGGAGGCGAGATTTCTGATGGGCCTCTGTTATGAGCTAAATTGAGTCCA
CCCCAAAATTCAGATGTTCAAATCCTAACCTGTAGTACCTAAGCATGTGGCCTTATTTGGAAGCAGAAGCATAACAGAGGTAATTCGTTAAGATGAGGT
CACAGTGGAAGAGGGTGGACCCCAATCTGATACAACTCGTGTCCTTATAAAAAGCAGAAATTTGAAGACAGACACACAAGAAGATGAAGGCAGAGA
CCTGGCTGATGCGGCTATAAGCCAAGGAACGCCAAAGATTGCCAGCAAAGCACCAGCAGCCAGGGAGAGGCCTCGAACAAATTCTCCCTCCCAGCAA
AAAGGAGGAAGGAAGGAACCTCCAAAAGAAGCAGGCCAGGCACAGTGGCTCACGCCTGTAATCCCAGTACTTTGGGAGGTTAAGGCAGGCGGATCA
CCTGAGATCAAGAGTTCAAGACCAGCCTGGCCAACATGGTGAAACCTCATCTCTACAGAAAAATACAAAAATTAGTCAGGTGTAGTGGCGTGCACCTG
TAGTCCCAGTTACTCGGGAGGCTGAGGCAAGAGAATCGCTTGAACCTGGGAGGTGGAGGTTGCAGTGAGCCAAGATCGTGCCATTGCACTCCAGCCT
GGGTGACAGAGCGAGACTCCATCTCAGAAAAACAAAAAAGAACCCTACTGACACCTGGATTTCAGACTTCCAGCCTCCAGAACTGTGAGATGTTACA
TTTCTGTCATTTAAACACCCAGTTTGTAGTACTTTGTTGCAACAGCTCTAGTGAGCTAATACAGCCTTGTAGGACAGGTAGGAATTTGTAATGCACAGA
GAAAGCCAACTGGCCACAGTTGTTGAAAATAATAGAATCGAAGCGCCCCTATCACTACCACCCCAGGCCATCACTTTCAAAGCTGTTCAAAATGATGC
AGCCGCGTGAGAAGGCCGGAAGGCCAGGGCGGGAGATGGGCTCCAGGTGGGCCCAGGCTGCCTGCAGAACCAGCTTGCCTCACTGCCTGGTTGCCA
AGGAGCACCTGTGGCTAGGAGGAGATGCCCAGGAAGAAAATTTGGCCACATCCCTGCCTGCCTCCCTGGCAATGGGTGATGCTCTCTGCCAGCTGGCC
CAGCTCCCCCACCACATACCTGGCTAGGAGTCAGTAGGAAAACAGTGTGTGTTTGTAGGGCTCTGCACTGAAGGGCATTAGATCCATGCAAAGGCTAA
TAAATTAGAGATAAGTGGGATTTGTAGATTTGCTGGAGGTTAGAGCTGCAGGGTCTGGCATCAGCTCTGGGTCTTTTCCATGGCCCCAGGCTGGCTCAG
GTGTGAGCTGAAGAGGCAAAACTGTCTGTTTAGGTTGCATTGGGTTCCCCTGAAAGATCTGTTGAAGTCCTAACCTCGGGTACCTCTGTTTGTGCTATT
TGCAAACAGGCTCTTTGCAGATGTAATCAAGGAAAGATGAGGCAACGCTGATTTAGTCAGGCCTTAATCCAGTGACTGGTGTCTTCATAAGACAAGGA
AATCTGGACACAGACACACAAAGAGGAAAAAGCCAAAGACACAGAGACACATGTACACAGGGAGAACTCCATGGGACAACGCAGGCACAGATCGG
AGTGACGCGGCTGCAAGCCAAGGAGCGCCAAGATTGCCAGCAACCCCCTGGGGCTGCAAGAGGTGAGAAGGAGCCTTCGGAGGGAACATGCACTGC
TGATTCCTTGATTCCAGATTTCTGACCTCCAGAAGCATGGGAGAACAGATTTCCCTTGTTTGAAGGCACCCAGTCTGTGGTCCTTCATCATGGCAGCCC
TCAGGAATGGATACAGGCCCTTCCCTGCCTGCCTGGTGGAGCAGCCTCCAAGGGGAGCCTTTGCTAGCAGGAAGAGGCATCGCATGGAGCTCAGACC
CGCCTCAGAGGAACTTCTCTCAGCCTCCTCTCTCCGCACACCCGACCAAGGTGTGAAATCCCTCCCCTTTACCTCTGCCACTTGCAGGTGGCAGGATG
CTGGAGCTGTCCCTGCCTCTTTGTAAAGTGAGGGAAGGGCAGCCCTCTCTGCTCTTTTTTTTTTTTTTTTTGAGACAGAGTCTCACTCTGTCTCCCAGG

CTGGAGTGCAATAGCACGATCTCGGCTCACTGCAACCTCCGCCTCCCAGGTTCAAGCGATTCTCCTGCCTCAACCTCCTGAGTAGCTGGAATTACAGGT
GCCTGCCACCACGCCCAGCTAATTTTGTATTGTTAGTAGAGCCAGGGTTTCACCATGTCGGCCAGGCTAGTCTCAAACTCCTGACCTCAGGTGATCCAC
CTGCCTCGGCCTCCCAAAGTGCTGCGATTACAGACATGAGCCACCACGCCCGGCCTGCTCCACTTCTAAGGCTTCTTGTGACAATGTAAGAGAAAGGA
GATGACAGAGCTTTGCAACGGGAGGAGGGCTATGTGTTCTGGTGACCAATTCACTGTCTTGTGTCGGGACAGGAAGAAGCCCTTCATACGGGCAGCA
GGCTGGGAGCCAGGGAGGAGGAAAGATCACGATCCACTCCCTGGTACATGGCCCTTCTGCACCCCGCAGTCTCCTTCCAGGTGCCACAACGAGAAGG
CACACATCCTTGGCACAGCACTTGAGGCTTTTCACCACTGGCTGCACTCACCCCTCCAGACTCACTGCCTTGCACCAACCCTTTTCCGCCCACCCCACT
CTATGCTGTCCACAGCCTCCACCCCAGCCACCTGATTCTGCAGGCCAATGTCACATTCTTCCAGTCCAGGTTCTATTCTGGCATTTCTTGTCATTATTTTG
CTGAGAATGTGTCTCTCTTGACTTTGAACTTATTGAGAGCAGGAATCATGACTCAGCCATATATCCCAGCACTTGGCCCAGGGCCTGTCGTTTCCAGGG
TAGGTGGTCTAGGCTGATTGAAGGAATGGCATTTAGTCTTTAAAATGAAAGCATGTTGCCTAGCTTGGTTATTTTTGAACTCTATAATCAAGGACTACGT
TTACCTGAATAGCCTCTGCAGAACACCAATTCCGTAAGGTGCTTCACACACACACACCAATTCTATCATTTAATACATTTTGGAAAGGCTACATACTACT
ACAGCCTCTTTTACAGATTAGCAATGTCCATGAGCGCACTAAAGGTTGAGACATTCTGCAGTGAAGAAGCCTATTTCATTTTGTTTAACCAAGTATTTCT
CAAATTTATTTGATCATATGTGGCAGAAAATGCTGTGTCTGGCATTCTTCATTTCCCTCTTCCTCCTTTAACATGGAACCCCTGATGTCTTTAGCTTGGCA
CATCGCCACCCAGAATAAAAACTACCTTTCCCAGCTCTTCCTGCAGCTAGGGGCAGCCCTGGGATAAATTCTGGACAATGAAATACAGGCAGAAGTAA
ATCATATACGATTTCCATGAAGGGACCTTAAACAGAAGTGTGCCCTTCTCTTCCCCACACATTCCTCCTCCTGTCTGAAATGTAGATGCAACTGCTGGCA
TTTGAGCAGCCATCTTGGGCCATGTGGTAGCTTCTTATGGATGATCTAGGACTAATTCAAGGGTCTAGATTTACCTCCAAACTTTGTTTATCTACAAAAA
ATAAAACTCTATCTTCTTAAAGC
>ENST00000551288.5|ENSG00000229847.8|OTTHUMG00000019125.6|OTTHUMT00000406746.1|CTA-109P11.3-006|EMX2OS|7280|
GTGCTTGGCTACAGCCGCTGCTGCCTCTCGCGAACTGGGCTCCGGGGCTCCCGGCTCCCGAGAACTAGAAGAGAAACGCGAGCGAAGGGATCGAAA
CCCGGGGGGTTACCGACTTGCAGACACCGCCAGGACAGTCTGTAACGCAGGAAGATCCCAGCGGCTCCGGGTCTGGTGAGGGGACCATAAGCATGAC
TGATAGCGAATGAGGAAGGGCAGCCCTAAACTTTTCAAGCAAAGCCTCAGAGTTTTGGGTTCACTCATTAGCATAGGAAATCGATTCACCGAAAACCC
AAACAAAGAAAAACAAGCCGACAGTCCAGGCAGGATGCAGGCAAATCCAGTTCGGGATTAAGGGTAAAAGGCTTTTTGGGTTTTTTTTCCTTTGGTTT
GATTTTTTAAAATATGGGGAGGGGGGTGACATCTACCCGATTCTAGGCTCCGGCAGGAACGCAATGGGTTAATGAATGGACAAGCCGCGGAGTATTGAT
CGGCTGCCGCCGGAGAAAGAAAGAAAAACAAAAACCAGACCGAACCTGCCTTCCCGCTGTGGCTGCTCGGCGCCCCAATTAAGCAGGGTCATCTCA
GGCTGGCTGCATGCCTCAGCTGAAGATCCCAGCTCCTGTCAATGCCACCTCTCTGCTTGACTGTCTCCTTCCAGATTCGAGCAGGTATGAGCTGGGAAG
AATGAAGGCAGGGCATGCCCGTGTGCCAGCTCTGCACAGCTGGATAGCTGAGGAAAGATGTGGAGGAGAAGCCGGGGATTGTGTGGAAGTCTAAGG
GTGTTGTTTGCCCTTTGGGTTCCAGAAGATGCATGCCAGGACCCTGGGTGGCACTGCCAGGAAGCAACAGAGAGGAGATAAAACTCACAGCAGACAG

ACTTGCCTTAACAACAACTCCCTTGAATTAAAACACGCTTTTCAAGAAAACAAATTATCAGTTCGATCAGCAAACAGCAGAGAAGTTTCTCTCATAATG
GCAAAGAAGGGCCGGGTTGCTGACCAGTGAAAGAGCTTCAGAAAAGGAGAGGGGAGATGAGATGGCCAGAAGGAGCAAGAGCACCGTACATCCCT
GGACAACCTCATTCTAATGGGTCAGGGGCTGGGACGTGCATTTTGGAGTGCAGGAGAAGTGGCAACTCACAAATGCTAGATTTTCTTCTAGAGATGAC
CAAGCTGTAGTTCTTAAAGCAGTGGCACTAGGGCAGAAAACTCTCACACTTTGATGTGCACACACAGCCCCTGGGGATCTTGTTGACATGTAGATTCT
GATTCCGTAAGTCGGGCTGAGATTCTGCATTTCCAACAAGCTCCTAGATGAGGTCCATTTTGCTGGTCCATGAAACACACTTAGAATAAGTAGCAAGGT
ATAGGAGGATACTGACTTTGCTCAGTGATGCTTGGGCTTCCGTCCAAACTAAAATAAAACAAAAGCAGACATAAATGGCCCAATTCAACAGCCTGAGA
AGTTTGGTGATAATGACCCAAGCCCTGGCCTGGTGACCAAGTGGCTGCTCAGAGAGCTCTATCTCCAAACTCCTTCCCTTCCTGCCTCCCCTCCAAGAG
TGAGTAATGTGCCAGAGTTCATTGGGGTCTGCTGTCCCCAGGGAAAGGTGGCCCAGTTGCCAAGTGCAAGTGTATAGAACGGCTTACCTGTGCAAAGC
TATCAGCCCTGGACCTATGGGCCTAGAAACATTACTTGCCACTCACTCACTCACTATCATTCATTCATTTCTGCAACAAACACTTCCTGAGCATATGCTAT
ACACCAGGCCCTGAGCTCAGCTCTGGGGATACAGGATGAATGGCAGATTGTGCCATCAGGAAGCCGACTGCACAGTCAGCCCGCTGGAGCACATATG
AAAAACAATACGGATGTTTTGATTTATTATCATATTGATCATTATGATGGTAGCGTATACTTTTGGAAGGCTTGCCATACATTAGGCATTGTGCTAAGCACA
TTATATGGTTAATCTCATCAATCTTCACAACCTATGTGGCAGGTACTATTATTAAACCCACTTCAAAGAGGAGGGAACTGAACCTCAGAGAGGGTAAGT
GACTTGCACAAGGACACAAGCTAGTGAGGAGAGGAACTGAGAGGAATGGCCAGACAGGCCATCAGCCTCTGTCCCCACATGCTGGGTCTGAGTGTCT
GACGGTGGACACATTCCCTTCCTGGAGGGAGAGCTCCTAGGGAGCTGATGGCAGCACTGAGTCTGGAATGTGATGGGAGTGCATTAGATGACGTGGG
TGATCTGATGAGTACAGAGGCGTCACACTGCAATGACTTCATGCACATATCTCCCCTTGAATGCACAGAGATTCAGCAGAGGCCAGGAGCTCAAGTTT
CTGGCCCAGTGTCCCCCTCTTCCCCTGGGGAAAGCCAGGGACACACTGCCTGGCTTTGCTACACCTTTCTAGGCAAAGGCAGTATGCTCTCCCTCTCCT
TGGCCTCCCTGGCTGTCCACACATCCACACCCACTCACACACACATGCACATGATCATACACATTACACAAGCCCACATGCCCACAGACATGCTCACAC
TCACACTCACATGCATACACTGCCATACACAACACACATACATACATTCACACACACTTGCACACACACACACACACACAGTTCTGATGGCCAATCTTT
TGGGGCTATGTTGACACTGTGAAGCAGGTGAAACAAAGTCTTTGCCACTCCCTGCTTTGGGCCTATTATGTTTGTTTTATAGTTTAACTCTCAAAAGACA
ACTTTATTTTCCAAAATTATTCAGGTTCCCAGCCAGGTGCAGTGGCTCACACCTGTAATCCCAGTACTTTGGGAGGCCAAGGCGGGCAGATCACCTGAG
GTCAGGAGTTTGAGACCAGCTTGGCCAACATGGTGAAACCCAGTCTCTACTAAAAATACAAAAATTAGCCAGACATGGTGGCACACACATGTAATCCC
AGCTATTCGGGAGGCTGAGGCAGGAGAATCGCTTGAACCCGGGAGGCGGAGGTTGCAGTGGGCCAAGATCGTGCCACTGCACTCCAGCCTGGGCAA
CAGAGCAAGACTCCATCTCAAAAAACAAAAACAAAATCATTCAGGTTCTGTAGGGGACCCTCACTGTGTCCTGGATTCCATTCCACTTTTAAAGTCCC
AAACAGCTTGTATGGCCACTTCCCTTGACACCCAAACCAGATAGGAGTCTTAAATGACACAGAGTATTGATGGTTTCTTTAAAAGGAAATCCACTCTCA
ATGGGAAGCTGGATAACTTCGCATCCAGATTTCATCCATCTTGGGCTTTGGTGGCTGCTGTTTGTTTTTGTGCAGGAGAATCTCTCCCAGCCAACGTCGA
TTTCACTAACACAGTGGTTAACCTTTGCTCTCTACTCCTGCATCAATCTTCCGCCTACCGCATGTTGAGCCATGCTGCTTTCTGGCATTCCTTTTGGATTT

TCAGTTTACTACCCAACTACCAGTCCTAATAACATCAGATAAAAGGGCTTCCGCTGTGCTGCACTTGTTTTCAAAAACTATAAATGCCATCGACCCAGA
GTTAAGTCCAGCCTCAGAGAGACAGAATATAAATACCCAAGTGGCCCTGGGCCACTGAGTTCCTCAGACATTATGTGCTATGGGAGTTCCAGAAAGGG
GGATTGCCTGGTTTTGAGTGCAGAAGGCTTCCTGGAGGAGGCGAGATTTCTGATGGGCCTCTGTTATGAGCTAAATTGAGTCCACCCCAAAATTCAGAT
GTTCAAATCCTAACCTGTAGTACCTAAGCATGTGGCCTTATTTGGAAGCAGAAGCATAACAGAGGTAATTCGTTAAGATGAGGTCACAGTGGAAGAGG
GTGGACCCCAATCTGATACAACTCGTGTCCTTATAAAAAGCAGAAATTTGAAGACAGACACACAAGAAGATGAAGGCAGAGACCTGGCTGATGCGGC
TATAAGCCAAGGAACGCCAAAGATTGCCAGCAAAGCACCAGCAGCCAGGGAGAGGCCTCGAACAAATTCTCCCTCCCAGCAAAAAGGAGGAAGGAA
GGAACCTCCAAAAGAAGCAGGCCAGGCACAGTGGCTCACGCCTGTAATCCCAGTACTTTGGGAGGTTAAGGCAGGCGGATCACCTGAGATCAAGAGT
TCAAGACCAGCCTGGCCAACATGGTGAAACCTCATCTCTACAGAAAAATACAAAAATTAGTCAGGTGTAGTGGCGTGCACCTGTAGTCCCAGTTACTC
GGGAGGCTGAGGCAAGAGAATCGCTTGAACCTGGGAGGTGGAGGTTGCAGTGAGCCAAGATCGTGCCATTGCACTCCAGCCTGGGTGACAGAGCGA
GACTCCATCTCAGAAAAACAAAAAAGAACCCTACTGACACCTGGATTTCAGACTTCCAGCCTCCAGAACTGTGAGATGTTACATTTCTGTCATTTAAA
CACCCAGTTTGTAGTACTTTGTTGCAACAGCTCTAGTGAGCTAATACAGCCTTGTAGGACAGGTAGGAATTTGTAATGCACAGAGAAAGCCAACTGGC
CACAGTTGTTGAAAATAATAGAATCGAAGCGCCCCTATCACTACCACCCCAGGCCATCACTTTCAAAGCTGTTCAAAATGATGCAGCCGCGTGAGAAG
GCCGGAAGGCCAGGGCGGGAGATGGGCTCCAGGTGGGCCCAGGCTGCCTGCAGAACCAGCTTGCCTCACTGCCTGGTTGCCAAGGAGCACCTGTGG
CTAGGAGGAGATGCCCAGGAAGAAAATTTGGCCACATCCCTGCCTGCCTCCCTGGCAATGGGTGATGCTCTCTGCCAGCTGGCCCAGCTCCCCCACCA
CATACCTGGCTAGGAGTCAGTAGGAAAACAGTGTGTGTTTGTAGGGCTCTGCACTGAAGGGCATTAGATCCATGCAAAGGCTAATAAATTAGAGATAA
GTGGGATTTGTAGATTTGCTGGAGGTTAGAGCTGCAGGGTCTGGCATCAGCTCTGGGTCTTTTCCATGGCCCCAGGCTGGCTCAGGTGTGAGCTGAAG
AGGCAAAACTGTCTGTTTAGGTTGCATTGGGTTCCCCTGAAAGATCTGTTGAAGTCCTAACCTCGGGTACCTCTGTTTGTGCTATTTGCAAACAGGCTC
TTTGCAGATGTAATCAAGGAAAGATGAGGCAACGCTGATTTAGTCAGGCCTTAATCCAGTGACTGGTGTCTTCATAAGACAAGGAAATCTGGACACAG
ACACACAAAGAGGAAAAAGCCAAAGACACAGAGACACATGTACACAGGGAGAACTCCATGGGACAACGCAGGCACAGATCGGAGTGACGCGGCTG
CAAGCCAAGGAGCGCCAAGATTGCCAGCAACCCCCTGGGGCTGCAAGAGGTGAGAAGGAGCCTTCGGAGGGAACATGCACTGCTGATTCCTTGATTC
CAGATTTCTGACCTCCAGAAGCATGGGAGAACAGATTTCCCTTGTTTGAAGGCACCCAGTCTGTGGTCCTTCATCATGGCAGCCCTCAGGAATGGATAC
AGGCCCTTCCCTGCCTGCCTGGTGGAGCAGCCTCCAAGGGGAGCCTTTGCTAGCAGGAAGAGGCATCGCATGGAGCTCAGACCCGCCTCAGAGGAAC
TTCTCTCAGCCTCCTCTCTCCGCACACCCGACCAAGGTGTGAAATCCCTCCCCTTTACCTCTGCCACTTGCAGGTGGCAGGATGCTGGAGCTGTCCCTG
CCTCTTTGTAAAGTGAGGGAAGGGCAGCCCTCTCTGCTCTTTTTTTTTTTTTTTTTGAGACAGAGTCTCACTCTGTCTCCCAGGCTGGAGTGCAATAGC
ACGATCTCGGCTCACTGCAACCTCCGCCTCCCAGGTTCAAGCGATTCTCCTGCCTCAACCTCCTGAGTAGCTGGAATTACAGGTGCCTGCCACCACGC
CCAGCTAATTTTGTATTGTTAGTAGAGCCAGGGTTTCACCATGTCGGCCAGGCTAGTCTCAAACTCCTGACCTCAGGTGATCCACCTGCCTCGGCCTCC

CAAAGTGCTGCGATTACAGACATGAGCCACCACGCCCGGCCTGCTCCACTTCTAAGGCTTCTTGTGACAATGTAAGAGAAAGGAGATGACAGAGCTTT
GCAACGGGAGGAGGGCTATGTGTTCTGGTGACCAATTCACTGTCTTGTGTCGGGACAGGAAGAAGCCCTTCATACGGGCAGCAGGCTGGGAGCCAGG
GAGGAGGAAAGATCACGATCCACTCCCTGGTACATGGCCCTTCTGCACCCCGCAGTCTCCTTCCAGGTGCCACAACGAGAAGGCACACATCCTTGGC
ACAGCACTTGAGGCTTTTCACCACTGGCTGCACTCACCCCTCCAGACTCACTGCCTTGCACCAACCCTTTTCCGCCCACCCCACTCTATGCTGTCCACA
GCCTCCACCCCAGCCACCTGATTCTGCAGGCCAATGTCACATTCTTCCAGTCCAGGTTCTATTCTGGCATTTCTTGTCATTATTTTGCTGAGAATGTGTCT
CTCTTGACTTTGAACTTATTGAGAGCAGGAATCATGACTCAGCCATATATCCCAGCACTTGGCCCAGGGCCTGTCGTTTCCAGGGTAGGTGGTCTAGGC
TGATTGAAGGAATGGCATTTAGTCTTTAAAATGAAAGCATGTTGCCTAGCTTGGTTATTTTTGAACTCTATAATCAAGGACTACGTTTACCTGAATAGCCT
CTGCAGAACACCAATTCCGTAAGGTGCTTCACACACACACACCAATTCTATCATTTAATACATTTTGGAAAGGCTACATACTACTACAGCCTCTTTTACA
GATTAGCAATGTCCATGAGCGCACTAAAGGTTGAGACATTCTGCAGTGAAGAAGCCTATTTCATTTTGTTTAACCAAGTATTTCTCAAATTTATTTGATCA
TATGTGGCAGAAAATGCTGTGTCTGGCATTCTTCATTTCCCTCTTCCTCCTTTAACATGGAACCCCTGATGTCTTTAGCTTGGCACATCGCCACCCAGAAT
AAAAACTACCTTTCCCAGCTCTTCCTGCAGCTAGGGGCAGCCCTGGGATAAATTCTGGACAATGAAATACAGGCAGAAGTAAATCATATACGATTTCCA
TGAAGGGACCTTAAACAGAAGTGTGCCCTTCTCTTCCCCACACATTCCTCCTCCTGTCTGAAATGTAGATGCAACTGCTGGCATTTGAGCAGCCATCTT
GGGCCATGTGGTAGCTTCTTATGGATGATCTAGGACTAATTCAAGGGTCTAGATTTACCTCCAAACTTTGTTTATCTACAAAAAATAAAACTCTATCTTCT
TAAAGC
>ENST00000423419.1|ENSG00000229847.8|OTTHUMG00000019125.6|OTTHUMT00000050573.1|CTA-109P11.3-003|EMX2OS|472|
GAACAGAACAATAGTATGCTCTCAATAAATAAGGATGAACGCATGATTGAAATAGTTTAAAAATCAAGACCCGGAAAACTGAAGCTTAAAAAGGAAGA
GAAGGACATCAGAAAGTTGATGGGTCATCTCAGGCTGGCTGCATGCCTCAGCTGAAGATCCCAGCTCCTGTCAATGCCACCTCTCTGCTTGACTGTCTC
CTTCCAGATTCGAGCAGGTATGAGCTGGGAAGAATGAAGGCAGGGCATGCCCGTGTGCCAGCTCTGCACAGCTGGATAGCTGAGGAAAGATGTGGAG
GAGAAGCCGGGGATTGTGTGGAAGTCTAAGGGTGTTGTTTGCCCTTTGGGTTCCAGAAGATGCATGCCAGGACCCTGGGTGGCACTGCCAGGAAGCA
ACAGAGAGGAGATAAAACTCACAGCAGACAGACTTGCCTTAACAACAACTCCCTTGAATTAAAACACGCTTTTCAAGAAAA
