# Supplementary material for: Integrated analysis of lncRNA-associated ceRNA network identified potential regulatory interactions in osteosarcoma
Source: Genet Mol Biol. 2020 May 20;43(2):e20190090. doi: 10.1590/1678-4685-GMB-2019-0090 (PMC7252519; doi:10.1590/1678-4685-GMB-2019-0090)
Supplement: Table S1 [file 1415-4757-GMB-43-2-e20190090-s2.pdf]

Genetics and Molecular Biology 43, 2, (2020)

Supplementary Material to “Integrated analysis of lncRNA-associated ceRNA network identified potential regulatory interactions in osteosarcoma”

Table S1 - Updated list of miRNAs using miRBase version 22.1.

miRNA	Version_21	Version_22
hsa-miR-182	hsa-miR-182-5p	hsa-miR-182-5p
hsa-miR-486-5p	hsa-miR-486-5p	hsa-miR-486-5p
hsa-miR-760	hsa-miR-760	hsa-miR-760
hsa-miR-941	hsa-miR-941	hsa-miR-941
hsa-miR-4539	hsa-miR-4539	hsa-miR-4539
hsa-miR-346	hsa-miR-346	hsa-miR-346
hsa-miR-4665-5p	hsa-miR-4665-5p	hsa-miR-4665-5p
hsa-miR-4449	hsa-miR-4449	hsa-miR-4449
hsa-miR-1292	hsa-miR-1292-5p	hsa-miR-1292-5p
hsa-miR-2276	hsa-miR-2276-3p	hsa-miR-2276-3p
hsa-miR-331-5p	hsa-miR-331-5p	hsa-miR-331-5p
hsa-miR-1231	hsa-miR-1231	hsa-miR-1231
hsa-miR-151-5p	hsa-miR-151a-5p	hsa-miR-151a-5p
hsa-miR-4486	hsa-miR-4486	hsa-miR-4486
hsa-miR-744	hsa-miR-744-5p	hsa-miR-744-5p
hsa-miR-3180-3p	hsa-miR-3180-3p	hsa-miR-3180-3p
hsa-miR-4443	hsa-miR-4443	hsa-miR-4443
hsa-miR-106b*	hsa-miR-106b-3p	hsa-miR-106b-3p
hsa-miR-3131	hsa-miR-3131	hsa-miR-3131
hsa-miR-93	hsa-miR-93-5p	hsa-miR-93-5p
hsa-miR-4728-5p	hsa-miR-4728-5p	hsa-miR-4728-5p
hsa-miR-551b*	hsa-miR-551b-5p	hsa-miR-551b-5p
hsa-miR-151b	hsa-miR-151b	hsa-miR-151b
hsa-miR-769-3p	hsa-miR-769-3p	hsa-miR-769-3p
hsa-miR-1208	hsa-miR-1208	hsa-miR-1208
hsa-miR-3607-5p	hsa-miR-3607-5p	/
hsa-miR-16-2*	hsa-miR-16-2-3p	hsa-miR-16-2-3p
hsa-miR-3916	hsa-miR-3916	hsa-miR-3916
hsa-miR-221	hsa-miR-221-3p	hsa-miR-221-3p
hsa-miR-146b-5p	hsa-miR-146b-5p	hsa-miR-146b-5p

miRNA	Version_21	Version_22
hsa-miR-4659a-5p	hsa-miR-4659a-5p	hsa-miR-4659a-5p
hsa-miR-362-3p	hsa-miR-362-3p	hsa-miR-362-3p
hsa-miR-4784	hsa-miR-4784	hsa-miR-4784
hsa-miR-1294	hsa-miR-1294	hsa-miR-1294
hsa-miR-4436b-3p	hsa-miR-4436b-3p	hsa-miR-4436b-3p
hsa-miR-4423-3p	hsa-miR-4423-3p	hsa-miR-4423-3p
hsa-miR-4288	hsa-miR-4288	hsa-miR-4288
hsa-miR-424	hsa-miR-424-5p	hsa-miR-424-5p
hsa-miR-411*	hsa-miR-411-3p	hsa-miR-411-3p
hsa-miR-323-3p	hsa-miR-323a-3p	hsa-miR-323a-3p
hsa-miR-29b-1*	hsa-miR-29b-1-5p	hsa-miR-29b-1-5p
hsa-miR-493*	hsa-miR-493-5p	hsa-miR-493-5p
hsa-miR-1270	hsa-miR-1270	hsa-miR-1270
hsa-miR-329	hsa-miR-329-3p	hsa-miR-329-3p
hsa-miR-199b-5p	hsa-miR-199b-5p	hsa-miR-199b-5p
hsa-miR-377*	hsa-miR-377-5p	hsa-miR-377-5p
hsa-miR-495	hsa-miR-495-3p	hsa-miR-495-3p
hsa-let-7a-2*	hsa-let-7a-2-3p	hsa-let-7a-2-3p
hsa-miR-485-3p	hsa-miR-485-3p	hsa-miR-485-3p
hsa-miR-758	hsa-miR-758-3p	hsa-miR-758-3p
hsa-miR-543	hsa-miR-543	hsa-miR-543
hsa-miR-154	hsa-miR-154-5p	hsa-miR-154-5p
hsa-miR-34a*	hsa-miR-34a-3p	hsa-miR-34a-3p
hsa-miR-34a	hsa-miR-34a-5p	hsa-miR-34a-5p
