# Supplementary material for: Integrated analysis of lncRNA-associated ceRNA network identified potential regulatory interactions in osteosarcoma
Source: Genet Mol Biol. 2020 May 20;43(2):e20190090. doi: 10.1590/1678-4685-GMB-2019-0090 (PMC7252519; doi:10.1590/1678-4685-GMB-2019-0090)
Supplement: Table S2 [file 1415-4757-GMB-43-2-e20190090-s3.pdf]

Genetics and Molecular Biology 43, 2, (2020)

Supplementary Material to "Integrated analysis of lncRNA-associated ceRNA network identified potential regulatory interactions in osteosarcoma"

Table S2 - Standardization and annotation mRNA data.

Symbol	Log FC	Ave Expression	t	P Value	adj. P. Value	B
ABI3BP	-4.949026748	4.846037635	-26.70714749	4.32E-07	0.007165822	3.128825519
TENM4	-3.699191517	6.683834905	-24.40740493	7.10E-07	0.007165822	3.05250884
TRIM22	-5.51886155	5.484939789	-22.05565167	1.24E-06	0.008354979	2.951625669
PLD5	-2.999529396	4.131808552	-19.02023321	2.80E-06	0.014154447	2.771117635
KRTAP1-1	-3.123169814	5.290961491	-16.63383465	5.85E-06	0.022708238	2.568563591
APCDD1L	-3.694586986	7.6610927	-16.20429091	6.75E-06	0.022708238	2.524253115
LINC00607	-2.881691784	5.564164442	-15.48170012	8.66E-06	0.024971453	2.443045144
CXCL5	-2.246590429	4.111855897	-14.56745636	1.21E-05	0.028104026	2.326629932
NETO1	-2.166787679	4.698519793	-14.41685956	1.28E-05	0.028104026	2.30581241
PTGS1	-2.244861604	6.220213892	-14.18925397	1.39E-05	0.028104026	2.273391951
MMP13	-3.944510609	5.761948213	-13.36860643	1.92E-05	0.035176025	2.146123733
PCDH19	-3.164761961	4.889250216	-13.16456158	2.09E-05	0.035176025	2.11173728
DNALI1	-2.225227392	4.958145166	-11.9030238	3.60E-05	0.053073162	1.870736223
B3GALT2	-1.954720067	3.94585975	-11.85577022	3.68E-05	0.053073162	1.86065361
COMP	-3.340126478	6.183179846	-11.57957729	4.18E-05	0.056230287	1.80001974
KCNK2	-4.485722919	5.327553911	-11.42887994	4.48E-05	0.05655888	1.76566855

Symbol	Log FC	Ave Expression	t	P Value	adj. P. Value	B
ISLR	-1.737767573	6.644130363	-11.15885839	5.10E-05	0.060516889	1.701763556
MIR34A	-3.092889812	5.854166778	-10.94289751	5.66E-05	0.063461612	1.648375479
EBF2	-1.8631628	4.829155905	-10.57881903	6.78E-05	0.072039952	1.553495479
POU3F3	-1.568558003	4.944102034	-10.35397186	7.60E-05	0.076745616	1.4916567
ZNF826P	-1.612791818	4.56967814	-9.977437172	9.26E-05	0.089010827	1.382144829
PQLC2L	-2.082376837	4.715935089	-9.886362244	9.72E-05	0.089203092	1.354477555
XG	-1.362369226	4.744246303	-9.146255793	0.000146669	0.128737253	1.110993681
LOC101060391	-1.794035429	4.751056652	-8.938119591	0.000165541	0.139247854	1.036048694
ZNF492	-1.432690691	3.925366578	-8.514183933	0.00021351	0.166497943	0.873657983
GRAMD2B	-2.190884894	6.936867148	-8.507165264	0.000214432	0.166497943	0.870854035
KCNJ6	-1.259739269	4.757339581	-8.316825359	0.000241282	0.180407604	0.793311075
IGSF5	-2.260447285	5.023610728	-7.812442162	0.000333728	0.232468103	0.573037061
C5orf30	-1.4819046	8.81772879	-7.811482597	0.00033394	0.232468103	0.572596419
GPAT2	-1.566600099	6.227555755	-7.559858995	0.00039527	0.253946503	0.45405259
EOGT	-1.656127763	8.075666322	-7.512063551	0.000408353	0.253946503	0.430845251
MDFIC	-1.929315633	7.891908248	-7.470513721	0.000420138	0.253946503	0.410486795
ZBTB16	-1.354978599	4.288603598	-7.403766457	0.00043991	0.253946503	0.377420191
KRT19	-1.353630703	5.169497829	-7.403464812	0.000440002	0.253946503	0.377269735
DUSP15	-1.523822607	7.14922069	-7.35840384	0.000453966	0.253946503	0.354689885
CCL20	-1.830901507	4.598253275	-7.352980236	0.000455682	0.253946503	0.351958145
GALNT10	-1.763628855	8.162927712	-7.316672612	0.000467361	0.253946503	0.333592896
LRRC32	-1.602913512	6.675842487	-7.284491274	0.000478005	0.253946503	0.31720077
TACSTD2	-1.139476936	5.620929139	-7.100944637	0.000544393	0.281800206	0.22161968
METTL7B	-1.966162845	6.181747436	-7.038052108	0.000569567	0.284333918	0.18803383
EGR2	-1.553247519	5.321774258	-7.006856021	0.000582554	0.284333918	0.171213073

Symbol	Log FC	Ave Expression	t	P Value	adj. P. Value	B
HMOX1	-2.924635548	8.674690285	-6.985740006	0.000591541	0.284333918	0.159766155
NTSR1	-1.899432067	7.393685046	-6.950513799	0.000606893	0.284929383	0.140559321
MVD	-1.603433802	7.468986924	-6.865842101	0.000645729	0.296272255	0.09381976
PTGDS	-2.28770198	7.610193202	-6.823164065	0.000666395	0.298959429	0.069950608
RALA	-1.500259419	10.82342562	-6.764789594	0.000695918	0.305417069	0.036961008
FLG	-1.578397493	4.305037922	-6.67022019	0.000747033	0.320874379	-0.01733378
TAGLN3	-1.264045511	6.499307507	-6.565728727	0.000808656	0.337061551	-0.07857114
SIPA1L2	2.747861361	7.064837791	6.52328622	0.000835358	0.337061551	-0.10382492
TRPV2	-1.854019594	6.351473572	-6.484639395	0.000860565	0.337061551	-0.12701397
GRIA1	-1.045631002	4.24789628	-6.481621138	0.00086257	0.337061551	-0.12883282
FN1	-3.463546294	10.35180468	-6.473192838	0.000868199	0.337061551	-0.13391788
MANSC1	-1.766107101	5.312380259	-6.415384562	0.000907976	0.338464076	-0.16903565
EMX2	-1.104573258	4.075147588	-6.390602935	0.000925672	0.338464076	-0.18421945
CTSK	-5.336728941	8.517490577	-6.371172122	0.000939827	0.338464076	-0.19617945
RNF128	-1.594190474	4.0101386	-6.323805932	0.000975397	0.338464076	-0.22553685
DGKI	-2.095093009	4.906945231	-6.31844897	0.000979517	0.338464076	-0.22887526
LOC102723409	-1.234737741	4.40607857	-6.306436221	0.00098883	0.338464076	-0.23637501
NNT-AS1	1.687738649	8.533801193	6.305999108	0.000989171	0.338464076	-0.23664826
TMEM100	-1.190072171	4.35298996	-6.261028205	0.001024972	0.344868839	-0.2648934
PCSK9	-1.215241358	5.908144618	-6.21994165	0.001058998	0.350476369	-0.29093032
P4HA3	-1.430465076	5.840150914	-6.132383634	0.001135971	0.369886957	-0.3471632
NKAPL	-1.04226916	4.912599894	-6.080367787	0.001184762	0.377741478	-0.38105707
FNDC4	-1.426367581	7.445884864	-6.060804723	0.001203736	0.377741478	-0.39389958
DDB2	-1.741557735	8.473126641	-6.045020329	0.001219302	0.377741478	-0.40429963
SLC35F4	-1.995851534	4.520351076	-6.029397501	0.001234938	0.377741478	-0.41462688

Symbol	Log FC	Ave Expression	t	P Value	adj. P. Value	B
MEOX2	-1.17791485	3.653581198	-6.00699921	0.001257763	0.378980821	-0.42949157
EYA2	-2.913770336	6.31723687	-5.959956466	0.001307308	0.388116584	-0.46093781
TNFSF4	-3.013983994	5.998804441	-5.892140682	0.001382757	0.399581365	-0.50681456
C11orf45	-1.596464795	4.809892059	-5.889746758	0.001385511	0.399581365	-0.50844587
KIAA0408	-1.075556714	3.931689191	-5.861171017	0.001418879	0.40344129	-0.5279813
EHD3	-1.023424916	6.82377246	-5.805437274	0.001486669	0.41200024	-0.56641841
DOCK4	-1.368882227	6.53248491	-5.802936692	0.001489797	0.41200024	-0.56815341
FBLN5	-7.230257775	7.548541536	-5.729508657	0.001585132	0.42765801	-0.6195046
TMEM35A	-4.149106857	5.47100375	-5.722490943	0.001594607	0.42765801	-0.62445351
SGIP1	-4.134745751	4.746084732	-5.71121597	0.001609967	0.42765801	-0.63241978
SCN9A	-1.025531717	3.79574726	-5.681611544	0.001651116	0.432892678	-0.65342564
AGMAT	-1.253468936	5.673380759	-5.659704257	0.001682349	0.435426513	-0.66905336
ADAMTS6	-2.603861867	6.37940822	-5.597761183	0.001774417	0.45153759	-0.71362751
MIOS	-1.715612043	8.810635255	-5.588076521	0.001789331	0.45153759	-0.72064854
TSC22D3	-1.154976834	6.840111464	-5.539242254	0.001866771	0.456815113	-0.75626736
PTPRG-AS1	-1.828148575	4.082547186	-5.527185226	0.001886481	0.456815113	-0.76511722
CASP4	-1.406411527	7.31220253	-5.518543166	0.001900756	0.456815113	-0.77147413
LGMN	2.603144188	9.484851417	5.463964719	0.001993841	0.473207074	-0.81188499
INAFM2	1.917902075	8.785307653	5.451493547	0.002015842	0.473207074	-0.8211832
RAC2	-1.423176815	6.794758413	-5.42591017	0.002061855	0.47844509	-0.84033293
LHX2	2.528157456	7.309622656	5.29365469	0.002319903	0.52389885	-0.94096238
CHRNA1	-1.118280473	4.651694783	-5.288518636	0.00233065	0.52389885	-0.94492603
ENPP1	-3.029056117	6.817281722	-5.286166978	0.00233559	0.52389885	-0.94674228
POSTN	-6.470171811	5.574060308	-5.239539302	0.002436069	0.537026404	-0.98293685
ENO1-AS1	1.376787267	5.759503053	5.234457729	0.002447317	0.537026404	-0.98690249

Symbol	Log FC	Ave Expression	t	P Value	adj. P. Value	B
SELENOW	-1.684032369	10.46693048	-5.150979795	0.002640912	0.573276762	-1.05264655
LTBP2	-4.224856072	8.448075966	-5.066198328	0.002855763	0.606864692	-1.12058338
PF4	-1.229751481	6.188751105	-5.053157628	0.002890561	0.607860878	-1.13113851
TNFAIP8L3	-1.554135217	5.455772235	-5.008249644	0.003014172	0.627320646	-1.16770371
CRYZL2P	-1.302631614	4.735098412	-4.980304842	0.00309415	0.63739495	-1.19062746
COL8A1	-3.021401469	6.209057409	-4.897871169	0.003344631	0.669963021	-1.25901766
ZIC2	5.35861455	8.891456453	4.895615022	0.003351806	0.669963021	-1.26090568
RUBCNL	-1.06537598	5.156266558	-4.867754338	0.003441882	0.672436507	-1.28429206
MFAP5	-2.457461863	4.69136931	-4.867138697	0.003443903	0.672436507	-1.28481033
ZNF43	-1.980596255	5.9882633	-4.861008187	0.003464107	0.672436507	-1.2899748
ABLIM1	2.846691763	8.356439141	4.801971988	0.003665779	0.701312862	-1.34003896
BAALC	-3.714428123	6.406207132	-4.756273183	0.003831143	0.72278461	-1.3792067
SLC8A1-AS1	-2.046128397	4.695898592	-4.746744389	0.00386669	0.72278461	-1.38741947
MAGI2-AS3	-1.25462033	6.4921052	-4.713064746	0.003995388	0.739989754	-1.41657473
GIPC2	-1.255816642	3.964593317	-4.685086669	0.004106031	0.753009835	-1.44094557
GPX4	1.502118093	11.79947832	4.676599082	0.004140286	0.753009835	-1.44836607
ANXA2P1	-1.184661791	6.844429218	-4.583714314	0.0045373	0.817848323	-1.53040616
GSTO1	-1.369300045	11.7560566	-4.557376717	0.004657663	0.832114231	-1.55394813
TET3	1.072499552	7.404026915	4.534404119	0.00476563	0.843934585	-1.57458368
SSR3	-1.437870812	10.06566326	-4.520911809	0.004830371	0.845674457	-1.58674755
GLRX	-2.640430242	10.20066223	-4.50469516	0.004909516	0.845674457	-1.60141085
EML6	1.634337452	6.442343275	4.462841285	0.005120705	0.863290719	-1.63947489
DNASE1L1	-1.311531525	8.478843012	-4.43187969	0.005283603	0.881532123	-1.66783698
SMYD3	-2.19470111	9.780015237	-4.401051587	0.005451689	0.894786173	-1.69624984
IGFBP3	-3.562901333	9.095936936	-4.360792488	0.00568044	0.913801051	-1.73361578

Symbol	Log FC	Ave Expression	t	P Value	adj. P. Value	B
FABP4	-1.401758528	4.747258044	-4.340519569	0.005799752	0.913801051	-1.75254405
ITPRIP	-1.811587813	7.347114166	-4.333932682	0.005839129	0.913801051	-1.75871027
LINC00960	1.29816169	6.532880137	4.310209262	0.005983492	0.919682241	-1.7809846
ZNF300P1	-1.822489037	4.648217283	-4.305379413	0.006013377	0.919682241	-1.78553211
EDNRA	-3.197825255	6.340819164	-4.270459943	0.006234565	0.93520229	-1.81853806
OSR1	-2.449565865	5.606094612	-4.267485467	0.006253829	0.93520229	-1.82135994
ERCC6	-1.104165707	6.852187159	-4.252870089	0.006349475	0.94098792	-1.83524922
ID3	3.245546619	10.6730358	4.230211331	0.00650106	0.944197151	-1.85686036
VAT1L	-2.506248693	5.764902488	-4.190427013	0.006777262	0.97466807	-1.89503533
DKFZP586I1420	1.412367239	7.986548519	4.186194754	0.00680742	0.97466807	-1.89911367
NEDD4L	1.534963869	7.490199018	4.165902652	0.006954148	0.981750621	-1.91871402
PLXDC2	-1.92307416	4.833797136	-4.130919456	0.007215605	0.988094054	-1.95268454
ABHD2	-1.305437514	8.360343025	-4.127232404	0.007243804	0.988094054	-1.95627814
NPC1	-1.112936604	6.510893586	-4.1104919	0.00737342	0.997409259	-1.97262622
SULT1B1	-1.242343899	6.610511585	-4.047506202	0.007885188	1	-2.03460435
MMP19	-1.515714881	7.345860859	-4.019665255	0.008124123	1	-2.06223644
SMAGP	-2.377586147	8.891070926	-4.016265362	0.008153859	1	-2.06562077
ARHGAP22	-2.486657171	6.15359719	-4.008114747	0.008225647	1	-2.07374291
ADGRL1	1.205488023	7.653712794	4.000064805	0.00829725	1	-2.08177695
TIMP3	-3.65963599	9.413704501	-3.920204286	0.009047079	1	-2.16213807
RPS6KA1	1.449467268	7.314756937	3.90707603	0.009177526	1	-2.17546318
SEMA7A	-1.928956306	7.611530531	-3.867369638	0.00958516	1	-2.2159619
HHIP	-2.82313888	5.750844236	-3.864585589	0.009614499	1	-2.21881261
MYLIP	2.111833101	7.456290022	3.862549402	0.009636021	1	-2.22089847
CALHM5	-1.318819256	6.916377886	-3.861206992	0.009650239	1	-2.22227406

Symbol	Log FC	Ave Expression	t	P Value	adj. P. Value	B
CMTM8	-2.729792633	8.232477755	-3.857585784	0.009688713	1	-2.22598645
ADAMTSL1	-1.594451791	5.380465623	-3.846065179	0.009812267	1	-2.2378135
TP53I3	-2.585658409	9.335086015	-3.827893685	0.010010777	1	-2.256519
RPS29	1.010767989	10.62804568	3.80920343	0.010219686	1	-2.27582315
DHCR7	-1.797806149	9.277486721	-3.789906244	0.010440545	1	-2.29582291
CLIP2	-1.709208744	6.648935082	-3.780775983	0.010546911	1	-2.30530992
KCNE4	-1.448343957	5.516861704	-3.771379414	0.010657654	1	-2.31508996
KLF10	1.351791569	10.20881338	3.77058635	0.01066706	1	-2.31591614
SMIM13	1.035660927	8.51574073	3.766802806	0.010712065	1	-2.31985932
FAM129A	2.389413222	9.262035587	3.763609968	0.010750209	1	-2.32318896
PYCARD	-2.27728052	6.30185507	-3.758474344	0.010811885	1	-2.32854863
CCDC80	-3.727740446	8.502264114	-3.726628414	0.011203335	1	-2.36189411
LINC00623	2.440211827	10.25314893	3.726047135	0.011210627	1	-2.36250452
UTP23	1.189821972	8.353301794	3.71858228	0.011304743	1	-2.37034914
LOC257396	1.066484479	6.090326439	3.714469965	0.01135697	1	-2.37467511
NOX4	-1.450477949	4.319261255	-3.701801789	0.011519567	1	-2.38802132
CCNA1	-2.083337291	5.79010475	-3.68501151	0.011739117	1	-2.40575643
LMCD1	-1.868867742	6.554038087	-3.683748927	0.011755816	1	-2.40709219
FBLIM1	-1.56695802	7.179344064	-3.673437088	0.0118932	1	-2.41801279
MSMO1	-1.545165803	12.11133761	-3.666587373	0.011985454	1	-2.42527784
LINC00312	-1.538841074	6.809943719	-3.660692217	0.012065495	1	-2.43153745
CORIN	-1.305062456	4.97864144	-3.650315254	0.012207849	1	-2.44257166
FAP	-4.417123457	8.29744864	-3.623815448	0.012580006	1	-2.47084075
SCN8A	-1.252455868	5.783893058	-3.619418069	0.012642984	1	-2.47554434
PROCR	-1.564150323	8.673247796	-3.618558919	0.01265533	1	-2.47646374

Symbol	Log FC	Ave Expression	t	P Value	adj. P. Value	B
FDFT1	-1.420813258	10.49037626	-3.598911151	0.012941383	1	-2.49752662
RHPN2	3.924384428	7.7849798	3.597434995	0.012963166	1	-2.49911198
SLIT2	-2.293326298	8.042108994	-3.588696434	0.013092957	1	-2.50850529
PTGFRN	2.078989233	9.072654286	3.547972036	0.013717334	1	-2.55246708
PARP12	1.119190855	8.198804516	3.538638743	0.013865079	1	-2.56258539
WDR63	-1.093147075	4.564645942	-3.528458473	0.014028265	1	-2.57364017
SYNE1	-1.397118352	5.658303481	-3.489735574	0.014668891	1	-2.61586309
SYNGR2	1.008666197	6.909489561	3.481433691	0.014810451	1	-2.62495105
FOXP1	-1.20679129	7.764070923	-3.470740962	0.014995033	1	-2.63667475
SLC26A4	-1.875675378	4.837942775	-3.455048901	0.015270582	1	-2.65391748
CCBE1	-3.156432264	7.080943768	-3.408855769	0.016115064	1	-2.70493438
CRLF1	-1.823223372	7.123887839	-3.407993288	0.016131319	1	-2.70589059
PELI2	1.214219989	6.123632988	3.399609756	0.016290277	1	-2.71519216
SLC7A11	1.516100488	8.618333137	3.394477893	0.016388441	1	-2.72089222
11-Sep	-1.877424685	8.812087641	-3.39404456	0.01639676	1	-2.72137375
ITGBL1	-3.864667835	7.748920637	-3.385963791	0.016552759	1	-2.73035942
LNPK	-1.158281345	8.347696342	-3.376851581	0.016730655	1	-2.74050607
PCOLCE2	3.187348667	9.867797471	3.366739948	0.016930558	1	-2.75178297
RPS6KA5	1.36012557	6.023459967	3.356100341	0.017143773	1	-2.76366838
COL10A1	-4.027544599	6.412445252	-3.346881314	0.017330938	1	-2.77398317
C17orf100	1.218762018	6.796418573	3.342886833	0.017412739	1	-2.77845712
ZDHHC9	-1.022791663	7.605273384	-3.333413903	0.017608451	1	-2.78907841
SUGCT	-3.314088689	8.14363597	-3.324057303	0.017804164	1	-2.79958486
ZNF469	-1.231960482	8.248474784	-3.323591997	0.017813959	1	-2.80010776
CHRDL1	-1.325505632	6.066228374	-3.322852445	0.017829541	1	-2.80093891

Symbol	Log FC	Ave Expression	t	P Value	adj. P. Value	B
OLMALINC	1.315108638	5.93709861	3.304480444	0.018221522	1	-2.82161752
INSIG1	-2.236552544	10.47070697	-3.30003261	0.018317853	1	-2.82663271
CNTNAP3P2	-1.054973801	4.174739629	-3.292392971	0.018484635	1	-2.83525494
GKAP1	1.519815365	6.400486607	3.2724824	0.018927282	1	-2.8577743
LINC01279	-4.373411561	7.256606357	-3.264595695	0.019105859	1	-2.86671347
HERC5	3.429590726	8.227987928	3.263344991	0.019134349	1	-2.86813208
CNIH3	-1.865214012	5.396895161	-3.261610284	0.019173943	1	-2.8701001
ENPP2	-3.386377385	7.822127723	-3.255294795	0.019318862	1	-2.87726944
CDH4	-1.054317371	5.824646237	-3.251578885	0.019404697	1	-2.88149097
C1orf54	-2.445245294	7.715006031	-3.239484527	0.019687008	1	-2.8952475
ACAT2	-1.120738482	11.6322194	-3.225666884	0.020015128	1	-2.91099501
ARHGAP18	-2.224636141	8.429746514	-3.211965866	0.020346471	1	-2.92664194
ITGA5	-2.419736211	8.9352072	-3.203965951	0.020542744	1	-2.93579289
THBS1	-2.798041378	7.445828242	-3.183607499	0.021051746	1	-2.95912962
TMEM150A	-1.060230138	7.007281536	-3.177643759	0.021203482	1	-2.9659791
DLX4	1.635893529	6.254474706	3.147071633	0.02200056	1	-3.00118572
ITGA4	1.967751384	6.687129626	3.143253945	0.022102398	1	-3.00559312
PRIM1	1.288445138	10.1520613	3.137926485	0.022245382	1	-3.01174755
GALNT1	-1.517189352	9.579275877	-3.135092126	0.02232187	1	-3.01502381
LOC728613	3.077120229	7.276252151	3.134660907	0.022333532	1	-3.01552237
BACE2	-3.683307995	6.698099745	-3.12839146	0.02250385	1	-3.02277445
MAP4K4	-1.934143825	9.044898033	-3.098107628	0.023346987	1	-3.05789598
C5orf34	1.222153781	8.864415138	3.096664184	0.023388035	1	-3.05957376
NR1D2	1.824139019	8.541164842	3.091505326	0.023535392	1	-3.06557291
KCTD21	-1.160364517	6.700968416	-3.086165639	0.023688993	1	-3.0717869

Symbol	Log FC	Ave Expression	t	P Value	adj. P. Value	B
RAD9A	1.305195901	8.274308777	3.077096266	0.023952419	1	-3.08235187
IGFBP4	-2.434134225	7.977327602	-3.069291549	0.024181697	1	-3.09145428
LOC100130987	1.35776884	7.430570033	3.061229827	0.024421065	1	-3.10086672
PP7080	1.711896793	7.943753404	3.052712297	0.024676804	1	-3.11082265
C21orf91	1.156057462	8.615839261	3.04234742	0.024991993	1	-3.12295353
CTSO	-1.546474425	6.803012564	-3.041603968	0.025014771	1	-3.12382431
PUS7L	-1.582337432	7.381416987	-3.03767814	0.025135428	1	-3.12842396
MED30	1.2286049	8.392435692	3.033565085	0.025262526	1	-3.13324558
CEP70	1.135138769	7.534932392	3.033094859	0.025277101	1	-3.13379699
CDC42EP3	-1.05192886	10.44134662	-3.019985195	0.025687204	1	-3.14918389
ARHGEF3	-2.167475609	7.286983304	-3.019809366	0.025692754	1	-3.14939045
CHST2	-1.600396958	5.901736444	-3.01523238	0.025837686	1	-3.15476897
SCD	-1.168373638	9.99525355	-2.995617012	0.026469067	1	-3.1778565
SULF1	-4.694262984	6.79630602	-2.982042678	0.02691591	1	-3.19386856
UAP1	-1.424093784	11.93920294	-2.975127564	0.027146721	1	-3.2020364
TGFBR2	-1.458889165	8.52179721	-2.974325684	0.027173627	1	-3.20298402
RNF144A	-1.086834403	6.159894782	-2.960226815	0.027651489	1	-3.21966135
RPL22L1	-2.299864199	10.96494973	-2.959464292	0.027677595	1	-3.22056418
DTD1	-1.127716415	8.397110905	-2.917918391	0.029141628	1	-3.26988691
PLXNA2	-1.120603698	6.104975768	-2.914347391	0.029271368	1	-3.27413831
BEX4	-2.100640032	4.809384517	-2.907137565	0.029535242	1	-3.28272757
COX7A1	-2.752861013	7.128142699	-2.904042	0.029649335	1	-3.28641773
C8orf48	-3.308377902	5.385153853	-2.903785353	0.029658816	1	-3.28672374
DNAJC18	-1.531001109	5.507001051	-2.899085552	0.029833019	1	-3.29232912
C1GALT1	-1.946245668	8.621002282	-2.893706394	0.030033778	1	-3.2987487

Symbol	Log FC	Ave Expression	t	P Value	adj. P. Value	B
WBP1L	-1.454164547	9.269495452	-2.889567602	0.030189249	1	-3.30369086
GSTK1	-1.668417331	9.154734607	-2.888070668	0.030245696	1	-3.30547896
CCDC8	-1.937138	6.043657113	-2.885879014	0.030328549	1	-3.3080975
SRPX2	-1.024047024	5.820580803	-2.882938135	0.030440116	1	-3.31161228
KLHL15	1.408179461	8.734532192	2.880798122	0.030521582	1	-3.31417069
WNT5B	-1.847601266	6.851344687	-2.862370428	0.031233024	1	-3.33622823
UGP2	-1.21795218	9.466585209	-2.857490395	0.031424447	1	-3.34207757
FNBP1L	1.610804755	10.07612331	2.856742494	0.031453896	1	-3.34297432
ANGPTL4	-1.290651258	7.19088287	-2.847483414	0.031820992	1	-3.35408267
GLT8D2	-2.051574647	8.899409195	-2.844459848	0.031941878	1	-3.35771272
KDELR3	-2.20869073	9.781113599	-2.841230882	0.032071529	1	-3.36159076
NEGR1	-1.43667507	5.297316369	-2.841021323	0.032079963	1	-3.3618425
LOC100996740	3.418884122	8.618357067	2.834473843	0.032344701	1	-3.36971076
SDCCAG8	-1.557958669	6.477912356	-2.828281311	0.03259728	1	-3.37715791
S100A4	3.536354392	12.43682174	2.822617242	0.032830186	1	-3.38397413
SLC38A5	-3.176500791	7.21864317	-2.812453027	0.033252696	1	-3.39621686
OLFML3	-4.062888346	8.692640172	-2.809484058	0.033377225	1	-3.3997956
TIMP1	-1.605767716	12.36412662	-2.801376432	0.033719883	1	-3.40957443
USP44	1.385091989	5.25591481	2.796433378	0.033930671	1	-3.4155407
LOXL3	-1.375000827	8.218598382	-2.783460473	0.034490726	1	-3.43121445
ROR1	-1.536095663	7.800906843	-2.782345288	0.034539337	1	-3.43256284
PRPF38A	1.137335344	9.200081191	2.777103234	0.034768838	1	-3.4389033
HIST1H2AM	1.423408703	7.362101958	2.775490198	0.034839791	1	-3.44085505
ACVR1	-1.038708127	10.14047522	-2.774721416	0.034873663	1	-3.44178539
ANO10	-1.064089283	8.135212188	-2.773996728	0.034905625	1	-3.44266243

Symbol	Log FC	Ave Expression	t	P Value	adj. P. Value	B
MT1H	-1.220443986	12.5634767	-2.767185809	0.035207577	1	-3.4509086
OLFML2B	-3.557095933	8.048340733	-2.76451357	0.03532682	1	-3.4541456
ID1	2.205395019	10.94354027	2.753369912	0.035828833	1	-3.46765427
THY1	-4.02111684	8.957407648	-2.752314133	0.035876796	1	-3.46893494
SPSB1	-1.392680344	7.36185121	-2.746907564	0.036123501	1	-3.47549538
NSDHL	-1.470305064	9.197381119	-2.74618071	0.036156808	1	-3.47637764
PPP2R3A	-1.792072192	8.729988328	-2.745608313	0.03618306	1	-3.47707247
LAPTM4B	1.218406912	12.06871331	2.73290484	0.036771051	1	-3.49250372
PHGDH	1.155203162	10.38498002	2.729232634	0.036942947	1	-3.4969682
LRIG3	2.196141386	7.479549247	2.723201717	0.037227147	1	-3.50430391
ZNF30	-1.324615718	6.460453407	-2.720739367	0.037343862	1	-3.50730028
APH1B	-1.107913196	7.980793514	-2.713647191	0.037682254	1	-3.51593473
CBX7	1.084853836	7.692392788	2.709165902	0.037897782	1	-3.52139368
DGKD	1.198180849	9.090141262	2.70374996	0.038160046	1	-3.52799445
IRAK4	-1.458941707	7.350582186	-2.690589973	0.038805533	1	-3.54404805
STK17B	-1.215260965	8.068142249	-2.682282446	0.039219086	1	-3.55419281
CXCL3	-1.777011222	6.806996982	-2.679263691	0.039370539	1	-3.55788118
NME5	-1.568507493	5.094829785	-2.678224724	0.039422811	1	-3.55915086
CAVIN1	-1.339521116	10.32807276	-2.674574943	0.039607033	1	-3.56361208
EPG5	-1.004464838	6.753970932	-2.669706558	0.039854213	1	-3.56956525
ANK2	-1.046501556	5.037329454	-2.663032473	0.040195785	1	-3.57773089
KCTD12	-5.257414514	8.67144097	-2.658413792	0.040434014	1	-3.58338475
ALX1	-1.266026301	7.293400516	-2.654442164	0.040640088	1	-3.58824848
FANCL	1.311754466	10.32041053	2.654119657	0.040656872	1	-3.5886435
SAMD9L	1.251340514	6.288809523	2.652535378	0.040739427	1	-3.59058419

Symbol	Log FC	Ave Expression	t	P Value	adj. P. Value	B
NAGS	-2.178856268	7.550993647	-2.649447713	0.040900844	1	-3.59436729
FABP3	-4.064725689	7.122462524	-2.648722362	0.040938864	1	-3.59525617
PLIN3	-1.394917079	10.67067597	-2.645348171	0.041116226	1	-3.59939182
ORMDL2	-1.084952004	10.44986598	-2.638551756	0.041475998	1	-3.60772583
VWA5A	-1.753238381	6.308306198	-2.637301176	0.041542568	1	-3.6092599
EVI2A	-2.320340387	7.546175148	-2.633696994	0.041735066	1	-3.61368204
GDNF	-1.000647324	7.192549686	-2.630591635	0.041901694	1	-3.61749329
KLHL23	1.542378207	7.460859399	2.628856677	0.041995101	1	-3.61962309
AP4M1	-1.268622321	8.14049153	-2.626082758	0.04214491	1	-3.62302897
UNG	1.195098922	10.34604999	2.624229251	0.042245332	1	-3.62530522
CEP85	1.015901522	8.544037783	2.611561074	0.042938628	1	-3.64087253
SLC31A2	-1.942978678	8.481915171	-2.60792998	0.0431396	1	-3.64533775
PAPSS2	-1.771978441	9.283381676	-2.604319941	0.043340412	1	-3.64977845
CDCP1	-1.320267396	5.937141667	-2.601504585	0.043497716	1	-3.65324256
HSD17B6	1.247273858	6.235696954	2.593836944	0.04392926	1	-3.66268125
CYTOR	-1.021799265	7.191819096	-2.581467137	0.044635183	1	-3.67792087
AGTR1	-3.259309023	5.26980881	-2.57922405	0.044764493	1	-3.68068601
HLTF	1.296236174	10.99004267	2.565888544	0.04554162	1	-3.69713554
HMGCS1	-1.584230122	9.859597836	-2.558763821	0.045962741	1	-3.70593113
ILF3-DT	1.54064473	9.02638303	2.554455632	0.046219412	1	-3.71125203
ZMAT3	-1.118179227	8.499043577	-2.553226516	0.046292921	1	-3.7127704
IFIT1	2.064669554	8.910440585	2.545498882	0.046757967	1	-3.72231988
HMGCR	-1.202614387	10.61191999	-2.542313525	0.046951116	1	-3.72625784
ACADM	1.201497238	11.08324412	2.542022155	0.046968826	1	-3.7266181
SOX11	-2.431777014	5.341628543	-2.534097502	0.047453268	1	-3.73641942

Symbol	Log FC	Ave Expression	t	P Value	adj. P. Value	B
C2orf68	1.397082563	7.727598732	2.531454077	0.047616052	1	-3.73969013
SLFN5	-1.477832658	7.102675456	-2.530253621	0.047690174	1	-3.74117567
TFB1M	-1.103712447	8.116661065	-2.529742195	0.04772179	1	-3.74180858
TMEM120A	-1.767715122	8.811887739	-2.522147264	0.048193949	1	-3.7512105
CD24	3.959982839	9.997633507	2.516578471	0.048543325	1	-3.7581075
ARHGAP28	1.206062209	6.084497902	2.510799093	0.048908782	1	-3.7652682
KLF6	-1.248364814	9.884328534	-2.506961644	0.049153068	1	-3.77002445
WNT5A	-2.186841844	10.32881676	-2.504796719	0.049291458	1	-3.77270828
ZBED8	1.580028526	8.078962996	2.501688281	0.049490892	1	-3.77656248
GAS6	-3.376351487	9.814521136	-2.494122784	0.049979899	1	-3.78594649
