# Supplementary material for: Integrated analysis of lncRNA-associated ceRNA network identified potential regulatory interactions in osteosarcoma
Source: Genet Mol Biol. 2020 May 20;43(2):e20190090. doi: 10.1590/1678-4685-GMB-2019-0090 (PMC7252519; doi:10.1590/1678-4685-GMB-2019-0090)
Supplement: Table S3 [file 1415-4757-GMB-43-2-e20190090-s4.pdf]

Genetics and Molecular Biology 43, 2, (2020)

Supplementary Material to "Integrated analysis of lncRNA-associated ceRNA network identified potential regulatory interactions in osteosarcoma"

Table S3 - Standardization and annotation miRNA data

miRNA	Log FC	Ave Expression	t	P Value	adj P Value	B
hsa-miR-424_st	-2.15806679	1.124370957	-11.6344903	5.57E-05	0.096460512	1.876569245
hsa-let-7a-2-star_st	-3.736499714	2.892516487	-9.756172094	0.000136772	0.096585869	1.337749858
hsa-miR-199b-5p_st	-3.331658363	1.422654905	-8.989122189	0.00020681	0.096585869	1.060707278
hsa-miR-323-3p_st	-2.463646453	1.450162838	-8.855820467	0.000222933	0.096585869	1.008481469
hsa-miR-4659a-5p_st	-1.205893787	0.873718481	-7.361210713	0.000558097	0.189813194	0.324409261
hsa-miR-493-star_st	-2.556456587	1.319980277	-7.119225927	0.000657172	0.189813194	0.194172728
hsa-miR-346_st	2.695513584	3.500217395	6.688350608	0.000889588	0.220236647	-0.053398662
hsa-miR-362-3p_st	-1.341820306	1.228715098	-5.8209174	0.001722853	0.373212991	-0.619824581
hsa-miR-1270_st	-2.635607341	1.361999879	-5.367741109	0.002509899	0.405907396	-0.956537574
hsa-miR-941_st	3.532968501	6.077739596	5.278938594	0.002709385	0.405907396	-1.026114139
hsa-miR-93_st	1.365904388	12.71566006	5.102734895	0.003162233	0.405907396	-1.167815347
hsa-miR-34a-star_st	-5.545578079	2.048070709	-5.086740191	0.003207515	0.405907396	-1.180922165
hsa-miR-16-2-star_st	-1.078855894	1.494091639	-5.06197543	0.003279113	0.405907396	-1.201296721
hsa-miR-769-3p_st	1.106719415	5.360195189	4.780044687	0.004239291	0.414004122	-1.440323224
hsa-miR-760_st	3.823891184	5.085488447	4.714698386	0.004505961	0.414004122	-1.497618917
hsa-miR-182_st	8.127984649	8.158802307	4.664732855	0.004722945	0.414004122	-1.541919186
hsa-miR-485-3p_st	-3.845486183	2.349274692	-4.517391966	0.005436287	0.436965075	-1.67505884
hsa-miR-3916_st	-1.14312543	1.424133006	-4.49649699	0.005547162	0.436965075	-1.694245658
hsa-miR-2276_st	1.812922587	4.554285291	4.346337433	0.006424922	0.481768633	-1.834392684
hsa-miR-106b-star_st	1.40000136	9.12293214	4.215109486	0.007324315	0.481768633	-1.96015872
hsa-miR-4436b-3p_st	-1.558348403	0.96768303	-4.203044538	0.007414003	0.481768633	-1.971876891
hsa-miR-151-5p_st	1.624041528	11.73992225	4.08417637	0.008368103	0.51792582	-2.08873692
hsa-miR-154_st	-4.187891835	1.845700744	-3.939377027	0.009725381	0.561802826	-2.234563882
hsa-miR-329_st	-2.920893608	1.561733243	-3.84796873	0.010710904	0.580062414	-2.328594674
hsa-miR-4539_st	2.937636121	3.743363314	3.788299272	0.011415378	0.599480299	-2.390801322
hsa-miR-29b-1-star_st	-2.55581428	6.659482331	-3.572265196	0.014442885	0.676473503	-2.621459232
hsa-miR-4288_st	-1.715610813	1.869459443	-3.528027827	0.015169275	0.691798757	-2.669735929
hsa-miR-1292_st	2.295457226	4.658056937	3.462719105	0.016318146	0.709473388	-2.741650539
hsa-miR-146b-5p_st	-1.172547235	1.326605348	-3.383098587	0.017853341	0.749524622	-2.830351697
hsa-miR-4728-5p_st	1.288030964	5.688377446	3.347223306	0.018597553	0.749524622	-2.870683536

miRNA	Log FC	Ave Expression	t	P Value	adj P Value	B
hsa-miR-744_st	1.559619331	10.14276253	3.295873949	0.019724023	0.759594045	-2.928801783
hsa-miR-758_st	-4.136642229	2.03354534	-3.238519557	0.021073416	0.777026173	-2.994253357
hsa-miR-331-5p_st	1.717073284	4.761919825	3.186315004	0.022391789	0.791938178	-3.054313131
hsa-miR-3607-5p_st	1.037972979	2.432651238	3.060796657	0.025954257	0.8002604	-3.200561103
hsa-miR-4486_st	1.583861981	4.924608723	3.049144866	0.026315679	0.8002604	-3.214266006
hsa-miR-3131_st	1.394921428	1.562763238	3.011378556	0.027526044	0.8002604	-3.258833421
hsa-miR-4449_st	2.454680537	6.785428074	2.952052768	0.02955376	0.8002604	-3.329286373
hsa-miR-151b_st	1.182321107	7.973054408	2.916424165	0.030850534	0.805491362	-3.371851487
hsa-miR-4423-3p_st	-1.70342391	1.765206235	-2.89813099	0.031540643	0.805491362	-3.393778469
hsa-miR-34a_st	-6.740539547	5.029442004	-2.833652095	0.034112094	0.825421307	-3.471446257
hsa-miR-551b-star_st	1.268907193	4.245158202	2.818708226	0.034740426	0.825421307	-3.489529404
hsa-miR-221_st	-1.156521455	13.10087728	-2.818021079	0.034769622	0.825421307	-3.490361631
hsa-miR-4443_st	1.451930293	7.523616399	2.802853127	0.035420996	0.829521427	-3.5087483
hsa-miR-495_st	-3.723709168	2.014241359	-2.772177801	0.036779606	0.849854105	-3.54602666
hsa-miR-1231_st	1.625902088	5.017238592	2.726950086	0.038887964	0.886747917	-3.601211833
hsa-miR-4784_st	-1.486751515	1.375223267	-2.644492221	0.043079693	0.900734425	-3.702467306
hsa-miR-543_st	-4.156732991	2.477112542	-2.628848604	0.043929153	0.900734425	-3.72176566
hsa-miR-3180-3p_st	1.51804523	4.659544769	2.619983259	0.044418617	0.900734425	-3.732714112
hsa-miR-486-5p_st	6.473041026	5.708524008	2.611720055	0.044880173	0.900734425	-3.742926591
hsa-miR-411-star_st	-2.455475632	1.719485925	-2.604284093	0.045299976	0.900734425	-3.752122921
hsa-miR-1208_st	1.064882465	2.767183865	2.599248842	0.045586663	0.900734425	-3.758353523
hsa-miR-4665-5p_st	2.683250979	3.824389757	2.597048453	0.045712562	0.900734425	-3.76107711
hsa-miR-377-star_st	-3.718509982	1.748810924	-2.58217054	0.046573783	0.901403323	-3.779505803
hsa-miR-1294_st	-1.530360184	1.438802354	-2.563096118	0.047703737	0.901403323	-3.803165457
