# Supplementary material for: Integrated analysis of lncRNA-associated ceRNA network identified potential regulatory interactions in osteosarcoma
Source: Genet Mol Biol. 2020 May 20;43(2):e20190090. doi: 10.1590/1678-4685-GMB-2019-0090 (PMC7252519; doi:10.1590/1678-4685-GMB-2019-0090)
Supplement: Table S4 [file 1415-4757-GMB-43-2-e20190090-s5.pdf]

Genetics and Molecular Biology 43, 2, (2020)

Supplementary Material to "Integrated analysis of lncRNA-associated ceRNA network identified potential regulatory interactions in osteosarcoma"

Table S4 - Interactions present in the ceRNA network.

miRNA	target	Interaction
hsa-miR-154-5p	CDC42EP3	miRNA-mRNA
hsa-miR-154-5p	KDELR3	miRNA-mRNA
hsa-miR-154-5p	KLF6	miRNA-mRNA
hsa-miR-154-5p	LRRC32	miRNA-mRNA
hsa-miR-154-5p	TMEM100	miRNA-mRNA
hsa-miR-154-5p	WNT5A	miRNA-mRNA
hsa-miR-154-5p	XG	miRNA-mRNA
hsa-miR-154-5p	MALAT1	miRNA-lncRNA
hsa-miR-154-5p	SNHG10	miRNA-lncRNA
hsa-miR-154-5p	NEAT1	miRNA-lncRNA
hsa-miR-154-5p	SNHG1	miRNA-lncRNA
hsa-miR-182-5p	ABLIM1	miRNA-mRNA
hsa-miR-182-5p	ACVR1	miRNA-mRNA
hsa-miR-182-5p	ARHGEF3	miRNA-mRNA
hsa-miR-182-5p	BACE2	miRNA-mRNA
hsa-miR-182-5p	C11orf45	miRNA-mRNA
hsa-miR-182-5p	C21orf91	miRNA-mRNA
hsa-miR-182-5p	CDC42EP3	miRNA-mRNA
hsa-miR-182-5p	DNAJC18	miRNA-mRNA
hsa-miR-182-5p	ERCC6	miRNA-mRNA
hsa-miR-182-5p	GALNT1	miRNA-mRNA
hsa-miR-182-5p	GDNF	miRNA-mRNA
hsa-miR-182-5p	MVD	miRNA-mRNA
hsa-miR-182-5p	PTGS1	miRNA-mRNA
hsa-miR-182-5p	SCN9A	miRNA-mRNA
hsa-miR-182-5p	SLC7A11	miRNA-mRNA
hsa-miR-182-5p	SOX11	miRNA-mRNA
hsa-miR-182-5p	TAGLN3	miRNA-mRNA
hsa-miR-182-5p	TENM4	miRNA-mRNA
hsa-miR-182-5p	VAT1L	miRNA-mRNA

miRNA	target	Interaction
hsa-miR-182-5p	WNT5A	miRNA-mRNA
hsa-miR-182-5p	ZMAT3	miRNA-mRNA
hsa-miR-182-5p	SNHG1	miRNA-lncRNA
hsa-miR-182-5p	XIST	miRNA-lncRNA
hsa-miR-199b-5p	C11orf45	miRNA-mRNA
hsa-miR-199b-5p	CDC42EP3	miRNA-mRNA
hsa-miR-199b-5p	CDCP1	miRNA-mRNA
hsa-miR-199b-5p	DHCR7	miRNA-mRNA
hsa-miR-199b-5p	DNASE1L1	miRNA-mRNA
hsa-miR-199b-5p	IGFBP3	miRNA-mRNA
hsa-miR-199b-5p	KCTD21	miRNA-mRNA
hsa-miR-199b-5p	KLHL23	miRNA-mRNA
hsa-miR-199b-5p	PLXNA2	miRNA-mRNA
hsa-miR-199b-5p	PTGS1	miRNA-mRNA
hsa-miR-199b-5p	SLC7A11	miRNA-mRNA
hsa-miR-199b-5p	SOX11	miRNA-mRNA
hsa-miR-199b-5p	UNG	miRNA-mRNA
hsa-miR-199b-5p	USP44	miRNA-mRNA
hsa-miR-199b-5p	SNHG12	miRNA-lncRNA
hsa-miR-34a-5p	AGTR1	miRNA-mRNA
hsa-miR-34a-5p	DHCR7	miRNA-mRNA
hsa-miR-34a-5p	ENPP1	miRNA-mRNA
hsa-miR-34a-5p	EPG5	miRNA-mRNA
hsa-miR-34a-5p	FAM129A	miRNA-mRNA
hsa-miR-34a-5p	FOXP1	miRNA-mRNA
hsa-miR-34a-5p	GALNT10	miRNA-mRNA
hsa-miR-34a-5p	HMGCS1	miRNA-mRNA
hsa-miR-34a-5p	IRAK4	miRNA-mRNA
hsa-miR-34a-5p	KLF6	miRNA-mRNA
hsa-miR-34a-5p	LOXL3	miRNA-mRNA
hsa-miR-34a-5p	NEDD4L	miRNA-mRNA
hsa-miR-34a-5p	P4HA3	miRNA-mRNA
hsa-miR-34a-5p	PLXNA2	miRNA-mRNA
hsa-miR-34a-5p	PPP2R3A	miRNA-mRNA
hsa-miR-34a-5p	PTGS1	miRNA-mRNA
hsa-miR-34a-5p	RAD9A	miRNA-mRNA
hsa-miR-34a-5p	RNF144A	miRNA-mRNA
hsa-miR-34a-5p	ROR1	miRNA-mRNA
hsa-miR-34a-5p	TENM4	miRNA-mRNA
hsa-miR-34a-5p	TGFBR2	miRNA-mRNA
hsa-miR-34a-5p	UNG	miRNA-mRNA
hsa-miR-34a-5p	VWA5A	miRNA-mRNA
hsa-miR-34a-5p	ZMAT3	miRNA-mRNA

miRNA	target	Interaction
hsa-miR-34a-5p	HCG18	miRNA-lncRNA
hsa-miR-34a-5p	SNHG7	miRNA-lncRNA
hsa-miR-34a-5p	XIST	miRNA-lncRNA
hsa-miR-34a-5p	KCNQ1OT1	miRNA-lncRNA
hsa-miR-486-5p	ADAMTSL1	miRNA-mRNA
hsa-miR-486-5p	BACE2	miRNA-mRNA
hsa-miR-486-5p	CLIP2	miRNA-mRNA
hsa-miR-486-5p	FBLIM1	miRNA-mRNA
hsa-miR-486-5p	FOXP1	miRNA-mRNA
hsa-miR-486-5p	LTBP2	miRNA-mRNA
hsa-miR-486-5p	NETO1	miRNA-mRNA
hsa-miR-486-5p	TNFSF4	miRNA-mRNA
hsa-miR-486-5p	ZMAT3	miRNA-mRNA
hsa-miR-486-5p	XIST	miRNA-lncRNA
hsa-miR-486-5p	KCNQ1OT1	miRNA-lncRNA
hsa-miR-495-3p	ABHD2	miRNA-mRNA
hsa-miR-495-3p	APH1B	miRNA-mRNA
hsa-miR-495-3p	ARHGAP28	miRNA-mRNA
hsa-miR-495-3p	C21orf91	miRNA-mRNA
hsa-miR-495-3p	CDCP1	miRNA-mRNA
hsa-miR-495-3p	CORIN	miRNA-mRNA
hsa-miR-495-3p	CTSO	miRNA-mRNA
hsa-miR-495-3p	CXCL5	miRNA-mRNA
hsa-miR-495-3p	DGKD	miRNA-mRNA
hsa-miR-495-3p	DOCK4	miRNA-mRNA
hsa-miR-495-3p	ERCC6	miRNA-mRNA
hsa-miR-495-3p	FAM129A	miRNA-mRNA
hsa-miR-495-3p	FNBP1L	miRNA-mRNA
hsa-miR-495-3p	FOXP1	miRNA-mRNA
hsa-miR-495-3p	IGFBP3	miRNA-mRNA
hsa-miR-495-3p	INSIG1	miRNA-mRNA
hsa-miR-495-3p	KCTD12	miRNA-mRNA
hsa-miR-495-3p	KLHL15	miRNA-mRNA
hsa-miR-495-3p	MDFIC	miRNA-mRNA
hsa-miR-495-3p	NEDD4L	miRNA-mRNA
hsa-miR-495-3p	PTGFRN	miRNA-mRNA
hsa-miR-495-3p	SCD	miRNA-mRNA
hsa-miR-495-3p	SLC7A11	miRNA-mRNA
hsa-miR-495-3p	TACSTD2	miRNA-mRNA
hsa-miR-495-3p	TSC22D3	miRNA-mRNA
hsa-miR-495-3p	UAP1	miRNA-mRNA
hsa-miR-495-3p	UNG	miRNA-mRNA
hsa-miR-495-3p	WBP1L	miRNA-mRNA

miRNA	target	Interaction
hsa-miR-495-3p	ZMAT3	miRNA-mRNA
hsa-miR-495-3p	NEAT1	miRNA-lncRNA
hsa-miR-543	ADAMTSL1	miRNA-mRNA
hsa-miR-543	APH1B	miRNA-mRNA
hsa-miR-543	ARHGEF3	miRNA-mRNA
hsa-miR-543	C1GALT1	miRNA-mRNA
hsa-miR-543	C21orf91	miRNA-mRNA
hsa-miR-543	C5orf30	miRNA-mRNA
hsa-miR-543	CBX7	miRNA-mRNA
hsa-miR-543	CDC42EP3	miRNA-mRNA
hsa-miR-543	EML6	miRNA-mRNA
hsa-miR-543	EPG5	miRNA-mRNA
hsa-miR-543	GALNT1	miRNA-mRNA
hsa-miR-543	IGFBP3	miRNA-mRNA
hsa-miR-543	KLF6	miRNA-mRNA
hsa-miR-543	MDFIC	miRNA-mRNA
hsa-miR-543	NR1D2	miRNA-mRNA
hsa-miR-543	POSTN	miRNA-mRNA
hsa-miR-543	PROCR	miRNA-mRNA
hsa-miR-543	RPS6KA5	miRNA-mRNA
hsa-miR-543	SCN9A	miRNA-mRNA
hsa-miR-543	SLC7A11	miRNA-mRNA
hsa-miR-543	STK17B	miRNA-mRNA
hsa-miR-543	TNFSF4	miRNA-mRNA
hsa-miR-543	UTP23	miRNA-mRNA
hsa-miR-543	WNT5B	miRNA-mRNA
hsa-miR-543	ZIC2	miRNA-mRNA
hsa-miR-543	NEAT1	miRNA-lncRNA
hsa-miR-543	SNHG7	miRNA-lncRNA
